# Unified Total Synthesis of Phrymarolin and Haedoxan Natural Products

**DOI:** 10.1021/jacs.5c16676

**Published:** 2025-12-04

**Authors:** Jan Paciorek, Antoine D. F. Guy, Alexander Sudau, David M. Barber, Thomas Magauer

**Affiliations:** Department of Organic Chemistry and Center for Molecular Biosciences, University of Innsbruck, 6020 Innsbruck, Austria; Research and Development, Profile Driven Chemistry, Bayer AG, Crop Science Division, 40789 Monheim am Rhein, Germany; Research and Development, Profile Driven Chemistry, Bayer AG, Crop Science Division, 65926 Frankfurt am Main, Germany; Department of Organic Chemistry and Center for Molecular Biosciences, University of Innsbruck, 6020 Innsbruck, Austria

## Abstract

We disclose a unified synthetic route to the furofuran lignans phrymarolin I and II as well as the insecticidal natural products haedoxan A and D. The furofuran core was constructed using a formal [3 + 2] cycloaddition between an *α*-silyloxy aldehyde and a styrene, followed by a samarium(II) iodide promoted-cyclization of a *β*-formyloxy ketone. While this sequence enabled the synthesis of phrymarolin I and II in eight steps, attempts to unmask the *ortho*-quinone necessary for a bioinspired formal [4 + 2] cycloaddition were unsuccessful, initially preventing access to the haedoxans. Revising the choice of the arene substitution pattern enabled the formation of the requisite *ortho*-quinone followed by a bioinspired cyclization to the 1,4-benzodioxane motif of the haedoxan framework. Finally, late-stage diversification at the acetal position enabled completion of the synthesis of haedoxan A and D and their analogues in 13 steps. The synthetic route facilitated insecticidal screening of fully synthetic analogues modified at the *O*-aryl residue.

**Figure:**
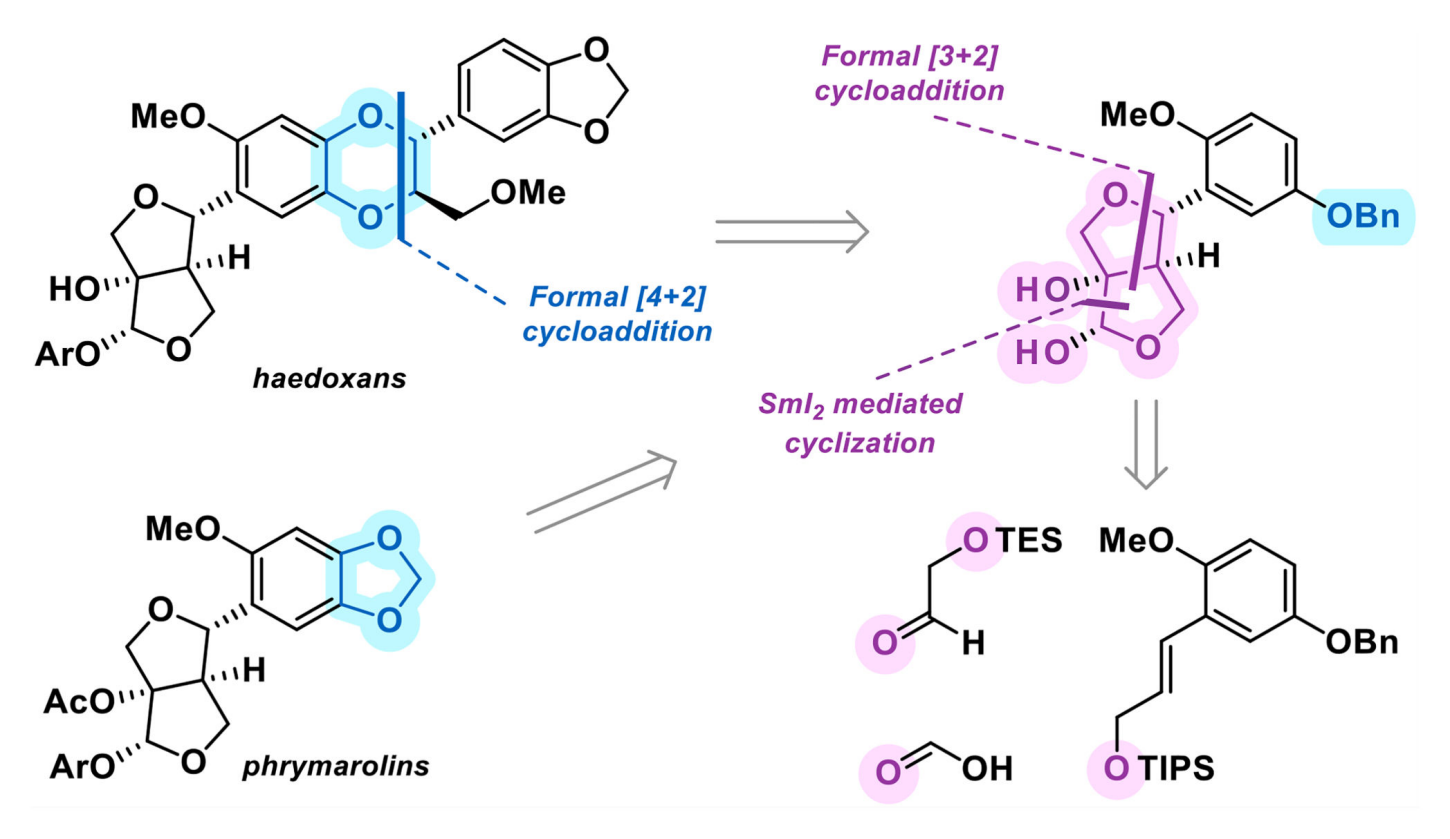

## Introduction

**Figure 1:**
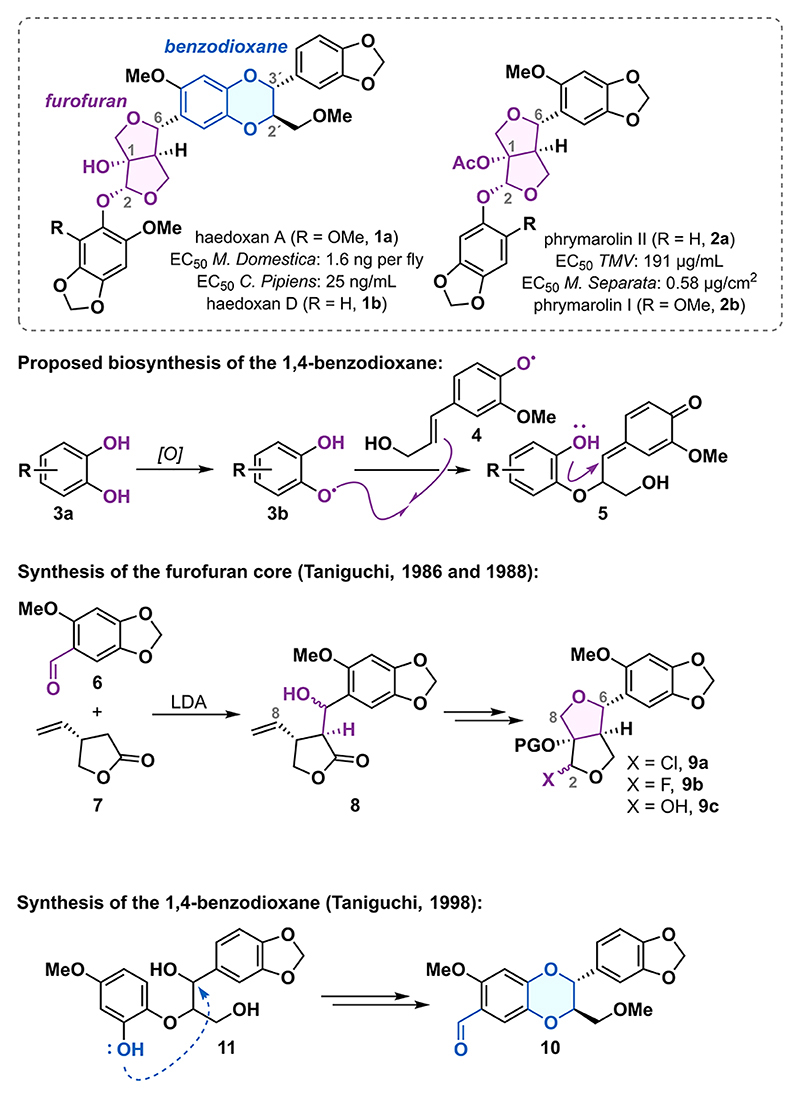
Introduction and selected prior art.

Lignans are a vast and structurally diverse family of natural products ubiquitous in the plant kingdom.^1^ The furofuran lignans represent one of the largest subclasses, characterized by a central 3,7-dioxa[3.3.0]bicyclooctane ring system, commonly termed the furofuran (Figure 1). This bicyclic core is typically substituted at the C2 and C6 positions with aryl residues featuring diverse combinations of hydroxy, methoxy, and methylenedioxy substituents. Some furofuran lignans undergo further oxidation either at the C1 position, in the form of a hydroxy or an acetoxy group,^2^ or at the C2 position, resulting in a phenolic acetal motif.^3,4^ Natural products isolated from the herbal lopseed species *Phryma leptostachya*, namely haedoxans A and D (**1a** and **1d**) and the phrymarolins I and II (**2b** and **2a**), possess both of these additional oxidation patterns.^5−7^ Another structural feature characteristic of the haedoxans is their 1,4-benzodioxane ring system functionalized with methoxymethyl and aryl residues at the C2′and C3′ positions, respectively. The biosynthetic origin of the 1,4-benzodioxane motif has not been thoroughly investigated; however, it is believed to arise, similarly to other 1,4-benzodioxane-containing natural products,^8^ from a cascade in which a catechol **3a** is enzymatically oxidized to an *O*-centered radical **3b**, which then couples with another radical derived from a styrene, most commonly coniferyl alcohol (**4**).^9,10^ In the second step, the *para*-quinone methide intermediate **5** cyclizes to form the 1,4-benzodioxane motif.

Aside from their appealing structure, the natural products isolated from lopseed *Phryma leptostachya* possess remarkable biological activities. Phrymarolins and their unnatural analogues were shown to exhibit antibacterial and antiviral properties. Their activity against tobacco mosaic virus (TMV) was recently reported (EC_50_ = 191 *μ*g/mL for phrymarolin II) highlighting their potential for crop protection.^11^ Additionally, some phrymarolin natural products recently isolated from *Phryma leptostachya* were tested as insecticides with EC_50_ values ranging from 0.58 to 10.1 *μ*g/cm^2^.^12^ Haedoxan A (**1a**) exhibits extraordinary insecticidal activity against a range of pests such as *Culex pipiens* (LC_50_ = 25 ng/mL) and *Musca domestica* (LC_50_ = 1.6 ng per fly).^7,13,14^ It also showed low risk of resistance development in *Aedes aegypti* mosquitos,^15^ making it an intriguing lead compound for the development of highly potent pest control agents.

Phrymarolin II and I (**2a** and **2b**), the first natural products of this class accessed by synthesis, were prepared by the group of Taniguchi in 1986 and 1988, respectively.^16,17^ The synthesis of the furofuran core began with an aldol reaction of aldehyde **6** with lactone **7** (Figure 1). Alcohol **8** was elaborated via a sequence of redox manipulations followed by an acid-catalyzed tetrahydrofuran ring cyclization to yield glycosyl donor **9a** (X = Cl) and **9b** (X = F). Reaction of those donors with the corresponding phenol gave a mixture of C2 and C6 epimeric acetals. In another synthesis of **2b**, an acid-catalyzed condensation between diol **9c** (X = OH) and a phenol also delivered a mixture of acetals, affording the desired diastereomer in low yield.^18^ In 1989, Taniguchi published a racemic synthesis of **1a** followed by an enantioselective sequence to the natural product in 1998.^19^ Both were designed on the basis of the phrymarolin synthesis, with the difference being that aldehyde **10** was used in the aldol reaction. The 1,4-benzodioxane ring system of aldehyde **10** was installed via an acid-catalyzed *O*-alkylation of **11**.^20^

More recently, the total synthesis of **2a** was reported by the Gao group.^11a^ Their synthetic strategy was considerably more efficient than the one developed by Taniguchi et al., as it enabled synthesis of the furofuran core in only five steps. The phenolic acetal was introduced by a Chan−Lam coupling, which avoided the acid-catalyzed acetalization and facilitated the synthesis of unnatural analogues of **2a**. However, the coupling failed to deliver any product when *ortho*-substituents on the boronic acid fragment were present.

Several synthetic analogues of **1a** have been synthesized and evaluated in structure−activity relationship (SAR) studies.^21^ The SAR studies primarily targeted modifications of the 1,4-benzodioxane fragment, revealing its essential role in insecticidal activity. None of the unnatural analogues surpassed the potency of the natural substitution pattern found in **1a**.

Herein, we report the development of a highly convergent strategy that enabled the synthesis of phrymarolin and haedoxan natural products, while also facilitating access to unnatural analogues for exploration of the SAR of the haedoxan acetal motif.

### Retrosynthetic Analysis

**Scheme 1:**
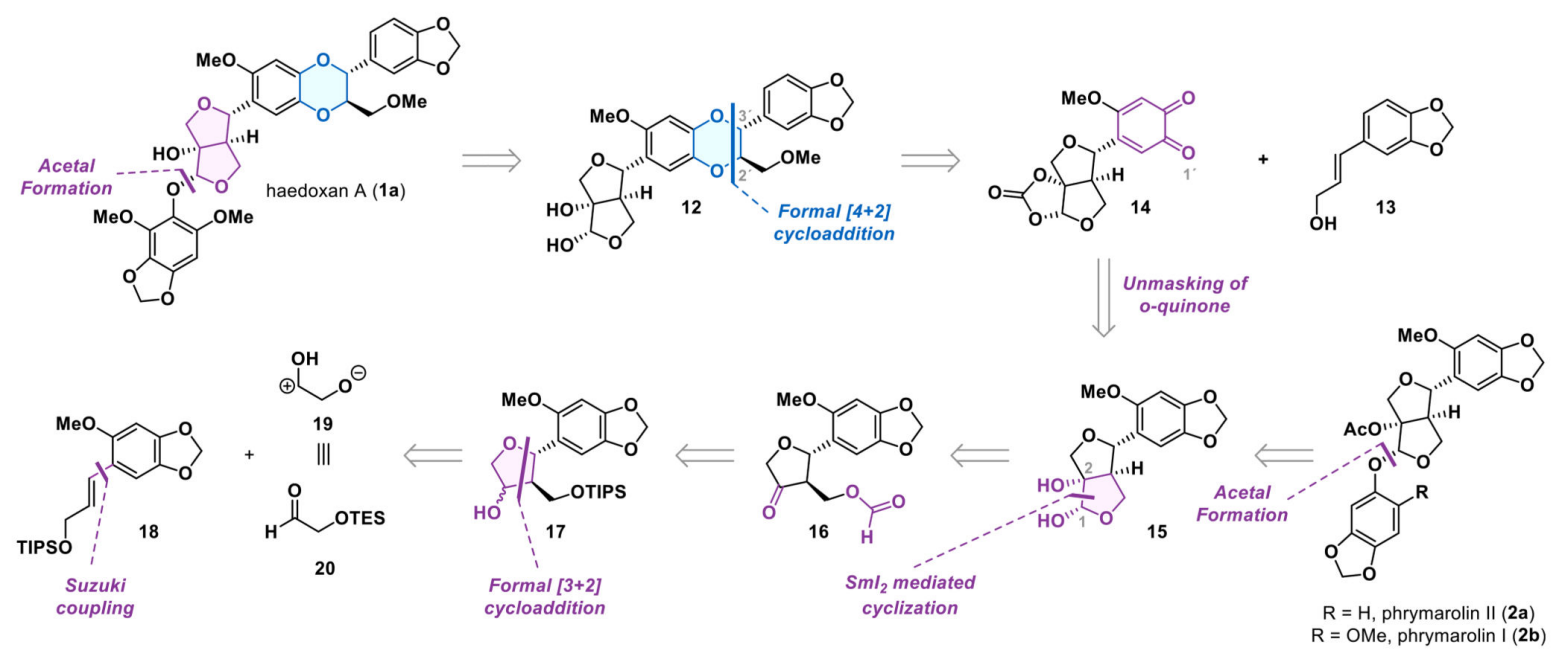
Unified Retrosynthetic Analysis for Phrymarolins I and II and Haedoxan A

In our initial retrosynthetic plan, we simplified haedoxan A (**1a**) to diol **12** by first cutting the acetal bond (Scheme 1).^18,19^ Next, we recognized an opportunity to forge the 1,4-benzodioxane fragment by leveraging the reactivity proposed in the biosynthesis of 1,4-benzodioxane natural products (Figure 1). A retrosynthetic cut of this motif produced styrene **13** and *ortho*-quinone **14**. The latter would serve as a precursor to a catechol-derived radical analogous to radical **3b**. Regarding the regioselectivity of the radical attack on the styrene, we expected this to be directed by the aromatic methoxy substituent, which stabilizes the radical centered at the O1′ oxygen. Based on the seminal work of Merlini,^22^ we expected this transformation to predominantly produce the desired relative *trans*-configuration of the C2′ and C3′ substituents. For the introduction of the *ortho*-quinone functionality, we opted for the oxidation of the 1,3-benzodioxole motif in diol **15**.^23^

Disconnection of the C1−C2 bond in diol **15** led to *β*-formyloxy ketone **16**. In the forward sense, this corresponds to a reductive cyclization initiated by exposing **16** to samarium-(II) iodide. We envisioned the carbon−carbon bond formation to be highly stereoselective due to the inherently high barrier leading to the undesired *trans*-fused [5,5]-bicyclic ring system.^24^ We also expected a high level of stereocontrol for the hemiacetal stereocenter owing to the possibility for the samarium counterion to coordinate to the ester in the reduced intermediate.^25^ Through several functional group interconversions, we traced *β*-formyloxy ketone **16** back to alcohol **17**. Disconnecting the tetrahydrofuran ring in **17** via a retro [3 + 2] cycloaddition revealed styrene **18** and dipolar synthon **19**, the synthetic equivalent of which was found in aldehyde **20**.^26^

## Results and Discussion

### Synthesis of Phrymarolin Natural Products

**Scheme 2:**
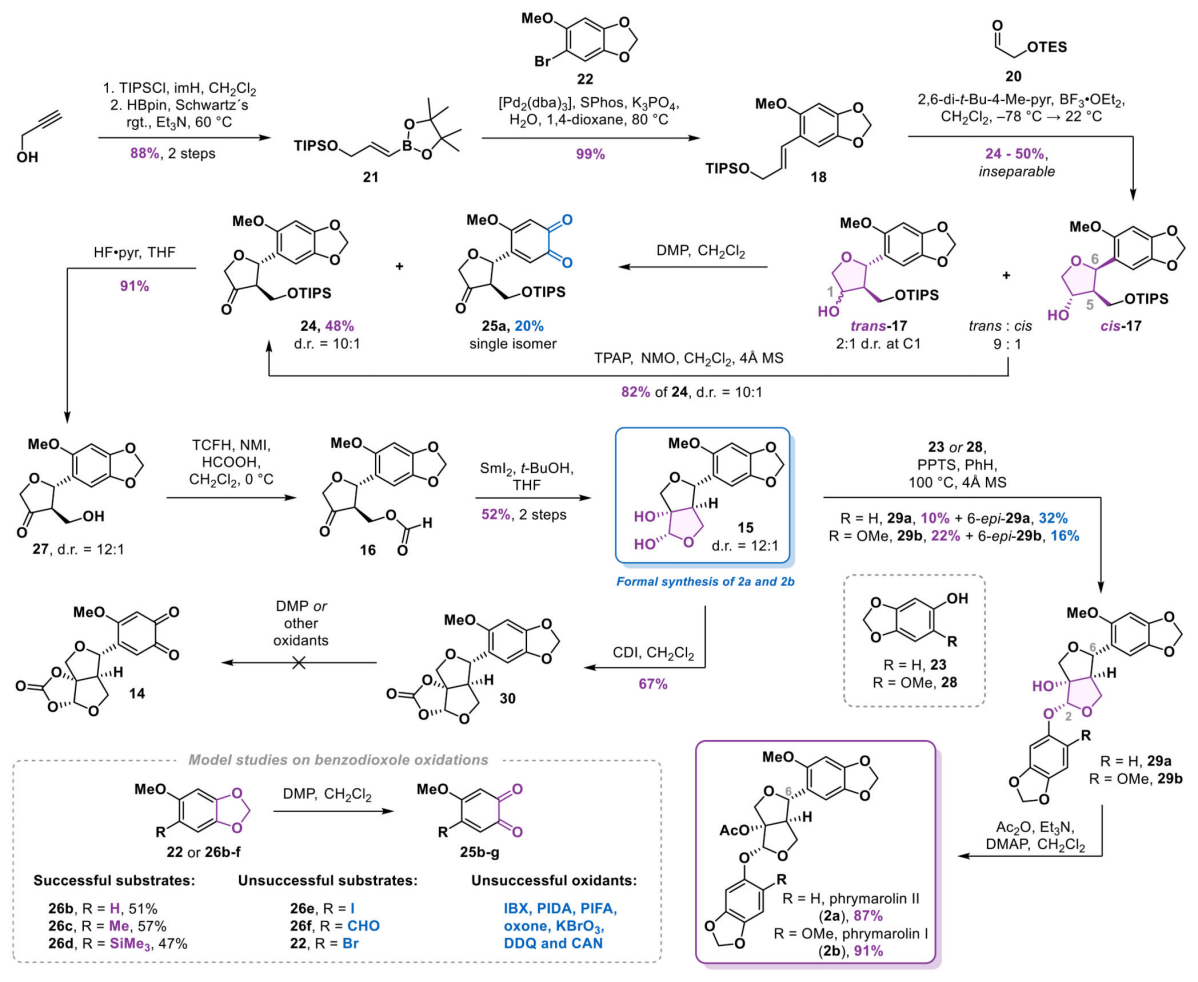
Initial Studies toward the Furofuran Core and Total Synthesis of Phrymarolins I and II

We began our endeavor with the synthesis of styrene **18** (Scheme 2). Toward this end, we synthesized boronic ester **21** from propargyl alcohol by protecting it as a triisopropylsilyl (TIPS) ether followed by hydroboration with pinacolborane catalyzed by Schwartz′s reagent.^27^ This two-step procedure yielded the boronic ester **21** in an 88% overall yield. The aryl bromide fragment **22** for the Suzuki coupling was synthesized following a two-step protocol consisting of *O*-methylation of sesamol (**23**) followed by bromination with *N*-bromosuccinimide (NBS) to give **22** in 99% overall yield. The two fragments were then unified in a highly efficient Suzuki cross-coupling, giving the styrene **18** in 99% yield.

Aldehyde **20** was synthesized in two steps from *cis*-but-2-ene-1,4-diol by a sequence involving triethylsilyl (TES) protection and ozonolysis (83% over two steps). With both components in hand, we performed the formal cycloaddition according to the protocol by Angle.^26^ Upon exposure of the mixture of styrene **18** and aldehyde **20** to the reported conditions (BF_3_·OEt_2_, CH_2_Cl_2_, −78 to 22 °C), alcohol **17** was obtained as a mixture of three isomers in up to 50% yield. The slight improvement in the yield at −90 °C proved to be irreproducible. While alternative Lewis acids such as BCl_3_, B(C_5_F_5_)_3_, SnCl_4_, and TiCl_4_ failed to promote the desired transformation, a more comprehensive screening would be required to draw a definitive conclusion. This should include the assessment of chiral Lewis acids to achieve control of absolute stereochemistry, which remains unachieved. The two desired *trans-*isomers of **17** (*trans* referring to relative configurations at C5 and C6) were diastereomeric at the C1 position, which was inconsequential for the following oxidation. The *cis*-isomer of **17** represented nearly 10% of the isomeric mixture and proved to be chromatographically inseparable.

We continued the synthesis by oxidizing the mixture of alcohols **17**. When using Ley−Griffith conditions (TPAP, NMO), clean conversion to ketone **24** was observed (82% yield). Surprisingly, reacting **17** with Dess−Martin periodinane (DMP) led to oxidation of 1,3-benzodioxole and formation of *ortho*-quinone **25a**. The amount of **25a** varied between different batches of DMP and never exceeded 20% even when a large excess of the reagent. Interestingly, submitting ketone **24** to the DMP oxidation conditions resulted in a negligible conversion to quinone **25a**. This suggested that the quinone formation predominately occurred from alcohol diastereomers **17**, making the oxidation highly sensitive to changes in the substituents. Out of curiosity, we synthesized several 1,3-benzodioxoles to explore the scope of this transformation. While hydrogen, methyl, and trimethylsilyl substituents allowed for oxidation of the 1,3-benzodioxoles **26** into *ortho*-quinones **25** with yields close to 50%, those containing halogens or the electron-withdrawing aldehyde completely lost reactivity. Among all oxidants tested (Scheme 2), only DMP was capable of effecting the transformation.

After this short exploration of unexpected reactivity, we continued with the synthesis of the bicyclic core. Ketone **24** was subjected to silyl deprotection using a hydrogen fluoride-pyridine complex (HF·pyr) which smoothly delivered the primary alcohol **27** in 91% yield. The first attempt to transform the alcohol into the formate ester **16** (HCOOH, EDC, DMAP, Et_3_N) resulted in elimination of the *β*-alcohol, and only the corresponding enone (not shown) was isolated. By using tetramethylformamidinium hexafluorophosphate (TCFH) and *N*-methylimidazole (NMI), elimination was fully suppressed. Since chromatographic purification also resulted in elimination of the format ester, crude ester **16** was telescoped into the samarium(II) iodide-mediated cyclization. To our delight, using an excess of SmI_2_ in the presence of *t*-BuOH delivered the diol **15** in 52% yield over two steps.^28^ With diol **15** in hand, we intercepted, for the first time, the known racemic synthesis of the phrymarolins (nine steps from sesamol), in which the C5 and C6 stereocenters were introduced simultaneously via a highly diastereoselective Barbier-type allylation.^11,18^ Transforming diol **15** into the corresponding acetals, according to the literature,^18,19^ proceeded in low yields mostly due to the facile acid-catalyzed epimerization of the C6 position and incomplete conversion of diol **15**. Pushing the equilibrium toward the acetal product was possible using ten equiv of either phenol **23** or phenol **28** yielding acetal **29a** in 10% and acetal **29b** in 22% yield, respectively. The natural products phrymarolin II (**2a**) and phrymarolin I (**2b**) were then obtained by the acetylation of the tertiary alcohol. The analytical data of the synthetic compounds fully matched the data reported in the literature.^5,11,18^ We also briefly explored the possibility to convert diol **15** into the acetals via transition metal catalysis;^29^ however, as the *O*-arylation protocols typically require harsh conditions (e.g., CuI, Cs_2_CO_3_, PhMe, 110 °C, 30 h),^29c^ only decomposition of **15** was observed.

Given our prior success in oxidizing 1,3-benzodioxoles with specific substitution patterns to *ortho*-quinones using DMP, we sought to apply this methodology to diol **15** to access *ortho*-quinone **14**, a potential substrate for the envisioned bioinspired formal [4 + 2] cycloaddition en route to haedoxan natural products. Toward this end, the diol moiety in **15** was protected as a cyclic carbonate affording intermediate **30** in 67% yield. Subjecting this compound to a reaction with DMP or other oxidants, previously tested in the oxidation of 1,3-benzodioxols **26**, failed to deliver the desired *ortho*-quinone **14**. Given the poor performance of the formal [3 + 2] cycloaddition and our inability to advance the synthesis of haedoxan A, we decided to redesign our route to address these issues.

### Improved Furofuran Core Synthesis

**Scheme 3:**
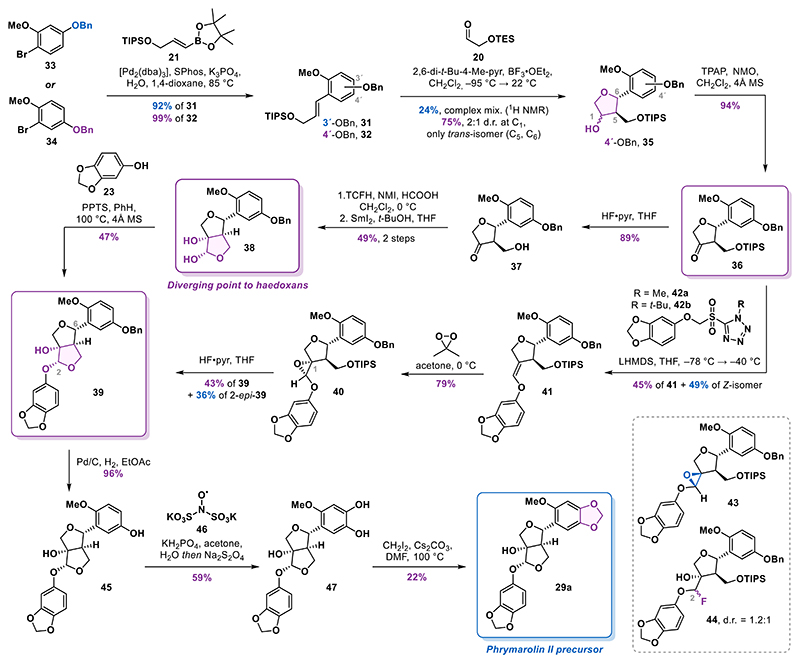
Improved Synthesis of the Furofuran Core and Alternative Route to Phrymarolins

Our revised strategy focused on designing a more suitable substrate for the formal [3 + 2] cycloaddition, aiming for incorporating a functional handle on the aromatic ring to enable efficient introduction of the quinone moiety. In the original study by Angle,^26^ a cyclization reaction analogous to that of styrene **18** was reported to afford the corresponding alcohol in 74% yield, with no detectable *cis*-isomer. This outcome is somewhat unexpected, as several other styrenes lacking the OTIPS substituent produced *trans*/*cis* mixture with ratios up to 12:1. However, those substrates contained only one or two oxygen substituents, in contrast to the three present in styrene **18**. We reasoned that the increased electron density within the conjugated system could substantially influence the reactivity of the intermediate benzylic cation formed during the reaction. Furthermore, the higher electron density may promote a reversible THF ring opening, leading to epimerization of the initially formed *trans*-isomer to the observed *trans*/*cis* mixture. Based on this rationale, we designed styrenes **31** and **32**, each incorporating a benzyloxy substituent (Scheme 3). On one hand, this modification not only reduced the electron density of the aromatic system compared to styrene **18** but also enabled unmasking of a phenol at a later stage that could subsequently be transformed into the *ortho*-quinone motif using established oxidation methods.^30,31^

The styrenes **31** and **32** were synthesized in high yields using a Suzuki cross-coupling reaction of boronic ester **21** with aryl bromides **33** and **34**, respectively. We observed a dramatic difference in the results of the formal [3 + 2] cycloaddition of these two substrates. Styrene **31**, bearing the benzyloxy group in the *para*-position with respect to the olefin, gave only 24% yield (estimated by NMR) of the corresponding product among a complex mixture of side products. In contrast, the regioisomeric styrene **32** was cleanly converted into the desired alcohols **35** in 75% yield, again as an inconsequential 2:1 mixture of C1 diastereomers, and notably, without any detectable traces of the undesired *cis*-isomer. Encouraged by this result, we subjected the mixture of alcohols to a Ley−Griffith oxidation to deliver ketone **36** in 94% yield. The ketone was then deprotected to furnish alcohol **37** in an 89% yield. The two-step sequence involving formylation and samarium(II) iodide-mediated cyclization afforded diol **38** in 49% yield over two steps as a single isomer. Starting from aryl bromide **34**, the developed sequence delivered diol **38** in 30% yield over six steps. This represents a significant improvement compared with the average 14% yield for the synthesis of diol **15** from aryl bromide **22** in the previous strategy.

Continuing with the synthesis of phrymarolins, the acidcatalyzed condensation of diol **38** with sesamol (**23**) afforded acetal **39** in 47% yield. Notably, C6 epimerization was much less pronounced with diol **38** compared to the first-generation diol **15** and the isomer 6*-epi*-**39** was observed only in trace amounts.

At this point, we investigated an alternative strategy to forge the second tetrahydrofuran ring, aiming to circumvent low-yielding acid-catalyzed acetalization. The acetal formation was envisioned via deprotection of epoxide **40**, featuring a pendant phenoxy residue, and concomitant cyclization. For the synthesis of **40**, we relied on our earlier intermediate ketone **36**. After a short evaluation of the olefination methods, we decided to use a Julia-Kocienski reaction to synthesize enol ether **41**. A comprehensive screen of reaction conditions (see Supporting Information for details) revealed that using methyl-substituted sulfonyltetrazole **42a** in combination with LHMDS was crucial to maximize the yield and (*E*)-selectivity. Under the optimized conditions (LHMDS, THF, −78 to −40 °C), the desired enol ether **41** was obtained in 45% yield together with the (*Z*)-isomer in 49% yield. Surprisingly, using the *tert*-butyl-substituted sulfonyltetrazole **42b** in the olefination reaction delivered enol ether **41** along with an epoxide side product in 49% yield. This unexpected product, identified as **43**, likely arises from a Corey−Chaykovsky-type reactivity. Hindered rotation around the C−C bond of the intermediate adduct may have prevented the Smiles rearrangement required for the alkene formation.

With desired enol ether **41** in hand, we proceeded with the epoxidation. Using *m*-CPBA resulted in the formation of epoxide **40** along with its minor diastereomer (dr = 2.5:1) resulting from the attack on the opposite face. Using dimethyldioxirane (DMDO) as the oxidant significantly improved diastereoselectivity, delivering desired epoxide **40** with complete stereocontrol in 79% yield. Contrary to our expectations, the epoxide was completely stable to both chromatographic purification on silica gel and long-term storage.^32^

Subjecting epoxide **40** to silyl deprotection using HF·pyr resulted in rapid formation of a mixture of diastereomeric fluorides **44** which converted into the two acetals **39** (43%) and 2-*epi*-**39** (36%) overnight. Alternative conditions, such as tetrabutylammonium fluoride (TBAF) or TBAF buffered with acetic acid, proved ineffective for this transformation. Silyl deprotection of an epoxide arising from oxidation of the (*Z*)-enol ether resulted in exclusive formation of the undesired acetal 2-*epi*-**39** in 62% yield. Following the epoxide cyclization strategy, alcohol **39** was obtained in 15% yield over three steps from ketone **36**, representing a modest improvement over the samarium(II) iodide-mediated cyclization that linked the intermediates in a 13% overall yield over four steps.

For the installation of the 1,3-benzodioxole motif in phrymarolin II precursor **29a**, a free phenol was required. Hydrogenolysis of **39** catalyzed by palladium on carbon delivered the corresponding phenol **45** in a 96% yield. The phenol was then oxidized using Frémy′s salt (**46**) and after a reductive workup with sodium dithionite catechol, **47** was obtained in 57% yield. Finally, the methylene bridge was installed by heating a solution of **47** in *N*,*N*-dimethylformamide (DMF) in the presence of diiodomethane and cesium carbonate to 100 °C to deliver alcohol **29a** in 22% yield.^33^ Accessing this previously obtained intermediate marked the completion of our second-generation approach to the phrymarolins.

### Model Studies of Bioinspired 1,4-Benzodioxane Formation

**Scheme 4:**
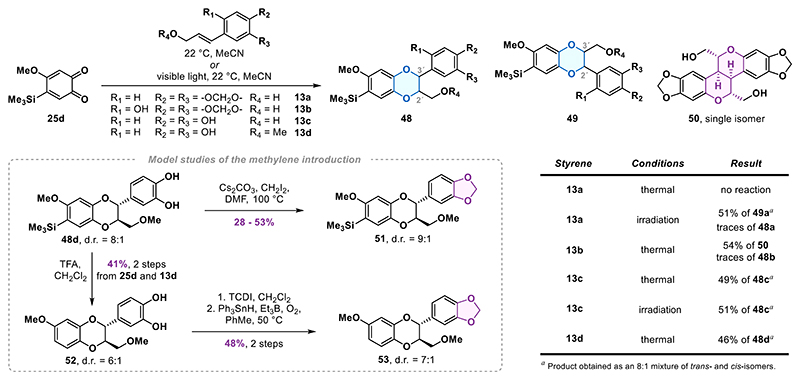
Model Studies of the Formal [4 + 2] Cycloaddition and Methylene Introduction

To find a suitable way to introduce the northeastern segment of haedoxan A (**1a**) via the bioinspired formal cycloaddition, we first investigated the transformation with a simplified model *ortho*-quinone (Scheme 4). From the quinones available from prior experiments, we chose trimethylsilyl-substituted quinone **25d** as our substrate of choice. This quinone offered an ideal balance of stability during long-term storage, ease of preparation and, as we found later, reactivity in the formal [4 + 2] cycloaddition.

We first attempted the reaction with styrene **13a**, already containing the benzodioxole ring present in **1a**. However, the formation of expected 1,4-benzodioxane product **48a** was not observed. Reactions similar to the envisioned formal [4 + 2] cycloaddition have also been reported under photochemical conditions.^34^ In the case of styrene **13a**, we observed the rapid formation of a 1,4-benzodioxane product under visible light irradiation (JBL Reptil LED lamp, 12W). However, 51% yield corresponded to undesired regioisomer **49a**. In a final attempt to directly incorporate the 1,3-benzodioxole ring in the formal cycloaddition reaction, we synthesized styrene **13b** possessing an additional hydroxy group, which we anticipated would exhibit greater reactivity toward *ortho*-quinone **25d** compared to styrene **13a**. Indeed, rapid conversion was observed with **13b**, but the desired 1,4-benzodioxane was not formed. Instead, we isolated hexacycle **50** in 54% yield which resulted from oxidative dimerization of styrene **13b** (see, Supporting Information for more details).^35^

Based on these findings, we shifted our focus from the initially envisioned introduction of the 1,3-benzodioxole motif directly in the formal cycloadditions step. Instead, we explored the formal [4 + 2] cycloaddition of **25d** with caffeyl alcohol (**13c**), which was reported to undergo similar transformations,^36^ with the prospect of installing the required methylene bridge in a subsequent step via transformation of the catechol motif.

Simply stirring a solution of styrene **13c** and quinone **25d** resulted in full conversion overnight. The 1,4-benzodioxane product **48c** was isolated in 49% yield as an 8:1 mixture of *trans-* and *cis-*isomers. A side product isolated in 10% from the reaction of **25d** and **13c** was identified as isomericanol A, a natural product resulting from dimerization of the styrene **13c**.^36b^ Under irradiation, *ortho*-quinone **25d** and caffeyl alcohol (**13c**) also gave the desired regioisomer **48c** in 51% yield as the sole product.^37^

To investigate the methylene installation, we chose to continue with 1,4-benzodioxane **48d**, which features a methoxy group instead of the free alcohol. This modification was expected to eliminate chemoselectivity issues and increase convergence, as haedoxan A (**1a**) also possesses the methoxy group. The 1,4-benzodioxane **48d** was obtained from the formal [4 + 2] cycloaddition of *ortho*-quinone **25d** and styrene **13d** in 46% yield as an 8:1 diastereomeric mixture. Standard methylenation conditions (CH_2_I_2_, Cs_2_CO_3_, DMF, 100 °C) delivered 1,3-benzodioxole **51** in yields ranging from 28 to 53% as a 9:1 mixture of isomers. When the reaction was carried out with pure *trans*-isomer **48c**, 1,3-benzodioxole **48a** was obtained as a 9:1 mixture of *trans*- and *cis*-isomers. We hypothesized that deprotonation of the catechol **48c** resulted in reversible C3′-O bond cleavage. This led to epimerization at C3′ while opening up pathways for side reactions of the intermediate *para*-quinone methide, which may explain the modest yield observed in catechol alkylation.

We also explored methylene installation under nonbasic conditions; however, most of our efforts were unsuccessful (see, the Supporting Information for details). A promising protocol for introducing the methylene bridge was identified through a two-step strategy involving thiocarbonate formation, followed by reduction under radical conditions. Due to the instability of the silyl group, we decided to remove it before proceeding with further investigation. Therefore, we converted **48d** into catechol **52** by the action of trifluoroacetic acid (TFA). Catechol **52** was then converted to the corresponding thiocarbonate, which was then transformed into the 1,3-benzodioxole **53** in 42% yield over two steps using triphenylstannane in combination with AIBN in toluene at 100 °C.^38^ The efficiency of this sequence improved to 48% over two steps when triethylborane-oxygen was used to initiate the radical reaction. Notably, the more commonly used tributylstannane resulted in a significantly lower overall yield of 17%.

### Synthesis of Haedoxan Natural Products

**Scheme 5:**
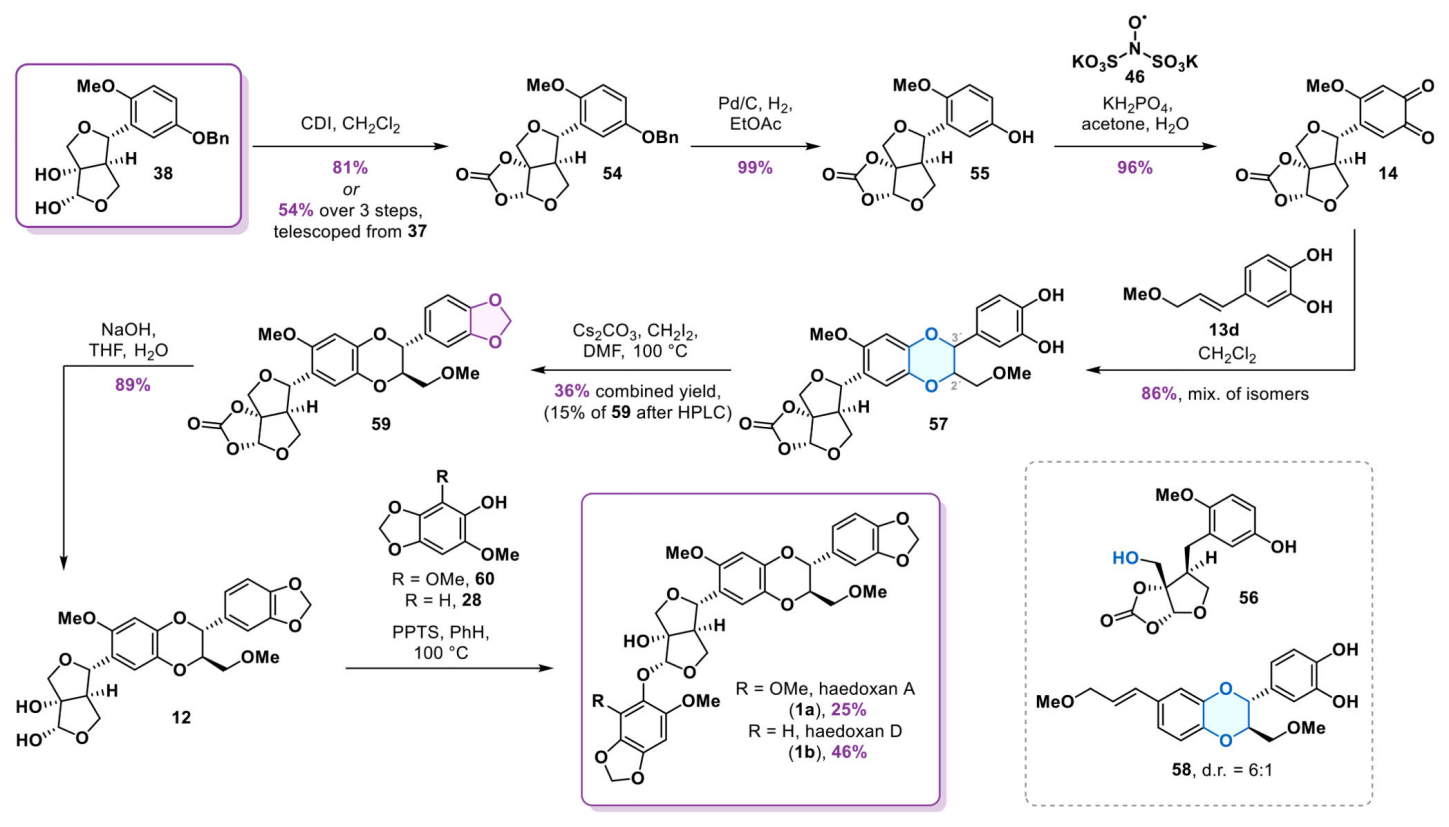
Total Synthesis of Haedoxans A and D

Based on our prior findings and aiming to generate unnatural haedoxan congeners via acetalic phenol modification, diol **38** was identified as the optimal branching point from the phrymarolin synthesis. We chose the route featuring the samarium(II) iodide-mediated cyclization since it offered the possibility to introduce structural diversity at the acetal carbon at a very late stage in the synthesis. Diol **38** was converted into cyclic carbonate **54** in 81% yield (Scheme 5). Telescoping the three-step sequence from alcohol **37** to carbonate **54** proved to be more efficient, delivering the product in 54% overall yield. The unmasking of the crucial *ortho*-quinone moiety commenced with debenzylation of carbonate **54** which allowed for isolation of phenol **55** in 99% yield. Using only 5% of the catalyst was crucial to enable careful monitoring of the hydrogenolysis and avoid benzylic reduction to alcohol **56**. The following oxidation to the requisite *ortho*-quinone **14** was achieved by exposing phenol **55** to IBX in DMF or alternatively using Frémy′s salt (**46**). Satisfyingly, the latter protocol enabled isolation of quinone **14** in an excellent 96% yield without the need for chromatographic purification, which was necessary in the case of IBX and inevitably led to a loss of product due to its sensitivity to silica.

Having already explored the bioinspired cyclization and the 1,3-benzodioxole synthesis on the model system, we moved on to performing the formal cycloaddition reaction with *ortho*-quinone **14**. Slow addition of catechol **13d** to a solution of the *ortho*-quinone resulted in conversion to the mixture of isomeric products **57** which were isolated in 86% combined yield. Additionally, we were able to isolate 10% of 1,4-benzodioxane **58** resulting from dimerization of styrene **13d**. Based on our previous work with 1,4-benzodioxane isomers, ^1^H NMR analysis indicated that the *trans*-isomers accounted for roughly 80% of the product mixture. Consistent with the results obtained from the model system, we observed excellent *trans*/*cis* selectivity and regioselectivity. However, with regard to the two possible *trans*-isomers, no diastereocontrol induced by the remote furofuran segment was detected as their ratio was found to be 1:1.

To install the requisite methylene bridge, we first employed the thiocarbonate reduction sequence; however, in this case, the yield of the 1,3-benzodioxole products never exceeded 20%. Since the promising conditions identified in our preliminary screening failed to meet our expectations, we decided to rely on the established method of *O*-alkylation using diiodomethane in the presence of cesium carbonate. Under these conditions, the complex mixture of isomers **57** was converted into a mixture of 1,3-benzodioxoles in 36% combined yield. Gratifyingly, the desired *trans*-isomer **59** could be cleanly isolated from the mixture by HPLC separation, achieving a 15% yield from catechol mixture **57**.

To obtain a sample of synthetic haedoxan A, the carbonate in **59** was removed using sodium hydroxide, affording diol **12** in 89% yield. Subsequent acid-catalyzed condensation with phenol **60** yielded haedoxan A (**1a**) in 25%, consistent with the yield previously reported by Taniguchi.^19^ Similarly, condensation with phenol **28** delivered haedoxan D (**1b**) in 46% yield. The analytical data for both synthetic compounds matched those of haedoxans A and D isolated from natural sources.^7,19^

### Modification of the *O*-Aryl Residue

**Scheme 6:**
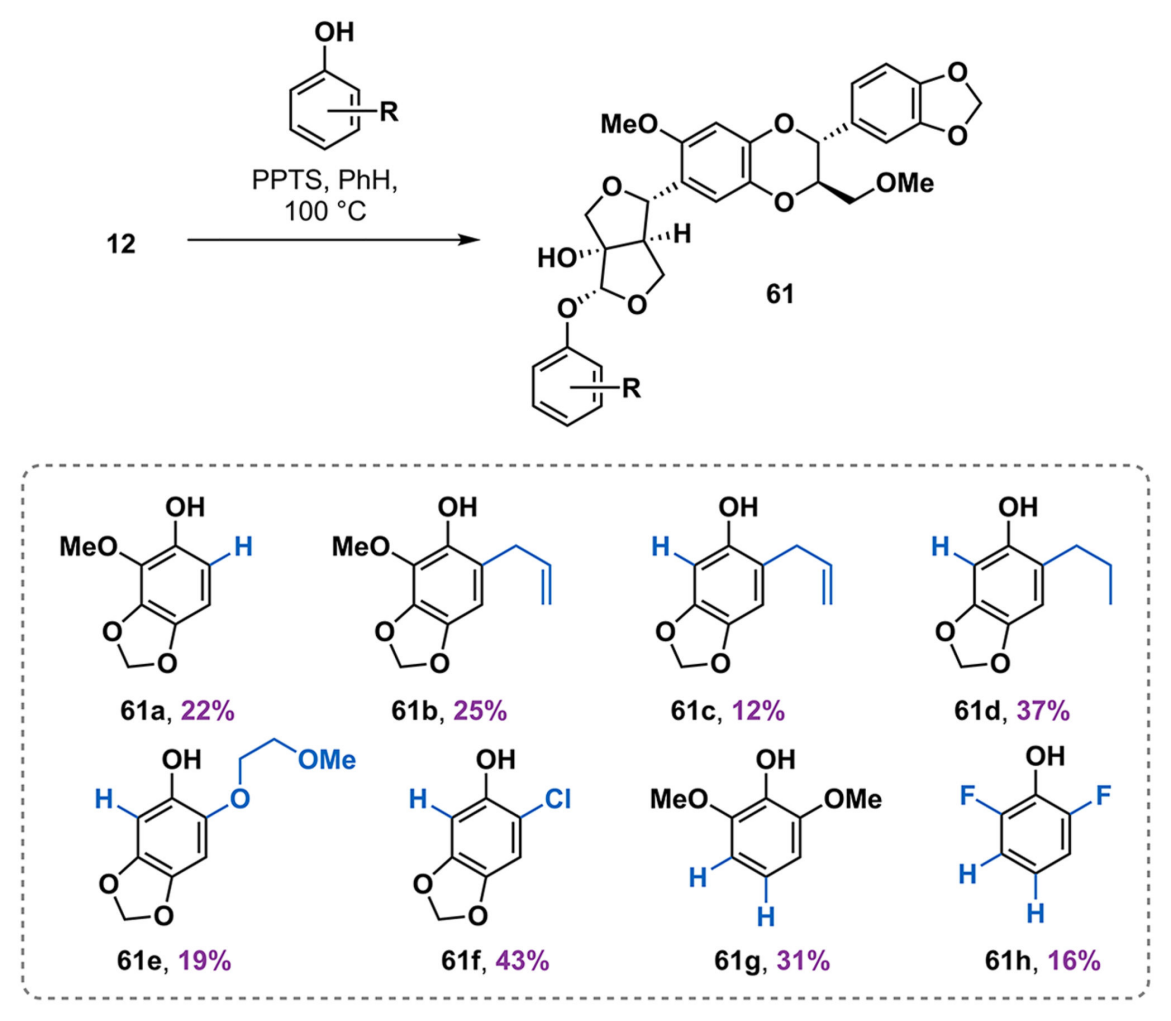
Haedoxan Analogues

Several synthetic analogues of **1a** have been synthesized and evaluated in previous structure−activity relationship (SAR) studies.^21^ The SAR studies primarily targeted modifications of the 1,4-benzodioxane fragment. To investigate the effect of the phenol substitution pattern on the insecticidal activity of the haedoxan scaffold, we synthesized a range of unnatural analogues of **1a** via the acid-catalyzed condensation with phenols. Similarly to the case of the phrymarolins and the hemedoxans, the analogues **61** were isolated in yields ranging from 12 to 43% (Scheme 6).

The efficacy of the synthetic racemic haedoxan A and D (**1a** and **1b**) and some of their analogues were tested in insecticidal bioassays (Table 1). While most tested analogues showed insecticidal activity, the synthetic racemate of haedoxan A (1a) was less potent in bioassays than the enantiopure natural (+)-haedoxan A. The markedly reduced activity of racemic haedoxan A (**1a**) remains unclear, but future studies will examine the biological activity of its individual enantiomers, which may interact differently with the molecular target. Haedoxan D (**1b**) exhibited 100% mortality against the cucumber beetle *Diabrotica balteata* but was inactive against the armyworm *Spodoptera frugiperda* and the greenfly *Myzus persicae*.

Based on a structural similarity between the methylenedioxy structural element in haedoxan A and known insecticidal synergists (e.g., sesamol),^39^ we aimed to explore the relevance of the methylenedioxy group in the western part of the molecule. Among the prepared derivatives **61a** to **61h**, we were surprised to find that compounds **61g** and **61h** showed a higher potency in the insecticidal tests than the racemic synthetic natural product **1a**. Other derivatives (**61b** to **61f**) were less potent or completely inactive at the tested concentration. These results indicate that the methylenedioxy group may not be a key structural element for the biological activity.^40^

## Conclusions

We have developed a synthetic strategy enabling access to furofuran lignan natural products phrymarolins I and II, as well as insecticidal haedoxans A and D. The first tetrahydrofuran ring was constructed via a formal [3 + 2] cycloaddition between *α*-silyloxy aldehyde **20** and styrene **18**. Subsequent cyclization of *β*-formyloxy ketone **16** triggered by samarium-(II) iodide led to construction of the furofuran core. This enabled the synthesis of phrymarolins **2a** and **2b** in eight steps from known aryl bromide **22**. Haedoxans were inaccessible at this stage, as the 1,3-benzodioxole handle in intermediate **30** could not be utilized for the unmasking of *ortho*-quinone **14** required for the bioinspired formal [4 + 2] cycloaddition. To overcome this, we designed styrene **32**. This enabled an increase in the efficiency of the formal [3 + 2] cycloaddition and facilitated the installation of the necessary *ortho*-quinone **14**. Applying the bioinspired cyclization to quinone **14** and styrene **13d** followed by methylenation afforded the full haedoxan scaffold. This strategy ultimately enabled the synthesis of haedoxan A (**1a**) and D (**1b**), along with their unnatural analogues, in 13 steps starting from aryl bromide **34**.

## Supplementary Material

The Supporting Information is available free of charge at https://pubs.acs.org/doi/10.1021/jacs.5c16676.

Experimental procedures, computational details, and characterization data for all compounds (PDF)

SI

## Figures and Tables

**Table 1:** Biological Testing of Haedoxans and Analogues

compound	*Spodoptera* *frugiperda* ^a^	*Diabrotica* *balteata* ^b^	*Myzus* *persicae* ^c^
natural (+)-**1a**	100	100	100
synthetic **(±)-1a**	33	50	0
synthetic (±)-**1b**	0	100	0
(±)-**61a**	100	80	0
(±)-**61b**	50	0	0
(±)-**61c**	0	0	0
(±)-**61d**	50	100	0
(±)-**61e**	17	60	0
(±)-**61f**	17	100	0
(±)-**61g**	67	100	100
(±)-**61h**	100	100	0

a Seed survival percentage at 20 g/ha.

b Larval mortality percentage at 20 g/ha in a treated leaf disk test.

c % Insect mortality percentage at 20 ppm in oral application.
