# Supplementary material for: Unified Total Synthesis of Phrymarolin and Haedoxan Natural Products
Source: J Am Chem Soc. 2025 Dec 4;147(50):46461–70. doi: 10.1021/jacs.5c16676 (PMC7618468; doi:10.1021/jacs.5c16676)

Supporting Information

Unified Total Synthesis of Phrymarolin and Haedoxan Natural Products

Jan Paciorek†, Antoine D. F. Guy†, Alexander Sudau⊥, David M. Barber§, Thomas Magauer*,†

†Department of Organic Chemistry and Center for Molecular Biosciences, University of Innsbruck, Innrain 80–82, 6020 Innsbruck, Austria

⊥Research and Development, Profile Driven Chemistry, Bayer AG, Crop Science Division, Alfred Nobel Str. 50, 40789 Monheim am Rhein, Germany

§Research and Development, Profile Driven Chemistry, Bayer AG, Crop Science Division, Industriepark Höchst, 65926 Frankfurt am Main, Germany

Corresponding author: Thomas.Magauer@uibk.ac.at

Table of Contents

General Information S1

Experimental Procedures S4

Initial studies and total synthesis of phrymarolins: S4

Improved synthesis of the furofuran core: S17

Synthesis of styrenes: S32

Model studies of the formal [4+2] cycloaddition reaction: S42

Formal [4+2] cycloaddition under photochemical conditions: S53

Methylenation investigation: S61

Synthesis of phenols: S68

Final stage of the total synthesis of haedoxans: S77

Synthesis of haedoxan analogues: S85

Epoxidation-cyclization strategy towards phrymarolins: S93

Comparison of Synthetic Compounds with Literature S106

References S113

NMR Spectra S115

General Information

Unless otherwise noted, all reactions were magnetically stirred and carried out in oven-dried glassware fitted with rubber septa, under an inert atmosphere of argon. Air– and moisture–sensitive liquids were transferred via syringes through the rubber septa. Solids were added either using standard Schlenk techniques or were dissolved in appropriate solvents. Unless otherwise noted, the reaction temperatures are assumed to be 22 °C. Reactions performed at low temperatures were cooled by immersing reaction flasks in a mixture of acetone/dry ice (–78 °C), water/ice (0 °C) or in an acetone bath cooled to the desired temperature by a Julabo FT902 cryostat. Reactions at temperatures above 22 °C were performed in flasks placed in an oil bath or an aluminum metal block heated to the appropriate temperature. The reactions were monitored by NMR spectroscopy or thin-layer chromatography (TLC) using aluminum sheets precoated with silica gel (Merck, 0.25 mm, 60 Å pore, impregnated with fluorescent indicator). The TLC plates were visualized by exposure to UV light (254 nm) or by immersing into an aqueous potassium permanganate staining solution followed by heating with a heat gun. Flash chromatography was performed using Merck silica gel 60 (0.040–0.063 mm). Automated flash column chromatography was performed using a puriFlash XS520+ (Advion Interchim Scientific) with pre-packed cartridges (Merck silica gel 60, 0.040–0.063 mm).

Reagents were obtained from commercial sources (Sigma Aldrich, TCI, BLDpharm, Fisher etc.) and were used as such without further purification unless otherwise noted. Reaction grade solvents were obtained from commercial sources (Acros Organics, Thermo Fisher, Sigma Aldrich) as extra dry and were transferred to reaction mixtures under argon via syringe. Tetrahydrofuran and diethyl ether were additionally dried over 4Å molecular sieves. Solvents for flash chromatography were obtained as crude and were distilled under reduced pressure prior to use.

High performance liquid chromatography (HPLC) separations were conducted on a normal-phase Varian Dynamax column (250 × 41.4 mm Microsorb 60-8 Si column) for preparative scale and on a normal-phase Shimadzu Shim-pack PRC-SIL(H) column (250 × 20 mm, 5 µm particle diameter) for semipreparative scale.

NMR spectra (^{1}H NMR, ^{13}C NMR, ^{19}F NMR) were recorded at 25 °C in deuterated chloroform ($CDCl_3$), deuterated benzene (C_6D_6), deuterated methanol (CD_3OD), deuterated acetonitrile (CD_3CN), deuterated acetone (acetone-d_6) or dimethylsulfoxide (DMSO-d_6) on a Bruker Avance Neo 400MHz or a Bruker Avance Neo 500 MHz spectrometer and are reported as follows: chemical shift δ in ppm (multiplicity, coupling constant J in Hz, number of protons) for ^{1}H NMR spectra and chemical shift δ in ppm for ^{13}C NMR and ^{19}F spectra. Multiplicities are abbreviated as follows: s = singlet, d = doublet, t = triplet, q = quartet, m = multiplet, br = broad, or combinations thereof. Residual solvent peaks of $CDCl_3$ (δ_H = 7.26 ppm, δ_C = 77.16 ppm), C_6D_6 (δ_H = 7.16 ppm, δ_C = 128.06 ppm), CD_3OD (δ_H = 3.31 ppm, δ_C = 49.00 ppm), CD_3CN (δ_H = 1.94 ppm, δ_C = 1.32 ppm), acetone-d_6 (δ_H = 2.05 ppm, δ_C = 29.84 ppm) and DMSO-d_6 (δ_H = 2.50 ppm, δ_C = 39.52 ppm) were used as internal reference. The ^{19}F NMR spectra are reported without a reference peak. NMR spectra were assigned using information ascertained from COSY, HMBC, HSQC and NOESY experiments.

Infrared spectra were recorded from 4000 cm^{-1} to 450 cm^{-1} on a Bruker™ ALPHA FT-IR Spectrometer. Samples were prepared as a neat film by evaporation of a solution in $CDCl_3$. IR data in wavenumber $\tilde{\nu}$ (cm^{-1}) are reported as follows: *w* = weak, *m* = medium, *s* = strong, *br* = broad or combinations thereof.

High-resolution mass spectra were recorded on a Thermo Scientific™ LTQ Orbitrap XL™ Hybrid Ion Trap-Orbitrap Mass Spectrometer at the Institute of Organic Chemistry and Center for Molecular Biosciences, University of Innsbruck.

Melting points were measured with an SRS MPA120 EZ-Melt Melting Point Apparatus in open glass capillaries and are uncorrected.

All yields were calculated from amounts of isolated pure products.

Biological activity testing

Diabrotica balteata - spray test

Solvent: 78.0 parts by weight of acetone / 1.5 parts by weight of dimethylformamide

Emulsifier: 0.5 parts by weight alkylarylpolyglycol etherTo produce a suitable preparation of active compound, 1 part by weight of active compound was mixed with the stated amount of solvent and emulsifier, and the concentrate was diluted with water to the desired concentration of 1000 ppm. Further test concentrations were prepared by dilution with emulsifier containing water. Soaked wheat seeds *(Triticum aestivum)* were placed in a multiple well plate filled with agar and some water and were incubated for 1 day to germinate (5 seeds per well). The germinated wheat seeds were sprayed with a test solution containing the desired concentration of the active ingredient. Afterwards, each unit was infected with 10-20 larvae of the banded cucumber beetle *(Diabrotica balteata).* After 7 days efficacy in % was determined as follows:

5 seedlings had been tested:

- 0 out of 5 seeds germinated and developed normally: 0% Efficacy
- 1 out of 5: 20%
- 2 out of 5: 40%
- 3 out of 5: 60%
- 4 out of 5: 80%
- 5 out of 5: 100%

Spodoptera frugiperda - spray test

Solvent: 78.0 parts by weight acetone / 1.5 parts by weight dimethylformamide

Emulsifier: 0.5 parts by weight alkylarylpolyglycol ether

To produce a suitable preparation of active compound, 1 part by weight of active compound was mixed with the stated amount of solvents and emulsifier and was diluted with water to the desired concentration of 1000 ppm. Further test concentrations were prepared by dilution with emulsifier containing water. Corn *(Zea mays)* leaf sections were sprayed with a preparation of the active ingredient of the desired concentration. Once dry, the leaf sections were infested with fall armyworm larvae *(Spodoptera frugiperda).* After 7 days mortality in % was determined as follows:

6 Larvae had been tested:

- 0 out of 6 alive: 100% Efficacy
- 1 out of 6 alive: 83%
- 2 out of 6: 67%
- 3 out of 6: 50%
- 4 out of 6: 33%
- 5 out of 6: 17%
- 6 out of 6: 0%

Myzus persicae - oral test

Solvent: 100 parts by weight acetone

To produce a suitable preparation of active compound, 1 part by weight of active compound was mixed with the stated amount of solvent, and the concentrate was diluted with water to the desired concentration. A 50 µL of the compound solution was filled in microtiter plates and 150 µL 33% (v/v) IPL41 insect medium (#I7760 Sigma-Aldrich + 15 % (w/v) sucrose) was added to obtain a total volume of 200 µL per well. Afterwards the plates were sealed with Parafilm through which a mixed population of the green peach aphid *(Myzus persicae)* could feed on the compound preparation. Mortality (%) was measured after 8 days: 100% means all aphids were dead, 0% means none were dead.

Thirty to 40 Aphids had been tested twice:

- 0 out of >30 alive / all dead: 100% Efficacy
- 1 alive: 95%
- 2 aphids alive: 90%; 3:85%; 4: 80%
- 6 aphids: 70%; 8: 60%; 10: 50%;
- 12 aphids: 40%;
- 14 aphids: 30%;
- 16 aphids :20%
- 18 aphids: 10%
- ≥20 aphids: 0%

Experimental Procedures

Initial studies and total synthesis of phrymarolins:

Synthesis of arene 26b

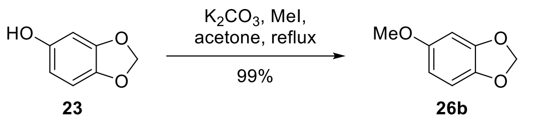

A suspension of sesamol (**23**) (15.0 g, 109 mmol, 1 equiv), potassium carbonate (45.0 g, 326 mmol, 3.00 equiv) and methyl iodide (40.7 mL, 651 mmol, 6.00 equiv) in acetone (200 mL) was vigorously stirred and refluxed for 16 h. The mixture was then allowed to cool to 22 °C and the mixture was concentrated under reduced pressure. An aqueous solution of hydrogen chloride (2 M, 200 mL) was added and the mixture was extracted with diethyl ether (200 mL). The organic solution was washed with a saturated aqueous solution of sodium chloride (100 mL). The washed solution was dried over sodium sulfate and filtered. The filtrate was concentrated under reduced pressure which afforded arene **26b** as a pale-yellow liquid (16.5 g, 108 mmol, 99%) that was used without further purification.

^{1}H NMR (400 MHz, $CDCl_3$): δ 6.71 (d, J = 8.4 Hz, 1H), 6.49 (d, J = 2.5 Hz, 1H), 6.32 (dd, J = 8.5, 2.5 Hz, 1H), 5.91 (s, 2H), 3.75 (s, 3H) ppm.

The analytical data matched those reported previously.[1]

Synthesis of aryl bromide 22

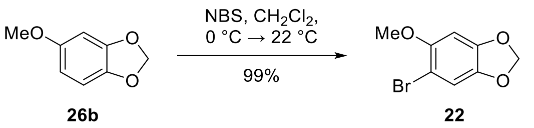

To a solution of arene **26b** (2.00 g, 13.1 mmol, 1 equiv) in dichloromethane (30 mL) cooled to 0 °C was added *N*-bromosuccinimide (2.46 g, 13.8 mmol, 1.05 equiv) portionwise. After the addition was completed, the solution was allowed to warm to 22 °C and stirred for 30 min. The solution was washed with water (3 × 30 mL) and a saturated aqueous sodium chloride solution (30 mL). The washed solution was dried over sodium sulfate and filtered. The filtrate was concentrated under reduced pressure which afforded aryl bromide **22** as a white solid (3.00 g, 13.0 mmol, 99%) that was used without further purification.

^{1}H NMR (400 MHz, $CDCl_3$): δ 7.00 (s, 1H), 6.57 (s, 1H), 5.94 (s, 2H), 3.83 (s, 3H) ppm.

The analytical data matched those reported previously.[2]

Synthesis of silyl ether SI-2

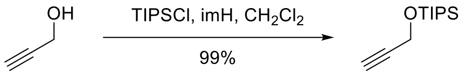

To a solution of propargyl alcohol (**SI-1**) (1.00 g, 17.8 mmol, 1 equiv) in dichloromethane (30 mL) was added imidazole (1.52 g, 22.3 mmol, 1.25 equiv) followed by triisopropylsilyl chloride (4.58 mL, 21.4 mmol, 1.20 equiv). After stirring for 3 h, the mixture was washed with water (30 mL) and a saturated aqueous sodium chloride solution (30 mL). The washed solution was dried over sodium sulfate and filtered. The filtrate was concentrated under reduced pressure and the residue was purified by flash chromatography on silica gel (5% diethyl ether in petroleum ether) which afforded silyl ether **SI-2** as a colorless liquid (3.78 g, 17.8 mmol, 99%).

^{1}H NMR (400 MHz, $CDCl_3$): δ 4.38 (d, J = 2.4 Hz, 2H), 2.39 (t, J = 2.4 Hz, 1H), 1.18 – 1.04 (m, 21H) ppm.

The analytical data matched those reported previously.[3]

Synthesis of boronic ester 21

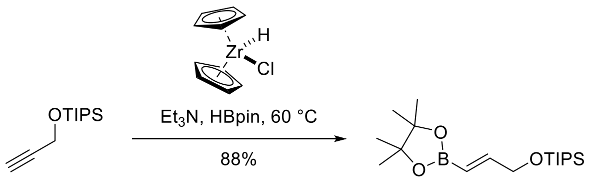

A mixture of silyl ether **SI-2** (5.53 g, 26.0 mmol, 1 equiv), triethylamine (726 µL, 5.21 mmol, 0.200 equiv), Schwartz´s reagent (672 mg, 2.60 mmol, 0.100 equiv) and pinacolborane (5.66 mL, 39.1 mmol, 1.50 equiv) was stirred at 60 °C for 20 h. The mixture was allowed to cool to 22 °C and methanol (1.84 mL, 45.6 mmol, 1.75 equiv) was added. Flash chromatography of the mixture on silica gel (5% diethyl ether in *n*-pentane grading to 10% diethyl ether in *n*-pentane) afforded boronic ester **21** as a colorless oil (7.80 g, 22.9 mmol, 88%).

TLC (5% ethyl acetate in cyclohexane): R_f = 0.55 (weak UV, $KMnO_4$).

^{1}H NMR (400 MHz, $CDCl_3$): δ 6.68 (dt, J = 18.0, 3.4 Hz, 1H), 5.81 (dt, J = 17.9, 2.2 Hz, 1H), 4.33 (dd, J = 3.4, 2.2 Hz, 2H), 1.27 (s, 12H), 1.13 – 1.03 (m, 21H) ppm.

^{13}C NMR (101 MHz, $CDCl_3$): δ 152.4, 83.3, 64.8, 24.9, 18.2, 12.2 ppm. Signal of the boron-bound carbon was not observed.

IR (ATR, neat): $\tilde{\nu}$ = 2943 (*m*), 2892 (*m*), 2866 (*m*), 1742 (*w*), 1645 (*m*), 1464 (*m*), 1370 (*m*), 1339 (*s*), 1319 (*s*), 1274 (*m*), 1214 (*m*), 1144 (*s*), 1058 (*w*), 973 (*m*), 947 (*w*), 882 (*m*), 852 (*m*), 804 (*w*), 682 (*m*) cm^{-1}.

HRMS (ESI): calcd. for $C_{18}H_{38}BO_3Si$ [M+H]$^+$: 341.2678; found: 341.2681.

Synthesis of styrene 18

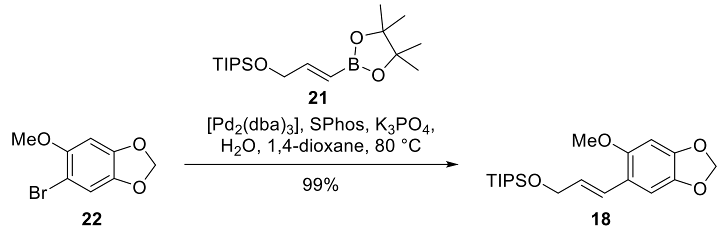

A mixture of bromide **22** (1.25 g, 5.41 mmol, 1 equiv), tripotassium phosphate (4.59 g, 21.6 mmol, 4.00 equiv), boronic ester **21** (2.30 mg, 6.76 mmol, 1.25 equiv), SPhos (222 mg, 541 µmol, 0.100 equiv) and $Pd_2(dba)_3$ (124 mg, 135 µmol, 2.5 mol%) in 1,4-dioxane (15 mL) and water (3 mL) was vigorously stirred and heated to 80 °C in a sealed tube. After 20 h, the mixture was allowed to cool to 22 °C. Water (20 mL) was added and the mixture was extracted with ethyl acetate (2 × 25 mL). The combined organic layers were washed with a saturated aqueous sodium chloride solution. The washed organic solution was dried over sodium sulfate and filtered. The filtrate was concentrated under reduced pressure. The residue was purified via flash column chromatography on silica gel (3% diethyl ether in petroleum ether grading to 5% diethyl ether in petroleum ether) which afforded styrene **18** as a colorless oil (1.95 g, 5.35 mmol, 99%).

TLC (5% ethyl acetate in cyclohexane): R_f = 0.44 (UV, $KMnO_4$).

^{1}H NMR (400 MHz, $CDCl_3$): δ 6.94 (s, 1H), 6.89 (dt, *J* = 16.0, 1.8 Hz, 1H), 6.50 (s, 1H), 6.11 (dt, *J* = 16.0, 5.2 Hz, 1H), 5.91 (s, 2H), 4.41 (dd, *J* = 5.2, 1.8 Hz, 2H), 3.77 (s, 3H), 1.15 – 1.06 (m, 21H) ppm.

^{13}C NMR (101 MHz, $CDCl_3$): δ 152.4, 147.6, 141.7, 127.9, 124.0, 119.3, 105.8, 101.3, 95.4, 64.6, 57.0, 18.19, 12.3 ppm.

IR (ATR, neat): $\tilde{\nu}$ = 2941 (*m*), 2891 (*m*), 2866 (*m*), 1505 (*m*), 1482 (*s*), 1464 (*m*), 1427 (*m*), 1381 (*w*), 1351 (*w*), 1283 (*m*), 1192 (*s*), 1154 (*m*), 1111 (*m*), 1041 (*s*), 1012 (*m*), 968 (*m*), 938 (*m*), 882 (*m*), 820 (*m*), 770 (*m*), 759 (*m*), 681 (*m*) cm^{-1}.

HRMS (ESI): calcd. for $C_{20}H_{33}O_4Si$ [M+H]$^+$: 365.2143; found: 365.2144.

Synthesis of silyl ether SI-3

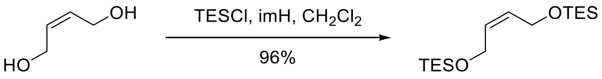

SI-3

To a heterogeneous mixture of *cis*-but-2-ene-1,4-diol (5.00 mL, 56.7 mmol, 1 equiv) in dichloromethane (100 mL) was added imidazole (8.50 g, 125 mmol, 2.20 equiv) followed by triethylsilyl chloride (20.0 mL, 119 mmol, 2.10 equiv). After stirring for 3 h, the mixture was washed with water (100 mL) and with a saturated aqueous sodium chloride solution (100 mL). The washed solution was dried over sodium sulfate and filtered. The filtrate was concentrated under reduced pressure and the residue was purified by flash chromatography on silica gel (5% diethyl ether in petroleum ether) which afforded silyl ether **SI-3** as a colorless liquid (17.3 g, 54.6 mmol, 96%).

^{1}H NMR (400 MHz, $CDCl_3$): δ 5.63 – 5.53 (m, 2H), 4.28 – 4.19 (m, 4H), 0.96 (t, *J* = 7.9 Hz, 18H), 0.61 (q, *J* = 8.0 Hz, 12H) ppm.

The analytical data matched those reported previously.[4]

Synthesis of aldehyde 20

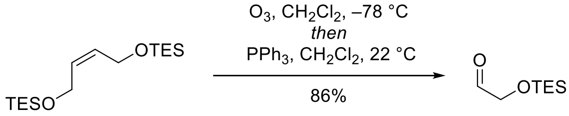

SI-3 **20**

A solution of silyl ether **SI-3** (3.78 g, 11.9 mmol, 1 equiv) in dichloromethane (120 mL) cooled to –78 °C was ozonolyzed until the blue color of ozone persisted. The solution was then sparged with oxygen until a colorless solution was obtained. Triphenylphosphine (3.44 g, 13.1 mmol, 1.10 equiv) was added at –78 °C and the mixture was allowed to warm to 22 °C. After 2 h, the mixture was concentrated until there was around 20 mL of liquid left. *n*-Pentane (200 mL) was added and the resulting suspension was filtered. The filtrate was concentrated under reduced pressure and the residue was purified by flash chromatography on silica gel (10% diethyl ether in *n*-pentane) which afforded aldehyde **20** as a colorless oil (3.56 g, 10.2 mmol, 86%).

^{1}H NMR (400 MHz, $CDCl_3$): δ 9.71 (t, *J* = 0.9 Hz, 1H), 4.20 (d, *J* = 1.0 Hz, 2H), 0.98 (t, *J* = 7.9 Hz, 9H), 0.65 (q, *J* = 7.9 Hz, 6H) ppm.

The analytical data matched those reported previously.[5]

Synthesis of alcohols 17

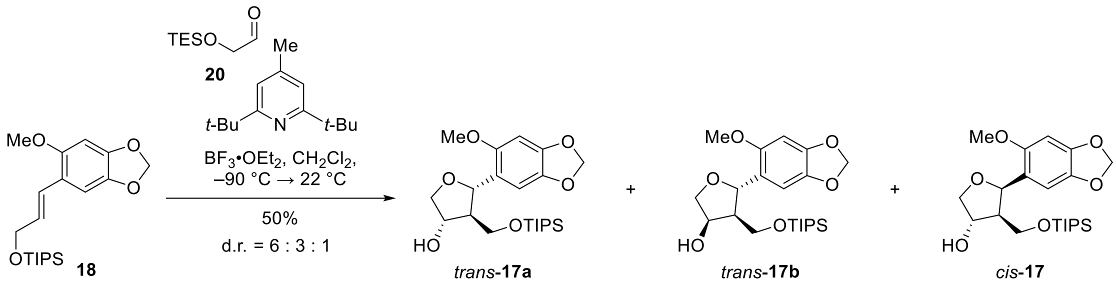

To a solution of styrene **18** (5.40 g, 14.8 mmol, 1 equiv), aldehyde **20** (4.00 g, 22.9 mmol, 1.55 equiv) and 2,6-di-*tert*-butyl-4-methylpyridine (4.56 g, 22.2 mmol, 1.50 equiv) in dichloromethane (30 mL) cooled to –90 °C was added dropwise BF_3•OEt_2 (2.25 mL, 17.8 mmol, 1.20 equiv). After 5 min, the solution was allowed to warm to 22 °C. After 30 min, the solution was poured into a flask containing a saturated aqueous sodium bicarbonate solution (50 mL) and the resulting mixture was stirred for 30 min. The organic layer was separated and the aqueous layer was extracted with dichloromethane (50 mL). The organic solutions were combined and washed with a saturated aqueous solution of sodium chloride (50 mL). The washed organic solution was dried over sodium sulfate and filtered. The filtrate was concentrated under reduced pressure and the residue was purified by flash chromatography on silica gel (15% ethyl acetate in petroleum ether grading to 20% ethyl acetate in petroleum ether) which afforded a diastereomeric mixture of alcohols **17** as a pale-yellow viscous oil (3.17 g, 7.47 mmol, 50%).

TLC (20% ethyl acetate in cyclohexane): R_f = 0.29 (UV, $KMnO_4$).

Note: The NMR and IR data are not reported due to the complexity of the spectra caused by presence of three inseparable isomers.

HRMS (ESI): calcd. for $C_{22}H_{36}NaO_6Si$ $[M+Na]^+$: 447.2173; found: 447.2165.

Synthesis of ketone 24

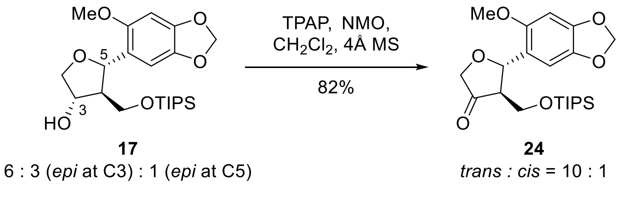

To a solution of the mixture of alcohols **17** (2.94 g, 6.92 mmol, 1 equiv) in dichloromethane (50 mL) were added 4Å molecular sieves (10 g), *N*-methylmorpholine *N*-oxide (1.22 g, 10.4 mmol, 1.50 equiv) and tetrapropylammonium perruthenate (122 mg, 346 µmol, 5 mol%) and the resulting mixture was stirred for 1 h. The mixture was filtered through celite and concentrated under reduced pressure. The residue was

purified by flash chromatography on silica gel (10% ethyl acetate in petroleum ether) which afforded ketone **24** as a 10:1 mixture of *trans*- and *cis*-isomers respectively (colorless oil, 2.40 g, 5.68 mmol, 82%).

TLC (20% ethyl acetate in cyclohexane): R_f = 0.55 (UV, $KMnO_4$).

Note: Only the NMR signals of the major *trans*-isomer are reported.

^{1}H NMR (400 MHz, $CDCl_3$): δ 6.92 (s, 1H), 6.54 (s, 1H), 5.93 – 5.91 (m, 2H), 5.67 (d, *J* = 8.4 Hz, 1H), 4.25 (dd, *J* = 16.6, 1.2 Hz, 1H), 4.17 (dd, *J* = 9.7, 3.4 Hz, 1H), 3.94 (d, *J* = 16.5 Hz, 1H), 3.86 (dd, *J* = 9.7, 2.8 Hz, 1H), 3.75 (s, 3H), 2.49 – 2.42 (m, 1H), 1.09 – 1.01 (m, 21H) ppm.

^{13}C NMR (101 MHz, $CDCl_3$): δ 215.7, 152.8, 148.2, 141.5, 121.1, 107.5, 101.5, 94.8, 77.0, 72.6, 60.3, 56.34, 56.31, 18.03, 18.01, 12.0 ppm.

IR (ATR, neat): $\tilde{\nu}$ = 2942 (*m*), 2865 (*m*), 1760 (*m*), 1624 (*w*), 1505 (*m*), 1486 (*m*), 1463 (*m*), 1447 (*m*), 1271(*w*), 1253 (*w*), 1191 (*s*), 1162 (*m*), 1113 (*s*), 1068 (*s*), 1038 (*s*), 1009 (*m*), 972 (*m*), 936 (*m*), 881 (*s*), 819 (*w*), 785 (*w*), 759 (*m*), 680 (*s*) cm^{-1}.

HRMS (ESI): calcd. for $C_{22}H_{35}O_6Si$ $[M+H]^+$: 423.2197; found: 423.2192.

Synthesis of ketone 24 and *ortho*-quinone 25a

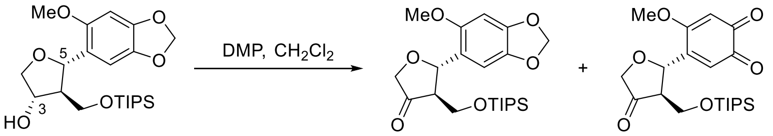

17
6 : 3 (*epi* at C3) : 1 (*epi* at C5)

24, 48%
trans : *cis* = 10 : 1

25a, 20%

To a solution of mixture of alcohols **17** (42 mg, 99 µmol, 1 equiv) in dichloromethane (0.5 mL) was added Dess–Martin periodinane (84 mg, 0.20 mmol, 2.0 equiv) and the resulting mixture was stirred for 2 h. The mixture was concentrated under reduced pressure and the residue was purified by flash chromatography on silica gel (10% ethyl acetate in petroleum ether grading to 30% ethyl acetate in petroleum ether) which afforded ketone **24** as a 10:1 mixture of *trans*- and *cis*-isomers respectively (colorless oil, 20 mg, 47 µmol, 48%) and quinone **25a** as a single *trans*-isomer (red solid, 8.0 mg, 20 µmol, 20%).

Analytical data for ketone **24** matched those obtained before (see above).

Characterization data for quinone **25a**:

TLC (30% ethyl acetate in cyclohexane): R_f = 0.34 (UV, $KMnO_4$).

^{1}H NMR (400 MHz, $CDCl_3$): δ 6.62 (d, J = 1.5 Hz, 1H), 5.83 (s, 1H), 5.46 (dd, J = 6.5, 1.5 Hz, 1H), 4.26 – 4.17 (m, 2H), 4.04 (d, J = 17.0 Hz, 1H), 3.98 (dd, J = 9.6, 2.8 Hz, 1H), 3.87 (s, 3H), 2.46 – 2.41 (m, 1H), 1.16 – 1.02 (m, 21H) ppm.

^{13}C NMR (101 MHz, $CDCl_3$): δ 213.3, 180.1, 178.4, 167.6, 151.8, 126.4, 103.7, 77.4, 71.8, 61.8, 57.1, 55.5, 18.0, 18.0, 12.0 ppm.

IR (ATR, neat): $\tilde{\nu}$ = 2945 (*m*), 2867 (*m*), 1762 (*s*), 1676 (*m*), 1649 (*m*), 1581 (*s*), 1463 (*w*), 1436 (*w*), 1384 (*s*), 1344 (*w*), 1264 (*m*), 1241 (*m*), 1196 (*m*), 1162 (*m*), 1114 (*s*), 1068 (*m*), 1014 (*w*), 983 (*m*), 947 (*w*), 883 (*m*), 839 (*m*), 767 (*w*), 689 (*m*) cm^{-1}.

HRMS (ESI): calcd. for $C_{21}H_{32}KO_6Si$ $[M+K]^+$: 447.1600; found: 447.1584.

Synthesis of alcohol 27

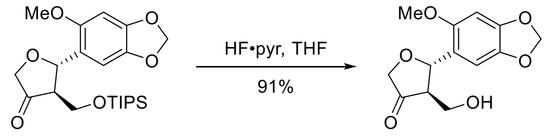

24 *trans : cis* = 10 : 1 → **27** *trans : cis* = 12 : 1

To a solution of ketone **24** (1.80 g, 4.26 mmol, 1 equiv) in tetrahydrofuran (20 mL) was added hydrogen fluoride-pyridine (3.32 mL, 70 wt% hydrogen fluoride, 128 mmol, 30.0 equiv) and the resulting solution was stirred at 40 °C. After 20 h, the reaction mixture was slowly poured into an aqueous solution prepared from saturated aqueous solution of sodium bicarbonate (50 mL) and aqueous solution of sodium hydroxide (2 M, 50 mL) cooled to 0 °C. The resulting mixture was stirred for 10 min at 0 °C. The mixture was extracted with ethyl acetate (2 × 50 mL). The combined organic solution was washed with a saturated aqueous sodium chloride solution (50 mL). The washed solution was dried over sodium sulfate and filtered. The filtrate was concentrated under reduced pressure and the residue was purified by flash chromatography on silica gel (40% ethyl acetate in petroleum ether) which afforded alcohol **27** as a 12:1 mixture of isomers (pale-yellow oil, 1.03 g, 3.87 mmol, 91%).

TLC (50% ethyl acetate in cyclohexane): R_f = 0.38 (UV, $KMnO_4$).

Note: Only the NMR signals of the major *trans*-isomer are reported.

^{1}H NMR (400 MHz, $CDCl_3$): δ 7.00 (s, 1H), 6.55 (s, 1H), 5.96 – 5.91 (m, 2H), 5.37 (d, J = 9.7 Hz, 1H), 4.33 (dd, J = 17.2, 0.8 Hz, 1H), 4.03 – 3.89 (m, 2H), 3.85 – 3.77 (m, 4H), 2.55 – 2.32 (m, 2H) ppm.

^{13}C NMR (101 MHz, $CDCl_3$): δ 215.9, 151.8, 148.3, 142.1, 120.4, 106.6, 101.6, 94.9, 76.6, 72.1, 59.1, 57.2, 56.8 ppm.

IR (ATR, neat): $\tilde{\nu}$ = 3458 (*br*, *w*), 2922 (*w*), 1755 (*m*), 1627 (*m*), 1504 (*m*), 1486 (*s*), 1466 (*m*), 1428 (*m*), 1274 (*w*), 1193 (*s*), 1164 (*m*), 1037 (*s*), 1006 (*w*), 932 (*w*), 862 (*w*) cm^{-1}.

HRMS (ESI): calcd. for $C_{13}H_{14}NaO_6$ $[M+Na]^+$: 289.0683; found: 289.0689.

Two-step synthesis of diol 15

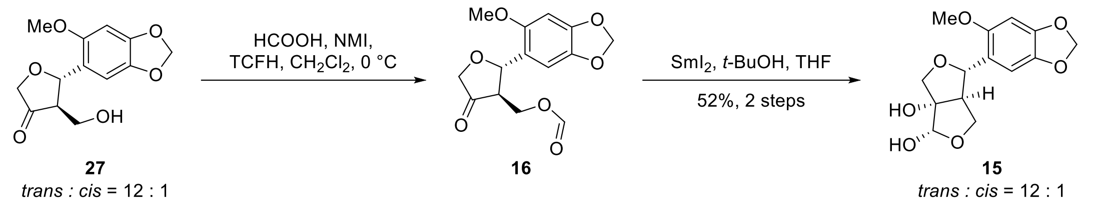

To a solution of alcohol **27** (400 mg, 1.50 mmol, 1 equiv) in dichloromethane (10 mL) was added *N*-methylimidazole (593 µL, 7.51 mmol, 5.00 equiv) and formic acid (283 µL, 7.51 mmol, 5.00 equiv). The resulting solution was cooled to 0 °C and *N,N,N',N'*-tetramethylchloroformamidinium hexafluorophosphate (1.23 g, 4.51 mmol, 3.00 equiv) was added portionwise over 5 min. After stirring at 0 °C for 5 min, the solution was added to a saturated aqueous ammonium chloride solution (20 mL). The mixture was extracted with dichloromethane (20 mL). The organic solution was washed with water (20 mL) and with an aqueous saturated sodium chloride solution (20 mL). The washed solution was dried over sodium sulfate and filtered. The filtrate was concentrated under reduced pressure and the residue containing formate ester **16** and tetramethylurea was directly used in the next step without further purification. To a solution of samarium diiodide (0.100 M in tetrahydrofuran, 60.1 mL, 6.01 mmol, 4.00 equiv) was added *tert*-butanol (1.44 mL, 15.0 mmol, 10.0 equiv) followed by a solution of the crude ester **16** (assumed 1.50 mmol, 1 equiv) in tetrahydrofuran (1 mL) and the resulting solution was stirred for 1 h. The solution was then poured into an aqueous solution prepared from 50 mL of saturated ammonium chloride solution and 100 mL of saturated aqueous sodium potassium tartrate solution. The mixture was stirred for 30 min and then extracted with ethyl acetate (2 × 100 mL). The combined organic solutions were washed with a saturated aqueous sodium chloride solution (100 mL). The washed solution was dried over sodium sulfate and filtered. The filtrate was concentrated under reduced pressure and the residue was purified by flash chromatography on silica gel (50% ethyl acetate in petroleum ether) which afforded diol **15** as a 12:1 mixture of *trans*- and *cis*-isomers respectively (white solid, 232 mg, 783 µmol, 52% over 2 steps).

TLC (60% ethyl acetate in cyclohexane): R_f = 0.38 (UV, $KMnO_4$).

Note: Only the NMR signals of the major *trans*-isomer are reported.

^{1}H NMR (500 MHz, DMSO-d_6): δ 6.94 (s, 1H), 6.77 (s, 1H), 6.37 (d, J = 5.1 Hz, 1H), 5.95 (dd, J = 9.7, 1.0 Hz, 2H), 4.96 – 4.90 (m, 2H), 4.68 (d, J = 6.2 Hz, 1H), 4.09 (dd, J = 9.0, 7.0 Hz, 1H), 4.00 (d, J = 9.4 Hz, 1H), 3.73 – 3.67 (m, 4H), 3.49 (d, J = 9.5 Hz, 1H), 2.23 (td, J = 6.8, 2.3 Hz, 1H) ppm.

^{13}C NMR (126 MHz, DMSO-d_6): δ 150.9, 146.7, 140.6, 122.6, 105.7, 100.9, 97.0, 95.0, 91.0, 83.0, 77.6, 68.3, 58.3, 56.4 ppm.

IR (ATR, neat): $\tilde{\nu}$ = 3364 (*br, w*), 2902 (*w*), 1505 (*w*), 1484 (*m*), 1429 (*w*), 1264 (*w*), 1193 (*m*), 1166 (*m*), 1036 (*s*), 997 (*m*), 934 (*w*), 886 (*w*), 859 (*w*) cm^{-1}.

HRMS (ESI): calcd. for $C_{14}H_{17}O_7$ $[M+H]^+$: 319.0788; found: 319.0782.

The analytical data matched those reported previously.[6]

Synthesis of carbonate 30

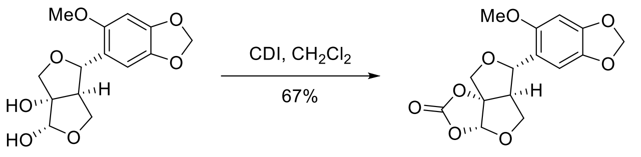

To a solution of diol **15** (80.0 mg, 270 µmol, 1 equiv) in dichloromethane (2 mL) was added 1,1´-carbonyldiimidazole (52.5 mg, 324 µmol, 1.20 equiv) and the resulting solution was stirred for 15 min. The solution was concentrated under reduced pressure and the residue was purified by flash chromatography on silica gel (25% ethyl acetate in petroleum ether grading to 30% ethyl acetate in petroleum ether) which afforded carbonate **30** as a white solid (58.3 mg, 181 µmol, 67%).

TLC (30% ethyl acetate in cyclohexane): R_f = 0.38 (UV, $KMnO_4$).

^{1}H NMR (400 MHz, $CDCl_3$): δ 6.97 (d, *J* = 0.6 Hz, 1H), 6.53 (s, 1H), 6.05 (s, 1H), 5.94 (q, *J* = 1.4 Hz, 2H), 4.93 (d, *J* = 8.5 Hz, 1H), 4.48 (d, *J* = 10.8 Hz, 1H), 4.36 (dd, *J* = 10.0, 1.2 Hz, 1H), 4.22 (dd, *J* = 9.9, 5.6 Hz, 1H), 4.13 (d, *J* = 10.8 Hz, 1H), 3.77 (s, 3H), 2.91 (ddd, *J* = 8.6, 5.6, 1.2 Hz, 1H) ppm.

^{13}C NMR (101 MHz, $CDCl_3$): δ 152.2, 151.8, 148.3, 141.8, 119.4, 108.0, 106.1, 101.6, 100.0, 94.6, 80.8, 71.7, 70.0, 57.7, 56.4 ppm.

IR (ATR, neat): $\tilde{\nu}$ = 2899 (*w*), 1812 (*s*), 1622 (*w*), 1504 (*w*), 1485 (*m*), 1467 (*w*), 1428 (*m*), 1354 (*w*), 1317 (*w*), 1285 (*w*), 1261 (*w*), 1213 (*w*), 1192 (*m*), 1164 (*w*), 1149 (*w*), 1110 (*m*), 1057 (*w*), 1038 (*m*), 1002 (*s*), 936 (*m*), 864 (*w*), 827 (*w*) cm^{-1}.

HRMS (ESI): calcd. for $C_{15}H_{14}NaO_8$ $[M+Na]^+$: 345.0581; found: 345.0579.

Synthesis of acetals 6-*epi*-29a and 29a

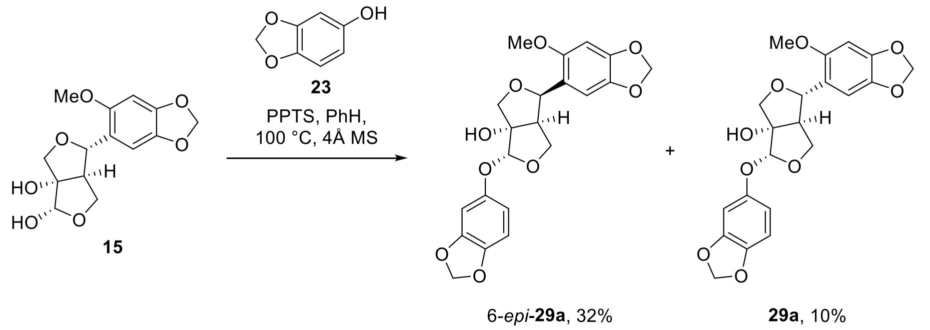

A mixture of diol **15** (20.0 mg, 67.5 µmol, 1 equiv), pyridinium 4-methylbenzenesulfonate (17.0 mg, 67.5 µmol, 1.00 equiv) and phenol **23** (93.2 mg, 675 mmol, 10.0 equiv) in benzene (1 mL) was heated to 100 °C in a sealed vial. After 4 h, the reaction mixture was allowed to cool to 22 °C. The solution was diluted with diethyl ether (3 mL) and dichloromethane (1 mL) and washed with an aqueous solution of sodium hydroxide (2 M, 5 mL). The aqueous layer was extracted with diethyl ether (3 mL). The organic solutions were combined and washed with a saturated aqueous sodium chloride solution (5 mL). The washed organic solution was dried over sodium sulfate and filtered. The filtrate was concentrated under reduced pressure and the residue was purified by flash chromatography on silica gel (15% ethyl acetate in petroleum ether) which afforded a mixture of acetals **29a** and 6-*epi*-**29a** which were further separated using semipreparative HPLC to afford acetal 6-*epi*-**29a** as a colorless oil (8.9 mg, 21 µmol, 32%) and acetal **29a** as a colorless oil (2.8 mg, 6.7 µmol, 10%).

Analytical data for acetal 6-*epi*-**29a**:

^{1}H NMR (500 MHz, $CDCl_3$): δ 7.05 (d, J = 0.8 Hz, 1H), 6.71 (d, J = 8.4 Hz, 1H), 6.64 (d, J = 2.4 Hz, 1H), 6.53 (dd, J = 8.5, 2.4 Hz, 1H), 6.49 (s, 1H), 5.92 (d, J = 7.0 Hz, 4H), 5.34 (d, J = 9.6 Hz, 2H), 4.16 (d, J = 10.0 Hz, 1H), 3.99 (d, J = 10.0 Hz, 1H), 3.93 (t, J = 9.1 Hz, 1H), 3.75 (s, 3H), 3.53 (dd, J = 9.5, 4.2 Hz, 1H), 3.16 (s, 1H), 3.03 (dt, J = 8.8, 4.5 Hz, 1H) ppm.

The analytical data matched those reported previously.[7]

Analytical data for acetal **29a**:

^{1}H NMR (400 MHz, $CDCl_3$): δ 7.15 (d, J = 0.7 Hz, 1H), 6.72 (d, J = 8.4 Hz, 1H), 6.67 (d, J = 2.4 Hz, 1H), 6.56 (dd, J = 8.4, 2.4 Hz, 1H), 6.51 (s, 1H), 5.94 (s, 2H), 5.92 (q, J = 1.4 Hz, 2H), 5.29 (s, 1H), 4.93 (d, J = 6.2 Hz, 1H), 4.39 – 4.30 (m, 2H), 4.08 (dd, J = 9.4, 2.5 Hz, 1H), 3.79 – 3.74 (m, 4H), 3.12 (d, J = 1.9 Hz, 1H), 2.56 (td, J = 6.8, 2.4 Hz, 1H) ppm.

The analytical data matched those reported previously.[7]

Synthesis of phrymarolin II (2a)

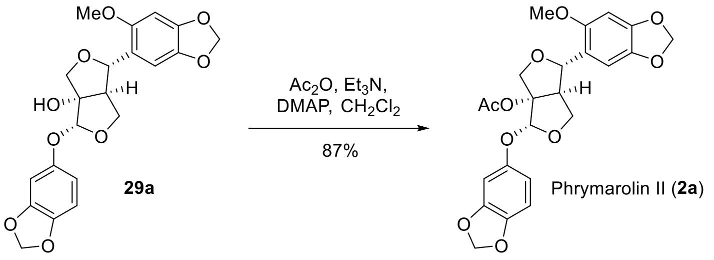

To a solution of alcohol **29a** (2.4 mg, 5.8 µmol, 1 equiv) in dichloromethane (0.1 mL) was added triethylamine (2.4 µL, 17 µmol, 3.0 equiv), *N*,*N*-dimethylaminopyridine (0.1 mg, 0.6 µmol, 0.1 equiv) and acetic anhydride (1.1 µL, 12 µmol, 2.0 equiv). After 6 h, a saturated aqueous ammonium chloride solution was added (1 mL) and the mixture was extracted with dichloromethane (3 × 1 mL). The combined organic solutions were dried over sodium sulfate and filtered. The filtrate was concentrated under reduced pressure which afforded phrymarolin II (**2a**) as a colorless oil (2.3 mg, 5.0 µmol, 87%).

TLC (30% ethyl acetate in cyclohexane): R_f = 0.55 (UV, $KMnO_4$).

^{1}H NMR (400 MHz, $CDCl_3$): *δ* 7.05 (s, 1H), 6.69 (dd, *J* = 8.5, 1.7 Hz, 1H), 6.58 (d, *J* = 2.3 Hz, 1H), 6.52 (s, 1H), 6.49 (dt, *J* = 8.5, 2.2 Hz, 1H), 5.93 (s, 2H), 5.92 (s, 2H), 5.70 (s, 1H), 4.87 (d, *J* = 7.0 Hz, 1H), 4.60 (d, *J* = 11.1 Hz, 1H), 4.29 (dd, *J* = 9.2, 6.8 Hz, 1H), 4.06 (d, *J* = 9.2 Hz, 1H), 3.81 (dd, *J* = 11.1, 1.7 Hz, 1H), 3.77 (d, *J* = 1.6 Hz, 3H), 2.84 (t, *J* = 7.0 Hz, 1H), 2.14 (s, 3H) ppm.

^{13}C NMR (101 MHz, $CDCl_3$): *δ* 170.9, 152.0, 151.6, 148.2, 147.7, 143.5, 141.5, 121.5, 110.6, 108.2, 106.5, 103.2, 101.44, 101.40, 101.36, 96.6, 94.4, 83.0, 75.8, 68.8, 56.6, 56.4, 21.3 ppm.

IR (ATR, neat): $\tilde{\nu}$ = 2961 (*w*), 2896 (*w*), 1739 (*m*), 1632 (*w*), 1503 (*w*), 1484 (*s*), 1428 (*w*), 1371 (*w*), 1345 (*w*), 1282 (*w*), 1240 (*s*), 1207 (*w*), 1189 (*m*), 1118 (*w*), 1095 (*w*), 1068 (*w*), 1037 (*m*), 1026 (*w*), 934 (*w*), 861 (*w*), 818 (*w*), 792 (*w*), 733 (*w*) cm^{-1}.

HRMS (ESI): calcd. for $C_{23}H_{22}NaO_{10}$ $[M+Na]^+$: 481.1105; found: 481.1095.

The analytical data matched those reported previously.[7]

Synthesis of acetals 6-*epi*-29b and 29b

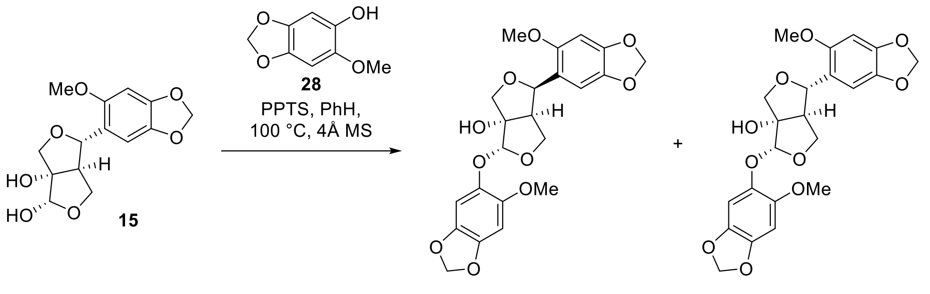

A mixture of diol **15** (20.0 mg, 67.5 μmol, 1 equiv), pyridinium 4-methylbenzenesulfonate (17.0 mg, 67.5 μmol, 1.00 equiv) and phenol **28** (114 mg, 675 mmol, 10.0 equiv) in benzene (1 mL) was heated to 100 °C in a sealed vial. After 4 h, the reaction mixture was allowed to cool to 22 °C. The solution was diluted with diethyl ether (3 mL) and dichloromethane (1 mL) and washed with an aqueous solution of sodium hydroxide (2 M, 5 mL). The aqueous layer was extracted with diethyl ether (3 mL). The organic solutions were combined and washed with a saturated aqueous sodium chloride solution (5 mL). The washed organic solution was dried over sodium sulfate and filtered. The filtrate was concentrated under reduced pressure and the residue was purified by flash chromatography on silica gel (15% ethyl acetate in petroleum ether grading to 20% ethyl acetate in petroleum ether) which afforded a mixture of acetals 6-*epi*-**29b** and **29b** which were further separated using semipreparative HPLC to afford acetal 6-*epi*-**29b** as a colorless oil (4.9 mg, 11 μmol, 16%) and acetal **29b** as a colorless oil (6.3 mg, 14 μmol, 22%).

Characterization data for acetal 6-*epi*-**29b**:

TLC (20% ethyl acetate in cyclohexane): R_f = 0.28 (UV, $KMnO_4$).

^{1}H NMR (400 MHz, $CDCl_3$): δ 7.05 (d, J = 0.7 Hz, 1H), 6.75 (s, 1H), 6.56 (s, 1H), 6.49 (s, 1H), 5.95 – 5.87 (m, 4H), 5.33 (d, J = 4.8 Hz, 1H), 5.20 (s, 1H), 4.11 (d, J = 10.0 Hz, 1H), 4.02 (t, J = 9.1 Hz, 1H), 3.97 (d, J = 9.9 Hz, 1H), 3.91 (s, 1H), 3.80 (s, 3H), 3.75 (s, 3H), 3.56 (dd, J = 9.4, 4.3 Hz, 1H), 3.07 (dq, J = 8.9, 4.4 Hz, 1H) ppm.

^{13}C NMR (101 MHz, $CDCl_3$): δ 150.6, 147.3, 145.7, 143.7, 141.2, 140.9, 139.8, 119.7, 109.2, 107.3, 103.1, 101.5, 101.3, 95.7, 94.1, 90.0, 78.9, 76.2, 68.3, 57.0, 56.2, 51.3 ppm.

IR (ATR, neat): $\tilde{\nu}$ = 3503 (*w*), 2943 (*w*), 2894 (*w*), 1630 (*w*), 1504 (*w*), 1485 (*s*), 1466 (*w*), 1454 (*w*), 1428 (*m*), 1380 (*w*), 1340 (*w*), 1281 (*w*), 1192 (*w*), 1160 (*s*), 1073 (*w*), 1036 (*s*), 1006 (*w*), 982 (*w*), 932 (*m*), 874 (*w*), 860 (*w*), 832 (*w*), 760 (*w*), 731 (*m*) cm^{-1}.

HRMS (ESI): calcd. for $C_{22}H_{22}NaO_{10}$ $[M+Na]^+$: 469.1105; found: 469.1097.

Analytical data for acetal **29b**:

^{1}H NMR (400 MHz, $CDCl_3$): δ 7.16 (d, J = 0.6 Hz, 1H), 6.79 (s, 1H), 6.56 (s, 1H), 6.51 (s, 1H), 5.91 (dp, J = 3.3, 1.6 Hz, 4H), 5.18 (s, 1H), 4.94 (d, J = 6.0 Hz, 1H), 4.45 (dd, J = 9.3, 7.4 Hz, 1H), 4.33 (d, J = 9.7 Hz, 1H), 4.09 (dd, J = 9.3, 2.6 Hz, 1H), 3.81 (d, J = 1.7 Hz, 1H), 3.80 – 3.72 (m, 7H), 2.64 – 2.55 (m, 1H) ppm.

The analytical data matched those reported previously.[8]

Synthesis of phrymarolin I (2b)

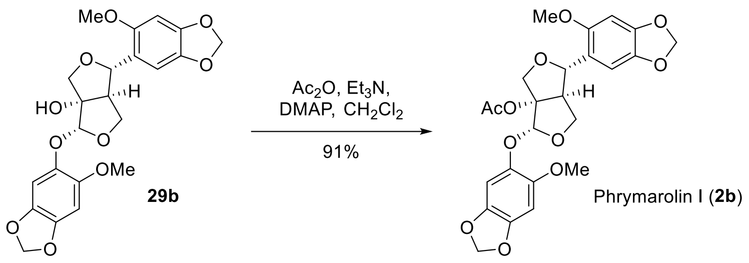

To a solution of alcohol **29b** (6.3 mg, 14 µmol, 1 equiv) in dichloromethane (0.3 mL) was added triethylamine (5.9 µL, 42 µmol, 3.0 equiv), *N,N*-dimethylaminopyridine (0.2 mg, 1 µmol, 0.1 equiv) and acetic anhydride (2.7 µL, 28 µmol, 2.0 equiv). After 10 h, a saturated aqueous ammonium chloride solution was added (1 mL) and the mixture was extracted with dichloromethane (3 × 1 mL). The combined organic solutions were dried over sodium sulfate and filtered. The filtrate was concentrated under reduced pressure which afforded phrymarolin I (**2b**) as a colorless oil (6.3 mg, 13 µmol, 91%).

TLC (20% ethyl acetate in cyclohexane): R_f = 0.28 (UV, $KMnO_4$).

^{1}H NMR (400 MHz, $CDCl_3$): δ 7.05 (s, 1H), 6.83 (s, 1H), 6.54 – 6.50 (m, 2H), 5.96 – 5.90 (m, 2H), 5.90 – 5.85 (m, 2H), 5.68 (s, 1H), 4.88 (d, J = 6.9 Hz, 1H), 4.62 (dt, J = 11.1, 0.8 Hz, 1H), 4.40 (dd, J = 9.2, 6.9 Hz, 1H), 4.05 (dd, J = 9.1, 1.9 Hz, 1H), 3.81 (d, J = 11.1 Hz, 1H), 3.77 (s, 3H), 3.74 (s, 3H), 2.90 (td, J = 6.9, 1.7 Hz, 1H), 2.13 (s, 3H) ppm.

^{13}C NMR (101 MHz, $CDCl_3$): δ 171.0, 151.6, 147.6, 145.8, 143.0, 141.5, 141.0, 140.5, 121.6, 106.5, 104.1, 101.9, 101.4, 101.3, 96.6, 96.3, 94.4, 83.1, 75.8, 69.0, 57.1, 56.4 (2C), 21.3 ppm.

IR (ATR, neat): $\tilde{\nu}$ = 2961 (*w*), 2897 (*w*), 1739 (*m*), 1622 (*w*), 1504 (*w*), 1484 (*s*), 1467 (*w*), 1454 (*w*), 1428 (*m*), 1368 (*w*), 1345 (*w*), 1282 (*w*), 1239 (*m*), 1191 (*w*), 1161 (*s*), 1118 (*w*), 1066 (*w*), 1036 (*s*), 987 (*w*), 934 (*m*), 861 (*m*), 821 (*w*), 794 (*w*), 758 (*w*), 731 (*m*) cm^{-1}.

HRMS (ESI): calcd. for $C_{24}H_{24}NaO_{11}$ $[M+Na]^+$: 511.1211; found: 511.1202.

The analytical data matched those reported previously.[8]

Improved synthesis of the furofuran core:

Synthesis of aryl bromide 33

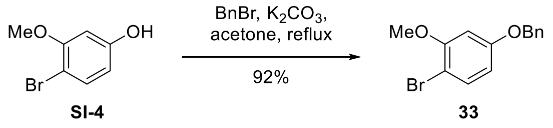

To a solution of phenol **SI-4** (815 mg, 4.01 mmol, 1 equiv) in acetone (8 mL) were added potassium carbonate (1.66 g, 12.0 mmol, 3.00 equiv) and benzyl bromide (525 µL, 4.42 mmol, 1.10 equiv) and the resulting suspension was stirred and heated to reflux. After 4 h, the suspension was allowed to cool to 22 °C. The suspension was filtered and the filtrate was concentrated under reduced pressure. Purification of the residue by flash chromatography on silica gel (10% diethyl ether in petroleum ether) afforded aryl bromide **33** as a white solid (1.08 g, 3.68 mmol, 92%).

^{1}H NMR (400 MHz, $CDCl_3$): δ 7.45 – 7.31 (m, 6H), 6.57 (d, J = 2.7 Hz, 1H), 6.47 (dd, J = 8.7, 2.7 Hz, 1H), 5.05 (s, 2H), 3.85 (s, 3H) ppm.

The analytical data matched those reported previously.[9]

Synthesis of styrene 31

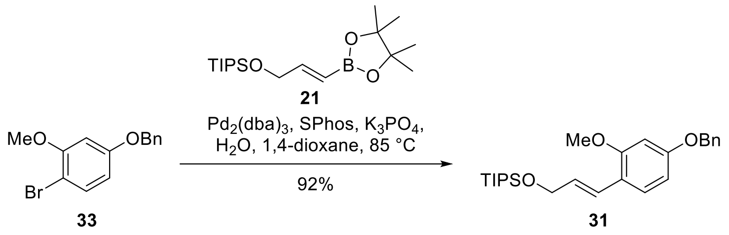

A mixture of aryl bromide **33** (880 mg, 3.00 mmol, 1 equiv), boronic ester **21** (1.28 g, 3.75 mmol, 1.25 equiv), tripotassium phosphate (2.55 g, 12.0 mmol, 4.00 equiv), SPhos (123 mg, 300 µmol, 0.100 equiv), $Pd_2(dba)_3$ (68.7 mg, 75.0 µmol, 2.5 mol%), 1,4-dioxane (8 mL) and water (2 mL), was stirred and heated to 85 °C. After 16 h, the mixture was allowed to cool to 22 °C. Water (20 mL) and ethyl acetate (20 mL) were added. The organic solution was separated and washed with a saturated aqueous solution of sodium chloride (20 mL). The washed organic solution was dried over sodium sulfate and filtered. The filtrate was concentrated under reduced pressure and the residue was purified by flash chromatography on silica gel (3% diethyl ether in petroleum ether grading to 10% diethyl ether in petroleum ether) which afforded styrene **31** as a colorless solid (1.18 g, 2.77 mmol, 92%).

TLC (5% ethyl acetate in cyclohexane): R_f = 0.57 (UV, $KMnO_4$).

^{1}H NMR (400 MHz, $CDCl_3$): δ 7.46 – 7.32 (m, 6H), 6.84 (dt, *J* = 16.0, 1.7 Hz, 1H), 6.56 – 6.50 (m, 2H), 6.21 (dt, *J* = 16.0, 5.2 Hz, 1H), 5.06 (s, 2H), 4.41 (dd, *J* = 5.2, 1.8 Hz, 2H), 3.80 (s, 3H), 1.17 – 1.07 (m, 21H) ppm.

^{13}C NMR (101 MHz, $CDCl_3$): δ 159.5, 157.9, 137.1, 128.8, 128.16, 128.17, 127.8, 127.7, 124.2, 119.8, 105.8, 99.6, 70.3, 64.8, 55.6, 18.2, 12.3 ppm.

IR (ATR, neat): $\tilde{\nu}$ = 2941 (*m*), 2890 (*w*), 2864 (*m*), 1607 (*s*), 1578 (*m*), 1502 (*s*), 1462 (*m*), 1376 (*w*), 1259 (*m*), 1191 (*s*), 1161 (*s*), 1037 (*s*), 966 (*m*), 882 (*m*), 835 (*m*), 766 (*m*), 734 (*m*), 681 (*s*) cm^{-1}.

HRMS (ESI): calcd. for $C_{26}H_{38}NaO_3Si$ $[M+Na]^+$: 449.2482 ; found: 449.2474.

Synthesis of aryl bromide 34

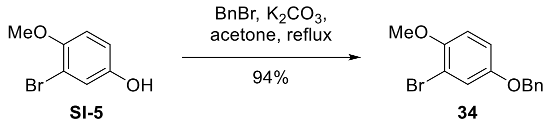

To a solution of phenol **SI-5** (3.00 g, 14.8 mmol, 1 equiv) in acetone (50 mL) were added potassium carbonate (6.13 g, 44.3 mmol, 3.00 equiv) and benzyl bromide (1.93 mL, 16.3 mmol, 1.10 equiv) and the resulting suspension was stirred and heated to reflux. After 4 h, the suspension was allowed to cool to 22 °C. The suspension was filtered and the filtrate was concentrated under reduced pressure. Purification of the residue by flash chromatography on silica gel (10% diethyl ether in petroleum ether) afforded aryl bromide **34** as a white solid (4.06 g, 13.8 mmol, 94%).

^{1}H NMR (400 MHz, $CDCl_3$): δ 7.43 – 7.32 (m, 5H), 7.22 (d, *J* = 2.9 Hz, 1H), 6.89 (dd, *J* = 9.0, 2.9 Hz, 1H), 6.83 (d, *J* = 9.0 Hz, 1H), 5.00 (s, 2H), 3.85 (s, 3H) ppm.

The analytical data matched those reported previously.[10]

Synthesis of styrene 32

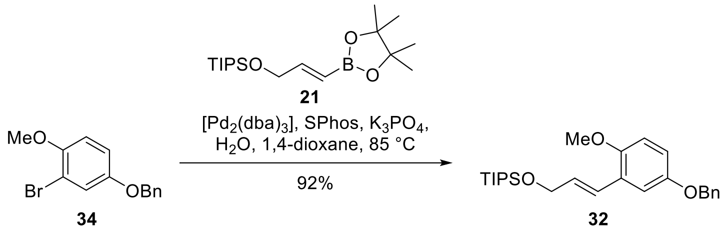

A mixture of aryl bromide **34** (4.00 g, 13.6 mmol, 1 equiv), boronic ester **21** (5.81 g, 17.1 mmol, 1.25 equiv), tripotassium phosphate (11.6 g, 54.6 mmol, 4.00 equiv), SPhos (560 mg, 1.36 mmol, 0.100 equiv), $Pd_2(dba)_3$

(312 mg, 341 µmol, 2.5 mol%), 1,4-dioxane (40 mL) and water (10 mL) was stirred and heated to 85 °C. After 16 h, the mixture was allowed to cool to 22 °C. Water (100 mL) and ethyl acetate (100 mL) were added. The organic solution was separated and washed with a saturated aqueous solution of sodium chloride (100 mL). The washed organic solution was dried over sodium sulfate and filtered. The filtrate was concentrated under reduced pressure and the residue was purified by flash chromatography on silica gel (3% diethyl ether in petroleum ether grading to 10% diethyl ether in petroleum ether) which afforded styrene **34** as a faintly yellow oil (5.80 g, 13.6 mmol, 99%).

TLC (5% ethyl acetate in cyclohexane): R_f = 0.60 (UV, $KMnO_4$).

^{1}H NMR (400 MHz, $CDCl_3$): δ 7.47 – 7.28 (m, 5H), 7.09 (d, J = 2.8 Hz, 1H), 6.92 (dt, J = 16.0, 1.8 Hz, 1H), 6.84 – 6.75 (m, 2H), 6.28 (dt, J = 16.0, 5.0 Hz, 1H), 5.03 (s, 2H), 4.43 (dd, J = 5.0, 1.8 Hz, 2H), 3.79 (s, 3H), 1.21 – 1.03 (m, 21H) ppm.

^{13}C NMR (101 MHz, $CDCl_3$): δ 153.1, 151.6, 137.5, 130.6, 128.7, 128.0, 127.7, 127.5, 124.1, 114.3, 113.8, 112.5, 70.9, 64.5, 56.5, 18.2, 12.3 ppm.

IR (ATR, neat): $\tilde{\nu}$ = 2941 (*m*), 2864 (*m*), 1581 (*w*), 1494 (*s*), 1462 (*m*), 1376 (*m*), 1285 (*m*), 1233 (*m*), 1181 (*m*), 1128 (*m*), 1046 (*m*), 970 (*m*), 882 (*m*), 733 (*m*), 682 (*m*) cm^{-1}.

HRMS (ESI): calcd. for $C_{26}H_{38}NaO_3Si$ [M+Na]$^+$: 449.2482; found: 449.2479.

Synthesis of styrene SI-6

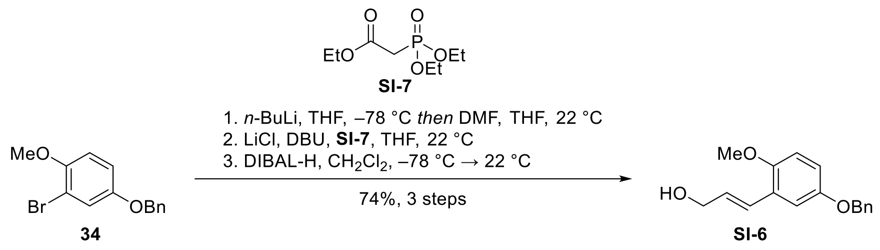

To a solution of aryl bromide **34** (2.00 g, 6.82 mmol, 1 equiv) in tetrahydrofuran (25 mL) cooled to –78 °C was added a solution of *n*-butyllithium (2.50 M in hexanes, 3.00 mL, 7.50 mmol, 1.10 equiv) followed by *N,N*-dimethylformamide (634 µL, 8.19 mmol, 1.20 equiv). After 5 min, the solution was allowed to warm to 22 °C. The solution was cannulated into a flask containing a mixture of triethyl phosphonoacetate **SI-7** (2.43 mL, 12.3 mmol, 1.80 equiv), lithium chloride (0.50 M in tetrahydrofuran, 24.5 mL, 12.3 mmol, 1.80 equiv) and 1,8-diazabicyclo[5.4.0]undec-7-ene (1.85 mL, 12.3 mmol, 1.80 equiv) at 0 °C. The mixture was allowed to warm to 22 °C and was stirred for 30 min. A saturated aqueous ammonium chloride solution (50 mL) and water (50 mL) were added. The organic phase was separated and washed with water (100 mL) and a saturated aqueous sodium chloride solution (100 mL). The washed solution was dried over sodium

sulfate and filtered. The filtrate was concentrated under reduced pressure. The residue was dissolved in dichloromethane (25 mL) and the resulting solution was cooled to –78 °C. A solution of diisobutylaluminium hydride (1.00 M in hexanes, 27.3 mL, 27.3 mmol, 4.00 equiv) was added and the resulting solution was allowed to warm to 22 °C. After 30 min, ethyl acetate (5 mL) was carefully added. The solution was poured into an aqueous saturated sodium potassium tartrate solution (200 mL) and stirred for 2 h. The mixture was extracted with ethyl acetate (2 × 100 mL). The organic solution was washed with a saturated aqueous sodium chloride solution (100 mL). The washed solution was dried over sodium sulfate and filtered. The filtrate was concentrated and the residue was purified by flash chromatography on silica gel (30% ethyl acetate in petroleum ether grading to 40% ethyl acetate in petroleum ether) which afforded alcohol **SI-6** as a colorless oil (1.37 g, 5.07 mmol, 74%).

TLC (30% ethyl acetate in cyclohexane): R_f = 0.33 (UV, $KMnO_4$).

^{1}H NMR (400 MHz, $CDCl_3$): δ 7.47 – 7.28 (m, 5H), 7.09 (d, J = 3.0 Hz, 1H), 6.91 (dt, J = 16.1, 1.5 Hz, 1H), 6.85 (dd, J = 8.9, 3.0 Hz, 1H), 6.79 (d, J = 8.9 Hz, 1H), 6.35 (dt, J = 16.0, 5.9 Hz, 1H), 5.04 (s, 2H), 4.34 (td, J = 5.9, 1.3 Hz, 2H), 3.81 (s, 3H), 1.47 (br t, J = 6.1 Hz, 1H) ppm.

^{13}C NMR (101 MHz, $CDCl_3$): δ 153.0, 151.6, 137.4, 129.7, 128.7, 128.1, 127.6, 126.7, 126.1, 115.0, 113.7, 112.2, 70.9, 64.3, 56.3 ppm.

IR (ATR, neat): $\tilde{\nu}$ = 3357 (*br*, *w*), 3033 (w), 2939 (*w*), 2906 (*w*), 2865 (*w*), 2834 (*w*), 1605 (*w*), 1571 (*w*), 1494 (*s*), 1462 (*m*), 1423 (*w*), 1380 (*w*), 1285 (*w*), 1221 (*s*), 1181 (*m*), 1121 (*w*), 1082 (*w*), 1026 (*m*), 972 (*m*), 913 (*w*), 874 (*w*), 906 (*w*), 735 (*m*), 697 (*m*) cm^{-1}.

HRMS (ESI): calcd. for $C_{17}H_{18}KO_3$ $[M+K]^+$: 309.0888; found: 309.0877.

Synthesis of styrene 32

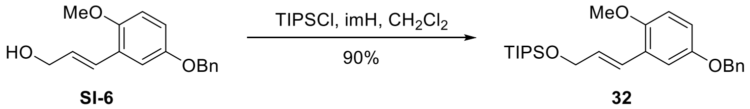

To a solution of alcohol **SI-6** (5.00 g, 18.5 mmol, 1 equiv) in dichloromethane (70 mL) was added imidazole (1.51 g, 22.2 mmol, 1.20 equiv) followed by triisopropylsilyl chloride (4.35 mL, 20.3 mmol, 1.10 equiv) and the resulting suspension was allowed to stir for 3 h. The mixture was washed with water (100 mL) and with a saturated aqueous sodium chloride solution (50 mL). The washed organic solution was dried over sodium sulfate and filtered. The filtrated was concentrated under reduced pressure and the residue was purified by flash chromatography on silica gel (10% diethyl ether in petroleum ether) which afforded styrene **32** as a colorless oil (7.08 g, 16.6 mmol, 90%).

The analytical data matched those obtained before (see above).

Synthesis of alcohols 35

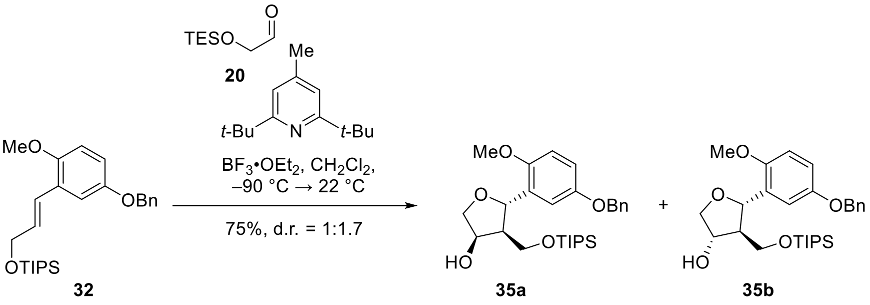

To a solution of styrene **32** (4.00 g, 9.37 mmol, 1 equiv), aldehyde **20** (3.27 g, 18.7 mmol, 2.00 equiv) and 2,6-di-*tert*-butyl-4-methylpyridine (4.24 g, 20.6 mmol, 2.20 equiv) in dichloromethane (70 mL) cooled to –90 °C was added dropwise BF_3•OEt_2 (2.38 mL, 18.7 mmol, 2.00 equiv). After 5 min, the solution was allowed to warm to 0 °C. After 5 min at 0 °C, the solution was poured into a flask containing a saturated aqueous sodium bicarbonate solution (150 mL) and the resulting mixture was stirred for 30 min. The organic layer was separated and the aqueous one was extracted with dichloromethane (100 mL). The organic solutions were combined and washed with a saturated aqueous solution of sodium chloride (100 mL). The washed organic solution was dried over sodium sulfate and filtered. The filtrate was concentrated under reduced pressure and the residue was purified by flash chromatography on silica gel (15% ethyl acetate in petroleum ether grading to 20% ethyl acetate in petroleum ether) afforded alcohols **35a** and **35b** (inconsequential 1:1.7 mixture of diastereomers) as a colorless viscous oil (3.41 g, 7.01 mmol, 75%).

Note: For the purpose of characterization the mixture was separated using a fully automated medium performance liquid chromatography (15% ethyl acetate in petroleum ether grading to 20% ethyl acetate in petroleum ether).

Characterization data for the minor isomer **35a**:

TLC (20% ethyl acetate in cyclohexane): R_f = 0.39 (UV, $KMnO_4$).

^{1}H NMR (400 MHz, $CDCl_3$): δ 7.45 – 7.29 (m, 5H), 7.17 (d, J = 3.0 Hz, 1H), 6.84 (dd, J = 8.8, 3.0 Hz, 1H), 6.78 (d, J = 8.9 Hz, 1H), 5.03 (s, 2H), 4.86 (d, J = 6.5 Hz, 1H), 4.44 (ddt, J = 6.4, 4.3, 3.2 Hz, 1H), 4.06 – 3.95 (m, 3H), 3.82 – 3.74 (m, 4H), 2.44 (d, J = 6.4 Hz, 1H), 2.32 (tq, J = 7.2, 4.4, 3.7 Hz, 1H), 1.13 – 1.04 (m, 21H) ppm.

^{13}C NMR (101 MHz, $CDCl_3$): δ 153.2, 150.9, 137.4, 131.6, 128.7, 128.0, 127.7, 114.4, 114.2, 111.7, 78.4, 76.9 (overlapping with the $CDCl_3$ signal), 75.0, 70.8, 64.0, 58.0, 55.9, 18.1, 12.0 ppm.

IR (ATR, neat): $\tilde{\nu}$ = 3441 (*br*, *w*), 2942 (*m*), 2891 (*m*), 2865 (*s*), 1498 (*s*), 1463 (*m*), 1382 (*w*), 1367 (*w*), 1278 (*m*), 1219 (*s*), 1179 (*m*), 1113 (*m*), 1065 (*s*), 1044 (*s*), 955 (*m*), 882 (*m*), 804 (*m*), 734 (*m*), 683 (*m*) cm^{-1}.

HRMS (ESI): calcd. for $C_{28}H_{42}O_5NaSi$ $[M+Na]^+$: 487.2874; found: 487.2860.

Characterization data for the major isomer **35b**:

TLC (20% ethyl acetate in cyclohexane): R_f = 0.34 (UV, $KMnO_4$).

^{1}H NMR (400 MHz, $CDCl_3$): δ 7.52 – 7.27 (m, 5H), 7.06 (d, *J* = 3.0 Hz, 1H), 6.82 (dd, *J* = 8.9, 3.0 Hz, 1H), 6.77 (d, *J* = 8.9 Hz, 1H), 5.16 (d, *J* = 7.9 Hz, 1H), 5.02 (d, *J* = 1.2 Hz, 2H), 4.60 (tt, *J* = 4.6, 3.3 Hz, 1H), 4.21 (ddd, *J* = 9.5, 4.8, 0.9 Hz, 1H), 4.13 – 4.06 (m, 2H), 3.93 (dd, *J* = 9.5, 3.0 Hz, 1H), 3.75 (s, 3H), 3.54 (dd, *J* = 4.0, 0.8 Hz, 1H), 2.35 (tt, *J* = 6.8, 5.7 Hz, 1H), 1.16 – 1.04 (m, 21H) ppm.

^{13}C NMR (101 MHz, $CDCl_3$): δ 153.1, 151.2, 137.4, 131.6, 128.7, 128.0, 127.7, 114.0, 113.9, 111.4, 76.5, 75.2, 74.5, 70.8, 61.9, 55.8, 52.8, 18.07, 18.06, 11.9 ppm.

IR (ATR, neat): $\tilde{\nu}$ = 3456 (*br*, *w*), 2942 (*m*), 2891 (*m*), 2865 (*m*), 1498 (*s*), 1463 (*m*), 1433 (*w*), 1382 (*w*), 1278 (*m*), 1179 (*m*), 1164 (m), 1114 (*m*), 1082 (*m*), 1046 (*s*), 1028 (*m*), 882 (*m*), 802 (*m*), 732 (*m*), 660 (*m*) cm^{-1}.

HRMS (ESI): calcd. for $C_{28}H_{42}O_5NaSi$ $[M+Na]^+$: 487.2874; found: 487.2860.

Synthesis of ketone 36

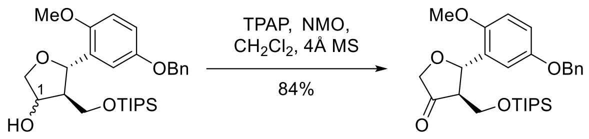

35, d.r. = 1.7:1 at C1 **36**

To a solution of the mixture of alcohols **35** (6.18 g, 12.7 mmol, 1 equiv) in dichloromethane (100 mL) were added 4Å molecular sieves (10 g), *N*-methymorpholine *N*-oxide (2.23 g, 19.0 mmol, 1.50 equiv) and tetrapropylammonium perruthenate (223 mg, 350 µmol, 5 mol%). After stirring for 2 h, the mixture was filtered through celite and the filtrate was concentrated under reduced pressure. Purification of the residue by flash chromatography on silica gel (10% ethyl acetate in petroleum ether) afforded ketone **36** as a colorless solid (5.19 g, 10.7 mmol, 84%).

TLC (10% ethyl acetate in cyclohexane): R_f = 0.46 (UV, $KMnO_4$).

^{1}H NMR (400 MHz, $CDCl_3$): δ 7.47 – 7.27 (m, 5H), 7.12 (d, J = 3.0 Hz, 1H), 6.88 (dd, J = 8.9, 3.0 Hz, 1H), 6.82 (d, J = 9.0 Hz, 1H), 5.71 (d, J = 7.7 Hz, 1H), 5.08 – 4.99 (m, 2H), 4.27 (dd, J = 16.7, 1.0 Hz, 1H), 4.19 (dd, J = 9.7, 3.3 Hz, 1H), 3.99 (d, J = 16.7 Hz, 1H), 3.93 (dd, J = 9.7, 2.8 Hz, 1H), 3.77 (s, 3H), 2.52 – 2.47 (m, 1H), 1.14 – 1.02 (m, 21H) ppm.

^{13}C NMR (101 MHz, $CDCl_3$): δ 215.8, 153.2, 151.5, 137.3, 130.5, 128.7, 128.1, 127.6, 114.9, 114.8, 111.8, 77.5 (overlapping with the $CDCl_3$ signal), 72.5, 70.9, 60.8, 56.3, 55.8, 18.1, 18.0, 12.0 ppm.

IR (ATR, neat): $\tilde{\nu}$ = 2942 (*m*), 2892 (*m*), 2866 (*m*), 1762 (*s*), 1592 (*w*), 1502 (*s*), 1463 (*m*), 1381 (*m*), 1306 (*m*), 1279 (*m*), 1248 (*m*), 1218 (*m*), 1177 (*m*), 1165 (*m*), 1111 (*m*), 1070 (*m*), 1028 (*m*), 997 (*m*), 973 (*m*), 918 (*m*), 882 (*m*), 818 (*m*), 735 (*m*), 694 (*m*) cm^{-1}.

HRMS (ESI): calcd. for $C_{28}H_{40}NaO_5Si$ $[M+Na]^+$: 507.2537; found: 507.2538.

Synthesis of alcohol 37

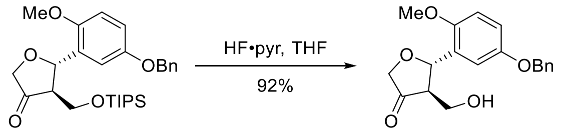

To a solution of ketone **36** (1.00 g, 2.06 mmol, 1 equiv) in tetrahydrofuran (10 mL) was added dropwise hydrogen fluoride-pyridine complex (2.03 mL, 70 wt% of HF, 40.0 equiv) at 22 °C. The resulting solution was stirred and heated to 40 °C. After 6 h, the solution was added to an aqueous solution prepared from an aqueous sodium hydroxide solution (2 M, 35 mL) and an aqueous saturated sodium bicarbonate solution (50 mL). The mixture was stirred for 15 min and then was extracted with ethyl acetate (2 × 50 mL). The organic solutions were combined and washed with an aqueous saturated sodium chloride solution (50 mL). The washed solution was dried over sodium sulfate and filtered. The filtrate was concentrated under reduced pressure and the residue was purified by flash chromatography on silica gel (35% ethyl acetate in petroleum ether) which afforded alcohol **37** as a colorless oil (624 mg, 1.90 mmol, 92%).

TLC (40% ethyl acetate in cyclohexane): R_f = 0.34 (UV, $KMnO_4$).

^{1}H NMR (400 MHz, $CDCl_3$): δ 7.46 – 7.30 (m, 5H), 7.22 (d, J = 3.0 Hz, 1H), 6.91 (dd, J = 8.9, 3.0 Hz, 1H), 6.85 (d, J = 8.9 Hz, 1H), 5.42 (d, J = 9.3 Hz, 1H), 5.08 – 5.00 (m, 2H), 4.35 (dd, J = 17.1, 0.7 Hz, 1H), 4.06 – 3.96 (m, 2H), 3.89 – 3.80 (m, 4H), 2.54 – 2.47 (m, 1H), 2.44 (dd, J = 7.6, 5.8 Hz, 1H) ppm.

^{13}C NMR (101 MHz, $CDCl_3$): δ 215.7, 153.7, 150.6, 137.2, 129.5, 128.7, 128.1, 127.7, 115.0, 113.7, 112.0, 76.8, 72.1, 70.8, 59.4, 57.4, 56.3 ppm.

IR (ATR, neat): $\tilde{\nu}$ = 3478 (*br*, *w*), 2937 (*w*), 2879 (*w*), 2837 (*w*), 1755 (*s*), 1591 (*w*), 1499 (*s*), 1463 (*m*), 1455 (*m*), 1433 (*m*), 1373 (*m*), 1279 (*m*), 1217 (*s*), 1183 (*m*), 1042 (*s*), 1028 (*s*), 957 (*w*), 925 (*w*), 805 (*w*), 737 (*m*) cm^{-1}.

HRMS (ESI): calcd. for $C_{19}H_{20}NaO_5$ [M+Na]$^+$: 351.1203; found: 351.1210.

Two-step synthesis of diol 38

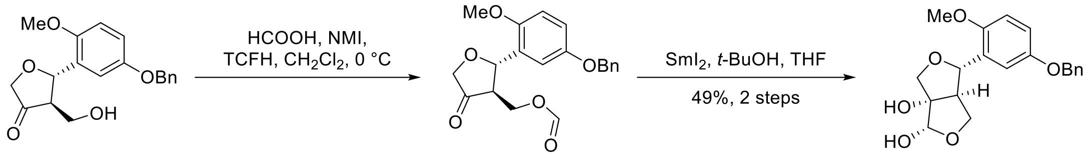

37 **SI-8** **38**

To a solution of alcohol **37** (2.28 g, 6.94 mmol, 1 equiv) in dichloromethane (40 mL) was added *N*-methylimidazole (2.77 mL, 34.7 mmol, 5.00 equiv) and formic acid (1.31 mL, 34.7 mmol, 5.00 equiv). The resulting solution was cooled to 0 °C and *N*,*N*,*N'*,*N'*-tetramethylchloroformamidinium hexafluorophosphate (2.92 g, 10.4 mmol, 1.50 equiv) was added portionwise over 5 min. After stirring for 5 min, the solution was added to a saturated aqueous ammonium chloride solution (100 mL). The mixture was extracted with dichloromethane (100 mL). The organic solution was washed with water (100 mL) and an aqueous saturated sodium chloride solution (100 mL). The washed solution was dried over sodium sulfate and filtered. The filtrate was concentrated under reduced pressure and the residue containing formate ester **SI-8** and tetramethylurea was directly used in the next step without further purification. To a solution of samarium diiodide (0.100 M in tetrahydrofuran, 278 mL, 27.8 mmol, 4.00 equiv) was added *tert*-butanol (6.64 mL, 69.4 mmol, 10.0 equiv) followed by a solution of crude ester **SI-8** (assumed 6.94 mmol, 1 equiv) in tetrahydrofuran (20 mL) and the resulting solution was stirred at 22 °C for 1 h. The solution was then poured into an aqueous solution prepared from 200 mL of a saturated ammonium chloride solution and 400 mL of a saturated sodium potassium tartrate solution. The mixture was stirred for 30 min and then extracted with ethyl acetate (2 × 200 mL). The combined organic solutions were washed with a saturated aqueous sodium chloride solution (200 mL). The washed solution was dried over sodium sulfate and filtered. The filtrate was concentrated under reduced pressure and the residue was purified by flash chromatography on silica gel (50% ethyl acetate in petroleum ether) which afforded diol **38** as a white solid (1.21 g, 3.38 mmol, 49% over 2 steps).

TLC (60% ethyl acetate in cyclohexane): R_f = 0.40 (UV, $KMnO_4$).

^{1}H NMR (400 MHz, $CDCl_3$): δ 7.46 – 7.28 (m, 6H), 6.84 (dd, *J* = 8.8, 3.0 Hz, 1H), 6.78 (d, *J* = 8.9 Hz, 1H), 5.21 (d, *J* = 3.4 Hz, 1H), 5.06 – 5.02 (m, 2H), 4.89 (d, *J* = 6.5 Hz, 1H), 4.35 (dd, *J* = 9.3, 7.1 Hz, 1H), 4.28 (d, *J* = 9.9 Hz, 1H), 4.05 (dd, *J* = 9.3, 2.4 Hz, 1H), 3.79 – 3.74 (m, 4H), 3.41 (br d, *J* = 3.3 Hz, 1H), 3.13 (s, 1H), 2.52 (td, *J* = 6.9, 2.4 Hz, 1H) ppm.

^{13}C NMR (101 MHz, $CDCl_3$): δ 153.2, 150.5, 137.4, 130.9, 128.7, 128.0, 127.7, 114.3, 113.2, 111.3, 97.7, 92.4, 84.3, 77.4, 70.8, 70.2, 58.8, 55.9 ppm.

IR (ATR, neat): $\tilde{\nu}$ = 3395 (*br, m*), 2946 (*w*), 2899 (*w*), 2863 (*w*), 2837 (*w*), 1608 (*w*), 1591 (*w*), 1498 (*s*), 1463 (*m*), 1455 (*m*), 1432 (*m*) 1278 (*m*), 1220 (*s*), 1181 (*m*), 1074 (*m*), 1027 (*s*), 914 (*w*), 852 (*w*), 780 (*w*), 737 (*m*), 697 (*m*) cm^{-1}.

HRMS (ESI): calcd. for $C_{20}H_{22}NaO_6$ $[M+Na]^+$: 381.1309; found: 381.1310.

Synthesis of acetal 39

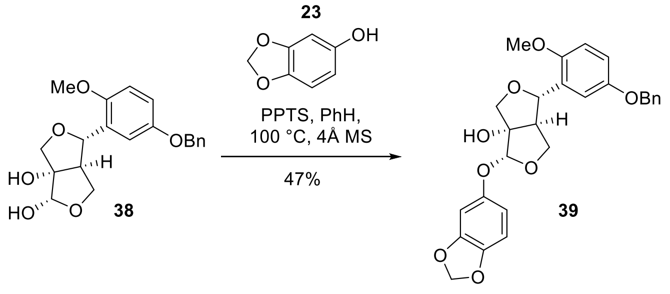

A mixture of diol **38** (800 mg, 2.23 mmol, 1 equiv), pyridinium 4-methylbenzenesulfonate (561 mg, 2.23 mmol, 1.00 equiv), phenol **23** (3.08 g, 22.3 mmol, 10.0 equiv) and molecular sieves (4Å, 1.00 g) in benzene (15 mL) was heated to 100 °C in a sealed vial. After 4 h, the reaction mixture was allowed to cool to 22 °C. The solution was diluted with diethyl ether (20 mL) and dichloromethane (20 mL) and washed with an aqueous solution of sodium hydroxide (2 M, 2 × 20 mL). The aqueous layer was extracted with diethyl ether (2 × 20 mL). The organic solutions were combined and washed with a saturated aqueous sodium chloride solution (20 mL). The washed organic solution was dried over sodium sulfate and filtered. The filtrate was concentrated under reduced pressure and the residue was purified by flash chromatography on silica gel (15% ethyl acetate in petroleum ether grading to 20% ethyl acetate in petroleum ether) which afforded acetal **39** as a pale-yellow oil (505 mg, 1.06 mmol, 47%).

TLC (30% ethyl acetate in cyclohexane): R_f = 0.50 (UV, $KMnO_4$).

^{1}H NMR (400 MHz, $CDCl_3$): δ 7.47 – 7.29 (m, 6H), 6.85 (dd, *J* = 8.8, 3.1 Hz, 1H), 6.78 (d, *J* = 8.9 Hz, 1H), 6.73 (d, *J* = 8.4 Hz, 1H), 6.68 (d, *J* = 2.4 Hz, 1H), 6.57 (dd, *J* = 8.5, 2.5 Hz, 1H), 5.94 (s, 2H), 5.31 (s, 1H), 5.05 (s, 2H), 4.96 (d, *J* = 6.2 Hz, 1H), 4.37 (m, 2H), 4.15 (dd, *J* = 9.4, 2.5 Hz, 1H), 3.85 – 3.75 (m, 4H), 3.12 (d, *J* = 1.8 Hz, 1H), 2.66 – 2.54 (m, 1H) ppm.

^{13}C NMR (101 MHz, $CDCl_3$): δ 153.2, 151.4, 150.4, 148.4, 143.5, 137.5, 131.0, 128.7, 128.0, 127.7, 114.3, 113.1, 111.1, 110.0, 108.3, 103.3, 101.6, 100.9, 92.3, 84.6, 77.4, 71.1, 70.8, 58.5, 55.8 ppm.

IR (ATR, neat): $\tilde{\nu}$ = 3548 (*w*, *br*), 2898 (*w*), 1610 (*w*), 1499 (*m*), 1485 (*s*), 1371 (*w*), 1276 (*m*), 1245 (*m*), 1219 (*m*), 1181 (*s*), 1129 (*w*), 1070 (*m*), 1037 (*s*), 1006 (*s*), 913 (*m*), 815 (*w*), 736 (*m*), 697 (*w*) cm^{-1}.

HRMS (ESI): calcd. for $C_{27}H_{26}NaO_8$ $[M+Na]^+$: 501.1520; found: 501.1513.

Synthesis of phenol 45

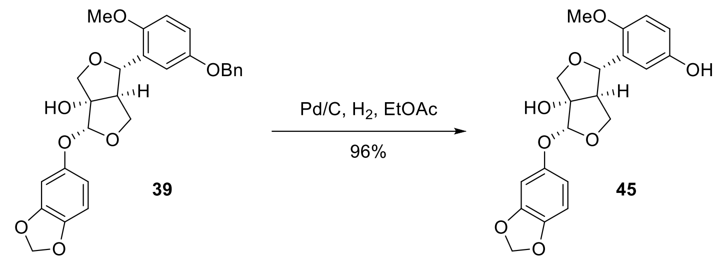

To a solution of acetal **39** (120 mg, 251 µmol, 1 equiv) was added palladium on carbon (13.3 mg, 10 wt% palladium, 12.5 µmol, 5 mol%), the stirred suspension was sparged with hydrogen gas for 30 min and then stirred under the hydrogen atmosphere. After 2 h, the mixture was sparged with argon for 1 min and filtered through celite. The filtrate was concentrated under reduced pressure and the residue was purified by flash chromatography on silica gel (40% ethyl acetate in petroleum ether) which afforded phenol **45** as a beige solid (93.2 mg, 240 µmol, 96%).

TLC (40% ethyl acetate in cyclohexane): R_f = 0.31 (UV, $KMnO_4$).

^{1}H NMR (400 MHz, $CDCl_3$): δ 7.15 (t, *J* = 1.7 Hz, 1H), 6.74 – 6.69 (m, 3H), 6.66 (d, *J* = 2.4 Hz, 1H), 6.55 (dd, *J* = 8.4, 2.4 Hz, 1H), 5.94 (s, 2H), 5.60 – 5.40 (br m, 1H), 5.29 (s, 1H), 4.96 (d, *J* = 6.2 Hz, 1H), 4.41 – 4.31 (m, 2H), 4.14 – 4.09 (m, 1H), 3.78 (d, *J* = 10.1 Hz, 1H), 3.75 (s, 3H), 3.40 (d, *J* = 8.5 Hz, 1H), 2.62 – 2.55 (m, 1H) ppm.

^{13}C NMR (101 MHz, $CDCl_3$): δ 151.4, 150.02, 149.97, 148.3, 143.5, 130.8, 114.6, 113.4, 111.5, 110.1, 108.2, 103.0, 101.5, 100.9, 92.4, 84.4, 77.3, 71.0, 58.4, 55.9 ppm.

IR (ATR, neat): $\tilde{\nu}$ = 3379 (*br*, *w*), 2956 (*w*), 2898 (*w*), 2837 (*w*), 1632 (*w*), 1612 (*w*), 1501 (*w*), 1486 (*s*), 1464 (*w*), 1439 (*w*), 1361 (*w*), 1339 (*w*), 1314 (*w*), 1269 (*w*), 1244 (*w*), 1219 (*w*), 1181 (*s*), 1129 (*w*), 1095 (*w*), 1068 (*w*), 1035 (*m*), 1007 (*w*), 975 (*w*), 947 (*w*), 930 (*w*), 863 (*w*), 815 (*w*), 736 (*m*) cm^{-1}.

HRMS (ESI): calcd. for $C_{20}H_{19}O_8$ $[M-H^+]^-$: 387.1085; found: 387.1090.

Synthesis of catechol 47

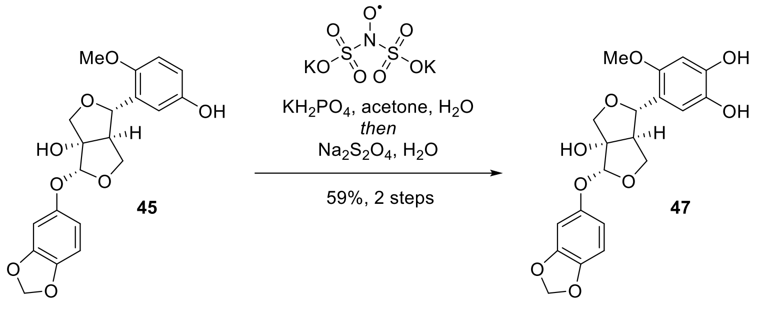

A mixture of potassium dihydrogen phosphate (189 mg, 1.39 mmol, 6.00 equiv), dipotassium nitrosodisulfonate (312 mg, 1.16 mmol, 5.00 equiv), phenol **45** (90.0 mg, 232 µmol, 1 equiv), acetone (2 mL) and water (1.5 mL) was stirred for 4 h. Ethyl acetate (2 mL) was added and the aqueous phase was separated. The aqueous phase was extracted with ethyl acetate (2 mL). The organic solutions were combined and washed with a saturated aqueous sodium chloride solution (2 mL) and then with an aqueous solution of sodium dithionite (239 mg in 1 mL). The organic solution was dried over sodium sulfate and filtered. The filtrate was concentrated under reduced pressure which afforded catechol **47** as a pale-yellow solid (55.4 mg, 137 µmol, 59% over 2 steps).

TLC (60% ethyl acetate in cyclohexane): R_f = 0.44 (UV, $KMnO_4$).

^{1}H NMR (500 MHz, $CDCl_3$): δ 7.12 (s, 1H), 6.72 – 6.69 (m, 1H), 6.65 (d, *J* = 2.5 Hz, 1H), 6.54 (dd, *J* = 8.4, 2.5 Hz, 1H), 6.47 (s, 1H), 5.94 (s, 2H), 5.27 (s, 1H), 4.94 (d, *J* = 6.4 Hz, 1H), 4.37 – 4.29 (m, 2H), 4.04 (dd, *J* = 9.4, 2.4 Hz, 1H), 3.75 – 3.73 (m, 1H), 3.70 (s, 3H), 2.59 (td, *J* = 6.9, 2.4 Hz, 1H) ppm.

^{13}C NMR (126 MHz, $CDCl_3$): δ 151.3, 150.8, 148.3, 144.7, 143.6, 136.7, 120.6, 113.9, 110.1, 108.3, 103.0, 101.6, 100.9, 99.7, 92.5, 84.1, 77.2 (overlapping with the $CDCl_3$ signal) 70.8, 58.4, 56.0 ppm.

IR (ATR, neat): $\tilde{\nu}$ = 3375 (*br, w*), 2899 (*w*), 1615 (*w*), 1502 (*w*), 1486 (*s*), 1307 (*w*), 1181 (*s*), 1128 (*w*), 1008 (*m*), 928 (*w*), 732 (*w*) cm^{-1}.

HRMS (ESI): calcd. for $C_{20}H_{19}O_9$ [M-H$^+$]$^-$: 403.1035; found: 403.1034.

Synthesis of acetal 29a

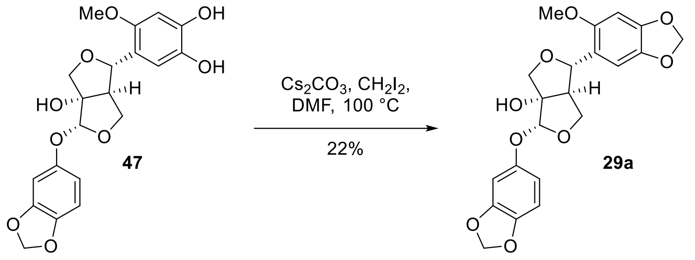

A suspension of catechol **47** (55.4 mg, 137 µmol, 1 equiv), cesium carbonate (112 mg, 343 µmol, 2.50 equiv) and diiodomethane (27.6 µL, 343 µmol, 2.50 equiv) in *N,N*-dimethylformamide (0.5 mL) was stirred and heated to 100 °C. After 1 h, the mixture was allowed to cool to 22 °C. Ethyl acetate (2 mL) was added and the mixture was washed with a saturated aqueous solution of ammonium chloride (2 mL) and then with saturated aqueous solution of lithium chloride (2 × 2 mL). The washed organic solution was dried over sodium sulfate and filtered. The filtrate was concentrated under reduced pressure and the residue was purified by flash chromatography on silica gel (15% ethyl acetate in petroleum ether which afforded acetal **29a** as a colorless oil (12.5 mg, 30.0 µmol, 22%).

The analytical data for acetal **29a** matched those obtained before (see above).

Synthesis of carbonate 54

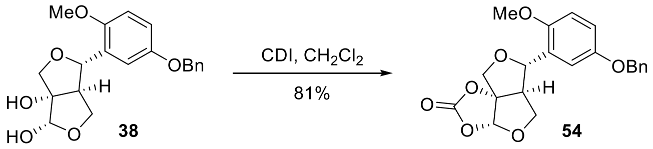

To a solution of diol **38** (1.13 g, 3.15 mmol, 1 equiv) in dichloromethane (15 mL) was added 1,1′-carbonyldiimidazole (614 mg, 3.78 mmol, 1.20 equiv) and the resulting solution was stirred for 4 h. The solution was concentrated under reduced pressure and the residue was purified by flash chromatography on silica gel (30% ethyl acetate in petroleum ether) which afforded carbonate **54** as a white solid (980 mg, 2.55 mmol, 81%).

TLC (20% ethyl acetate in cyclohexane): R_f = 0.25 (UV, KMnO4).

^{1}H NMR (400 MHz, $CDCl_3$): δ 7.45 – 7.30 (m, 5H), 7.16 (d, J = 3.0 Hz, 1H), 6.88 (dd, J = 8.9, 3.0 Hz, 1H), 6.81 (d, J = 8.9 Hz, 1H), 6.06 (s, 1H), 5.04 (s, 2H), 4.97 (d, J = 8.2 Hz, 1H), 4.49 – 4.43 (m, 2H), 4.24 (dd, J = 9.9, 5.6 Hz, 1H), 4.17 (d, J = 10.7 Hz, 1H), 3.79 (s, 3H), 2.95 – 2.89 (m, 1H) ppm.

^{13}C NMR (101 MHz, $CDCl_3$): δ 153.3, 152.2, 150.6, 137.2, 128.73, 128.70, 128.1, 127.7, 114.9, 113.1, 111.6, 108.1, 99.6, 81.2, 71.5, 70.9, 70.6, 57.3, 55.8 ppm.

IR (ATR, neat): $\tilde{\nu}$ = 2944 (*w*), 2903 (*w*), 2839 (*w*), 1812 (*s*), 1592 (*w*), 1499 (*m*), 1464 (*w*), 1434 (*w*), 1375 (*w*), 1319 (*w*), 1282 (*m*), 1220 (*m*), 1182 (*w*), 1109 (*m*), 1059 (*m*), 1005 (*m*), 873 (*w*), 766 (*m*), 736 (*w*) cm^{-1}.

HRMS (ESI): calcd. for $C_{21}H_{20}NaO_7$ $[M+Na]^+$: 407.1101; found: 407.1095.

Telescoped synthesis of carbonate 54

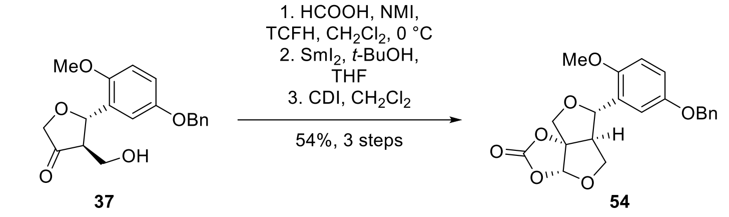

To a solution of alcohol **37** (7.20 g, 21.9 mmol, 1 equiv) in dichloromethane (130 mL) was added *N*-methylimidazole (8.74 mL, 110 mmol, 5.00 equiv) and formic acid (4.14 mL, 110 mmol, 5.00 equiv). The solution was cooled to 0 °C and *N,N,N',N'*-tetramethylchloroformamidinium hexafluorophosphate (10.5 g, 37.4 mmol, 1.70 equiv) was added portionwise over 5 min. After stirring at 0 °C for 5 min, the solution was added to a saturated aqueous ammonium chloride solution (200 mL). The organic solution was separated. The aqueous solution was extracted with dichloromethane (200 mL). The combined organic solutions were washed with water (200 mL) and with an aqueous saturated sodium chloride solution (200 mL). The washed solution was dried over sodium sulfate and filtered. The filtrate was concentrated under reduced pressure and the residue containing formate ester **SI-8** and tetramethylurea was directly used in the next step without further purification.

To a solution of samarium diiodide (0.100 M in tetrahydrofuran, 875 mL, 87.5 mmol, 4.00) was added *tert*-butanol (20.8 mL, 219 mmol, 10.0 equiv) followed by a solution of crude ester **SI-8** (assumed 21.9 mmol, 1 equiv) in tetrahydrofuran (50 mL) and the resulting solution was stirred for 40 min. The solution was then poured into an aqueous solution prepared from 200 mL of saturated ammonium chloride solution and 800 mL of saturated sodium potassium tartrate solution. The mixture was stirred for 30 min and then extracted with ethyl acetate (2 × 500 mL). The combined organic solutions were washed with a saturated aqueous sodium chloride solution (500 mL). The washed solution was dried over sodium sulfate and filtered. The filtrate was concentrated under reduced pressure and the residue containing diol **38** was used without further purification.

To a solution of diol **38** (assumed 21.9 mmol, 1 equiv) in dichloromethane (120 mL) was added 1,1′-carbonyldiimidazole (4.26 g, 26.3 mmol, 1.20 equiv) and the resulting solution was stirred for 15 min. The solution was concentrated under reduced pressure and the residue was purified by flash chromatography on silica gel (30% ethyl acetate in petroleum ether) which afforded carbonate **54** as a white solid (4.51 g, 11.7 mmol, 54% over 3 steps).

Synthesis of phenol 55

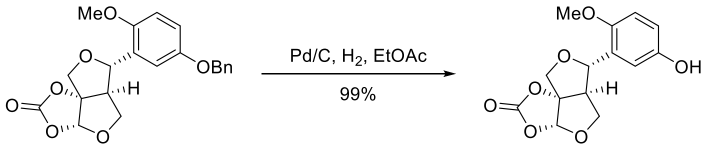

54 55

Carbonate **54** (8.99 g, 23.4 mmol, 1 equiv) was dissolved in 190 mL of ethyl acetate and split into 12 batches (16 mL each). To each batch was added palladium on carbon (104 mg, 10 wt% palladium, 97.5 μmol, 5 mol%), the stirred suspensions were sparged with hydrogen gas for 30 min and then stirred under the hydrogen atmosphere. After 2 h, the mixtures were sparged with argon for 1 min and then combined. The resulting mixture was filtered through celite and the filtrate was concentrated under reduced pressure which afforded phenol **55** as a beige solid (6.80 g, 23.1 mmol, 99%) that was directly used without further purification.

Characterization data for phenol **55**:

TLC (40% ethyl acetate in cyclohexane): R_f = 0.37 (weak UV, $KMnO_4$).

^{1}H NMR (400 MHz, $CDCl_3$): δ 7.01 – 6.99 (m, 2H), 6.78 – 6.75 (m, 2H), 6.06 (s, 1H), 4.95 (d, J = 8.2 Hz, 1H), 4.85 (s, 1H), 4.51 – 4.43 (m, 2H), 4.24 (dd, J = 10.0, 5.6 Hz, 1H), 4.17 (d, J = 10.7 Hz, 1H), 3.78 (s, 3H), 2.95 – 2.90 (m, 1H) ppm.

^{13}C NMR (101 MHz, $CDCl_3$): δ 152.2, 150.4, 149.9, 128.8, 115.4, 113.1, 111.9, 108.1, 99.7, 81.0, 71.5, 70.5, 57.4, 55.9 ppm.

IR (ATR, neat): $\tilde{\nu}$ = 3413 (*br, m*), 2964 (*w*), 2901 (*w*), 2839 (*w*), 1807 (*s*), 1502 (*m*), 1462 (*m*), 1439 (*w*), 1356 (*m*), 1325 (*w*), 1285 (*m*), 1220 (*s*), 1152 (*w*), 1109 (*m*), 1058 (*m*), 1007 (*m*), 912 (*w*), 871 (*m*), 768 (*w*), 730 (*m*) cm^{-1}.

HRMS (ESI): calcd. for $C_{14}H_{14}NaO_7$ $[M+Na]^+$: 317.0632; found: 317.0627.

Characterization data for alcohol **56**:

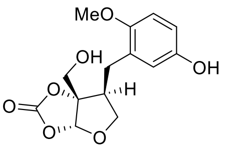

56

Note: Alcohol **56** was isolated when 20 mol% palladium on carbon was used in the hydrogenation reaction.

TLC (40% ethyl acetate in cyclohexane): R_f = 0.25 (weak UV, $KMnO_4$).

^{1}H NMR (400 MHz, CD_3OD): *δ* 6.79 (d, *J* = 8.8 Hz, 1H), 6.64 (dd, *J* = 8.7, 3.0 Hz, 1H), 6.59 (d, *J* = 3.0 Hz, 1H), 6.06 (s, 1H), 4.02 (d, *J* = 12.6 Hz, 1H), 3.96 – 3.90 (m, 2H), 3.85 (dd, *J* = 9.7, 0.8 Hz, 1H), 3.76 (s, 3H), 2.82 – 2.74 (m, 2H), 2.37 (dd, *J* = 14.0, 13.0 Hz, 1H) ppm.

^{13}C NMR (101 MHz, CD_3OD): *δ* 156.1, 152.3, 152.1, 128.9, 118.8, 115.0, 113.0, 107.1, 96.7, 71.8, 61.4, 56.3, 46.7, 28.8 ppm.

IR (ATR, neat): $\tilde{v}$ = 3398 (*br, m*), 3383 (*w*), 2931 (*w*), 2837 (*w*), 1797 (*s*), 1707 (*w*), 1503 (*m*), 1461 (*w*), 1439 (*w*), 1361 (*w*), 1304 (*w*), 1287 (*w*), 1219 (*s*), 1153 (*w*), 1118 (*w*), 1087 (*w*), 1056 (*w*), 1032 (*w*), 1005 (*s*), 965 (*w*), 936 (*w*), 899 (*w*), 810 (*w*), 772 (*w*), 743 (*w*), 722 (*w*) cm^{-1}.

HRMS (ESI): calcd. for $C_{14}H_{16}NaO_7$ $[M+Na]^+$: 319.0788; found: 319.0783.

Synthesis of *ortho*-quinone 14

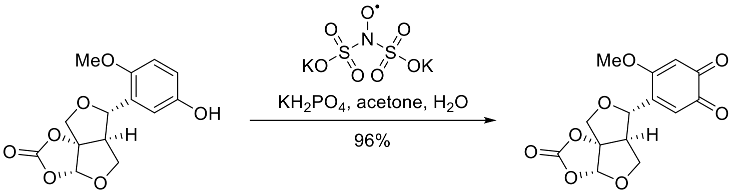

55 14

A mixture of potassium dihydrogen phosphate (9.43 g, 69.3 mmol, 6.00 equiv), dipotassium nitrosodisulfonate (15.6 g, 57.8 mmol, 5.00 equiv), phenol **55** (3.40 g, 11.6 mmol, 1 equiv), acetone (150 mL) and water (75 mL) was stirred for 4 h. Ethyl acetate (200 mL) was added and the aqueous phase was separated. The aqueous phase was extracted with ethyl acetate (50 mL). The organic solutions were combined and concentrated under reduced pressure. Water (100 mL) was added and the mixture was extracted with ethyl acetate (3 × 200 mL). The combined organic solutions were washed with a saturated aqueous sodium chloride solution (300 mL). The washed solution was dried over sodium sulfate and filtered. The filtrate was concentrated under reduced pressure which afforded *ortho*-quinone **14** as a dark red solid (3.41 g, 11.1 mmol, 96%) that was used without further purification.

TLC (60% ethyl acetate in cyclohexane): R_f = 0.25 (UV, $KMnO_4$).

^{1}H NMR (400 MHz, $CDCl_3$): *δ* 6.62 (d, *J* = 1.8 Hz, 1H), 6.07 (s, 1H), 5.84 (s, 1H), 4.76 (dd, *J* = 7.2, 1.8 Hz, 1H), 4.39 – 4.32 (m, 3H), 4.27 (d, *J* = 10.5 Hz, 1H), 3,89 (s, 3H), 3.01 – 2.95 (m, 1H) ppm.

^{13}C NMR (101 MHz, $CDCl_3$): *δ* 179.7, 178.1, 166.6, 151.5, 149.60, 125.3, 107.9, 104.1, 97.9, 80.7, 71.2, 70.7, 57.4, 55.5 ppm.

IR (ATR, neat): $\tilde{\nu}$ = 2943 (*w*), 2917 (*w*), 2854 (*w*), 1813 (*s*), 1645 (*m*), 1579 (*m*), 1458 (*w*), 1384 (*w*), 1355 (*m*), 1289 (*w*), 1255 (*m*), 1240 (*m*), 1205 (*w*), 1101 (*m*), 1063 (*m*), 995 (*m*), 914 (*w*), 831 (*w*), 668 (*m*) cm^{-1}.

Synthesis of styrenes:

General procedure A for synthesis of styrenes SI-10 from aryl aldehydes SI-9

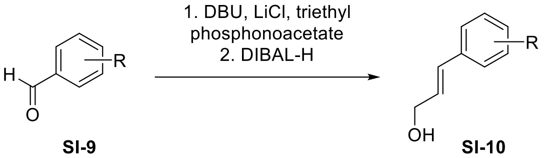

SI-9 **SI-10**

To a solution of lithium chloride (1.2 equiv) in tetrahydrofuran (0.35 M) was added triethyl phosphonoacetate (1.2 equiv) followed by 1,8-diazabicyclo[5.4.0]undec-7-ene (1.2 equiv). The resulting suspension was cooled to 0 °C and aldehyde **SI-9** (1 equiv) was added. The reaction mixture was allowed to warm to 22 °C and was stirred for 1 h. A saturated aqueous solution of ammonium chloride (5 mL per mmol of aldehyde) was added and the mixture was extracted with ethyl acetate (5 mL per mmol of aldehyde). The organic solution was dried over sodium sulfate and filtered. The filtrate was concentrated under reduced pressure which afforded the crude ester that was used in the following step without further purification.

To a solution of the crude ester (assumed 1 equiv) in dichloromethane (0.25 M) cooled to –78 °C was added a solution of diisobutylaluminium hydride (1.0 M in hexane, 2.5 equiv). The resulting solution was allowed to warm to 22 °C and stirred for 30 min. The solution was poured into a saturated aqueous solution of sodium potassium tartrate (20 mL per mmol of ester) and stirred for 2 h. The mixture was extracted with ethyl acetate (2 × 10 mL per mmol of ester), the organic solution was dried over sodium sulfate and filtered. The filtrate was concentrated under reduced pressure which afforded the crude ester **SI-10** that was either used in the following step without further purification, or purified by flash chromatography.

4-Chlorostyrene SI-10a

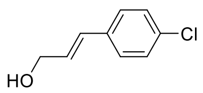

General procedure A was followed using 4-chlorobenzaldehyde (1.00 g, 7.11 mmol). Styrene **SI-10a** was obtained as a white solid (1.03 g, 6.11 mmol, 86% over 2 steps) and was used without further purification.

^{1}H NMR (400 MHz, $CDCl_3$): δ 7.34 – 7.26 (m, 4H), 6.58 (dt, J = 16.0, 1.6 Hz, 1H), 6.34 (dt, J = 15.9, 5.6 Hz, 1H), 4.32 (dd, J = 5.6, 1.6 Hz, 2H) ppm.

The analytical data matched those reported previously.[11]

4-Methoxystyrene SI-10b

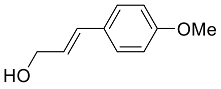

General procedure A was followed using 4-methoxybenzaldehyde (200 mg, 1.47 mmol). Styrene **SI-10b** was obtained as a white solid (194 mg, 1.18 mmol, 80% over 2 steps) and was used without further purification.

^{1}H NMR (400 MHz, $CDCl_3$): δ 7.35 – 7.30 (m, 2H), 6.88 – 6.83 (m, 2H), 6.56 (dt, J = 15.8, 1.6 Hz, 1H), 6.24 (dt, J = 15.8, 6.0 Hz, 1H), 4.30 (dt, J = 5.7, 2.0 Hz, 2H), 3.81 (s, 3H) ppm.

The analytical data matched those reported previously.[12]

4-Nitrostyrene SI-10c

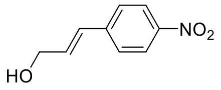

General procedure A was followed using 4-nitrobenzaldehyde (200 mg, 1.32 mmol). Styrene **SI-10c** was purified by flash chromatography on silica gel (30% ethyl acetate in petroleum ether) and was obtained as an orange solid (118 mg, 659 µmol, 50% over 2 steps).

^{1}H NMR (400 MHz, $CDCl_3$): δ 8.22 – 8.16 (m, 2H), 7.55 – 7.49 (m, 2H), 6.72 (dt, J = 15.9, 1.8 Hz, 1H), 6.54 (dt, J = 15.9, 5.0 Hz, 1H), 4.41 (dd, J = 5.2, 1.6 Hz, 2H) ppm.

The analytical data matched those reported previously.[13]

4-Fluorostyrene SI-10d

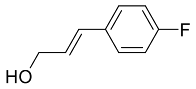

General procedure A was followed using 4-fluorobenzaldehyde (200 mg, 1.61 mmol). Styrene **SI-10d** was obtained as a white solid (219 mg, 1.44 mmol, 89% over 2 steps) and was used without further purification.

^{1}H NMR (400 MHz, $CDCl_3$): δ 7.39 – 7.32 (m, 2H), 7.05 – 6.98 (m, 2H), 6.59 (dt, J = 15.9, 1.7 Hz, 1H), 6.33 – 6.24 (m, 1H), 4.32 (dd, J = 5.7, 1.6 Hz, 2H) ppm.

The analytical data matched those reported previously.[11]

Synthesis of aldehyde SI-9a

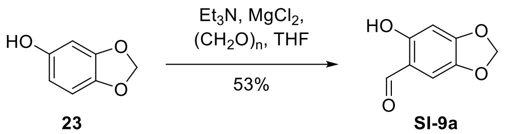

Aldehyde **SI-9a** was prepared according to a procedure reported in the literature.[14] A mixture of sesamol (**23**) (1.40 g, 10.1 mmol, 1 equiv), magnesium chloride (1.50 g, 15.8 mmol, 1.55 equiv), triethylamine (5.00 mL, 35.9 mmol, 3.54 equiv) and parafomaldehyde (1.50 g, 50.0 mmol, 4.93 equiv) in tetrahydrofuran (50 mL) was refluxed for 10 h. The reaction mixture was allowed to cool to 22 °C and an aqueous hydrogen chloride solution was added (2.0 M, 2.0 mL). The mixture was extracted with ethyl acetate (3 × 50 mL). The combined organic solutions were washed with a saturated aqueous sodium chloride solution (50 mL). The washed solution was dried over sodium sulfate, filtered, and concentrated under reduced pressure. The residue was purified by flash chromatography on silica gel (20% ethyl acetate in petroleum ether) which afforded aldehyde **SI-9a** as a light-yellow solid (900 mg, 5.42 mmol, 53%).

^{1}H NMR (400 MHz, $CDCl_3$): δ 11.78 (s, 1H), 9.62 (s, 1H), 6.85 (s, 1H), 6.46 (s, 1H), 6.01 (s, 2H) ppm.

The analytical data matched those reported previously.[14]

Synthesis of styrene SI-10e

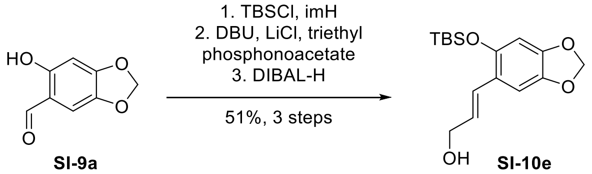

To a solution of aldehyde **SI-9a** (900 mg, 5.42 mmol, 1 equiv) in dichloromethane (20 mL) was added imidazole (369 mg, 5.42 mmol, 1.00 equiv) and the solution was cooled to −78 °C. *Tert*-butyldimethylsilyl trifluoromethanesulfonate (1.24 mL, 5.42 mmol, 1.00 equiv) was added and the resulting solution was allowed to warm to 22 °C. The solution was washed with water (20 mL) and with a saturated aqueous sodium chloride solution (20 mL). The washed organic solution was dried over sodium sulfate and filtered.

The filtrate was concentrated under reduced pressure which afforded crude aldehyde that was used in the next step without further purification.

General procedure A was followed using crude 4,5-methylenedioxy-2-(*tert*-butyldimethylsilyloxy)-benzaldehyde (assumed 5.42 mmol). Styrene **SI-10e** was purified by flash chromatography on silica gel (10% ethyl acetate in petroleum ether) and was obtained as a yellow solid (848 mg, 2.75 mmol, 51% over 3 steps).

TLC (20% ethyl acetate in cyclohexane): R_f = 0.51 (UV, $KMnO_4$).

^{1}H NMR (500 MHz, $CDCl_3$): δ 6.92 (s, 1H), 6.85 (d, J = 15.9 Hz, 1H), 6.35 (s, 1H), 6.11 (dt, J = 15.9, 6.1 Hz, 1H), 5.90 (s, 2H), 4.27 (dd, J = 6.0, 1.5 Hz, 2H), 1.01 (s, 9H), 0.18 (s, 6H) ppm.

^{13}C NMR (126 MHz, $CDCl_3$): δ 148.1, 147.8, 142.4, 126.8, 126.1, 120.6, 104.9, 101.8, 101.3, 64.5, 25.9, 18.1, –4.1 ppm.

IR (ATR, neat): $\tilde{\nu}$ = 3329 (*br, w*), 2955 (*w*), 2929 (*w*), 2886 (*w*), 2858 (*w*), 1623 (*w*), 1503 (*w*), 1478 (*s*), 1431 (*m*), 1391 (*w*), 1363 (*w*), 1318 (*w*), 1280 (*w*), 1253 (*m*), 1174 (*s*), 1073 (*w*), 1038 (*m*), 1007 (*w*), 969 (*w*), 938 (*w*), 897 (*m*), 837 (*s*), 780 (*m*) cm^{-1}.

HRMS (ESI): calcd. for $C_{16}H_{24}NaO_4Si$ $[M+Na]^+$: 331.1336; found: 331.1308.

Synthesis of styrene 13b

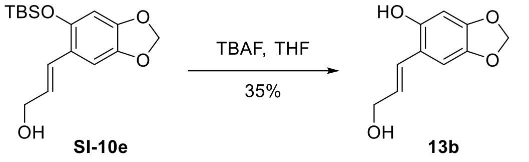

To a solution of styrene **SI-10e** (600 mg, 1.95 mmol, 1 equiv) in tetrahydrofuran (5 mL) was added a solution of tetrabutylammonium fluoride (1.00 M in tetrahydrofuran, 2.14 mL, 2.14 mmol, 1.10 equiv). After 15 min, a saturated aqueous ammonium chloride solution (5 mL) was added. The mixture was extracted with ethyl acetate (2 × 10 mL). The organic solutions were combined, dried over sodium sulfate and filtered. The filtrate was concentrated under reduced pressure and the residue was purified by flash chromatography on silica gel (50% ethyl acetate in petroleum ether) which afforded styrene **13b** as a white solid (131 mg, 675 µmol, 35%).

TLC (50% ethyl acetate in cyclohexane): R_f = 0.34 (UV, $KMnO_4$).

^{1}H NMR (500 MHz, acetone-d_6): δ 6.94 (s, 1H), 6.85 (dd, J = 16.1, 1.9 Hz, 1H), 6.45 (s, 1H), 6.19 (dt, J = 16.1, 5.7 Hz, 1H), 5.89 (s, 2H), 4.18 (d, J = 5.7 Hz, 2H) ppm.

^{13}C NMR (126 MHz, acetone-d_6): δ 150.3, 148.3, 142.1, 128.1, 125.1, 117.4, 105.8, 101.8, 98.5, 63.7 ppm.

IR (ATR, neat): $\tilde{\nu}$ = 3273 (*br, m*), 2892 (*w*), 1625 (*w*), 1502 (*w*), 1482 (*m*), 1443 (*s*), 1411 (*w*), 1306 (*w*), 1279 (*w*), 1249 (*w*), 1172 (*s*), 1086 (*w*), 1063 (*w*), 1037 (*s*), 969 (*m*), 934 (*w*), 868 (*m*), 832 (*w*), 764 (*w*) cm^{-1}.

HRMS (ESI): calcd. for $C_{10}H_{10}NaO_4$ $[M+Na]^+$: 217.0471; found: 217.0453.

Synthesis of styrene 13a

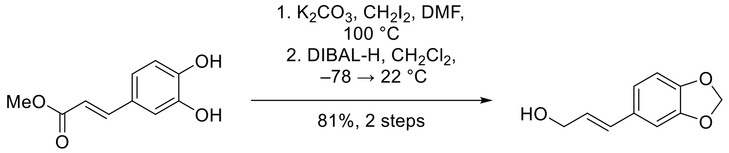

SI-11 **13a**

A suspension of ester **SI-11** (2.00 g, 10.3 mmol, 1 equiv), potassium carbonate (4.27 g, 30.9 mmol, 3.00 equiv) and diiodomethane (1.25 mL, 15.4 mmol, 1.50 equiv) in *N,N*-dimethylformamide (50 mL) was stirred and heated to 100 °C. After 6 h, the mixture was allowed to cool to 22 °C. Ethyl acetate (100 mL) was added and the mixture was washed with a saturated aqueous solution of lithium chloride (3 × 50 mL). The washed organic solution was dried over sodium sulfate and filtered. The filtrate was concentrated under reduced pressure which afforded crude benzodioxole that was used in the next step without further purification.

To a solution of the crude benzodioxole (assumed 10.3 mmol, 1 equiv) in dichloromethane (25 mL) cooled to –78 °C was added a solution of diisobutylaluminium hydride (1.00 M in hexanes, 25.8 mL, 25.8 mmol, 2.50 equiv) and the resulting solution was allowed to warm to 22 °C. The solution was poured into a saturated aqueous solution of sodium potassium tartrate (200 mL) and stirred for 2 h. The mixture was extracted with ethyl acetate (2 × 100 mL). The organic solution was dried over sodium sulfate and filtered. The filtrate was concentrated under reduced pressure and the residue was purified by flash chromatography on silica gel (20% ethyl acetate in petroleum ether) which afforded styrene **13a** as a white solid (1.48 g, 8.31 mmol, 81% over 2 steps).

^{1}H NMR (400 MHz, $CDCl_3$): δ 6.93 (d, J = 1.7 Hz, 1H), 6.82 (dd, J = 8.1, 1.7 Hz, 1H), 6.76 (d, J = 8.0 Hz, 1H), 6.53 (dt, J = 15.8, 1.5 Hz, 1H), 6.20 (dt, J = 15.8, 5.9 Hz, 1H), 5.96 (s, 2H), 4.29 (td, J = 5.9, 1.5 Hz, 2H), 1.39 (t, J = 5.9 Hz, 1H) ppm.

The analytical data matched those reported previously.[15]

Synthesis of styrene 13e

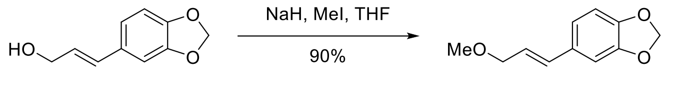

To a solution of styrene **13a** (500 mg, 2.81 mmol, 1 equiv) in tetrahydrofuran (10 mL) was added sodium hydride (60 wt% in mineral oil, 258 mg, 6.45 mmol, 2.30 equiv) and the mixture was stirred for 1 h. Methyl iodide (526 µL, 8.42 mmol, 3.00 equiv) was added and the resulting mixture was stirred for 20 h. A saturated aqueous ammonium chloride solution (5 mL) was added and the mixture was extracted with dichloromethane (2 × 10 mL). The organic solution was dried over sodium sulfate and filtered. The filtrate was concentrated under reduced pressure and the residue was purified by flash chromatography on silica gel (10% ethyl acetate in petroleum ether) which afforded styrene **13e** as a colorless oil (487 mg, 2.53 mmol, 90%).

^{1}H NMR (400 MHz, $CDCl_3$): δ 6.93 (d, J = 1.7 Hz, 1H), 6.82 (dd, J = 8.1, 1.7 Hz, 1H), 6.75 (d, J = 8.0 Hz, 1H), 6.52 (dt, J = 15.8, 1.6 Hz, 1H), 6.11 (dt, J = 15.8, 6.1 Hz, 1H), 5.95 (s, 2H), 4.06 (dd, J = 6.1, 1.4 Hz, 2H), 3.38 (s, 3H) ppm.

The analytical data matched those reported previously.[16]

Synthesis of styrene SI-12

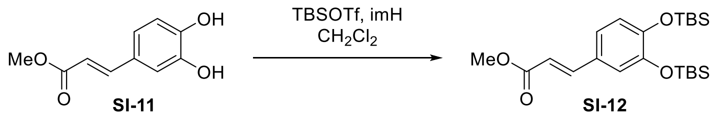

To a suspension of ester **SI-11** (3.00 g, 15.4 mmol, 1 equiv) in dichloromethane (100 mL) was added imidazole (2.42 g, 35.5 mmol, 2.30 equiv) followed by *tert*-butyldimethylsilyl trifluoromethanesulfonate (7.81 mL, 34.0 mmol, 2.20 equiv). After 6 h, the mixture was washed with water (2 × 100 mL) and with a saturated aqueous sodium chloride solution (100 mL). The washed organic solution was dried over sodium sulfate and filtered. The filtrate was concentrated under reduced pressure and the residue containing crude ester **SI-12** was used in the next step without further purification.

Note: An analytical sample of crude ester **SI-12** was purified by flash chromatography on silica gel (5% ethyl acetate in petroleum ether).

^{1}H NMR (400 MHz, $CDCl_3$): δ 7.57 (d, J = 15.9 Hz, 1H), 7.04 – 6.97 (m, 2H), 6.82 (d, J = 8.8 Hz, 1H), 6.24 (d, J = 15.9 Hz, 1H), 3.79 (s, 3H), 1.05 – 0.95 (m, 18H), 0.27 – 0.15 (m, 12H) ppm.

The analytical data matched those reported previously.[17]

Synthesis of styrene SI-13

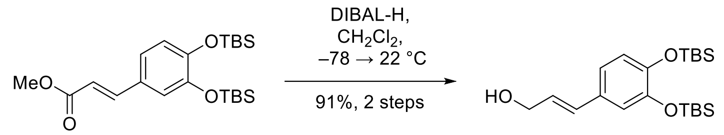

To a solution of crude ester **SI-12** (assumed 15.4 mmol, 1 equiv) in dichloromethane (50 mL) cooled to −78 °C was added a solution of diisobutylaluminium hydride (1.00 M in hexane, 38.6 mL, 38.6 mmol, 2.50 equiv) and the resulting solution was allowed to warm to 22 °C. After 30 min, the solution was cooled to 0 °C and ethyl acetate (10 mL) was added over 5 min. The solution was poured into a saturated solution of sodium potassium tartrate (400 mL) and the resulting mixture was vigorously stirred for 2 h. The mixture was extracted with ethyl acetate (2 × 200 mL). The organic solution was washed with a saturated aqueous sodium chloride solution (200 mL). The washed organic solution was dried over sodium sulfate and filtered. The filtrate was concentrated under reduced pressure and the residue was purified by flash chromatography on silica gel (20% ethyl acetate in petroleum ether) which afforded alcohol **SI-13** as a pale-yellow solid (5.54 g, 14.0 mmol, 91% over 2 steps).

^{1}H NMR (400 MHz, $CDCl_3$): *δ* 6.88 (d, *J* = 2.1 Hz, 1H), 6.85 (dd, *J* = 8.2, 2.1 Hz, 1H), 6.77 (d, *J* = 8.2 Hz, 1H), 6.48 (dt, *J* = 15.8, 1.4 Hz, 1H), 6.18 (dt, *J* = 15.8, 6.0 Hz, 1H), 4.29 (t, *J* = 4.6 Hz, 2H), 1.01 – 0.96 (m, 18H), 0.22 – 0.18 (m, 12H) ppm.

The analytical data matched those reported previously.[18]

Synthesis of styrene SI-14

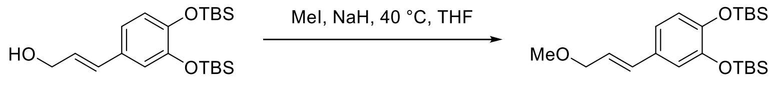

To a solution of alcohol **SI-13** (2.00 g, 5.07 mmol, 1 equiv) and methyl iodide (3.21 mL, 50.7 mmol, 10.0 equiv) in tetrahydrofuran (40 mL) heated to 40 °C was added sodium hydride (1.01 g, 60 wt% in mineral oil, 25.3 mmol, 5.00 equiv) in one portion and the resulting solution was stirred at 40 °C for 1 h. A saturated aqueous ammonium chloride solution was added slowly and the mixture was allowed to cool to 22 °C and stirred for 10 min. The phases were separated and the organic phase was dried over sodium sulfate and filtered. The filtrate was concentrated under reduced pressure which afforded crude styrene **SI-14** that was used without further purification.

Note: An analytical sample of styrene **SI-14** was purified by flash chromatography on silica gel (5% ethyl acetate in petroleum ether).

TLC (5% ethyl acetate in cyclohexane): R_f = 0.50 (UV, $KMnO_4$).

^{1}H NMR (400 MHz, $CDCl_3$): δ 6.88 (d, *J* = 2.2 Hz, 1H), 6.84 (dd, *J* = 8.2, 2.2 Hz, 1H), 6.76 (d, *J* = 8.1 Hz, 1H), 6.47 (dt, *J* = 15.9, 1.4 Hz, 1H), 6.08 (dt, *J* = 15.9, 6.2 Hz, 1H), 4.06 (dd, *J* = 6.2, 1.4 Hz, 2H), 3.38 (s, 3H), 0.98 (d, *J* = 4.2 Hz, 18H), 0.20 (d, *J* = 1.5 Hz, 12H) ppm.

^{13}C NMR (101 MHz, $CDCl_3$): δ 147.0, 147.0, 132.6, 130.5, 123.9, 121.1, 120.1, 119.2, 73.4, 58.0, 26.1, 26.1, 18.6, 18.6, –3.9 ppm.

IR (ATR, neat): $\tilde{\nu}$ = 2955 (*m*), 2930 (*m*), 2887 (*w*), 2858 (*m*), 1569 (*w*), 1510 (*s*), 1472 (*m*), 1464 (*w*), 1302 (*m*), 1253 (*m*), 1166 (*w*), 1122 (*m*), 997 (*w*), 965 (*w*) 905 (*m*), 930 (*s*), 781 (*s*) cm^{-1}.

HRMS (ESI): calcd. for $C_{22}H_{40}NaO_3Si_2$ [M+Na]$^+$: 431.2408; found: 431.2393.

Synthesis of styrene 13d

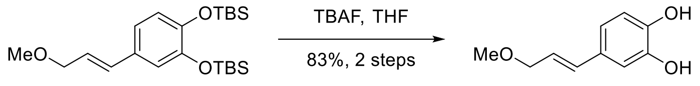

SI-14 **13d**

To a solution of crude styrene **SI-14** (assumed 5.07 mmol, 1 equiv) in tetrahydrofuran (10 mL) was added acetic acid (700 µL, 12.2 mmol, 2.41 equiv) followed by a solution of tetrabutylammonium fluoride (1.00 M in tetrahydrofuran, 10.6 mL, 10.6 mmol, 2.10 equiv) and the resulting solution was stirred for 2 h. Water (100 mL) was added and the mixture was extracted with ethyl acetate (2 × 100 mL). The organic solutions were combined and concentrated under reduced pressure. The residue was purified by flash chromatography on silica gel (40% ethyl acetate in petroleum ether) which afforded styrene **13d** as a yellow solid (760 mg, 4.22 mmol, 83% over 2 steps).

TLC (30% ethyl acetate in cyclohexane): R_f = 0.26 (UV, $KMnO_4$).

^{1}H NMR (400 MHz, $CDCl_3$): δ 6.95 – 6.92 (m, 1H), 6.82 – 6.77 (m, 2H), 6.48 (dt, *J* = 15.9, 1.2 Hz, 1H), 6.09 (dt, *J* = 15.9, 6.3 Hz, 1H), 5.81 (br s, 2H), 4.10 (dd, *J* = 6.4, 1.3 Hz, 2H), 3.41 (s, 3H) ppm.

^{13}C NMR (101 MHz, $CDCl_3$): δ 144.1, 143.9, 133.3, 130.0, 123.2, 120.4, 115.4, 112.8, 73.5, 57.9 ppm.

IR (ATR, neat): $\tilde{\nu}$ = 3260 (*br, s*), 2936 (*w*), 2830 (*w*), 1602 (*m*), 1518 (*m*), 1442 (*m*), 1372 (*m*), 1282 (*s*), 1193 (*m*), 1157 (*w*), 1106 (*m*), 1068 (*m*), 967 (*m*), 870 (*w*), 797 (*w*) cm^{-1}.

HRMS (ESI): calcd. for $C_{10}H_{12}NaO_3$ [M+Na]$^+$: 203.0679; found: 203.0671.

Synthesis of styrene SI-15

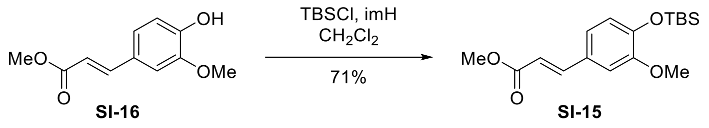

To a suspension of styrene **SI-16** (3.98 g, 19.1 mmol, 1 equiv) in dichloromethane (50 mL) was added imidazole (1.95 g, 28.7 mmol, 1.50 equiv) followed by *tert*-butyldimethylsilyl chloride (4.32 g, 28.7 mmol, 1.50 equiv). After 6 h, the mixture was washed with water (2 × 50 mL) and with a saturated aqueous sodium chloride solution (50 mL). The washed organic solution was dried over sodium sulfate and filtered. The filtrate was concentrated under reduced pressure and the residue was purified by flash chromatography on silica gel (5% ethyl acetate in petroleum ether) which afforded styrene **SI-15** as colorless oil (4.40 g, 13.6 mmol, 71%).

^{1}H NMR (400 MHz, $CDCl_3$): δ 7.62 (d, *J* = 15.9 Hz, 1H), 7.04 – 6.98 (m, 2H), 6.84 (d, *J* = 8.7 Hz, 1H), 6.30 (d, *J* = 15.9 Hz, 1H), 3.83 (s, 3H), 3.79 (s, 3H), 0.99 (s, 9H), 0.17 (s, 6H) ppm.

The analytical data matched those reported previously.[19]

Synthesis of styrene SI-17

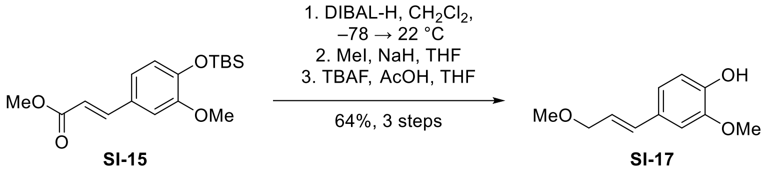

To a solution of styrene **SI-15** (4.39 g, 13.6 mmol, 1 equiv) in dichloromethane (20 mL) cooled to –78 °C was added a solution of diisobutylaluminium hydride (1.00 M in hexane, 34.0 mL, 34.0 mmol, 2.50 equiv) and the resulting solution was allowed to warm to 22 °C. After 30 min, the solution was cooled to 0 °C and ethyl acetate (10 mL) was added over 5 min. The solution was poured into a saturated solution of sodium potassium tartrate (400 mL) and the resulting mixture was vigorously stirred for 2 h. The mixture was extracted with ethyl acetate (2 × 200 mL). The organic solution was washed with a saturated aqueous sodium chloride solution (200 mL). The washed organic solution was dried over sodium sulfate and filtered. The filtrate was concentrated under reduced pressure and the residue was used in the following step without further purification.

To a solution of the crude alcohol (assumed 13.6 mmol, 1 equiv) and methyl iodide (4.25 mL, 68.0 mmol, 5.0 equiv) in tetrahydrofuran (20 mL) heated to 40 °C was added sodium hydride (1.09 g, 60 wt% in mineral oil, 27.2 mmol, 2.00 equiv) in one portion and the resulting solution was stirred at 40 °C for 1 h. A saturated aqueous ammonium chloride solution (20 mL) was added slowly and the mixture was allowed to cool to 22

°C and stirred for 10 min. The organic phase was separated, dried over sodium sulfate and filtered. The filtrate was concentrated under reduced pressure which afforded crude methyl ether that was used in the next step without further purification.

To a solution of the crude methyl ether (assumed 13.6 mmol, 1 equiv) in tetrahydrofuran (20 mL) was added acetic acid (973 µL, 17.0 mmol, 1.25 equiv) followed by a solution of tetrabutylammonium fluoride (1.00 M in tetrahydrofuran, 16.3 mL, 16.3 mmol, 1.20 equiv) and the resulting solution was stirred for 2 h. Water (50 mL) was added and the mixture was extracted with ethyl acetate (2 × 50 mL). The organic solutions were combined and concentrated under reduced pressure. The residue was purified by flash chromatography on silica gel (20% ethyl acetate in petroleum ether) which afforded styrene **SI-17** as a colorless solid (1.70 g, 8.75 mmol, 64% over 3 steps).

TLC (20% ethyl acetate in cyclohexane): R_f = 0.31 (UV, $KMnO_4$).

^{1}H NMR (400 MHz, $CDCl_3$): δ 6.99 – 6.81 (m, 3H), 6.53 (dt, J = 15.9, 1.5 Hz, 1H), 6.13 (dt, J = 15.8, 6.2 Hz, 1H), 5.66 (s, 1H), 4.07 (dd, J = 6.2, 1.4 Hz, 2H), 3.90 (s, 3H), 3.38 (s, 3H) ppm.

^{13}C NMR (101 MHz, $CDCl_3$): δ 146.7, 145.7, 132.8, 129.4, 123.7, 120.5, 114.5, 108.4, 73.4, 58.0, 56.0 ppm.

IR (ATR, neat): $\tilde{\nu}$ = 3365 (*w*), 2935 (*w*), 2825 (*w*), 1595 (*m*), 1511 (*s*), 1463 (*w*), 1450 (*w*), 1427 (*m*), 1375 (*w*), 1269 (*s*), 1234 (*w*), 1204 (*w*), 1155 (*m*), 1108 (*m*), 1081 (*w*), 1031 (*m*), 965 (*s*), 930 (*w*), 902 (*w*), 857 (*m*), 826 (*w*), 796 (*m*), 756 (*w*), 735 (*w*) cm^{-1}.

HRMS (ESI): calcd. for $C_{11}H_{13}O_3$ $[M]^-$: 193.0870; found: 193.0863.

Model studies of the formal [4+2] cycloaddition reaction:

Synthesis of quinone 25b

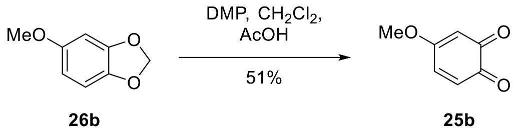

To a solution of benzodioxole **26b** (100 mg, 657 µmol, 1 equiv) in dichloromethane (5 mL) was added Dess–Martin peroiodinane (1.12 g, 2.63 mmol, 4.00 equiv) followed by acetic acid (215 µL, 3.94 mmol, 6.00 equiv) and the resulting suspension was stirred for 20 h. The mixture was diluted with ethyl acetate (10 mL) and washed with a saturated aqueous sodium bicarbonate solution (3 × 10 mL). The organic solution was dried over sodium sulfate and filtered. The filtrate was concentrated and the residue was purified by flash chromatograhphy on silica gel (40% ethyl acetate in petroleum ether) which afforded quinone **25b** as a red solid (46.3 mg, 335 µmol, 51%).

^{1}H NMR (400 MHz, $CDCl_3$): δ 6.88 (dd, J = 10.4, 3.0 Hz, 1H), 6.42 (d, J = 10.4 Hz, 1H), 5.79 (d, J = 3.1 Hz, 1H), 3.86 (s, 3H) ppm.

The analytical data matched those reported previously.[20]

Synthesis of benzodioxole 26c

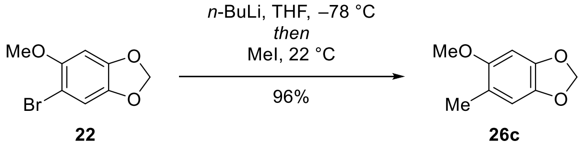

To a solution of aryl bromide **22** (620 mg, 2.68 mmol, 1 equiv) in tetrahydrofuran (5 mL) cooled to –78 °C was added dropwise a solution of *n*-butyllithium (1.60 M in hexanes, 2.01 mL, 3.22 mmol, 1.20 equiv) and the resulting solution was stirred for 5 min. Methyl iodide (252 µL, 4.03 mmol, 1.50 equiv) was added dropwise at –78 °C and the solution was allowed to warm to 22 °C. After 1 h, a saturated aqueous ammonium chloride solution (10 mL) was added and the mixture was extracted with ethyl acetate (2 × 10 mL). The combined organic solutions were washed with a saturated aqueous sodium chloride solution. The washed organic solution was dried over sodium sulfate and filtered. The filtrate was concentrated under reduced pressure and the residue was purified by flash chromatography on silica gel (5% diethyl ether in petroleum ether) which afforded benzodioxole **26c** as a colorless oil (427 mg, 2.57 mmol, 96%).

^{1}H NMR (400 MHz, $CDCl_3$): δ 6.64 (d, J = 0.8 Hz, 1H), 6.50 (s, 1H), 5.88 (s, 2H), 3.77 (s, 3H), 2.14 (s, 3H) ppm.

The analytical data matched those reported previously.[21]

Synthesis of quinone 25c

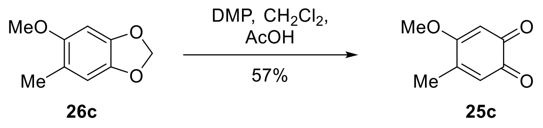

To a solution of benzodioxole **26c** (80.0 mg, 481 µmol, 1 equiv) in dichloromethane (5 mL) was added Dess-Martin peroiodinane (817 g, 1.93 mmol, 4.00 equiv) followed by acetic acid (165 µL, 2.89 mmol, 6.00 equiv) and the resulting suspension was stirred for 20 h. The mixture was diluted with ethyl acetate (10 mL) and washed with a saturated aqueous sodium bicarbonate solution (3 × 10 mL). The organic solution was dried over sodium sulfate and filtered. The filtrate was concentrated and the residue was purified by flash chromatograhphy on silica gel (30% ethyl acetate in petroleum ether) which afforded quinone **25c** as a red solid (41.9 mg, 275 µmol, 57%).

^{1}H NMR (400 MHz, $CDCl_3$): δ 6.26 (q, J = 1.7 Hz, 1H), 5.75 (s, 1H), 3.87 (s, 3H), 2.13 (d, J = 1.6 Hz, 3H) ppm.

The analytical data matched those reported previously.[22]

Synthesis of benzodioxole 26d

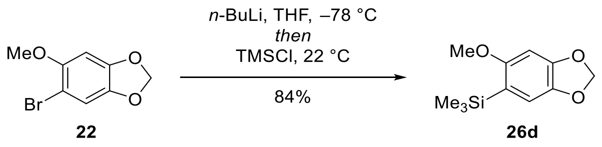

To a solution of aryl bromide **22** (8.00 g, 34.6 mmol, 1 equiv) in tetrahydrofuran (80 mL) cooled to –78 °C was added dropwise a solution of *n*-butyllithium (1.6M in hexanes, 24.9 mL, 39.8 mmol, 1.15 equiv) and the resulting solution was stirred for 5 min. Chlorotrimethylsilane (5.49 mL, 43.3 mmol, 1.25 equiv) was added dropwise at –78 °C and the solution was allowed to warm to 22 °C. After 1 h, a saturated aqueous ammonium chloride solution (80 mL) was added and the mixture was extracted with ethyl acetate (2 × 50 mL). The combined organic solutions were washed with a saturated aqueous sodium chloride solution. The washed organic solution was dried over sodium sulfate and filtered. The filtrate was concentrated under reduced pressure and the residue was purified by flash chromatography on silica gel (4% diethyl ether in petroleum ether) which afforded benzodioxole **26d** as a colorless oil (6.50 g, 29.0 mmol, 84%).

TLC (5% ethyl acetate in cyclohexane): R_f = 0.63 (UV, $KMnO_4$).

^{1}H NMR (400 MHz, $CDCl_3$): δ 6.81 (s, 1H), 6.49 (s, 1H), 5.90 (s, 2H), 3.75 (s, 3H), 0.22 (s, 9H) ppm.

^{13}C NMR (101 MHz, $CDCl_3$): *δ* 160.2, 149.6, 141.4, 119.0, 113.4, 101.2, 94.4, 56.2, –0.7 ppm.

IR (ATR, neat): $\tilde{\nu}$ = 2954 (*w*), 2896 (*w*), 1610 (*w*), 1502 (*w*), 1475 (*m*), 1404 (*w*), 1385 (*w*), 1329 (*w*), 1261 (*w*), 1246 (*m*), 1223 (*w*), 1194 (*s*), 1170 (*m*), 1120 (*w*), 1045 (*m*), 977 (*m*), 937 (*m*), 835 (*s*), 763 (*w*) cm^{-1}.

HRMS (ESI): calcd. for $C_{11}H_{16}O_3Si$ $[M\text{-}e^-]^{\bullet+}$: 224.0863; found: 224.0861.

Synthesis of quinone 25d

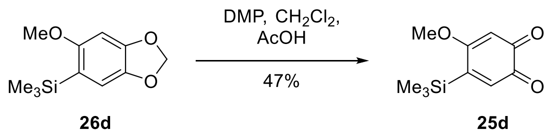

To a solution of benzodioxole **26d** (100 mg, 446 µmol, 1 equiv) in dichloromethane (5 mL) was added Dess-Martin peroiodinane (756 mg, 1.78 mmol, 4.00 equiv) followed by acetic acid (153 µL, 2.67 mmol, 6.00 equiv) and the resulting suspension was stirred for 20 h. The mixture was diluted with ethyl acetate (10 mL) and washed with a saturated aqueous sodium bicarbonate solution (3 × 10 mL). The organic solution was dried over sodium sulfate and filtered. The filtrate was concentrated and the residue was purified by flash chromatograhphy on silica gel (30% ethyl acetate in petroleum ether) which afforded quinone **25d** as a red solid (44.0 mg, 209 µmol, 47%).

TLC (30% ethyl acetate in cyclohexane): R_f = 0.38 (UV, $KMnO_4$).

^{1}H NMR (400 MHz, $CDCl_3$): *δ* 6.53 (s, 1H), 5.77 (s, 1H), 3.86 (s, 3H), 0.26 (s, 9H) ppm.

^{13}C NMR (101 MHz, $CDCl_3$): *δ* 180.2, 178.6, 172.9, 155.3, 137.8, 102.4, 56.8, –1.2 ppm.

IR (ATR, neat): $\tilde{\nu}$ = 2954 (*w*), 2902 (*w*), 2851 (*w*), 1732 (*w*), 1657 (*m*), 1645 (*w*), 1600 (*m*), 1560 (*w*), 1483 (*w*), 1459 (*w*), 1444 (*w*), 1393 (*w*), 1354 (*m*), 1297 (*w*), 1242 (*s*), 1205 (*w*), 1165 (*w*), 1116 (*w*), 1065 (*w*), 1013 (*w*), 995 (*w*), 908 (*w*), 841 (*s*), 797 (*w*), 763 (*w*) cm^{-1}.

HRMS (ESI): calcd. for $C_{10}H_{14}NaO_3Si$ $[M+Na]^+$: 233.0604; found: 233.0602.

Synthesis of benzodioxole 26e

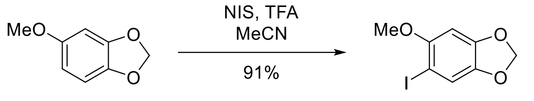

To a solution of arene **26b** (500 mg, 3.29 mmol, 1 equiv) in acetonitrile (6 mL) cooled to 0 °C was added trifluoroacetic acid (38 µL, 0.49 mmol, 0.15 equiv) followed by portionwise addition of *N*-iodosuccinimide (813 mg, 3.61 mmol, 1.10 equiv). After the addition was completed, the solution was allowed to warm to 22 °C and stirred for 30 min. The solution was washed with water (3 × 30 mL) and a saturated aqueous sodium chloride solution (30 mL). The washed solution was dried over sodium sulfate and filtered. The filtrate was concentrated under reduced pressure which afforded **26e** as a brown solid (835 mg, 3.00 mmol, 91%) that was used without further purification.

^{1}H NMR (400 MHz, $CDCl_3$): δ 7.18 (s, 1H), 6.52 (s, 1H), 5.95 (s, 2H), 3.81 (s, 3H) ppm.

The analytical data matched those reported previously.[23]

Synthesis of aldehyde 26f

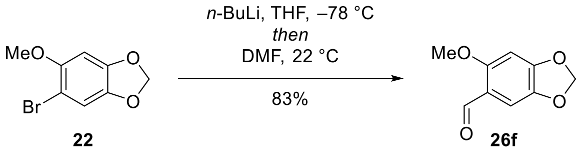

To a solution of aryl bromide **22** (300 mg, 1.30 mmol, 1 equiv) in tetrahydrofuran (5 mL) cooled to –78 °C was added dropwise a solution of *n*-butyllithium (1.60 M in hexanes, 1.01 mL, 1.62 mmol, 1.25 equiv) and the resulting solution was stirred for 5 min. *N,N*-dimethylformamide (201 µL, 2.60 mmol, 2.00 equiv) was added dropwise at –78 °C and the solution was allowed to warm to 22 °C. After 1 h, a saturated aqueous ammonium chloride solution (10 mL) was added and the mixture was extracted with ethyl acetate (2 × 10 mL). The combined organic solutions were washed with a saturated aqueous sodium chloride solution. The washed organic solution was dried over sodium sulfate and filtered. The filtrate was concentrated under reduced pressure and the residue was purified by flash chromatography on silica gel (20% ethyl acetate in petroleum ether) which afforded aldehyde **26f** as a colorless oil (194 mg, 1.08 mmol, 83%).

^{1}H NMR (400 MHz, $CDCl_3$): δ 10.27 (s, 1H), 7.26 (s, 1H), 6.54 (s, 1H), 6.00 (s, 2H), 3.88 (s, 3H) ppm.

The analytical data matched those reported previously.[24]

Synthesis of phenol SI-18

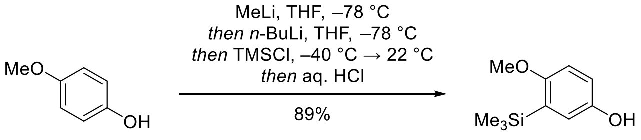

SI-19 **SI-18**

To a solution of phenol **SI-19** (3.0 g, 15 mmol, 1 equiv) in tetrahydrofuran (36 mL) cooled to –78 °C was added a solution of methyllithium (1.6 M in hexanes, 14 mL, 22 mmol, 1.5 equiv) followed by a solution of *n*-butyllithium (1.6 M in hexanes, 11 mL, 18 mmol, 1.2 equiv) upon which the mixture solidified. The mixture was allowed to warm to –40 °C and to the thawed mixture was added trimethylsilyl chloride (5.4 mL, 43 mmol, 2.9 equiv). The resulting solution was allowed to warm to 22 °C. The solution was then poured into an aqueous solution of hydrogen chloride (1.0 M, 75 mL, 5.0 equiv) and the mixture was stirred for 10 min. Ethyl acetate (50 mL) was added and the organic solution was separated and washed with a saturated aqueous solution of sodium chloride (50 mL). The washed solution was dried over sodium sulfate and filtered. The filtrate was concentrated under reduced pressure and the residue was purified by flash chromatography on silica gel (10% ethyl acetate in petroleum ether) which afforded phenol **SI-18** as a pale-yellow oil (2.6 g, 13 mmol, 89%).

TLC (20% ethyl acetate in cyclohexane): R_f = 0.45 (UV, $KMnO_4$).

^{1}H NMR (400 MHz, $CDCl_3$): δ 6.85 (d, J = 3.1 Hz, 1H), 6.79 (dd, J = 8.6, 3.1 Hz, 1H), 6.71 (d, J = 8.7 Hz, 1H), 4.49 (s, 1H), 3.76 (s, 3H), 0.25 (s, 9H) ppm.

^{13}C NMR (101 MHz, $CDCl_3$): δ 158.8, 149.2, 129.8, 121.8, 116.7, 111.0, 55.9, –0.9 ppm.

IR (ATR, neat): $\tilde{\nu}$ = 3333 (*w*), 2953 (*w*), 2899 (*w*), 1481 (*m*), 1465 (*w*), 1426 (*w*), 1277 (*w*), 1245 (*w*), 1221 (*m*), 1177 (*w*), 1141 (*w*), 1075 (*w*), 1031 (*w*), 908 (*w*), 838 (*s*), 809 (*w*), 763 (*w*), 737 (*w*), 686 (*w*) cm^{-1}.

HRMS (ESI): calcd. for $C_{10}H_{15}O_2Si$ $[M\text{-}H^+]^-$: 195.0847; found: 195.0840.

Synthesis of quinone 25d

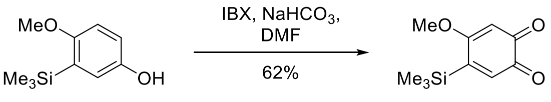

SI-18 **25d**

To a flask containing 2-iodoxybenzoic acid (1.43 g, 5.09 mmol, 1.00 equiv), phenol **SI-18** (1.00 g, 5.09 mmol, 1 equiv) and sodium bicarbonate (856 mg, 10.2 mmol, 2.00 equiv) was added *N,N*-dimethylformamide (25 mL) and the mixture was stirred for 30 min. Ethyl acetate (50 mL) was added to the resulting solution

and the organic layer was washed three times with a 10% aqueous solution of lithium chloride (3 × 50 mL). The organic layer was then washed with a saturated aqueous sodium bicarbonate solution (3 × 50 mL). The organic solution was dried over sodium sulfate and filtered. The filtrate was concentrated under reduced pressure which afforded quinone **25d** as a dark red solid (667 mg, 3.17 mmol, 62%) that was used without further purification.

The analytical data matched those obtained before (see above).

Synthesis of benzodioxane SI-20

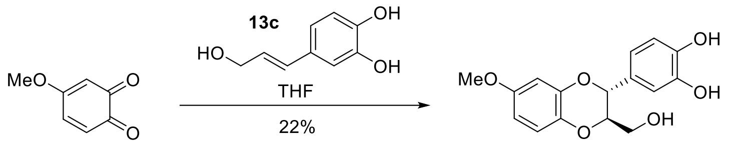

25b **SI-20**
6 : 1 *trans*:*cis*

To a solution of quinone **25b** (254 mg, 1.84 mmol, 1.80 equiv) in tetrahydrofuran (5 mL) was added caffeyl alcohol (**13c**) (170 mg, 1.02 mmol, 1 equiv) and the suspension was stirred for 20 h. The mixture was concentrated under reduced pressure and the residue was purified by flash chromatography on silica gel (50% ethyl acetate in petroleum ether) which afforded benzodioxane **SI-20** as a 6:1 mixture of *trans* and *cis* isomers respectively (brown solid, 68 mg, 0.22 mmol, 22%).

TLC (40% ethyl acetate in cyclohexane): R_f = 0.18 (UV, $KMnO_4$).

Note: Compound **SI-20** was obtained as a 6:1 mixture of *trans* and *cis* isomers. Only the NMR signals of the major *trans* isomer are reported.

^{1}H NMR (400 MHz, CD_3OD): δ 6.87 – 6.82 (m, 2H), 6.80 (d, J = 8.0 Hz, 1H), 6.75 (dd, J = 8.1, 2.0 Hz, 1H), 6.48 (d, J = 2.9 Hz, 1H), 6.44 (dd, J = 8.9, 2.9 Hz, 1H), 4.79 (d, J = 8.0 Hz, 1H), 3.92 (ddd, J = 7.8, 4.8, 2.6 Hz, 1H), 3.72 (s, 1H), 3.71 (s, 3H), 3.64 (dd, J = 12.2, 2.6 Hz, 1H), 3.46 (dd, J = 12.2, 4.8 Hz, 1H) ppm.

^{13}C NMR (101 MHz, CD_3OD): δ 155.7, 147.1, 146.6, 145.6, 138.9, 129.7, 120.4, 118.1, 116.3, 115.5, 108.2, 103.4, 79.7, 77.8, 62.2, 56.1 ppm.

IR (ATR, neat): $\tilde{\nu}$ = 3372 (*w*), 2938 (*w*), 1704 (*w*), 1602 (*w*), 1504 (*s*), 1445 (*w*), 1374 (*w*), 1260 (*m*), 1206 (*m*), 1155 (*s*), 1116 (*w*), 1036 (*m*), 901 (*w*), 817 (*w*), 740 (*w*) cm^{-1}.

HRMS (ESI): calcd. for $C_{16}H_{15}O_6$ $[M-H^+]^-$: 303.0874; found: 303.0875.

Synthesis of benzodioxane 48c

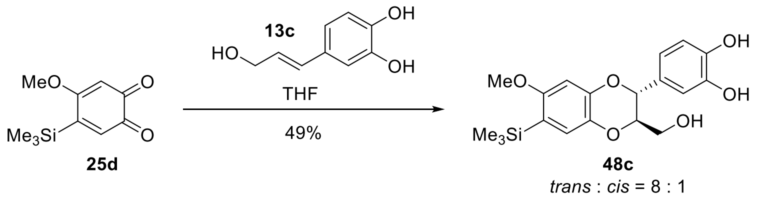

To a solution of quinone **25d** (64 mg, 0.30 mmol, 1.2 equiv) in tetrahydrofuran (2 mL) was added caffeyl alcohol (**13c**) (42 mg, 0.25 mmol, 1 equiv) and the suspension was stirred for 20 h. The mixture was concentrated under reduced pressure and the residue was purified by flash chromatography on silica gel (40% ethyl acetate in petroleum ether) which afforded benzodioxane **48c** as an 8:1 mixture of *trans* and *cis* isomers respectively (brown solid, 47 mg, 0.12 mmol, 49%).

TLC (40% ethyl acetate in cyclohexane): R_f = 0.22 (UV, $KMnO_4$).

Note: Compound **48c** was obtained as an 8:1 mixture of *trans* and *cis* isomers. Only the NMR signals of the major *trans* isomer are reported.

^{1}H NMR (400 MHz, CD_3OD): δ 6.91 (s, 1H), 6.85 (d, *J* = 2.0 Hz, 1H), 6.80 (d, *J* = 8.0 Hz, 1H), 6.75 (dd, *J* = 8.2, 2.0 Hz, 1H), 6.47 (d, *J* = 1.7 Hz, 1H), 4.80 (d, *J* = 8.0 Hz, 1H), 3.91 (ddt, *J* = 6.2, 3.9, 2.0 Hz, 1H), 3.73 – 3.68 (m, 3H), 3.64 (dd, *J* = 12.2, 2.6 Hz, 1H), 3.50 – 3.44 (m, 1H), 0.21 (s, 9H) ppm.

^{13}C NMR (101 MHz, CD_3OD): δ 160.4, 147.1, 146.8, 146.6, 138.5, 129.7, 123.7, 120.6, 120.4, 116.3, 115.5, 100.5, 79.6, 78.0, 62.2, 56.0, –0.7 ppm.

IR (ATR, neat): $\tilde{\nu}$ = 3364 (*br*, *w*), 2953 (*w*), 2898 (*w*), 1606 (*w*), 1587 (*m*), 1522 (*w*), 1481 (*m*), 1463 (*w*), 1446 (*w*), 1399 (*w*), 1301 (*m*), 1284 (*w*), 1244 (*w*), 1202 (*s*), 1150 (*w*), 1114 (*w*), 1067 (*m*), 1042 (*w*), 1011 (*w*), 998 (*w*), 906 (*w*), 836 (*s*), 777 (*w*), 762 (*w*), 690 (*w*) cm^{-1}.

HRMS (ESI): calcd. for $C_{19}H_{24}NaO_6Si$ $[M+Na]^+$: 399.1234; found: 399.1232.

Synthesis of benzodioxane 48d

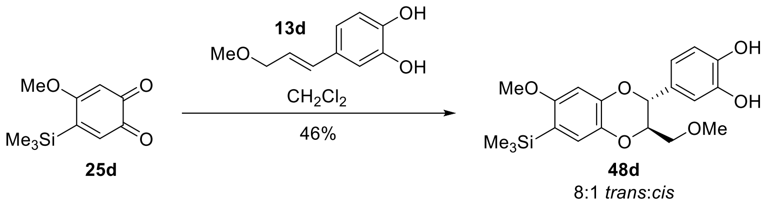

To a solution of quinone **25d** (250 mg, 1.19 mmol, 1 equiv) in dichloromethane (5 mL) was added styrene **13d** (236 mg, 1.31 mmol, 1.10 equiv) and the suspension was stirred for 20 h. The mixture was concentrated under reduced pressure and the residue was purified by flash chromatography on silica gel (25% ethyl acetate and 10% dichoromethane in petroleum ether) which afforded benzodioxane **48d** as an 8:1 mixture of *trans* and *cis* isomers respectively (brown solid, 214 mg, 548 µmol, 46%).

TLC (25% ethyl acetate 10% DCM in cyclohexane): R_f = 0.33 (UV, $KMnO_4$).

Note: The ^{13}C NMR in $CDCl_3$ could not be obtained due to desilylation of the compound during the course of the NMR experiment. Compound **48d** was obtained as an 8:1 mixture of *trans* and *cis* isomers. Only the NMR signals of the major *trans* isomer are reported.

^{1}H NMR (400 MHz, $CDCl_3$): δ 6.97 (d, *J* = 1.2 Hz, 1H), 6.83 (dd, *J* = 4.9, 3.0 Hz, 2H), 6.75 (dd, *J* = 8.2, 2.0 Hz, 1H), 6.43 (s, 1H), 4.84 (d, *J* = 8.0 Hz, 1H), 3.99 (ddd, *J* = 7.7, 4.6, 2.2 Hz, 1H), 3.67 (s, 3H), 3.48 (dd, *J* = 10.9, 2.3 Hz, 1H), 3.30 (s, 4H), 2.75 – 2.49 (m, 2H), 0.19 (d, *J* = 1.2 Hz, 9H) ppm.

^{1}H NMR (400 MHz, acetonitrile-d_3) δ 6.88 – 6.76 (m, 5H), 6.50 (s, 1H), 4.85 (d, *J* = 7.8 Hz, 1H), 4.09 (ddd, *J* = 7.5, 4.6, 2.3 Hz, 1H), 3.71 (s, 4H), 3.43 (dd, *J* = 11.1, 2.4 Hz, 1H), 3.29 – 3.20 (m, 4H), 0.22 (s, 9H) ppm.

^{13}C NMR (101 MHz, acetonitrile-d_3): δ 160.1, 146.4, 146.1, 145.6, 138.0, 129.9, 123.4, 120.69, 120.65, 116.2, 115.4, 100.7, 77.8, 77.4, 72.2, 59.4, 56.4, –0.8 ppm.

IR (ATR, neat): $\tilde{\nu}$ = 3381 (*w*), 2951 (*w*), 2900 (*w*), 1606 (*w*), 1587 (*m*), 1522 (*w*), 1481 (*m*), 1463 (*w*), 1447 (*w*), 1403 (*w*), 1370 (*w*), 1304 (*m*), 1285 (*w*), 1245 (*w*), 1203 (*s*), 1151 (*w*), 1113 (*w*), 1065 (*m*), 1022 (*w*), 1000 (*w*), 919 (*w*), 839 (*s*), 778 (*w*) cm^{-1}.

HRMS (ESI): calcd. for $C_{20}H_{26}NaO_6Si$ $[M+Na]^+$: 413.1391; found: 413.1387.

Synthesis of benzodioxane 52

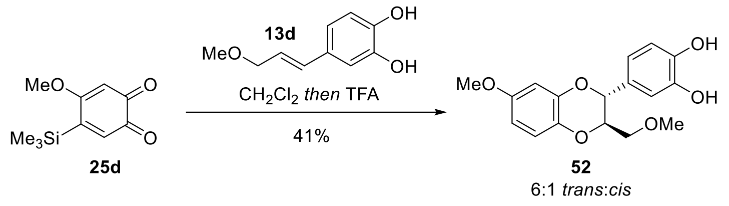

To a solution of quinone **25d** (500 mg, 2.38 mmol, 1 equiv) in dichloromethane (10 mL) was added styrene **13d** (471 mg, 2.62 mmol, 1.10 equiv) and the suspension was stirred for 20 h. Trifluoroacetic acid (275 µL, 3.56 mmol, 1.50 equiv) was added and the resulting dark brown solution was stirred for 10 min. The solution was added to a saturated aqueous solution of sodium bicarbonate (50 mL) and the mixture was vigorously stirred for 15 min. The mixture was extracted with ethyl acetate (3 × 20 mL). The organic solution was dried oved sodium sulfate and filtered. The filtrate was concentrated under reduced pressure and the residue was purified by flash chromatography on silica gel (30% ethyl acetate and 10% dichoromethane in petroleum ether) which afforded benzodioxane **52** as a 6:1 mixture of *trans* and *cis* isomers respectively (brown solid, 307 mg, 964 µmol, 41%).

TLC (25% ethyl acetate in cyclohexane): R_f = 0.29 (UV, $KMnO_4$).

Note: Compound **52** was obtained as a 6:1 mixture of *trans* and *cis* isomers. Only the NMR signals of the major *trans* isomer are reported.

^{1}H NMR (500 MHz, methanol-*d₄*): δ 6.87 – 6.73 (m, 6H), 6.50 (d, *J* = 2.9 Hz, 1H), 6.45 (dd, *J* = 8.8, 2.9 Hz, 1H), 4.84 (d, *J* = 7.8 Hz, 1H), 4.11 – 4.07 (m, 1H), 3.71 (s, 3H), 3.44 (dd, *J* = 11.1, 2.5 Hz, 1H), 3.24 (s, 4H) ppm.

^{13}C NMR (101 MHz, methanol-d_4): δ 155.3, 146.1, 145.7, 145.2, 138.3, 129.9, 120.7, 117.9, 116.3, 115.5, 108.1, 103.3, 77.8, 77.3, 72.2, 59.4, 56.3 ppm.

IR (ATR, neat): $\tilde{\nu}$ = 3394 (*w*), 2931 (*w*), 2836 (*w*), 1602 (*w*), 1504 (*s*), 1465 (*w*), 1446 (*w*), 1359 (*w*), 1316 (*w*), 1284 (*w*), 1262 (*w*), 1213 (*w*), 1198 (*m*), 1157 (*m*), 1115 (*w*), 1065 (*w*), 1035 (*w*), 956 (*w*), 915 (*w*), 887 (*w*), 830 (*w*), 815 (*w*) cm^{-1}.

HRMS (ESI): calcd. for $C_{17}H_{18}NaO_6$ [M+Na]$^+$: 341.0996; found: 341.0991.

Synthesis of benzodioxane SI-21

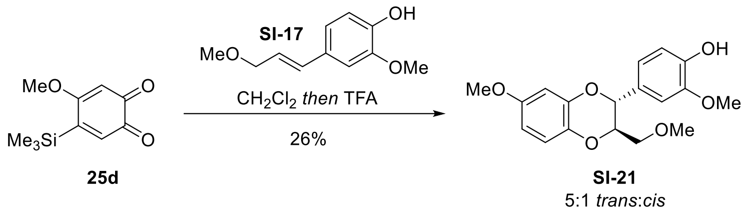

To a solution of quinone **25d** (330 mg, 1.57 mmol, 1 equiv) in dichloromethane (10 mL) was added styrene **SI-17** (335 mg, 1.73 mmol, 1.10 equiv) and the suspension was stirred for 20 h. Trifluoroacetic acid (242 µL, 3.14 mmol, 2.00 equiv) was added and the resulting dark brown solution was stirred for 10 min. The solution was added to a saturated aqueous solution of sodium bicarbonate (50 mL) and the mixture was vigorously stirred for 15 min. The mixture was extracted with ethyl acetate (3 × 20 mL). The organic solution was dried oved sodium sulfate and filtered. The filtrate was concentrated under reduced pressure and the residue was purified by flash chromatography on silica gel (30% ethyl acetate in petroleum ether) which afforded benzodioxane **SI-21** as a 5:1 mixture of *trans* and *cis* isomers respectively (brown solid, 136 mg, 410 µmol, 26%).

TLC (30% ethyl acetate in cyclohexane): R_f = 0.32 (UV, $KMnO_4$).

Note: Compound **SI-21** was obtained as a 5:1 mixture of *trans* and *cis* isomers. Only the NMR signals of the major *trans* isomer are reported.

^{1}H NMR (400 MHz, $CDCl_3$): δ 6.95 – 6.90 (m, 4H), 6.54 (d, *J* = 2.9 Hz, 1H), 6.46 (dd, *J* = 8.9, 3.0 Hz, 1H), 5.69 (s, 1H), 4.98 (d, *J* = 8.1 Hz, 1H), 4.03 (ddd, *J* = 8.1, 4.1, 2.3 Hz, 1H), 3.92 (s, 3H), 3.74 (s, 3H), 3.59 – 3.52 (m, 1H), 3.34 (s, 3H), 3.30 – 3.25 (m, 1H) ppm.

^{13}C NMR (101 MHz, $CDCl_3$): δ 154.4, 147.0, 146.4, 144.2, 137.6, 128.7, 120.9, 117.6, 114.7, 109.6, 107.6, 102.5, 77.4, 76.7, 71.5, 59.6, 56.1, 55.8 ppm.

IR (ATR, neat): $\tilde{\nu}$ = 3437 (*w*), 2925 (*w*), 2837 (*w*), 1602 (*w*), 1504 (*s*), 1465 (*w*), 1453 (*w*), 1435 (*w*), 1371 (*w*), 1320 (*w*), 1261 (*w*), 1236 (*w*), 1213 (*m*), 1199 (*w*), 1157 (*m*), 1123 (*w*), 1068 (*w*), 1034 (*m*), 915 (*w*), 883 (*w*), 827 (*w*), 798 (*w*) cm^{-1}.

HRMS (ESI): calcd. for $C_{18}H_{20}NaO_6Si$ $[M+Na]^+$: 355.1152; found: 355.1148.

Synthesis of diol 50

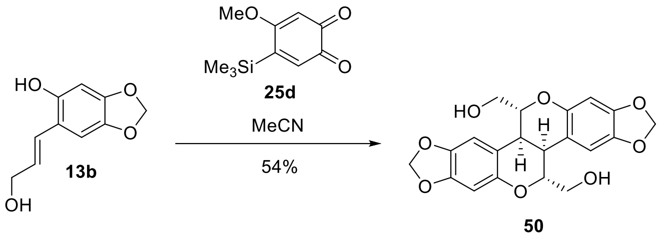

To a stirred solution of quinone **25d** (39 mg, 0.19 mmol, 1.2 equiv) in acetonitrile (1 mL) was added alcohol **13b** (30 mg, 0.15 mmol, 1 equiv). After 1 h, the solvent was removed under reduced pressure and the residue was purified by flash chromatography on silica gel (25% ethyl acetate in petroleum ether to 30% ethyl acetate in petroleum ether) which afforded the diol **50** as a white solid (32 mg, 83 µmol, 54%).

TLC (35% ethyl acetate in cyclohexane): R_f = 0.33 (UV, $KMnO_4$).

^{1}H NMR (500 MHz, acetonitrile-d_3): δ 7.30 (s, 2H), 6.73 (s, 2H), 6.42 (s, 2H), 5.86 (s, 4H), 4.85 (d, J = 4.2 Hz, 2H), 4.24 – 4.16 (m, 2H), 3.89 (dd, J = 9.2, 3.2 Hz, 2H), 3.06 – 2.98 (m, 2H) ppm.

^{13}C NMR (126 MHz, acetonitrile-d_3): δ 150.3, 148.3, 141.8, 119.8, 106.9, 102.2, 99.1, 83.9, 72.7, 54.3 ppm.

IR (ATR, neat): $\tilde{v}$ = 3319 (*br*, *w*), 2887 (*w*), 1630 (*w*), 1502 (*w*), 1484 (*s*), 1442 (*m*), 1405 (*w*), 1303 (*w*), 1246 (*w*), 1215 (*w*), 1170 (*s*), 1072 (*w*), 1035 (*s*), 933 (*m*), 860 (*w*), 818 (*w*), 766 (*w*) cm^{-1}.

HRMS (ESI): calcd. for $C_{20}H_{18}NaO_8$ $[M+H]^+$: 409.0894; found: 409.0887.

Formal [4+2] cycloaddition under photochemical conditions:

Synthesis of benzodioxane 48c

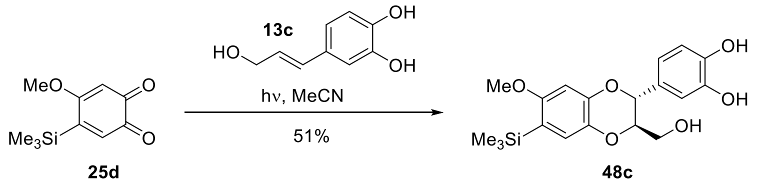

A solution of *ortho*-quinone **25d** (20 mg, 95 µmol, 2.0 equiv) and styrene **13c** (7.9 mg, 48 µmol, 1 equiv) in acetonitrile (0.5 mL) was irradiated (12W, 1520 lm, JBL Reptil LED Daylight lamp) for 30 min. The solution was concentrated under reduced pressure and the residue was purified by flash chromatography on silica gel (40% ethyl acetate in petroleum ether) which afforded benzodioxane **48c** as a colorless oil (9.2 mg, 24 µmol, 51%).

The analytical data matched those obtained before (see above).

Synthesis of benzodioxane 49a

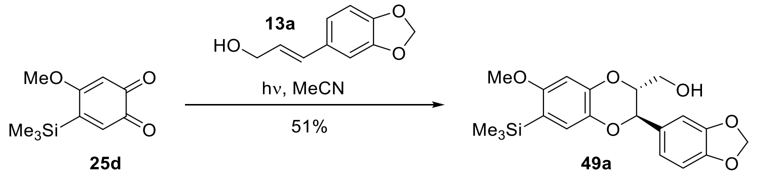

A solution of *ortho*-quinone **25d** (20 mg, 95 µmol, 2.0 equiv) and styrene **13a** (8.5 mg, 48 µmol, 1 equiv) in acetonitrile (0.5 mL) was irradiated (12W, 1520 lm, JBL Reptil LED Daylight lamp) for 30 min. The solution was concentrated under reduced pressure and the residue was purified by flash chromatography on silica gel (15 % ethyl acetate in petroleum ether grading to 20% ethyl acetate in petroleum ether) which afforded benzodioxane **49a** as a colorless oil (9.5 mg, 24 µmol, 51%).

TLC (20% ethyl acetate in cyclohexane): R_f = 0.36 (UV, $KMnO_4$).

^{1}H NMR (400 MHz, $CDCl_3$): δ 6.98 (s, 1H), 6.94 – 6.88 (m, 2H), 6.84 (d, *J* = 8.4 Hz, 1H), 6.50 (s, 1H), 5.99 (s, 2H), 4.86 (d, *J* = 8.2 Hz, 1H), 4.03 (ddd, *J* = 8.2, 4.1, 2.7 Hz, 1H), 3.81 – 3.73 (m, 4H), 3.56 (dt, *J* = 11.7, 5.6 Hz, 1H), 1.91 (br s, 1H), 0.23 (s, 9H) ppm.

^{13}C NMR (101 MHz, $CDCl_3$): δ 159.4, 148.4, 148.3, 144.6, 137.4, 130.3, 123.3, 121.5, 120.8, 108.7, 107.8, 101.5, 99.5, 78.7, 76.2, 61.9, 55.8, –0.8 ppm.

IR (ATR, neat): $\tilde{\nu}$ = 3464 (*w*), 2953 (*w*), 2924 (*w*), 2898 (*w*), 2856 (*w*), 1606 (*w*), 1587 (*m*), 1504 (*w*), 1484 (*m*), 1463 (*w*), 1447 (*w*), 1399 (*w*), 1378 (*w*), 1303 (*m*), 1272 (*w*), 1249 (*m*), 1204 (*s*), 1152 (*w*), 1090 (*w*), 1066 (*w*), 1038 (*m*), 1001 (*w*), 935 (*w*), 898 (*w*), 840 (*s*), 815 (*w*), 762 (*w*) cm^{-1}.

HRMS (ESI): calcd. for $C_{20}H_{24}NaO_6Si$ [M+Na]$^+$: 411.1234; found: 411.1222.

Synthesis of benzodioxanes SI-22a and SI-22b

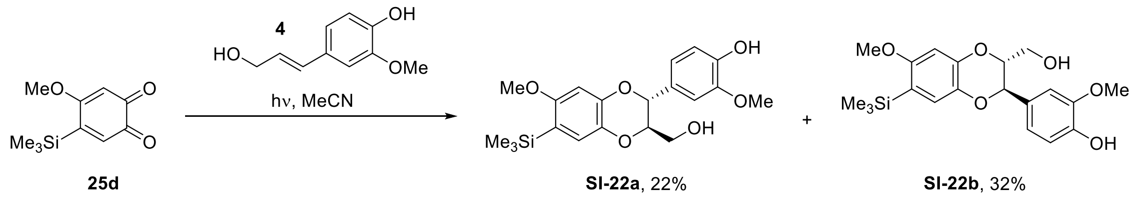

A solution of *ortho*-quinone **25d** (20 mg, 95 µmol, 2.0 equiv) and styrene **4** (8.6 mg, 48 µmol, 1 equiv) in acetonitrile (0.5 mL) was irradiated (12W, 1520 lm, JBL Reptil LED Daylight lamp) for 30 min. The solution was concentrated under reduced pressure and the residue was purified by flash chromatography on silica gel (20% ethyl acetate in petroleum ether grading to 30% ethyl acetate in petroleum ether) which afforded benzodioxane **SI-22a** as a colorless oil (4.1 mg, 10 µmol, 22%) and benzodioxane **SI-22b** as a colorless oil (6.0 mg, 15 µmol, 32%).

Characterization data for benzodioxane **SI-22a**:

TLC (25% ethyl acetate in cyclohexane): R_f = 0.22 (UV, $KMnO_4$).

^{1}H NMR (400 MHz, $CDCl_3$): δ 6.98 (s, 1H), 6.96 – 6.92 (m, 2H), 6.91 (d, J = 1.6 Hz, 1H), 6.51 (s, 1H), 5.70 (s, 1H), 4.94 (d, J = 8.3 Hz, 1H), 3.97 (ddd, J = 8.3, 4.1, 2.8 Hz, 1H), 3.92 (s, 3H), 3.81 – 3.69 (m, 4H), 3.53 (d, J = 12.0 Hz, 1H), 1.96 (br s, 1H), 0.25 (s, 9H) ppm.

^{13}C NMR (101 MHz, $CDCl_3$): δ 159.4, 147.0, 146.5, 145.2, 136.8, 128.2, 122.9, 120.9, 120.7, 114.8, 109.6, 99.8, 78.0, 76.9, 62.0, 56.2, 55.8, –0.7 ppm.

IR (ATR, neat): $\tilde{\nu}$ = 3456 (*w*), 2953 (*w*), 1605 (*w*), 1588 (*m*), 1519 (*m*), 1481 (*w*), 1464 (*w*), 1434 (*w*), 1400 (*w*), 1303 (*m*), 1275 (*w*), 1244 (*w*), 1204 (*s*), 1152 (*w*), 1124 (*w*), 1068 (*m*), 1033 (*w*), 839 (*m*), 774 (*w*) cm^{-1}.

HRMS (ESI): calcd. for $C_{20}H_{26}NaO_6Si$ [M+Na]$^+$: 413.1391; found: 413.1386.

Characterization data for benzodioxane **SI-22b**:

TLC (25% ethyl acetate in cyclohexane): R_f = 0.14 (UV, $KMnO_4$).

^{1}H NMR (400 MHz, $CDCl_3$): δ 7.00 (s, 1H), 6.95 – 6.91 (m, 3H), 6.52 (d, *J* = 2.1 Hz, 1H), 5.71 (s, 1H), 4.86 (d, *J* = 8.3 Hz, 1H), 4.06 (ddd, *J* = 8.4, 4.2, 2.8 Hz, 1H), 3.92 (s, 3H), 3.79 – 3.74 (m, 4H), 3.58 – 3.51 (m, 1H), 1.91 (br s, 1H), 0.23 (s, 9H) ppm.

^{13}C NMR (101 MHz, $CDCl_3$): δ 159.4, 147.1, 146.5, 144.6, 137.5, 128.4, 123.3, 121.0, 120.8, 114.8, 109.6, 99.6, 78.7, 76.3, 62.0, 56.2, 55.8, –0.8 ppm.

IR (ATR, neat): $\tilde{\nu}$ = 3413 (*w*), 2924 (*m*), 2854 (*w*), 1605 (*w*), 1588 (*w*), 1518 (*m*), 1482 (*w*), 1464 (*w*), 1399 (*w*), 1302 (*w*), 1275 (*w*), 1243 (*w*), 1203 (*s*), 1154 (*w*), 1125 (*w*), 1093 (*w*), 1066 (*w*), 1034 (*w*), 1001 (*w*), 839 (*s*), 735 (*w*) cm^{-1}.

HRMS (ESI): calcd. for $C_{20}H_{26}NaO_6Si$ $[M+Na]^+$: 413.1391; found: 413.1386.

Synthesis of benzodioxanes SI-23a and SI-23b

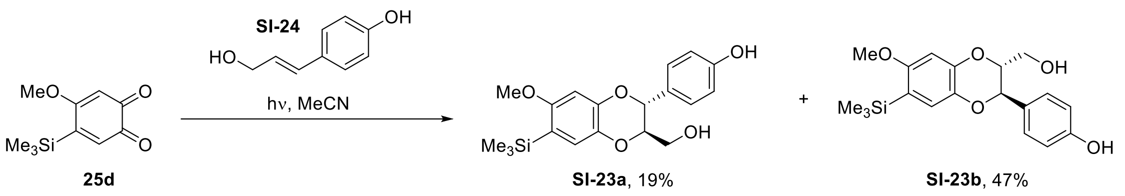

A solution of *ortho*-quinone **25d** (20 mg, 95 µmol, 2.0 equiv) and styrene **SI-24** (7.1 mg, 48 µmol, 1 equiv) in acetonitrile (0.5 mL) was irradiated (12W, 1520 lm, JBL Reptil LED Daylight lamp) for 30 min. The solution was concentrated under reduced pressure and the residue was purified by flash chromatography on silica gel (20% ethyl acetate in petroleum ether grading to 25% ethyl acetate in petroleum ether) which afforded benzodioxane **SI-23a** as a colorless oil (3.3 mg, 9.2 µmol, 19%) and benzodioxane **SI-23b** as a colorless oil (8.1 mg, 22 µmol, 47%).

Characterization data for benzodioxane **SI-23a**:

TLC (25% ethyl acetate in cyclohexane): R_f = 0.17 (UV, $KMnO_4$).

^{1}H NMR (400 MHz, $CDCl_3$): δ 7.33 – 7.27 (m, 2H), 6.98 (s, 1H), 6.90 – 6.86 (m, 2H), 6.50 (s, 1H), 5.01 (br s, 1H), 4.96 (d, *J* = 8.2 Hz, 1H), 3.97 (ddd, *J* = 8.2, 4.1, 2.7 Hz, 1H), 3.77 (d, *J* = 11.3 Hz, 1H), 3.72 (s, 3H), 3.56 – 3.48 (m, 1H), 1.97 (br s, 1H), 0.25 (s, 9H) ppm.

^{13}C NMR (101 MHz, $CDCl_3$): δ 159.4, 156.4, 145.3, 136.8, 129.1, 128.7, 122.9, 120.7, 115.9, 99.8, 78.0, 76.6, 62.0, 55.8, –0.7 ppm.

IR (ATR, neat): $\tilde{\nu}$ = 3393 (*br*, *w*), 2953 (*w*), 2923 (*w*), 2853 (*w*), 1650 (*w*), 1588 (*m*), 1518 (*w*), 1482 (*m*), 1463 (*w*), 1400 (*w*), 1367 (*w*), 1304 (*w*), 1246 (*w*), 1205 (*s*), 1172 (*w*), 1151 (*w*), 1093 (*w*), 1067 (*m*), 1040 (*w*), 1012 (*w*), 906 (*w*), 838 (*s*), 761 (*w*), cm^{-1}.

HRMS (ESI): calcd. for $C_{19}H_{24}NaO_5Si$ $[M+Na]^+$: 383.1285; found: 383.1276.

Characterization data for benzodioxane **SI-23b**:

TLC (25% ethyl acetate in cyclohexane): R_f = 0.11 (UV, $KMnO_4$).

^{1}H NMR (400 MHz, $CDCl_3$): δ 7.28 – 7.23 (m, 2H), 6.86 – 6.80 (m, 3H), 6.54 (s, 1H), 4.80 (d, *J* = 8.0 Hz, 1H), 4.01 (ddd, *J* = 8.0, 4.6, 2.5 Hz, 1H), 3.73 (d, *J* = 1.5 Hz, 3H), 3.66 (dd, *J* = 12.2, 2.6 Hz, 1H), 3.45 (dd, *J* = 12.2, 4.7 Hz, 1H), 0.20 (s, 9H) ppm.

^{13}C NMR (101 MHz, $CDCl_3$): δ 160.6, 159.2, 146.4, 139.0, 130.0, 129.3, 123.6, 120.5, 116.4, 100.5, 80.3, 77.2, 62.1, 56.0, –0.7 ppm.

IR (ATR, neat): $\tilde{\nu}$ = 3393 (*br*, *w*), 2954 (*w*), 2928 (*w*), 2904 (*w*), 2855 (*w*), 1613 (*w*), 1586 (*m*), 1517 (*w*), 1480 (*m*), 1462 (*w*), 1400 (*w*), 1349 (*w*), 1303 (*w*), 1247 (*w*), 1204 (*s*), 1172 (*w*), 1151 (*w*), 1091 (*w*), 1064 (*w*), 1041 (*w*), 1003 (*w*), 903 (*w*), 876 (*w*), 838 (*s*), 800 (*w*), 762 (*w*) cm^{-1}.

HRMS (ESI): calcd. for $C_{19}H_{24}NaO_5Si$ $[M+Na]^+$: 383.1285; found: 383.1276.

Synthesis of benzodioxane SI-25

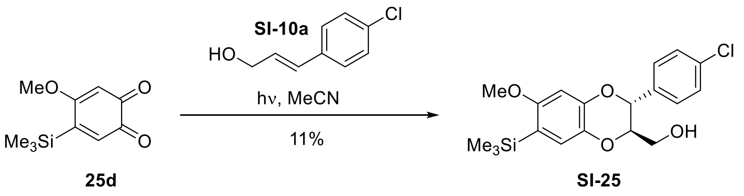

A solution of *ortho*-quinone **25d** (20 mg, 95 µmol, 2.0 equiv) and styrene **SI-10a** (8.0 mg, 48 µmol, 1 equiv) in acetonitrile (0.5 mL) was irradiated (12W, 1520 lm, JBL Reptil LED Daylight lamp) for 30 min. The solution was concentrated under reduced pressure and the residue was purified by flash chromatography on silica gel (10% ethyl acetate in petroleum ether grading to 15% ethyl acetate in petroleum ether) which afforded benzodioxane **SI-25** as a colorless oil (2.0 mg, 5.3 µmol, 11%).

TLC (20% ethyl acetate in cyclohexane): R_f = 0.31 (UV, $KMnO_4$).

^{1}H NMR (400 MHz, $CDCl_3$): δ 7.45 – 7.35 (m, 4H), 6.98 (s, 1H), 6.51 (s, 1H), 4.97 (d, J = 8.2 Hz, 1H), 4.01 (ddd, J = 8.2, 3.7, 2.6 Hz, 1H), 3.83 – 3.79 (m, 1H), 3.76 (s, 3H), 3.50 (d, J = 12.6 Hz, 1H), 1.90 (br s, 1H), 0.23 (s, 9H) ppm.

^{13}C NMR (101 MHz, $CDCl_3$): δ 159.5, 144.5, 137.2, 135.2, 135.1, 129.3, 129.0, 123.3, 121.0, 99.6, 78.6, 75.6, 61.7, 55.8, –0.8 ppm.

IR (ATR, neat): $\tilde{\nu}$ = 3411 (*br*, *w*), 2924 (*m*), 1648 (*w*), 1586 (*m*), 1482 (*m*), 1463 (*w*), 1397 (*w*), 1304 (*m*), 1246 (*w*), 1204 (*s*), 1153 (*w*), 1089 (*m*), 1064 (*w*), 1003 (*w*), 839 (*s*), 763 (*w*) cm^{-1}.

HRMS (ESI): calcd. for $C_{19}H_{23}O_4SiCl$ [M-e$^-$]$^{•+}$: 378.1049; found: 378.1049.

Synthesis of benzodioxane SI-26

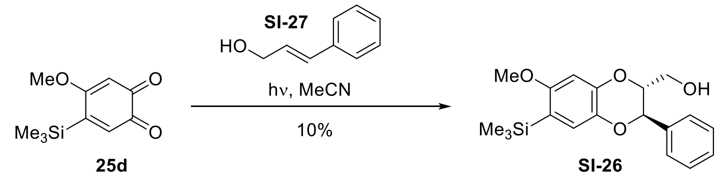

A solution of *ortho*-quinone **25d** (20 mg, 95 µmol, 1 equiv) and styrene **SI-27** (14 mg, 0.10 mmol, 1.1 equiv) in acetonitrile (0.5 mL) was irradiated (12W, 1520 lm, JBL Reptil LED Daylight lamp) for 20 h. The solution was concentrated under reduced pressure and the residue was purified by flash chromatography on silica gel (20 % ethyl acetate in petroleum ether) which afforded the product contaminated with styrene **SI-27**. The two compounds were further separated using a semi-preparative HPLC (15% ethyl acetate in hexane grading to 25% ethyl acetate in hexane) which afforded benzodioxane **SI-26** as a red solid (3.3 mg, 9.6 µmol, 10%).

TLC (30% ethyl acetate in cyclohexane): R_f = 0.55 (UV, $KMnO_4$).

^{1}H NMR (500 MHz, $CDCl_3$): δ 7.49 – 7.35 (m, 5H), 7.00 (s, 1H), 6.52 (s, 1H), 4.95 (d, J = 8.2 Hz, 1H), 4.08 (ddd, J = 8.2, 4.1, 2.7 Hz, 1H), 3.81 – 3.73 (m, 4H), 3.53 (ddd, J = 12.1, 7.7, 4.2 Hz, 1H), 1.89 (t, J = 6.6 Hz, 1H), 0.23 (s, 9H) ppm.

^{13}C NMR (101 MHz, $CDCl_3$): δ 159.4, 144.6, 137.5, 136.6, 129.3, 129.1, 127.6, 123.3, 120.8, 99.6, 78.7, 76.4, 61.9, 55.8, –0.8 ppm.

IR (ATR, neat): $\tilde{\nu}$ = 3458 (*w*), 2953 (*w*), 2926 (*m*), 2855 (*w*), 1646 (*w*), 1606 (*w*), 1588 (*m*), 1482 (*m*), 1463 (*w*), 1391 (*w*), 1351 (*w*), 1303 (*m*), 1270 (*w*), 1245 (*w*), 1204 (*s*), 1152 (*w*), 1095 (*w*), 1065 (*w*), 1026 (*w*), 1002 (*m*), 902 (*w*), 839 (*s*), 813 (*w*), 761 (*w*), 699 (*w*) cm^{-1}.

HRMS (ESI): calcd. for $C_{19}H_{24}O_4Si$ $[M-e^-]^{\bullet+}$: 344.1438; found: 344.1433.

Synthesis of benzodioxane SI-28

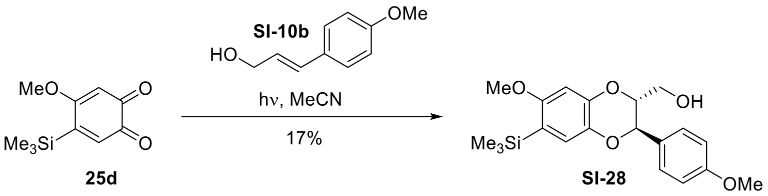

A solution of *ortho*-quinone **25d** (20 mg, 95 µmol, 1 equiv) and styrene **SI-10b** (17 mg, 0.10 mmol, 1.1 equiv) in acetonitrile (0.5 mL) was irradiated (12W, 1520 lm, JBL Reptil LED Daylight lamp) for 1 h. The solution was concentrated under reduced pressure and the residue was purified by flash chromatography on silica gel (20% ethyl acetate in petroleum ether) which afforded the product contaminated with styrene **SI-10b.** The two compounds were further separated using a semi-preparative HPLC (15% ethyl acetate in hexane grading to 25% ethyl acetate in hexane) which afforded benzodioxane **SI-28** as a red solid (6.1 mg, 16 µmol, 17%).

TLC (40% ethyl acetate in cyclohexane): R_f = 0.63 (UV, $KMnO_4$).

^{1}H NMR (400 MHz, $CDCl_3$): δ 7.40 – 7.31 (m, 2H), 7.04 – 6.90 (m, 3H), 6.51 (s, 1H), 4.89 (d, *J* = 8.2 Hz, 1H), 4.07 (ddd, *J* = 8.3, 4.2, 2.8 Hz, 1H), 3.83 (s, 3H), 3.80 – 3.70 (m, 4H), 3.53 (ddd, *J* = 12.2, 7.9, 4.2 Hz, 1H), 1.87 (dd, *J* = 8.0, 5.2 Hz, 1H), 0.23 (s, 9H) ppm.

^{13}C NMR (101 MHz, $CDCl_3$): δ 160.4, 159.4, 144.6, 137.6, 128.9, 128.6, 123.3, 120.7, 114.5, 99.6, 78.7 76.1, 62.0, 55.8, 55.5, –0.8 ppm.

IR (ATR, neat): $\tilde{\nu}$ = 3424 (*w*), 2954 (*w*), 1613 (*w*), 1587 (*m*), 1515 (*m*), 1481 (*m*), 1463 (*w*), 1400 (*w*), 1303 (*m*), 1249 (*m*), 1204 (*s*), 1175 (*w*), 1152 (*w*), 1094 (*w*), 1065 (*w*), 1034 (*m*), 1002 (*w*), 836 (*s*), 757 (*w*), 640 (*w*) cm^{-1}.

HRMS (ESI): calcd. for $C_{20}H_{27}O_5Si$ $[M+H]^+$: 375.1622; found: 375.1608.

Scheme 1: Discussion of mechanisms of formation of products observed in the model studies.

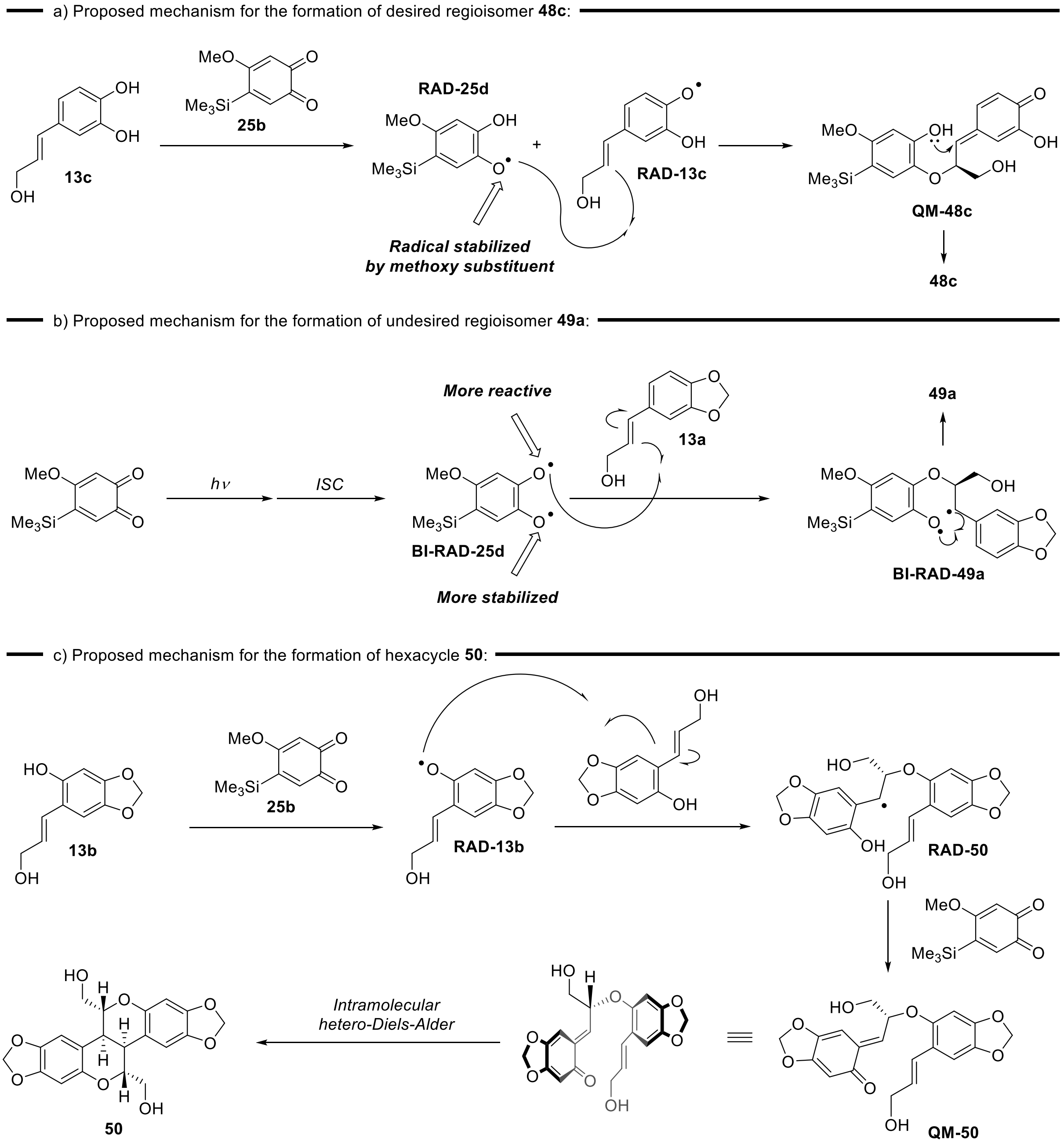

A) To explain the regiochemical outcome of the reaction of styrene **13c** with quinone **25d** we hypothesize that the quinone oxidizes **13c** by performing a hydrogen atom transfer (HAT) or a single electron transfer (SET) followed by proton exchange (Scheme 1a). In either case, the transformation generates a pair of radicals **RAD-25d** and **RAD-13c**. Presumably, the oxygen centered radical of **RAD-25d** is found predominantly in the depicted position due to stabilization by the methoxy group in conjugation. The radical pair is in close contact after formation (solvent cage effect), making it possible for **RAD-25d** to react with **RAD-13c** via radical recombination. Successively, intermediate quinone methide **QM-48c** undergoes an intramolecular attack by the phenol generating the observed regioisomer **48c**.

B) Our hypothesis for the switch of regioselectivity under photochemical conditions is as follows: When quinone **25d** is irradiated, it first enters the singlet excited state that rapidly undergoes intersystem crossing (ISC) generating the triplet excited state (Scheme 1b). The triplet state, depicted as biradical **BI-RAD-25d**, features two oxygen-centered radicals. The radical in conjugation with the methoxy group is presumably more stabilized and thereby less reactive than the radical *para* to the silyl substituent. Attack of the more reactive radical on the more electron-rich olefinic position of **13a** results in formation of biradical **BI-RAD-49a.** This intermediate is via radical recombination transformed into observed regioisomer **49a**.

Same regiochemical outcome as for **13a** was observed for styrene substrates **SI-10b** and **SI-27**. Styrenes **4** and **SI-24** produced mixtures of the regioisomers, For **13c**, we observed predominant formation of isomer **48c** even under photochemical conditions. We hypothesize that in the case of styrenes bearing phenolic groups, **BI-RAD-25d** can either attack the electron-rich position of the olefin or perform a HAT from the styrene. For compounds **4** and **SI-24**, these two processes are competitive giving rise to mixtures of regioisomers. For styrene **13c**, the HAT pathway is dominant.

C) We hypothesize that due to the high electron density of styrene **13b** (Scheme 1c), the generated radical **RAD-13b** might be persistent enough to break out of the solvent cage without reacting with **RAD-25d**. This enables it to react with a neutral molecule of **13b** generating highly stabilized benzylic radical **RAD-50.** Although this intermediate can already undergo a cyclization, we believe that the generation of a secondary non-stabilized radical is unfavorable. Instead, oxidation of **RAD-50** via HAT forms quinone methide **QM-50** which undergoes an intramolecular Diels-Alder reaction to deliver hexacycle **50**. The stereospecificity of the pericyclic reaction might explain why a single stereoisomer of **50** was observed.

Methylenation investigation:

Table 1: Screening of methylenation conditions

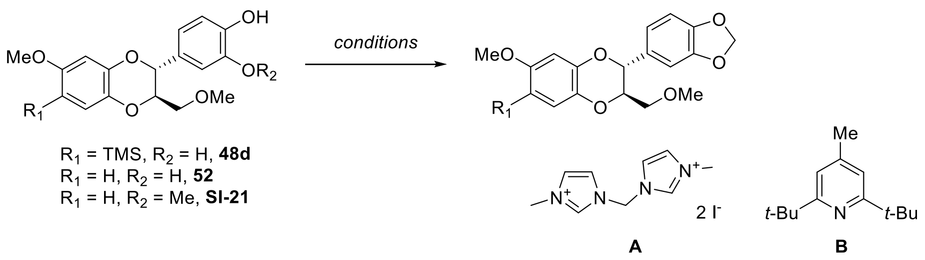

entry	conditions	*result*
1, **48d**	CH_2I_2, Cs_2CO_3, DMF, 100 °C	32%
2, **48d**	CH_2I_2, DBU, DMF, 100 °C	33%
3,[25] **48d**	CH_2I_2, Cs_2CO_3, CuO (10 mol%), DMF, 100 °C	26%[a]
4, **48d**	**A**, DMF, 22 °C → 100 °C	no reaction
5, **48d**	**A**, Cs_2CO_3, DMF, 22 °C → 100 °C	no reaction
6, **48d**	$CH_2(OTf)_2$, Cs_2CO_3, DMF, 22 °C → 100 °C	no reaction
7, **48d**	$CH_2(OTf)_2$, DBU, DMF, 22 °C → 100 °C	no reaction
8, **48d**	$CH_2(OTf)_2$, **B**, DMF, 22 °C → 100 °C	no reaction
9, **48d**	$(CH_2O)_n$, TsOH, PhMe, 22 °C → 100 °C	desilylation
10, **48d**	$CH_2(OMe)_2$, TsOH, PhMe, 22 °C → 100 °C	desilylation
11, **52**	TCDI, CH_2Cl_2 *then* AIBN, Bu_3SnH, PhMe, 110 °C	17% over 2 steps
12, **52**	TCDI, CH_2Cl_2 *then* AIBN, Ph_3SnH, PhMe, 110 °C	42% over 2 steps
13, **52**	TCDI, CH_2Cl_2 *then* Et_3B, O_2, Ph_3SnH, PhMe, 50 °C	48% over 2 steps
14,[26,27] **SI-21**	Fe[L], H_2O_2, MeCN [b]	complex mixture
15,[28] **SI-21**	Ag_2O, CH_2Cl_2 *then* hν	complex mixture after first step
16,[28] **SI-21**	$K_3[Fe(CN)_6]$, KOH, H_2O, CH_2Cl_2 *then* hν	complex mixture after first step
17,[28] **SI-21**	DDQ, CH_2Cl_2 *then* hν	complex mixture after first step

[a] NMR yield; [b] [L] = 5,10,15,20-tetrakis(pentafluorophenyl)porphyrin

Synthesis of benzodioxane SI-29

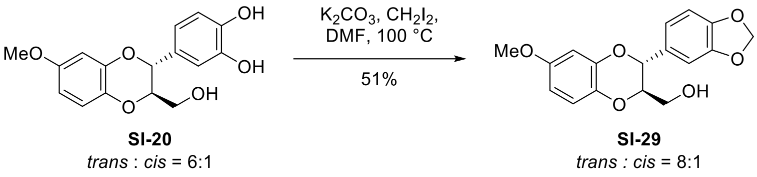

To a stirred solution of catechol **SI-20** (66 mg, 0.22 mmol, 1 equiv) in *N,N*-dimethylformamide (1.5 mL) heated to 100 °C was added diiodomethane (18 µL, 0.23 mmol, 1.1 equiv) and potassium carbonate (75 mg, 0.54 mmol, 2.5 equiv). After 2 h, the mixture was allowed to cool to 22 °C. Ethyl acetate (5 mL) was added and the mixture was washed with a 10% aqueous lithium chloride solution (3 × 5 mL). The organic solution was dried over sodium sulfate and filtered. The filtrate was concentrated under reduced pressure and the residue was purified by flash chromatography on silica gel (20% ethyl acetate in petroleum ether) which afforded benzodioxane **SI-29** as an 8:1 mixture of *trans*- and *cis*-isomers (colorless oil, 35 mg, 0.11 mmol, 51%).

Note: Compound **SI-29** was obtained as an 8:1 mixture of *trans* and *cis* isomers respectively. Only the NMR signals of the major *trans*-isomer are reported.

^{1}H NMR (400 MHz, $CDCl_3$): *δ* 6.94 – 6.90 (m, 2H), 6.88 (d, *J* = 8.8 Hz, 1H), 6.86 – 6.83 (m, 1H), 6.55 (d, *J* = 2.9 Hz, 1H), 6.48 (dd, *J* = 8.8, 2.9 Hz, 1H), 5.99 (s, 2H), 4.93 (d, *J* = 8.2 Hz, 1H), 3.95 (ddd, *J* = 8.2, 4.1, 2.7 Hz, 1H), 3.83 – 3.71 (m, 4H), 3.54 (ddd, *J* = 12.2, 8.0, 4.1 Hz, 1H), 1.98 (br s, 1H) ppm.

The analytical data matched those reported previously.[29]

Synthesis of benzodioxanes 48a

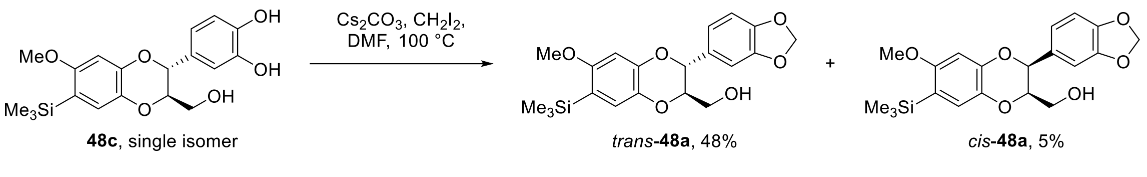

To a stirred solution of benzodioxane **48c** (42.0 mg, 112 µmol, 1 equiv) in *N,N*-dimethylformamide (1.0 mL) heated to 100 °C was added diiodomethane (18 µL, 0.22 mmol, 2.0 equiv) and cesium carbonate (73 mg, 0.22 mmol, 2.0 equiv). After 1 h, the mixture was allowed to cool to 22 °C. A saturated aqueous ammonium chloride solution (5 mL) was added and the mixture was extracted with ethyl acetate (2 × 5 mL). The organic solution was washed with a 10% aqueous lithium chloride solution (3 × 5 mL). The organic solution was dried over sodium sulfate and filtered. The filtrate was concentrated under reduced pressure and the residue was purified by flash chromatography on silica gel (10% ethyl acetate in petroleum ether grading

to 15% ethyl acetate in petroleum ether) which afforded benzodioxane *trans*-**48a** as a colorless oil (20.6 mg, 53.0 µmol, 48%) and benzodioxane *cis*-**48a** as a colorless oil (2.1 mg, 5.4 µmol, 5%).

Characterization data for isomer *trans*-**48a**:

TLC (20% ethyl acetate in cyclohexane): R_f = 0.42 (UV, $KMnO_4$).

^{1}H NMR (500 MHz, C_6D_6): δ 6.98 (s, 1H), 6.91 (d, J = 7.8 Hz, 2H), 6.88 – 6.82 (m, 1H), 6.50 (s, 1H), 6.00 (s, 2H), 4.94 (d, J = 8.2 Hz, 1H), 3.95 (ddd, J = 8.3, 4.0, 2.8 Hz, 1H), 3.79 (ddd, J = 12.3, 5.1, 2.7 Hz, 1H), 3.73 (s, 3H), 3.54 (ddd, J = 12.3, 8.1, 4.1 Hz, 1H), 2.02 – 1.94 (m, 1H), 0.25 (s, 9H) ppm.

^{13}C NMR (101 MHz, $CDCl_3$): δ 159.4, 148.4, 148.3, 145.1, 136.8, 130.1, 122.9, 121.5, 120.7, 108.7, 107.7, 101.5, 99.7, 78.0, 76.8, 61.9, 55.8, –0.8 ppm.

IR (ATR, neat): $\tilde{\nu}$ = 3496 (*w*), 2952 (*w*), 1607 (*w*), 1588 (*m*), 1490 (*m*), 1445 (*w*), 1402 (*w*), 1303 (*m*), 1248 (*m*), 1204 (*s*), 1151 (*w*), 1068 (*w*), 1039 (*m*), 935 (*w*), 907 (*w*), 839 (*s*), 760 (*w*) cm^{-1}.

HRMS (ESI): calcd. for $C_{20}H_{24}NaO_6Si$ $[M+Na]^+$: 411.1234; found: 411.1232.

Characterization data for isomer *cis*-**48a**:

TLC (20% ethyl acetate in cyclohexane): R_f = 0.34 (UV, $KMnO_4$).

^{1}H NMR (0 MHz, $CDCl_3$): δ 6.97 (d, J = 1.6 Hz, 1H), 6.90 – 6.82 (m, 3H), 6.47 (s, 1H), 5.98 (s, 2H), 5.20 (d, J = 2.9 Hz, 1H), 4.44 (dt, J = 8.8, 3.3 Hz, 1H), 3.75 – 3.67 (m, 4H), 3.49 (ddd, J = 12.3, 9.1, 3.8 Hz, 1H), 1.68 (dd, J = 9.1, 3.6 Hz, 1H), 0.25 (s, 9H) ppm.

^{13}C NMR (101 MHz, $CDCl_3$): δ 159.6, 148.3, 147.8, 144.6, 135.0, 130.0, 123.5, 121.1, 120.0, 108.6, 106.9, 101.4, 99.8, 77.1, 75.8, 59.5, 55.8, –0.8 ppm.

IR (ATR, neat): $\tilde{\nu}$ = 3427 (*w*), 2952 (*w*), 2897 (*w*), 1605 (*w*), 1586 (*m*), 1482 (*m*), 1445 (*m*), 1398 (*w*), 1301 (*w*), 1243 (*m*), 1203 (*s*), 1149 (*w*), 1040 (*m*), 1002 (*w*), 934 (*w*), 839 (*s*), 760 (*w*), 733 (*w*) cm^{-1}.

HRMS (ESI): calcd. for $C_{20}H_{24}NaO_6Si$ $[M+Na]^+$: 411.1234; found: 411.1232.

Synthesis of benzodioxane 51

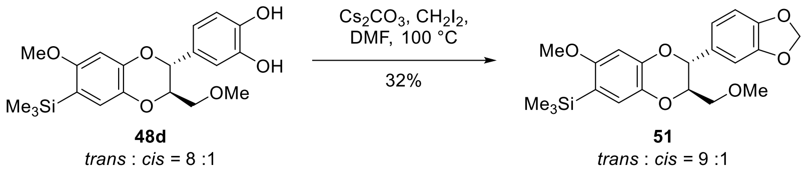

To a stirred solution of benzodioxane **48d** (20 mg, 51 µmol, 1 equiv) in *N,N*-dimethylformamide (0.3 mL) heated to 100 °C was added diiodomethane (10 µL, 0.13 mmol, 2.5 equiv) and cesium carbonate (33 mg, 0.10 mmol, 2.0 equiv). After 1 h, the mixture was allowed to cool to 22 °C. A saturated aqueous ammonium chloride solution (2 mL) was added and the mixture was extracted with ethyl acetate (2 × 2 mL). The organic solution was washed with a 10% aqueous lithium chloride solution (3 × 2 mL). The organic solution was dried over sodium sulfate and filtered. The filtrate was concentrated under reduced pressure and the residue was purified by flash chromatography on silica gel (5% ethyl acetate in petroleum ether) which afforded benzodioxane **51** as a 9:1 mixture of *trans*- and *cis*-isomers respectively (colorless oil, 5.8 mg, 14 µmol, 32%).

TLC (10% ethyl acetate in cyclohexane): R_f = 0.66 (UV, $KMnO_4$).

Note: Compound **51** was obtained as a 9:1 mixture of *trans* and *cis* isomers respectively. Only the NMR signals of the major *trans*-isomer are reported.

^{1}H NMR (500 MHz, C_6D_6): δ 7.34 (s, 1H), 6.94 (d, *J* = 1.6 Hz, 1H), 6.77 (dd, *J* = 8.0, 1.7 Hz, 1H), 6.62 (d, *J* = 8.0 Hz, 1H), 6.52 (s, 1H), 5.27 (s, 2H), 5.05 (d, *J* = 7.8 Hz, 1H), 3.88 (ddd, *J* = 7.8, 3.7, 2.5 Hz, 1H), 3.36 (dd, *J* = 11.0, 2.5 Hz, 1H), 3.27 (s, 3H), 3.14 (dd, *J* = 11.0, 3.8 Hz, 1H), 3.02 (s, 3H), 0.39 (s, 9H) ppm.

^{13}C NMR (126 MHz, C_6D_6): δ 159.6, 148.43, 148.37, 145.9, 137.9, 131.5, 123.7, 121.7, 120.3, 108.5, 108.3, 101.2, 100.0, 77.8, 76.9, 71.7, 59.1, 55.3, –0.5 ppm.

IR (ATR, neat): $\tilde{\nu}$ = 2951 (*w*), 2926 (*m*), 2857 (*w*), 1607 (*w*), 1588 (*m*), 1504 (*w*), 1489 (*m*), 1464 (*w*), 1445 (*w*), 1406 (*w*), 1305 (*m*), 1273 (*w*), 1249 (*m*), 1206 (*s*), 1151 (*w*), 1095 (*w*), 1066 (*m*), 1035 (*m*), 1002 (*w*), 937 (*w*), 922 (*w*), 840 (*s*) cm^{-1}.

HRMS (ESI): calcd. for $C_{21}H_{26}NaO_6Si$ $[M+Na]^+$: 425.1391; found: 425.1384.

Synthesis of benzodioxane 53

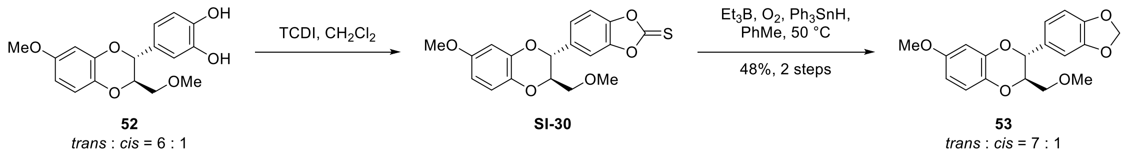

To a solution of benzodioxane **52** (193 mg, 606 µmol, 1 equiv) in dichloromethane (3 mL) was added thiocarbonyldiimidazole (162 mg, 909 µmol, 1.50 equiv) and the mixture was stirred for 3 hours. The solution was diluted with dichloromethane (10 mL) and washed with an aqueous hydrogen chloride solution (2 × 15 mL). The aqueous layers were combined and extracted with dichloromethane (15 mL). The combined organic layers were dried over sodium sulfate and concentrated under reduced pressure which afforded thiocarbonate **SI-30** (190 mg) that was used without further purification.

To a solution of crude thiocarbonate **SI-30** (40 mg, assumed 0.11 mmol, 1 equiv) and triphenylstannane (0.12 g, 0.33 mmol, 3.0 equiv) in toluene (1 mL) was added a solution of triethylborane (1.0 M in hexane, 0.11 mL, 0.11 mmol, 1.0 equiv) at 22 °C. The resulting solution was heated to 50 °C and oxygen gas (1 mL) was added via syringe pump over 3 h. After the addition of oxygen was done, the reaction mixture was allowed to cool to 22 °C. The resulting suspension was concentrated under reduced pressure and the residue was purified by flash chromatography on silica gel (5% ethyl acetate in petroleum ether) which afforded benzodioxane **53** as a 7:1 mixture of *trans* and *cis* isomers respectively (colorless oil, 20 mg, 61 µmol, 48% over 2 steps).

TLC (10% ethyl acetate in cyclohexane): R_f = 0.50 (UV, $KMnO_4$).

Note: Compound **53** was obtained as a 7:1 mixture of *trans* and *cis* isomers respectively. Only the NMR signals of the major *trans*-isomer are reported.

^{1}H NMR (500 MHz, $CDCl_3$): δ 6.93 – 6.88 (m, 3H), 6.86 – 6.83 (m, 1H), 6.53 (d, *J* = 2.9 Hz, 1H), 6.46 (dd, *J* = 8.9, 2.9 Hz, 1H), 5.99 (s, 2H), 4.98 (d, *J* = 8.1 Hz, 1H), 4.00 (ddd, *J* = 8.1, 4.1, 2.3 Hz, 1H), 3.74 (d, *J* = 9.3 Hz, 3H), 3.57 (dd, *J* = 10.8, 2.4 Hz, 1H), 3.37 – 3.28 (m, 4H) ppm.

^{13}C NMR (126 MHz, $CDCl_3$): δ 154.4, 148.23, 148.21, 144.1, 137.5, 130.6, 121.4, 117.6, 108.6, 107.72, 107.65, 102.5, 101.4, 77.3, 76.6, 71.4, 59.6, 55.8 ppm.

IR (ATR, neat): $\tilde{\nu}$ = 2895 (*w*), 1599 (*w*), 1503 (*s*), 1467 (*w*), 1444 (*m*), 1385 (*w*), 1319 (*w*), 1293 (*w*), 1250 (*m*), 1241 (*w*), 1214 (*m*), 1197 (*w*), 1157 (*m*), 1123 (*w*), 1107 (*w*), 1070 (*w*), 1036 (*s*), 932 (*w*), 916 (*w*), 885 (*w*), 830 (*w*), 808 (*m*), 743 (*w*), 730 (*w*) cm^{-1}.

HRMS (ESI): calcd. for $C_{18}H_{18}NaO_6$ $[M+Na]^+$: 353.0996; found: 353.0990.

Determination of 1,4-benzodioxane isomers

To determine the identity of a particular 1,4-benzodioxane isomer, generated in the formal [4+2] cycloaddition model studies, it was not possible to use 2D NMR data due to the lack of appropriate HMBC correlations. The isomers could be easily differentiated based on the pattern observed in their ^{1}H NMR spectra. This is exemplified by isomers *trans*-**48a**, *cis*-**48a** and *trans*-**49a** (Figure 1). The difference of chemical shifts of the 1,4-benzodioxane C2 and C3 proton signals in *trans*-**48a** is significantly larger than that of the chemical shifts of the C2 and C3 proton signals in *trans*-**49a**. Both chemical shifts of the C2 and C3 proton signals in *cis*-**48a** are higher than in the case of the *trans*-isomers, additionally, the C2 proton experiences a smaller *J*-coupling which distinguishes the signal of *cis*-**48a** even further. This pattern was observed for all 1,4-benzodioxanes generated in the model studies regardless of the substitution pattern on the eastern aromatic ring. The chemical shifts of the C2 and C3 proton signals of obtained 1,4-benzodioxane isomers have been summarized in Table 2. The table was used to determine the identity of the respective isomer for compounds **SI-29**, **SI-30** and **SI-32** of which only one isomer was isolated and characterized.

Figure 1: Comparison of ^{1}H NMR spectra of isomers *trans*-48a, *cis*-48a and 49a

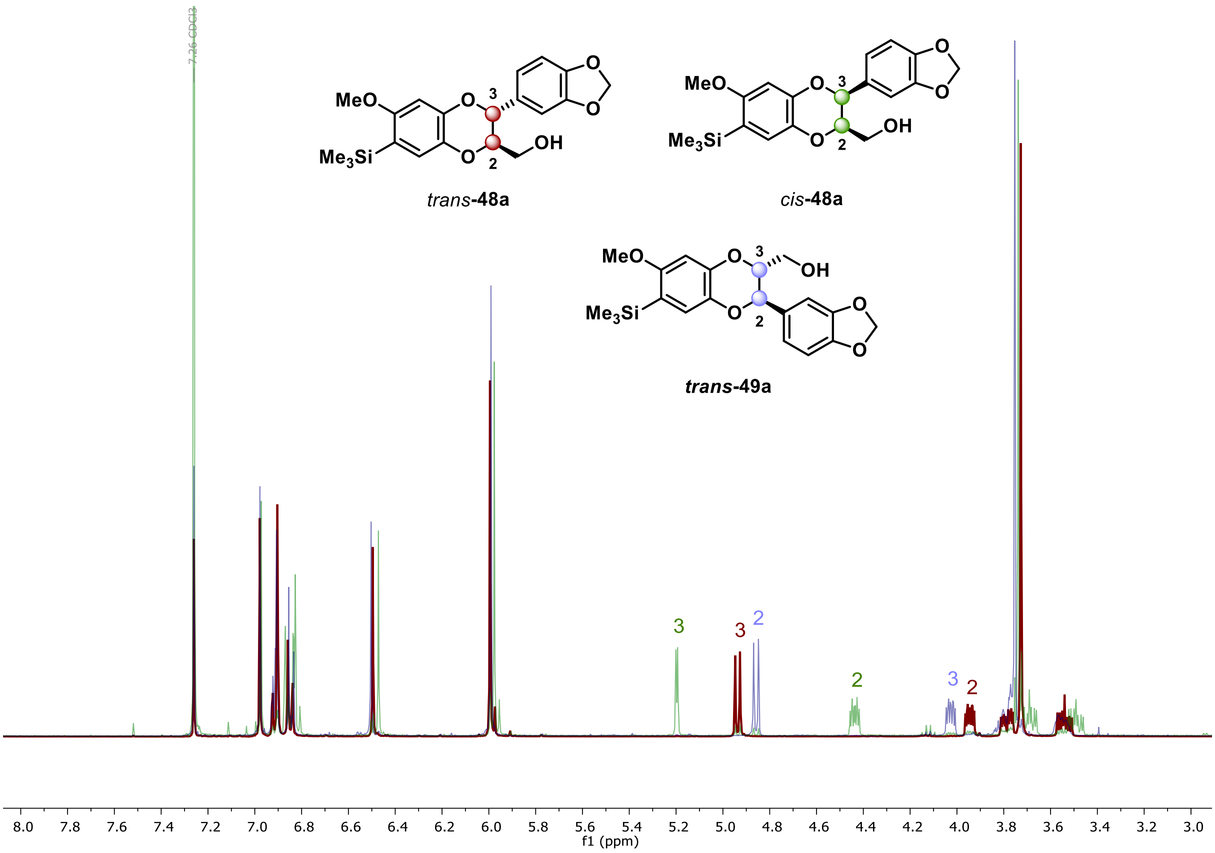

Table 2: Summary of chemical shifts of the C2 and C3 proton signals of obtained 1,4-benzodioxane *trans*-isomers.

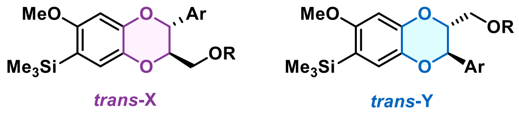

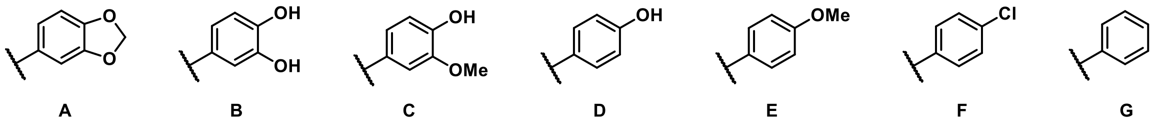

δ in $CDCl_3$ (ppm)					
R = H	R = Me	Benzylic H	Non-benzylic H	Δ	Isomer
**48a** (A)		4,95	3,95	1,00	**X**
**49a** (A)		4,86	4,03	0,83	**Y**
	**51a** (A)	4,97	4,00	0,97	**X**
	**51b** (A)[b]	4,87	4,07	0,80	**Y**
**SI-22a** (C)		4,94	3,97	0,97	**X**
**SI-22b** (C)		4,86	4,06	0,80	**Y**
	**48d** (B)	4,84	3,99	0,85	**X**
	**49d** (B)	4,75	4,07	0,68	**Y**
**SI-25** (F)[a]		4,97	4,01	0,96	**X**
**SI-26** (G)[a]		4,95	4,08	0,87	**Y**
**SI-28** (E)[a]		4,89	4,06	0,83	**Y**

δ in CD_3OD (ppm)				
	Benzylic	Non-benzylic	Δ	Isomer
**48c** (B)	4,80	3,91	0,89	**X**
**49c** (B)[b]	4,72	3,99	0,73	**Y**
**48a** (A)	4,90	3,92	0,98	**X**
**49a** (A)	4,82	3,98	0,84	**Y**
**SI-22a** (C)	4,88	3,95	0,93	**X**
**SI-22b** (C)	4,80	4,03	0,77	**Y**
**SI-23a** (D)	4,89	3,94	0,95	**X**
**SI-23b** (D)	4,80	4,02	0,78	**Y**
**SI-25** (F)[a]	4,96	4,03	0,93	**X**

[a] Only one isomer was observed. The identity of the isomer was determined by comparison of the Δ value to Δ values of the other 1,4-benzodioxane compounds. [b] This compound was only observed in a crude ^1H NMR but never isolated.

Synthesis of phenols:

Synthesis of phenol 28

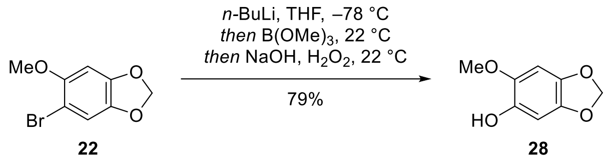

To a solution of aryl bromide **22** (3.00 g, 13.0 mmol, 1 equiv) in tetrahydrofuran (20 mL) cooled to –78 °C was added a solution of *n*-butyllithium (2.50 M in hexane, 6.23 mL, 15.6 mmol, 1.20 equiv) dropwise over 5 min. After stirring at –78 °C for 5 min, trimethyl borate (1.88 mL, 16.9 mmol, 1.30 equiv) was added dropwise over 5 min. The resulting solution was allowed to warm to 22 °C. After 30 min, the solution was cooled to 0 °C and an aqueous solution of sodium hydroxide (2.00 M, 7.79 mL, 15.6 mmol, 1.20 equiv) and an aqueous solution of hydrogen peroxide (30 wt% of H_2O_2, 1.59 mL, 15.6 mmol, 1.20 equiv) were added. The solution was allowed to warm to 22 °C and stirred for 30 min. Aqueous solution of hydrogen chloride (1 M, 30 mL) was added and the mixture was extracted with ethyl acetate (2 × 20 mL). The combined organic solutions were washed with a saturated aqueous sodium chloride solution (20 mL). The washed solution was dried over sodium sulfate and filtered. The filtrate was concentrated under reduced pressure and the resulting residue was purified by flash chromatography on silica gel (20% ethyl acetate in petroleum ether) which afforded phenol **28** as a pale-brown oil (1.72 g, 10.2 mmol, 79%).

^{1}H NMR (400 MHz, $CDCl_3$): δ 6.56 – 6.54 (m, 2H), 5.86 (s, 2H), 3.83 (s, 3H) ppm.

The analytical data matched those reported previously.[30]

Synthesis of aldehyde SI-31

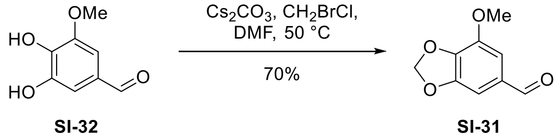

To a suspension of catechol **SI-32** (1.00 g, 5.95 mmol, 1 equiv) and cesium carbonate (3.88 g, 11.9 mmol, 2.00 equiv) in *N,N*-dimethylformamide (20 mL) heated to 50 °C was added bromochloromethane (1.16 mL, 17.8 mmol, 3.00 equiv) and the resulting suspension was stirred for 6 h. The suspension was allowed to cool to 22 °C. An aqueous hydrogen chloride solution (2 M, 20 mL) and ethyl acetate (50 mL) were added. The aqueous solution was discarded and the organic solution was washed with a saturated aqueous solution of lithium chloride (2 × 50 mL). The organic solution was dried over sodium sulfate and filtered. The filtrate was concentrated under reduced pressure which afforded aldehyde **SI-31** as a brown solid (753 mg, 4.18 mmol, 70%) that was used without further purification in the next step.

^{1}H NMR (400 MHz, $CDCl_3$): δ 9.79 (s, 1H), 7.14 (d, J = 1.3 Hz, 1H), 7.06 (d, J = 1.3 Hz, 1H), 6.10 (s, 2H), 3.97 (s, 3H) ppm.

The analytical data matched those reported previously.[31]

Synthesis of phenol SI-33

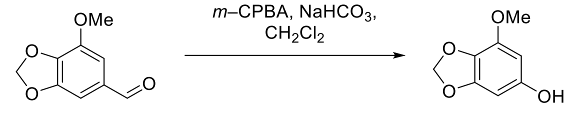

To a solution of aldehyde **SI-31** (1.00 g, 5.55 mmol, 1 equiv) in dichloromethane (12 mL) cooled to 0 °C was added sodium bicarbonate (933 mg, 11.1 mmol, 2.00 equiv) followed by 3-chloroperoxobenzoic acid (77 wt%, 1.75 g, 7.83 mmol, 1.41 equiv). The mixture was allowed to warm to 22 °C. After 2 h, an aqueous solution of sodium hydroxide (2 M, 20 mL) was added and the resulting mixture was vigorously stirred for 16 h. The solution was acidified with an aqueous solution of hydrogen chloride (2 M, 50 mL) and the mixture was extracted with dichloromethane (2 × 50 mL). The organic solution was washed with a saturated aqueous sodium chloride solution (50 mL). The washed solution was dried over sodium sulfate and filtered. The filtrate was concentrated under reduced pressure and the residue was used in the next step without further purification.

Note: An analytic sample of the intermediate phenol was obtained as follows. The crude residue was dissolved in a 2 M aqueous solution of sodium hydroxide and the resulting dark-brown solution was carefully acidified with a 2 M aqueous solution of hydrogen chloride until pH = 7. The resulting light-brown solution was extracted twice with dichloromethane and the organic solution was dried over sodium sulfate, filtered and the filtrate was concentrated under reduced pressure which afforded pure phenol **SI-33**.

^{1}H NMR (400 MHz, $CDCl_3$): δ 6.10 (d, J = 2.3 Hz, 1H), 6.03 (d, J = 2.3 Hz, 1H), 5.90 (s, 2H), 4.58 (br s, 1H), 3.86 (s, 3H) ppm.

The analytical data matched those reported previously.[31]

Synthesis of benzodioxole SI-34

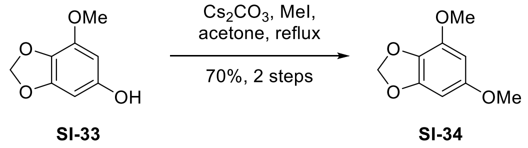

To a solution of crude phenol **SI-33** (assumed 5.55 mmol, 1 equiv) in acetone (10 mL) was added cesium carbonate (3.62 g, 11.1 mmol, 2.00 equiv) and iodomethane (1.77 mL, 27.8 mmol, 5.00 equiv) and the resulting mixture was refluxed for 6 h. The mixture was concentrated under reduced pressure. To the residue was added an aqueous solution of hydrogen chloride (2 M, 20 mL) and the mixture was extracted with ethyl acetate (2 × 20 mL). The organic solution was washed with a saturated aqueous sodium chloride solution (20 mL). The washed solution was dried over sodium sulfate and filtered. The filtrate was concentrated under reduced pressure and the resulting residue was purified by flash chromatography on silica gel (5% ethyl acetate in petroleum ether) which afforded benzodioxole **SI-34** as a pale-yellow solid (706 mg, 3.88 mmol, 70% over 2 steps).

^{1}H NMR (400 MHz, $CDCl_3$): δ 6.18 (d, J = 2.3 Hz, 1H), 6.09 (d, J = 2.3 Hz, 1H), 5.91 (s, 2H), 3.88 (s, 3H), 3.74 (s, 3H) ppm.

The analytical data matched those reported previously.[31]

Synthesis of aryl bromide SI-35

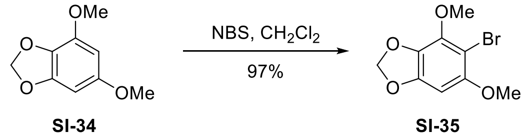

To a solution of benzodioxole **SI-34** (700 mg, 3.84 mmol, 1 equiv) in dichloromethane (15 mL) cooled to 0 °C was added *N*-bromosuccinimide (718 mg, 4.03 mmol, 1.05 equiv) and the resulting solution was allowed to warm to 22 °C. After 30 min, the solution was diluted with dichloromethane (15 mL) and washed with water (3 × 30 mL). The washed solution was dried over sodium sulfate and filtered. The filtrate was concentrated under reduced pressure which afforded aryl bromide **SI-35** as a pale-brown solid (971 mg, 3.72 mmol, 97%) that was used in the next step without further purification.

^{1}H NMR (400 MHz, $CDCl_3$): δ 6.31 (s, 1H), 5.92 (s, 2H) 4.05 (s, 3H), 3.82 (s, 3H) ppm.

The analytical data matched those reported previously.[31]

Synthesis of phenol 60

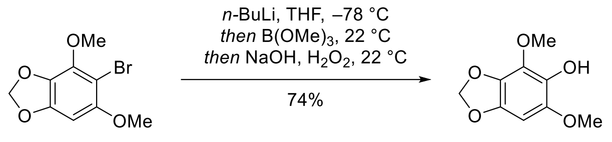

To a solution of aryl bromide **SI-35** (1.50 g, 5.75 mmol, 1 equiv) in tetrahydrofuran (10 mL) cooled to –78 °C was added a solution of *n*-butyllithium (2.50 M in hexane, 2.76 mL, 6.89 mmol, 1.20 equiv) dropwise over 5 min. After stirring at –78 °C for 5 min, trimethyl borate (961 µL, 8.62 mmol, 1.50 equiv) was added dropwise over 5 min. The resulting solution was allowed to warm to 22 °C. After 30 min, the solution was cooled to 0 °C and an aqueous solution of sodium hydroxide (2.00 M, 3.45 mL, 6.90 mmol, 1.20 equiv) and an aqueous solution of hydrogen peroxide (30 wt% of H_2O_2, 704 µL, 6.89 mmol, 1.20 equiv) were added. The solution was allowed to warm to 22 °C and stirred for 30 min. Aqueous solution of hydrogen chloride (1.00 M, 20 mL) was added and the mixture was extracted with ethyl acetate (2 × 20 mL). The combined organic solutions were washed with a saturated aqueous sodium chloride solution (20 mL). The washed solution was dried over sodium sulfate and filtered. The filtrate was concentrated under reduced pressure and the resulting residue was purified by flash chromatography on silica gel (30% ethyl acetate in petroleum ether) which afforded phenol **60** as a pale-brown oil (840 mg, 4.24 mmol, 74%).

^{1}H NMR (400 MHz, $CDCl_3$): δ 6.30 (s, 1H), 5.93 (s, 2H), 5.21 (s, 1H), 4.05 (s, 3H), 3.82 (s, 3H) ppm.

The analytical data matched those reported previously.[32]

Synthesis of phenol SI-36

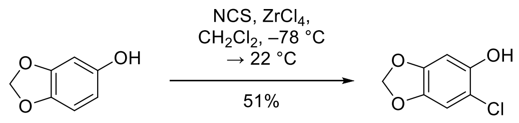

To a suspension of *N*-chlorosuccinimide (96.7 mg, 724 µmol, 1.00 equiv) in 3 ml of dichloromethane cooled to –78°C were added phenol **23** (100 mg, 724 µmol, 1 equiv) and zirconium tetrachloride (33.7 mg, 145 µmol, 0.200 equiv) and the mixture was allowed to warm to 22 °C. After 3 hours, a saturated sodium bicarbonate aqueous solution (5 mL) was added to the crude and the layers were separated. The aqueous layer was extracted with dichloromethane (3 × 10 mL). The organic layers were combined, dried over sodium sulfate and concentrated under reduced pressure. The resulting solid was then dissolved in 10 mL of diethyl ether and then washed with water (3 × 5 mL). The organic layer was dried over sodium sulfate and concentrated under reduced pressure. Phenol **SI-36** was afforded as a white solid (64 mg, 0.37 µmol, 51 %).

[1]**H NMR** (400 MHz, $CDCl_3$): δ 6.77 (s, 1H), 6.57 (s, 1H), 5.92 (s, 2H), 5.27 (br s, 1H) ppm.

The analytical data matched those reported previously.[33]

Synthesis of ether SI-37

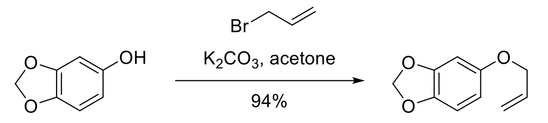

23 SI-37

To a solution of sesamol **23** (500 mg, 3.62 mmol, 1 equiv) in acetone (2.5 mL) were added potassium carbonate (1.00 g, 7.24 mmol, 2.00 equiv) and allyl bromide (376 µL, 4.34 mmol, 1.20 equiv) and the resulting suspension was stirred for 48 h. The suspension was filtered and the solvent was evaporated under reduced pressure. The residue was purified by flash chromatography on silica gel (5% ethyl acetate in petroleum ether) which afforded ether **SI-37** as a colorless liquid (603 mg, 3.38 mmol, 94%).

[1]**H NMR** (400 MHz, $CDCl_3$): δ 6.70 (d, J = 8.5 Hz, 1H), 6.51 (d, J = 2.5 Hz, 1H), 6.34 (dd, J = 8.5, 2.5 Hz, 1H), 6.03 (ddt, J = 17.4, 10.5, 5.3 Hz, 1H), 5.91 (s, 2H), 5.39 (dq, J = 17.2, 1.6 Hz, 1H), 5.27 (dt, J = 10.5, 1.5 Hz, 1H), 4.46 (dt, J = 5.4, 1.6 Hz, 2H) ppm.

The analytical data matched those reported previously.[34]

Synthesis of phenol SI-38

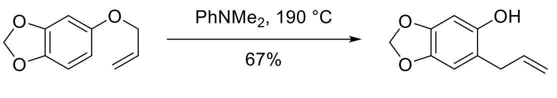

SI-37 SI-38

A solution of ether **SI-37** (40 mg, 0.22 mmol, 1 equiv) in *N,N*-dimethylaniline (1 mL) was stirred at 190°C for 20 h. The solution was allowed to cool to 22 °C. An aqueous 2 M solution of hydrogen chloride (5 mL) was added and the mixture was extracted with ethyl acetate (3 × 10 mL). The organic layers were combined, dried over anhydrous sodium sulfate and then concentrated under reduced pressure. The residue was purified by flash chromatography on silica gel (8% ethyl acetate in petroleum ether) which afforded phenol **SI-38** as a white solid (27 mg, 0.15 mmol, 67%).

[1]**H NMR** (400 MHz, $CDCl_3$): δ 6.58 (s, 1H), 6.43 (s, 1H), 6.03 – 5.91 (m, 1H), 5.88 (s, 2H), 5.19 – 5.15 (m, 1H), 5.15 – 5.11 (m, 1H), 4.68 (s, 1H), 3.31 (dt, J = 6.3, 1.6 Hz, 2H) ppm.

The analytical data matched those reported previously.[34]

Synthesis of phenol SI-39

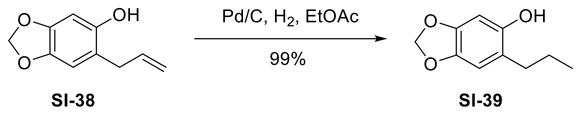

To a solution of phenol **SI-38** (100 mg, 561 µmol, 1 equiv) in ethyl acetate (5 mL) was added palladium on carbon (59.7 mg, 10 wt% Pd, 56.1 µmol, 10 mol%) and the mixture was sparged with hydrogen gas for 5 min. The mixture was then stirred under hydrogen atmosphere for 1 h. The suspension was filtered over a pad of celite and the filtered solution was concentrated under reduced pressure which afforded phenol **SI-43** as pink solid (100 mg, 555 µmol, 98.9 %) that was used without further purification.

^{1}H NMR (400 MHz, $CDCl_3$): δ 6.59 (s, 1H), 6.39 (s, 1H), 5.87 (s, 2H), 4.42 (s, 1H), 2.52 – 2.43 (m, 2H), 1.63 – 1.54 (m, 2H), 0.96 (t, J = 7.3 Hz, 3H) ppm.

The analytical data matched those reported previously.[35]

Synthesis of quinone SI-40

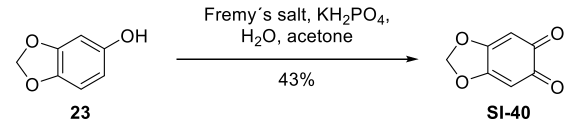

To a solution of sesamol **23** (1.00 g, 7.24 mmol, 1 equiv) in acetone (40 mL) and water (10 mL) was added potassium dihydrogen phosphate (5.91 g, 43.4 mmol, 6.00 equiv) followed by Fremy´s salt (3.90 g, 14.5 mmol, 2.00 equiv). The resulting suspension was stirred for 4 h. Ethyl acetate (100 mL) was added and the mixture was washed with a saturated aqueous sodium chloride solution (2 × 100 mL). The organic solution was dried over sodium chloride and filtered. The filtrate was concentrated under reduced pressure which afforded quinone **SI-40** as a brown solid (470 mg, 3.09 mmol, 43%) that was used without further purification.

^{1}H NMR (400 MHz, $CDCl_3$): δ 6.10 (s, 2H), 6.04 (s, 2H) ppm.

The analytical data matched those reported previously.[36]

Synthesis of catechol SI-41

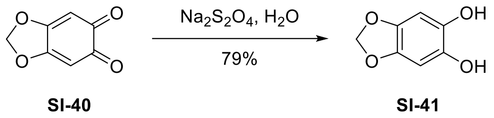

A mixture of quinone **SI-40** (224 mg, 1.47 mmol, 1 equiv) and sodium dithionite (577 mg, 3.31 mmol, 2.25 equiv) in acetonitrile (14 mL) and water (6 mL) was stirred for 30 min. An aqueous hydrogen chloride solution (2 M, 20 mL) was added. The solution was extracted with ethyl acetate (3 × 20 mL). The combined organic layers were dried over anhydrous sodium sulfate and concentrated under reduced pressure which afforded catechol **SI-41** as a pink solid (178 mg, 1.16 mmol, 79%).

^{1}H NMR (500 MHz, $CDCl_3$): δ 6.50 (s, 2H), 5.85 (s, 2H), 4.76 (s, 2H) ppm.

The analytical data matched those reported previously.[37]

Synthesis of phenol SI-42

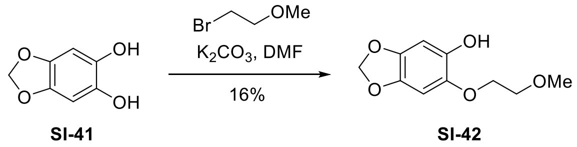

To a solution of catechol **SI-41** (175 mg, 1.14 mmol, 1 equiv) in *N,N*-dimethylformamide (3.5 mL) was added potassium carbonate (314 mg, 2.27 mmol, 2.00 equiv) followed by 2-bromoethylmethyl ether (108 µL, 1.14 mmol, 1.00 equiv). The suspension was stirred at 60°C for 16 h. The resulting solution was then carefully poured into an aqueous solution of hydrochloric acid (2 M, 10 mL). The mixture was extracted with ethyl acetate (3 × 10 mL). The organic layers were combined and washed with an aqueous 10% lithium chloride solution (3 × 10 mL). The organic layer was then dried over sodium sulfate and filtered. The filtrate was concentrated under reduced pressure and the residue was purified by flash chromatography on silica gel (30% ethyl acetate in petroleum ether) which afforded phenol **SI-42** is afforded as a beige solid (40.0 mg, 189 µmol, 16%).

TLC (30% ethyl acetate in cyclohexane): R_f = 0.31 (UV, $KMnO_4$).

^{1}H NMR (400 MHz, $CDCl_3$): δ 6.68 (s, 1H), 6.57 (s, 1H), 6.52 (s, 1H), 5.86 (s, 2H), 4.09 – 4.04 (m, 2H), 3.63 (dd, J = 5.3, 3.6 Hz, 2H), 3.47 (s, 3H) ppm.

^{13}C NMR (101 MHz, $CDCl_3$): δ 143.5, 143.3, 140.1, 139.3, 101.1, 100.8, 98.1, 72.7, 71.0, 59.2 ppm.

IR (ATR, neat): $\tilde{\nu}$ = 3360 (*w*), 2925 (*w*), 2889 (*w*), 1649 (*w*), 1504 (*w*), 1482 (*s*), 1455 (*w*), 1361 (*w*), 1333 (*w*), 1287 (*w*), 1198 (*w*), 1153 (*s*), 1123 (*w*), 1102 (*w*), 1077 (*w*), 1031 (*s*), 933 (*m*), 900 (*w*), 853 (*s*), 759 (*w*), 629 (*w*) cm^{-1}.

HRMS (ESI): calcd. for $C_{10}H_{12}NaO_5$ $[M+Na]^+$: 235.0577; found: 235.0574.

Synthesis of phenol SI-43

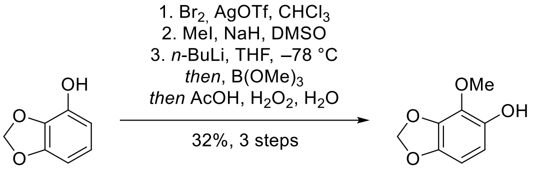

Bromine (373 µL, 7.24 mmol, 1.00 equiv) was added to a stirred mixture of phenol **SI-44** (1.00 g, 7.24 mmol, 1 equiv) and silver(I) trifluoroacetate (1.60 g, 7.24 mmol, 1.00 equiv) in chloroform (25 mL) at 0 °C and the mixture was allowed to warm to 22 °C. After 1 h, a saturated aqueous sodium thiosulfate solution (50 mL) was added. The phases were separated, and the aqueous layer was extracted with chloroform (3 × 50 mL). The combined organic phases were dried over sodium sulfate filtered. The filtrate was concentrated under reduced pressure and the residue was purified by flash chromatography (15% ethyl acetate in petroleum ether) which afforded the monobrominated phenol contaminated with the inseparable dibrominated phenol.

Sodium hydride (60 wt% in mineral oil, 284 mg, 7.10 mmol, 1.10 equiv) was added to a solution of mono- and dibrominated phenols (1.40 g, assumed 6.45 mmol of monobrominated phenol, 1 equiv) in dimethylsulfoxide (20 mL). After 30 min, methyl iodide (484 µL, 7.74 mmol, 1.20 equiv) was added. After 1 h, water (40 mL) was added and the mixture was extracted with diethyl ether (3 × 40 mL). The combined organic phases were washed with a saturated aqueous sodium chloride solution (20 mL), dried over magnesium sulfate and filtered. The filtrate was concentrated under reduced pressure. The residue was purified by flash chromatography (2.5% diethyl ether in petroleum ether) which afforded the monobrominated arene contaminated with the inseparable dibrominated arene.

To a solution of the mono- and dibrominated arenes (700 mg, assumed 3.03 mmol of monobrominated arene, 1 equiv) in tetrahydrofuran (12 mL) cooled to –78 °C was added *n*-butyllithium (2.08 mL, 1.60 molar, 3.33 mmol, 1.10 equiv) and the reaction was stirred at –78 °C. After 30 min, trimethyl borate (413 µL, 3.64 mmol, 1.20 equiv) was added and the reaction was allowed to warm to 22 °C. After 2 h, acetic acid (867 µL, 15.1 mmol, 5.00 equiv) and aqueous hydrogen peroxide (1.55 mL, 30 wt%, 15.1 mmol, 5.00 equiv) were added successively. After 2 h, water (30 mL) was added and the mixture was extracted with diethyl ether (3 × 50 mL). The organic layers were combined and washed with a saturated aqueous sodium sulfite solution (50 mL) and saturated aqueous sodium chloride solution (50 mL). The organic layer was dried over

sodium sulfate and filtered. The filtrate was concentrated under reduced pressure and the residue was purified by flash chromatography on silica gel (15% ethyl acetate in petroleum ether) which afforded phenol **SI-43** as a white solid (205 mg, 1.22 mmol, 32% over 3 steps).

^{1}H NMR (400 MHz, $CDCl_3$): *δ* 6.41 (d, *J* = 8.4 Hz, 1H), 6.38 (d, *J* = 8.4 Hz, 1H), 5.89 (s, 2H), 5.29 (s, 1H), 4.05 (s, 3H) ppm.

The analytical data matched those reported previously.[38]

Synthesis of phenol SI-45

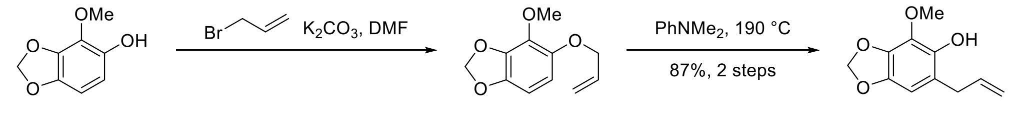

To a suspension of phenol **SI-43** (100 mg, 595 µmol, 1 equiv) and potassium carbonate (164 mg, 1.19 mmol, 2.00 equiv) in acetone (2.5 mL) was added allyl bromide (86.3 mg, 61.8 µL, 714 µmol, 1.20 equiv) and the resulting suspension was stirred at 23°C overnight. The suspension was filtered and the filtrate was concentrated under reduced pressure. The residue containing ether product **SI-46** was used in the next step without further purification.

A solution of crude ether **SI-46** (assumed 595 µmol, 1 equiv) in *N*,*N*-dimethylaniline (2.5 mL) was stirred overnight at 190 °C. The solution was allowed to cool to 22 °C. An aqueous hydrogen chloride solution (2 M, 30 mL) was added and the mixture was extracted with ethyl acetate (3 × 50 mL). The organic layers were combined, dried over sodium sulfate and filtered. The filtrate was concentrated under reduced pressure and the residue was purified by flash chromatography on silica gel (10 % ethyl acetate in petroleum ether) which afforded phenol **SI-45** as a colorless oil (80.0 mg, 384 µmol, 65% over 2 steps).

^{1}H NMR (500 MHz, $CDCl_3$): *δ* 6.33 (s, 1H), 5.95 (ddt, *J* = 16.7, 10.0, 6.5 Hz, 1H), 5.85 (s, 2H), 5.39 (s, 1H), 5.09 – 5.02 (m, 2H), 4.04 (s, 3H), 3.31 (dt, *J* = 6.5, 1.6 Hz, 2H) ppm.

The analytical data matched those reported previously.[38]

Final stage of the total synthesis of haedoxans:

Synthesis of mixture of benzodioxanes 57 and benzodioxane 58

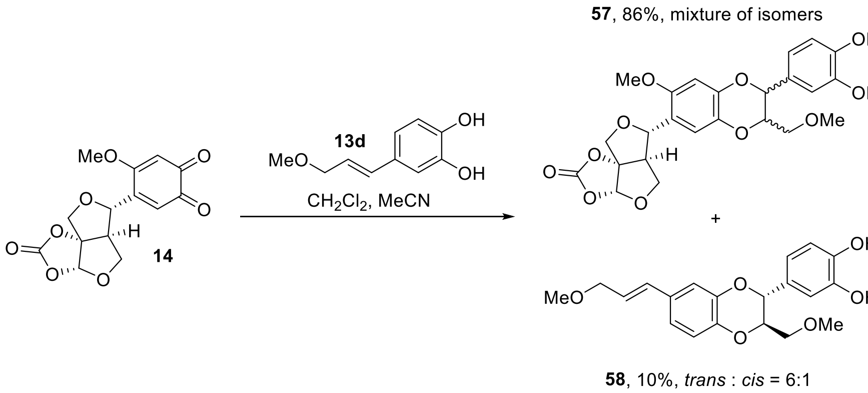

To a solution of quinone **14** (3.41 g, 11.1 mmol, 1 equiv) in a mixture of dichloromethane and acetonitrile (60 mL, 5:1) was added dropwise over 10 h a solution of catechol **13d** (2.19 g, 12.2 mmol, 1.10 equiv) in a mixture of dichloromethane and acetonitrile (60 mL, 5:1). After stirring for further 10 h, the mixture was concentrated under reduced pressure and the residue was purified by flash chromatography on silica gel (30% ethyl acetate and 20% dichloromethane in petroleum ether grading to 40% ethyl acetate and 20% dichloromethane in petroleum ether) which afforded benzodioxane **57** as a complex, inseparable mixture of isomers (brown solid, 4.63 g, 9.48 mmol, 86%) and benzodioxane **58** as a 6:1 mixture of *trans* and *cis* isomers (brown solid, 410 mg, 1.14 mmol, 10%).

Characterization data for benzodioxane **58**:

TLC (35% ethyl acetate in cyclohexane): R_f = 0.28 (UV, $KMnO_4$).

Note: Compound **58** was isolated as a 6:1 mixture of *trans* and *cis* isomers. Only the NMR signals of the major *trans*-isomer are reported.

^{1}H NMR (500 MHz, $CDCl_3$): δ 6.95 (d, J = 1.8 Hz, 1H), 6.91 – 6.80 (m, 5H), 6.49 (dt, J = 15.9, 1.4 Hz, 1H), 6.12 (ddt, J = 16.0, 10.5, 6.3 Hz, 3H), 4.87 (d, J = 8.0 Hz, 1H), 4.09 (td, J = 6.4, 1.4 Hz, 2H), 4.02 (ddd, J = 8.0, 4.3, 2.3 Hz, 1H), 3.53 (dd, J = 11.0, 2.4 Hz, 1H), 3.40 (d, J = 5.5 Hz, 3H), 3.35 – 3.28 (m, 4H) ppm.

^{13}C NMR (126 MHz, $CDCl_3$): δ 144.8, 144.2, 143.8, 143.3, 132.9, 130.5, 129.1, 123.8, 120.3, 120.3, 117.4, 115.6, 114.9, 114.4, 77.6, 76.2, 73.4, 71.3, 59.6, 57.9 ppm.

IR (ATR, neat): $\tilde{\nu}$ = 3296 (*br, m*), 2930 (*w*), 2895 (*w*), 2830 (*w*), 1611 (*w*), 1586 (*w*), 1506 (*s*), 1448 (*w*), 1365 (*w*), 1273 (*s*), 1196 (*w*), 1159 (*w*), 1114 (*m*), 1084 (*w*), 1064 (*w*), 1023 (*w*), 967 (*w*), 911 (*w*), 872 (*w*), 815 (*w*), 785 (*w*), 730 (*m*) cm^{-1}.

HRMS (ESI): calcd. for $C_{20}H_{22}NaO_6$ [M+Na]$^+$: 381.1309; found: 381.1303.

Synthesis of benzodioxole 59

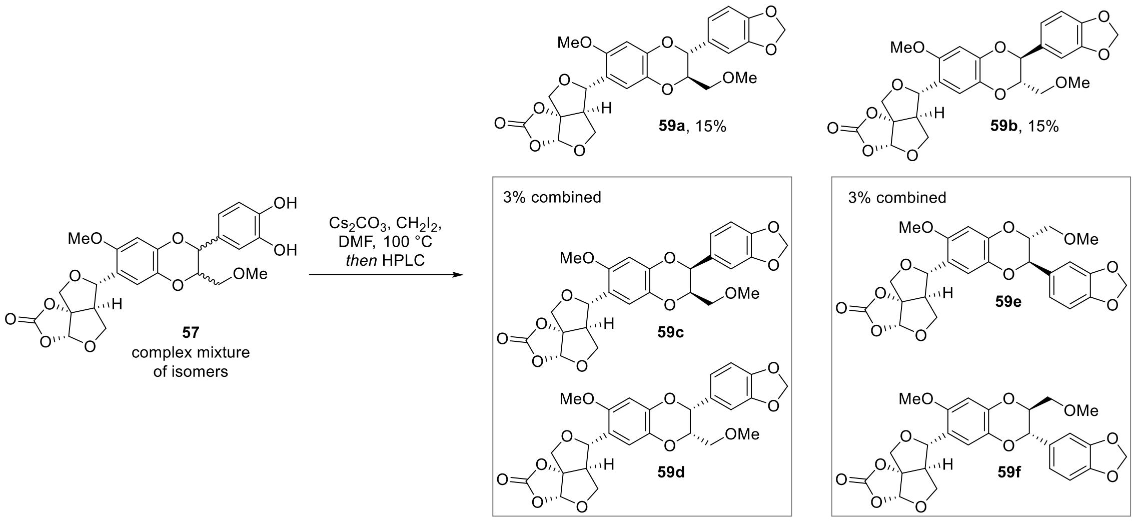

To a solution of a mixture of isomers of catechol **57** (2.00 g, 4.09 mmol, 1 equiv) in degassed *N*,*N*-dimethylformamide (20 mL) heated to 100 °C was added diiodomethane (826 µl, 10.2 mmol, 2.50 equiv) followed by cesium carbonate (2.67 g, 8.19 mmol, 2.00 equiv). After stirring for 1 h, the mixture was allowed to cool to 22 °C. Ethyl acetate was added (100 mL) and the mixture was washed with a saturated aqueous lithium chloride solution (2 × 100 mL) and a saturated aqueous sodium chloride solution (100 mL). The washed solution was dried over sodium sulfate and filtered. The filtrate was concentrated under reduced pressure and the residue was purified by flash chromatography on silica gel (35% ethyl acetate in petroleum ether) which afforded benzodioxole **59** as a complex mixture of isomers. The isomers were further separated by preparative HPLC (25% ethyl acetate in hexane grading to 35% ethyl acetate in hexane) affording the desired *trans*-isomer **59a** as a white solid (305 mg, 609 µmol, 15%), undesired *trans*-isomer **59b** as a white solid (317 mg, 633 µmol, 15%), mixture of putatively assigned *cis*-isomers **59c** and **59d** as a white solid (67.6 mg, 135 µmol, 3%) and mixture of putatively assigned regioisomers **59e** and **59f** as a white solid (56.7 mg, 113 µmol, 3%) in this order of elution.

Characterization data for benzodioxole **59a:**

TLC (30% ethyl acetate in cyclohexane): R_f = 0.30 (UV, $KMnO_4$).

^{1}H NMR (400 MHz, $CDCl_3$): δ 7.14 (d, J = 0.7 Hz, 1H), 6.95 – 6.80 (m, 3H), 6.52 (s, 1H), 6.05 (s, 1H), 6.02 – 5.94 (m, 2H), 4.99 (d, J = 8.0 Hz, 1H), 4.91 (d, J = 8.3 Hz, 1H), 4.46 (d, J = 10.7 Hz, 1H), 4.36 (dd, J = 9.9, 1.2 Hz, 1H), 4.23 (dd, J = 9.9, 5.5 Hz, 1H), 4.15 (d, J = 10.6 Hz, 1H), 4.00 (ddd, J = 8.0, 3.8, 2.3 Hz, 1H), 3.74 (s, 3H), 3.58 (dd, J = 10.9, 2.4 Hz, 1H), 3.35 (s, 3H), 3.30 (dd, J = 10.9, 3.8 Hz, 1H), 2.97 – 2.91 (m, 1H) ppm.

^{13}C NMR (101 MHz, $CDCl_3$): δ 152.2, 151.1, 148.32, 148.25, 143.9, 137.3, 130.3, 121.4, 120.4, 114.9, 108.6, 108.1, 107.7, 101.5, 100.5, 99.6, 80.9, 77.4, 76.8, 71.5, 71.3, 70.3, 59.7, 57.1, 55.9 ppm.

IR (ATR, neat): $\tilde{\nu}$ = 2897 (*w*), 1816 (*s*), 1602 (*w*), 1504 (*s*), 1445 (*m*), 1318 (*m*), 1250 (*m*), 1199 (*s*), 1109 (*m*), 1065 (*m*), 1005 (*s*), 932 (*m*), 811 (*w*), 735 (*w*) cm^{-1}.

HRMS (ESI): calcd. for $C_{25}H_{25}O_{11}$ $[M+H]^+$: 501.1391; found: 501.1405.

Characterization data for benzodioxole **59b**:

TLC (30% ethyl acetate in cyclohexane): R_f = 0.30 (UV, $KMnO_4$).

^{1}H NMR (400 MHz, $CDCl_3$): δ 7.13 (s, 1H), 6.93 – 6.83 (m, 3H), 6.52 (s, 1H), 6.05 (s, 1H), 5.00 (s, 2H), 4.97 (d, *J* = 8.1 Hz, 1H), 4.89 (d, *J* = 8.3 Hz, 1H), 4.47 (d, *J* = 10.7 Hz, 1H), 4.37 (d, *J* = 9.9 Hz, 1H), 4.23 (dd, *J* = 9.9, 5.5 Hz, 1H), 4.14 (d, *J* = 10.7 Hz, 1H), 4.04 – 3.99 (m, 1H), 3.74 (s, 3H), 3.56 (dd, *J* = 9.9, 2.3 Hz, 1H), 3.35 (s, 3H), 3.32 (dd, *J* = 10.9, 3.9 Hz, 1H), 2.96 (dd, *J* = 8.1, 5.6 Hz, 1H) ppm.

^{13}C NMR (101 MHz, $CDCl_3$): δ 152.2, 151.1, 148.32, 148.25, 143.9, 137.2, 130.3, 121.4, 120.5, 115.1, 108.7, 108.1, 107.7, 101.5, 100.5, 99.7, 81.1, 77.4, 76.7, 71.5, 71.3, 70.4, 59.7, 57.2, 55.9 ppm.

IR (ATR, neat): $\tilde{\nu}$ = 2895 (*w*), 1814 (*s*), 1602 (*w*), 1503 (*s*), 1445 (*m*), 1317 (*m*), 1250 (*m*), 1199 (*s*), 1109 (*m*), 1065 (*m*), 1005 (*s*), 932 (*m*), 890 (*w*), 812 (*w*), 735 (*m*) cm^{-1}.

HRMS (ESI): calcd. for $C_{25}H_{25}O_{11}$ $[M+H]^+$: 501.1391; found: 501.1405.

Note: The analytical data for isomers **59c**, **59d**, **59e**, and **59f** are not reported due to their complexity arising from the mixture of diastereomers.

Synthesis of diol 12

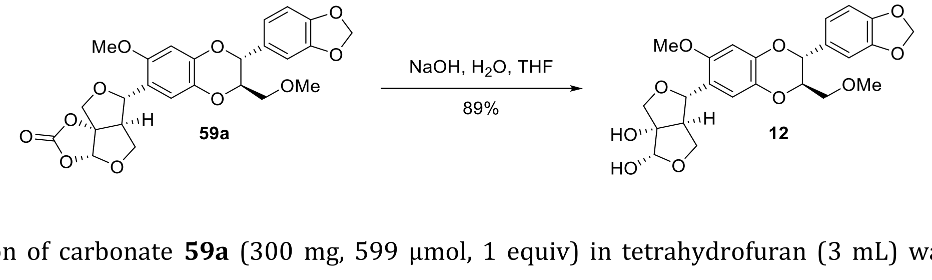

To a solution of carbonate **59a** (300 mg, 599 µmol, 1 equiv) in tetrahydrofuran (3 mL) was added an aqueous solution of sodium hydroxide (2.00 M, 3.00 mL, 6.00 mmol, 10.0 equiv) and the resulting mixture was vigorously stirred for 30 min. A saturated aqueous ammonium chloride solution (5 mL) was added and the mixture was extracted with ethyl acetate (3 × 5 mL). The organic solution was dried over sodium sulfate and filtered. The filtrate was concentrated under reduced pressure which afforded diol **12** as a white solid (253 mg, 533 µmol, 89%).

TLC (60% ethyl acetate in cyclohexane): R_f = 0.25 (UV, $KMnO_4$).

^{1}H NMR (400 MHz, $CDCl_3$): δ 7.23 (s, 1H), 6.92 – 6.88 (m, 2H), 6.84 (d, J = 8.6 Hz, 1H), 6.49 (s, 1H), 6.01 – 5.98 (m, 2H), 5.20 (s, 1H), 4.97 (d, J = 8.0 Hz, 1H), 4.86 (d, J = 6.3 Hz, 1H), 4.32 (dd, J = 9.3, 7.1 Hz, 1H), 4.24 (d, J = 9.9 Hz, 1H), 4.03 – 3.94 (m, 2H), 3.76 – 3.71 (m, 4H), 3.61 – 3.54 (m, 2H), 3.35 (s, 3H), 3.30 (dd, J = 10.9, 3.8 Hz, 1H), 3.26 (br s, 1H), 2.56 – 2.49 (m, 1H) ppm.

^{13}C NMR (101 MHz, $CDCl_3$): δ 150.9, 148.23, 148.21, 143.2, 137.1, 130.5, 122.9, 121.4, 115.3, 108.6, 107.7, 101.4, 100.2, 97.9, 92.3, 83.9, 77.4, 77.3, 76.7, 71.3, 70.1, 59.6, 58.7, 55.9 ppm.

IR (ATR, neat): $\tilde{\nu}$ = 3390 (*br*, *w*), 2895 (*w*), 1603 (*w*), 1504 (*s*), 1464 (*w*), 1445 (*m*), 1426 (*w*), 1385 (*w*), 1331 (*w*), 1316 (*w*), 1251 (*m*), 1199 (*m*), 1171 (*w*), 1128 (*w*), 1068 (*w*), 1024 (*s*), 919 (*m*), 810 (*w*), 731 (*m*) cm^{-1}.

HRMS (ESI): calcd. for $C_{24}H_{26}NaO_{10}$ $[M+Na]^+$: 497.1418; found: 497.1411.

The analytical data matched those reported previously.[29]

Synthesis of diol SI-47

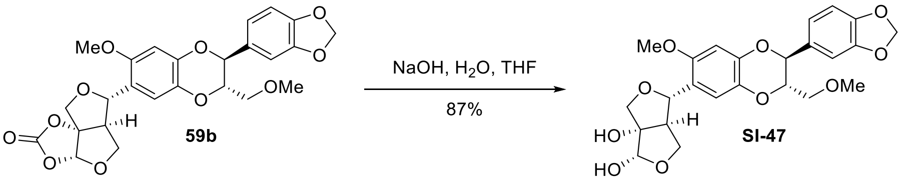

To a solution of carbonate **59b** (68 mg, 0.14 mmol, 1 equiv) in tetrahydrofuran (0.70 mL) was added an aqueous solution of sodium hydroxide (2.0 M, 0.68 mL, 1.4 mmol, 10 equiv) and the resulting mixture was vigorously stirred for 30 min. A saturated aqueous ammonium chloride solution (1 mL) was added and the mixture was extracted with ethyl acetate (3 × 1 mL). The organic solution was dried over sodium sulfate and filtered. The filtrate was concentrated under reduced pressure which afforded diol **SI-47** as a white solid (56 mg, 0.12 mmol, 87%).

TLC (60% ethyl acetate in cyclohexane): R_f = 0.25 (UV, $KMnO_4$).

^{1}H NMR (400 MHz, $CDCl_3$): δ 7.23 (d, J = 0.7 Hz, 1H), 6.89 (d, J = 7.4 Hz, 2H), 6.86 – 6.83 (m, 1H), 6.50 (s, 1H), 5.99 (s, 2H), 5.20 (s, 1H), 4.95 (d, J = 7.9 Hz, 1H), 4.84 (d, J = 6.3 Hz, 1H), 4.33 (dd, J = 9.2, 7.1 Hz, 1H), 4.25 (d, J = 10.0 Hz, 1H), 4.04 – 3.96 (m, 2H), 3.79 – 3.69 (s, 4H), 3.54 (dd, J = 10.8, 2.4 Hz, 1H), 3.46 – 3.26 (s, 5H), 3.17 (s, 1H), 2.55 (td, J = 6.5, 2.2 Hz, 1H) ppm.

^{13}C NMR (101 MHz, $CDCl_3$): δ 150.9, 148.24, 148.22, 143.2, 137.0, 130.5, 122.9, 121.4, 115.4, 108.6, 107.7, 101.4, 100.2, 97.8, 92.3, 84.1, 77.4, 77.3, 76.7, 71.4, 70.2, 59.7, 58.7, 55.9 ppm.

IR (ATR, neat): $\tilde{\nu}$ = 3395 (*br*, *w*), 2893 (*w*), 1602 (*w*), 1503 (*s*), 1464 (*w*), 1445 (*m*), 1426 (*w*), 1383 (*w*), 1330 (*w*), 1316 (*w*), 1250 (*m*), 1199 (*m*), 1172 (*w*), 1128 (*w*), 1067 (*w*), 1024 (*s*), 929 (*w*), 893 (*w*), 860 (*w*), 810 (*w*) cm^{-1}.

HRMS (ESI): calcd. for $C_{24}H_{26}NaO_{10}$ $[M+Na]^+$: 497.1418; found: 497.1411.

Synthesis of haedoxan A (1a)

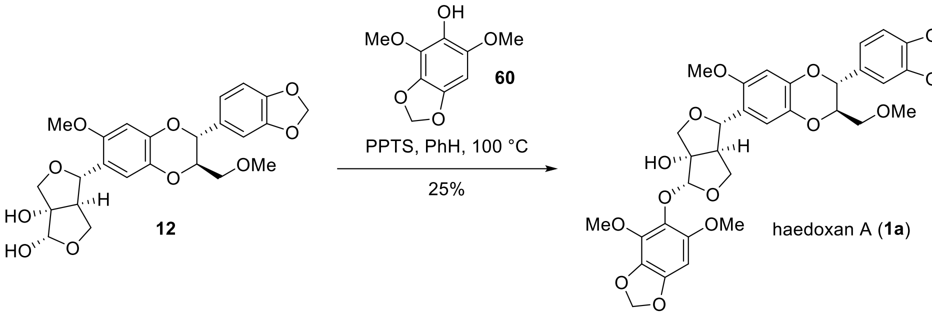

A mixture of diol **12** (45.0 mg, 94.8 µmol, 1 equiv), pyridinium 4-methylbenzenesulfonate (11.9 mg, 47.4 µmol, 0.500 equiv) and phenol **60** (188 mg, 948 µmol, 10.0 equiv) in benzene (1.0 mL) was heated to 100 °C in a sealed vial. After 3 h, the reaction mixture was allowed to cool to 22 °C. The solution was diluted with ethyl acetate (5 mL) and washed with an aqueous solution of sodium hydroxide (2 M, 5 mL) and with a saturated aqueous sodium chloride solution (5 mL). The washed organic solution was dried over sodium sulfate and filtered. The filtrate was concentrated under reduced pressure and the residue was purified by flash chromatography on silica gel (30% ethyl acetate in petroleum ether to 40% ethyl acetate in petroleum ether) which afforded haedoxan A (**1a**) as a white solid (15.3 mg, 23.4 µmol, 25%).

TLC (40% ethyl acetate in cyclohexane): R_f = 0.31 (UV, $KMnO_4$).

^{1}H NMR (400 MHz, $CDCl_3$): δ 7.31 (s, 1H), 6.94 – 6.88 (m, 2H), 6.84 (d, *J* = 8.6 Hz, 1H), 6.49 (s, 1H), 6.29 (s, 1H), 5.99 (s, 2H), 5.90 – 5.85 (m, 2H), 5.24 (s, 1H), 4.99 (d, *J* = 7.9 Hz, 1H), 4.93 (d, *J* = 5.9 Hz, 1H), 4.57 (dd, *J* = 9.2, 7.4 Hz, 1H), 4.30 (d, *J* = 9.6 Hz, 1H), 4.07 – 3.96 (m, 5H), 3.95 (d, *J* = 1.5 Hz, 1H), 3.79 (s, 3H), 3.76 – 3.71 (m, 4H), 3.58 (dd, *J* = 10.9, 2.5 Hz, 1H), 3.35 (s, 3H), 3.30 (dd, *J* = 10.9, 3.8 Hz, 1H), 2.67 – 2.61 (m, 1H) ppm.

^{13}C NMR (101 MHz, $CDCl_3$): δ 150.8, 148.2 (2C), 147.8, 145.0, 143.1, 138.2, 137.1, 131.3, 131.2, 130.7, 123.3, 121.4, 115.4, 108.6, 107.8, 106.6, 101.4, 101.2, 100.0, 92.6, 89.8, 84.0, 77.7, 77.4, 76.7, 71.9, 71.4, 60.3, 59.7, 58.0, 57.1, 55.9 ppm.

IR (ATR, neat): $\tilde{\nu}$ = 3464 (*br*, *w*), 2898 (*w*), 1626 (*w*), 1603 (*w*), 1502 (*s*), 1487 (*w*), 1467 (*w*), 1446 (*w*), 1428 (*w*), 1373 (*w*), 1332 (*w*), 1315 (*w*), 1250 (*m*), 1197 (*s*), 1173 (*w*), 1128 (*m*), 1078 (*w*), 1041 (*s*), 1013 (*w*), 985 (*w*), 918 (*m*), 866 (*w*), 809 (*w*), 731 (*m*) cm^{-1}.

HRMS (ESI): calcd. for $C_{33}H_{34}NaO_{14}$ $[M+Na]^+$: 677.1841; found: 677.1836.

The analytical data matched those reported previously.[29,32]

Synthesis of an unnatural diastereomer of haedoxan A (SI-48)

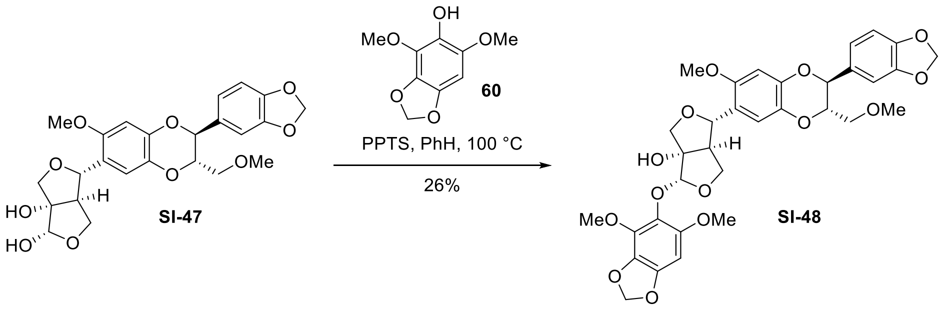

A mixture of diol **SI-47** (30.0 mg, 63.2 µmol, 1 equiv), pyridinium 4-methylbenzenesulfonate (7.94 mg, 31.6 µmol, 0.500 equiv) and phenol **60** (125 mg, 632 µmol, 10.0 equiv) in benzene (1.0 mL) was heated to 100 °C in a sealed vial. After 3 h, the reaction mixture was allowed to cool to 22 °C. The solution was diluted with ethyl acetate (5 mL) and washed with an aqueous solution of sodium hydroxide (2 M, 5 mL) and with a saturated aqueous sodium chloride solution (5 mL). The washed organic solution was dried over sodium sulfate and filtered. The filtrate was concentrated under reduced pressure and the residue was purified by flash chromatography on silica gel (30% ethyl acetate in petroleum ether to 40% ethyl acetate in petroleum ether) which afforded **SI-48** as a white solid (10.8 mg, 16.5 µmol, 26%).

TLC (40% ethyl acetate in cyclohexane): R_f = 0.31 (UV, $KMnO_4$).

^{1}H NMR (400 MHz, $CDCl_3$): δ 7.31 (s, 1H), 6.93 – 6.87 (m, 2H), 6.84 (d, J = 8.6 Hz, 1H), 6.49 (s, 1H), 6.29 (s, 1H), 5.99 (s, 2H), 5.91 – 5.85 (m, 2H), 5.24 (s, 1H), 4.97 (d, J = 8.0 Hz, 1H), 4.91 (d, J = 6.0 Hz, 1H), 4.58 (dd, J = 9.1, 7.3 Hz, 1H), 4.31 (d, J = 9.7 Hz, 1H), 4.06 (dd, J = 9.1, 2.5 Hz, 1H), 4.04 – 3.99 (m, 4H), 3.97 (d, J = 1.6 Hz, 1H), 3.78 (s, 3H), 3.76 – 3.71 (m, 4H), 3.55 (dd, J = 11.0, 2.5 Hz, 1H), 3.37 – 3.29 (m, 4H), 2.69 – 2.59 (m, 1H) ppm.

^{13}C NMR (101 MHz, $CDCl_3$): δ 150.8, 148.2 (2C), 147.8, 145.0, 143.0, 138.2, 137.0, 131.3, 131.2, 130.7, 123.4, 121.5, 115.3, 108.6, 107.8, 106.6, 101.4, 101.2, 100.0, 92.5, 89.8, 84.2, 77.7, 77.4, 76.7, 72.0, 71.4, 60.3, 59.7, 58.1, 57.1, 55.9 ppm.

IR (ATR, neat): $\tilde{\nu}$ = 3531 (*br*, *w*), 2902 (*w*), 1629 (*w*), 1603 (*w*), 1502 (*s*), 1486 (*w*), 1468 (*w*), 1446 (*w*), 1428 (*w*), 1370 (*w*), 1331 (*w*), 1315 (*w*), 1250 (*m*), 1197 (*s*), 1174 (*w*), 1128 (*m*), 1080 (*w*), 1041 (*s*), 1013 (*w*), 985 (*w*), 931 (*m*), 919 (*w*), 868 (*w*), 809 (*w*), 772 (*w*), 731 (*m*), 649 (*w*) cm^{-1}.

HRMS (ESI): calcd. for $C_{33}H_{34}NaO_{14}$ $[M+Na]^+$: 677.1841; found: 677.1833.

Synthesis of haedoxan D (1b)

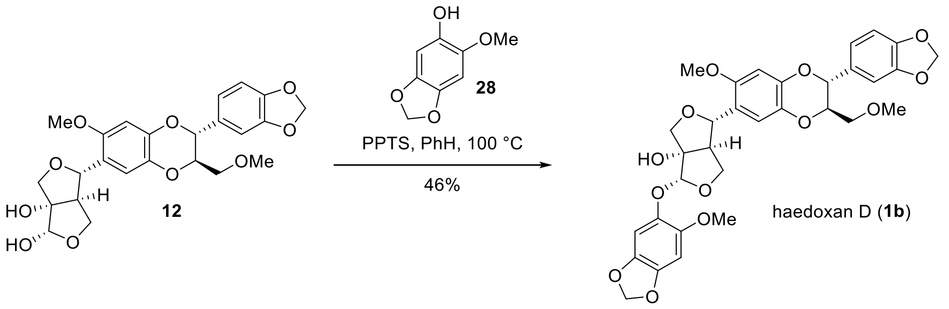

A mixture of diol **12** (50.0 mg, 105 µmol, 1 equiv), pyridinium 4-methylbenzenesulfonate (26.5 mg, 105 µmol, 1.00 equiv) and phenol **28** (177 mg, 1.05 mmol, 10.0 equiv) in benzene (1.0 mL) was heated to 100 °C in a sealed vial. After 2 h, the reaction mixture was allowed to cool to 22 °C. The solution was diluted with diethyl ether (3 mL) and dichloromethane (1 mL) and washed with an aqueous solution of sodium hydroxide (2 M, 5 mL). The aqueous solution was extracted with diethyl ether (3 mL). The organic solutions were combined and washed with a saturated aqueous sodium chloride solution (5 mL). The washed organic solution was dried over sodium sulfate and filtered. The filtrate was concentrated under reduced pressure and the residue was purified by flash chromatography on silica gel (30% ethyl acetate in petroleum ether to 40% ethyl acetate in petroleum ether) which afforded haedoxan D (**1b**) as a white solid (30.2 mg, 48.4 µmol, 46%).

TLC (50% ethyl acetate in cyclohexane): R_f = 0.50 (UV, $KMnO_4$).

^{1}H NMR (400 MHz, $CDCl_3$): δ 7.31 (s, 1H), 6.93 – 6.88 (m, 2H), 6.84 (dd, J = 8.6, 1.4 Hz, 1H), 6.80 (s, 1H), 6.56 (s, 1H), 6.50 (s, 1H), 5.99 (s, 2H), 5.94 – 5.85 (m, 2H), 5.19 (s, 1H), 4.99 (d, J = 8.0 Hz, 1H), 4.95 (d, J = 5.8 Hz, 1H), 4.45 (dd, J = 9.3, 7.3 Hz, 1H), 4.31 (d, J = 9.7 Hz, 1H), 4.08 (dd, J = 9.2, 2.6 Hz, 1H), 4.02 – 3.96 (m, 1H), 3.82 – 3.63 (m, 8H), 3.58 (dd, J = 10.9, 2.4 Hz, 1H), 3.35 (s, 3H), 3.30 (dd, J = 11.0, 3.7 Hz, 1H), 2.69 – 2.62 (m, 1H) ppm.

^{13}C NMR (101 MHz, $CDCl_3$): δ 150.8, 148.2 (2C), 145.8, 143.7, 143.1, 140.9, 139.7, 137.1, 130.7, 123.1, 121.5, 115.4, 108.6, 107.8, 106.2, 103.0, 101.5, 101.4, 100.0, 95.9, 92.3, 84.0, 77.6, 77.4, 76.7, 71.7, 71.4, 59.7, 57.9, 57.0, 55.9 ppm.

IR (ATR, neat): $\tilde{\nu}$ = 2944 (*w*), 2896 (*w*), 2851 (*w*), 1602 (*w*), 1502 (*w*), 1487 (*s*), 1465 (*w*), 1451 (*w*), 1426 (*w*), 1383 (*w*), 1331 (*w*), 1315 (*w*), 1250 (*w*), 1198 (*m*), 1182 (*w*), 1167 (*w*), 1128 (*w*), 1069 (*w*), 1036 (*s*), 1012 (*w*), 978 (*w*), 930 (*m*), 916 (*w*), 892 (*w*), 859 (*w*), 811 (*w*), 730 (*m*) cm^{-1}.

HRMS (ESI): calcd. for $C_{32}H_{32}NaO_{13}$ $[M+Na]^+$: 647.1735; found: 647.1734.

The analytical data matched those reported previously.[29,32]

Synthesis of an unnatural diastereomer of haedoxan D (SI-49)

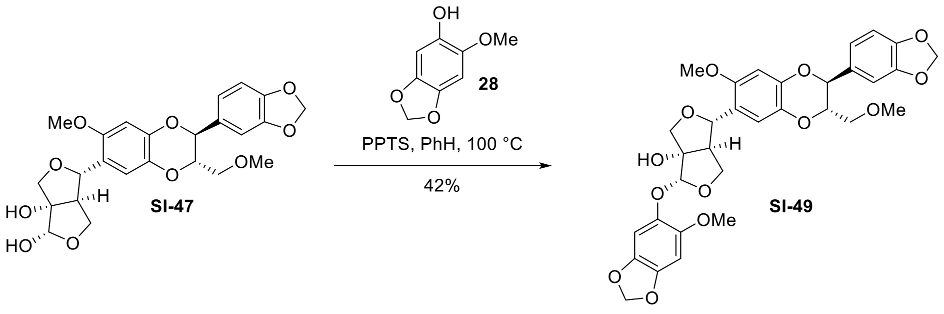

A mixture of diol **SI-47** (50.0 mg, 105 µmol, 1 equiv), pyridinium 4-methylbenzenesulfonate (26.5 mg, 105 µmol, 1.00 equiv) and phenol **28** (177 mg, 1.05 mmol, 10.0 equiv) in benzene (1.0 mL) was heated to 100 °C in a sealed vial. After 2 h, the reaction mixture was allowed to cool to 22 °C. The solution was diluted with diethyl ether (3 mL) and dichloromethane (1 mL) and washed with an aqueous solution of sodium hydroxide (2 M, 5 mL). The aqueous solution was extracted with diethyl ether (3 mL). The organic solutions were combined and washed with a saturated aqueous sodium chloride solution (5 mL). The washed organic solution was dried over sodium sulfate and filtered. The filtrate was concentrated under reduced pressure and the residue was purified by flash chromatography on silica gel (30% ethyl acetate in petroleum ether to 40% ethyl acetate in petroleum ether) which afforded acetal **SI-49** as a white solid (27.6 mg, 44.2 µmol, 42%).

TLC (50% ethyl acetate in cyclohexane): R_f = 0.50 (UV, $KMnO_4$).

^{1}H NMR (400 MHz, $CDCl_3$): δ 7.30 (s, 1H), 6.93 – 6.87 (m, 2H), 6.86 – 6.82 (m, 1H), 6.80 (s, 1H), 6.55 (s, 1H), 6.49 (s, 1H), 5.99 (s, 2H), 5.91 – 5.88 (m, 2H), 5.19 (s, 1H), 4.97 (d, *J* = 8.0 Hz, 1H), 4.92 (d, *J* = 5.8 Hz, 1H), 4.46 (dd, *J* = 9.3, 7.3 Hz, 1H), 4.32 (d, *J* = 9.8 Hz, 1H), 4.09 (dd, *J* = 9.3, 2.6 Hz, 1H), 4.01 (ddd, *J* = 7.9, 4.1, 2.4 Hz, 1H), 3.81 – 3.69 (m, 8H), 3.55 (dd, *J* = 11.0, 2.5 Hz, 1H), 3.36 – 3.29 (m, 4H), 2.66 (ddd, *J* = 7.8, 6.4, 2.6 Hz, 1H) ppm.

^{13}C NMR (101 MHz, $CDCl_3$): δ 150.8, 148.2 (2C), 145.8, 143.7, 143.1, 140.9, 139.7, 137.0, 130.7, 123.2, 121.4, 115.3, 108.6, 107.7, 106.1, 102.9, 101.5, 101.4, 100.0, 95.8, 92.2, 84.2, 77.5, 77.4, 76.6, 71.7, 71.4, 59.7, 57.9, 57.0, 55.8 ppm.

IR (ATR, neat): $\tilde{\nu}$ = 3505 (*br*, *w*), 2933 (*w*), 2895 (*w*), 2839 (*w*), 2250 (*w*), 1625 (*w*), 1602 (*w*), 1503 (*w*), 1487 (*s*), 1466 (*w*), 1451 (*w*), 1426 (*w*), 1382 (*w*), 1331 (*w*), 1315 (*w*), 1250 (*m*), 1198 (*m*), 1183 (*w*), 1165 (*w*), 1128 (*w*), 1069 (*w*), 1035 (*s*), 1012 (*w*), 977 (*w*), 931 (*m*), 914 (*w*), 858 (*w*), 811 (*w*), 730 (*m*), 647 (*w*) cm^{-1}.

HRMS (ESI): calcd. for $C_{32}H_{32}NaO_{13}$ $[M+Na]^+$: 647.1735; found: 647.1734.

Synthesis of haedoxan analogues:

General procedure B for synthesis of acetals from diol 12

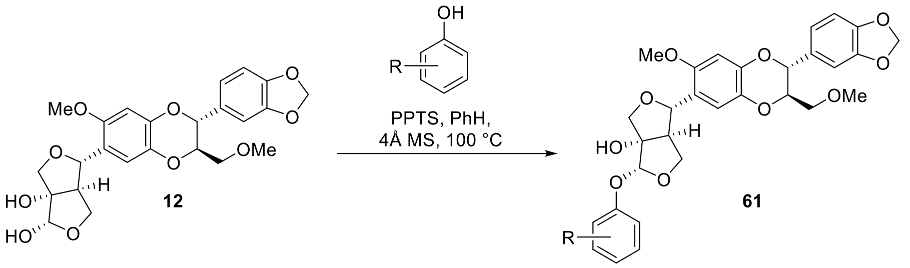

A mixture of diol **12** (1 equiv), pyridinium 4-methylbenzenesulfonate (1.00 equiv), corresponding phenol (10.0 equiv) and 4Å molecular sieves (50 mg) in benzene (0.1 M) was heated to 100 °C in a sealed vial. After 3 h, the reaction mixture was allowed to cool to 22 °C. The solution was diluted with diethyl ether (3 mL) and dichloromethane (1 mL) and washed with an aqueous solution of sodium hydroxide (2 M, 5 mL). The aqueous layer was extracted with diethyl ether (3 mL). The organic solutions were combined and washed with a saturated aqueous sodium chloride solution (5 mL). The washed organic solution was dried over sodium sulfate and filtered. The filtrate was concentrated under reduced pressure and the residue containing acetal **61** was purified by flash chromatography on silica gel.

Acetal 61a:

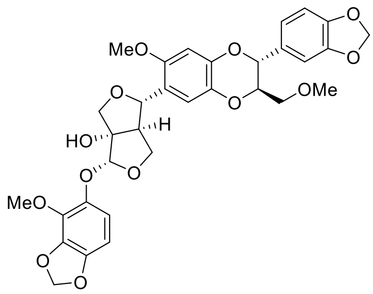

Compound **61a** was synthesized according to general procedure B using diol **12** (15 mg, 32 µmol, 1 equiv), pyridinium 4-methylbenzenesulfonate (7.9 mg, 32 µmol, 1.0 equiv) and phenol **SI-43** (53 mg, 0.32 mmol, 10.0 equiv). The product was purified by flash chromatography on silica gel (40% ethyl acetate in petroleum ether) which afforded acetal **61a** as a colorless oil (4.3 mg, 6.9 µmol, 22%).

TLC (40% ethyl acetate in cyclohexane): R_f = 0.41 (UV, $KMnO_4$).

^{1}H NMR (400 MHz, $CDCl_3$): δ 7.31 (s, 1H), 6.91 (d, J = 7.1 Hz, 2H), 6.87 – 6.82 (m, 1H), 6.65 (d, J = 8.4 Hz, 1H), 6.50 (s, 1H), 6.44 (d, J = 8.5 Hz, 1H), 6.00 (s, 2H), 5.96 – 5.89 (m, 2H), 5.21 (s, 1H), 4.99 (d, J = 8.0 Hz, 1H), 4.95 (d, J = 5.8 Hz, 1H), 4.44 (dd, J = 9.3, 7.3 Hz, 1H), 4.32 (d, J = 9.7 Hz, 1H), 4.07 (dd, J = 9.2, 2.5 Hz, 1H), 4.00 (s, 4H), 3.77 (d, J = 9.8 Hz, 1H), 3.73 (s, 3H), 3.59 (dd, J = 11.0, 2.4 Hz, 1H), 3.35 (s, 3H), 3.30 (dd, J = 11.0, 3.8 Hz, 1H), 2.67 – 2.60 (m, 1H) ppm.

^{13}C NMR (101 MHz, $CDCl_3$): δ 150.8, 148.2, 145.4, 143.4, 143.1, 137.5, 137.1, 136.2, 130.6, 123.1, 121.5, 115.4, 112.5, 108.6, 107.8, 106.0, 101.7, 101.5, 101.4, 100.1, 92.3, 84.0, 77.5, 77.4, 76.9, 76.7, 71.6, 71.4, 60.2, 59.7, 58.0, 55.9 ppm.

IR (ATR, neat): $\tilde{\nu}$ = 3520 (*w*), 2896 (*w*), 1603 (*w*), 1492 (*m*), 1465 (*s*), 1426 (*w*), 1332 (*w*), 1244 (*m*), 1199 (*w*), 1172 (*w*), 1129 (*w*), 1066 (*w*), 1041 (*s*), 920 (*w*), 809 (*w*), 731 (*w*), 649 (*w*) cm^{-1}.

HRMS (ESI): calcd. for $C_{32}H_{32}NaO_{13}$ $[M+Na]^+$: 647.1735; found: 647.1727.

Acetal 61b:

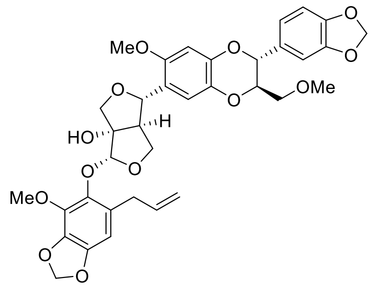

Compound **61b** was synthesized according to general procedure B using diol **12** (20.0 mg, 42.2 µmol, 1 equiv), pyridinium 4-methylbenzenesulfonate (10.6 mg, 42.2 µmol, 1.00 equiv) and phenol **SI-45** (87.8 mg, 422 µmol, 10.0 equiv). The product was purified by flash chromatography on silica gel (30% ethyl acetate in petroleum ether) which afforded acetal **61b** as a colorless oil (7.0 mg, 11 µmol, 25%).

TLC (30% ethyl acetate in cyclohexane): R_f = 0.23 (UV, $KMnO_4$).

^{1}H NMR (400 MHz, $CDCl_3$): δ 7.31 (s, 1H), 6.94 – 6.88 (m, 2H), 6.87 – 6.82 (m, 1H), 6.50 (s, 1H), 6.38 (s, 1H), 5.99 (s, 2H), 5.97 – 5.86 (m, 3H), 5.15 (s, 1H), 5.08 (dq, *J* = 7.3, 1.7 Hz, 1H), 5.05 (t, *J* = 1.5 Hz, 1H), 4.99 (d, *J* = 8.0 Hz, 1H), 4.93 (d, *J* = 6.0 Hz, 1H), 4.47 (dd, *J* = 9.3, 7.1 Hz, 1H), 4.32 (d, *J* = 9.6 Hz, 1H), 4.07 (dd, *J* = 9.3, 2.5 Hz, 1H), 4.01 (s, 4H), 3.82 (d, *J* = 1.2 Hz, 1H), 3.73 (s, 4H), 3.58 (dd, *J* = 11.0, 2.4 Hz, 1H), 3.43 (ddt, *J* = 15.5, 6.7, 1.5 Hz, 1H), 3.38 – 3.28 (m, 5H), 2.70 – 2.62 (m, 1H) ppm.

^{13}C NMR (101 MHz, $CDCl_3$): δ 150.8, 148.2, 145.6, 143.1, 140.8, 137.1, 137.1, 136.8, 135.5, 130.7, 126.7, 123.2, 121.5, 116.0, 115.3, 108.6, 107.9, 107.8, 102.9, 101.4, 100.0, 92.1, 83.8, 78.1, 77.4, 76.7, 71.5, 71.4, 60.0, 59.7, 58.2, 55.9, 34.5 ppm.

IR (ATR, neat): $\tilde{\nu}$ = 3522 (*w*), 2895 (*w*), 1626 (*w*), 1603 (*w*), 1502 (*m*), 1482 (*w*), 1457 (*w*), 1426 (*m*), 1381 (*w*), 1332 (*w*), 1314 (*w*), 1250 (*m*), 1199 (*m*), 1173 (*w*), 1127 (*w*), 1081 (*w*), 1040 (*s*), 988 (*w*), 917 (*m*), 866 (*w*), 833 (*w*), 810 (*w*) cm^{-1}.

HRMS (ESI): calcd. for $C_{35}H_{36}NaO_{13}$ $[M+Na]^+$: 687.2048; found: 687.2041.

Acetal 61c:

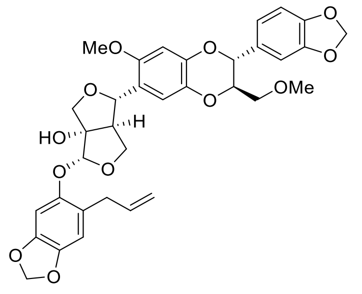

Compound **61c** was synthesized according to general procedure B using diol **12** (12 mg, 25 µmol, 1 equiv), pyridinium 4-methylbenzenesulfonate (6.4 mg, 25 µmol, 1.0 equiv) and phenol **SI-38** (45 mg, 0.25 mmol, 10 equiv). The product was purified by flash chromatography on silica gel (30% ethyl acetate in petroleum ether) which afforded acetal **61c** as a colorless oil (2.0 mg, 3.2 µmol, 12%).

TLC (30% ethyl acetate in cyclohexane): R_f = 0.37 (UV, $KMnO_4$).

^{1}H NMR (400 MHz, $CDCl_3$): *δ* 7.30 (d, *J* = 0.8 Hz, 1H), 6.91 (d, *J* = 7.4 Hz, 2H), 6.86 – 6.83 (m, 1H), 6.80 (s, 1H), 6.64 (s, 1H), 6.50 (s, 1H), 6.00 (q, *J* = 1.4 Hz, 2H), 5.95 – 5.85 (m, 3H), 5.27 (s, 1H), 5.07 – 4.97 (m, 3H), 4.95 (d, *J* = 6.0 Hz, 1H), 4.38 – 4.30 (m, 2H), 4.07 (dd, *J* = 9.3, 2.5 Hz, 1H), 4.00 (ddd, *J* = 8.0, 3.7, 2.4 Hz, 1H), 3.78 (d, *J* = 9.7 Hz, 1H), 3.73 (s, 3H), 3.59 (dd, *J* = 10.9, 2.4 Hz, 1H), 3.35 (s, 3H), 3.34 – 3.23 (m, 3H), 3.13 (br s, 1H), 2.63 – 2.56 (m, 1H) ppm.

^{13}C NMR (101 MHz, $CDCl_3$): *δ* 150.8, 148.6, 148.23, 148.22, 146.7, 143.2, 137.2, 137.1, 130.6, 123.0, 122.2, 121.5, 115.8, 115.3, 109.8, 108.6, 107.8, 103.5, 101.4, 100.13, 100.07, 92.5, 84.1, 77.4, 77.2 (overlapping with $CDCl_3$ signal), 76.7, 71.4, 71.2, 59.7, 58.4, 55.9, 34.9 ppm.

IR (ATR, neat): $\tilde{\nu}$ = 3526 (*br*, *w*), 2894 (*w*), 1603 (*w*), 1503 (*s*), 1484 (*w*), 1444 (*w*), 1426 (*w*), 1374 (*w*), 1332 (*w*), 1315 (*w*), 1251 (*m*), 1199 (*m*), 1174 (*w*), 1148 (*w*), 1128 (*w*), 1070 (*w*), 1037 (*s*), 930 (*m*), 892 (*w*), 870 (*w*), 861 (*w*), 834 (*w*), 810 (*w*), 783 (*w*), 730 (*w*) cm^{-1}.

HRMS (ESI): calcd. for $C_{34}H_{34}NaO_{12}$ $[M+Na]^+$: 657.1942; found: 657.1936.

Acetal 61d:

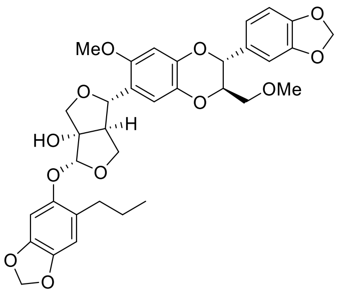

Compound **61d** was synthesized according to general procedure B using diol **12** (10 mg, 21 µmol, 1 equiv), pyridinium 4-methylbenzenesulfonate (5.3 mg, 21 µmol, 1.0 equiv) and phenol **SI-39** (38 mg, 0.21 mmol, 10 equiv). The product was purified by flash chromatography on silica gel (30% ethyl acetate in petroleum ether) which afforded acetal **61d** as a colorless oil (4.9 mg, 7.7 µmol, 37%).

TLC (30% ethyl acetate in cyclohexane): R_f = 0.38 (UV, $KMnO_4$).

^{1}H NMR (500 MHz, $CDCl_3$): δ 7.31 (d, J = 0.7 Hz, 1H), 6.91 (d, J = 7.3 Hz, 2H), 6.87 – 6.82 (m, 1H), 6.80 (s, 1H), 6.63 (s, 1H), 6.50 (s, 1H), 6.02 – 5.98 (m, 2H), 5.91 (s, 2H), 5.24 (s, 1H), 4.99 (d, J = 8.0 Hz, 1H), 4.96 (d, J = 6.0 Hz, 1H), 4.41 – 4.31 (m, 2H), 4.08 (dd, J = 9.4, 2.5 Hz, 1H), 4.00 (ddd, J = 8.0, 3.7, 2.4 Hz, 1H), 3.80 (d, J = 9.8 Hz, 1H), 3.74 (s, 3H), 3.59 (dd, J = 11.0, 2.4 Hz, 1H), 3.35 (s, 3H), 3.30 (dd, J = 10.9, 3.7 Hz, 1H), 3.09 (s, 1H), 2.62 (ddd, J = 7.9, 4.8, 1.6 Hz, 1H), 2.54 – 2.45 (m, 2H), 1.55 (dt, J = 9.7, 7.4 Hz, 2H), 0.93 (t, J = 7.3 Hz, 3H) ppm.

^{13}C NMR (126 MHz, $CDCl_3$): δ 150.8, 148.5, 148.22, 148.21, 146.1, 143.2, 143.1, 137.1, 130.6, 125.2, 123.0, 121.5, 115.3, 109.5, 108.6, 107.8, 104.0, 101.4, 101.3, 100.4, 100.1, 92.4, 84.0, 77.41 (overlapping with $CDCl_3$ signal), 77.37, 76.7, 71.4, 71.1, 59.7, 58.5, 55.9, 32.4, 23.8, 14.1 ppm.

IR (ATR, neat): $\tilde{\nu}$ = 2956 (*w*), 2894 (*w*), 2873 (*w*), 1603 (*w*), 1503 (*w*), 1484 (*s*), 1445 (*w*), 1427 (*w*), 1376 (*w*), 1331 (*w*), 1315 (*w*), 1250 (*m*), 1199 (*m*), 1175 (*w*), 1148 (*w*), 1129 (*w*), 1069 (*w*), 1036 (*s*), 996 (*w*), 933 (*m*), 917 (*w*), 893 (*w*), 859 (*w*), 833 (*w*), 808 (*w*), 732 (*w*) cm^{-1}.

HRMS (ESI): calcd. for $C_{34}H_{36}NaO_{12}$ $[M+Na]^+$: 659.2099; found: 659.2101.

Acetal 61e:

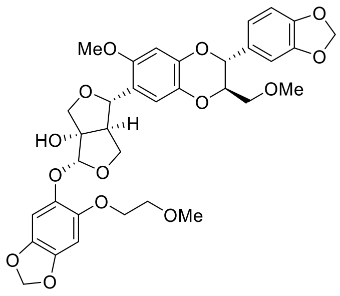

Compound **61e** was synthesized according to general procedure B using diol **12** (15 mg, 32 µmol, 1 equiv), pyridinium 4-methylbenzenesulfonate (7.9 mg, 32 µmol, 1.0 equiv) and phenol **SI-42** (67 mg, 0.32 mmol, 10 equiv). The product was purified by flash chromatography on silica gel (40% ethyl acetate in petroleum ether grading to 60% ethyl acetate in petroleum ether) which afforded acetal **61e** as a colorless oil (4.0 mg, 6.0 µmol, 19%).

TLC (40% ethyl acetate in cyclohexane): R_f = 0.16 (UV, $KMnO_4$).

^{1}H NMR (500 MHz, $CDCl_3$): δ 7.32 (s, 1H), 6.91 (d, J = 7.3 Hz, 2H), 6.86 – 6.82 (m, 1H), 6.81 (s, 1H), 6.60 (s, 1H), 6.49 (s, 1H), 5.99 (s, 2H), 5.93 – 5.86 (m, 2H), 5.23 (s, 1H), 4.99 (d, J = 7.9 Hz, 1H), 4.94 (d, J = 6.0 Hz, 1H), 4.42 (dd, J = 9.2, 7.2 Hz, 1H), 4.30 (d, J = 9.6 Hz, 1H), 4.17 (d, J = 1.5 Hz, 1H), 4.08 – 3.96 (m, 4H), 3.76 (dd, J = 9.7, 1.6 Hz, 1H), 3.73 (s, 3H), 3.67 (td, J = 4.1, 2.7 Hz, 2H), 3.59 (dd, J = 11.0, 2.4 Hz, 1H), 3.40 (s, 3H), 3.35 (s, 3H), 3.30 (dd, J = 11.0, 3.7 Hz, 1H), 2.65 – 2.58 (m, 1H) ppm.

^{13}C NMR (126 MHz, $CDCl_3$): δ 150.8, 148.2, 144.0, 143.07, 143.05, 142.4, 141.9, 137.1, 130.7, 123.3, 121.4, 115.4, 108.6, 107.8, 105.2, 102.0, 101.5, 101.4, 100.2, 100.0, 92.3, 84.0, 77.9, 77.4, 76.7, 71.5, 71.39, 71.35, 59.7, 59.3, 58.1, 55.9 ppm.

IR (ATR, neat): $\tilde{\nu}$ = 3505 (*br*, *w*), 2893 (*w*), 1603 (*w*), 1503 (*s*), 1486 (*w*), 1445 (*w*), 1332 (*w*), 1250 (*m*), 1199 (*m*), 1170 (*m*), 1127 (*w*), 1069 (*w*), 1034 (*s*), 931 (*m*), 859 (*w*), 731 (*w*) cm^{-1}.

HRMS (ESI): calcd. for $C_{34}H_{36}NaO_{14}$ $[M+Na]^+$: 691.1997; found: 691.1991.

Acetal 61f:

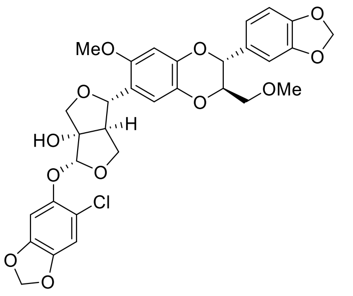

Compound **61f** was synthesized according to general procedure B using diol **12** (10 mg, 21 µmol, 1 equiv), pyridinium 4-methylbenzenesulfonate (5.3 mg, 21 µmol, 1.0 equiv) and phenol **SI-36** (36 mg, 0.21 mmol, 10 equiv). The product was purified by flash chromatography on silica gel (40% ethyl acetate in petroleum ether) which afforded acetal **61f** as a colorless oil (5.7 mg, 9.1 µmol, 43%).

TLC (40% ethyl acetate in cyclohexane): R_f = 0.45 (UV, $KMnO_4$).

^{1}H NMR (400 MHz, $CDCl_3$): δ 7.33 (s, 1H), 6.95 – 6.89 (m, 3H), 6.87 (d, J = 9.0 Hz, 2H), 6.52 (s, 1H), 6.02 (s, 2H), 5.99 (s, 2H), 5.31 (s, 1H), 5.00 (dd, J = 10.4, 6.9 Hz, 2H), 4.46 (dd, J = 9.3, 7.3 Hz, 1H), 4.37 (d, J = 9.8 Hz, 1H), 4.12 (dd, J = 9.3, 2.6 Hz, 1H), 4.02 (ddd, J = 7.9, 3.7, 2.4 Hz, 1H), 3.82 (d, J = 9.8 Hz, 1H), 3.76 (s, 3H), 3.61 (dd, J = 10.9, 2.4 Hz, 1H), 3.49 – 3.29 (m, 5H), 2.74 – 2.65 (m, 1H) ppm.

^{13}C NMR (101 MHz, $CDCl_3$): δ 150.8, 148.21, 148.20, 147.2, 146.7, 143.7, 143.2, 137.1, 130.6, 123.0, 121.5, 116.2, 115.3, 109.7, 108.6, 107.8, 104.8, 102.1, 101.42, 101.39, 100.1, 92.5, 84.1, 77.4, 77.3, 76.7, 71.8, 71.4, 59.7, 57.9, 55.9 ppm.

IR (ATR, neat): $\tilde{\nu}$ = 3546 (*w*), 2896 (*w*), 1652 (*w*), 1626 (*w*), 1603 (*w*), 1503 (*w*), 1476 (*s*), 1445 (*w*), 1426 (*w*), 1383 (*w*), 1331 (*w*), 1315 (*w*), 1249 (*m*), 1198 (*w*), 1178 (*m*), 1128 (*w*), 1072 (*w*), 1036 (*s*), 1011 (*w*), 973 (*w*), 932 (*m*), 913 (*w*), 855 (*w*), 811 (*w*), 793 (*w*), 731 (*m*), 649 (*w*) cm^{-1}.

HRMS (ESI): calcd. for $C_{31}H_{29}ClNaO_{12}$ $[M+Na]^+$: 651.1240; found: 651.1231.

Acetal 61g:

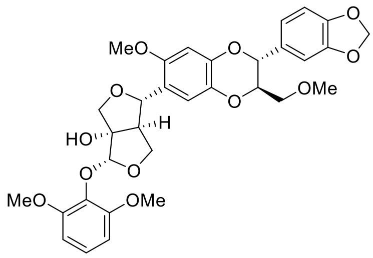

Compound **61g** was synthesized according to general procedure B using diol **12** (14 mg, 30 µmol, 1 equiv), pyridinium 4-methylbenzenesulfonate (7.4 mg, 30 µmol, 1.0 equiv) and 2,6-dimethoxyphenol (46 mg, 0.30 mmol, 10 equiv). The product was purified by flash chromatography on silica gel (40% ethyl acetate in petroleum ether) which afforded acetal **61g** as an orange solid (5.5 mg, 9.0 µmol, 31%).

TLC (40% ethyl acetate in cyclohexane): R_f = 0.31 (UV, $KMnO_4$).

^{1}H NMR (400 MHz, $CDCl_3$): δ 7.31 (d, *J* = 0.6 Hz, 1H), 7.03 (t, *J* = 8.4 Hz, 1H), 6.92 – 6.88 (m, 2H), 6.86 – 6.82 (m, 1H), 6.61 (s, 1H), 6.59 (s, 1H), 6.49 (s, 1H), 5.99 (s, 2H), 5.36 (s, 1H), 4.99 (d, *J* = 7.9 Hz, 1H), 4.94 (d, *J* = 5.9 Hz, 1H), 4.59 (dd, *J* = 9.2, 7.2 Hz, 1H), 4.29 (d, *J* = 9.6 Hz, 1H), 4.17 (br s, 1H), 4.06 (dd, *J* = 9.1, 2.6 Hz, 1H), 3.99 (ddd, *J* = 8.0, 3.7, 2.4 Hz, 1H), 3.86 (s, 6H), 3.74 (d, *J* = 9.9 Hz, 4H), 3.58 (dd, *J* = 10.9, 2.4 Hz, 1H), 3.35 (s, 3H), 3.30 (dd, *J* = 10.9, 3.7 Hz, 1H), 2.71 – 2.63 (m, 1H) ppm.

^{13}C NMR (101 MHz, $CDCl_3$): δ 153.1, 150.9, 148.2, 143.0, 137.1, 134.2, 130.7, 124.5, 123.4, 121.5, 115.4, 108.6, 107.8, 106.2, 105.4, 101.4, 100.0, 92.6, 84.0, 77.8, 77.4, 76.7, 72.1, 71.4, 59.7, 58.0, 56.3, 55.9 ppm.

IR (ATR, neat): $\tilde{\nu}$ = 3488 (*br*, *w*), 2979 (*w*), 2936 (*w*), 2897 (*w*), 2839 (*w*), 1600 (*m*), 1496 (*s*), 1479 (*w*), 1466 (*w*), 1445 (*w*), 1426 (*w*), 1384 (*w*), 1331 (*w*), 1312 (*w*), 1254 (*m*), 1211 (*w*), 1200 (*m*), 1172 (*w*), 1111 (*s*), 1080 (*w*), 1033 (*w*), 975 (*w*), 931 (*w*), 891 (*w*), 858 (*w*), 826 (*w*), 809 (*w*), 775 (*w*), 730 (*w*) cm^{-1}.

HRMS (ESI): calcd. for $C_{32}H_{34}NaO_{12}$ $[M+Na]^+$: 633.1942; found: 633.1933.

Acetal 61h:

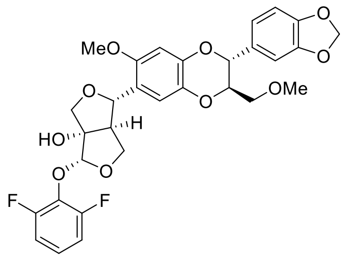

Compound **61h** was synthesized according to general procedure B using diol **12** (15 mg, 32 μmol, 1 equiv), pyridinium 4-methylbenzenesulfonate (7.9 mg, 32 μmol, 1.0 equiv) and 2,6-difluorophenol (41 mg, 0.32 mmol, 10 equiv). The product was purified by flash chromatography on silica gel (30% ethyl acetate in petroleum ether) which afforded acetal **61h** as a colorless oil (3.0 mg, 5.1 μmol, 16%).

TLC (30% ethyl acetate in cyclohexane): R_f = 0.40 (UV, $KMnO_4$).

^{1}H NMR (400 MHz, $CDCl_3$): δ 7.31 (s, 1H), 7.08 – 7.02 (m, 1H), 6.97 – 6.89 (m, 4H), 6.85 (d, J = 7.9 Hz, 1H), 6.50 (s, 1H), 6.00 (s, 2H), 5.42 (s, 1H), 4.99 (d, J = 8.0 Hz, 1H), 4.94 (d, J = 5.9 Hz, 1H), 4.55 (dd, J = 9.3, 7.5 Hz, 1H), 4.37 (d, J = 9.8 Hz, 1H), 4.09 (dd, J = 9.4, 2.6 Hz, 1H), 4.00 (ddd, J = 8.1, 3.8, 2.4 Hz, 1H), 3.77 (d, J = 9.9 Hz, 1H), 3.73 (s, 3H), 3.59 (dd, J = 11.0, 2.5 Hz, 1H), 3.35 (s, 3H), 3.31 (dd, J = 11.0, 3.8 Hz, 1H), 3.14 (s, 1H), 2.71 – 2.64 (m, 1H) ppm.

^{13}C NMR (101 MHz, $CDCl_3$): δ 157.7, 157.6, 155.7, 155.6, 150.8, 148.22, 148.21, 143.2, 137.1, 132.0 (t), 130.6, 124.5 (t), 122.9, 121.5, 115.3, 112.5 (d), 112.3 (d), 108.6, 107.8, 105.5 (t), 101.4, 100.1, 93.1, 84.3, 77.4, 76.7, 72.2, 71.4, 59.7, 57.7, 55.9 ppm.

IR (ATR, neat): $\tilde{\nu}$ = 3586 (*br*, *w*), 2906 (*w*), 1600 (*w*), 1498 (*s*), 1475 (*w*), 1445 (*w*), 1426 (*w*), 1332 (*w*), 1315 (*w*), 1292 (*w*), 1243 (*m*), 1213 (*w*), 1199 (*m*), 1172 (*w*), 1129 (*w*), 1081 (*w*), 1035 (*w*), 1008 (*s*), 966 (*w*), 915 (*m*), 870 (*w*), 832 (*w*), 810 (*w*), 780 (*m*), 731 (*m*) cm^{-1}.

HRMS (ESI): calcd. for $C_{30}H_{28}F_2NaO_{10}$ $[M+Na]^+$: 609.1543; found: 609.1536.

Epoxidation-cyclization strategy towards phrymarolins:

Synthesis of thioacetal SI-51

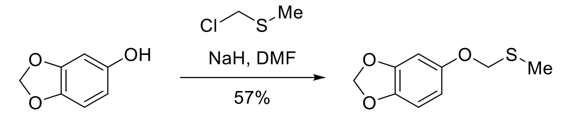

To a solution of phenol **23** (2.00 g, 14.5 mmol, 1 equiv) in *N,N*-dimethylformamide (20 mL) cooled to 0 °C was added sodium hydride (60 wt% in mineral oil, 695 mg, 17.4 mmol, 1.20 equiv) portionwise. After stirring for 15 min, chloromethyl methyl sulfide (1.43 mL, 17.4 mmol, 1.20 equiv) was added. After stirring for 5 min at 0 °C, the suspension was allowed to warm to 22 °C. After stirring for 2 h, a saturated aqueous sodium bicarbonate solution (50 mL) was added. The mixture was extracted with ethyl acetate (50 mL). The organic solution was washed with a 10% aqueous lithium chloride solution (2 × 50 mL). The washed solution was dried with sodium sulfate and filtered. The filtrate was concentrated under reduced pressure and the residue was purified by flash chromatography on silica gel (5% diethyl ether in petroleum ether) which afforded thioacetal **SI-51** as a yellow oil (1.63 g, 8.22 mmol, 57%).

TLC (5% ethyl acetate in cyclohexane): R_f = 0.50 (UV, $KMnO_4$).

^{1}H NMR (400 MHz, $CDCl_3$): δ 6.72 (d, J = 8.4 Hz, 1H), 6.55 (d, J = 2.5 Hz, 1H), 6.40 (dd, J = 8.5, 2.5 Hz, 1H), 5.93 (s, 2H), 5.07 (s, 2H), 2.24 (s, 3H) ppm.

^{13}C NMR (101 MHz, $CDCl_3$): δ 152.4, 148.4, 142.7, 108.2, 108.1, 101.4, 99.8, 73.9, 14.7 ppm.

IR (ATR, neat): $\tilde{\nu}$ = 2921 (*w*), 2893 (*w*), 1631 (*w*), 1501 (*m*), 1483 (*s*), 1450 (*w*), 1266 (*w*), 1240 (*m*), 1174 (*s*), 1129 (*m*), 1098 (*w*), 1037 (*m*), 997 (*m*), 747 (*w*), 733 (*w*) cm^{-1}.

HRMS (ESI): calcd. for $C_9H_{11}O_3S$ [M+H]$^+$: 199.0423; found: 199.0419.

Synthesis of chloromethyl ether SI-52

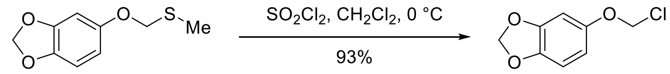

To a solution of thioacetal **SI-51** (1.63 g, 8.22 mmol, 1 equiv) in dichloromethane (40 mL) cooled to 0 °C was added sulfuryl dichloride (668 µL, 8.22 mmol, 1 equiv) and the resulting solution was stirred at 0 °C for 30 min. The solution was concentrated under reduced pressure and the residue was purified by flash

chromatography on silica gel (10% diethyl ether in petroleum ether) which afforded chloromethyl ether **SI-52** as a colorless oil (1.42 g, 7.61 mmol, 93%).

TLC (20% ethyl acetate in cyclohexane): R_f = 0.45 (UV, $KMnO_4$).

^{1}H NMR (400 MHz, $CDCl_3$): δ 6.76 (d, J = 8.5 Hz, 1H), 6.65 (d, J = 2.5 Hz, 1H), 6.56 (dd, J = 8.5, 2.5 Hz, 1H), 5.96 (s, 2H), 5.82 (s, 2H) ppm.

^{13}C NMR (101 MHz, $CDCl_3$): δ 151.0, 148.5, 143.9, 108.7, 108.3, 101.7, 99.9, 78.8 ppm.

IR (ATR, neat): $\tilde{\nu}$ = 2897 (*w*), 1632 (*w*), 1502 (*m*), 1484 (*s*), 1325 (*w*), 1271 (*w*), 1241 (*m*), 1174 (*s*), 1137 (*s*), 1112 (*w*), 1097 (*w*), 1037 (*s*), 938 (*w*), 919 (*w*), 844 (*w*), 814 (*w*), 746 (*w*) cm^{-1}.

HRMS (ESI): calcd. for $C_8H_7O_3$ [M-Cl$^-$]$^+$: 151.0390; found: 151.0386.

Synthesis of thioacetal SI-53:

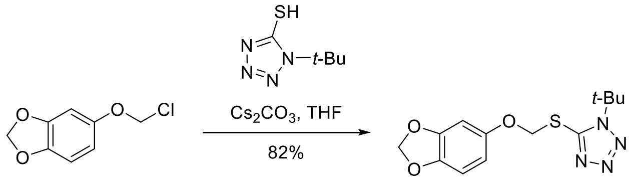

SI-52 **SI-53**

To a stirred suspension of 1-*tert*-butyl-1H-tetrazol-5-thiol (204 mg, 1.29 mmol, 1.20 equiv) in tetrahydrofuran (5 mL) at 22 °C was added cesium carbonate (698 mg, 2.14 mmol, 2.00 equiv) followed by a solution of chloromethyl ether **SI-52** (200 mg, 1.07 mmol, 1 equiv) in tetrahydrofuran (5 mL). After 1 h, water was added (10 mL) and the mixture was extracted with dichloromethane (2 × 20 mL). The organic solution was washed with a saturated aqueous sodium chloride solution (20 mL). The washed solution was dried over sodium sulfate, filtered and concentrated under reduced pressure. The residue was purified by flash chromatography on silica gel (20% ethyl acetate in petroleum ether) which afforded thioacetal **SI-53** as a pale-yellow oil (270 mg, 876 μmol, 82%).

TLC (20% ethyl acetate in cyclohexane): R_f = 0.39 (UV, $KMnO_4$).

^{1}H NMR (400 MHz, $CDCl_3$): δ 6.70 (d, J = 8.5 Hz, 1H), 6.55 (d, J = 2.5 Hz, 1H), 6.40 (dd, J = 8.5, 2.5 Hz, 1H), 5.96 (s, 2H), 5.93 (s, 2H), 1.69 (s, 9H) ppm.

^{13}C NMR (101 MHz, $CDCl_3$): δ 151.6, 150.8, 148.6, 143.5, 108.4, 108.1, 101.6, 100.1, 73.1, 61.5, 29.1 ppm.

IR (ATR, neat): $\tilde{\nu}$ = 2985 (*w*), 2897 (*w*), 1631 (*w*), 1502 (*m*), 1483 (*s*), 1393 (*m*), 1364 (*m*), 1240 (*m*), 1221 (*m*), 1172 (*s*), 1127 (*s*), 1094 (*s*), 1024 (*s*), 934 (*m*), 913 (*m*), 840 (*m*), 814 (*m*), 744 (*m*), 666 (*m*), 592 (*m*) cm^{-1}.

HRMS (ESI): calcd. for $C_{13}H_{16}N_4NaO_3S$ $[M+Na]^+$: 331.0835; found: 331.0840.

Synthesis of sulfone 42b

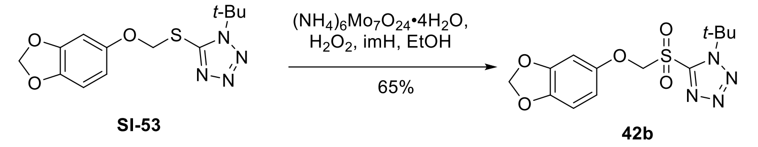

To a stirred solution of thioacetal **SI-53** (533 mg, 1.73 mmol, 1 equiv) in ethanol (20 mL) was added imidazole (400 mg, 5.88 mmol, 3.40 equiv), aqueous hydrogen peroxide (30 wt%, 1.77 mL, 17.3 mmol, 10.0 equiv) and ammonium heptamolybdate tetrahydrate (680 mg, 584 µmol, 33 mol%). After 16 h at 22 °C, another portion of aqueous hydrogen peroxide (30 wt%, 1.23 mL, 12.0 mmol, 7.00 equiv) was added. After 2 h, ethyl acetate (30 mL), a saturated aqueous sodium chloride solution (15 mL) and water (15 mL) were added. The organic phase was separated and the aqueous solutions were combined and extracted with ethyl acetate (30 mL). The organic solutions were combined and washed with a saturated aqueous sodium chloride solution (30 mL). The organic solution was dried over sodium sulfate, filtered and concentrated under reduced pressure. The residue was purified by flash chromatography on silica gel (20% ethyl acetate in petroleum ether) which afforded sulfone **42b** as a white solid (383 mg, 1.13 mmol, 65%).

TLC (20% ethyl acetate in cyclohexane): R_f = 0.45 (UV, $KMnO_4$).

^{1}H NMR (400 MHz, $CDCl_3$): δ 6.71 (d, *J* = 8.5 Hz, 1H), 6.63 (d, *J* = 2.5 Hz, 1H), 6.53 (dd, *J* = 8.5, 2.5 Hz, 1H), 5.96 (s, 2H), 5.73 (s, 2H), 1.87 (s, 9H) ppm.

^{13}C NMR (101 MHz, $CDCl_3$): δ 153.1, 152.6, 148.6, 144.3, 109.0, 108.2, 101.9, 100.4, 86.2, 65.7, 29.8 ppm.

IR (ATR, neat): $\tilde{\nu}$ = 2990 (*w*), 2907 (*w*), 1633 (*w*), 1503 (*m*), 1485 (*s*), 1408 (*w*), 1376 (*w*), 1348 (*m*), 1240 (*m*), 1181 (*m*), 1159 (*s*), 1136 (*m*), 1122 (*m*), 1037 (*m*), 936 (*w*), 917 (*w*), 815 (*w*), 751 (*w*), 635 (*w*) cm^{-1}.

HRMS (ESI): calcd. for $C_{13}H_{16}N_4NaO_5S$ $[M+Na]^+$: 363.0734; found: 363.0729.

Synthesis of thioacetal SI-54

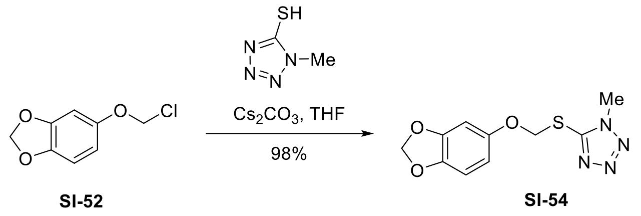

To a stirred suspension of 1-methyl-1H-tetrazol-5-thiol (373 mg, 3.22 mmol, 1.20 equiv) in tetrahydrofuran (15 mL) at 22 °C was added cesium carbonate (1.75 g, 5.36 mmol, 2.00 equiv) followed by a solution of chloromethyl ether **SI-52** (500 mg, 2.68 mmol, 1 equiv) in tetrahydrofuran (10 mL). After 1 h at 22 °C, water was added (20 mL) and the mixture was extracted with dichloromethane (2 × 20 mL). The organic solution was washed with a saturated aqueous sodium chloride solution (20 mL). The washed solution was dried over sodium sulfate, filtered and concentrated under reduced pressure which afforded thioacetal **SI-54** as a pale-yellow oil (700 mg, 2.63 mmol, 98%) that was used without further purification.

TLC (20% ethyl acetate in cyclohexane): R_f = 0.20 (UV, $KMnO_4$).

^{1}H NMR (400 MHz, $CDCl_3$): δ 6.71 (d, J = 8.4 Hz, 1H), 6.52 (d, J = 2.5 Hz, 1H), 6.38 (dd, J = 8.5, 2.5 Hz, 1H), 5.94 (s, 2H), 5.84 (s, 2H), 3.90 (s, 3H) ppm.

^{13}C NMR (101 MHz, $CDCl_3$): δ 152.3, 151.1, 148.6, 143.7, 108.3, 108.2, 101.7, 100.0, 73.4, 33.9 ppm.

IR (ATR, neat): $\tilde{\nu}$ = 2898 (*w*), 1631 (*w*), 1504 (*m*), 1483 (*s*), 1451 (*m*), 1394 (*w*), 1269 (*m*), 1241 (*m*), 1171 (*s*), 1130 (*m*), 1104 (*m*), 1094 (*m*), 1030 (*s*), 937 (*m*), 914 (*m*), 815 (*w*), 700 (*w*), 666 (*w*) cm^{-1}.

HRMS (ESI): calcd. for $C_{10}H_{10}N_4NaO_3S$ [M+Na]$^+$: 289.0366; found: 289.0364.

Synthesis of sulfone 42a

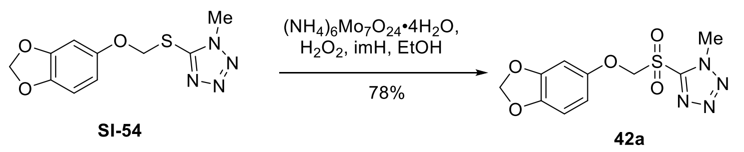

To a stirred solution of thioacetal **SI-54** (700 mg, 2.63 mmol, 1 equiv) in ethanol (12 mL) was added imidazole (537 mg, 7.89 mmol, 3.00 equiv), aqueous hydrogen peroxide (30 wt%, 2.68 mL, 26.3 mmol, 10.0 equiv) and ammonium heptamolybdate tetrahydrate (918 mg, 789 µmol, 30 mol %). After 16 h, another portion of aqueous hydrogen peroxide (30 wt%, 1.00 mL, 9.79 mmol, 3.72 equiv) was added. After 2 h, ethyl acetate (30 mL), a saturated aqueous sodium chloride solution (15 mL) and water (15 mL) were added. The organic phase was separated and the aqueous solutions were combined and extracted with ethyl acetate

(30 mL). The organic solutions were combined and washed with a saturated aqueous sodium chloride solution (30 mL). The organic solution was dried over sodium sulfate, filtered and concentrated under reduced pressure. The residue was purified by flash chromatography on silica gel (30% ethyl acetate in petroleum ether) which afforded sulfone **42a** as a white solid (612 mg, 2.05 mmol, 78%).

TLC (30% ethyl acetate in cyclohexane): R_f = 0.42 (UV, $KMnO_4$).

^{1}H NMR (400 MHz, $CDCl_3$): δ 6.69 (d, J = 8.5 Hz, 1H), 6.53 (d, J = 2.6 Hz, 1H), 6.41 (dd, J = 8.5, 2.6 Hz, 1H), 5.96 (s, 2H), 5.44 (s, 2H), 4.35 (s, 3H) ppm.

^{13}C NMR (101 MHz, $CDCl_3$): δ 152.2, 151.9, 148.8, 144.6, 108.4, 108.3, 102.0, 99.7, 85.2, 36.6 ppm.

IR (ATR, neat): $\tilde{v}$ = 2911 (*w*), 1633 (*w*), 1613 (*w*), 1502 (*w*), 1484 (*s*), 1449 (*w*), 1423 (*w*), 1406 (*w*), 1356 (*s*), 1312 (*w*), 1268 (*w*), 1238 (*w*), 1178 (*m*), 1147 (*w*), 1129 (*s*), 1035 (*s*), 935 (*m*), 915 (*w*), 842 (*w*), 815 (*w*), 758 (*m*), 715 (*w*) cm^{-1}.

HRMS (ESI): calcd. for $C_{10}H_{10}N_4NaO_5S$ $[M+Na]^+$: 321.0264; found: 321.0263.

Table 3: Julia-Kocienski olefination screening

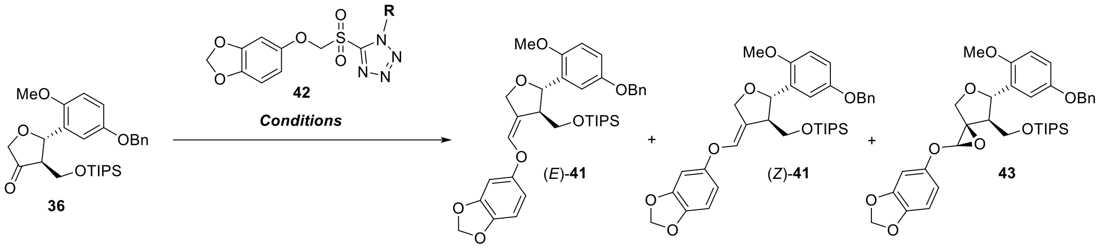

Entry	*R*	*Base*	*Solvent*	*Time Deprot.*[a] *(min)*	*Temp. Reaction (°C)*	*NMR Yield*[b] *of E-41 : Z-41 : 43*[c] *: 36*	*E : Z*
1	*t*-Bu	LHMDS	THF	1	−78 → −40	32 : 5 : 56 : 0	34 : 66
2	*t*-Bu	NHMDS	THF	1	−78 → −40	34 : 66 : 0 : 0	34 : 66
3	*t*-Bu	KHMDS	THF	1	−78 → −40	23 : 73 : 0 : 0	24 : 76
4	*t*-Bu	NHMDS	THF	-[d]	−40	32 : 58 : 0 : 11	36 : 64
5	*t*-Bu	LHMDS	THF	1	−78	30 : 14 : 42 : 0	35 : 65
6	*t*-Bu	LHMDS	THF, HMPA (3 eq)	1	−78 → −40	34 : 22 : 32 : 0	38 : 62
7[e]	Me	LHMDS	THF	-[d]	−78 → −40	56 : 34 : 0 : 2	62 : 38
8	Me	NHMDS	THF	-[d]	−78 → −40	22 : 67: 0 : 4	26 : 74
9	Me	KHMDS	THF	-[d]	−78 → −40	14 : 76 : 0 : 2	16 : 84
10	Me	LHMDS	PhMe	-[d]	−55	19 : 19 : 9 : 5	50 : 50
11	Me	LHMDS	DMF	-[d]	−55	27 : 28 : 0 : 23	49 : 51
12	Me	LHMDS	THF	1	−55	32 : 36 : 0 : 4	47 : 53
13	Me	LHMDS	DME	-[d]	−55	39 : 56 : 4 : 1	43 : 57
14	Me	LHMDS	Et_2O	-[d]	−55	18 : 14 : 0 : 9	56 : 44
15	Me	LHMDS	THF	-[d]	−95 → −40	40 : 49 : 0 : 3	45 : 55
16	Me	LHMDS	THF, LiCl (5 eq)	-[d]	−78 → −40	40 : 44 : 0 : 4	48 : 52
17	*t*-Bu	KHMDS	THF, 18-C-6	-[d]	−78	28 : 20 : 0 : 0	58 : 42

[a] Base was added to sulfone **42** followed by addition of ketone **36** after the indicated time. [b] The yield was determined from the crude reaction mixture using 1,2,4,5-tetrachloro-3-nitrobenzene as an internal reference; [c] The compound arises from a Corey-Chaykovsky-like reactivity; [d] Sulfone **42** and ketone **36** were mixed prior to addition of base. [e] Even though these were found to be the best-performing conditions, a variable amount up to 10% of unreacted **36** was observed when repeating the experiment. Since **36** was co-polar with the enol ether products **41**, we decided for the preparative scale to add ketone **36** to deprotonated sulfone **42a** which led to complete consumption of the ketone while slightly diminishing the favorable selectivity; HMPA = hexamethylphosphoramide, DME = 1,2-dimethoxyethane.

Synthesis of enol ethers (*E*)-41 and (*Z*)-41

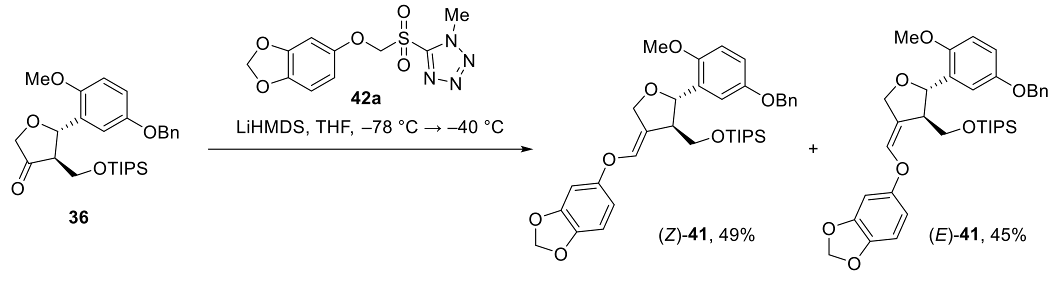

To a solution of sulfone **42a** (160 mg, 536 µmol, 1.30 equiv) in tetrahydrofuran (2 mL) cooled to –78 °C was added a solution of lithium bis(trimethylsilyl)amide (1.00 M, 536 µL, 536 µmol, 1.30 equiv) and the resulting solution was stirred for 5 min. A solution of ketone **36** (200 mg, 413 µmol, 1 equiv) in tetrahydrofuran (2 mL) was added dropwise at –78 °C and the resulting solution was allowed to warm to –40 °C. After 1 h, a saturated solution of ammonium chloride (5 mL) was added and the resulting mixture was allowed to warm to 22 °C. The mixture was extracted with ethyl acetate (2 × 10 mL). The organic solution was dried over sodium sulfate and filtered. The filtrate was concentrated under reduced pressure and the residue was purified by flash chromatography on silica gel (8% diethyl ether in petroleum ether grading to 10% diethyl ether in petroleum ether) which afforded enol ether (*Z*)-**41** as a colorless oil (125 mg, 202 µmol, 49%) and enol ether (*E*)-**41** as a colorless oil (115 mg, 186 µmol, 45%).

Characterization data for enol ether (*Z*)-**41**:

TLC (10% ethyl acetate in cyclohexane): R_f = 0.50 (UV, $KMnO_4$).

^{1}H NMR (400 MHz, $CDCl_3$): δ 7.45 – 7.27 (m, 5H), 7.08 (d, *J* = 3.0 Hz, 1H), 6.83 (dd, *J* = 8.8, 3.0 Hz, 1H), 6.77 (d, *J* = 8.9 Hz, 1H), 6.68 (d, *J* = 8.4 Hz, 1H), 6.54 (d, *J* = 2.5 Hz, 1H), 6.44 – 6.37 (m, 2H), 5.97 – 5.85 (m, 2H), 5.14 (d, *J* = 4.2 Hz, 1H), 5.02 (s, 2H), 4.83 (dd, *J* = 13.5, 2.3 Hz, 1H), 4.64 (ddd, *J* = 13.4, 2.5, 1.8 Hz, 1H), 3.96 (dd, *J* = 9.5, 5.4 Hz, 1H), 3.82 – 3.68 (m, 4H), 2.93 (m, 1H), 1.16 – 1.00 (m, 21H) ppm.

^{13}C NMR (101 MHz, $CDCl_3$): δ 153.0, 152.9, 150.9, 148.4, 143.1, 137.5, 135.0, 132.0, 128.7, 128.0, 127.7, 123.7, 113.9, 113.7, 111.2, 108.2, 108.1, 101.5, 99.4, 78.5, 70.8, 68.6, 65.3, 55.8, 51.3, 18.2, 12.1 ppm.

IR (ATR, neat): $\tilde{\nu}$ = 2942 (*m*), 2892 (*m*), 2864 (*m*), 1699 (*w*), 1499 (*m*), 1484 (*s*), 1381 (*w*), 1277 (*m*), 1246 (*m*), 1212 (*m*), 1179 (*s*), 1141 (*m*), 1104 (*m*), 1039 (*s*), 943 (*m*), 882 (*m*), 772 (*m*), 735 (*w*), 683 (*w*) cm^{-1}.

HRMS (ESI): calcd. for $C_{36}H_{46}NaO_7Si$ $[M+Na]^+$: 641.2905; found: 641.2896.

Characterization data for enol ether (*E*)-**41**:

TLC (10% ethyl acetate in cyclohexane): R_f = 0.42 (UV, $KMnO_4$).

^{1}H NMR (400 MHz, $CDCl_3$): δ 7.43 – 7.30 (m, 5H), 7.08 (d, J = 3.0 Hz, 1H), 6.81 (dd, J = 8.9, 3.0 Hz, 1H), 6.76 (d, J = 8.8 Hz, 1H), 6.68 (d, J = 8.5 Hz, 1H), 6.49 (d, J = 2.5 Hz, 1H), 6.38 (q, J = 1.8 Hz, 1H), 6.35 (dd, J = 8.5, 2.5 Hz, 1H), 5.92 (s, 2H), 5.50 (d, J = 4.4 Hz, 1H), 5.02 (s, 2H), 4.62 – 4.48 (m, 2H), 4.01 (d, J = 4.5 Hz, 2H), 3.76 (s, 3H), 3.17 (m, 1H), 1.14 – 1.01 (m, 21H) ppm.

^{13}C NMR (101 MHz, $CDCl_3$): δ 153.0, 152.9, 151.2, 148.3, 143.1, 137.6, 134.1, 132.6, 128.6, 127.9, 127.7, 124.1, 114.2, 113.8, 111.3, 108.10, 108.10, 101.5, 99.4, 78.5, 70.8, 69.5, 63.5, 55.8, 50.6, 18.1, 12.2 ppm.

IR (ATR, neat): $\tilde{\nu}$ = 2942 (*m*), 2891 (*m*), 2864 (*m*), 1819 (*w*), 1702 (*w*), 1632 (*w*), 1500 (*m*), 1484 (*s*), 1382 (*w*), 1277 (*m*), 1247 (*m*), 1216 (*m*), 1178 (*s*), 1143 (*m*), 1108 (*m*), 1071 (*m*), 1037 (*s*), 929 (*m*), 882 (*m*), 798 (*m*), 735 (*w*) cm^{-1}.

HRMS (ESI): calcd. for $C_{36}H_{46}NaO_7Si$ $[M+Na]^+$: 641.2905; found: 641.2896.

Synthesis of enol ether (*E*)-41 and epoxide 43

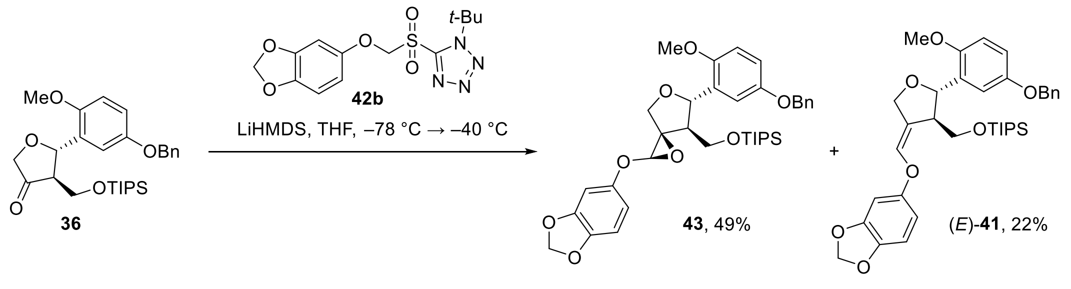

To a solution of sulfone **42b** (54 mg, 0.16 mmol, 1.1 equiv) in tetrahydrofuran (0.50 mL) cooled to –78 °C was added a solution of lithium bis(trimethylsilyl)amide (1.0 M, 0.16 mL, 0.16 mmol, 1.1 equiv) and the resulting solution was stirred for 5 min. A solution of ketone **36** (70 mg, 0.14 mmol, 1 equiv) in tetrahydrofuran (0.50 mL) was added dropwise at –78 °C and the resulting solution was allowed to warm to –40 °C. After 1 h, a saturated solution of ammonium chloride (5 mL) was added and the resulting mixture was allowed to warm to 22 °C. The mixture was extracted with ethyl acetate (2 × 10 mL). The organic solution was dried over sodium sulfate and filtered. The filtrate was concentrated under reduced pressure and the residue was purified by flash chromatography on silica gel (6% diethyl ether in petroleum ether grading to 10% diethyl ether in petroleum ether) which afforded epoxide **43** as a colorless wax (45 mg, 71 µmol, 49%) and enol ether (*E*)-**41** as a colorless oil (20 mg, 32 µmol, 22%).

The analytical data of enol ether (*E*)-**41** matched those obtained before (see above).

Characterization data for epoxide **43**:

TLC (10% ethyl acetate in cyclohexane): R_f = 0.45 (UV, $KMnO_4$).

¹H NMR (400 MHz, $CDCl_3$): δ 7.46 – 7.29 (m, 5H), 7.17 (d, J = 3.0 Hz, 1H), 6.86 (dd, J = 8.9, 3.1 Hz, 1H), 6.79 (d, J = 8.9 Hz, 1H), 6.73 (d, J = 8.4 Hz, 1H), 6.62 (d, J = 2.4 Hz, 1H), 6.52 (dd, J = 8.4, 2.5 Hz, 1H), 5.94 (t, J = 1.0 Hz, 2H), 5.24 (d, J = 8.6 Hz, 1H), 5.13 (d, J = 0.6 Hz, 1H), 5.03 (d, J = 1.3 Hz, 2H), 4.45 (d, J = 10.8 Hz, 1H), 4.04 (d, J = 10.8 Hz, 1H), 3.86 (dd, J = 9.9, 7.8 Hz, 1H), 3.77 (s, 3H), 3.71 (dd, J = 9.9, 4.0 Hz, 1H), 2.44 (td, J = 8.1, 4.0 Hz, 1H), 1.07 – 0.97 (m, 21H) ppm.

¹³C NMR (101 MHz, $CDCl_3$): δ 153.2, 151.6, 151.3, 148.5, 143.5, 137.4, 130.3, 128.7, 128.0, 127.7, 114.8, 114.0, 111.6, 108.29, 108.26, 101.6, 99.7, 82.4, 70.9, 69.2, 68.9, 59.5, 55.9, 50.6, 18.1, 18.1, 12.0 ppm.

IR (ATR, neat): $\tilde{\nu}$ = 2942 (*w*), 2865 (*m*), 1612 (*w*), 1501 (*s*), 1487 (*w*), 1382 (*w*), 1278 (*w*), 1247 (*w*), 1221 (*w*), 1179 (*s*), 1135 (*w*), 1110 (*w*), 1078 (*w*), 1039 (*m*), 944 (*w*), 928 (*w*), 883 (*w*), 842 (*w*), 799 (*w*), 736 (*w*), 694 (*w*) cm^{-1}.

HRMS (ESI): calcd. for $C_{36}H_{46}NaO_8Si$ $[M+Na]^+$: 657.2854; found: 657.2853.

Synthesis of epoxide 2-*epi*-40

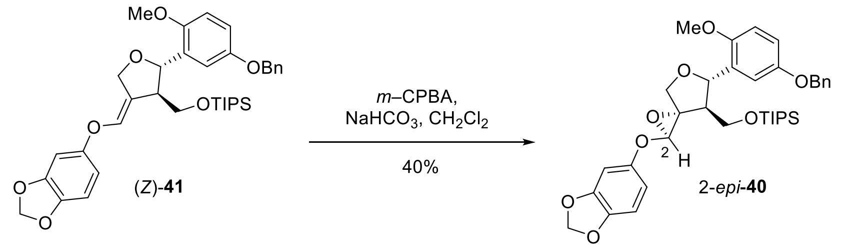

To a solution of enol ether (*Z*)-**41** (63 mg, 0.10 mmol, 1 equiv) in dichloromethane (0.50 mL) was added sodium bicarbonate (21 mg, 0.24 mmol, 2.4 equiv) followed by 3-chloroperoxybenzoic acid (27 mg, 77 wt%, 0.12 mmol, 1.20 equiv). After 30 min a saturated aqueous sodium bicarbonate solution (1 mL) was added and the mixture was extracted with dichloromethane (2 × 1 mL). The organic solutions were combined and dried over sodium sulfate and filtered. The filtrate was concentrated under reduced pressure and the residue was purified by flash chromatography on silica gel (10% diethyl ether in petroleum ether) which afforded epoxide 2-*epi*-**40** as a colorless oil (39 mg, 41 µmol, 40%).

TLC (10% ethyl acetate in cyclohexane): R_f = 0.34 (UV, $KMnO_4$).

¹H NMR (400 MHz, $CDCl_3$): δ 7.48 – 7.30 (m, 5H), 7.26 (d, J = 3.0 Hz, 1H), 6.88 (dd, J = 8.8, 3.1 Hz, 1H), 6.81 (d, J = 8.9 Hz, 1H), 6.75 (d, J = 8.4 Hz, 1H), 6.65 (d, J = 2.4 Hz, 1H), 6.57 (dd, J = 8.5, 2.4 Hz, 1H), 5.97 (s, 2H),

5.36 (s, 1H), 5.26 (d, *J* = 5.9 Hz, 1H), 5.06 (s, 2H), 4.34 (d, *J* = 11.2 Hz, 1H), 4.20 (d, *J* = 11.2 Hz, 1H), 4.05 – 3.89 (m, 2H), 3.80 (s, 3H), 2.35 (td, *J* = 6.1, 4.3 Hz, 1H), 1.08 (m, 21H) ppm.

^{13}C NMR (101 MHz, $CDCl_3$): *δ* 153.2, 151.7, 151.0, 148.4, 143.5, 137.5, 130.7, 128.7, 128.0, 127.7, 114.7, 113.9, 111.4, 108.6, 108.2, 101.5, 99.9, 81.1, 78.1, 70.8, 70.8, 70.1, 61.9, 55.8, 52.9, 18.1, 12.0 ppm.

IR (ATR, neat): $\tilde{\nu}$ = 2942 (*m*), 2892 (*m*), 2865 (*m*), 1612 (*w*), 1500 (*s*), 1487 (*s*), 1465 (*s*), 1382 (*s*), 1278 (*m*), 1246 (*m*), 1218 (*m*), 1179 (*s*), 1112 (*m*), 1066 (*m*), 1039 (*s*), 925 (*m*), 882 (*m*), 813 (*m*), 735 (*m*), 683 (*m*) cm^{-1}.

HRMS (ESI): calcd. for $C_{36}H_{46}NaO_8Si$ $[M+Na]^+$: 657.2854; found: 657.2853.

Synthesis of epoxide 40

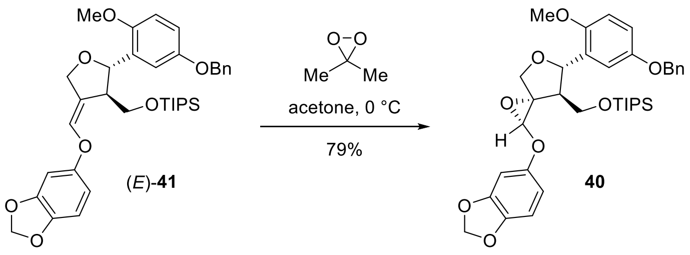

To a solution of enol ether (*E*)-**41** (180 mg, 291 µmol, 1 equiv) in acetone (1 mL) at 0 °C was added a solution of dimethyldioxirane in acetone (65 mM, 5.4 mL, 0.35 mmol, 1.2 equiv). After stirring for 2 h, the solution was concentrated under reduced pressure and the residue was purified by flash chromatography on silica gel (10% diethyl ether in petroleum ether) which afforded epoxide **40** as a colorless oil (145 mg, 228 µmol, 79%).

Note: The dimethyldioxirane solution was prepared following a procedure reported in literature.[39] The concentration of the solution was determined from a ^{1}H NMR spectrum of the reaction of 8.5 mg of thioanisol with 1.00 mL of the dimethyldioxirane solution.

TLC (10% ethyl acetate in cyclohexane): R_f = 0.25 (UV, $KMnO_4$).

^{1}H NMR (400 MHz, $CDCl_3$): *δ* 7.47 – 7.27 (m, 6H), 6.82 (dd, *J* = 8.9, 3.0 Hz, 1H), 6.76 (d, *J* = 8.9 Hz, 1H), 6.72 (d, *J* = 8.4 Hz, 1H), 6.61 (d, *J* = 2.4 Hz, 1H), 6.53 (dd, *J* = 8.5, 2.4 Hz, 1H), 5.94 (s, 2H), 5.60 (d, *J* = 4.1 Hz, 1H), 5.08 (s, 1H), 5.04 (s, 2H), 4.41 – 4.25 (m, 2H), 4.09 (dd, *J* = 9.5, 2.5 Hz, 1H), 3.84 (d, *J* = 10.1 Hz, 1H), 3.77 (s, 3H), 2.51 (q, *J* = 3.5 Hz, 1H), 1.23 – 1.09 (m, 21H) ppm.

^{13}C NMR (101 MHz, $CDCl_3$): *δ* 153.2, 151.7, 150.9, 148.5, 143.6, 137.6, 132.4, 128.6, 127.9, 127.8, 114.1, 113.7, 111.2, 108.5, 108.3, 101.6, 99.8, 80.8, 78.8, 73.1, 70.8, 69.9, 61.4, 55.7, 53.2, 18.18, 18.17, 12.2 ppm.

IR (ATR, neat): ṽ = 2942 (*w*), 2891 (*w*), 2865 (*w*), 1612 (*w*), 1489 (*s*), 1382 (*w*), 1277 (*m*), 1246 (*m*), 1219 (*m*), 1179 (*s*), 1105 (*m*), 1074 (*m*), 1038 (*s*), 927 (*m*), 882 (*m*), 802 (*m*), 753 (*m*), 693 (*m*) cm^{-1}.

HRMS (ESI): calcd. for $C_{36}H_{46}NaO_8Si$ $[M+Na]^+$: 657.2854; found: 657.2853.

Deprotection-cyclization cascade of epoxide 40

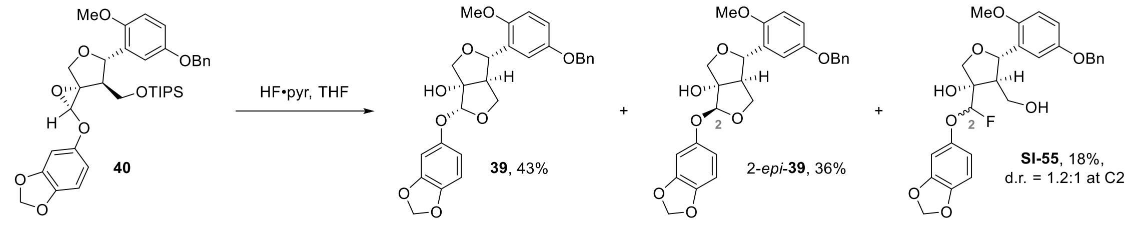

To a solution of epoxide **40** (140 mg, 221 µmol, 1 equiv) in tetrahydrofuran (2.5 mL) was added hydrogen fluoride-pyridine complex (190 µL, 7.72 mmol, 35.0 equiv) and the resulting solution was stirred for 16 h. The solution was poured into a vigorously stirred saturated aqueous sodium bicarbonate solution (20 mL). The mixture was extracted with ethyl acetate (2 × 20 mL). The organic solution was dried over sodium sulfate and filtered. The filtrate was concentrated under reduced pressure and the residue was purified by flash chromatography on silica gel (20% ethyl acetate in petroleum ether grading to 40% ethyl acetate in petroleum ether) which afforded acetal **39** as a colorless oil (39.1 mg, 93.9 µmol, 43%), acetal 2-*epi*-**39** as a colorless oil (33.2 mg, 79.7 µmol, 36%) and a 1.2:1 mixture of two fluorides **SI-55** (17.3 mg, 39.6 µmol, 18%).

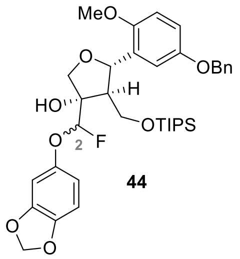

Note: The fluoride intermediates **44** formed in the initial phase of the reaction were identified by analyzing an aliquot of the reaction mixture - ^{1}H NMR: 6.15 (d, J_{H-F} = 60.6 Hz) and 6.13 (d, J_{H-F} = 60.6 Hz) ppm; ^{19}F NMR: –134.0 (d, J_{H-F} = 60.6 Hz) and –135.8 (d, J_{H-F} = 60.6 Hz) ppm; HRMS calcd. for $C_{36}H_{47}FNaO_8Si$ $[M+Na]^+$: 677.2916; found: 677.2891.

Analytical data for acetal **39** matched those obtained before (see above).

Characterization data for acetal 2-*epi*-**39**:

TLC (30% ethyl acetate in cyclohexane): R_f = 0.39 (UV, $KMnO_4$).

^{1}H NMR (400 MHz, $CDCl_3$): δ 7.46 – 7.29 (m, 5H), 7.24 (d, J = 3.1 Hz, 1H), 6.85 (dd, J = 8.8, 3.1 Hz, 1H), 6.78 (d, J = 8.9 Hz, 1H), 6.72 (d, J = 8.4 Hz, 1H), 6.68 (d, J = 2.4 Hz, 1H), 6.55 (dd, J = 8.5, 2.5 Hz, 1H), 5.93 (s, 2H), 5.43 (s, 1H), 5.04 (s, 2H), 5.00 (d, J = 6.5 Hz, 1H), 4.49 (d, J = 10.2 Hz, 1H), 4.27 (dd, J = 9.5, 8.1 Hz, 1H), 4.20 (dd, J = 9.6, 5.9 Hz, 1H), 4.07 (d, J = 10.2 Hz, 1H), 3.77 (s, 3H), 2.72 (m, 1H), 2.41 (br s, 1H) ppm.

^{13}C NMR (101 MHz, $CDCl_3$): δ 153.1, 152.1, 150.5, 148.3, 143.1, 137.4, 130.8, 128.7, 128.0, 127.7, 114.2, 113.3, 111.3, 109.2, 108.2, 107.3, 101.4, 100.4, 94.1, 82.5, 74.3, 70.8, 70.5, 60.9, 55.8 ppm.

IR (ATR, neat): $\tilde{\nu}$ = 3403 (*w, br*), 2947 (*w*), 2891 (*w*), 2836 (*w*), 1610 (*w*), 1499 (*s*), 1486 (*s*), 1465 (*m*), 1374 (*w*), 1277 (*m*), 1246 (*m*), 1219 (*m*), 1182 (*s*), 1131 (*m*), 1037 (*s*), 929 (*m*), 737 (*m*), 697 (*w*) cm^{-1}.

HRMS (ESI): calcd. for $C_{27}H_{26}NaO_8$ $[M+Na]^+$: 501.1520; found: 501.1515.

Characterization data for fluorides **SI-55**:

TLC (40% ethyl acetate in cyclohexane): R_f = 0.28 (UV, $KMnO_4$).

^{1}H NMR (400 MHz, $CDCl_3$): δ 7.47 – 7.29 (m, 5H), 7.21 – 7.17 (m, 1H), 6.90 – 6.80 (m, 2H), 6.74 – 6.70 (m, 1H), 6.66 – 6.63 (m, 1H), 6.58 – 6.54 (m, 1H), 5.95 (s, 2H), 5.67 (d, J = 62.0, 1H), 5.43 – 5.35 (m, 1H), 5.06 – 4.98 (m, 2H), 4.49 – 4.41 (m, 1H), 4.15 – 4.00 (m, 2H), 3.97 – 3.92 (m, 1H), 3.91 – 3.80 (m, 4H), 2.63 – 2.53 (m, 1H), 2.33 – 2.25 (m, 1H) ppm.

Note: The ^{13}C NMR and the IR spectra were not reported due to the complexity arising from the diastereomeric mixture.

HRMS (ESI): calcd. for $C_{27}H_{27}FNaO_8$ $[M+Na]^+$: 521.1582; found: 521.1570.

Synthesis of acetal 2-*epi*-39

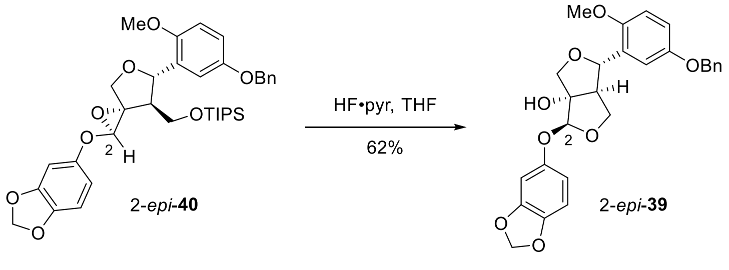

To a solution of epoxide 2-*epi*-**40** (40 mg, 63 µmol, 1 equiv) in tetrahydrofuran (1 mL) was added hydrogen fluoride-pyridine complex (54 µL, 2.2 mmol, 35 equiv) and the resulting solution was stirred for 16 h. The solution was poured into a vigorously stirred saturated aqueous sodium bicarbonate solution (20 mL). The mixture was extracted with ethyl acetate (2 × 10 mL). The organic solution was dried over sodium sulfate and filtered. The filtrate was concentrated under reduced pressure and the residue was purified by flash

chromatography on silica gel (20% ethyl acetate in petroleum ether) which afforded acetal 2-*epi*-**39** as a colorless oil (19 mg, 39 µmol, 62%).

The analytical data for acetal 2-*epi*-**39** matched those obtained before (see above).

Comparison of Synthetic Compounds with Literature

Haedoxan A (1a):

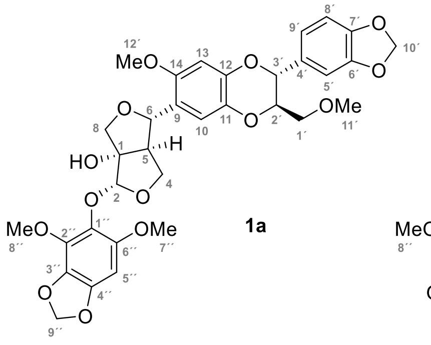

1a

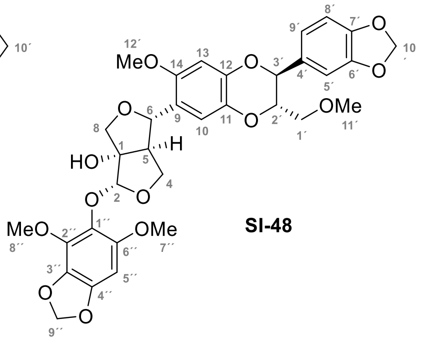

SI-48

^{1}H NMR (400 MHz, $CDCl_3$):

Position	Natural **1a**[32]	Synthetic **1a**	**SI-48**	Δ*δ* (**1a**)	Δ*δ* (**SI-48**)
2	5.24 (s, 1H)	5.24 (s, 1H)	5.24 (s, 1H)	0.00	0.00
4	4.57 (dd, *J* = 9, 7 Hz, 1H),<br>4.05 (dd, *J* = 9, 2 Hz, 1H)	4.57 (dd, *J* = 9.2, 7.4 Hz, 1H),<br>4.04 (dd, *J* = 9.2, 2.6 Hz, 1H)	4.58 (dd, *J* = 9.2, 7.4 Hz, 1H),<br>4.06 (dd, *J* = 9.1, 2.4 Hz, 1H)	0.00<br>0.01	0.01<br>0.01
5	2.64 (m, 1H)	2.67 – 2.61 (m, 1H)	2.68 – 2.64 (m, 1H)	0.00[a]	0.02[a]
6	4.94 (d, *J* = 6 Hz, 1H)	4.93 (d, *J* = 5.9 Hz, 1H)	4.90 (d, *J* = 6.1 Hz, 1H)	0.00	0.04
8	4.30 (d, *J* = 10 Hz, 1H),<br>3.73 (d, *J* = 10 Hz, 1H)	4.30 (d, *J* = 9.6 Hz, 1H),<br>3.75 – 3.71 (m, 1H)	4.31 (d, *J* = 9.6 Hz, 1H),<br>3.75 – 3.71 (m, 1H)	0.00<br>0.00[a]	0.01<br>0.00[a]
10	7.31 (s, 1H)	7.31 (s, 1H)	7.31 (s, 1H)	0.00	0.00
13	6.49 (s, 1H)	6.49 (s, 1H)	6.49 (s, 1H)	0.00	0.00
1´	3.52 (dd, *J* = 11, 2 Hz, 1H),<br>3.30 (dd, *J* = 11, 4 Hz, 1H)	3.58 (dd, *J* = 10.9, 2.5 Hz, 1H),<br>3.30 (dd, *J* = 10.9, 3.8 Hz, 1H)	3.55 (dd, *J* = 10.9, 2.6 Hz, 1H),<br>3.32 (dd, *J* = 11.0, 4.0 Hz, 1H)	0.06[b]<br>0.00	0.03<br>0.02
2´	4.00 (m, 1H)	4.02 – 3.96 (m, 1H)	4.04 – 3.98 (m, 1H)	0.01[a]	0.02[a]
3´	4.99 (d, *J* = 8 Hz, 1H)	4.99 (d, *J* = 7.9 Hz, 1H)	4.97 (d, *J* = 7.9 Hz, 1H)	0.00	0.02
5´	6.96 – 6.79 (m, 3H)	6.94 – 6.88 (m, 2H)	6.93 – 6.87 (m, 2H)	-	-
8´	6.96 – 6.79 (m, 3H)	6.84 (d, *J* = 8.6 Hz, 1H)	6.84 (d, *J* = 8.6 Hz, 1H)	-	-
9´	6.96 – 6.79 (m, 3H)	6.94 – 6.88 (m, 2H)	6.93 – 6.87 (m, 2H)	-	-
10´	5.99 (t, *J* = 1.5 Hz, 2H)	5.99 (s, 2H)	5.99 (s, 2H)	0.00	0.00
11´	3.35 (s, 3H)	3.35 (s, 3H)	3.35 (s, 3H)	0.00	0.00
12´	3.73 (s, 3H)	3.73 (s, 3H)	3.73 (s, 3H)	0.00	0.00
5´´	6.29 (s, 1H)	6.29 (s, 1H)	6.29 (s, 1H)	0.00	0.00
7´´	3.78 (s, 3H)	3.79 (s, 3H)	3.78 (s, 3H)	0.01	0.00
8´´	4.02 (s, 3H)	4.02 (s, 3H)	4.02 (s, 3H)	0.00	0.00
9´´	5.88 (d, *J* = 1.5 Hz, 1H),<br>5.87 (d, *J* = 1.5 Hz, 1H)	5.89 – 5.86 (m, 2H)	5.89 – 5.86 (m, 2H)	0.00[a]	0.00[a]
-OH	1.68 (s, 1H)	3.95 (d, *J* = 1.5 Hz, 1H)	3.97 (d, *J* = 1.5 Hz, 1H)	-	-

[a] The comparison was done by subtracting the literature value from the average of the interval boundries of the signal of synthetic **1a**.
[b] This discrepancy was caused by a mistake in ref. 32. Upon inspection of their attached COSY NMR spectrum it is evident that the shift of the C1´ hydrogens is 3.58 and 3.30 ppm.

^{13}C NMR (101 MHz, $CDCl_3$):

Position	Natural **1a**[32]	Synthetic **1a**	**SI-48**	$\Delta\delta$ (**1a**)[a]	$\Delta\delta$ (**SI-48**)[a]
1	92.3	92.6	92.5	0.3	0.2
2	106.3	106.6	106.6	0.3	0.3
4	71.7	71.9	72.0	0.2	0.3
5	57.8	58.0	58.1	0.2	0.3
6	83.8	84.0	84.2	0.2	0.4
8	77.5	77.7	77.7	0.2	0.2
9	123.0	123.3	123.4	0.3	0.4
10	107.5 (115.1)[b]	115.4	115.3	0.3	0.2
11	136.8	137.1	137.0	0.3	0.2
12	142.8	143.1	143.0	0.3	0.2
13	99.7	100.0	100.0	0.3	0.3
14	150.5	150.8	150.8	0.3	0.3
1′	71.2	71.4	71.4	0.2	0.2
2′	77.2	77.4	77.4	0.2	0.2
3′	76.4	76.7	76.7	0.3	0.3
4′	130.4	130.7	130.7	0.3	0.3
5′	108.3	108.6	108.6	0.3	0.3
6′	147.9	148.2	148.2	0.3	0.3
7′	147.9	148.2	148.2	0.3	0.3
8′	115.1 (107.5)[b]	107.8	107.8	0.3	0.3
9′	121.1	121.4	121.5	0.3	0.4
10′	101.2	101.4	101.4	0.2	0.2
11′	59.4	59.7	59.7	0.3	0.3
12′	55.8	55.9	55.9	0.1	0.1
1′′	130.9	131.2	131.2	0.3	0.4
2′′	137.9	138.2	138.2	0.3	0.3
3′′	131.0	131.3	131.3	0.3	0.3
4′′	147.5 (144.7)[b]	145.0	145.0	0.3	0.3
5′′	89.5	89.8	89.8	0.3	0.3
6′′	144.7 (147.5)[b]	147.8	147.8	0.3	0.3
7′′	56.7	57.1	57.1	0.4	0.4
8′′	59.9	60.3	60.3	0.4	0.4
9′′	101.1	101.2	101.2	0.1	0.1

[a] The consistent difference of 0.3 ppm was probably caused by a different chloroform resonance reference in ref. 32. [b] Incorrect assignment in ref. 32.

Haedoxan D (1b):

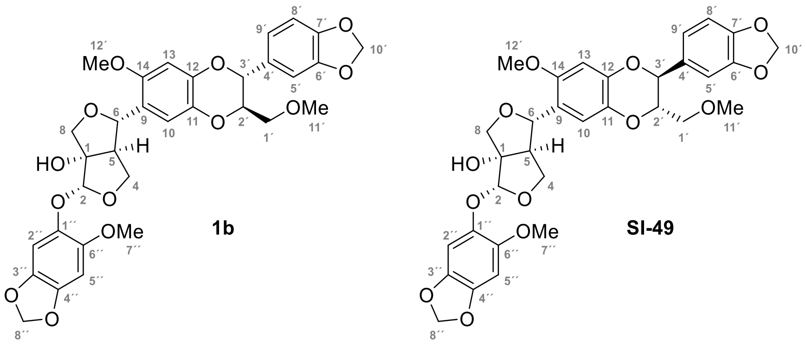

¹H NMR (400 MHz, CDCl₃):

Position	Literature **1b**[29]	Synthetic **1b**	**SI-49**	Δδ (**1b**)	Δδ (**SI-49**)
2	5.17 (s, 1H)	5.19 (s, 1H)	5.19 (s, 1H)	0.02	0.02
4	4.44 (dd, *J* = 10, 8 Hz, 1H),<br>4.15 – 3.87 (m, 2H)	4.45 (dd, *J* = 9.2, 7.4 Hz, 1H),<br>4.08 (dd, *J* = 9.2, 2.6 Hz, 1H)	4.46 (dd, *J* = 9.4, 7.4 Hz, 1H),<br>4.09 (dd, *J* = 9.5, 2.4 Hz, 1H)	0.01<br>-	0.02<br>-
5	2.64 (m, 1H)	2.67 – 2.61 (m, 1H)	2.69 – 2.63 (m, 1H)	0.00	0.02
6	4.92 (d, *J* = 6 Hz, 1H)	4.95 (d, *J* = 5.9 Hz, 1H)	4.92 (d, *J* = 5.8 Hz, 1H)	0.03	0.00
8	4.29 (d, *J* = 11 Hz, 1H),<br>3.74 (d, *J* = 11 Hz, 1H)	4.31 (d, *J* = 9.7 Hz, 1H),<br>3.75 – 3.71 (m, 1H)	4.32 (d, *J* = 10.1 Hz, 1H),<br>3.75 – 3.71 (m, 1H)	0.02<br>0.00	0.03<br>0.00
10	7.28 (s, 1H)	7.31 (s, 1H)	7.30 (s, 1H)	0.03	0.02
13	6.47 (s, 1H)	6.49 (s, 1H)	6.49 (s, 1H)	0.02	0.02
1′	3.58 (dd, *J* = 11, 3 Hz, 1H),<br>3.28 (dd, *J* = 11, 5 Hz, 1H)	3.58 (dd, *J* = 10.9, 2.5 Hz, 1H),<br>3.30 (dd, *J* = 10.9, 3.8 Hz, 1H)	3.55 (dd, *J* = 10.9, 2.5 Hz, 1H),<br>3.32 (dd, *J* = 10.9, 3.8 Hz, 1H)	0.00<br>0.02	0.03<br>0.04
2′	4.15 – 3.87 (m, 2H)	4.02 – 3.96 (m, 1H)	4.03 – 3.99 (m, 1H)	-	-
3′	4.96 (d, *J* = 8 Hz, 1H)	4.99 (d, *J* = 7.9 Hz, 1H)	4.96 (d, *J* = 7.9 Hz, 1H)	0.03	0.00
5′	6.98 – 6.80 (m, 3H)	6.93 – 6.88 (m, 2H)	6.92 – 6.87 (m, 2H)	-	-
8′	6.98 – 6.80 (m, 3H)	6.93 – 6.88 (m, 2H)	6.92 – 6.87 (m, 2H)	-	-
9′	6.98 – 6.80 (m, 3H)	6.84 (dd, *J* = 8.6, 1.5 Hz, 1H)	6.84 (dd, *J* = 8.6, 1.5 Hz, 1H)	-	-
10′	5.98 (s, 2H)	5.99 (s, 2H)	5.99 (s, 2H)	0.01	0.01
11′	3.34 (s, 3H)	3.35 (s, 3H)	3.35 (s, 3H)	0.01	0.01
12′	3.72 (s, 3H)	3.73 (s, 3H)	3.73 (s, 3H)	0.01	0.01
2′′	6.76 (s, 1H)	6.79 (s, 1H)	6.80 (s, 1H)	0.03	0.04
5′′	6.53 (s, 1H)	6.56 (s, 1H)	6.55 (s, 1H)	0.03	0.02
7′′	3.77 (s, 3H)	3.78 (s, 3H)	3.77 (s, 3H)	0.01	0.00
8′′	5.88 (s, 2H)	5.92 – 5.88 (m, 2H)	5.92 – 5.88 (m, 2H)	0.02	0.02
-OH	1.62 (s, 1H)	-	-	-	-

¹³C NMR data for Haedoxan D have never been reported.

Phrymarolin II (2a):

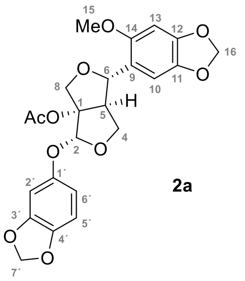

2a

^{1}H NMR (400 MHz, $CDCl_3$):

Position	Natural **2a**[40]	Synthetic **2a**	Δδ
2	5.70 (s, 1H)	5.70 (s, 1H)	0.00
4	4.29 (dd, *J* = 9.1, 6.9 Hz, 1H),<br>4.06 (dd, *J* = 9.2, 1.5 Hz, 1H)	4.29 (dd, *J* = 9.2, 6.8 Hz, 1H),<br>4.06 (d, *J* = 9.2 Hz, 1H)	0.00,<br>0.00
5	2.84 (t, *J* = 6.6, 1H)	2.84 (t, *J* = 7.0 Hz, 1H)	0.00
6	4.87 (d, *J* = 7.0 Hz, 1H)	4.87 (d, *J* = 7.0 Hz, 1H	0.00
8	4.60 (d, *J* = 11.1 Hz, 1H),<br>3.81 (d, *J* = 11.1 Hz, 1H)	4.60 (d, *J* = 11.1 Hz, 1H),<br>3.81 (dd, *J* = 11.1, 1.7 Hz, 1H)	0.00,<br>0.00
10	7.05 (s, 1H)	7.05 (s, 1H)	0.00
13	6.52 (s, 1H)	6.52 (s, 1H)	0.00
15	3.77 (s, 3H)	3.77 (s, 3H)	0.00
16	5.93 (s, 2H)	5.93 (s, 2H)	0.00
2´	6.58 (d, *J* = 2.3 Hz, 1H)	6.58 (d, *J* = 2.3 Hz, 1H)	0.00
5´	6.69 (d, *J* = 8.4 Hz, 1H)	6.69 (dd, *J* = 8.5, 1.7 Hz, 1H)	0.00
6´	6.49 (dd, *J* = 8.4, 2.3 Hz, 1H)	6.49 (dt, *J* = 8.5, 2.2 Hz, 1H)	0.00
7´	5.92 (s, 2H)	5.92 (s, 2H)	0.00
-OCO*CH₃*	2.13 (s, 3H)	2.14 (s, 3H)	0.01

^{13}C NMR (101 MHz, $CDCl_3$):

Position	Natural **2a**[40]	Synthetic **2a**	$\Delta\delta$ (ppm)
1	96.6	96.6	0.0
2	101.4	101.4	0.0
4	68.7	68.8	0.1
5	56.7	56.6	0.1
6	83.1	83.0	0.1
8	75.8	75.8	0.0
9	121.5	121.5	0.0
10	103.2	103.2	0.0
11	143.5	143.5	0.0
12	147.7	147.7	0.0
13	94.4	94.4	0.0
14	151.6	151.6	0.0
15	56.4	56.4	0.0
16	101.4	101.4	0.0
1′	152.0	152.0	0.0
2′	106.5	106.5	0.0
3′	148.2	148.2	0.0
4′	141.6	141.5	0.1
5′	108.2	108.2	0.0
6′	110.6	110.6	0.0
7′	101.4	101.4	0.0
-O*C*OCH$_3$	170.9	170.9	0.0
-OCO*CH$_3$*	21.2	21.3	0.1

Phrymarolin I (2b):

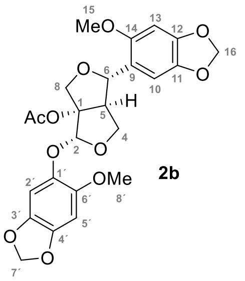

2b

^{1}H NMR (400 MHz, $CDCl_3$):

Position	Natural **2b**[40]	Synthetic **2b**	Δ*δ*
2	5.68 (s, 1H)	5.68 (s, 1H)	0.00
4	4.40 (d, *J* = 9.1, 7.0 Hz, 1H),<br>4.05 (d, *J* = 9.1, 1.8 Hz, 1H)	4.40 (dd, *J* = 9.2, 6.9 Hz, 1H),<br>4.05 (dd, *J* = 9.1, 1.9 Hz, 1H)	0.00,<br>0.00
5	2.90 (t, *J* = 6.4 Hz, 1H)	2.94 – 2.86 (m, 1H)	0.00
6	4.88 (d, *J* = 6.9 Hz, 1H)	4.88 (d, *J* = 6.9 Hz, 1H)	0.00
8	4.61 (d, *J* = 11.1 Hz, 1H),<br>3.81 (d, *J* = 11.1 Hz, 1H)	4.62 (dt, *J* = 11.1, 0.8 Hz, 1H),<br>3.81 (d, *J* = 11.1 Hz, 1H)	0.01,<br>0.00
10	7.05 (s, 1H)	7.05 (s, 1H)	0.00
13	6.52 (s, 2H)	6.54 – 6.50 (m, 2H)	0.00
15	3.74 (s, 3H)	3.74 (s, 3H)	0.00
16	5.87 (s, 2H)	5.90 – 5.84 (m, 2H)	0.00
2′	6.83 (s, 1H)	6.83 (s, 1H)	0.00
5′	6.52 (s, 2H)	6.54 – 6.50 (m, 2H)	0.00
7′	5.92 (s, 2H)	5.96 – 5.90 (m, 2H)	0.01
8′	3.77 (s, 3H)	3.77 (s, 3H)	0.00
-OCO*CH₃*	2.13 (s, 3H)	2.13 (s, 3H)	0.00

^{13}C NMR (101 MHz, $CDCl_3$):

Position	Natural **2b**[40]	Synthetic **2b**	$\Delta\delta$ (ppm)
1	96.6	96.6	0.0
2	101.8	101.9	0.1
4	69.0	69.0	0.0
5	57.1	57.1	0.0
6	83.1	83.1	0.0
8	75.8	75.8	0.0
9	121.5	121.6	0.1
10	104.0	104.1	0.1
11	140.4	140.5	0.1
12	145.8	145.8	0.0
13	96.2	96.3	0.1
14	151.6	151.6	0.0
15	56.4	56.4	0.0
16	101.3	101.3	0.0
1′	147.6	147.6	0.0
2′	106.5	106.5	0.0
3′	141.5	141.5	0.0
4′	140.9	141.0	0.1
5′	94.4	94.4	0.0
6′	143.0	143.0	0.0
7′	101.4	101.4	0.0
8′	56.4	56.4	0.0
-O*C*OCH$_3$	171.0	171.0	0.0
-OCO*CH$_3$*	21.2	21.3	0.1

References

1) Jiang, J.-A.; Chen, Ch.; Guo, Y.; Liao, D.-H.; Pan, X.-D.; Ji, Y.-F. A highly efficient approach to vanillin starting from 4-cresol. *Green Chem.*, **2014**, *16*, 2807–2814.

2) Novak, Z.; Timari, G.; Kotschy, A. The first total synthesis of Cicerfuran utilizing a one-pot synthesis of hydroxylated benzofurans. *Tetrahedron* **2003**, *59*, 7509–7513.

3) Pisella, G.; Gagnebin, A.; Waser, J. Copper-Catalyzed Oxyvinylation of Diazo Compounds *Org. Lett.* **2020**, *22*, 3884–3889.

4) Eichenauer, N. C.; Nordschild, A. C. M.; Bischop, M.; Schumacher, D.; Mackwitz, M. K. W.; Tschersich, R.; Wilhelm, T.; Pietruszka, J. Total Synthesis of Solandelactones A and B. *Eur. J. Org. Chem.* **2015**, 5620–5632.

5) Angle, S. R.; Choi, I.; Tham, F. S. Stereoselective Synthesis of 3-Alkyl-2-aryltetrahydrofuran-4-ols: Total Synthesis of (±)-Paulownin. *J. Org. Chem.* **2008**, *73*, 6268–6278.

6) Chi, Y.; Zhou, H.; He, H.-W.; Ma, Y.-D.; Li, B.; Xu, D.; Gao, J.-M.; Xu, G. Total Synthesis and Anti-Tobacco Mosaic Virus Activity of the Furofuran Lignan (±)-Phrymarolin II and Its Analogues. *J. Nat. Prod.* **2021**, *84*, 2937–2944.

7) Ishibashi, F.; Taniguchi, E. Syntheses of (±)-Phrymarolin II and Its Stereoisomers. *Agric. Biol. Chem.* **1986**, *50*, 3119–3125.

8) Okazaki, M.; Ishibashi, F.; Shuto, Y.; Taniguchi, E. Total Synthesis of (+)-Phrymarolin I from (+)-Malic Acid. *Biosci. Biotech. Biochem.* **1997**, *61*, 660–663.

9) Toshiyuki, K.; Kunitomo, A.; Yoshihito, T.; Kunio, S.; Ayumi, T.; Masatoshi, K. Bi-Aryl Compound Having Immunosuppressive Activity, WO2005014525A2, February 17, 2005.

10) Dodsworth, D. J.; Calcagno, M.-P.; Ehrmann, E. U.; Devadas, B.; Sammes, P. G. A new route to anthraquinones. *J. Chem. Soc., Perkin Trans. 1* **1981**, 2120–2124.

11) Hu, D. X.; Shibuya, G. M.; Burns, N. Z. *J. Am. Chem. Soc.* **2013**, *135*, 12960- 12963.

12) Ju, D.; Kang, J.; Song, Z. Enantioselective Intramolecular Oxidative Aminoacetoxylation from Aryl-Substituted Alkene via Chiral Triazole-oxazoline Palladium Complexes. *Org. Lett.* **2024**, *26*, 45, 9637–9642.

13) Chatterjee, P. N.; Roy, S. Allylic activation across an Ir-Sn heterobimethallic catalyst: nucleopholic substitution and disproportionation of allylic alcohol. *Tetrahedron* **2012**, *68*, 3776–3785.

14) Zhao, Y.; Ku, C.-F.; Xu, X.-Y.; Tsang, N.-Y.; Zhu, Y.; Zhao, C.-L.; Liu, K.-L.; Rong, L.; Zhang, H.-J. Stable Axially Chiral Isomers of Arylnaphthalene Lignan Glycosides with Antiviral Potential Discovered from Justicia Procumbens. *J. Org. Chem.* **2021**, *86*, 5568–5583.

15) Yang, M.-H.; Orsi, D. L.; Altman, R. A. Ligand-Controlled Regiodivergent Palladium-Catalyzed Decarboxylative Allylation Reaction to Access α,α-Difluoroketones. *Angew. Chem. Int. Ed.* **2015**, *54*, 2361–2365.

16) Holla, H.; Jenkins, I. D.; Neve, J.; Pouwer, R. H.; Pham, N.; Teague, S. J.; Quinn, R. J. Synthesis of melicodenines C, D and E. *Tetrahedron Lett.* **2012**, *53*, 7101–7103.

17) Wang, B.; Wang, L.; Peng, Y.; Pang, Y.; Xiao, H.; Wang, X. The First Total Synthesis of (±)-Methyl Salvianolate A Using a Convergent Strategy. *Molecules* **2019**, *24*, 999.

18) Chavarria, D.; Benefito, S.; Soares, P.; Lima, C.; Garrido, J.; Serrao, P.; Soares-da Silva, P.; Remiao, F.; Oliveira, P. J.; Borges, F. Boosting caffeic acid performance as antioxidant and monoamine oxidase B/catechol-O-methyltransferase inhibitor. *Eur. J. Med. Chem.* **2022**, *243*, 114740.

19) Suh, S. M.; Jambu, S.; Chin, M. T.; Diao, T. Selective Cleavege of Lignin Model Compounds via a Reverse Biosynthesis Mechanism. *Org. Lett.* **2023**, *25*, 4792–4796.

20) Qin, Y.; Zhang, L.; Lv, J.; Luo, S.; Cheng, J.-P. Bioinspired Organocatalytic Aerobic C-H Oxidation of Amines with an *ortho*-Quinone Catalyst. *Org. Lett.* **2015**, *17*, 1469–1472.

21) Khan, J.; Taneja, N.; Yadav, N.; Hazra, C. K. Silane-mediated, facile C-H and N-H methylation using formaldehyde. *Chem. Commun.* **2024**, *60*, 11367–11370.

22) Saito, S.; Kawabata, J. Effects of electron-withdrawing substituents of DPPH radical scavenging reactions of protocatechuic acid and its analogues in alcoholic solvents. *Tetrahedron* **2005**, *61*, 8101–8108.

23) Wu, Z.; Wei, F.; Wan, B.; Zhang, Y. Pd-Catalyzed *ipso,meta*-Dimethylation of *ortho*-Substituted Iodoarenes via a Base Controlled C-H Activation Cascade with Dimethyl Carbonate as the Methyl Source. *J. Am. Chem. Soc.* **2021**, *143*, 4524–4530.

24) Luo, T.; Li, Z.; Deng, X.-M.; Jiang, K.; Liu, D.; Thang, H.-H.; Shi, T.; Liu, L.-Y.; Wen, H.-X.; Li, Q.-E.; Wang, Z. Isolation, synthesis and bioactivity evaluation of isoquinoline alkaloids from *Corydalis hendersonii* Hemsl. Against gastric cancer *in vitro* and *in vivo*. *Bioorg. Med. Chem.* **2022**, *60*, 116705.

25) Tomita, M.; Aoyagi, Y. Cupric Oxide As An Efficient Catalyst For The Methylenation of Catechols. *Chem. Pharm. Bull.* **1968**, *16*, 523 – 526.

26) Ono, E.; Nakai, M.; Fukui, Y.; Tomimori, N.; Fukuchi-Mizutani, M.; Saito, M.; Satake, H.; Tanaka, T.; Katsuta, M.; Umezawa, T.; Tanaka, Y. Formation of two methylenedioxy bridges by a Sesamum CYP81Q protein yielding a furofuran lignan (+)-sesamin. *PNAS* **2006**, *103*, 10116–10121.

27) Pereira, M. M.; Dias, L. D.; Calvete, M. J. F. Metalloporphyrins: Bioinspired Oxidation Catalysts. *ACS Catal.* **2018**, *8*, 10784–10808.

28) Thommen, C.; Neuburger, M.; Gademann, K. Collective Syntheses of Icetexane Natural Products Based on Biogenetic Hypotheses. *Chem. Eur. J.* **2017**, *23*, 120 – 127.

29) Ishibashi, F.; Taniguchi, E. Synthesis and Absolute Configuration of the Insectidical Sesquilignan (+)-Haedoxan A. *Phytochemistry* **1998**, *49*, 613–622.

30) Devakumar, C.; Saxena, V. S.; Mukerjee, S. K. New Sesamol Ethers as Pyrethrum Synergists. *Agric. Biol. Chem.* **1985**, *49*, 725–730.

31) Estrada, M. A.; Zhao, X.; Lorent, K.; Kriegermeier, A.; Nagao, S. A.; Berritt, S.; Wells, R. G.; Pack, M.; Winkler, J. D. Synthesis and Structure–Activity Relationship Study of Biliatresone, a Plant Isoflavonoid That Causes Biliary Atresia. *ACS Med. Chem. Lett.* **2018**, *9*, 61–64.

32) Taniguchi, E.; Imamura, K.; Ishibashi, F.; Matsui, T.; Nishio, A. Structure of the Novel Insecticidal Sesquilignan, Haedoxan A. *Agric. Biol. Chem.* **1989**, *53*, 631–643.

33) Klövekorn, P.; Pfaffenrot, B.; Juchum, M.; Selig, R.; Albrecht, W.; Zender, L.; Laufer, S. A. From off- to on-target: New BRAF-inhibitor-template-derived compounds selectively targeting mitogen activated protein kinase 4 (MKK4). *Eur. J. Med. Chem.* **2021**, *210*, 112963.

34) Wu, J.; Kozlowski, M. C. Visible-Light-Induced Oxidative Coupling of Phenols and Alkenylphenols with a Recyclable, Solid Photocatalyst. *Org. Lett.* **2023**, *25*, 907–911.

35) Borzatta, V.; Capparella, E.; Moroni, L.; Moores, G.; Philippou, D. Substituted alkynyl phenoxy compounds as new synergists in pesticidal compositions. WO2011020848A1, February 2, 2011.

36) Hellberg, J.; Dahlstedt, E.; Pelcman, M. E. Synthesis of annulated dioxins as electron-rich donors for cation radical salts. *Tetrahedron,* **2004**, *60*, 8899–8912.

37) Wawrzinek, R.; Ziomkowska, J.; Heuveling, J.; Mertens, M.; Herrmann, A.; Schneider, E.; Wessig, P. BDB Dyes as Fluorescence Lifetime Probes to Study Conformational Changes in Proteins. *Chem. Eur. J.* **2013**, *19*, 17349–17357.

38) Majerus, S. L.; Alibhai, N.; Tripathy, S.; Durst, T. New syntheses of dillapiol [4,5-dimethoxy-6-(2-propenyl)-1,3-benzodioxole], its 4-methylthio and other analogs. *Can. J. Chem.* **2000**, *78*, 1345–1355.

39) *Org. Synth*. **2013**, *90*, 350–357.

40) Li, Y.; Wei, J.; Fang, J.; Lv, W.; Ji, Y.; Aioub, A. A. A.; Zhang, J.; Hu, Z. Insecticidal Activity of Four Lignans Isolated from *Phryma leptostachya*. *Molecules* **2019**, *24*, 1976.

NMR Spectra

^{1}H NMR (400 MHz, $CDCl_3$) of methyl ether **26b**:

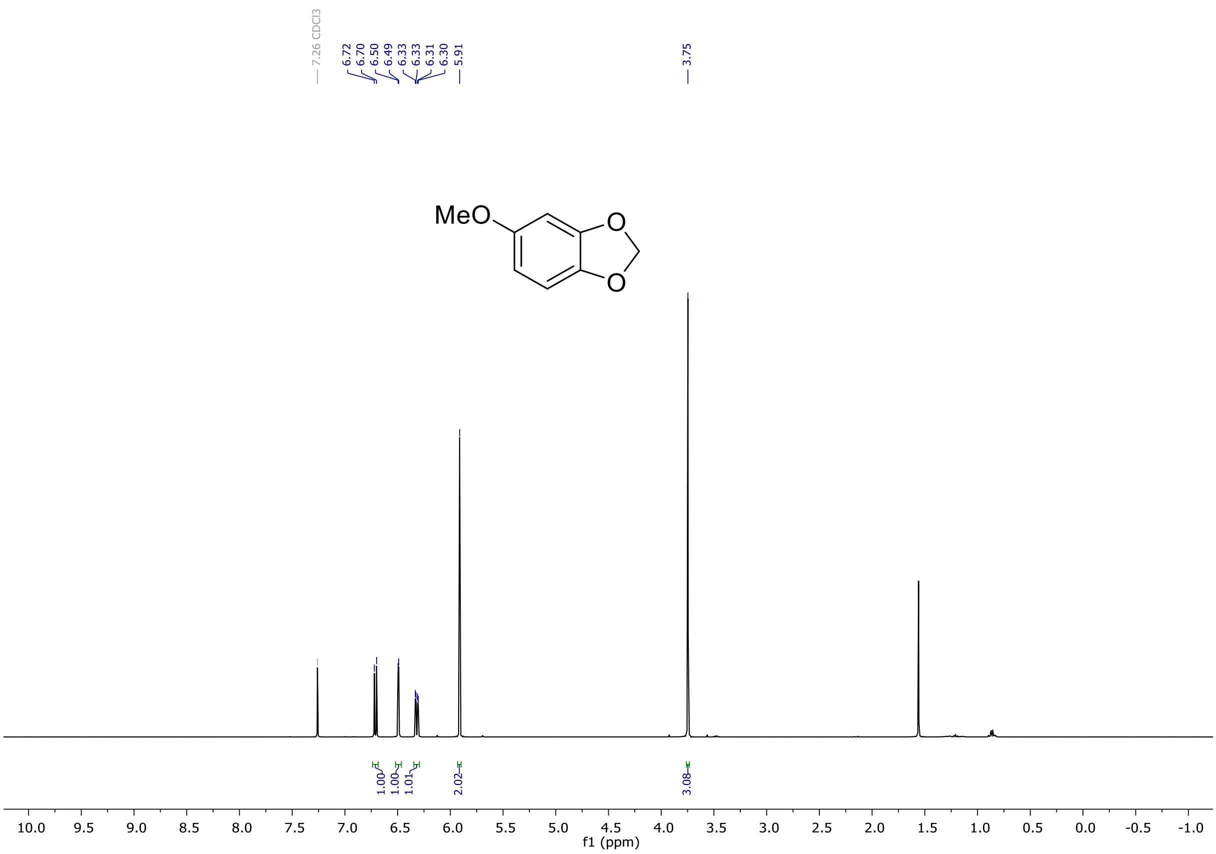

^{1}H NMR (400 MHz, $CDCl_3$) of aryl bromide **22**:

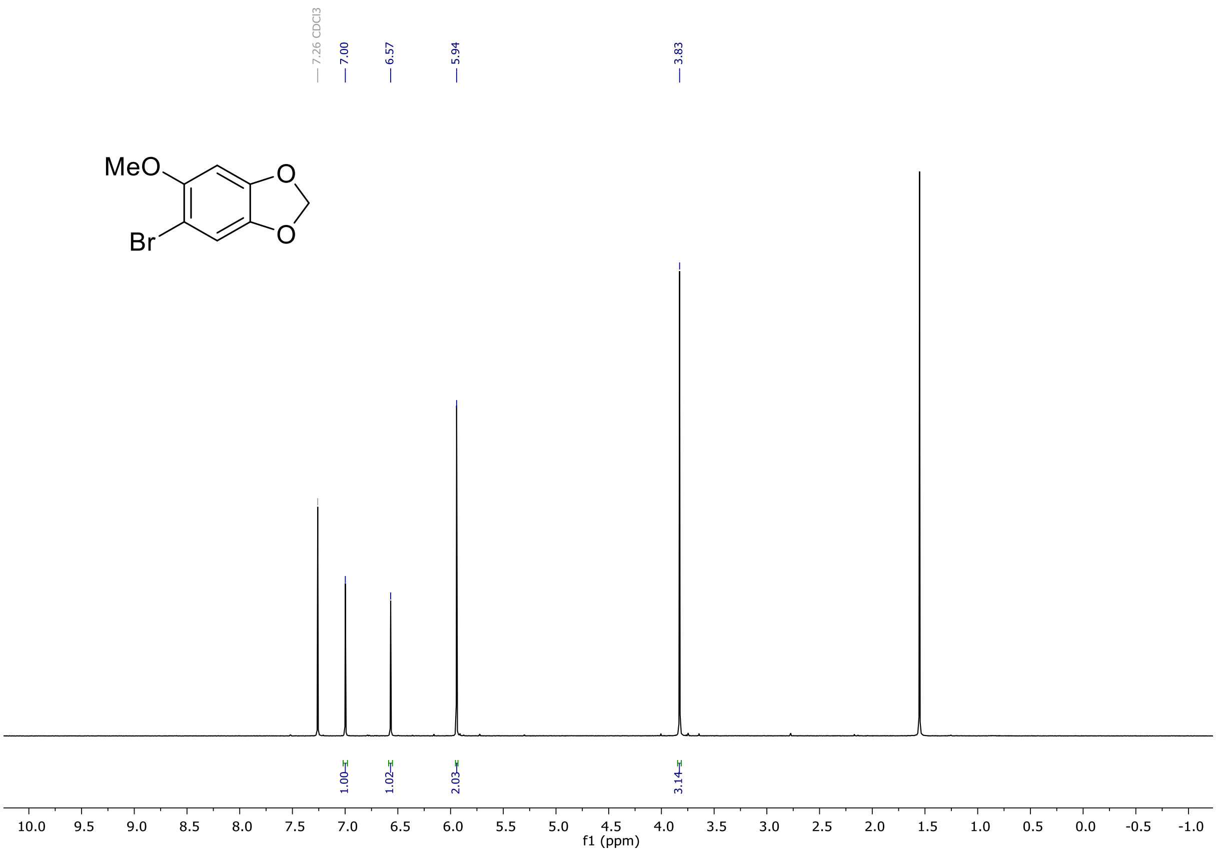

^{1}H NMR (400 MHz, $CDCl_3$) of silyl ether **SI-2**:

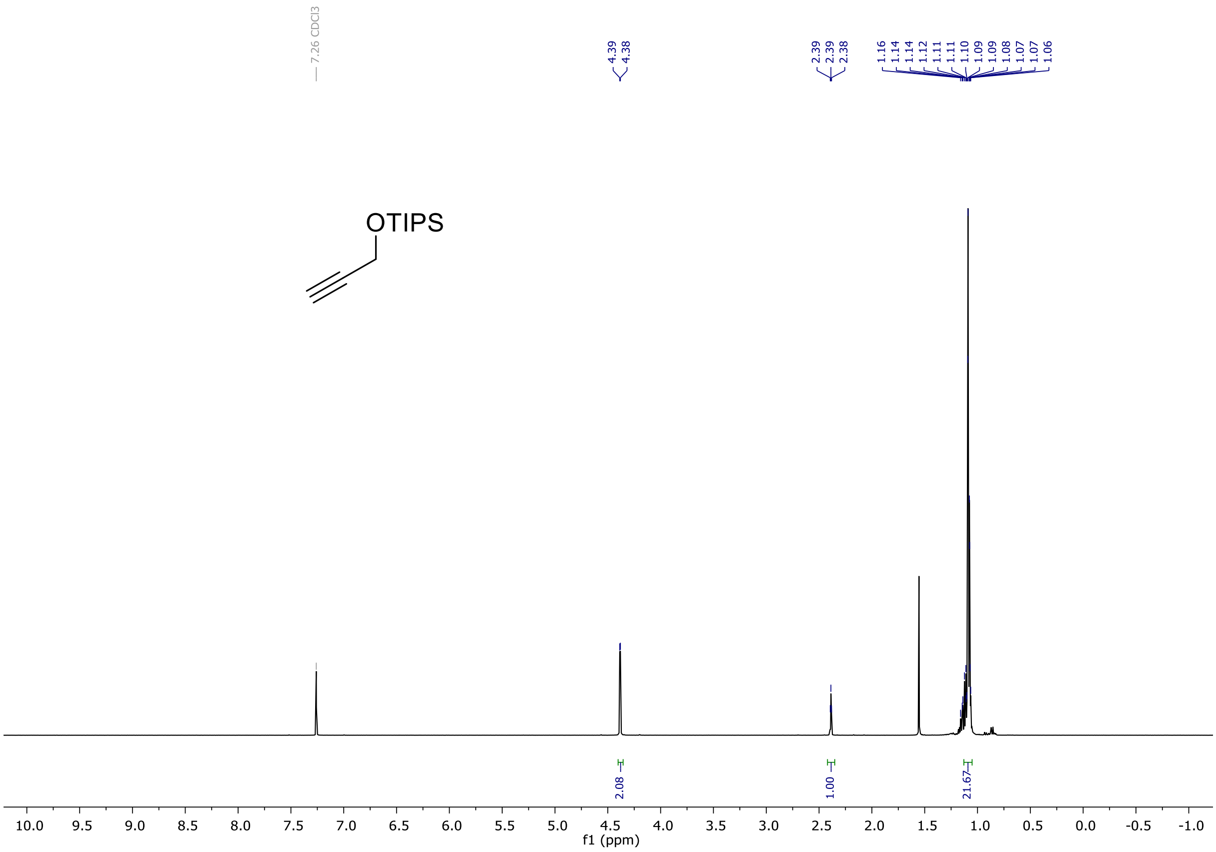

^{1}H NMR (400 MHz, $CDCl_3$) of boronic ester **21**:

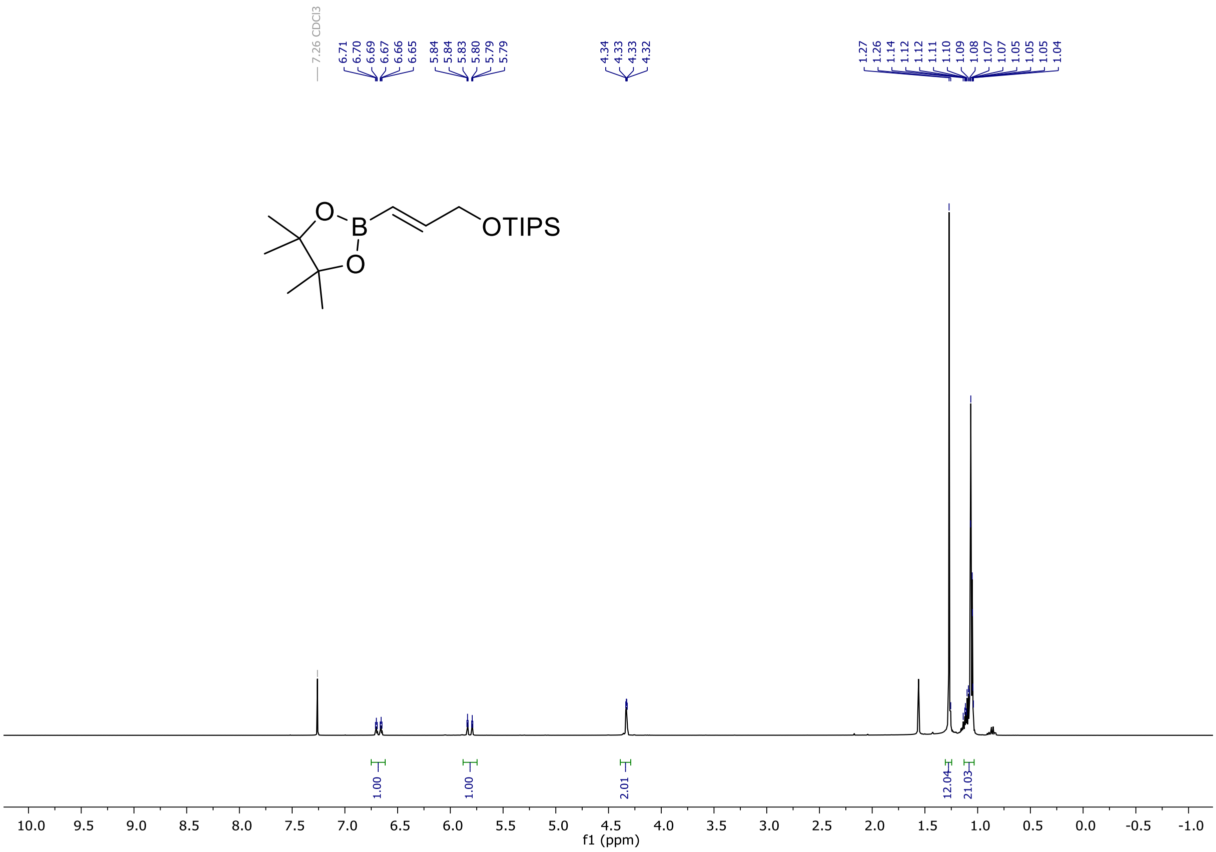

^{13}C NMR (101 MHz, $CDCl_3$) of boronic ester **21:**

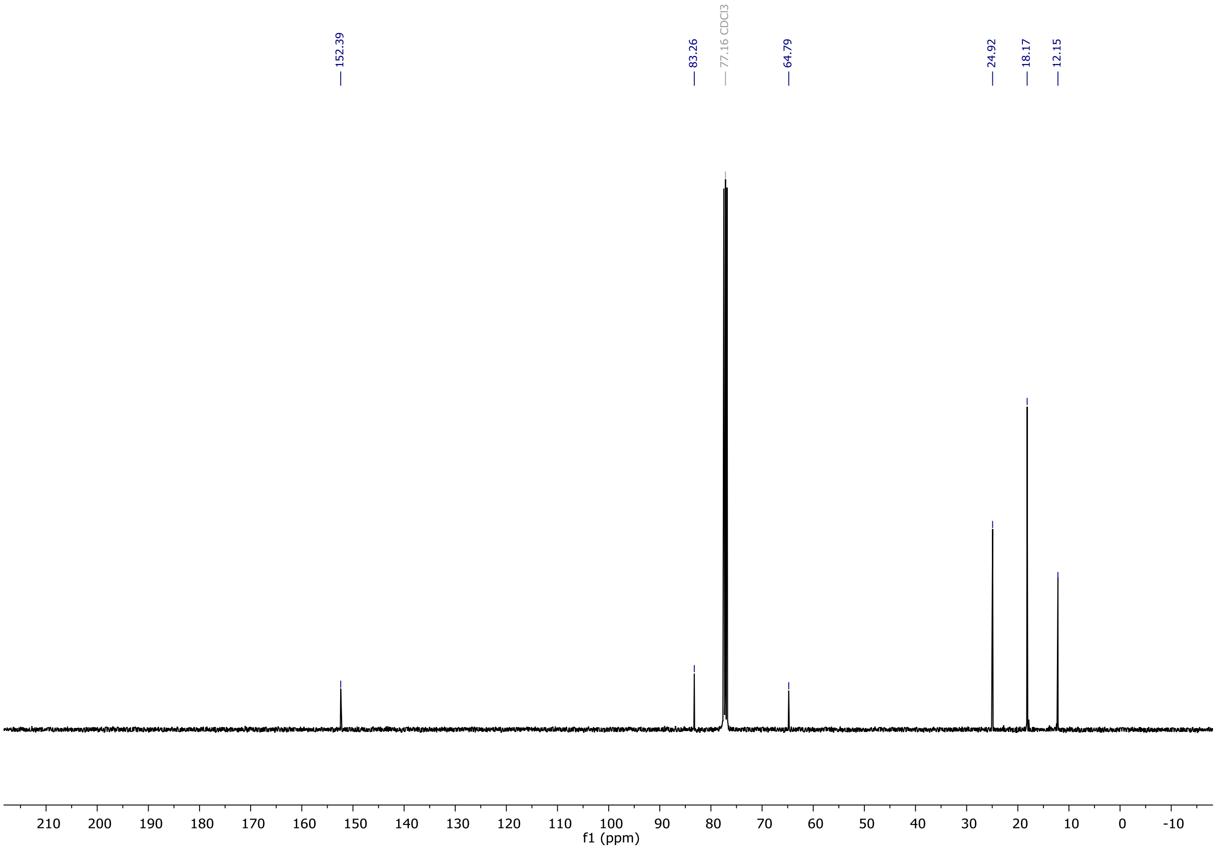

^{1}H NMR (400 MHz, $CDCl_3$) of styrene **18**:

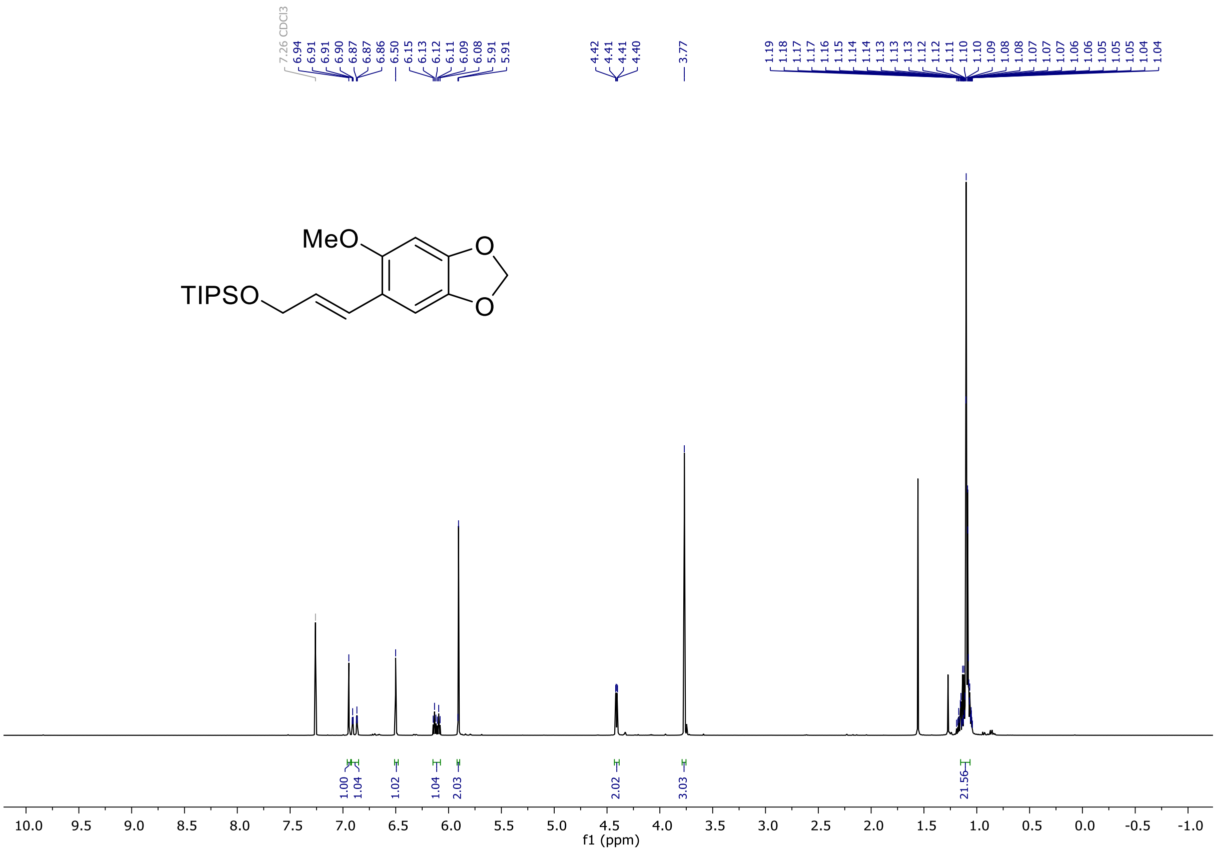

^{13}C NMR (101 MHz, $CDCl_3$) of styrene **18:**

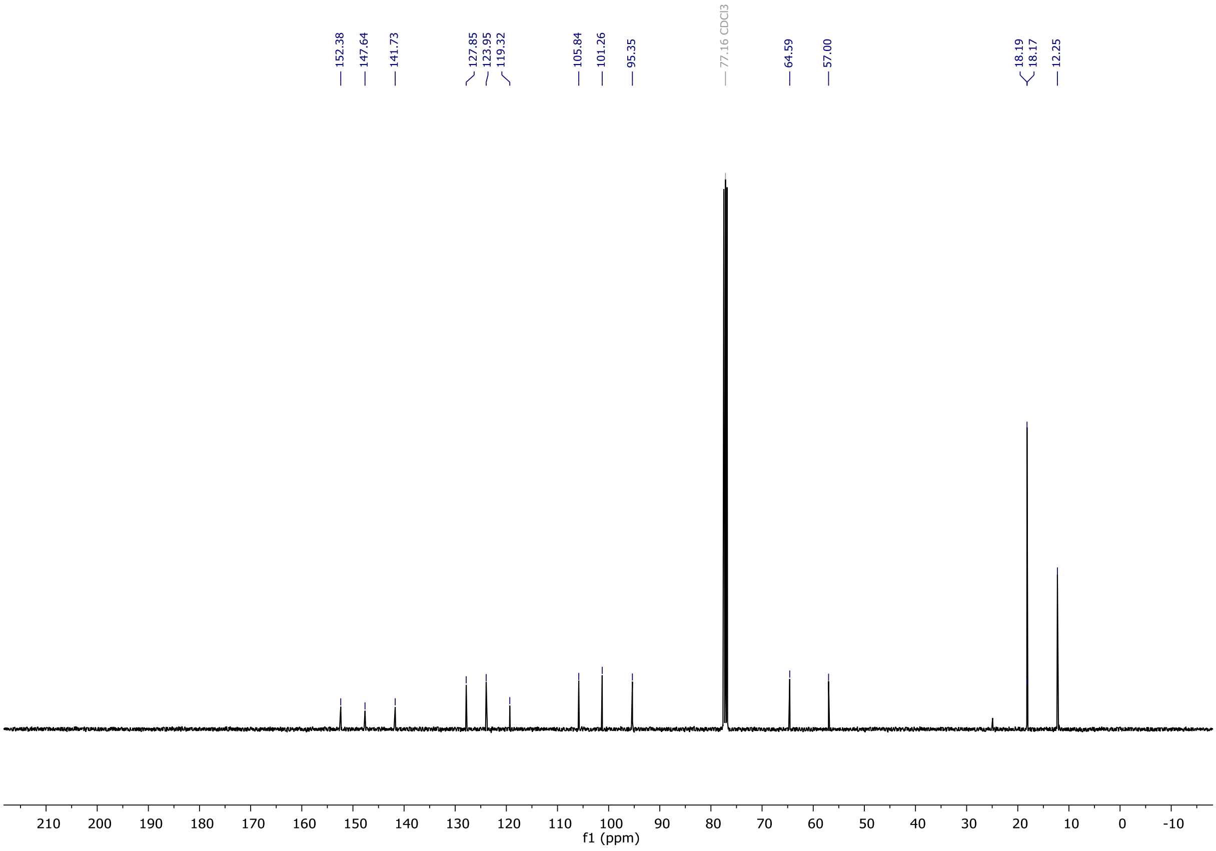

^{1}H NMR (400 MHz, $CDCl_3$) of silyl ether **SI-3**:

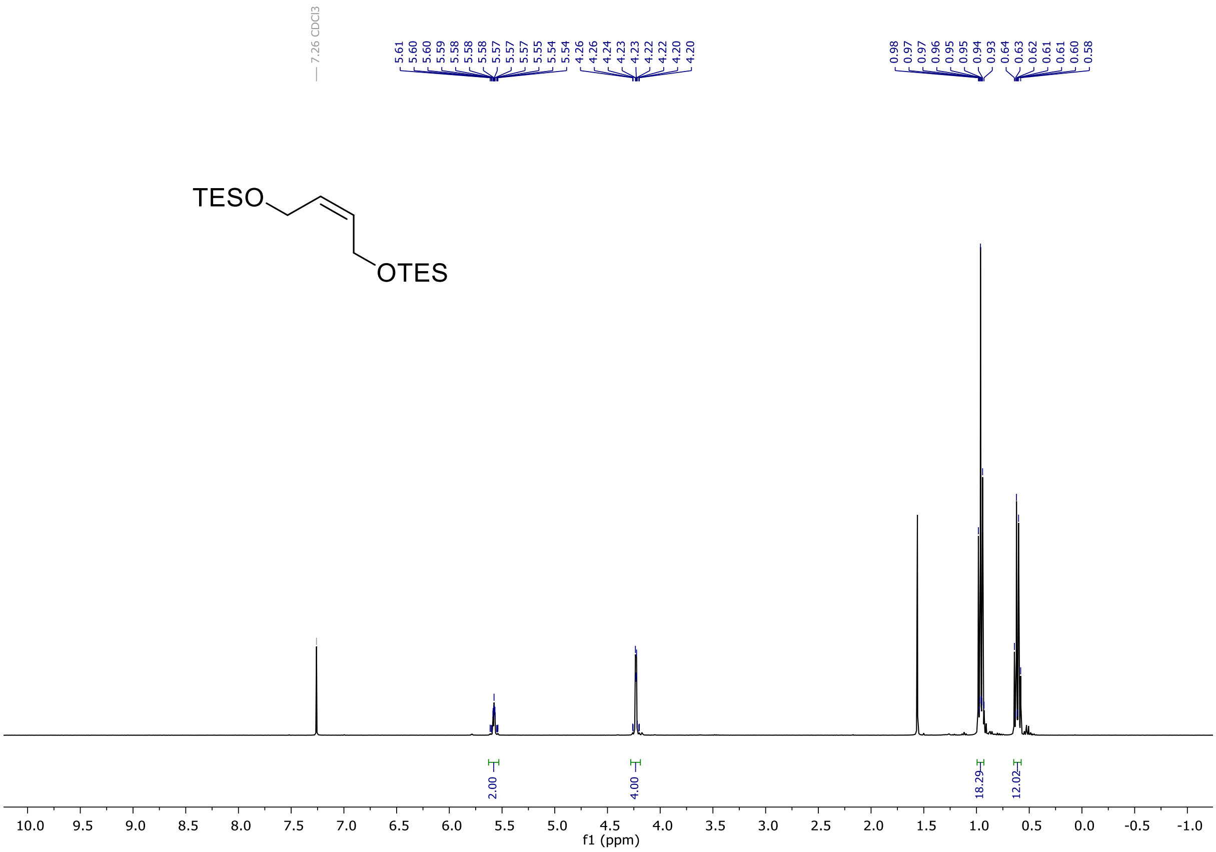

^{1}H NMR (400 MHz, $CDCl_3$) of aldehyde **20**:

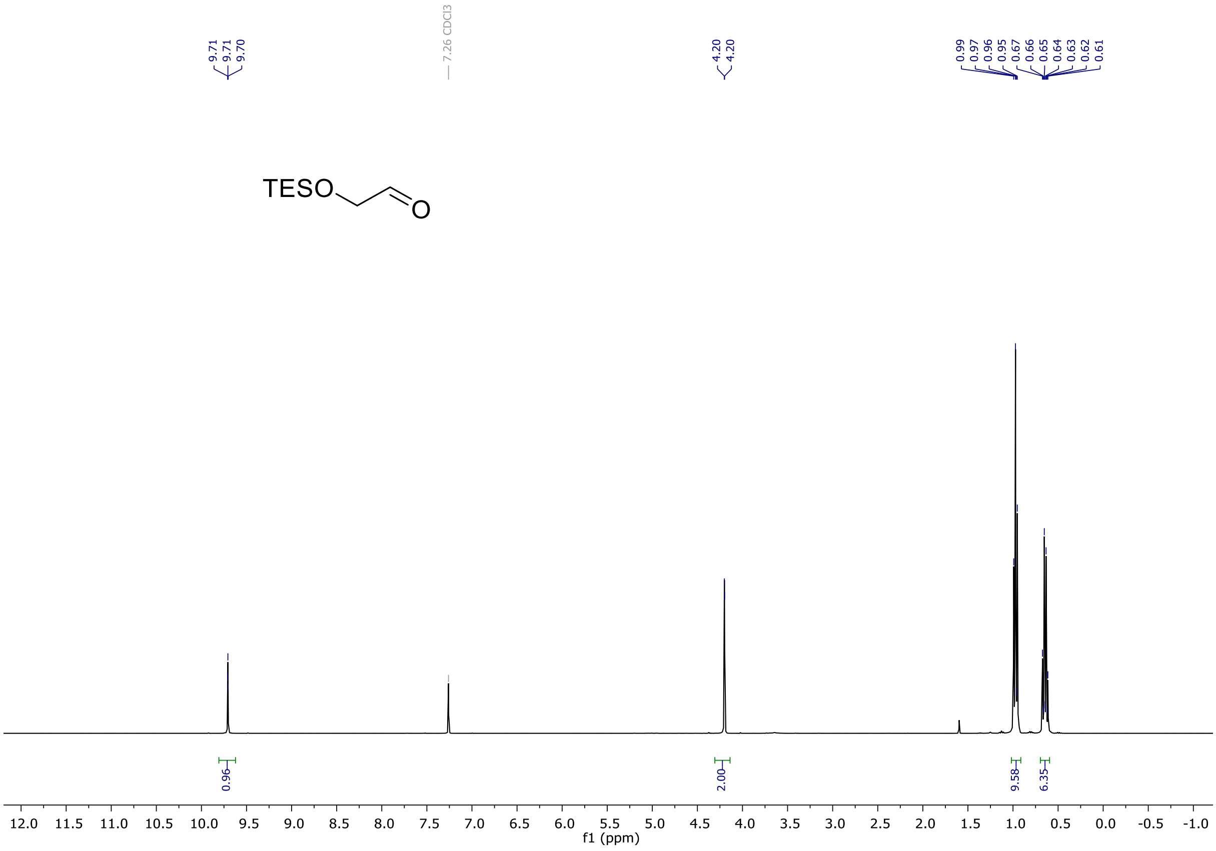

^{1}H NMR (400 MHz, $CDCl_3$) of ketone **24**:

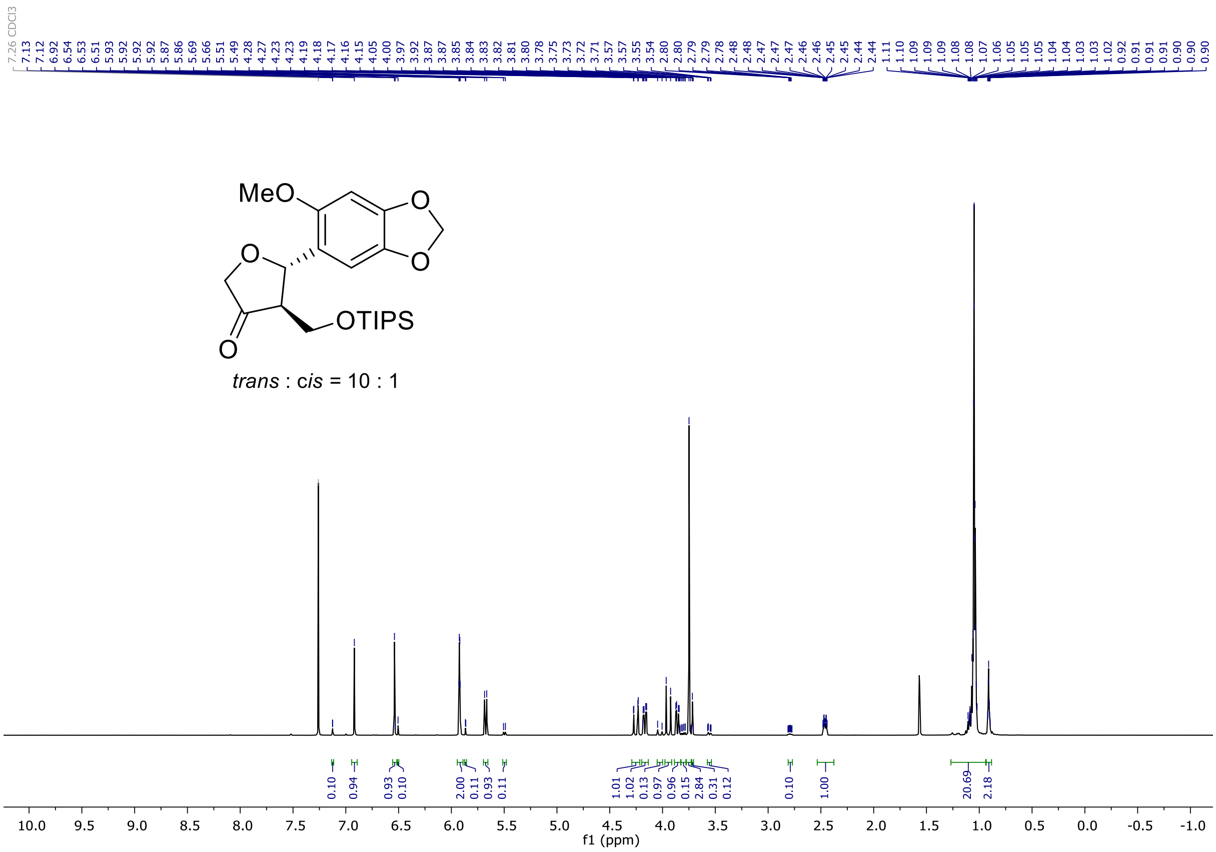

^{13}C NMR (101 MHz, $CDCl_3$) of ketone **24:**

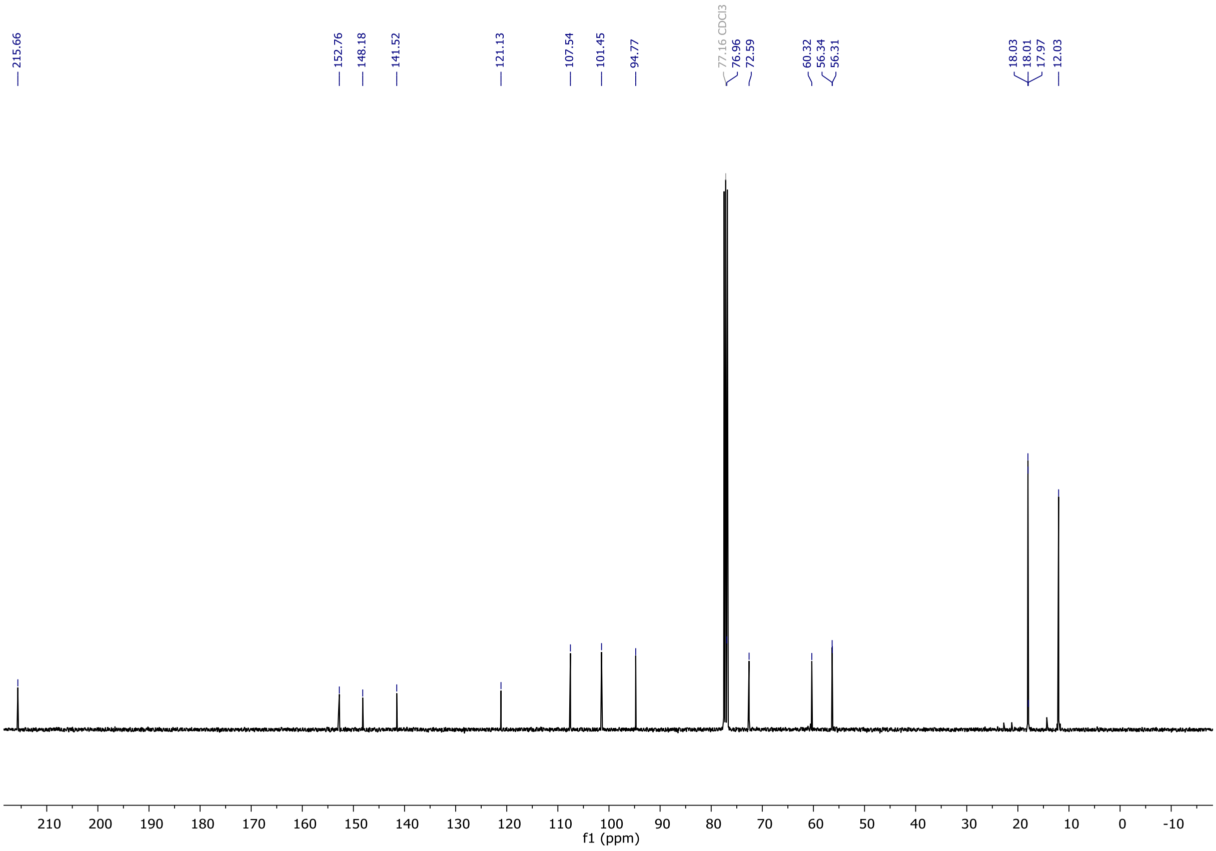

^{1}H NMR (400 MHz, $CDCl_3$) of quinone **25a**:

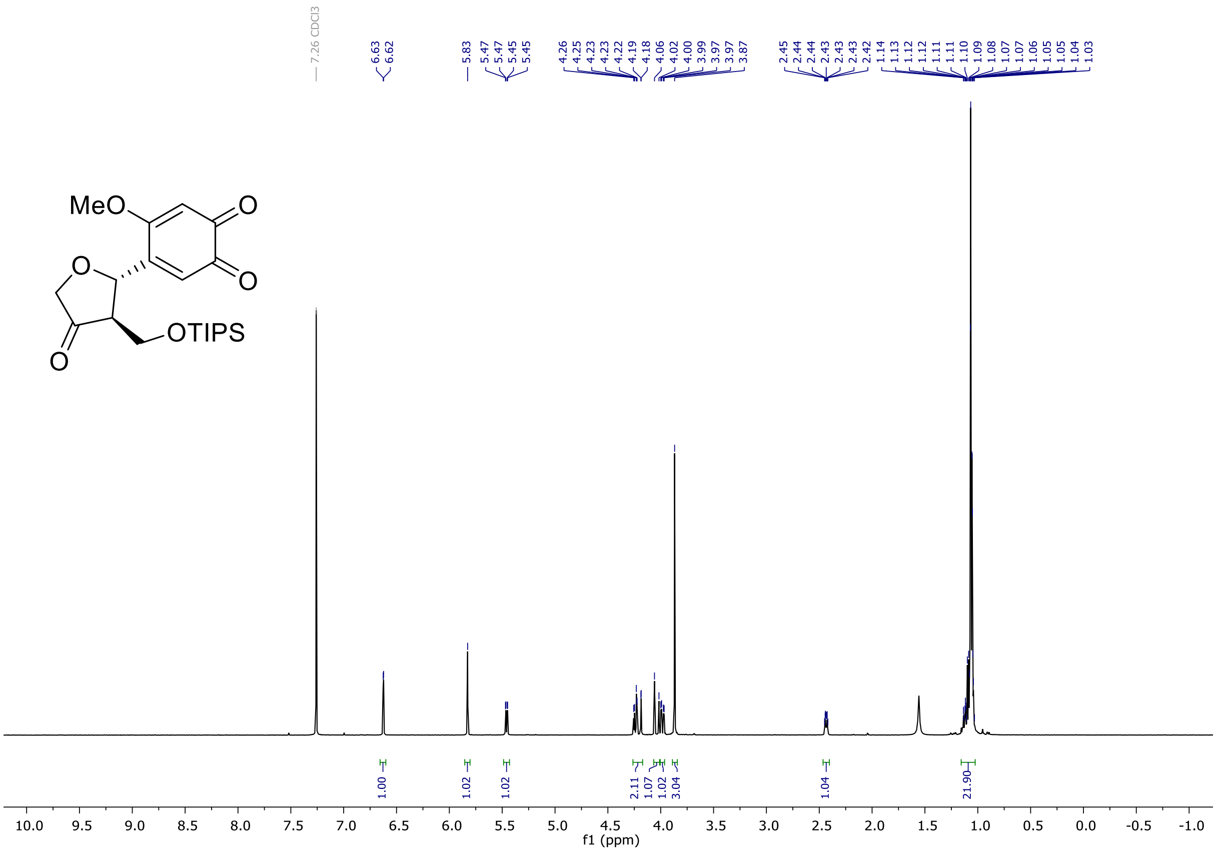

^{13}C NMR (101 MHz, $CDCl_3$) of quinone **25a:**

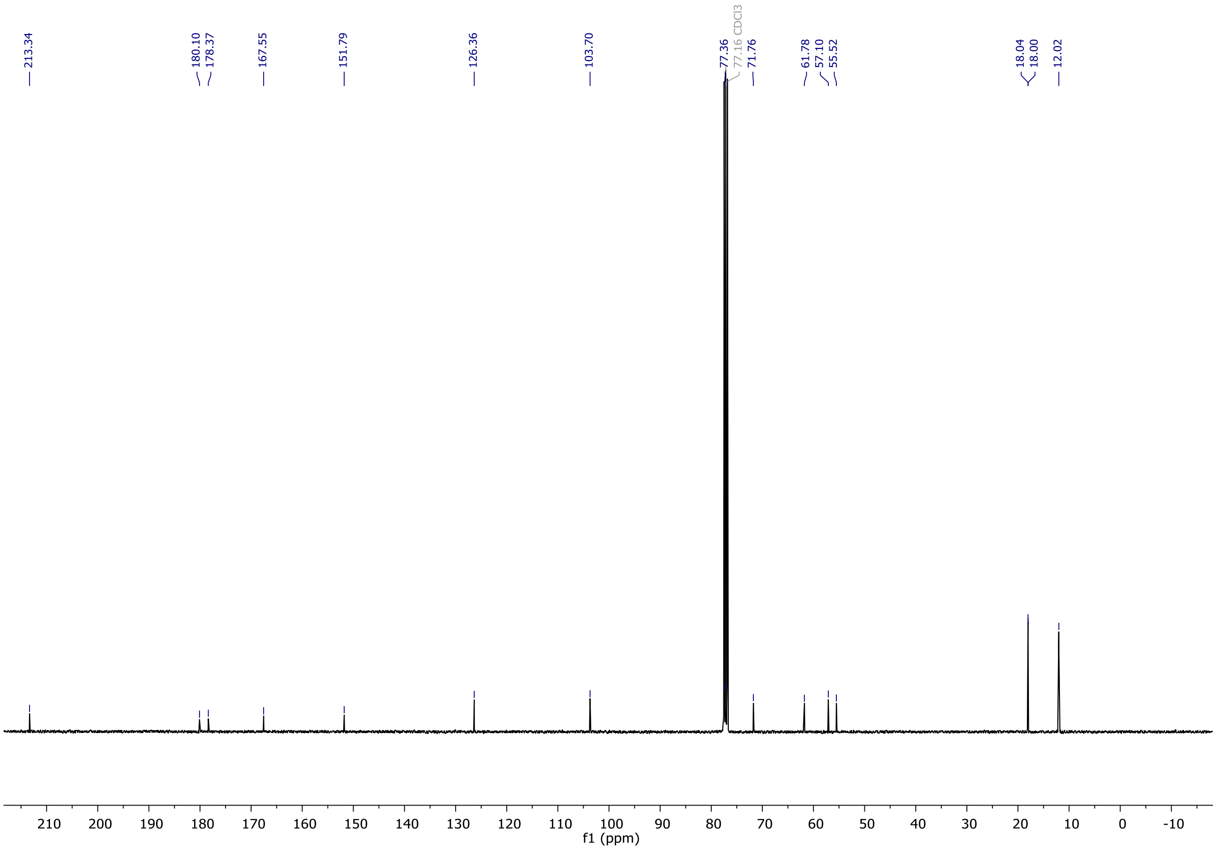

^{1}H NMR (400 MHz, CDCl$_3$) of alcohol **27**:

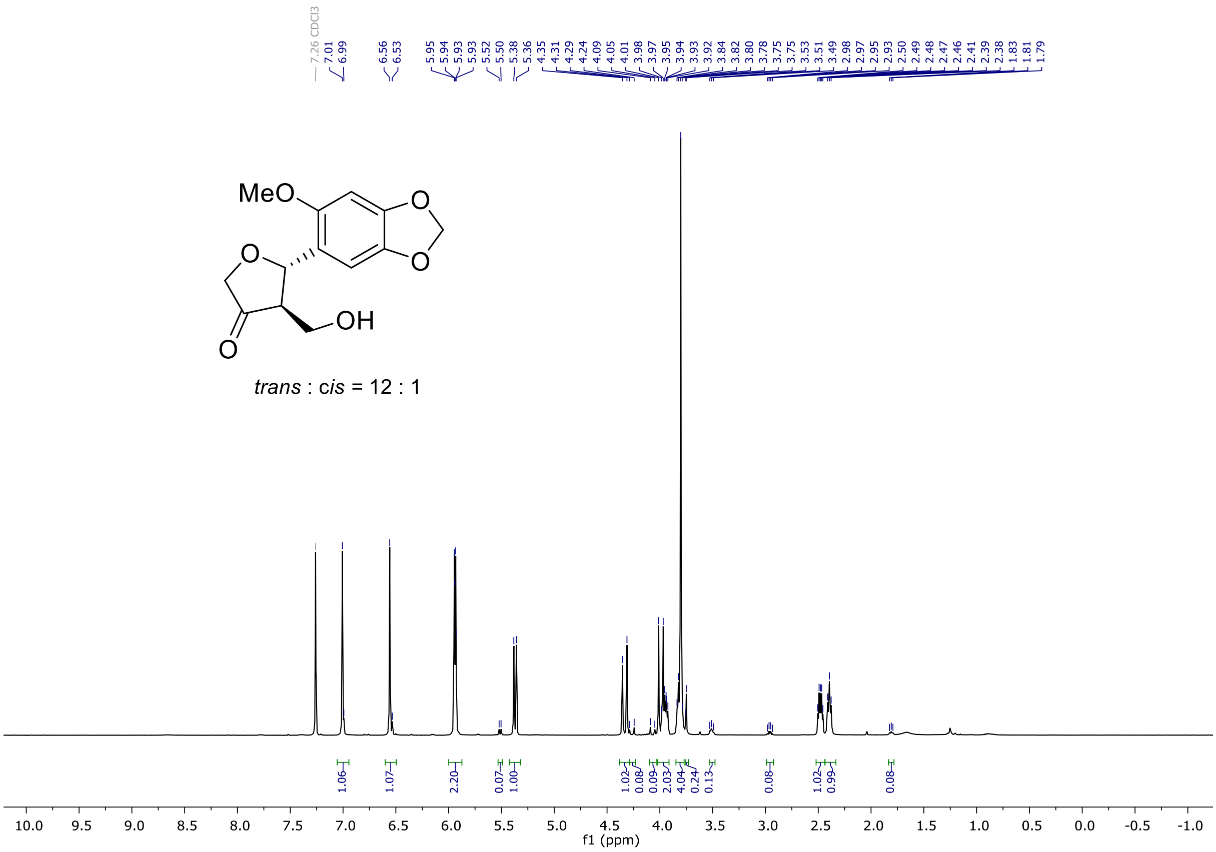

^{13}C NMR (101 MHz, CDCl$_3$) of alcohol **27:**

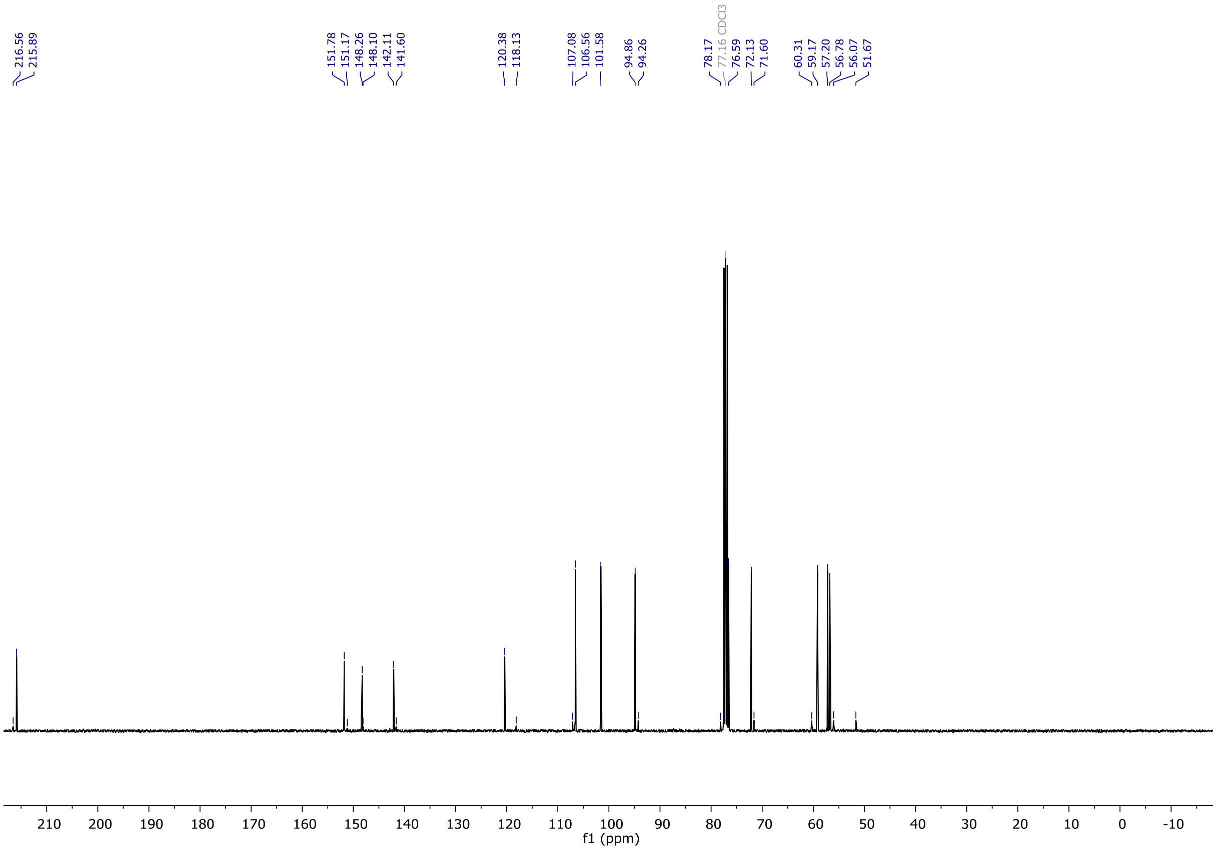

^{1}H NMR (500 MHz, DMSO-d_6) of diol **15**:

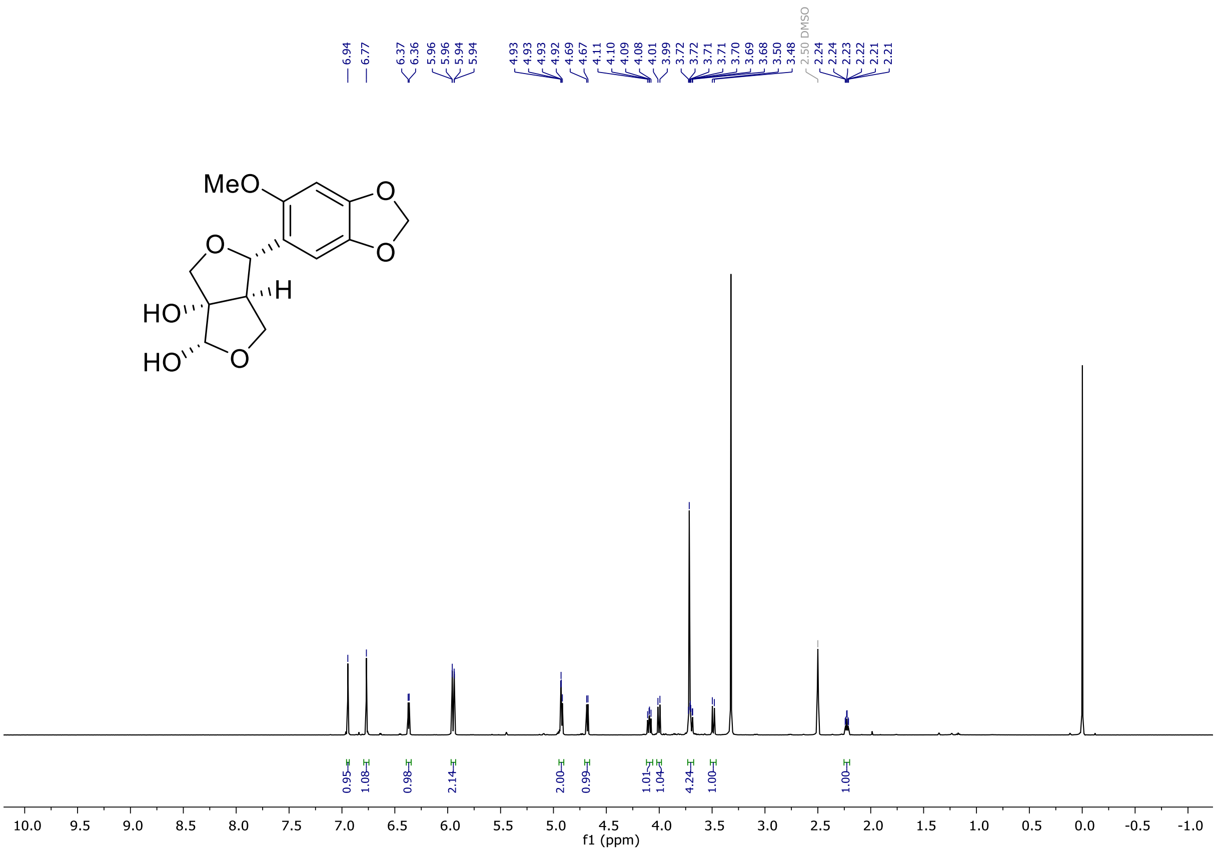

^{13}C NMR (126 MHz, DMSO-d_6) of diol **15:**

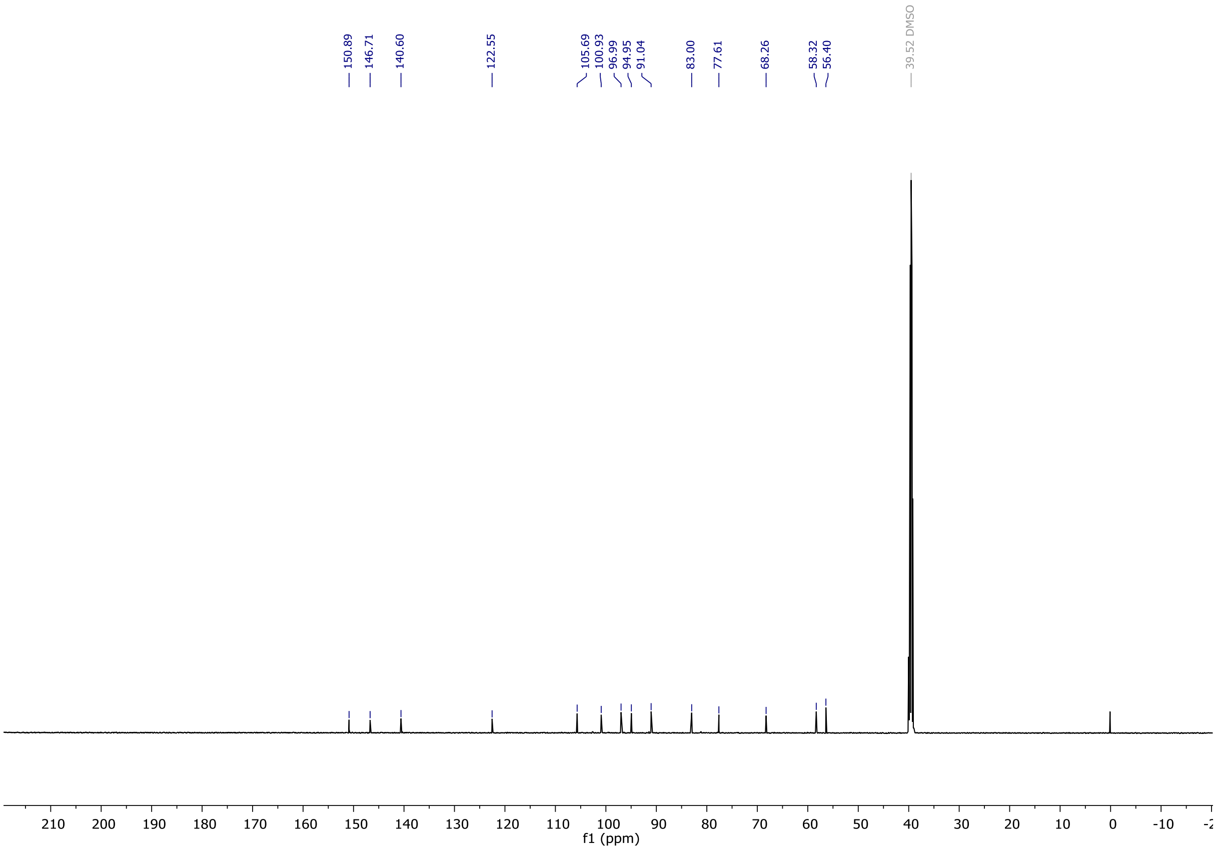

^{1}H NMR (400 MHz, $CDCl_3$) of carbonate **30**:

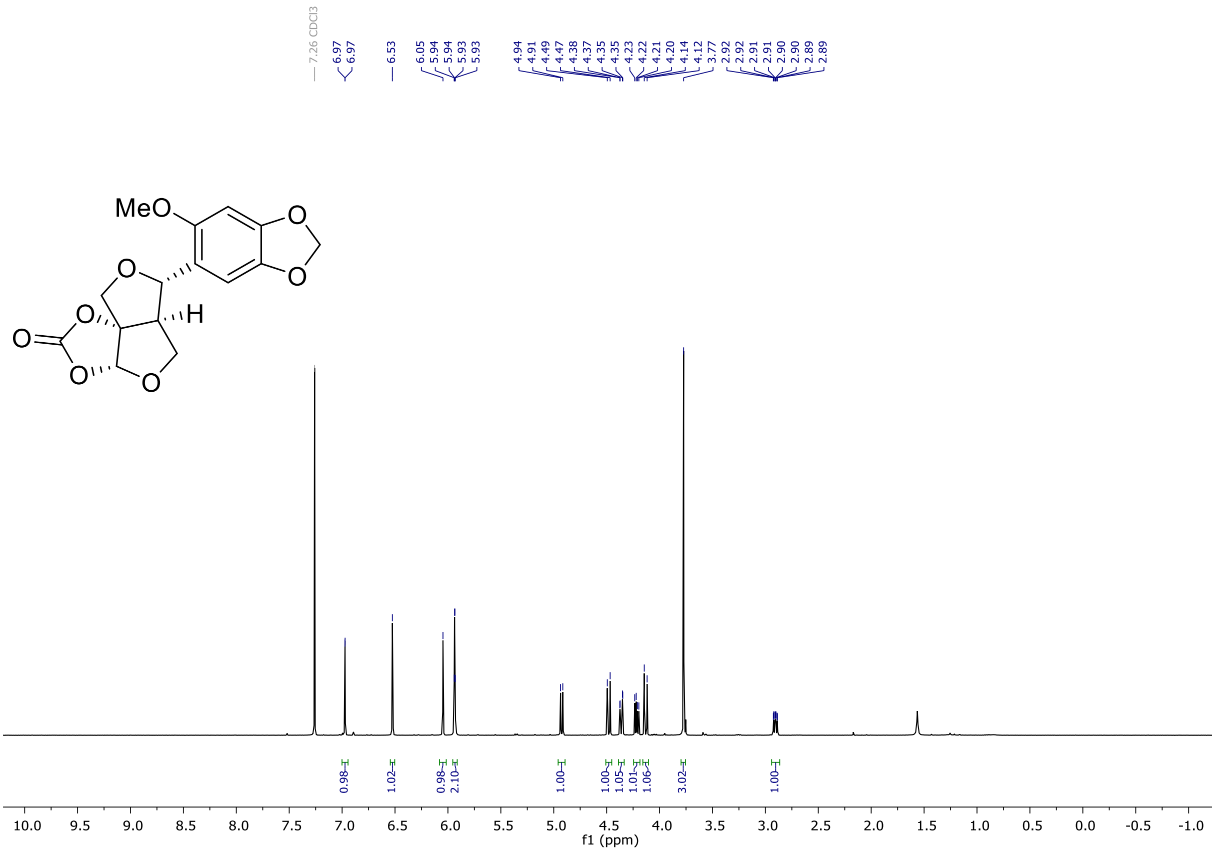

^{13}C NMR (101 MHz, $CDCl_3$) of carbonate **30:**

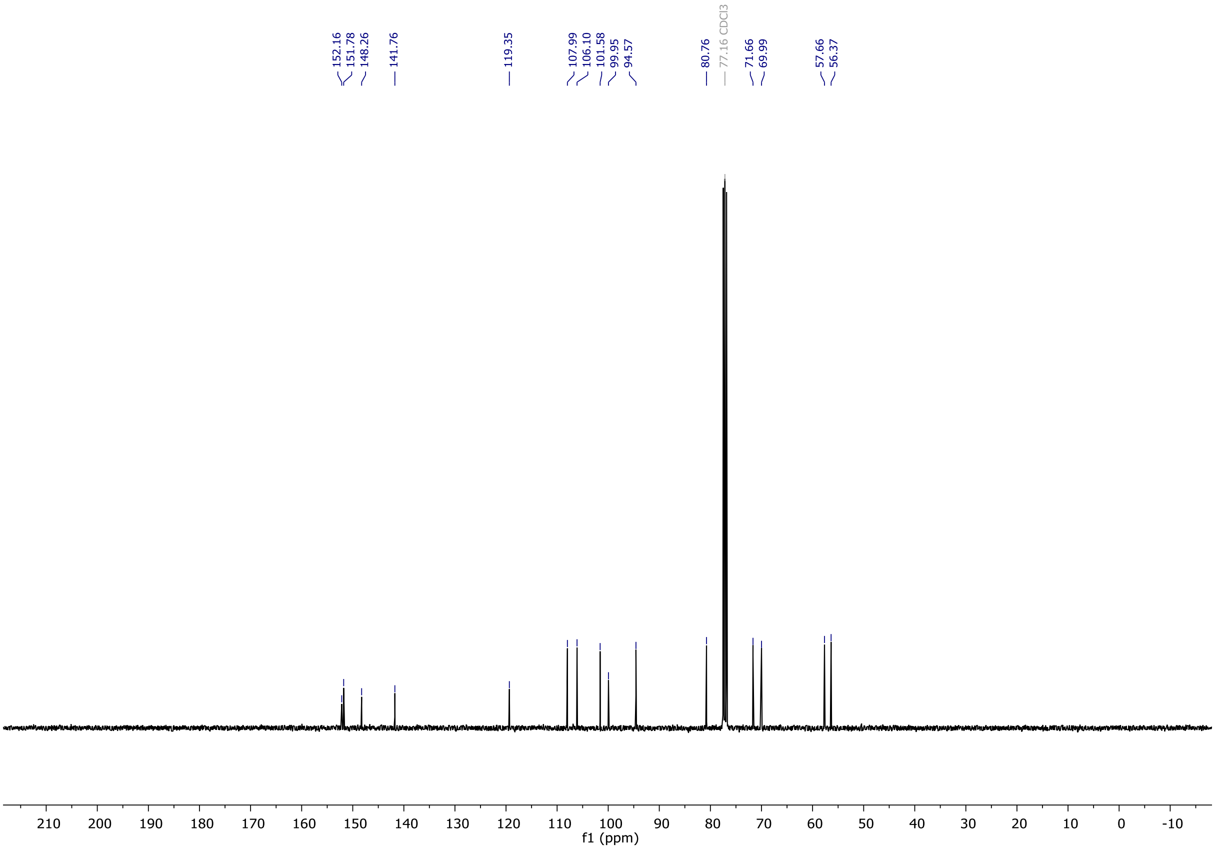

^{1}H NMR (400 MHz, $CDCl_3$) of acetal 6-*epi*-**29a**:

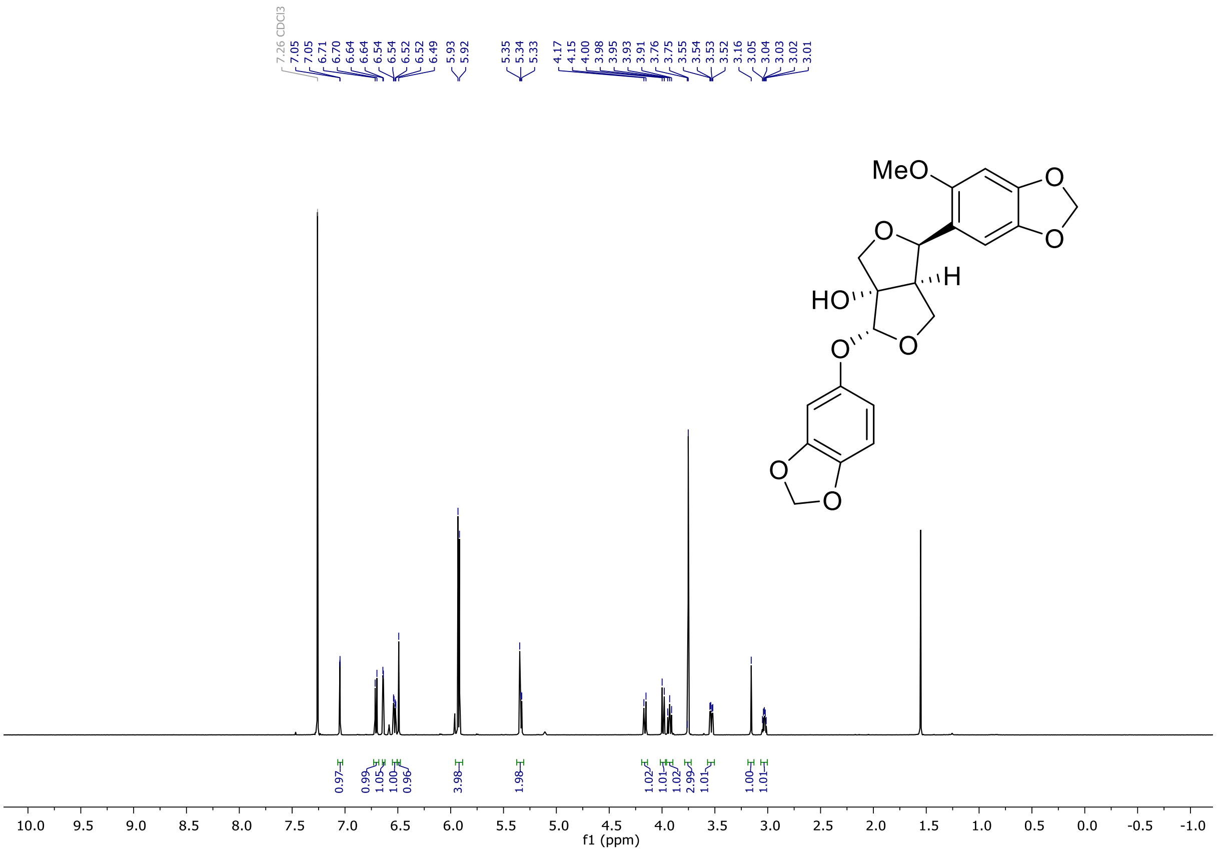

^{1}H NMR (400 MHz, $CDCl_3$) of acetal **29a**:

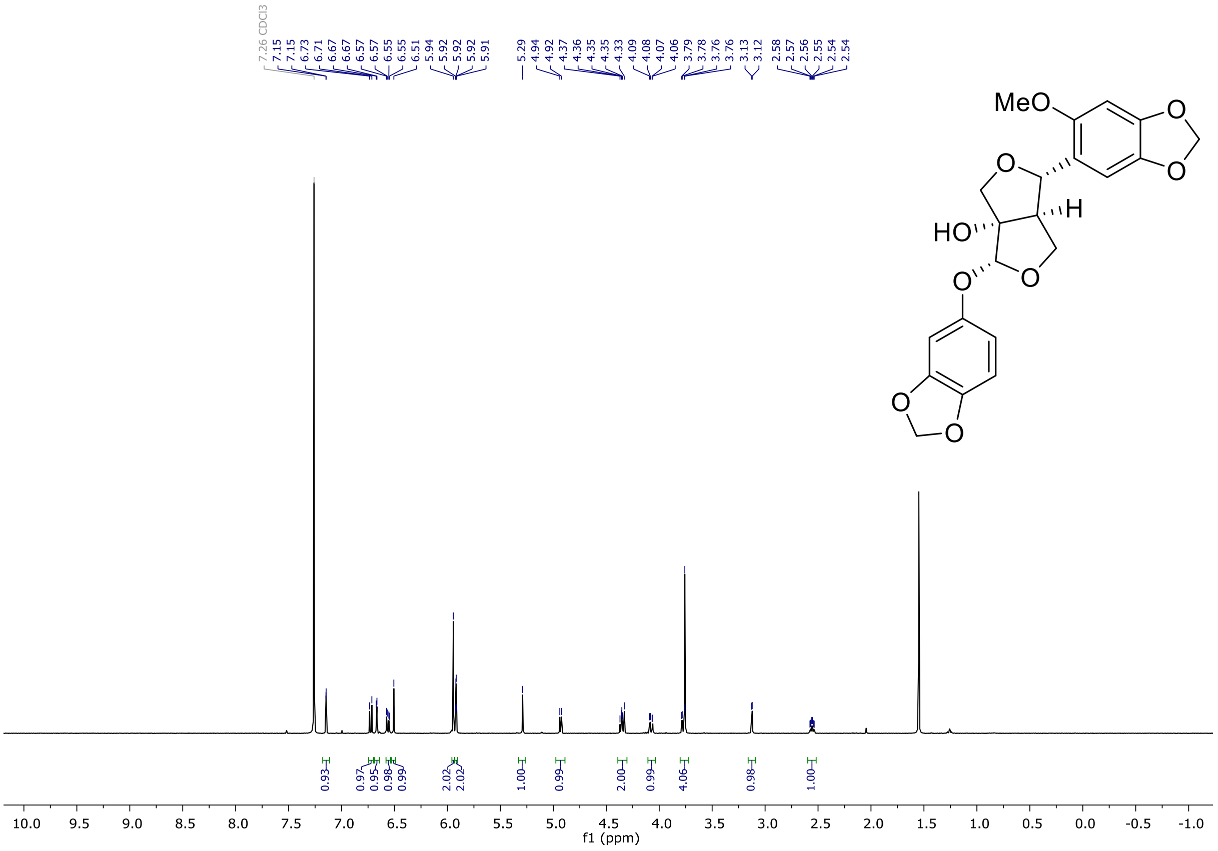

^{1}H NMR (400 MHz, $CDCl_3$) of phrymarolin II (**2a**):

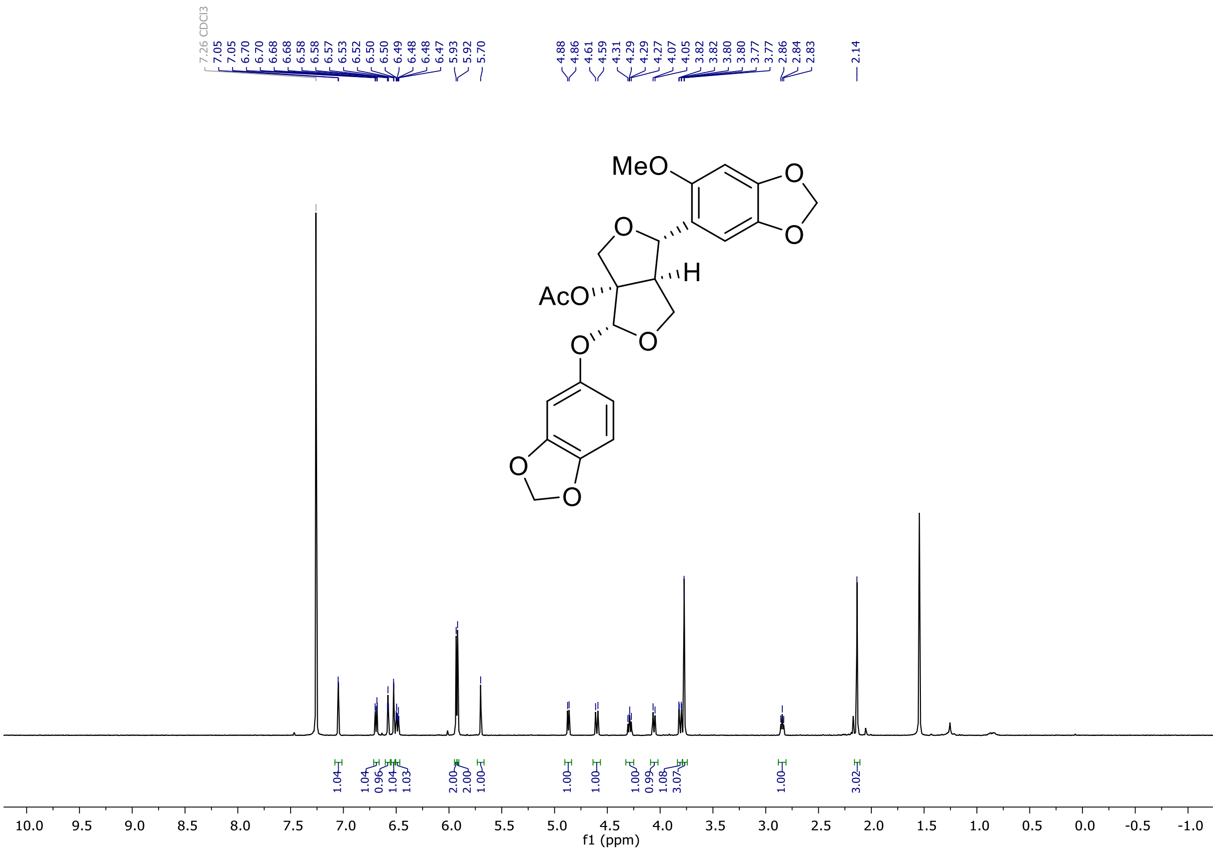

^{13}C NMR (101 MHz, $CDCl_3$) of phrymarolin II (**2a**):

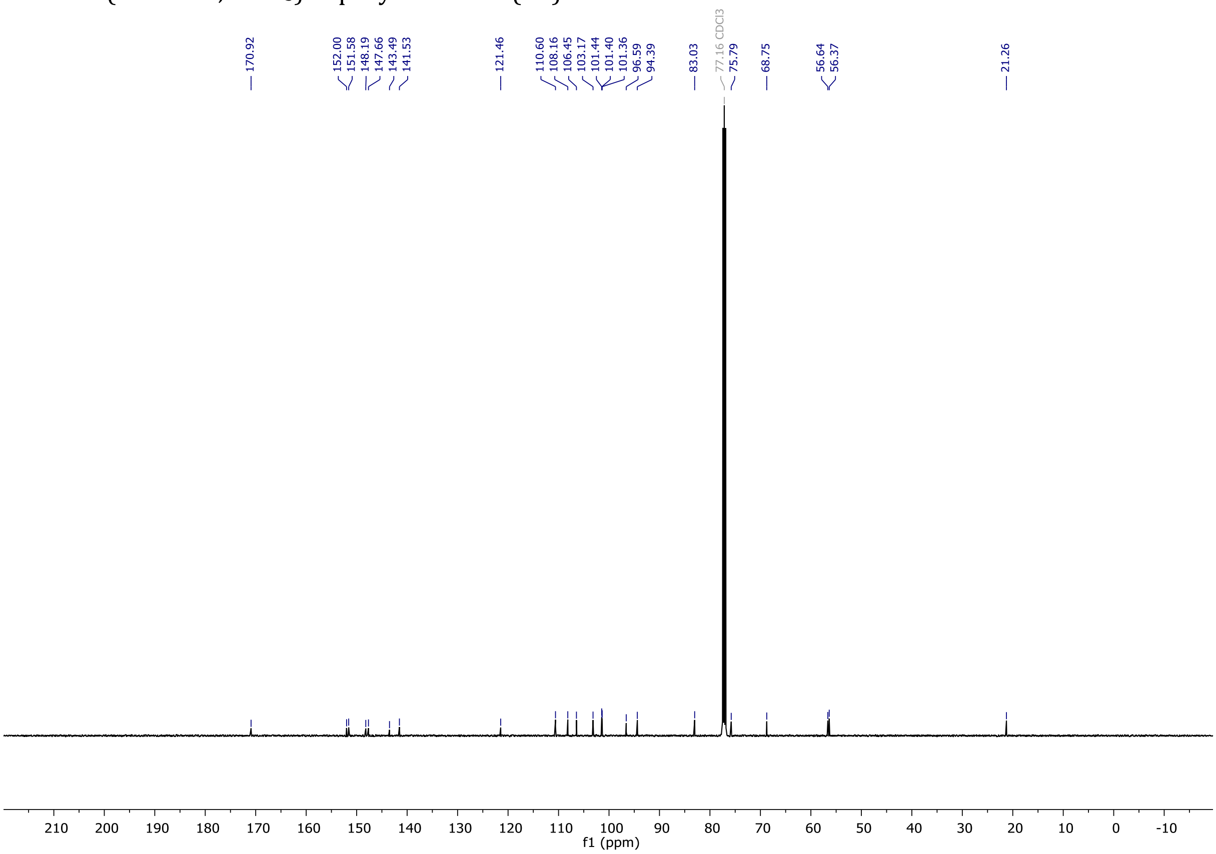

^{1}H NMR (400 MHz, $CDCl_3$) of acetal 6-*epi*-**29b**:

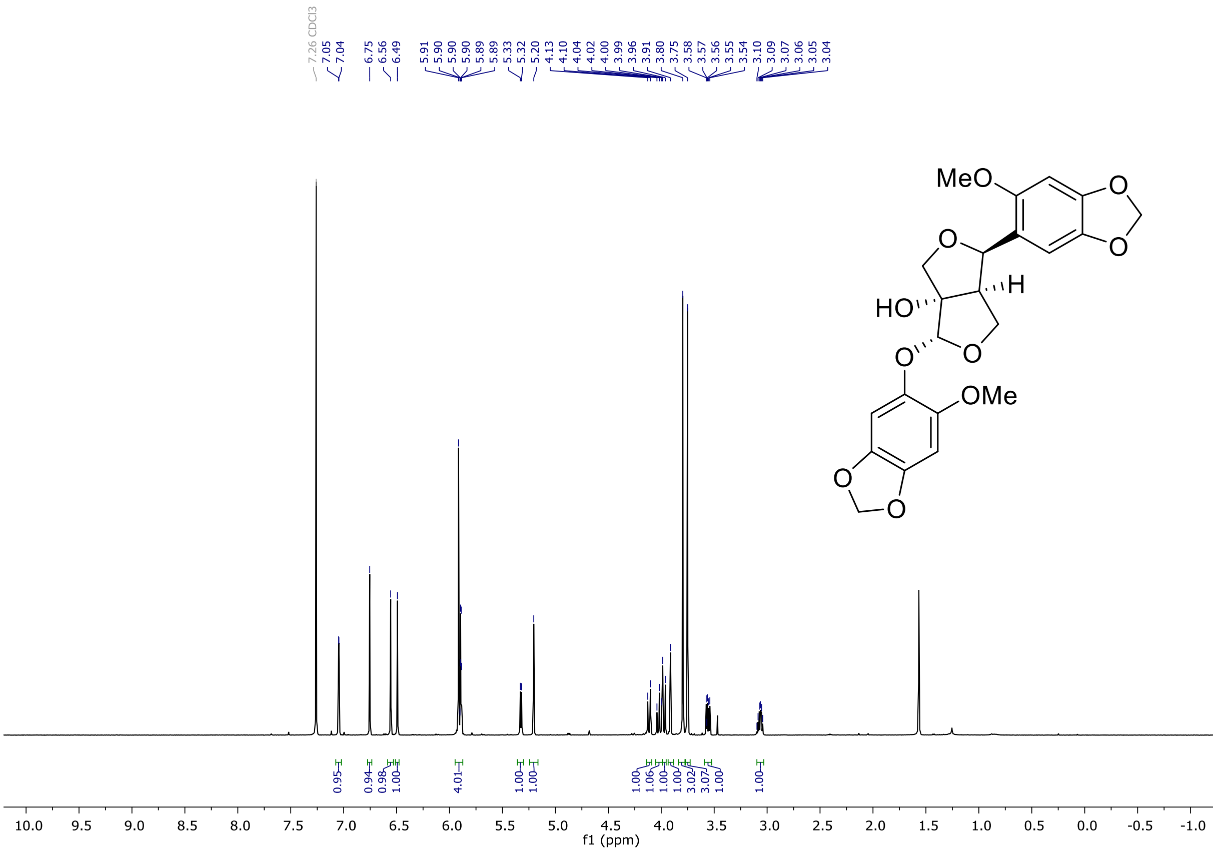

^{13}C NMR (101 MHz, $CDCl_3$) of acetal 6-*epi*-**29b:**

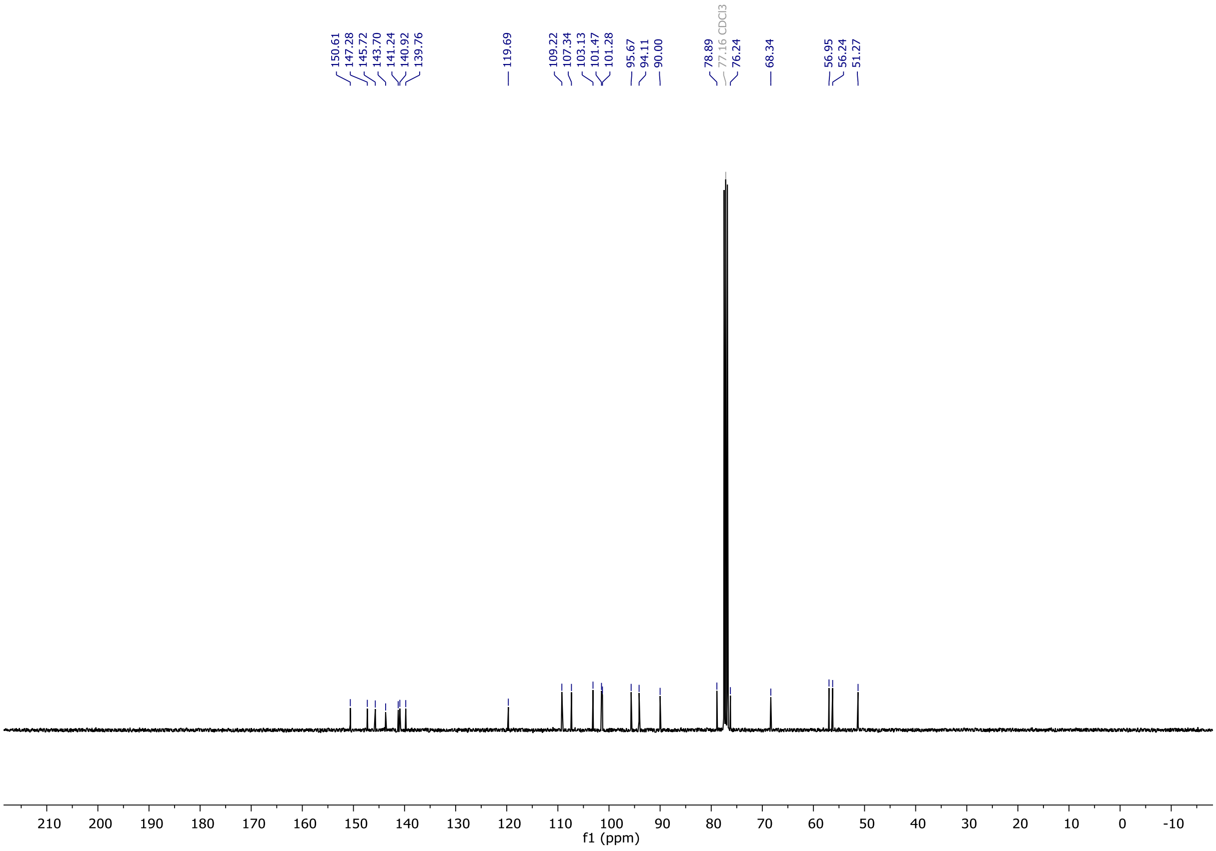

^{1}H NMR (400 MHz, $CDCl_3$) of acetal **29b**:

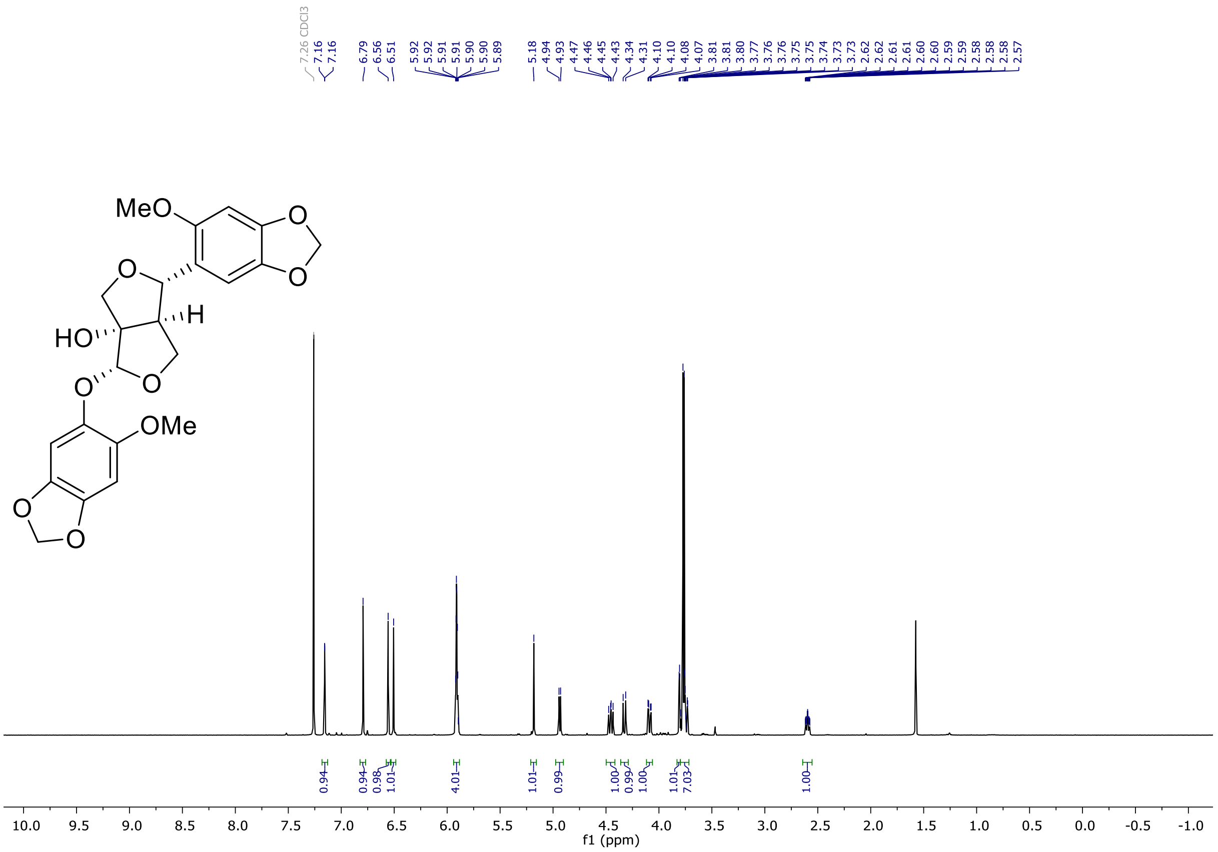

^{1}H NMR (400 MHz, $CDCl_3$) of phrymarolin I (**2b**):

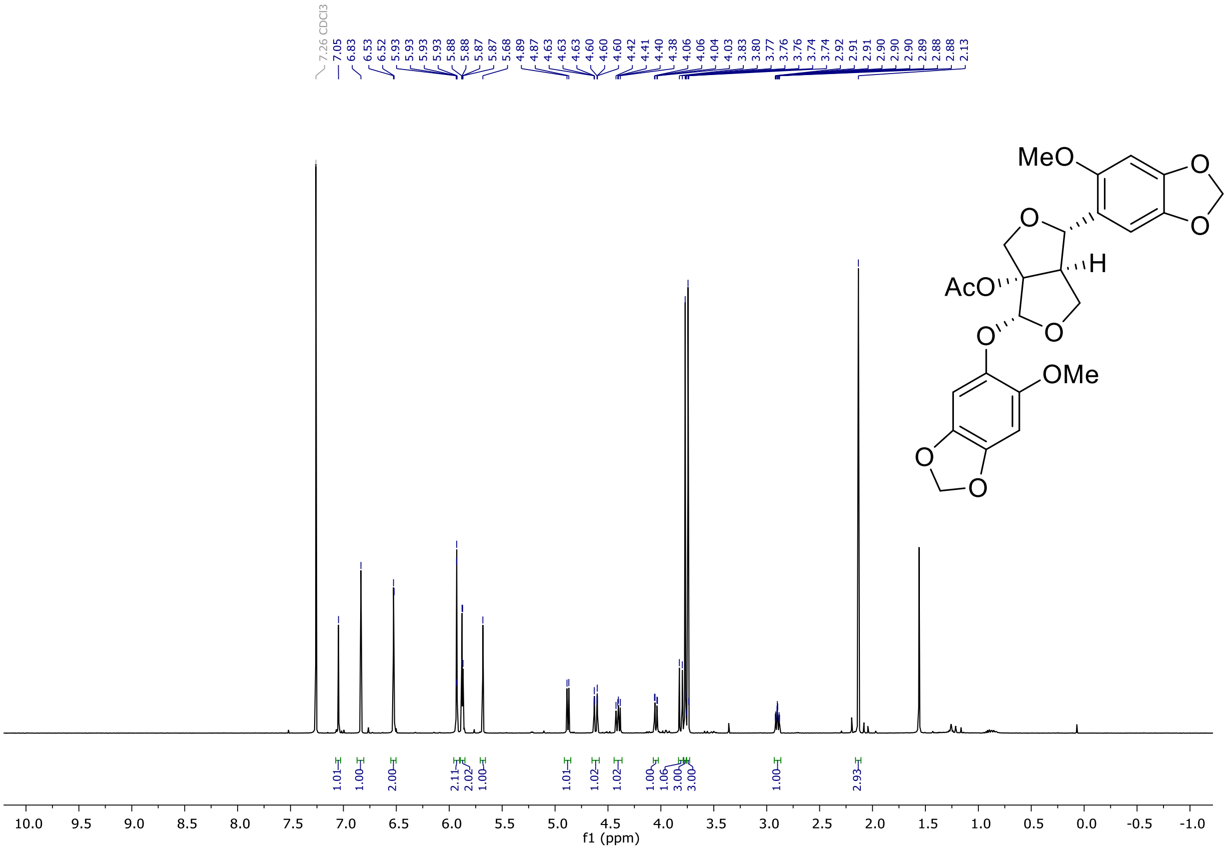

^{13}C NMR (101 MHz, $CDCl_3$) of phrymarolin I (**2b**):

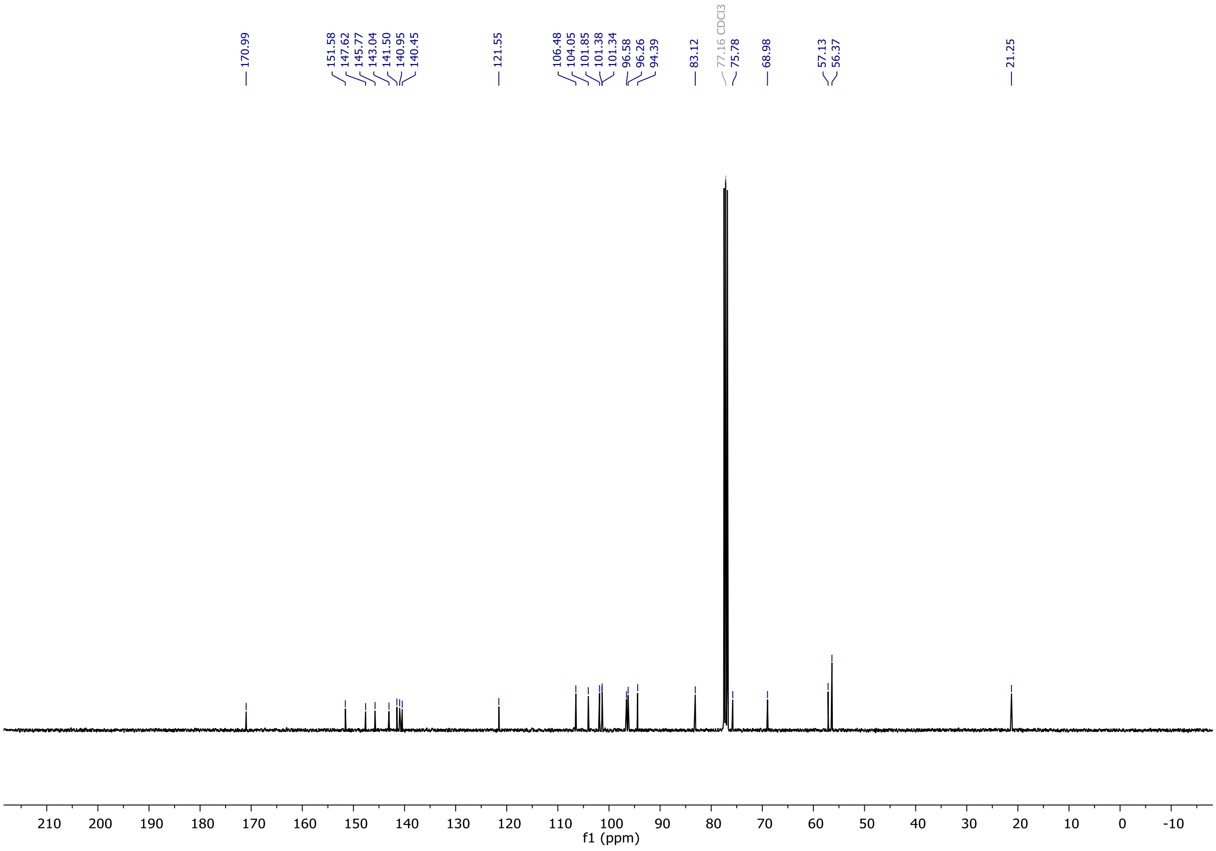

^{1}H NMR (400 MHz, $CDCl_3$) of aryl bromide **34**:

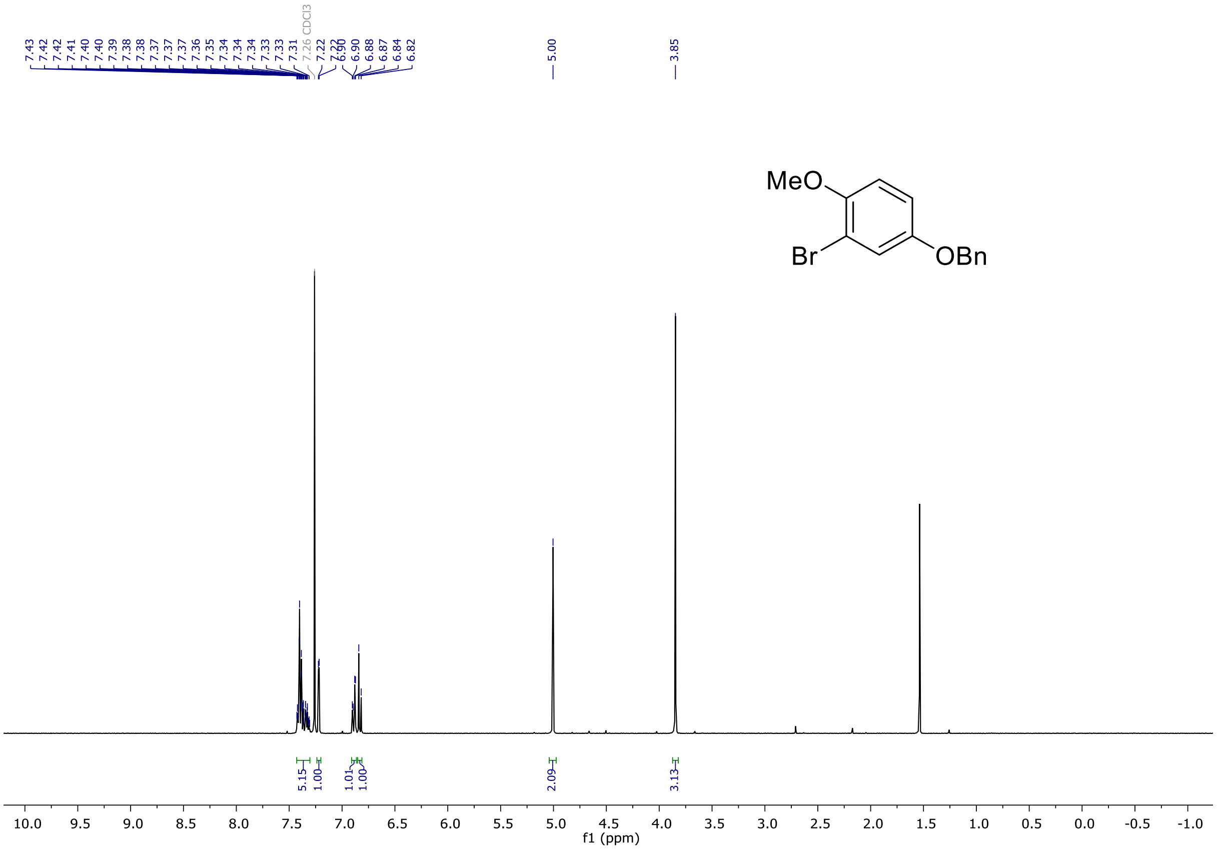

^{1}H NMR (400 MHz, $CDCl_3$) of aryl bromide **33**:

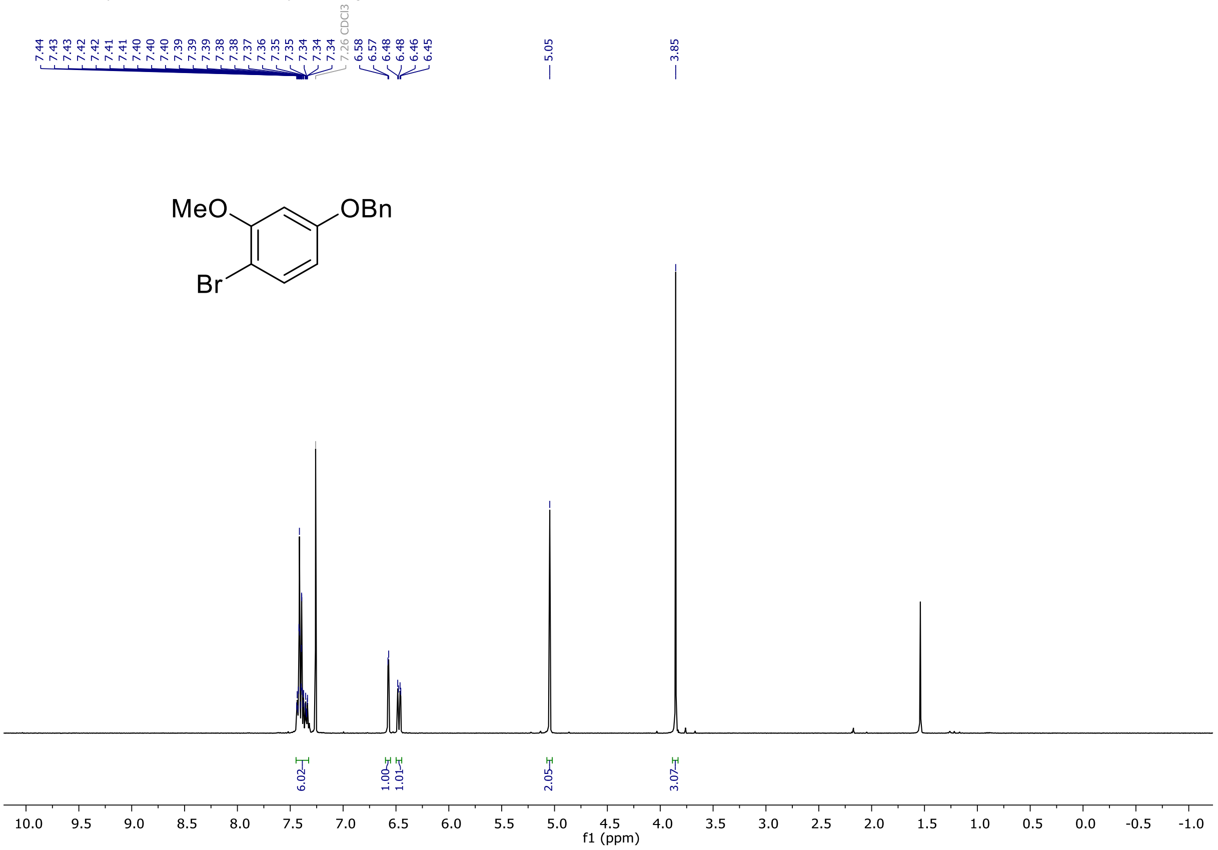

^{1}H NMR (400 MHz, CDCl$_3$) of styrene **32**:

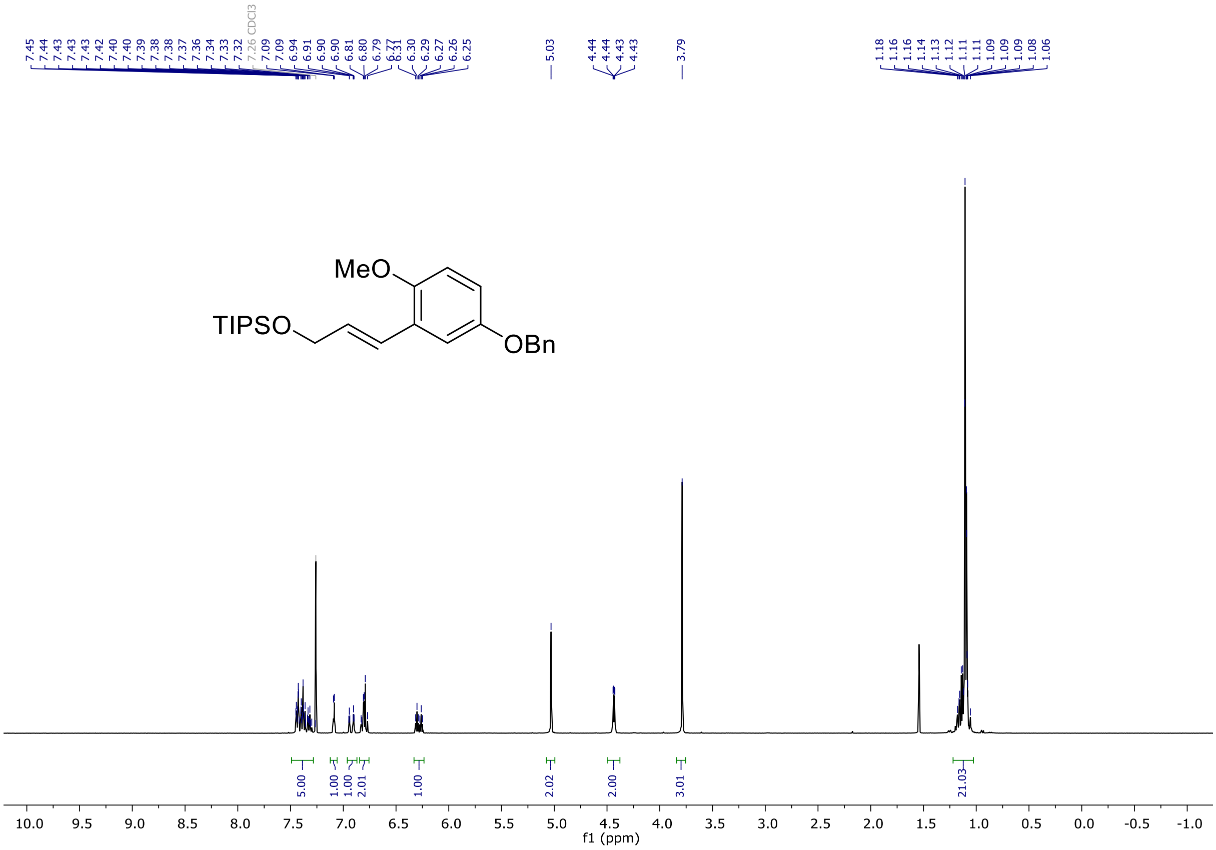

^{13}C NMR (101 MHz, CDCl$_3$) of styrene **32:**

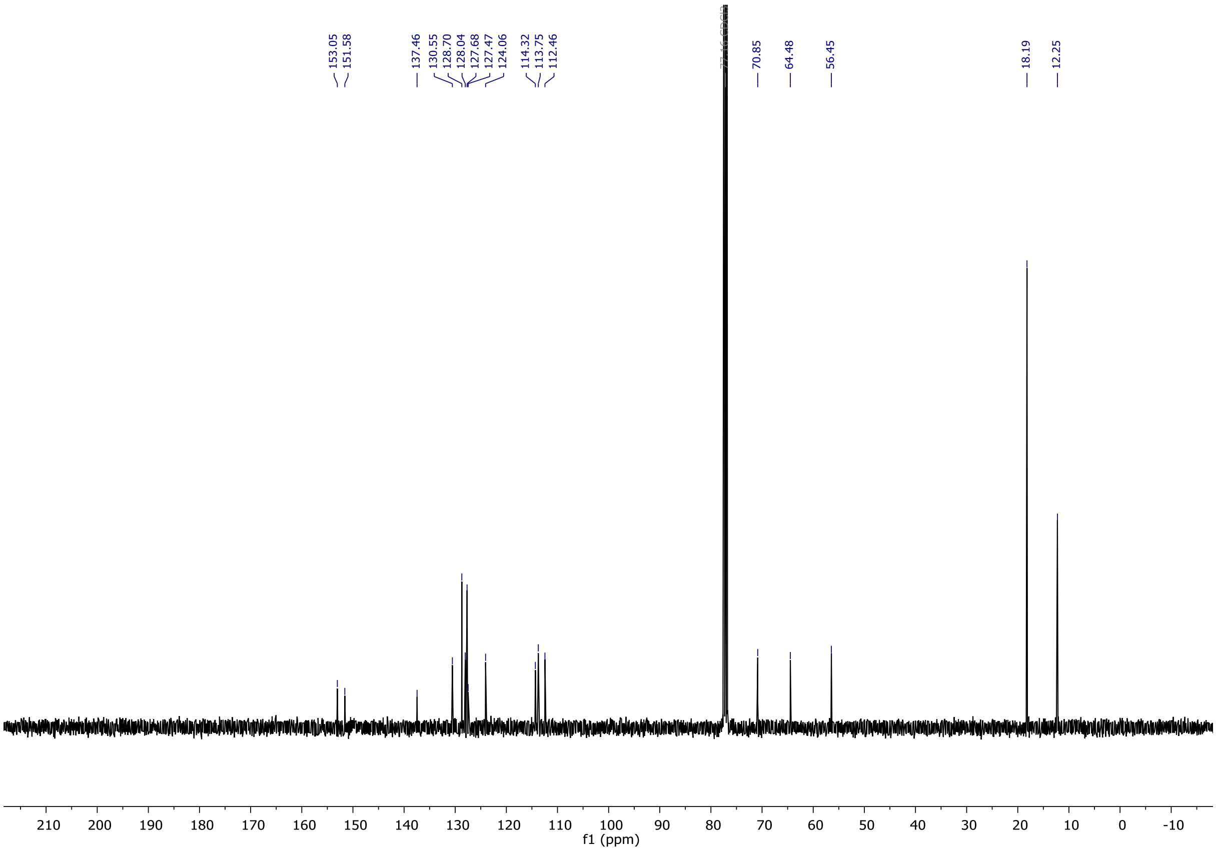

^{1}H NMR (400 MHz, $CDCl_3$) of styrene **31**:

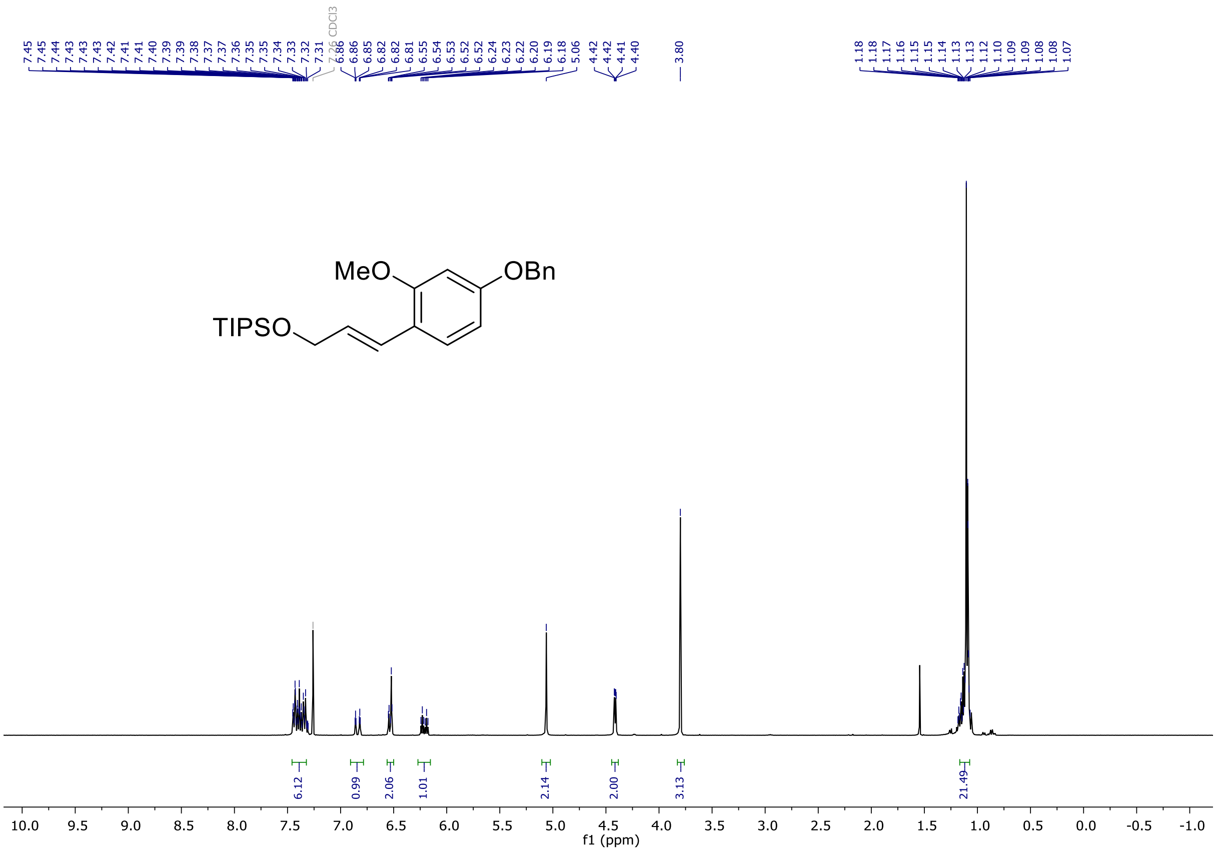

^{13}C NMR (101 MHz, $CDCl_3$) of styrene **31:**

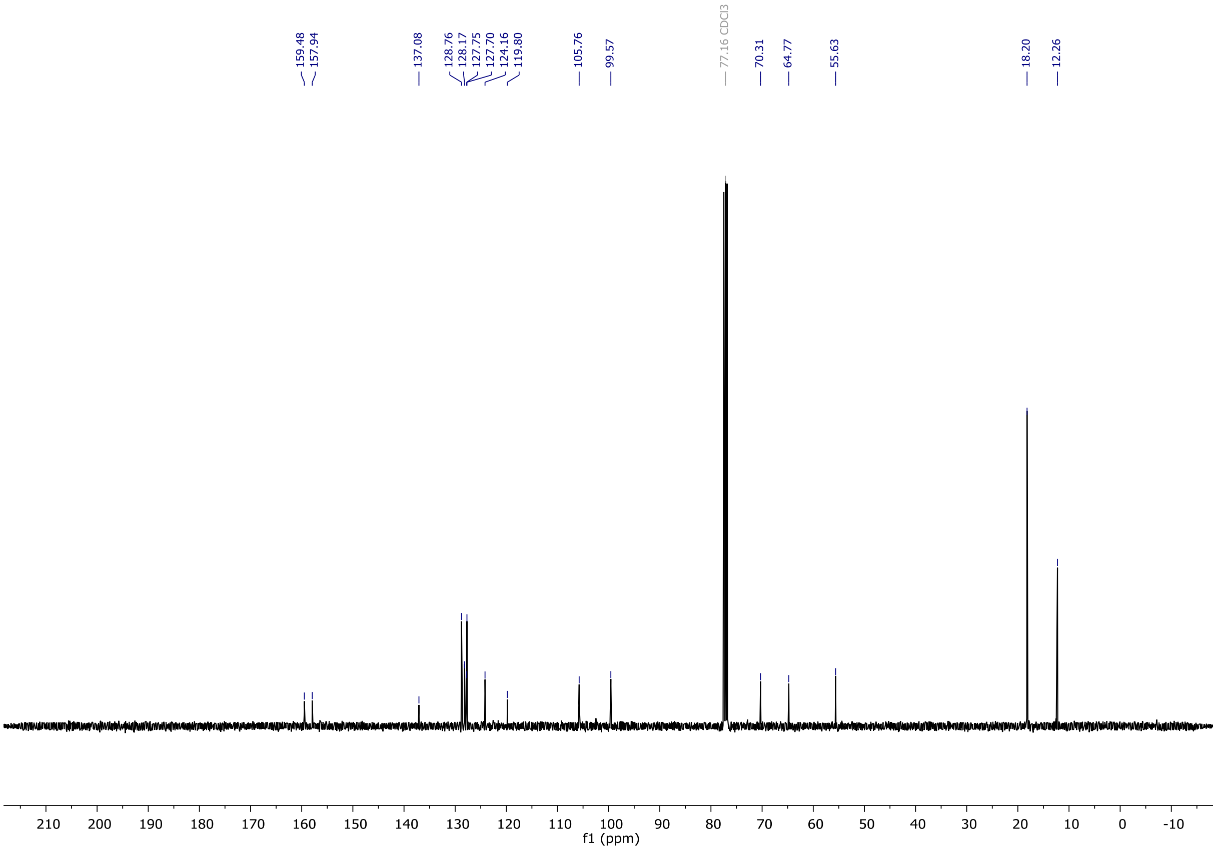

^{1}H NMR (400 MHz, $CDCl_3$) of styrene **SI-6**:

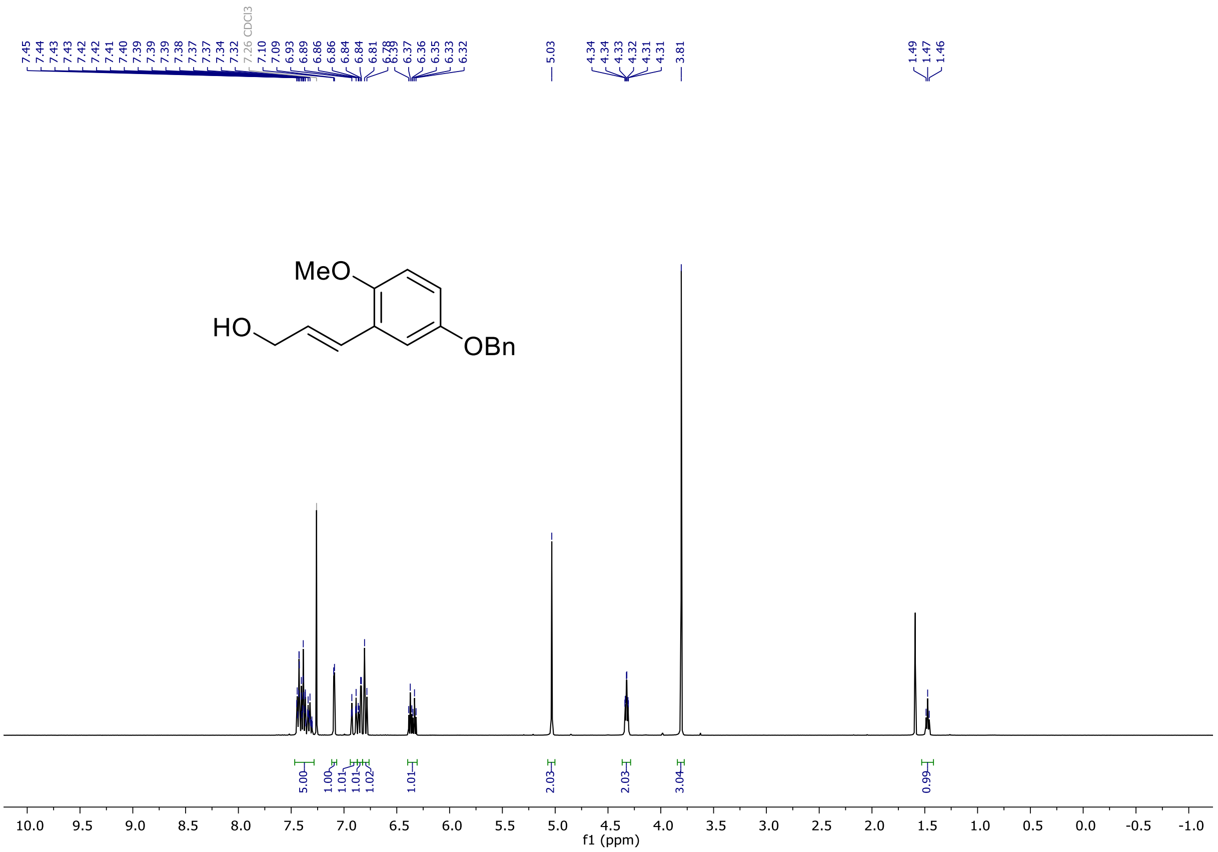

^{13}C NMR (101 MHz, $CDCl_3$) of styrene **SI-6:**

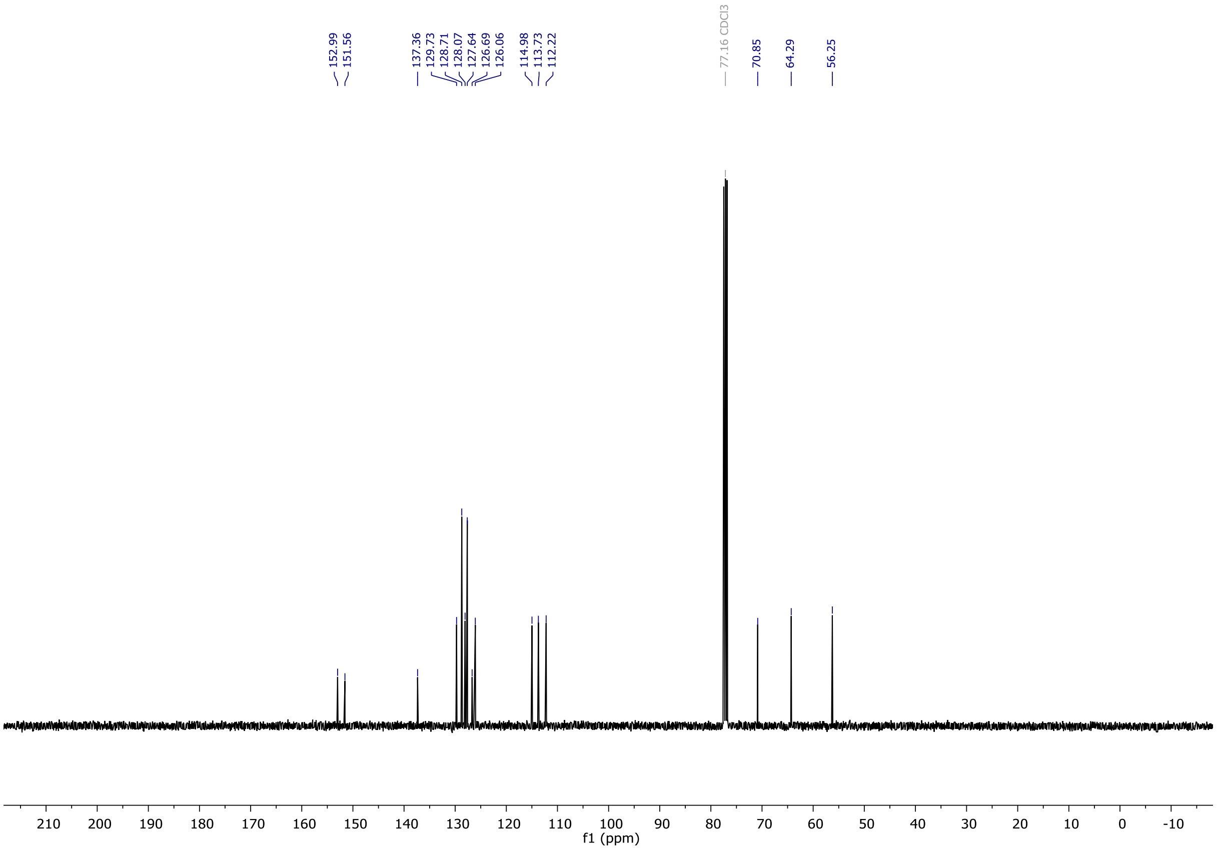

^{1}H NMR (400 MHz, $CDCl_3$) of alcohol **35a**:

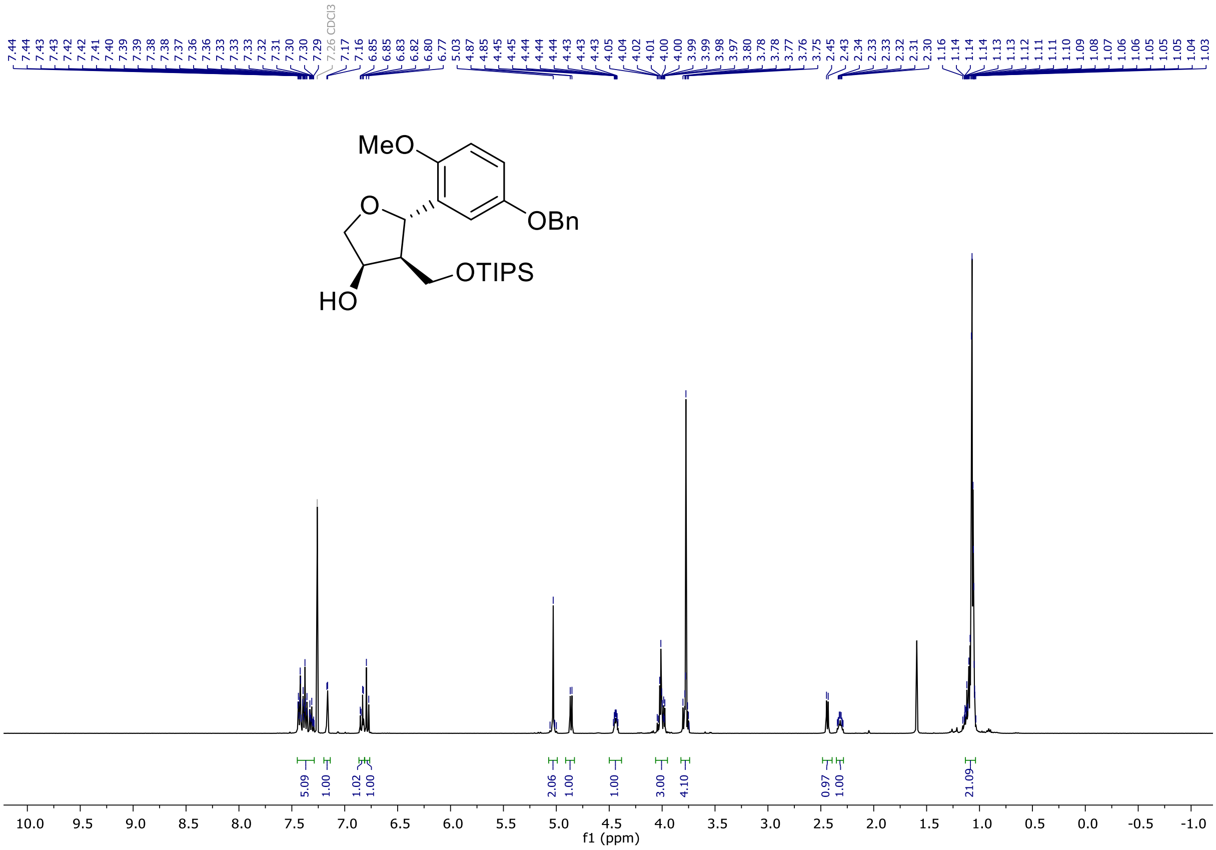

^{13}C NMR (101 MHz, $CDCl_3$) of alcohol **35a:**

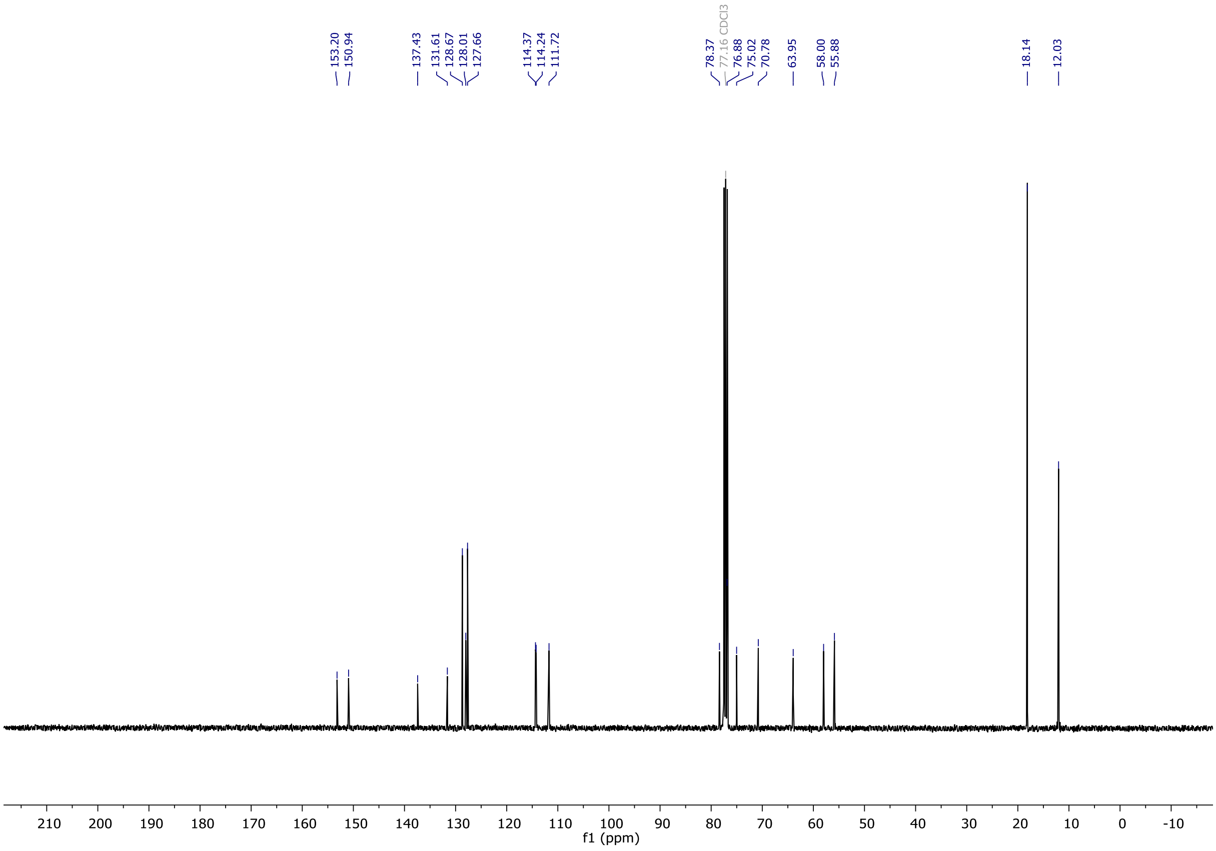

^{1}H NMR (400 MHz, $CDCl_3$) of alcohol **35b**:

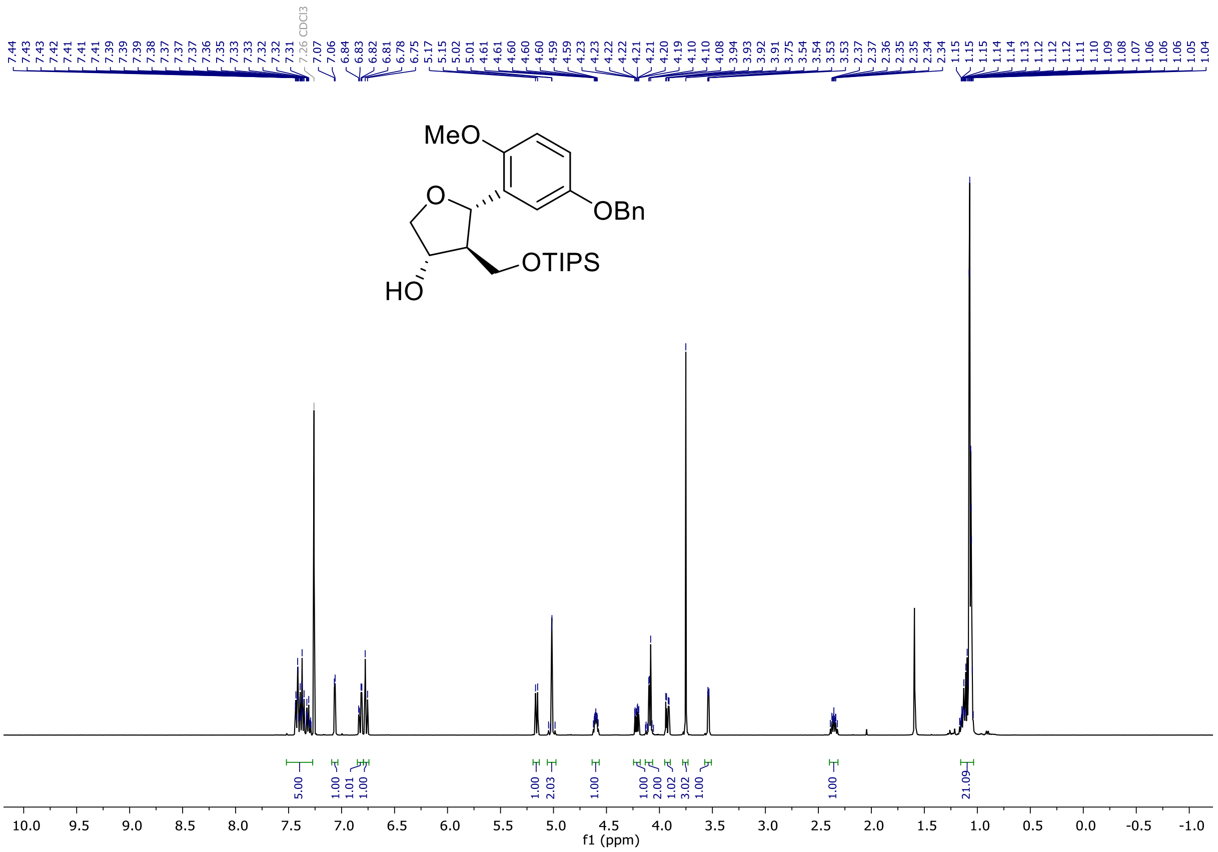

^{13}C NMR (101 MHz, $CDCl_3$) of alcohol **35b:**

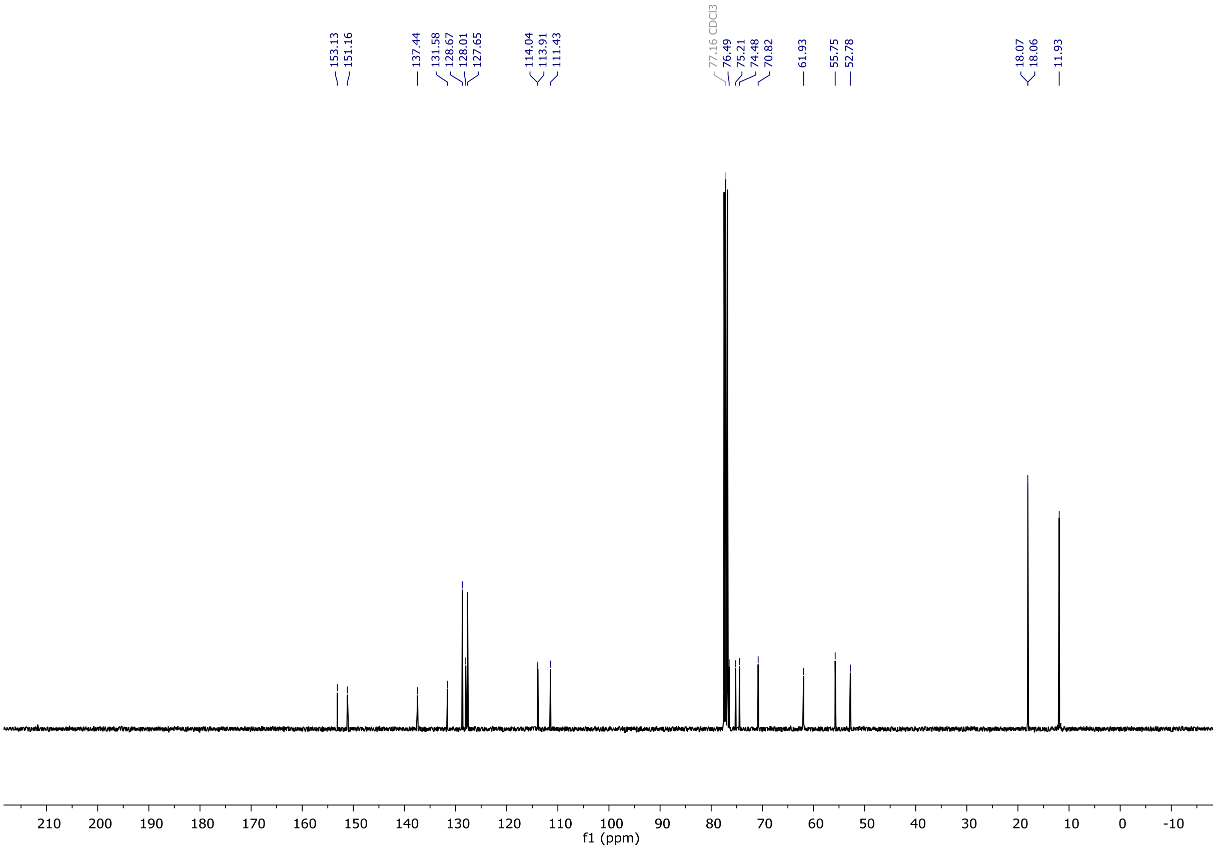

^{1}H NMR (400 MHz, $CDCl_3$) of ketone **36**:

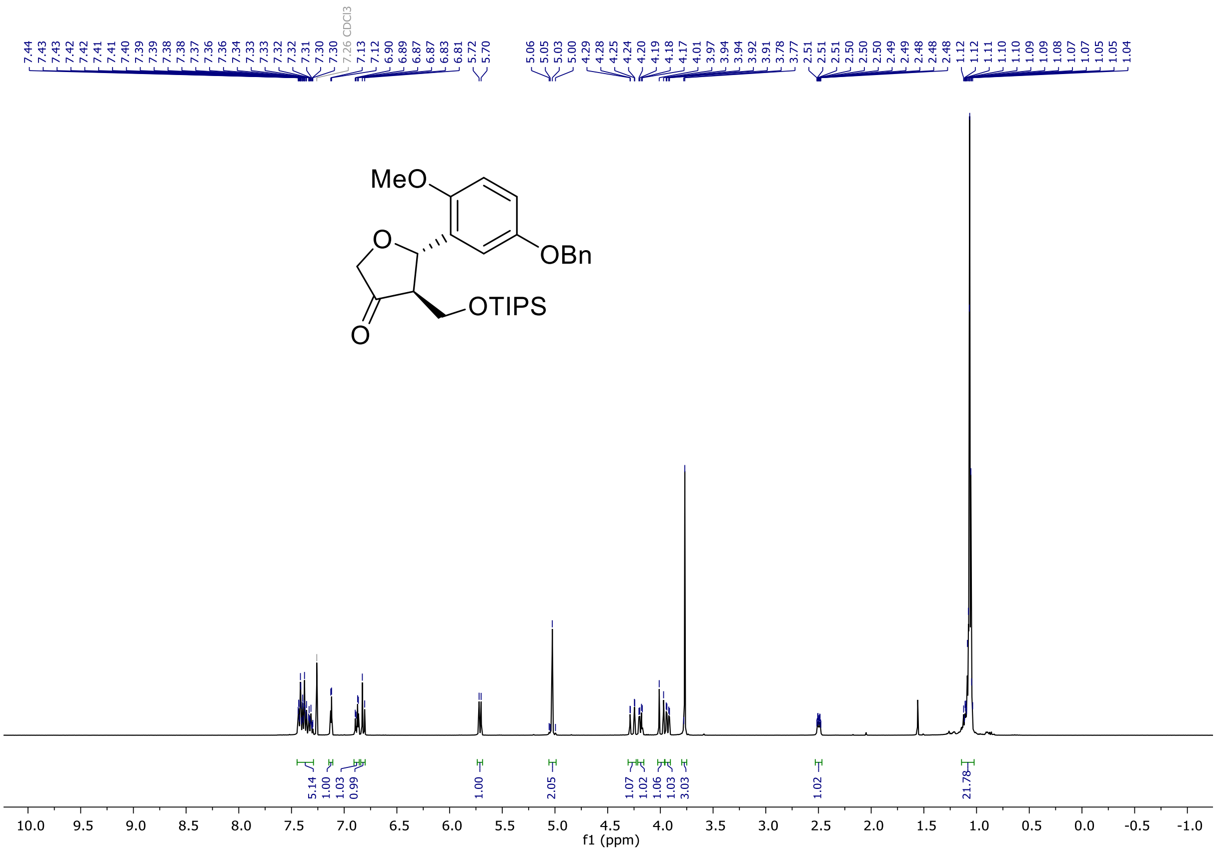

^{13}C NMR (101 MHz, $CDCl_3$) of ketone **36:**

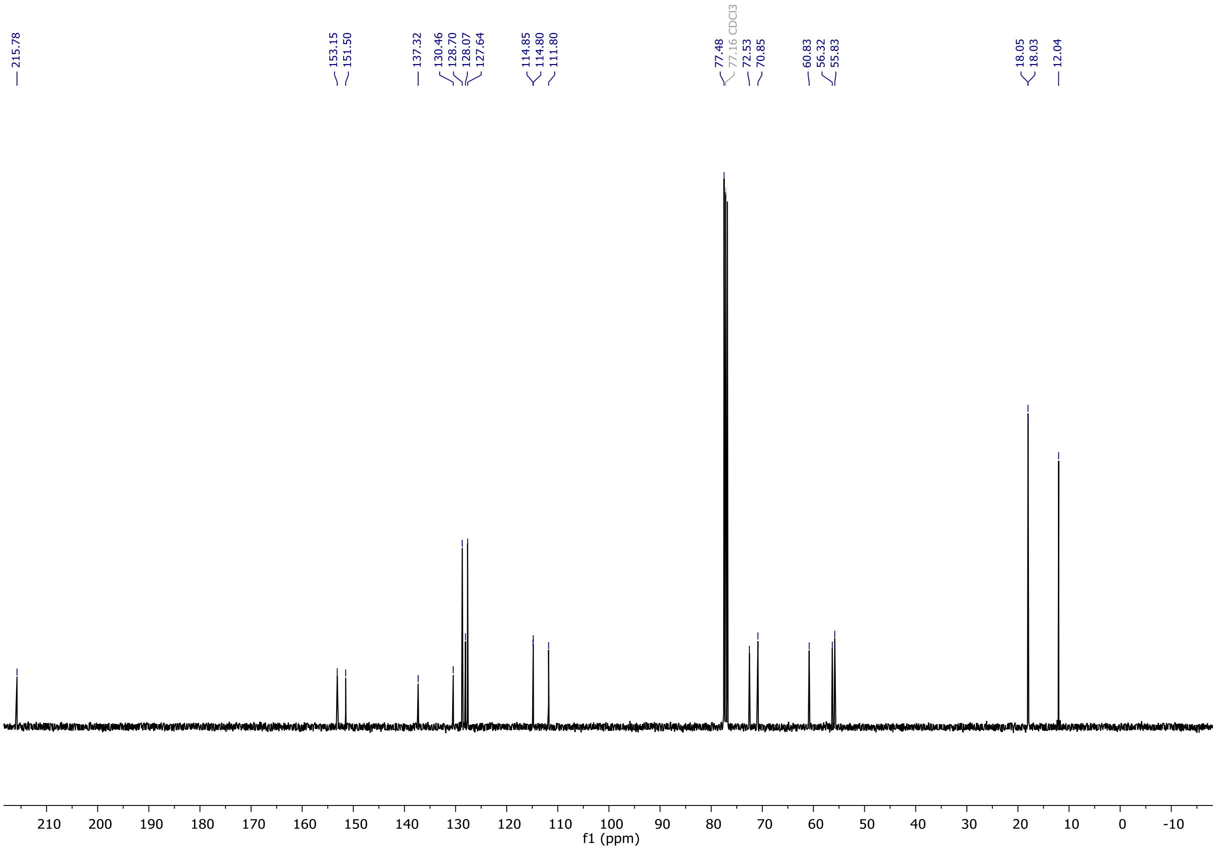

^{1}H NMR (400 MHz, $CDCl_3$) of alcohol **37**:

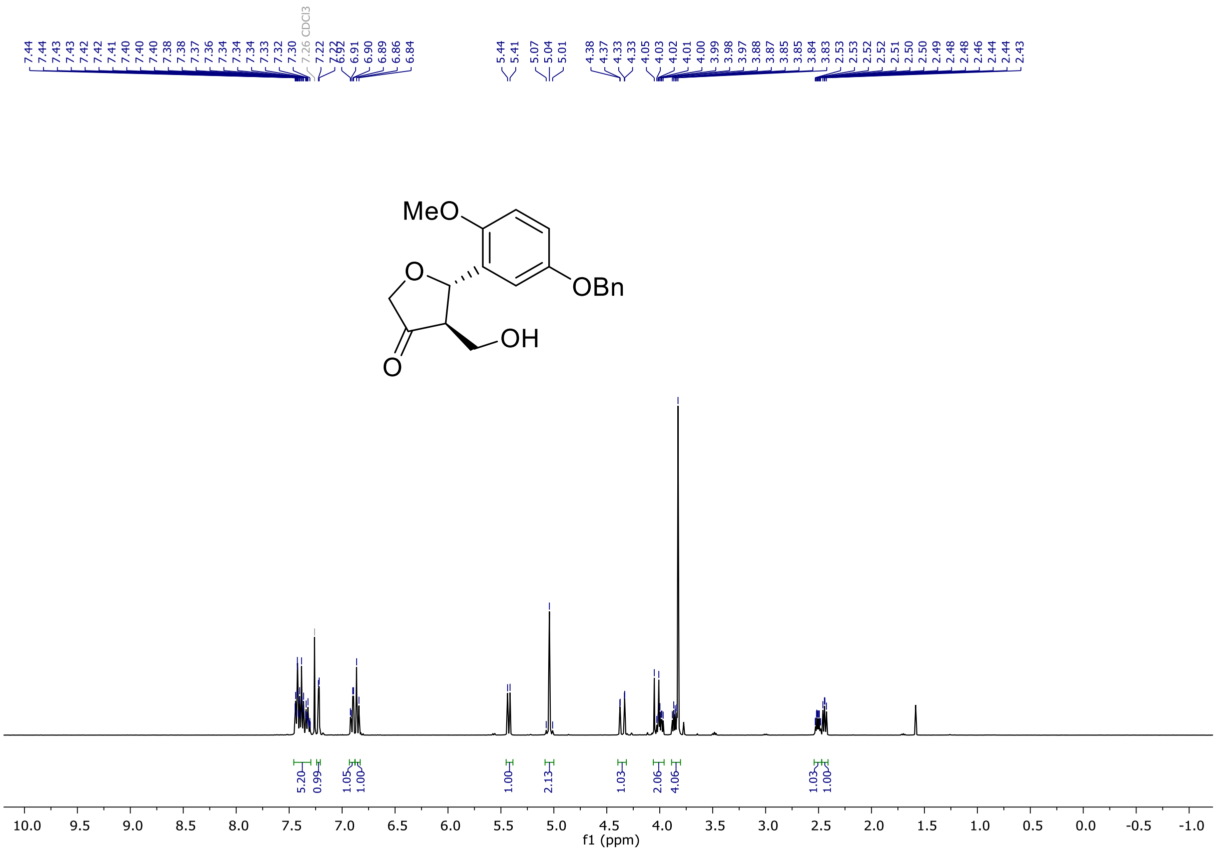

^{13}C NMR (101 MHz, $CDCl_3$) of alcohol **37:**

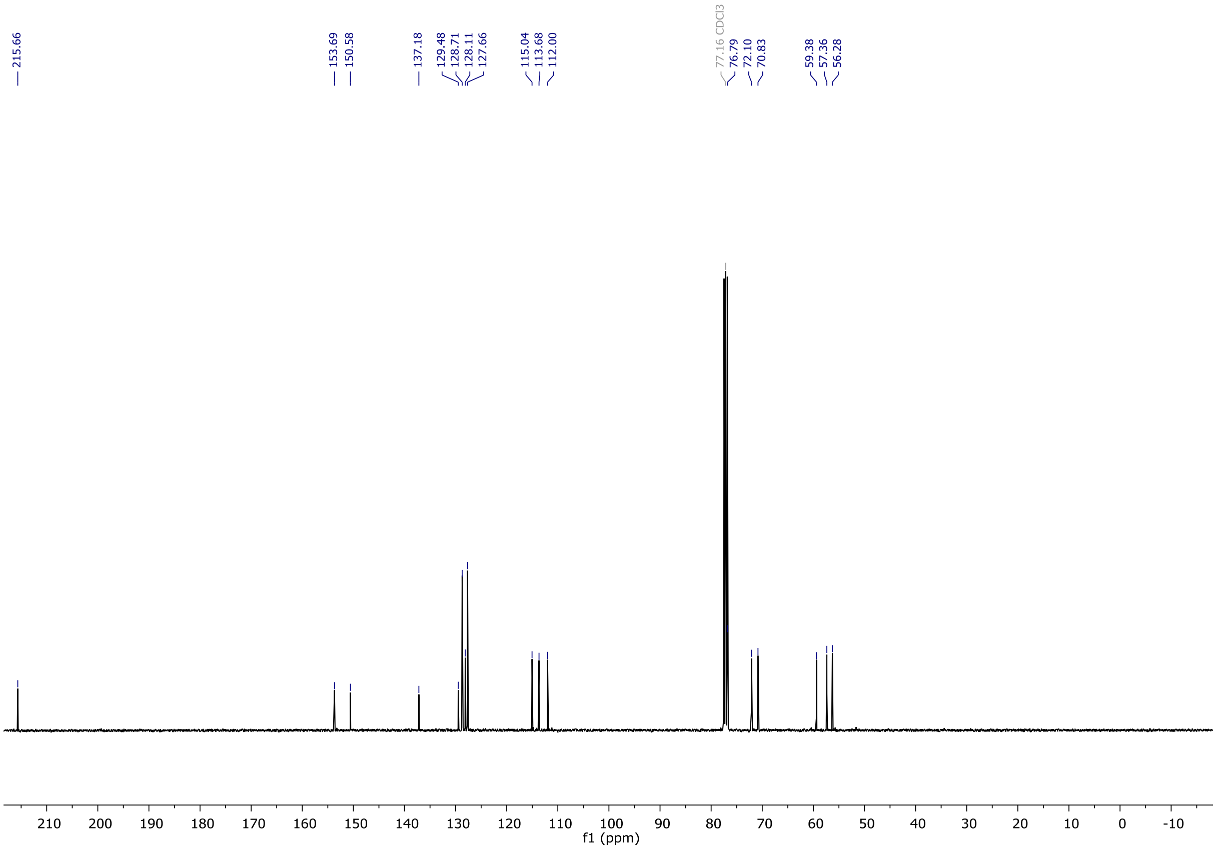

^{1}H NMR (400 MHz, $CDCl_3$) of diol **38**:

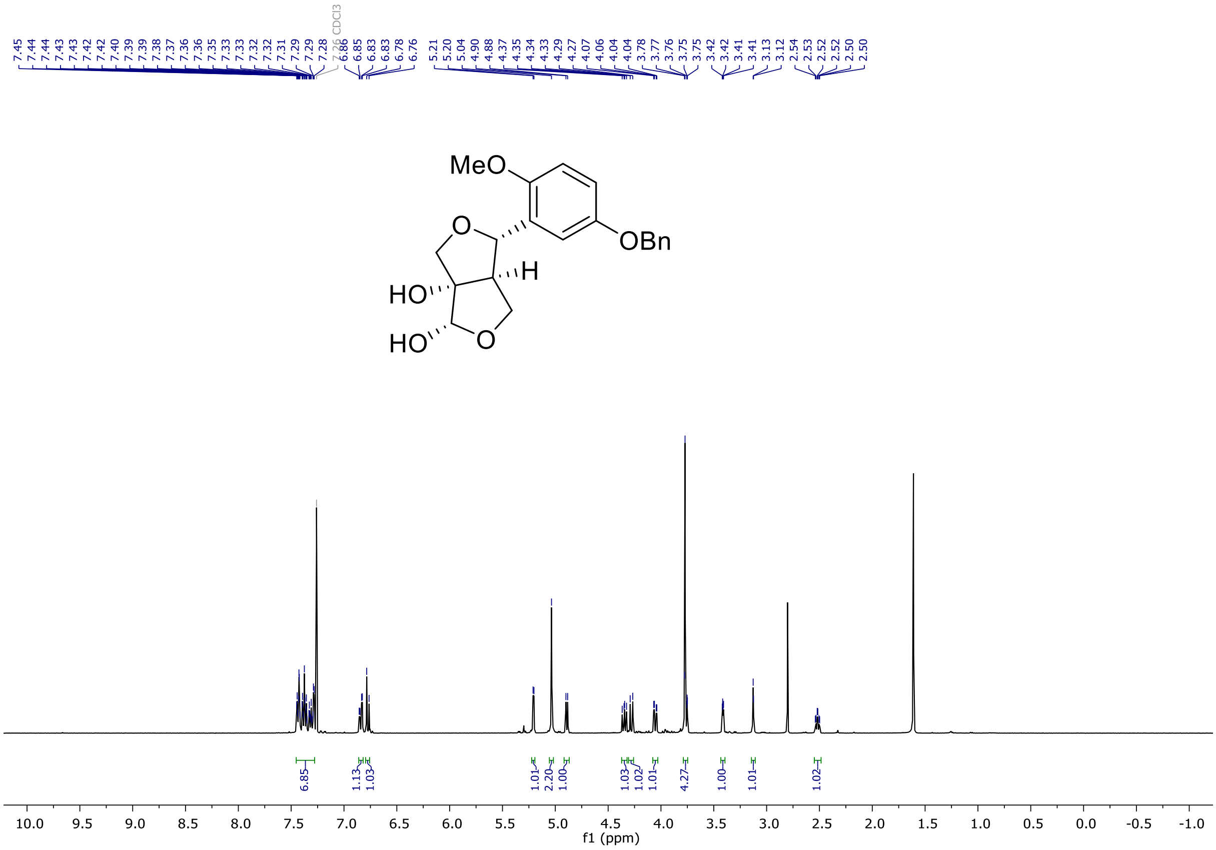

^{13}C NMR (101 MHz, $CDCl_3$) of diol **38:**

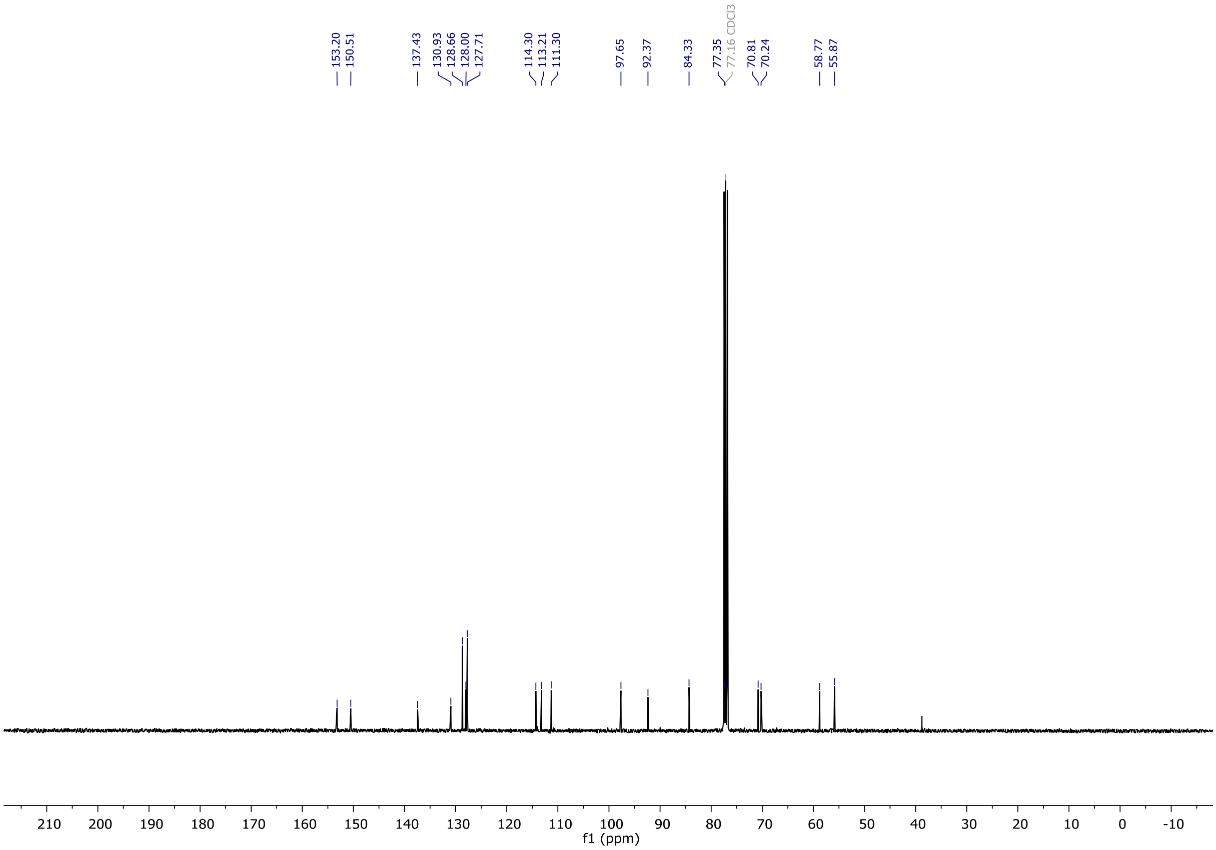

^{1}H NMR (400 MHz, $CDCl_3$) of acetal **39**:

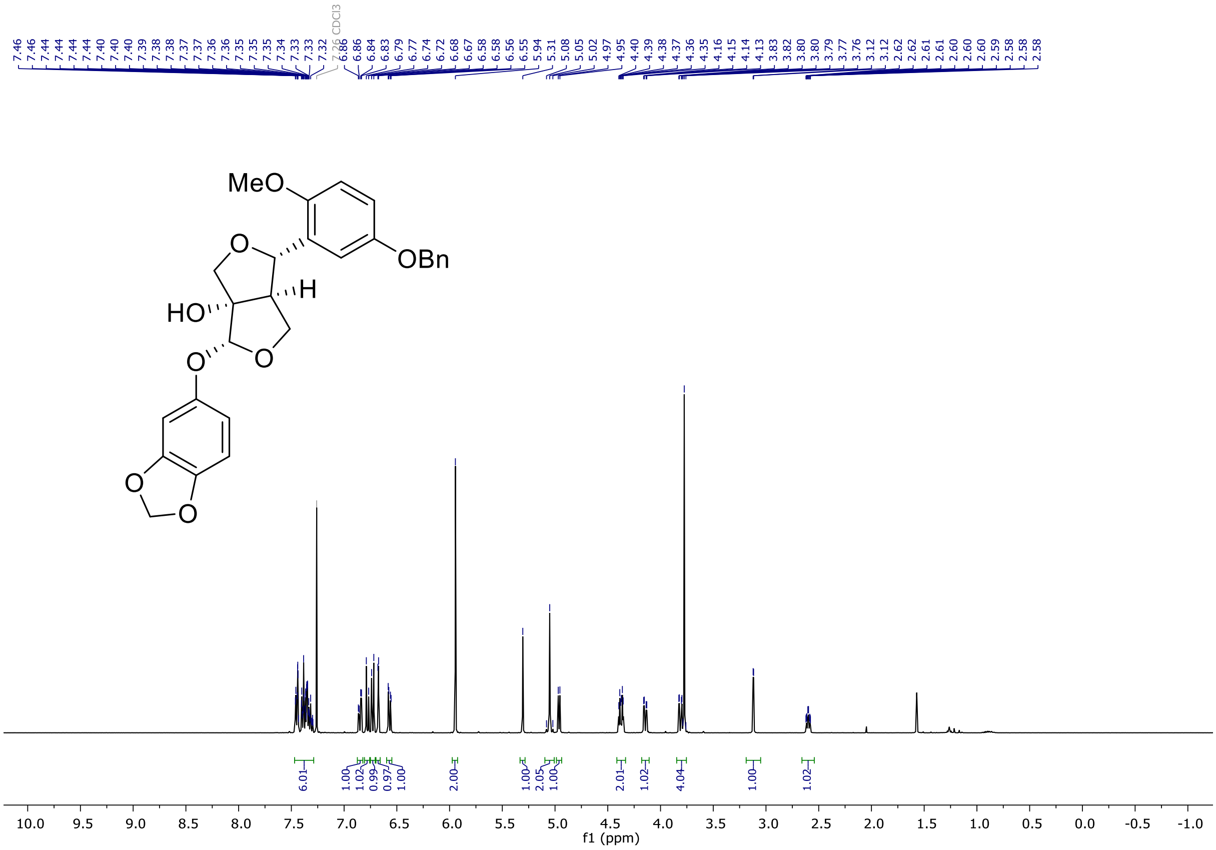

^{13}C NMR (101 MHz, $CDCl_3$) of acetal **39:**

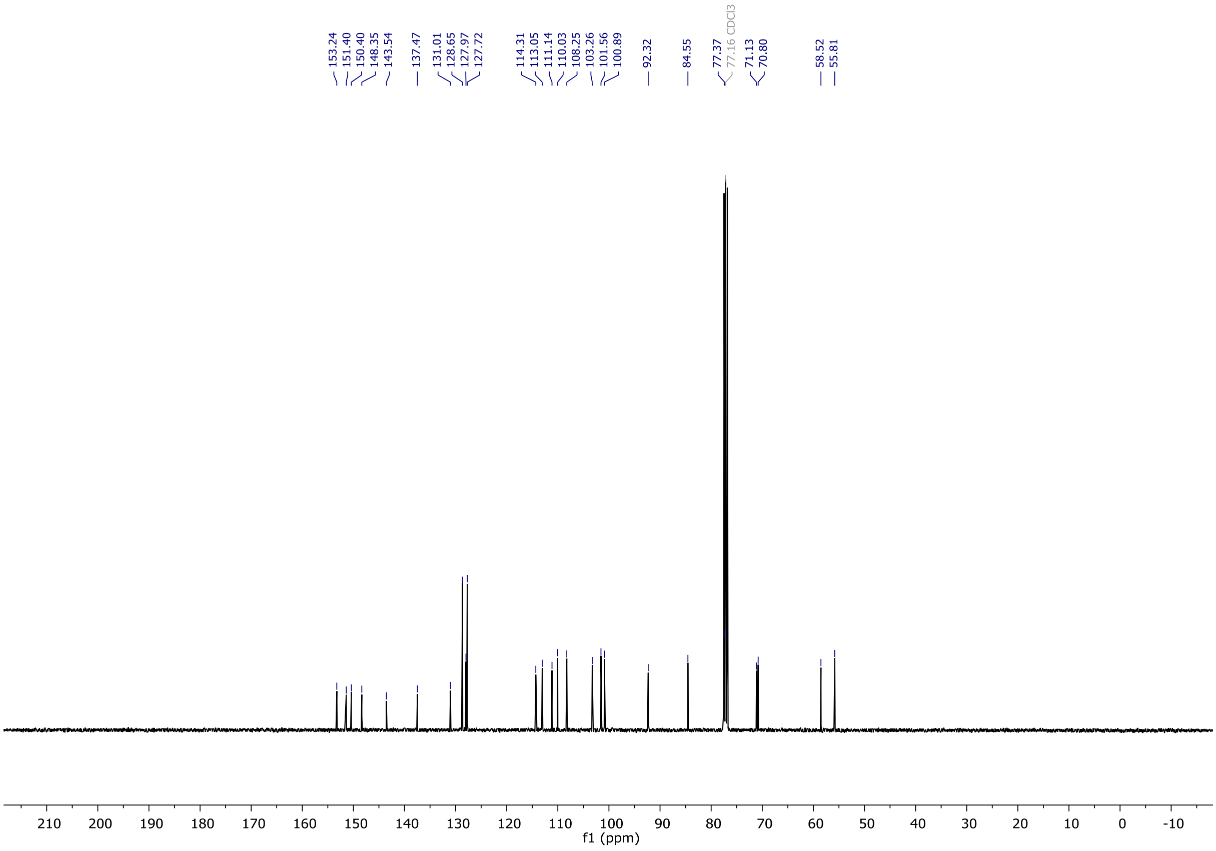

^{1}H NMR (400 MHz, $CDCl_3$) of phenol **45**:

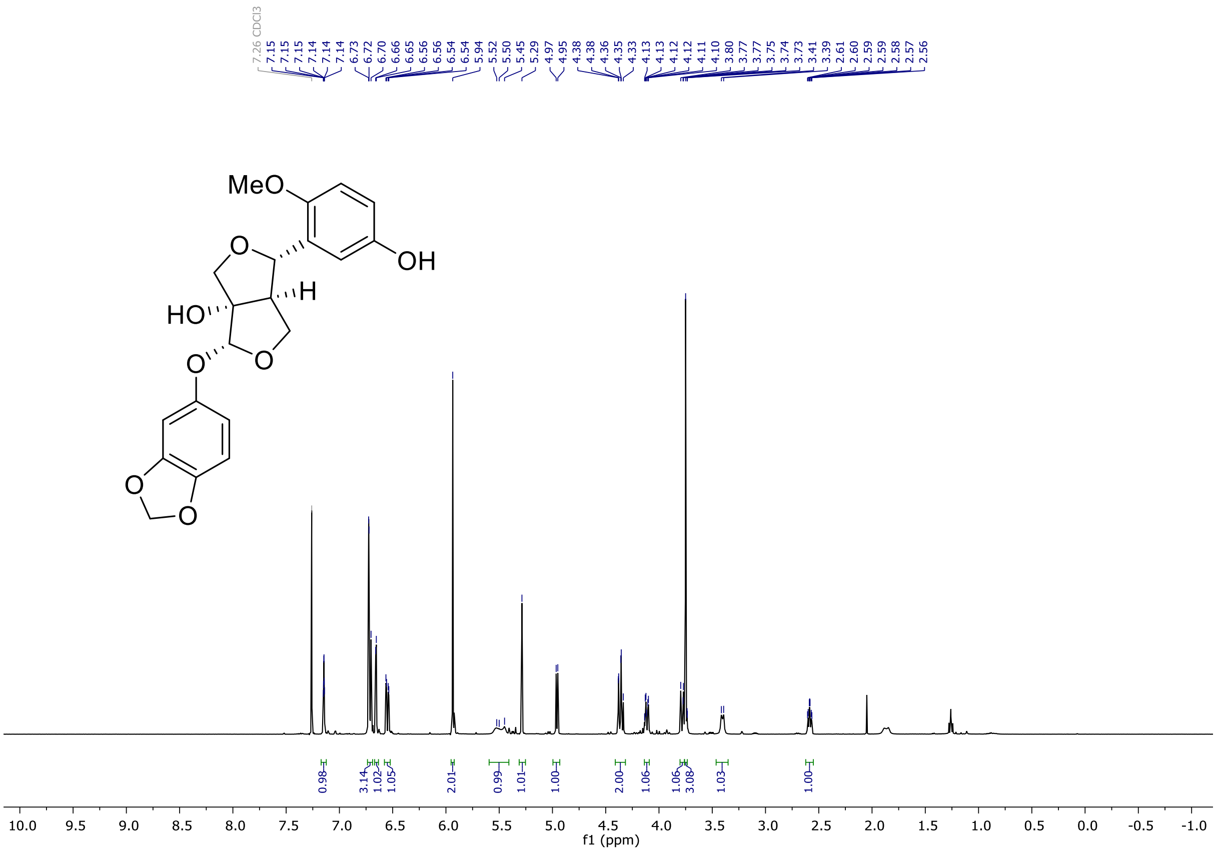

^{13}C NMR (101 MHz, $CDCl_3$) of phenol **45:**

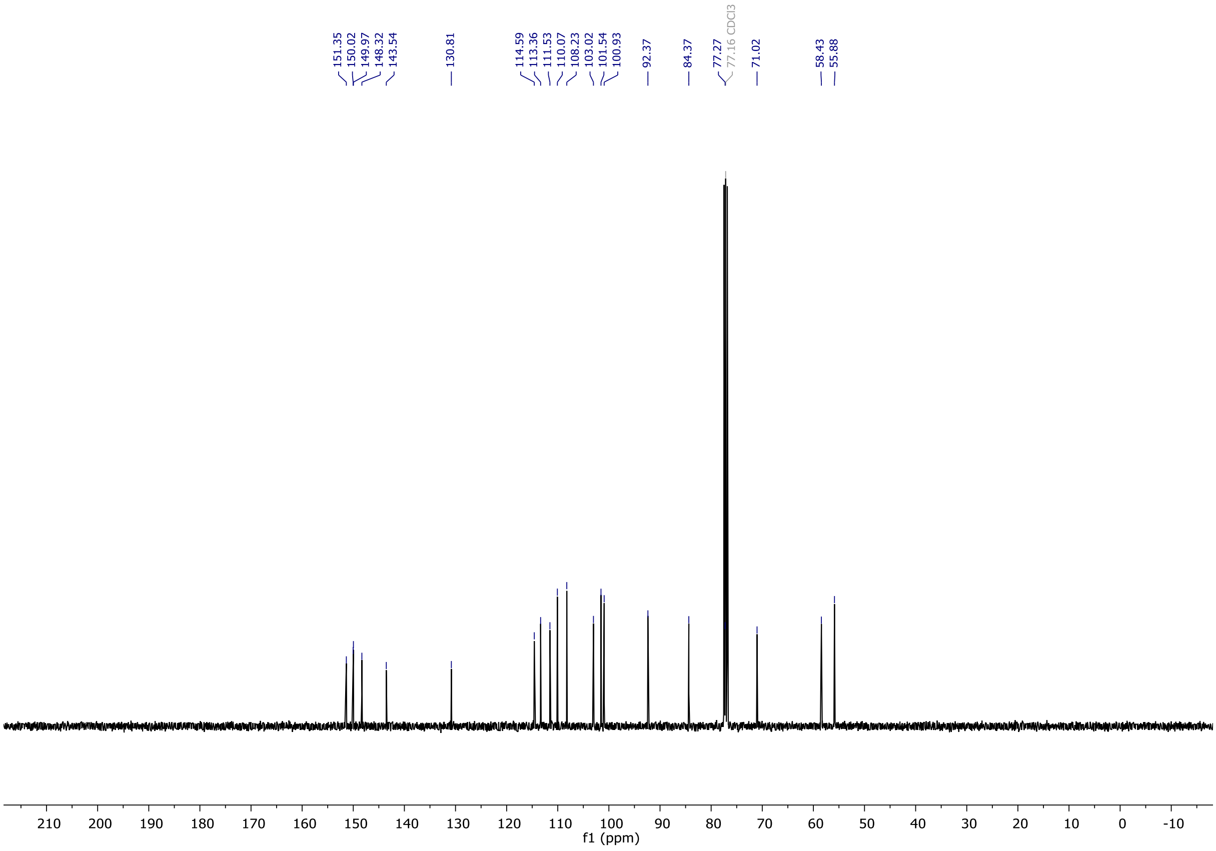

^{1}H NMR (500 MHz, $CDCl_3$) of catechol **47**:

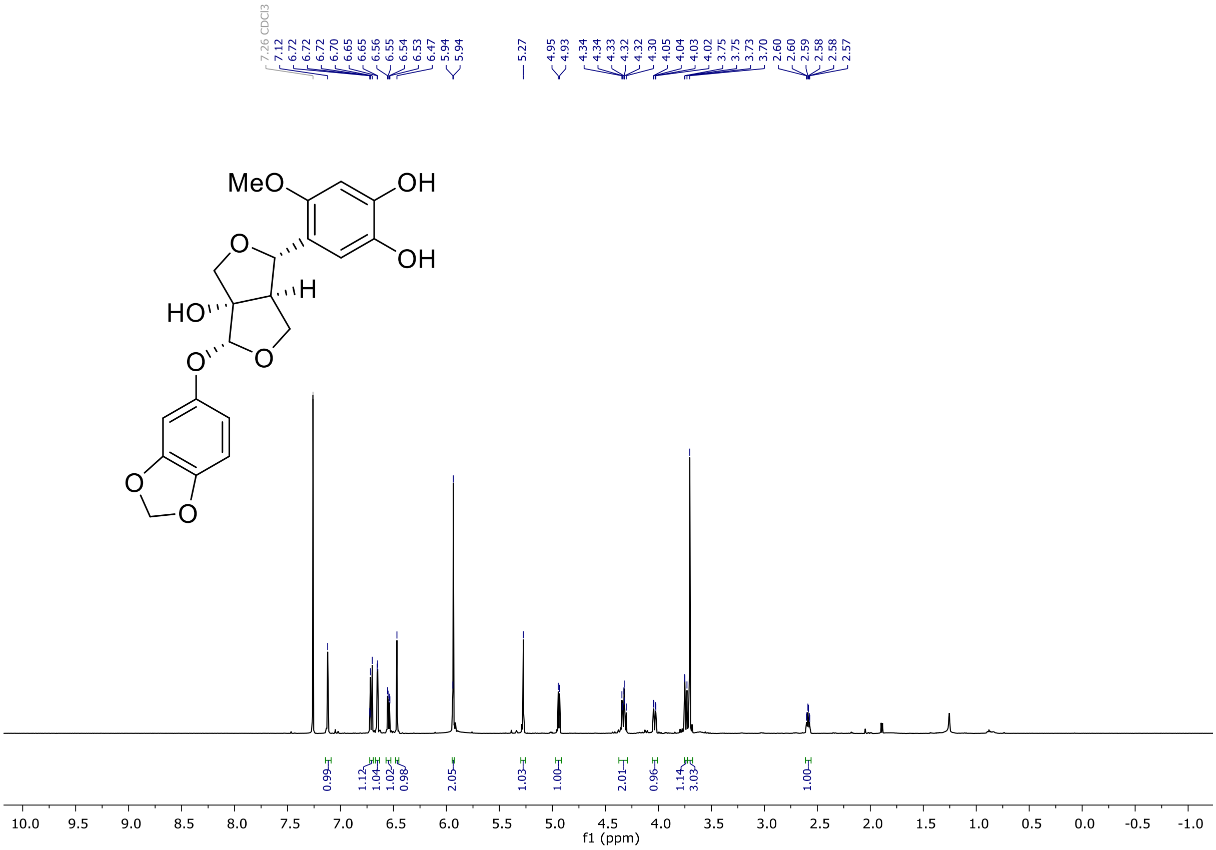

^{13}C NMR (126 MHz, $CDCl_3$) of catechol **47:**

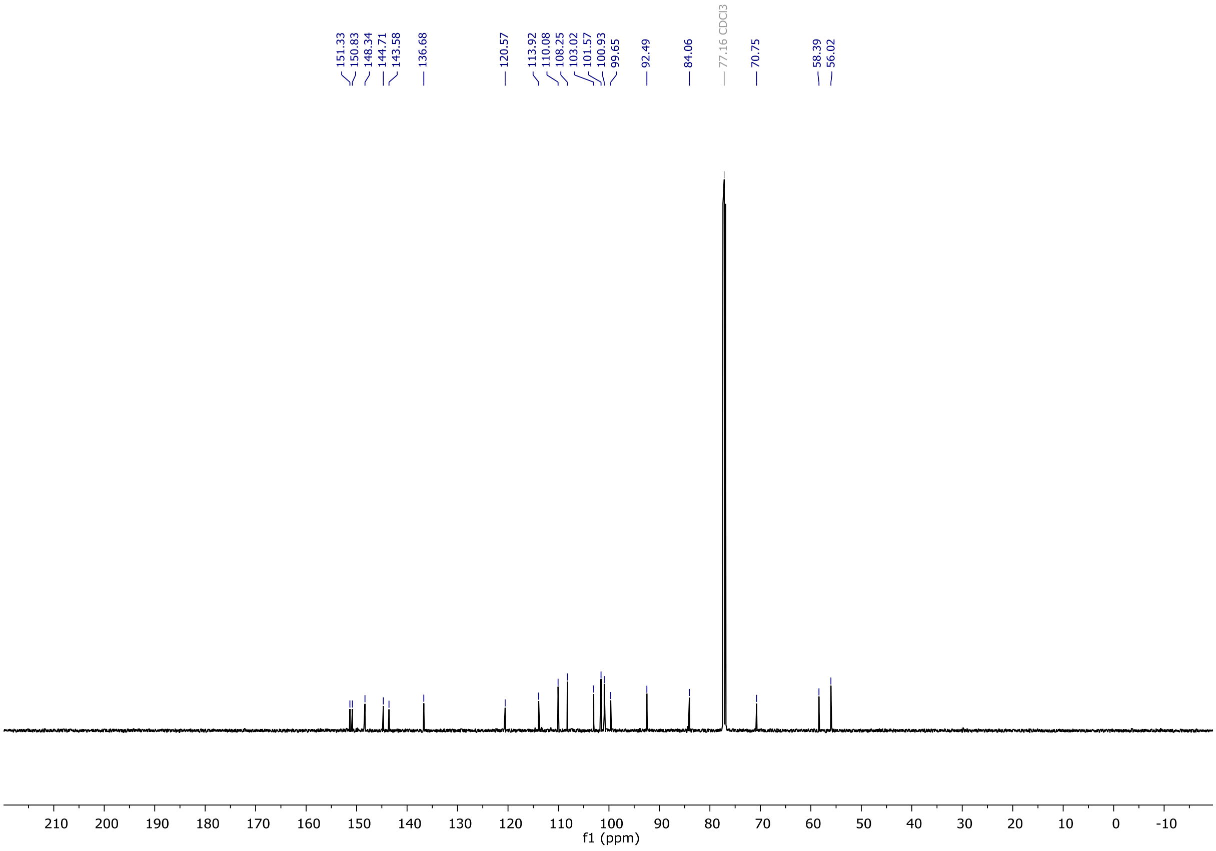

^{1}H NMR (400 MHz, $CDCl_3$) of carbonate **54**:

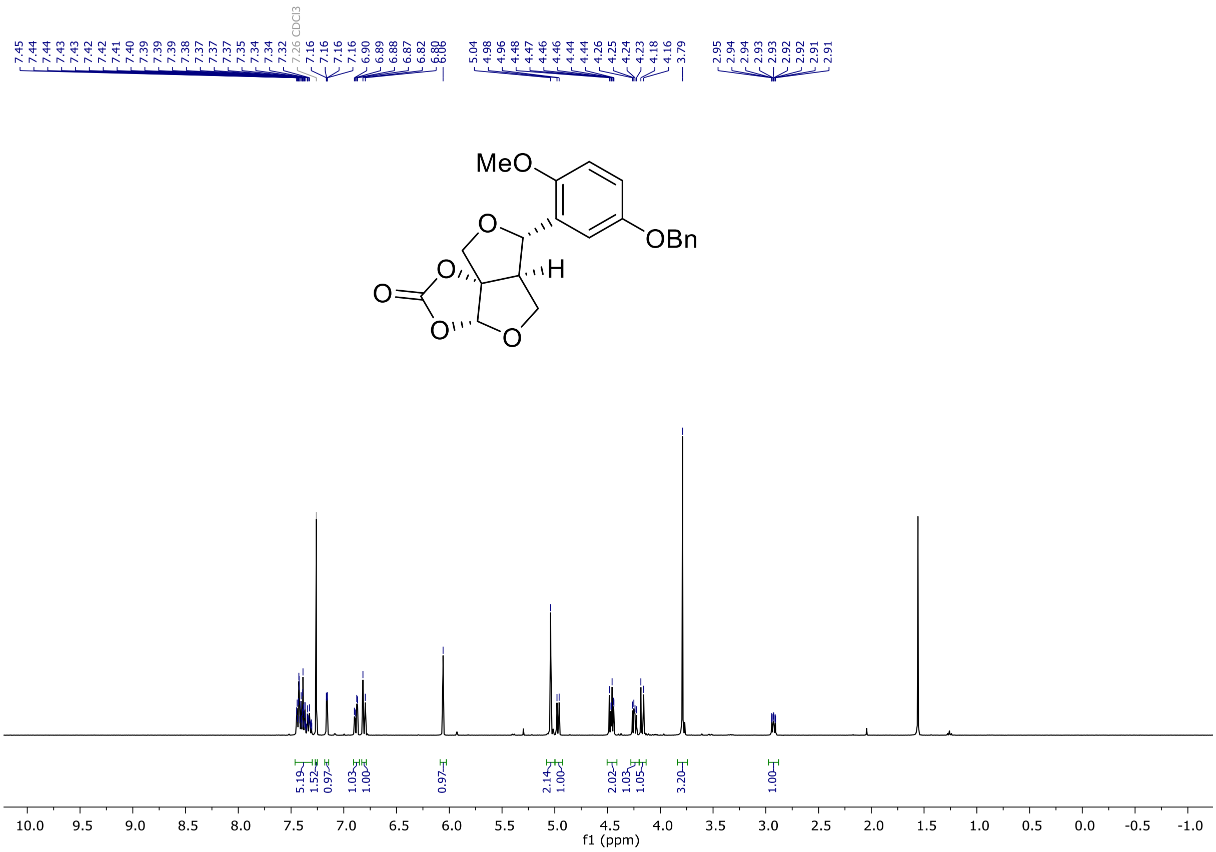

^{13}C NMR (101 MHz, $CDCl_3$) of carbonate **54:**

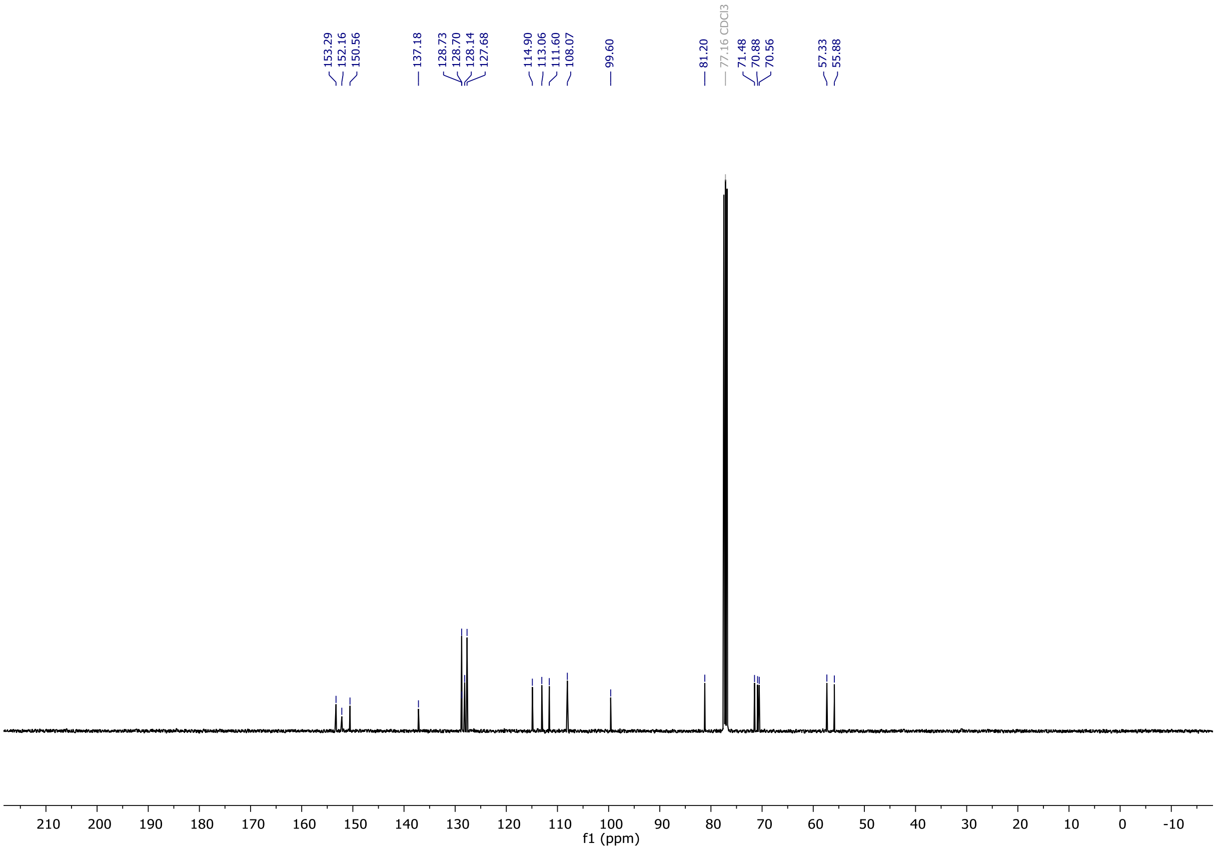

^{1}H NMR (400 MHz, $CDCl_3$) of phenol **55**:

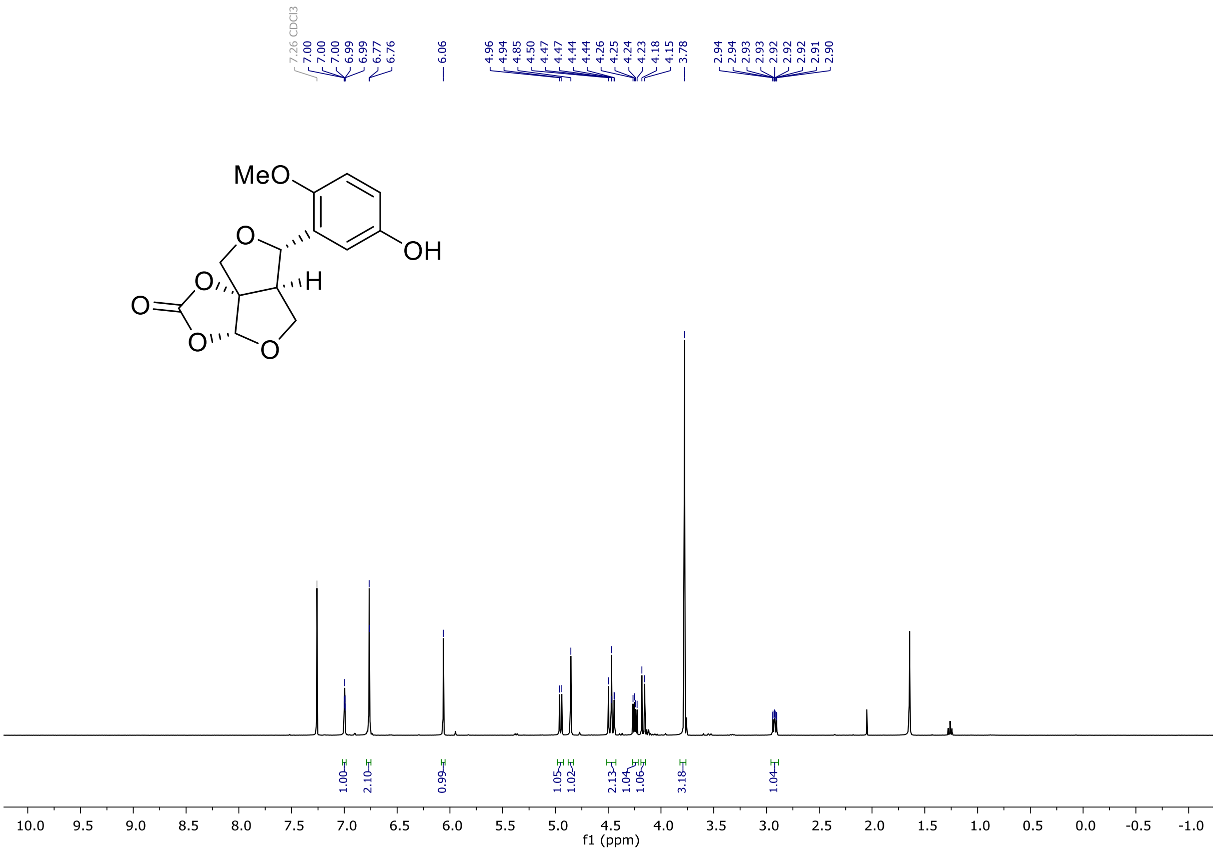

^{13}C NMR (101 MHz, $CDCl_3$) of phenol **55:**

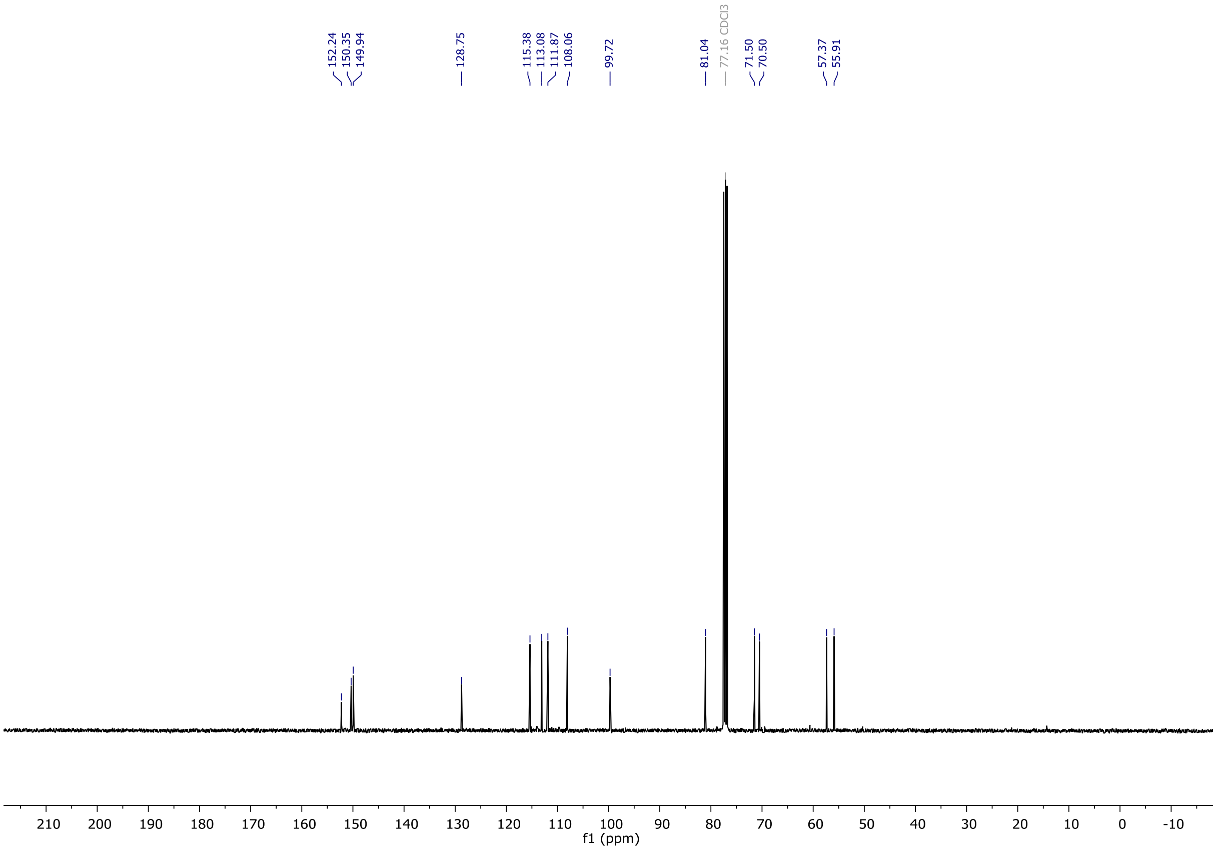

^{1}H NMR (400 MHz, CD_3OD) of alcohol **56**:

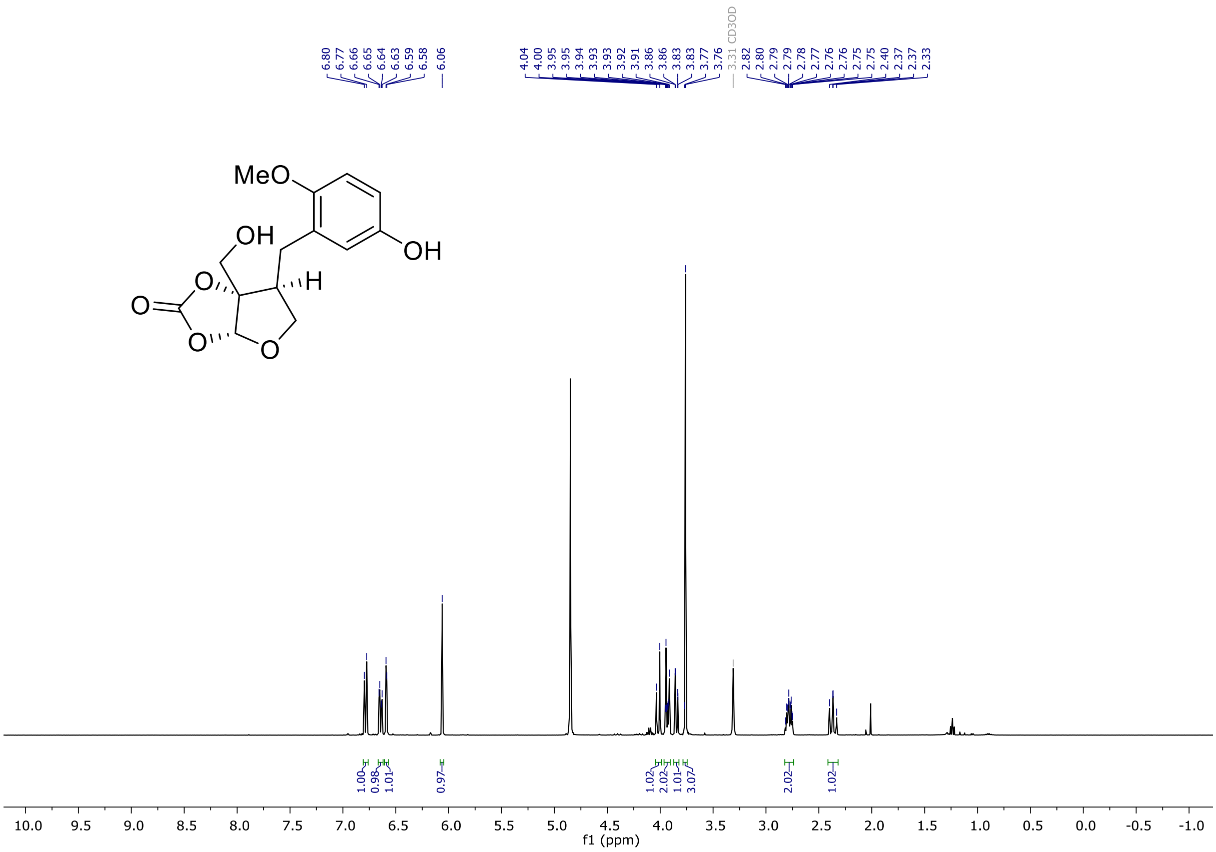

^{13}C NMR (101 MHz, CD_3OD) of alcohol **56:**

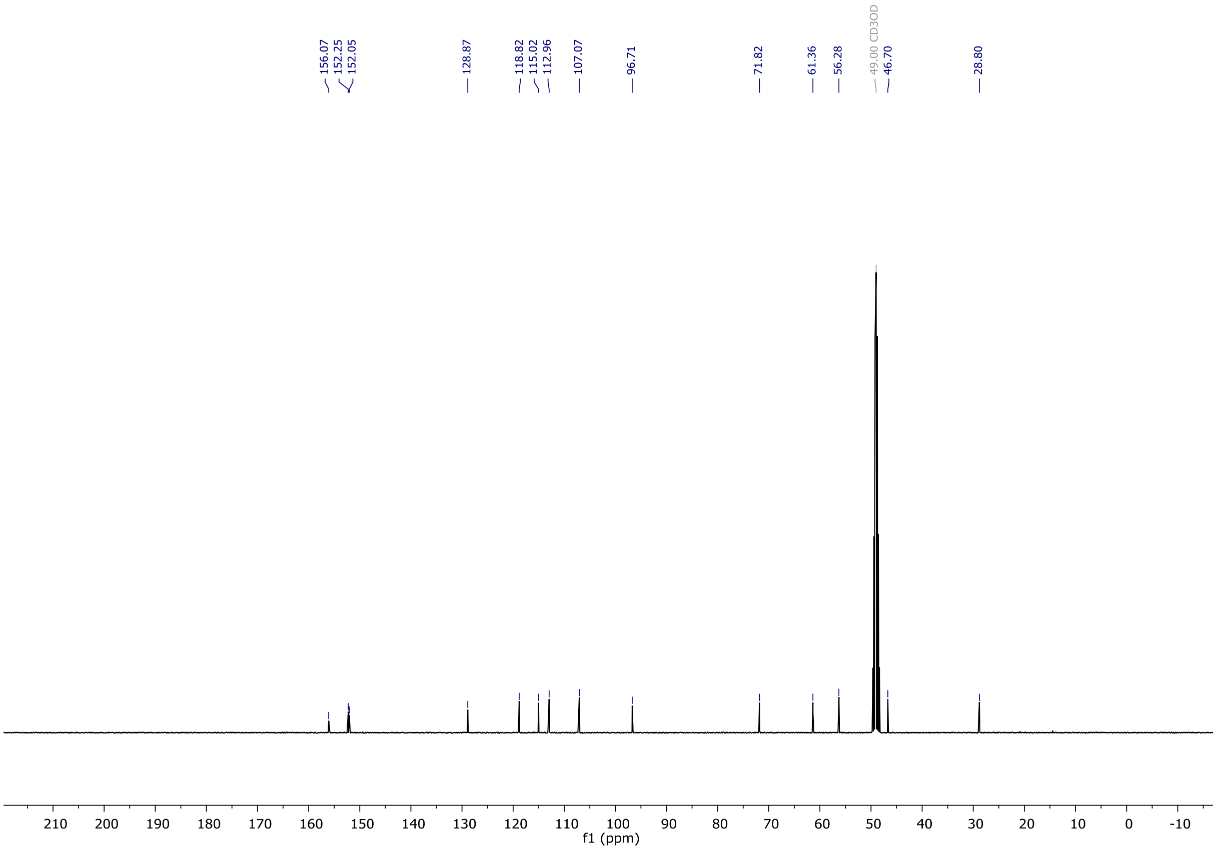

^{1}H NMR (400 MHz, $CDCl_3$) of *ortho*-quinone **14**:

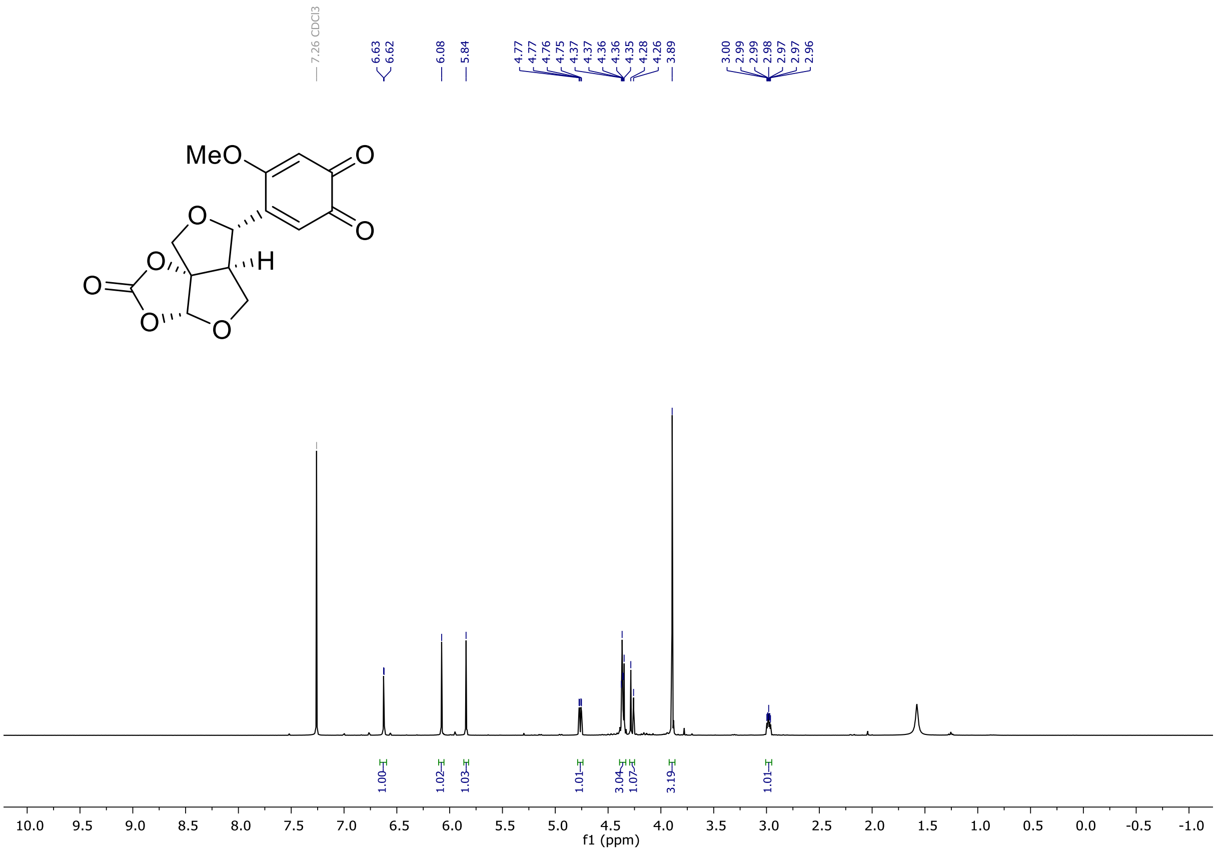

^{13}C NMR (101 MHz, $CDCl_3$) of *ortho*-quinone **14:**

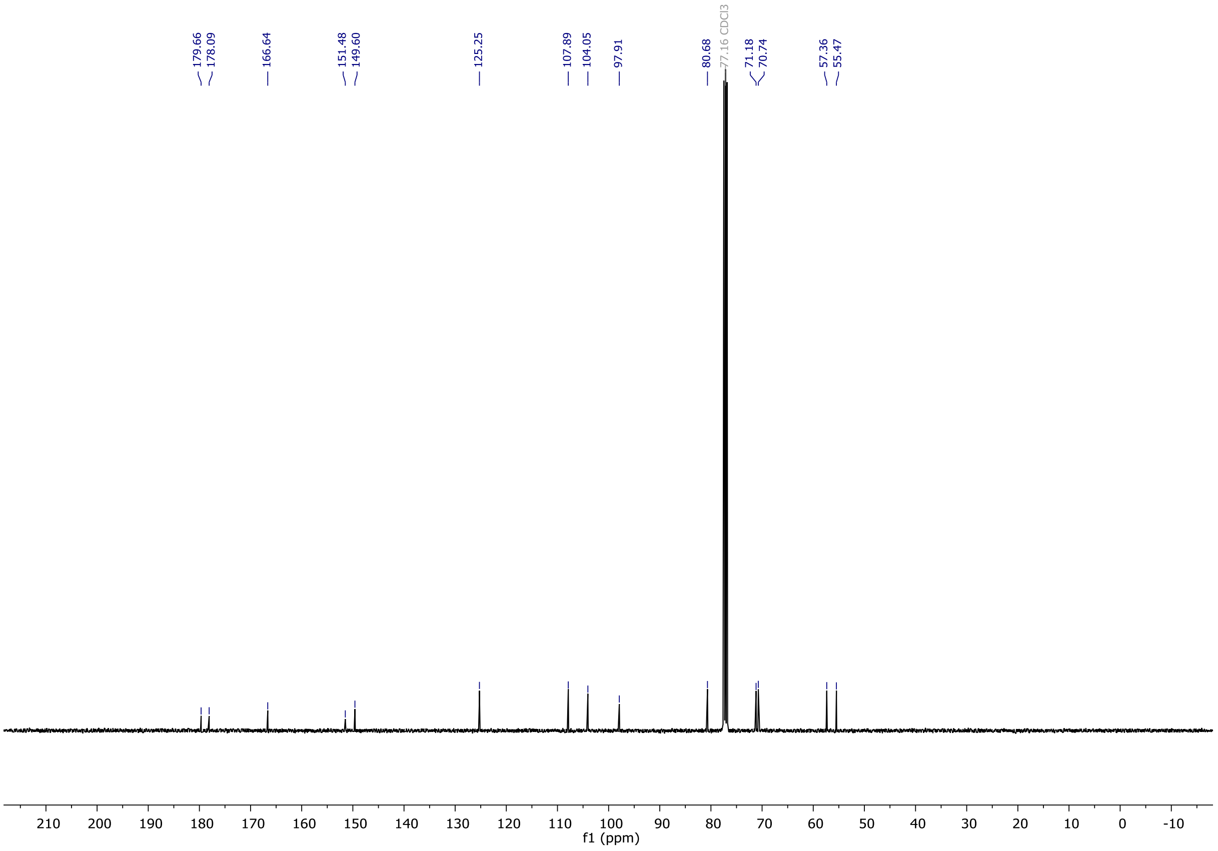

^{1}H NMR (400 MHz, $CDCl_3$) of styrene **SI-10a**:

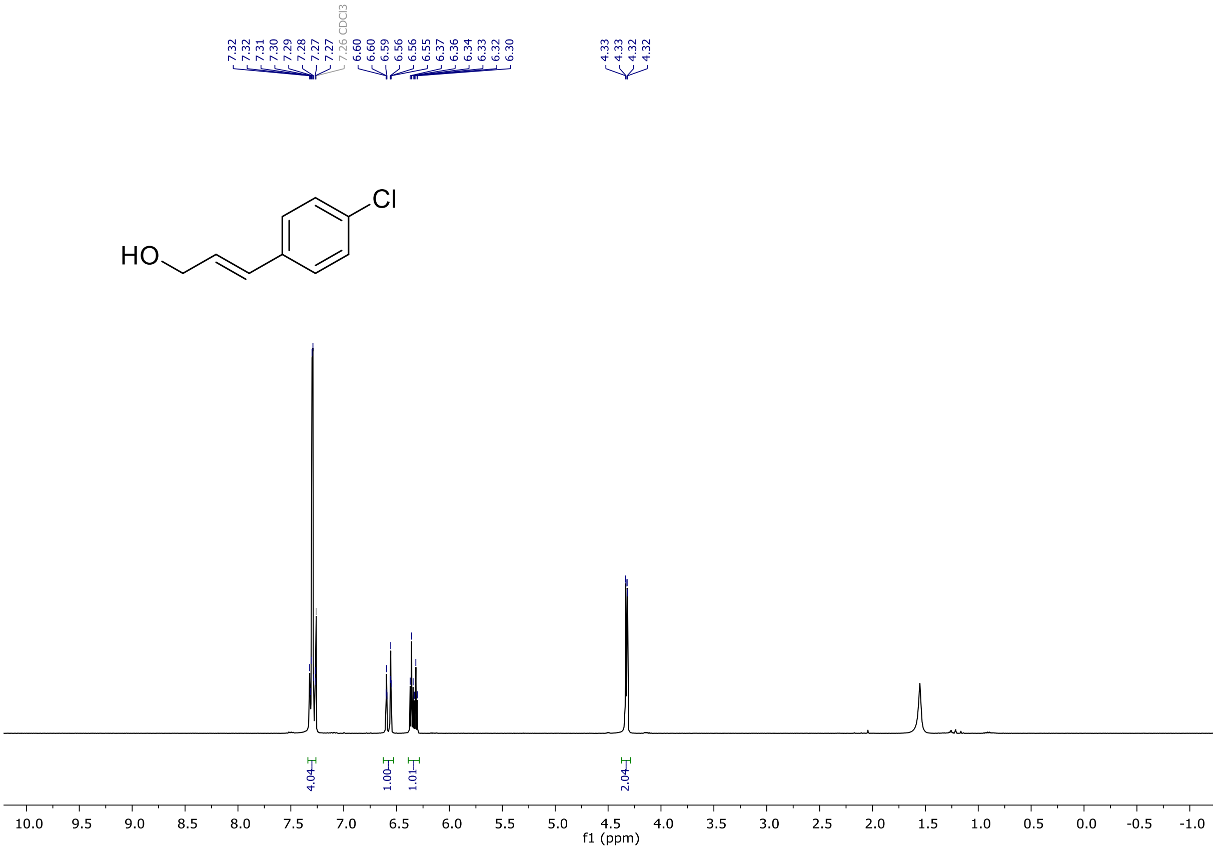

^{1}H NMR (400 MHz, $CDCl_3$) of styrene **SI-10b**:

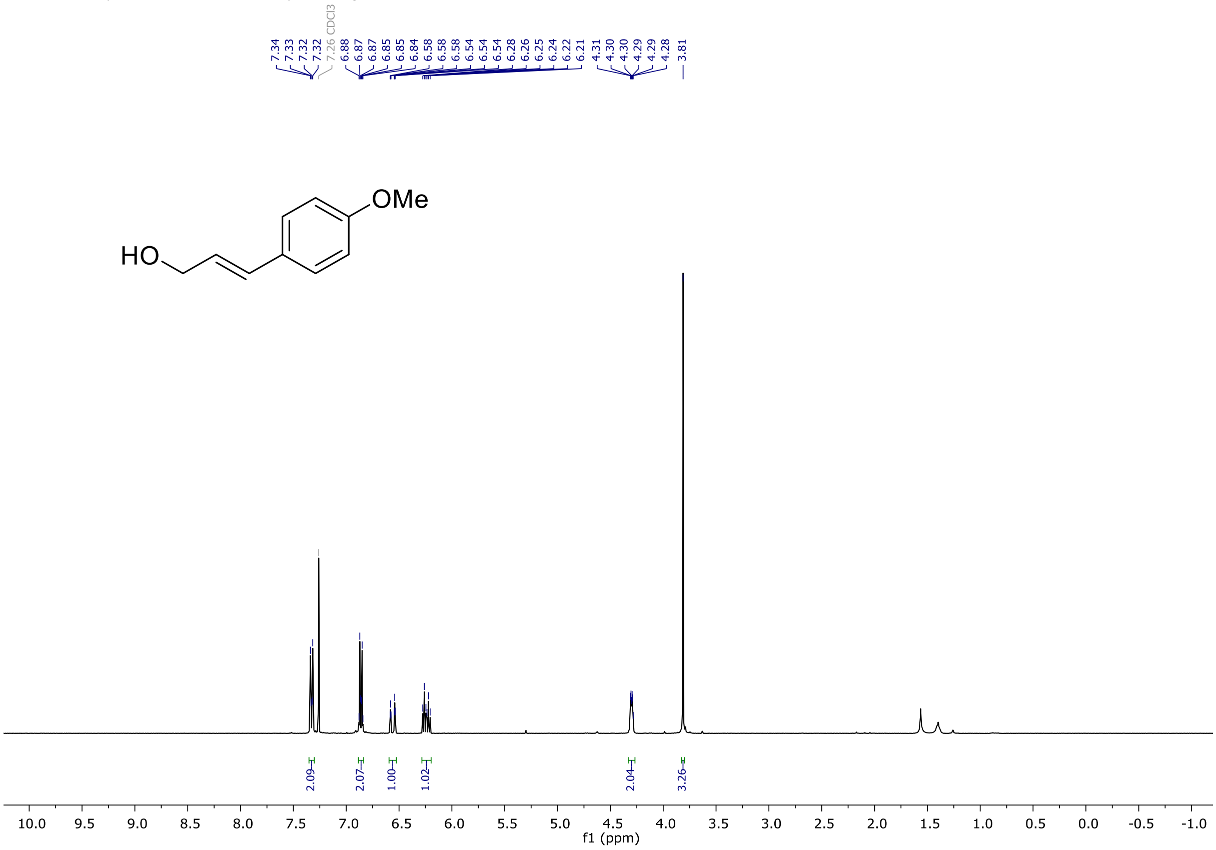

^{1}H NMR (400 MHz, $CDCl_3$) of styrene **SI-10c**:

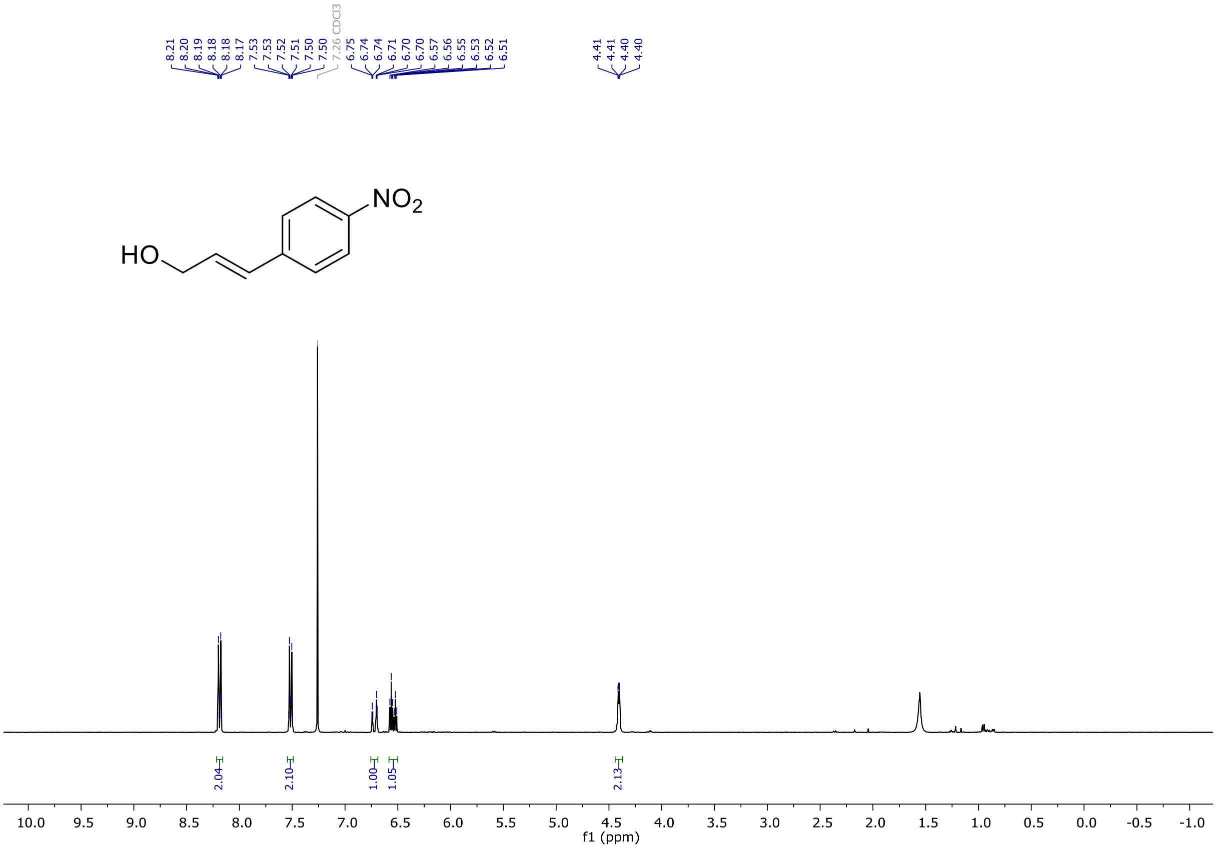

^{1}H NMR (400 MHz, $CDCl_3$) of styrene **SI-10d**:

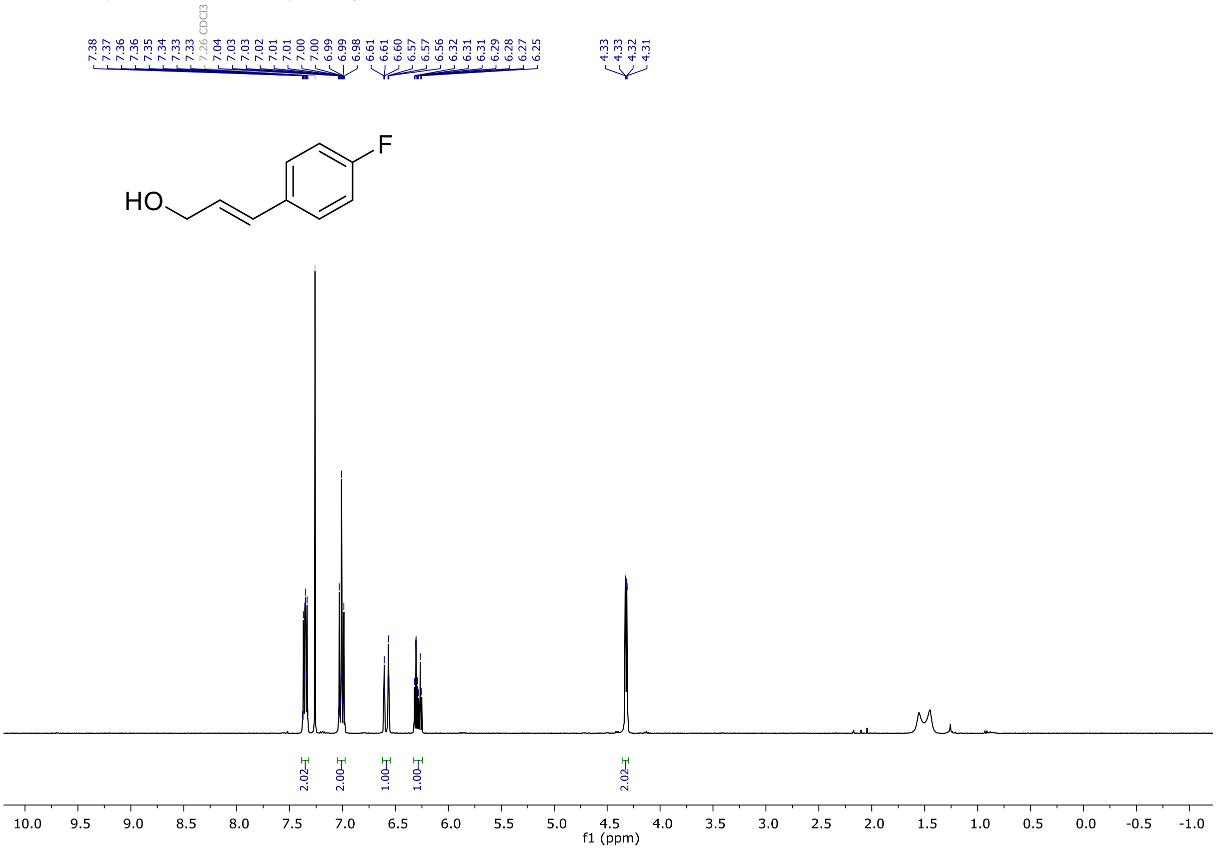

^{1}H NMR (400 MHz, $CDCl_3$) of aldehyde **SI-9a**:

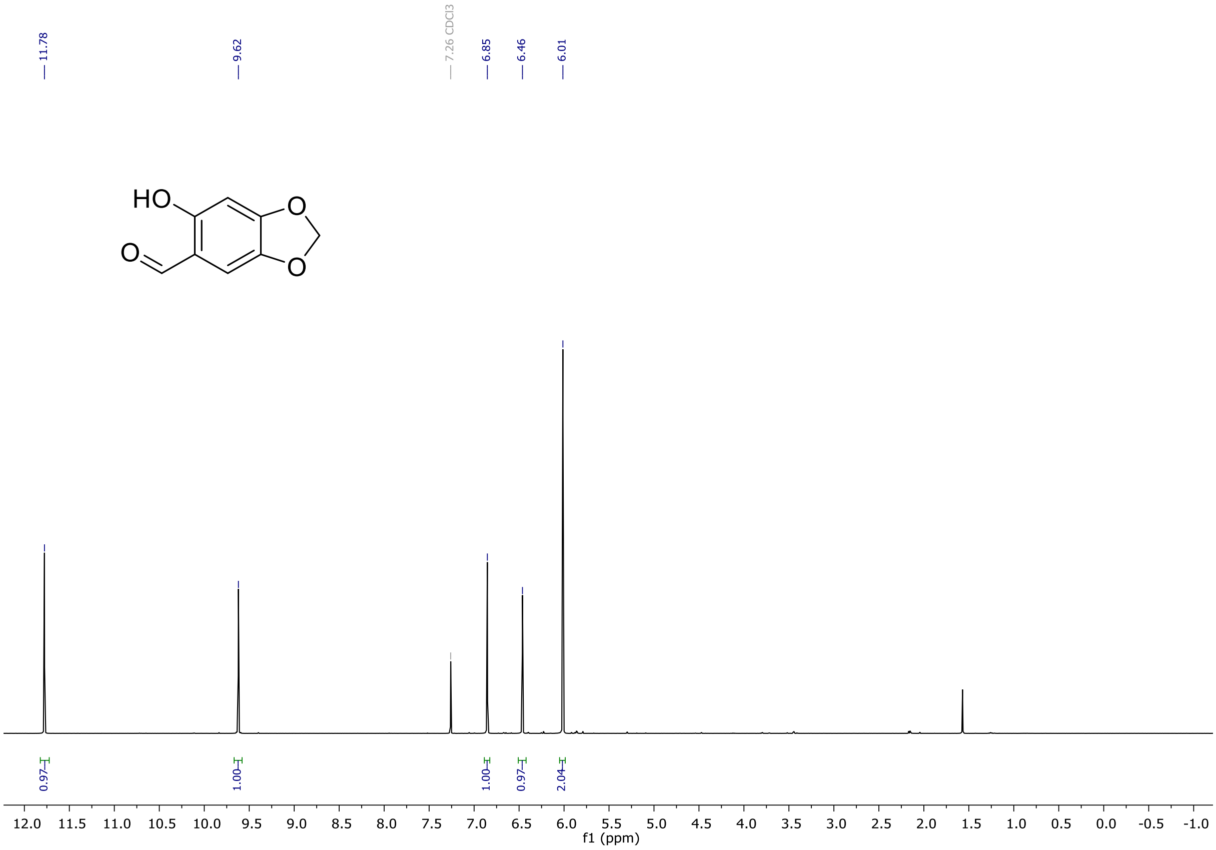

^{1}H NMR (500 MHz, $CDCl_3$) of styrene **SI-10e**:

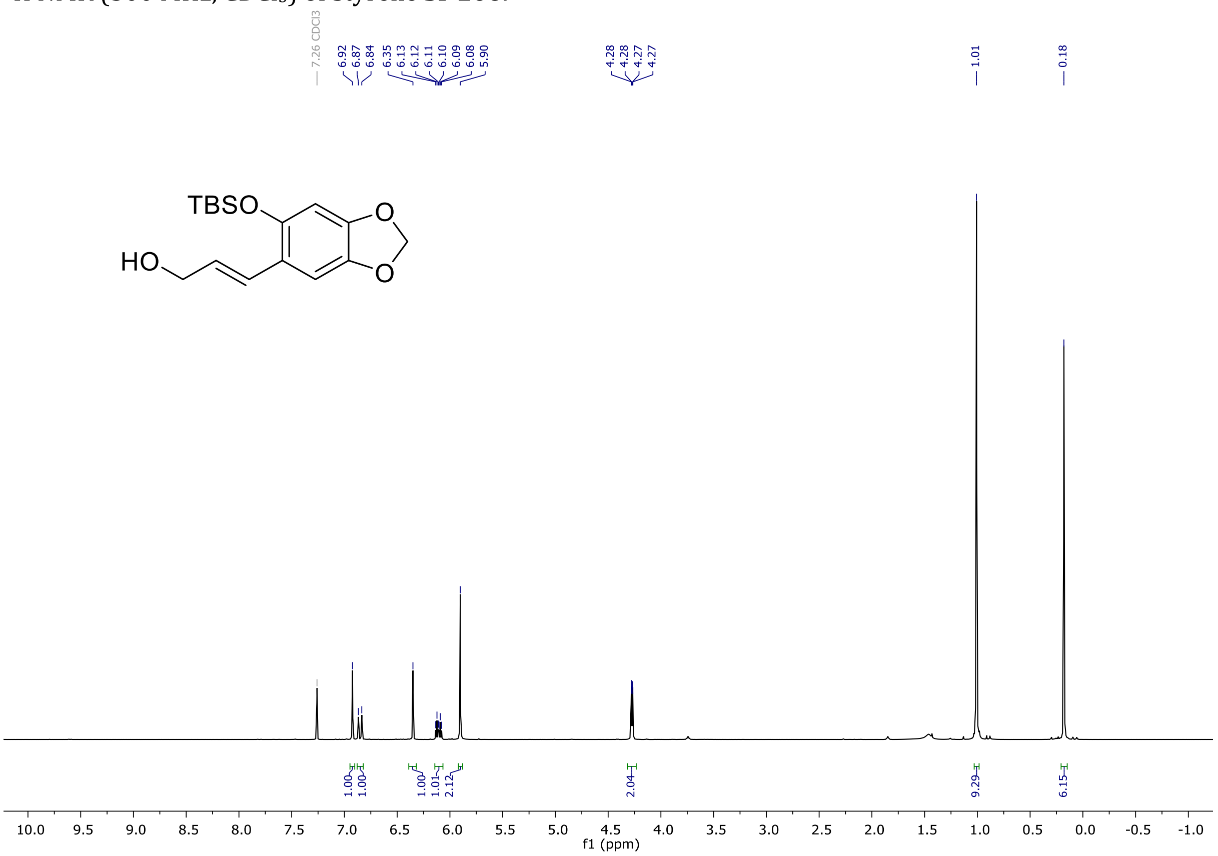

^{13}C NMR (126 MHz, $CDCl_3$) of styrene **SI-10e**:

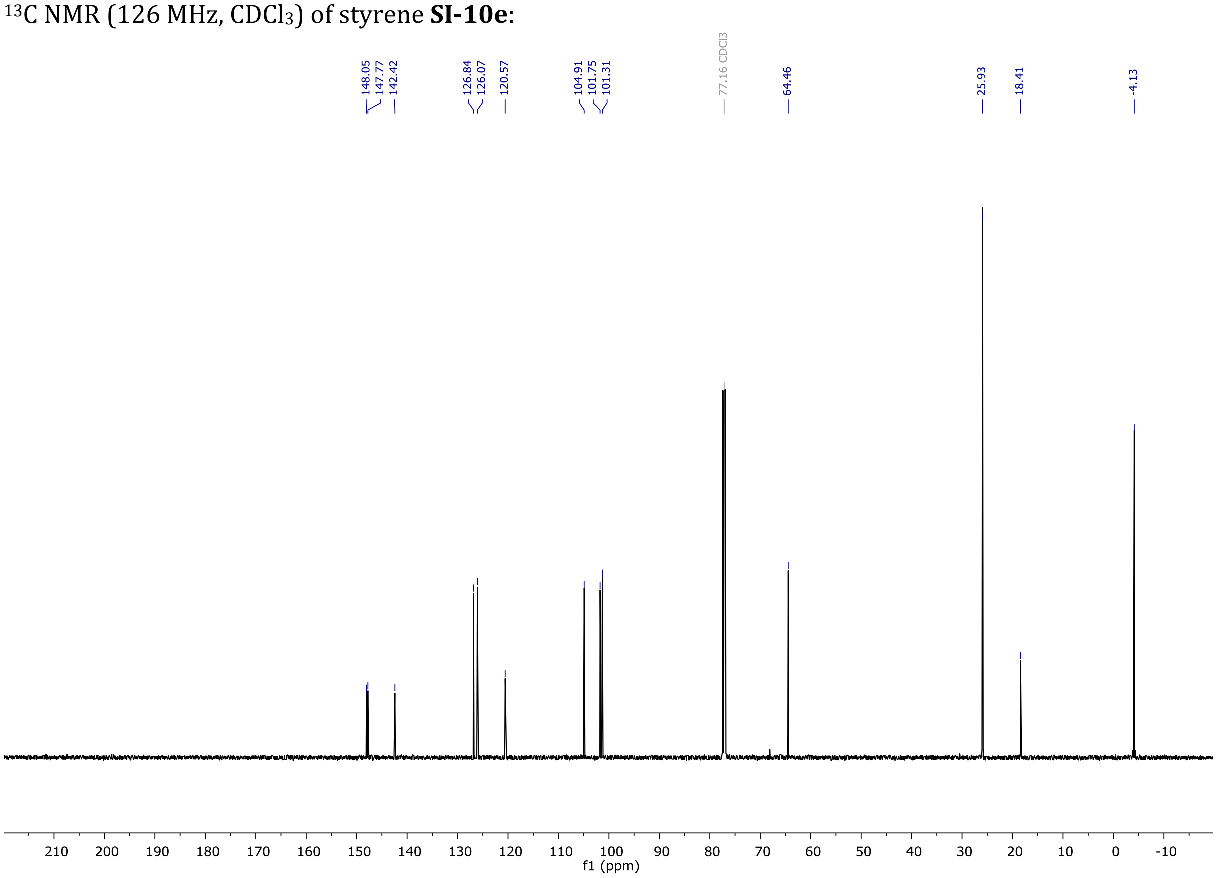

^{1}H NMR (500 MHz, acetone-d_6) of styrene **13b**:

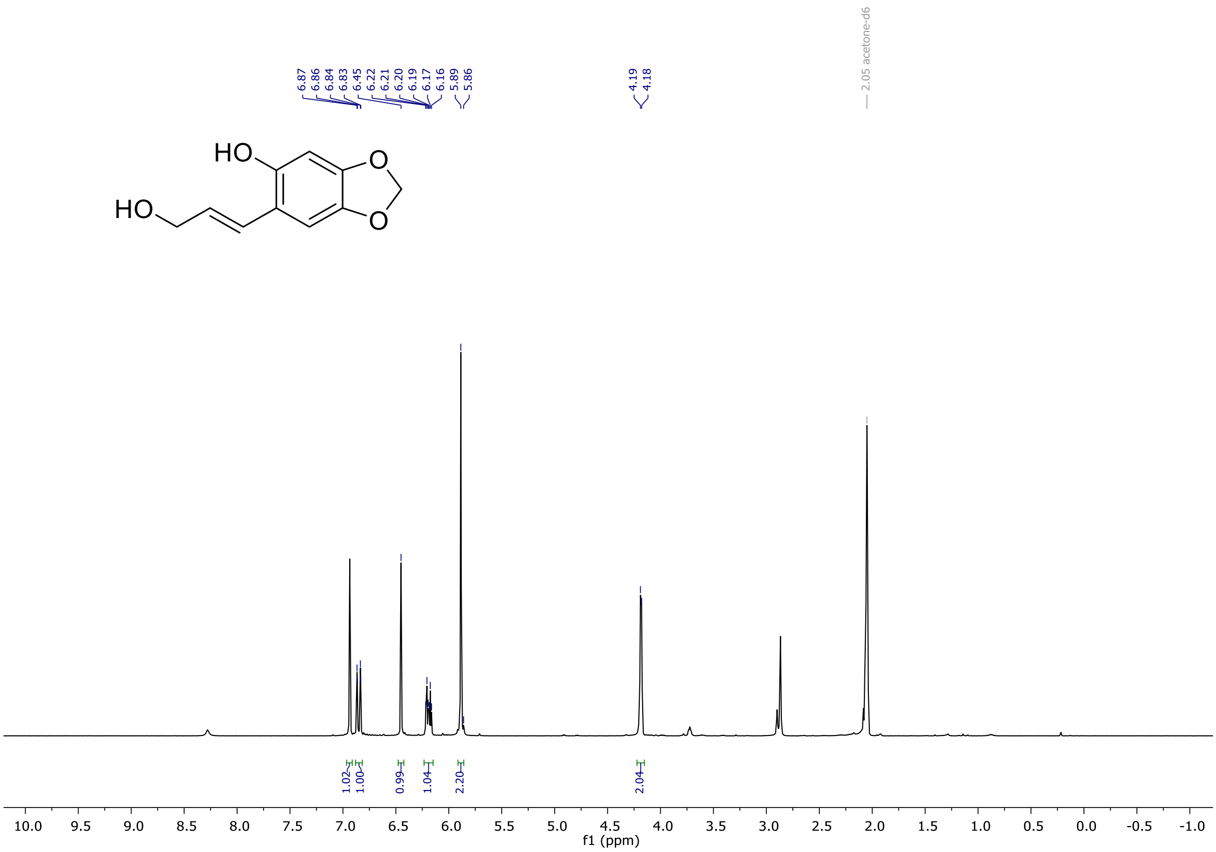

^{13}C NMR (126 MHz, acetone-d_6) of styrene **13b**:

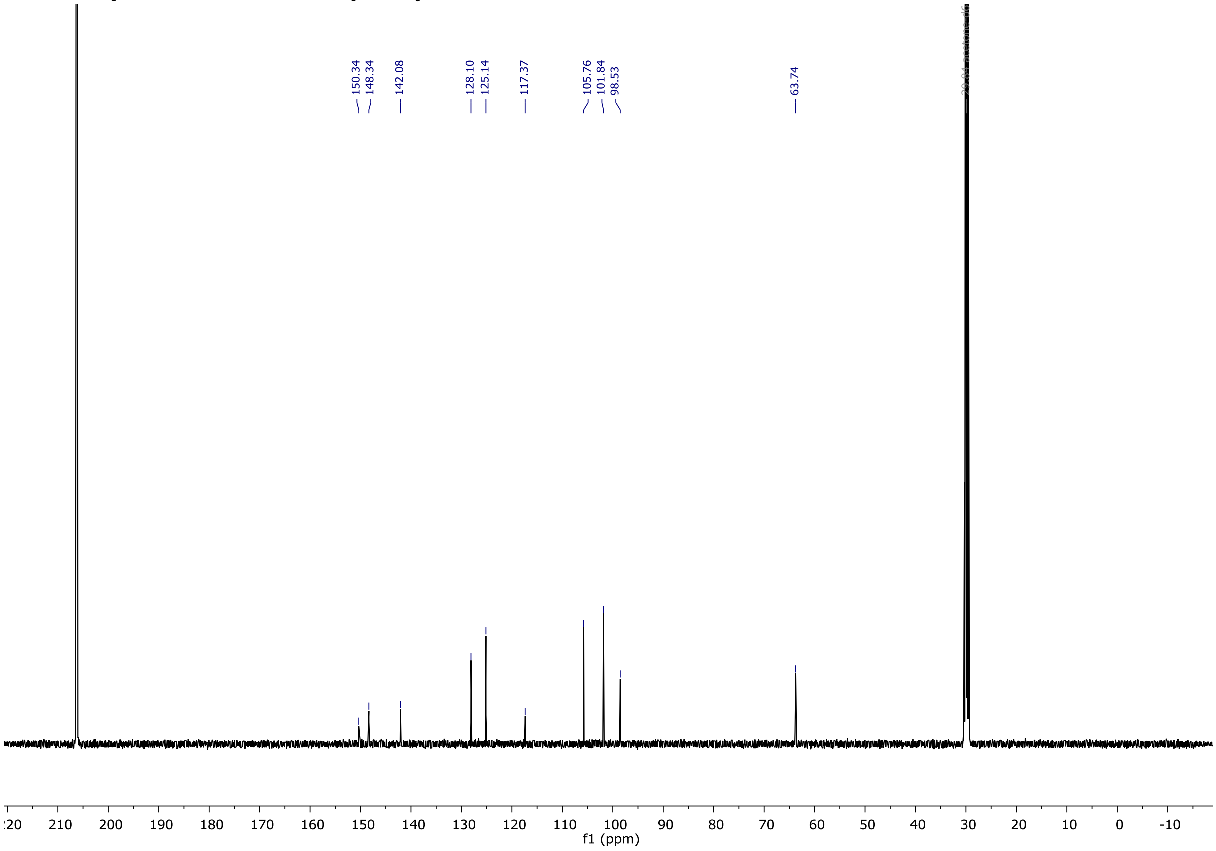

^{1}H NMR (400 MHz, $CDCl_3$) of styrene **13a**:

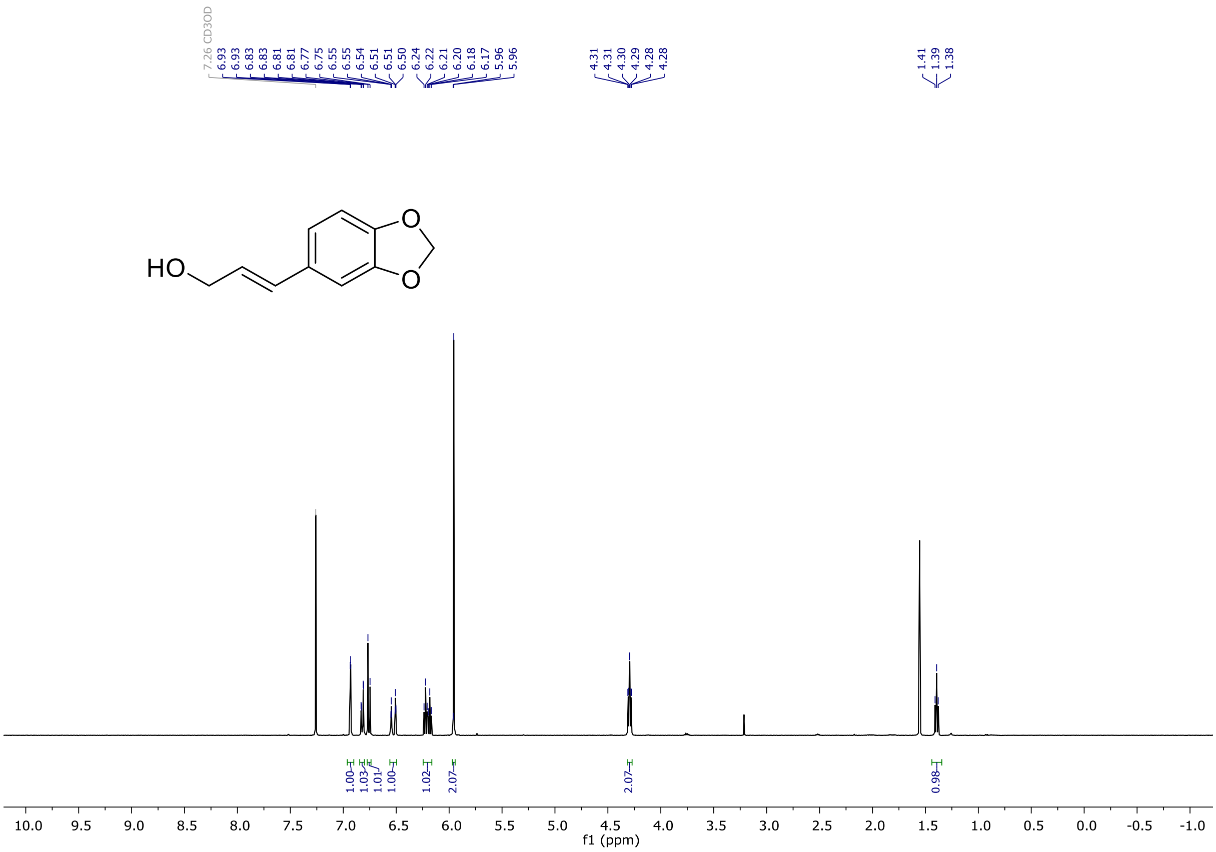

^{1}H NMR (400 MHz, $CDCl_3$) of styrene **13e**:

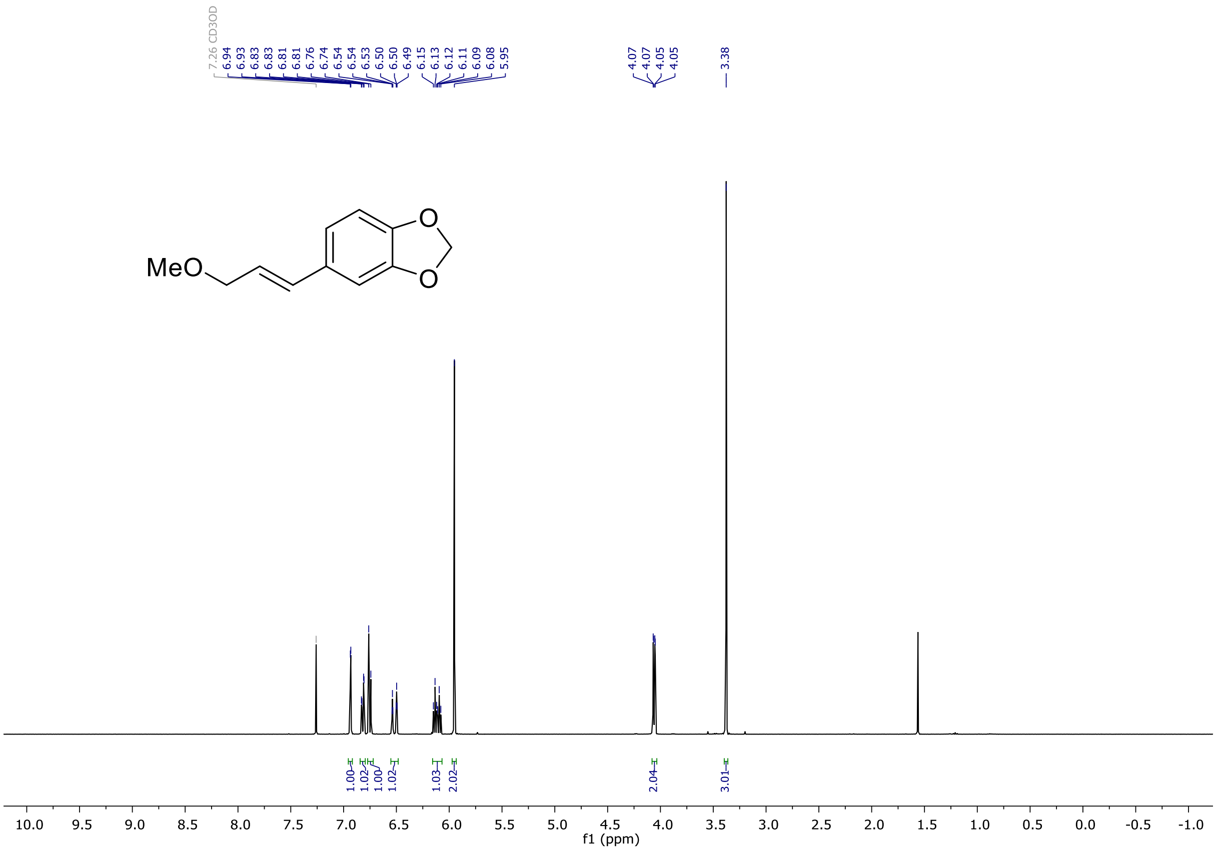

^{1}H NMR (400 MHz, $CDCl_3$) of styrene **SI-12**:

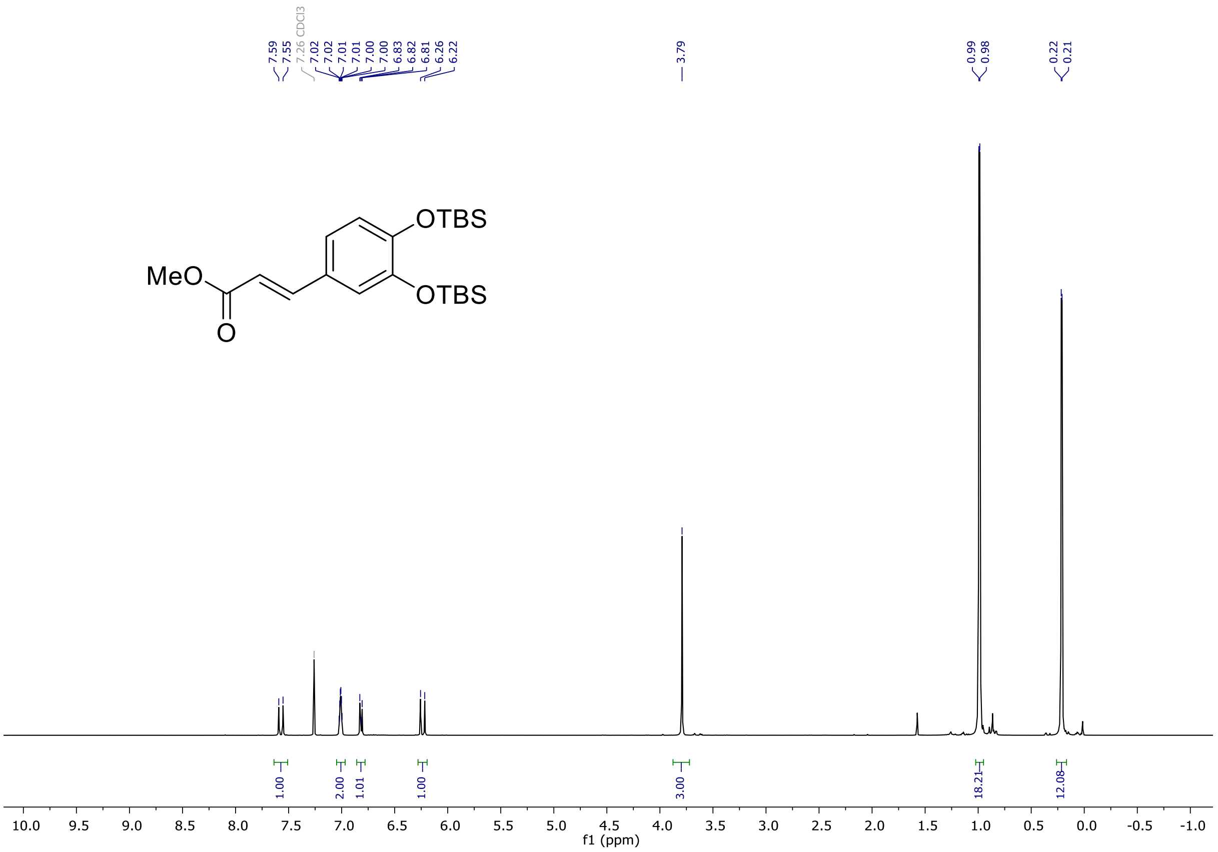

^{1}H NMR (400 MHz, $CDCl_3$) of styrene **SI-13**:

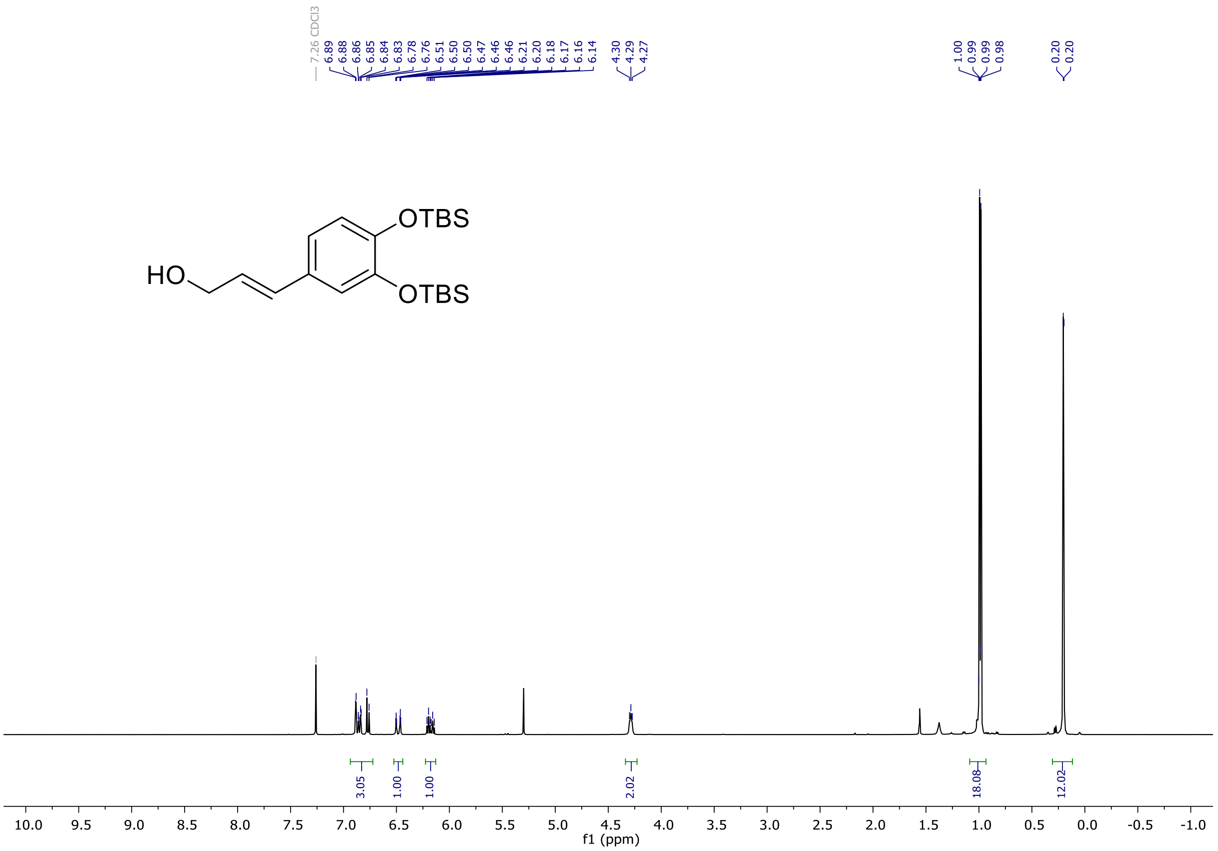

^{1}H NMR (400 MHz, $CDCl_3$) of styrene **SI-14**:

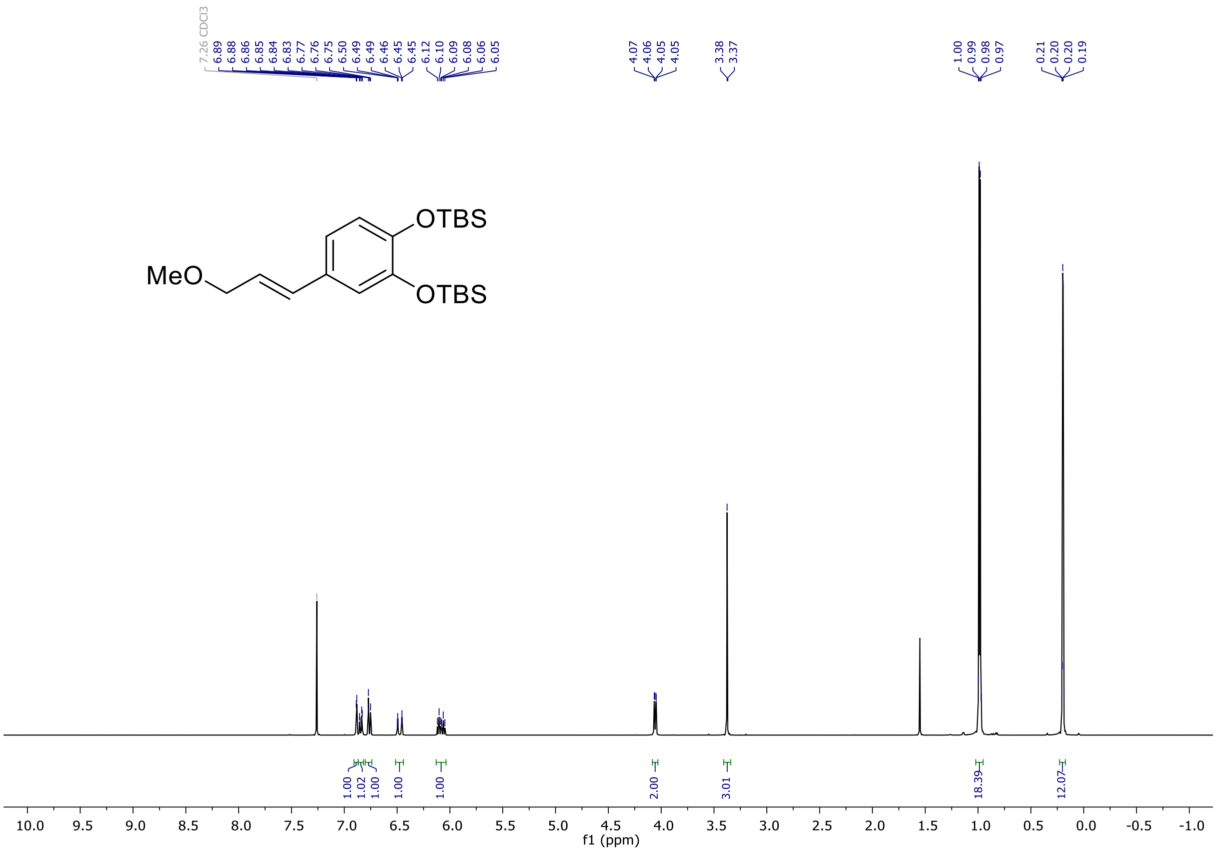

^{13}C NMR (101 MHz, $CDCl_3$) of styrene **SI-14:**

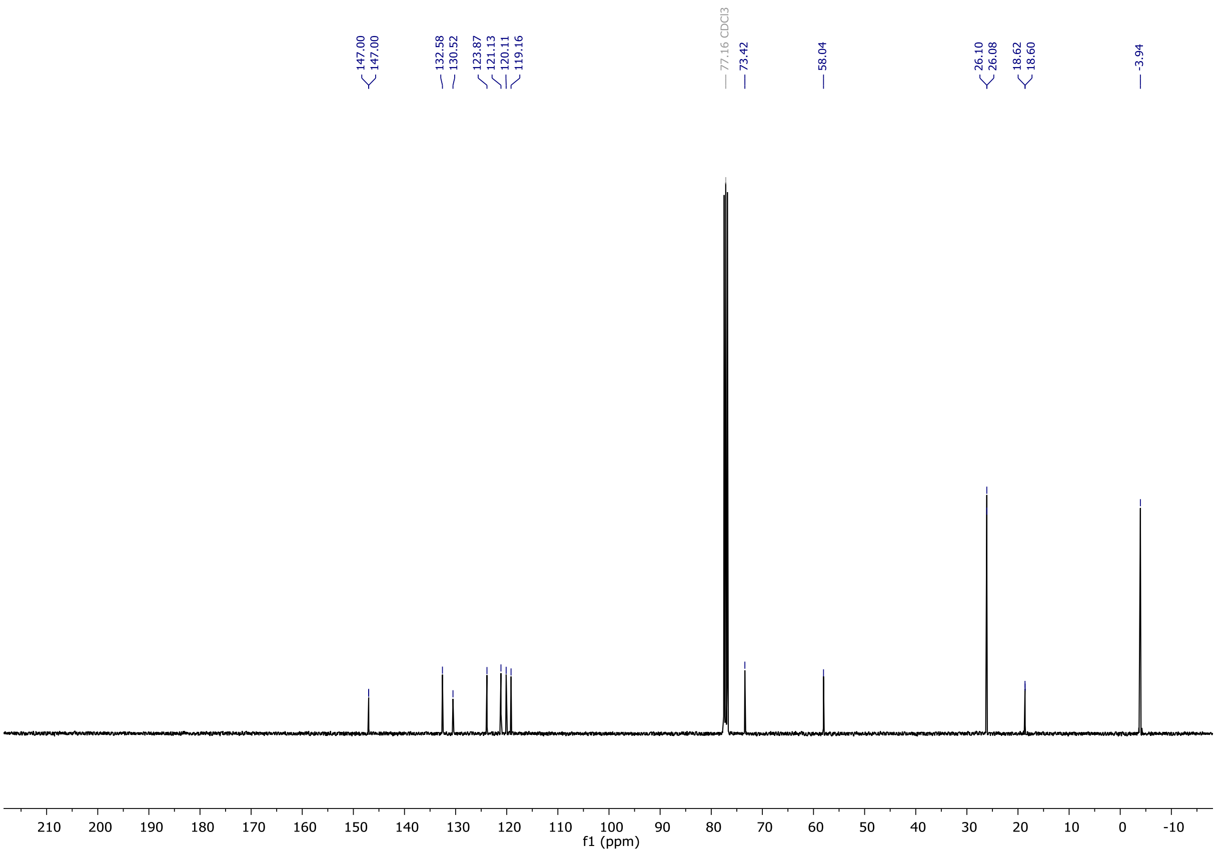

^{1}H NMR (400 MHz, $CDCl_3$) of catechol **13d**:

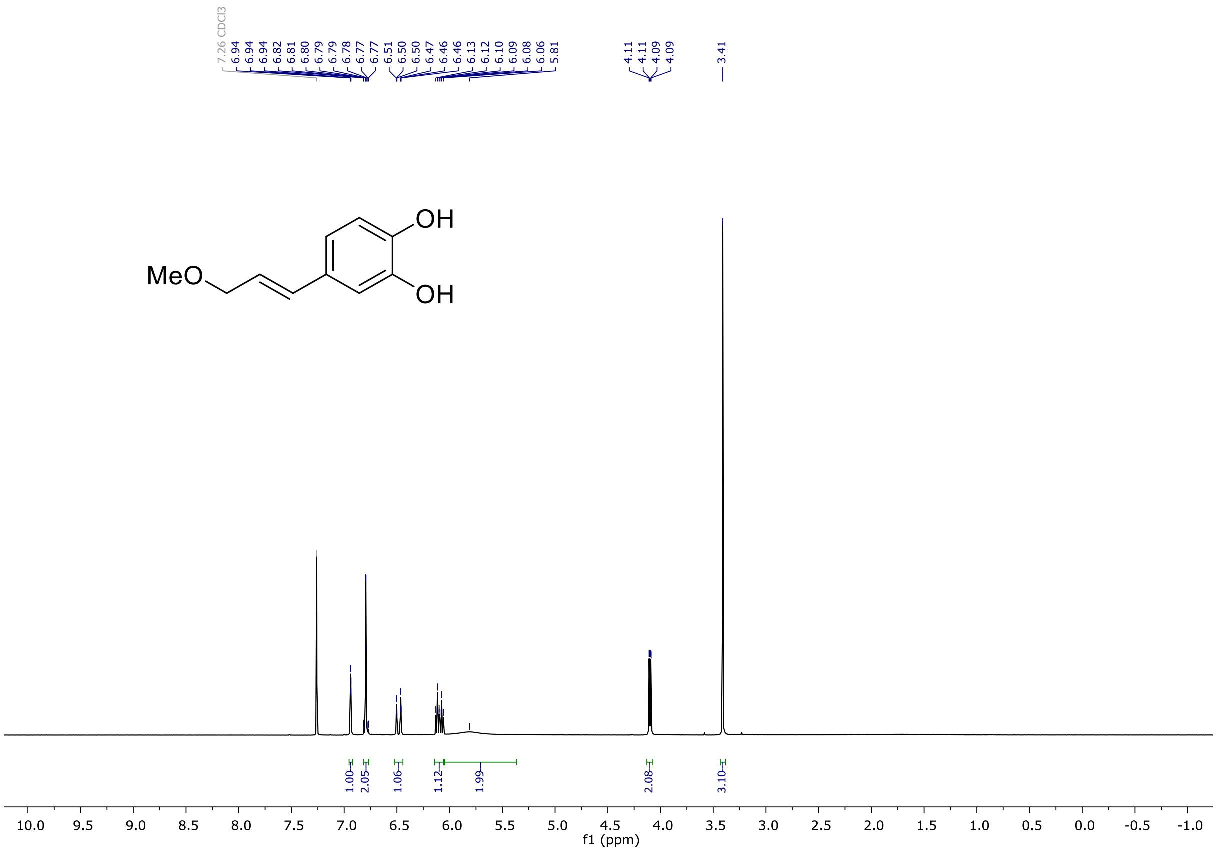

^{13}C NMR (101 MHz, $CDCl_3$) of catechol **13d:**

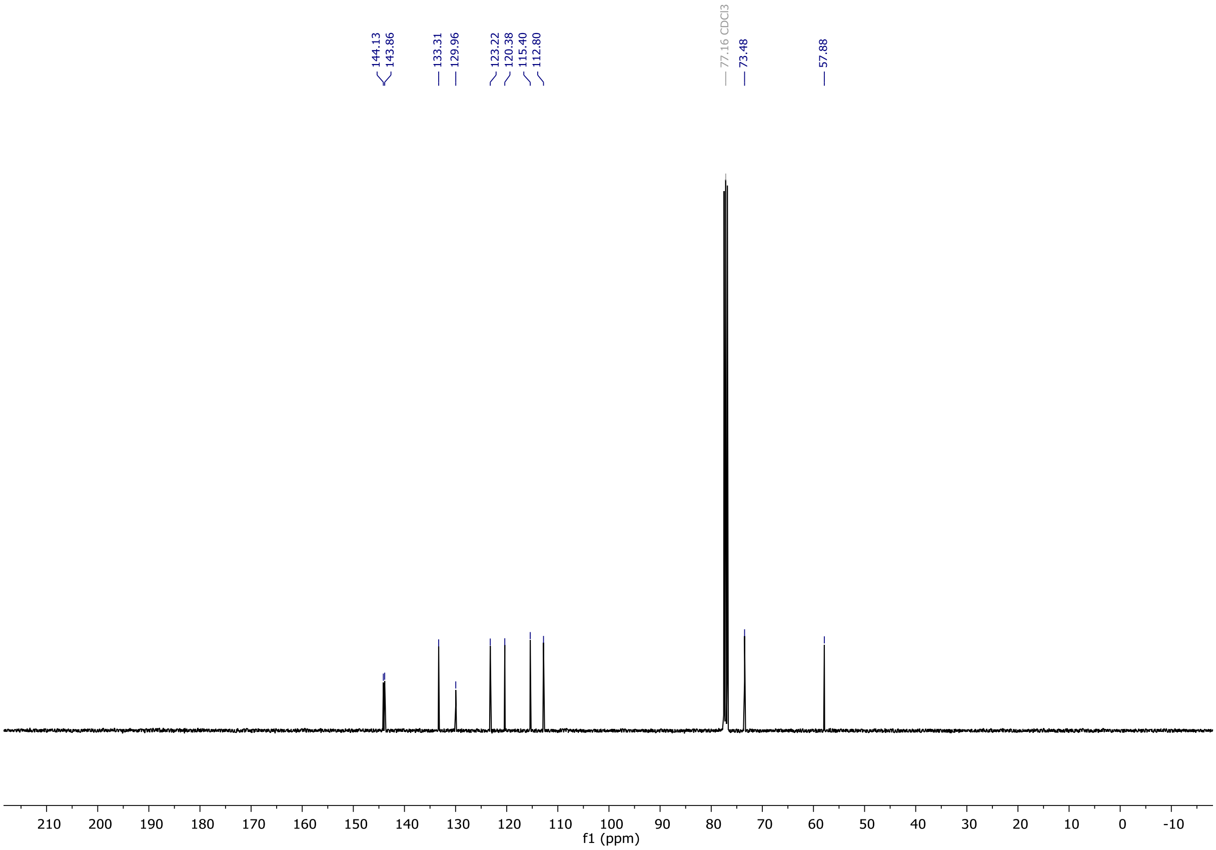

^{1}H NMR (400 MHz, $CDCl_3$) of styrene **SI-15**:

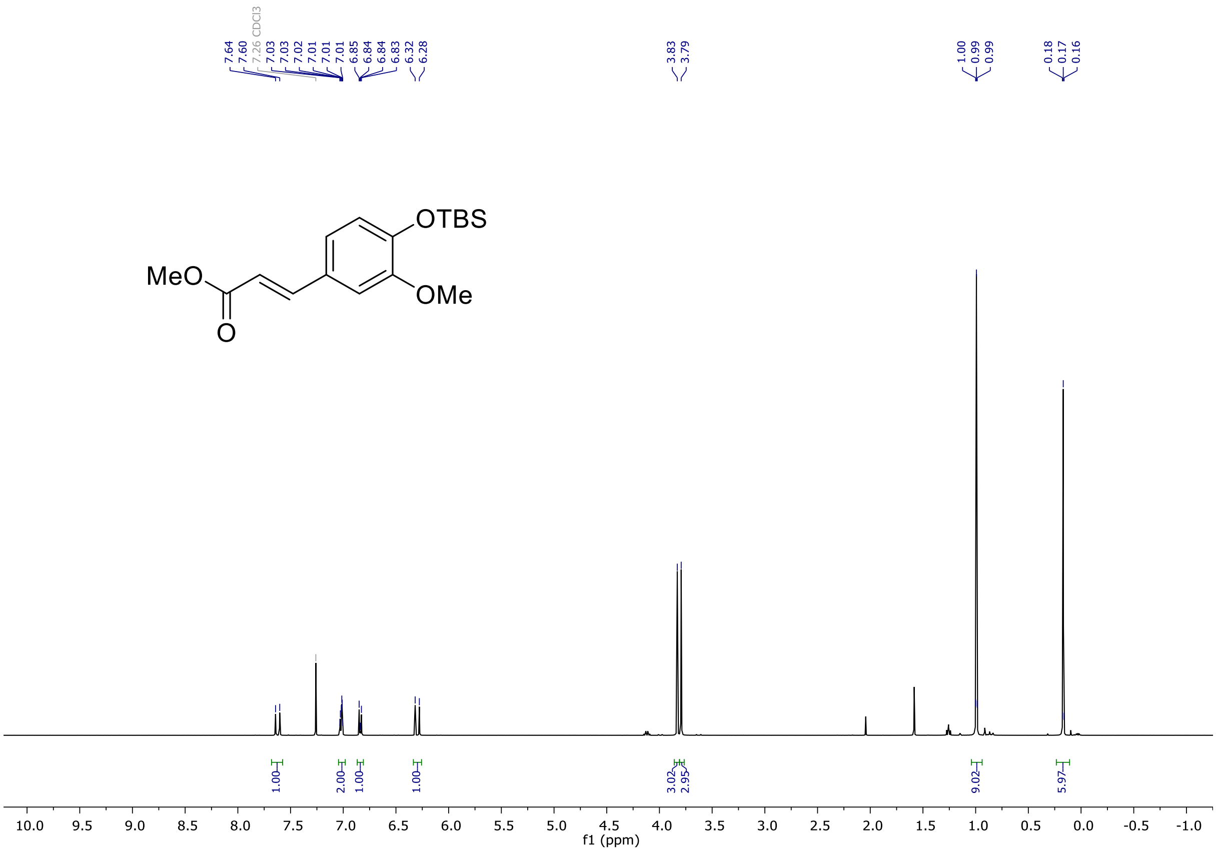

^{1}H NMR (400 MHz, $CDCl_3$) of styrene **SI-17**:

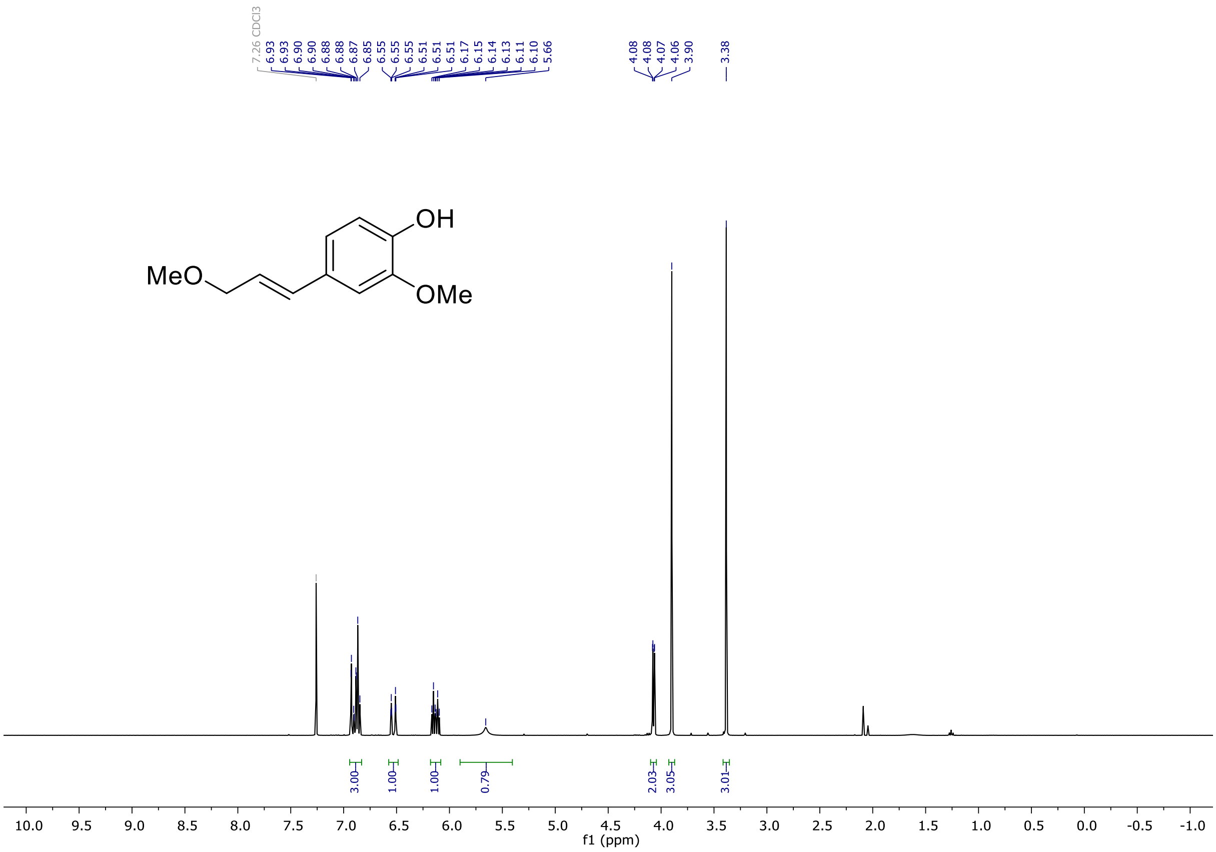

^{13}C NMR (101 MHz, $CDCl_3$) of styrene **SI-17:**

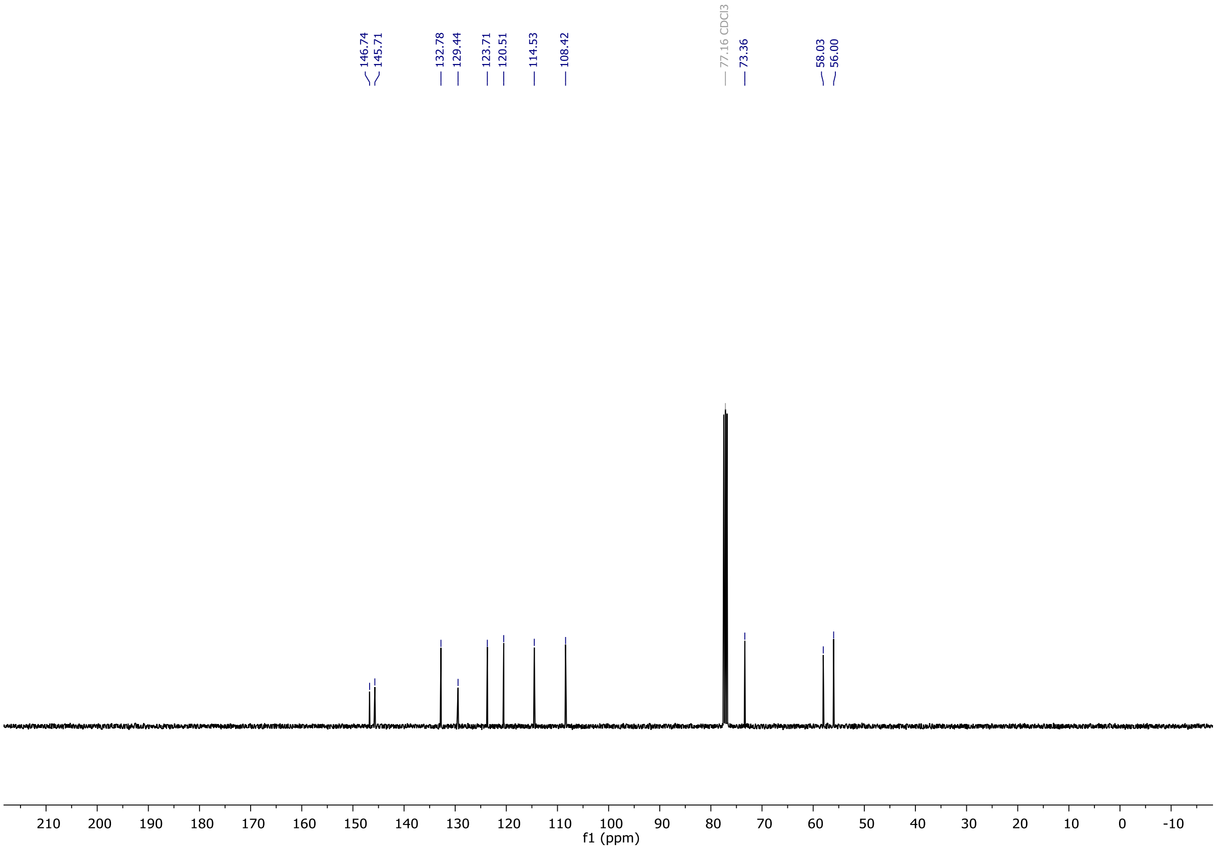

^{1}H NMR (400 MHz, $CDCl_3$) of benzodioxole **26c**:

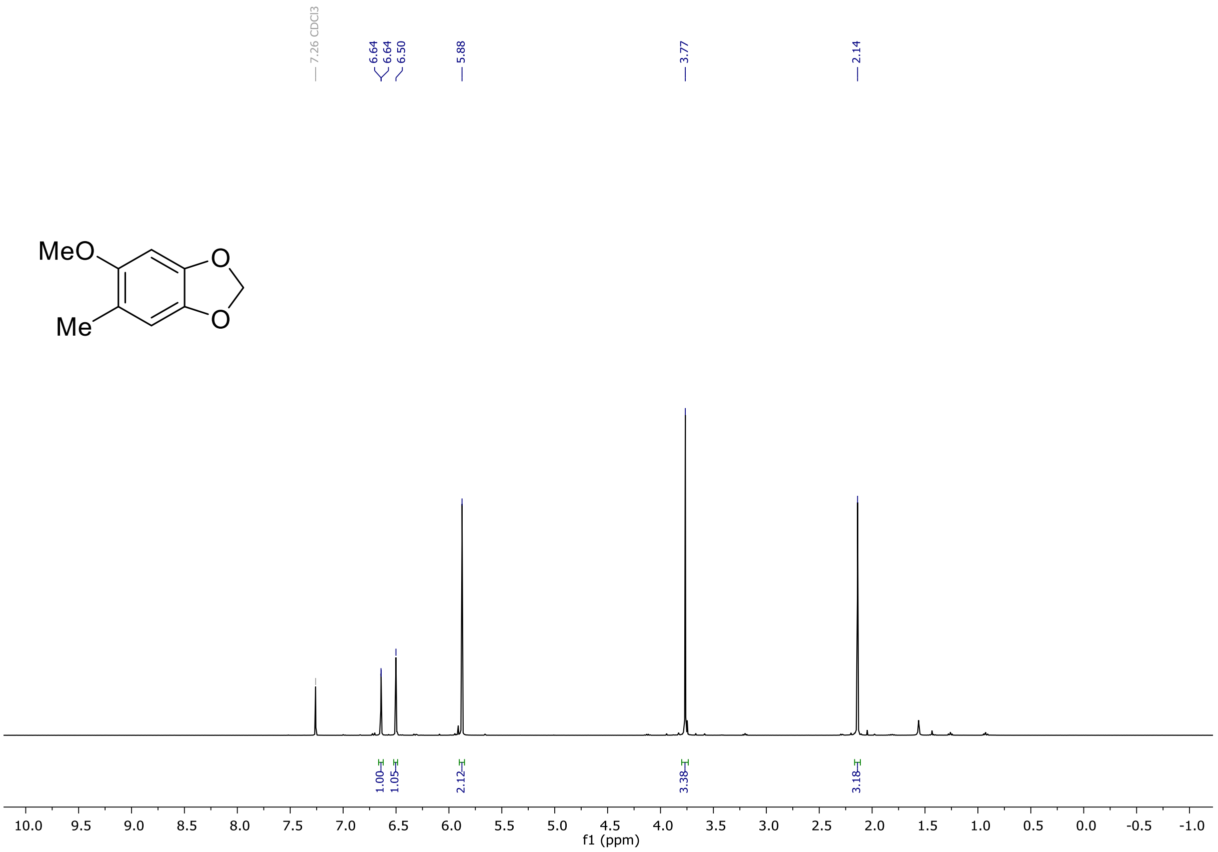

^{1}H NMR (400 MHz, $CDCl_3$) of quinone **25c**:

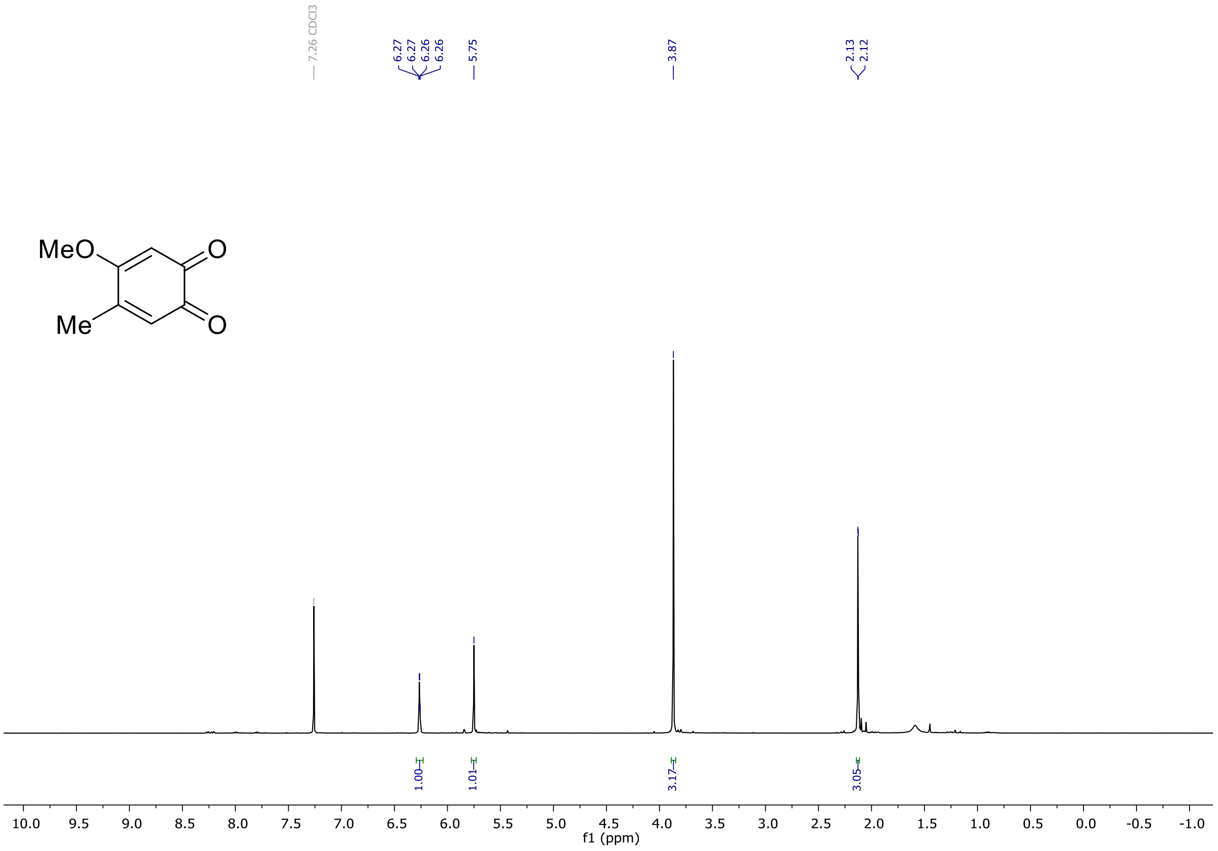

^{1}H NMR (400 MHz, $CDCl_3$) of benzodioxole **26d**:

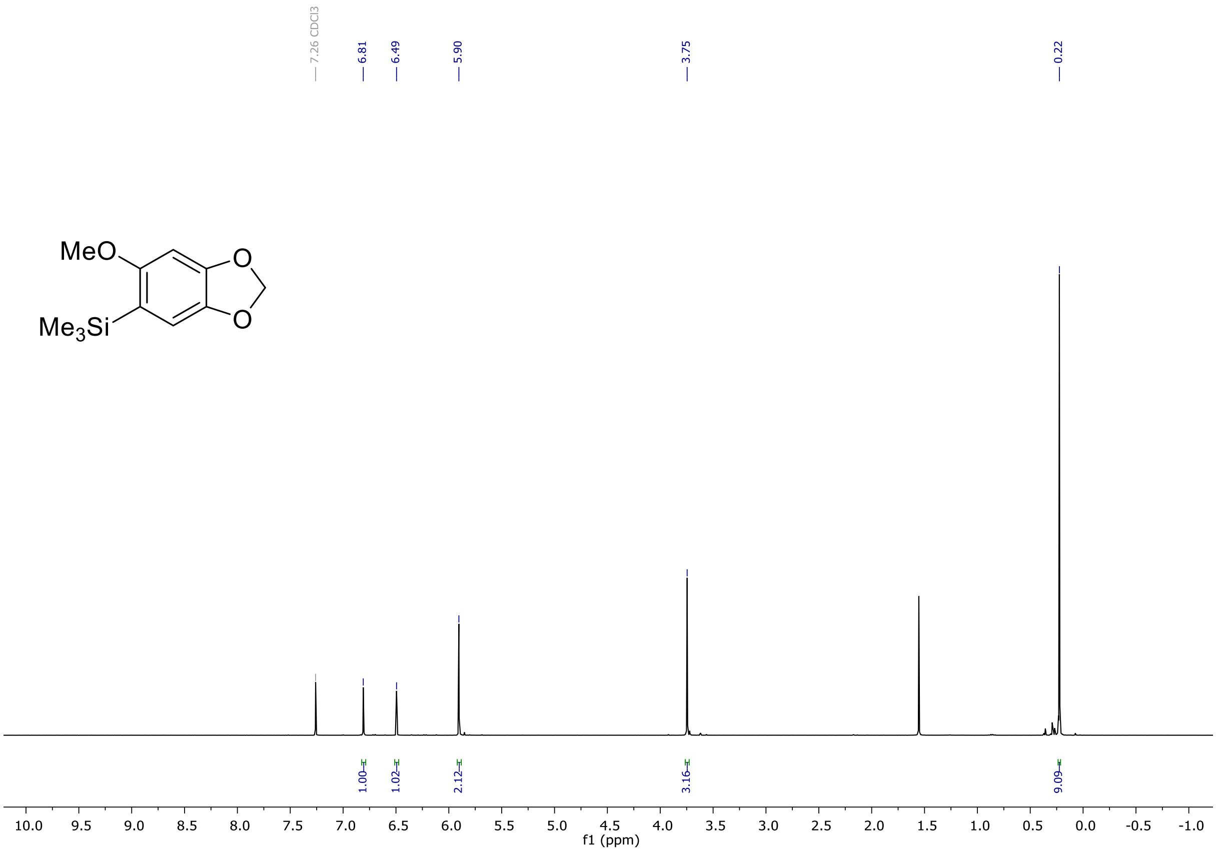

^{13}C NMR (101 MHz, $CDCl_3$) of benzodioxole **26d:**

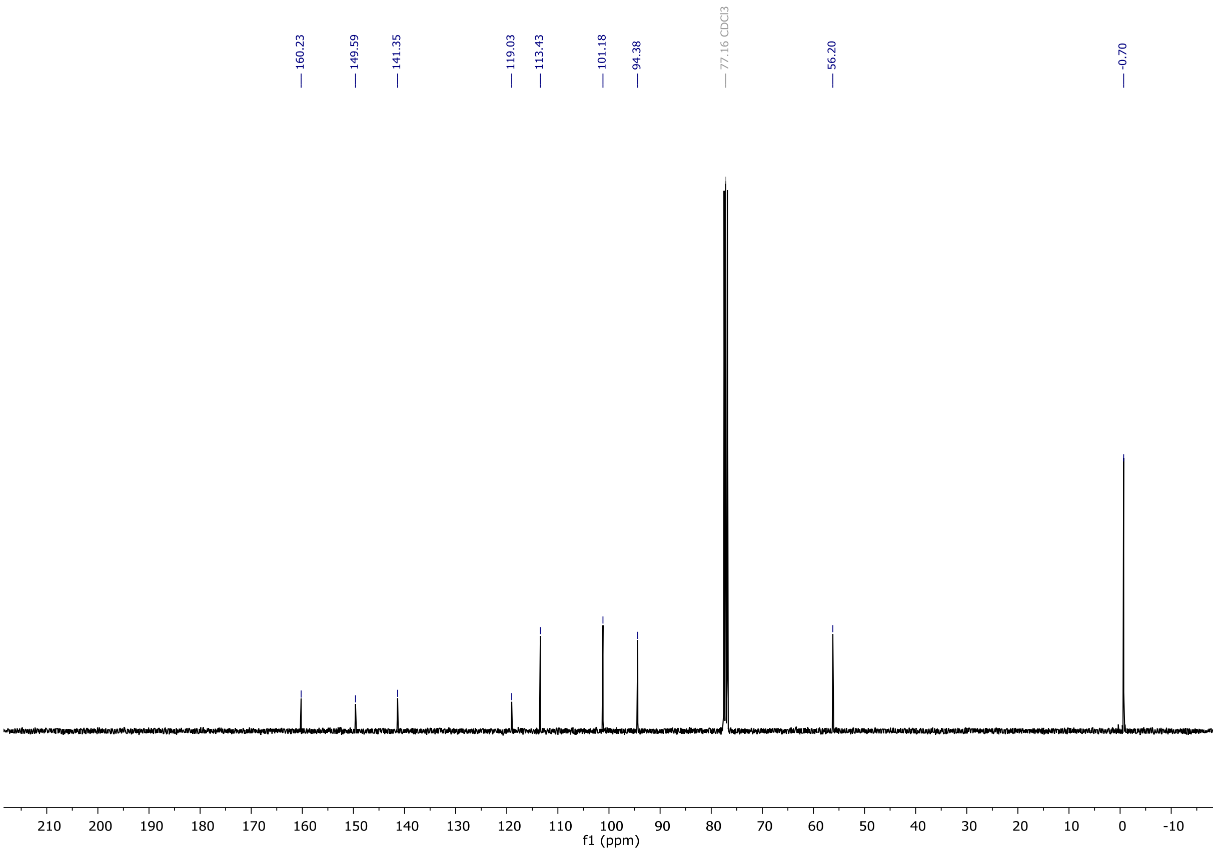

^{1}H NMR (400 MHz, $CDCl_3$) of *ortho*-quinone **25d**:

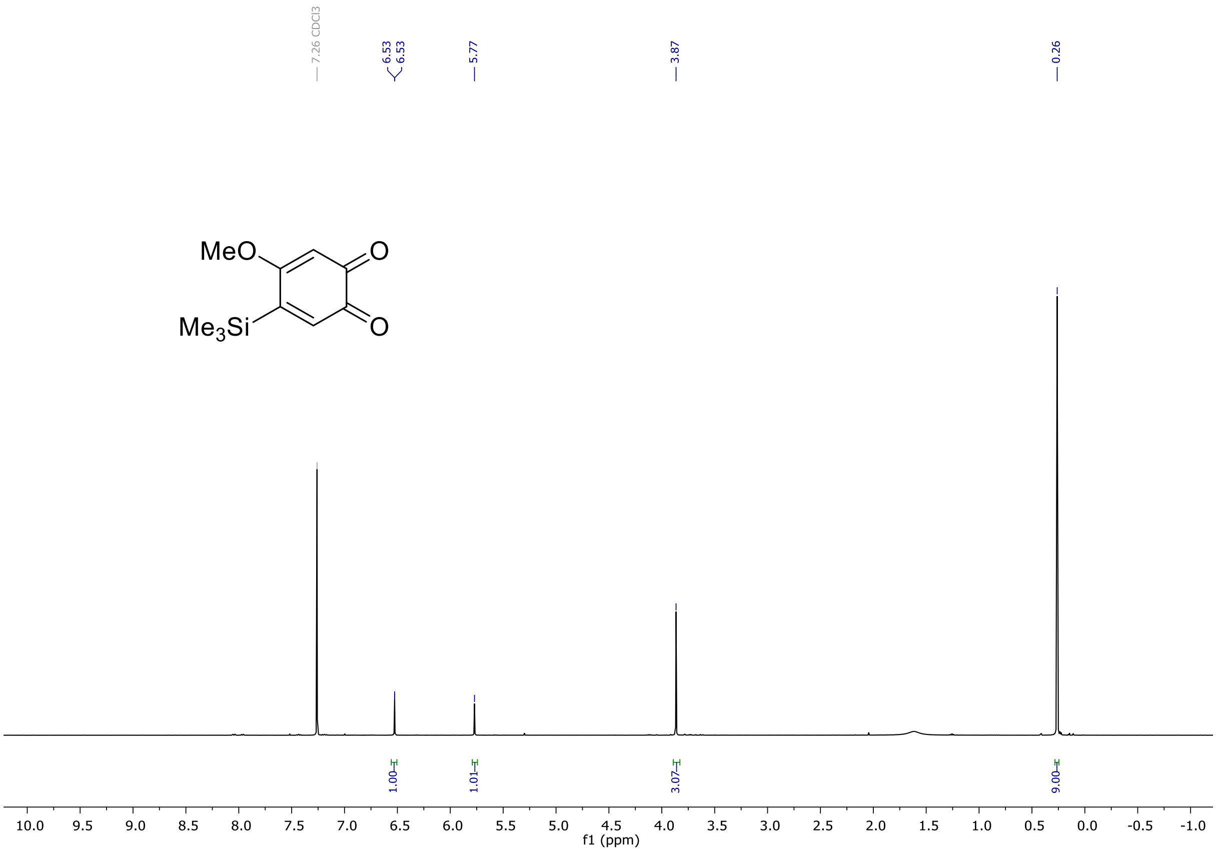

^{13}C NMR (101 MHz, $CDCl_3$) of *ortho*-quinone **25d:**

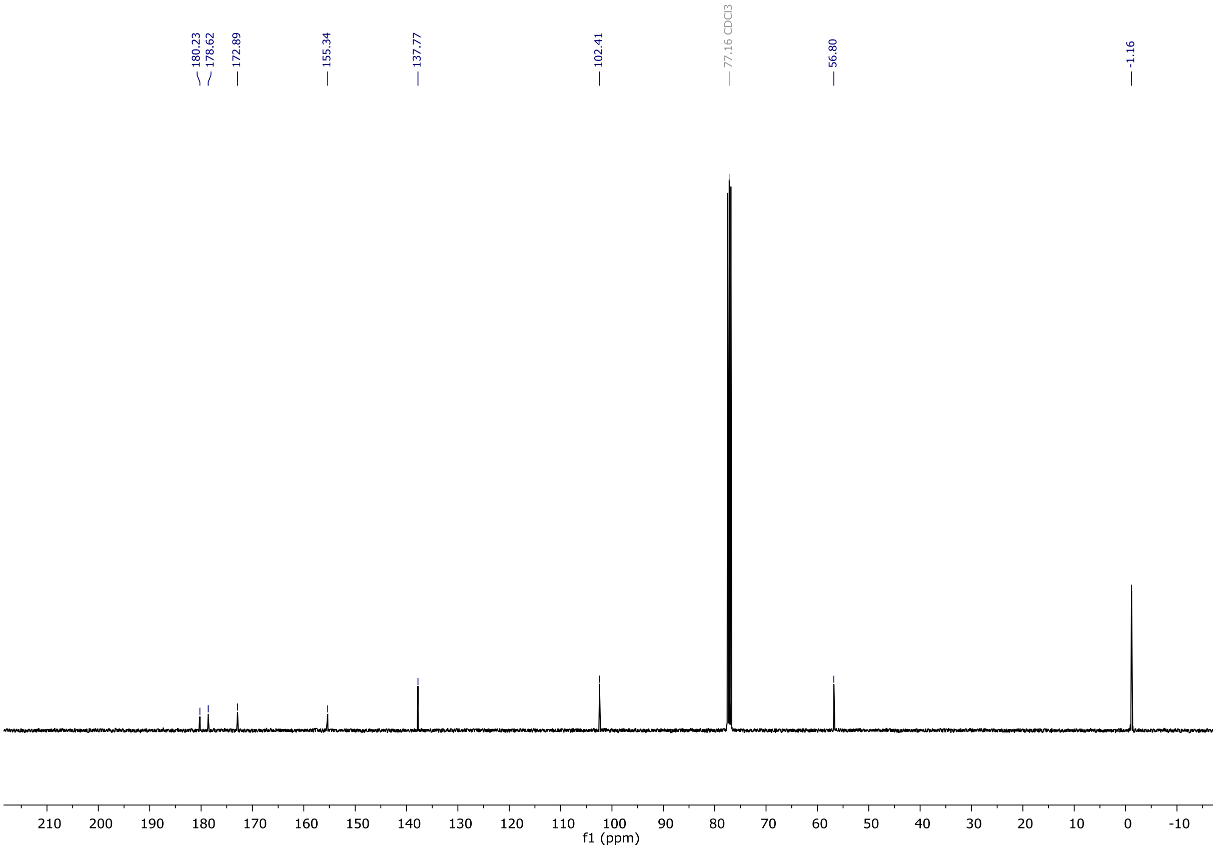

^1H NMR (400 MHz, $CDCl_3$) of benzodioxole **26e**:

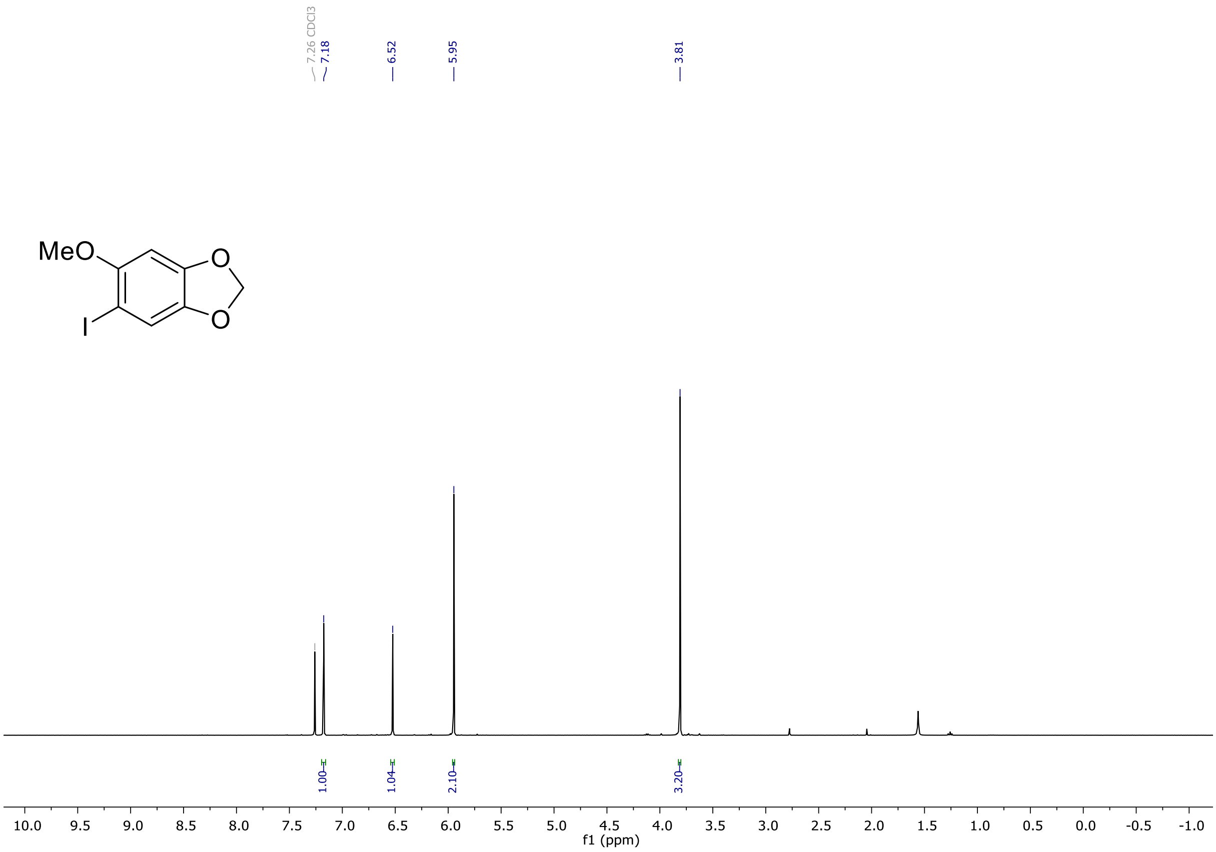

^1H NMR (400 MHz, $CDCl_3$) of benzodioxole **26f**:

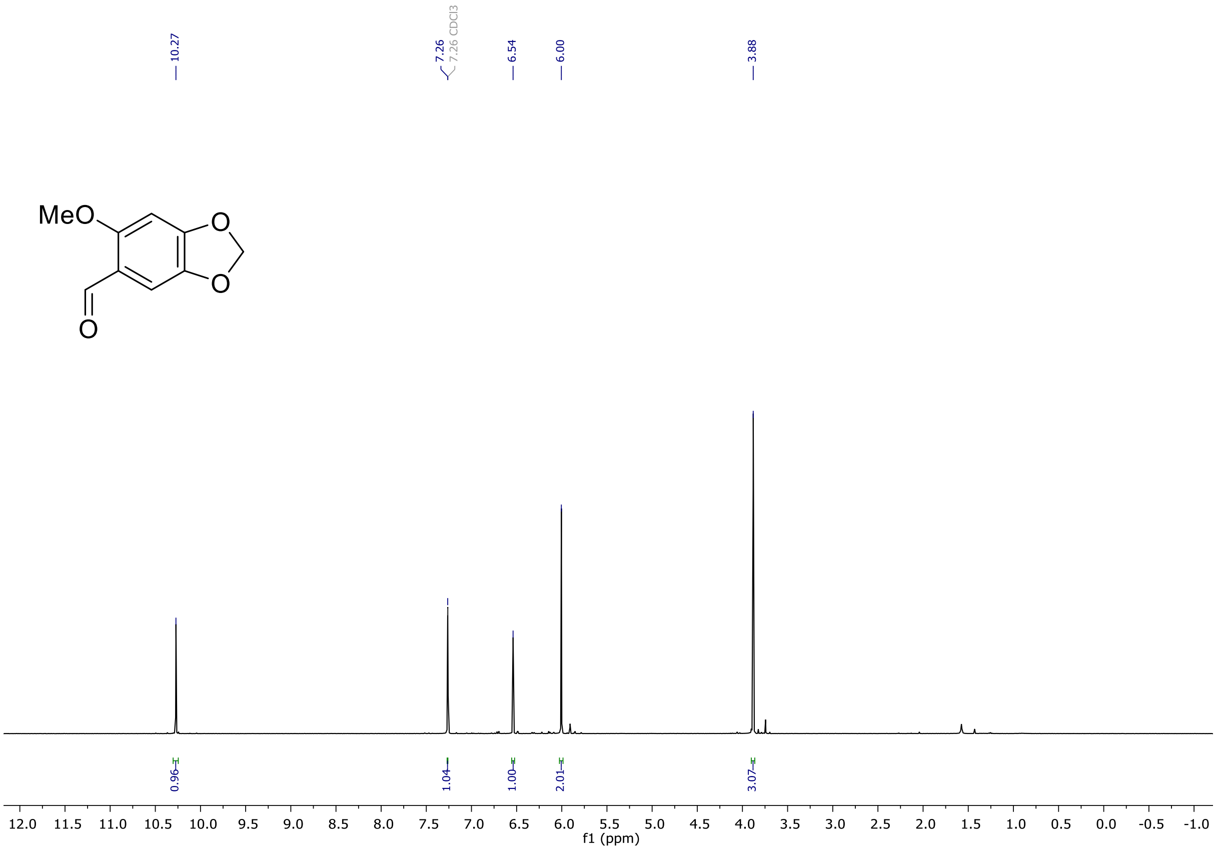

^{1}H NMR (400 MHz, $CDCl_3$) of phenol **SI-18**:

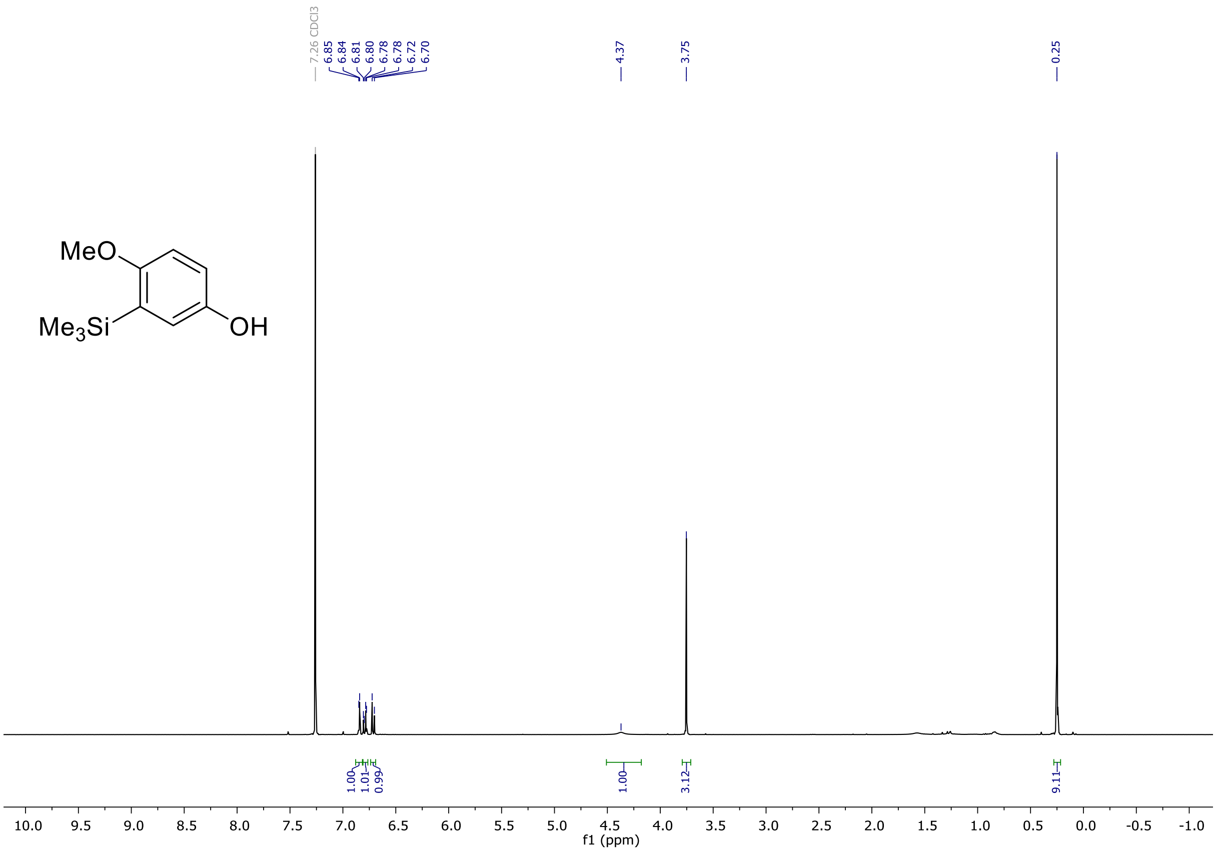

^{13}C NMR (101 MHz, $CDCl_3$) of phenol **SI-18:**

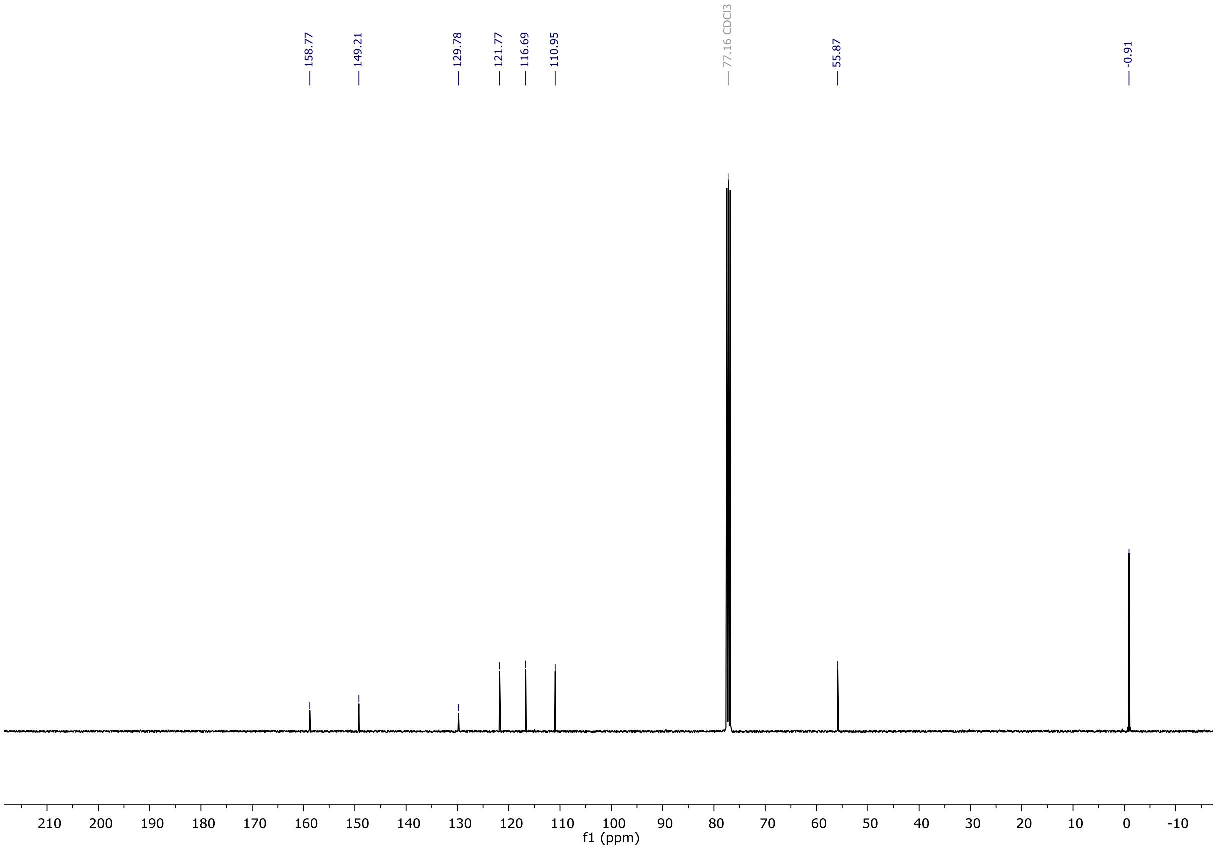

^{1}H NMR (400 MHz, CD_3OD) of catechol **SI-20**:

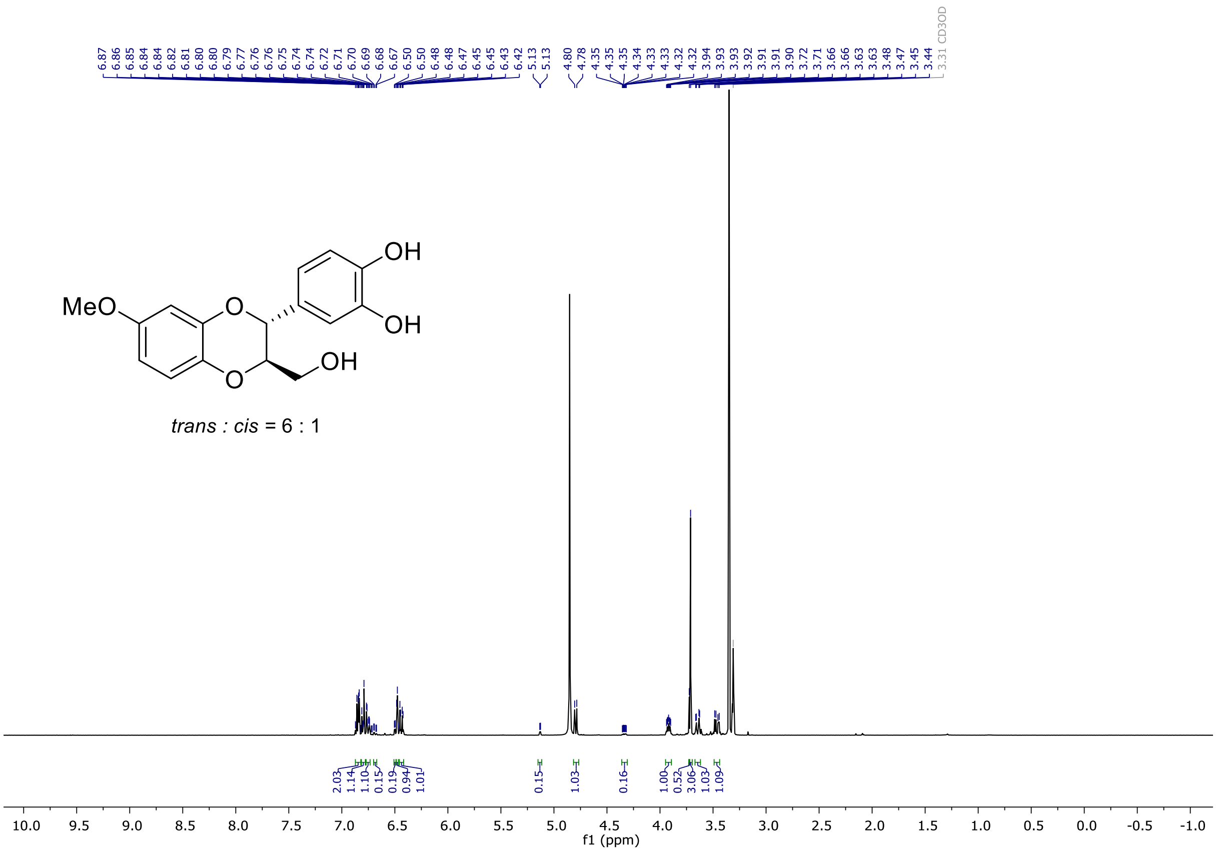

trans : *cis* = 6 : 1

^{13}C NMR (101 MHz, CD_3OD) of catechol **SI-20**:

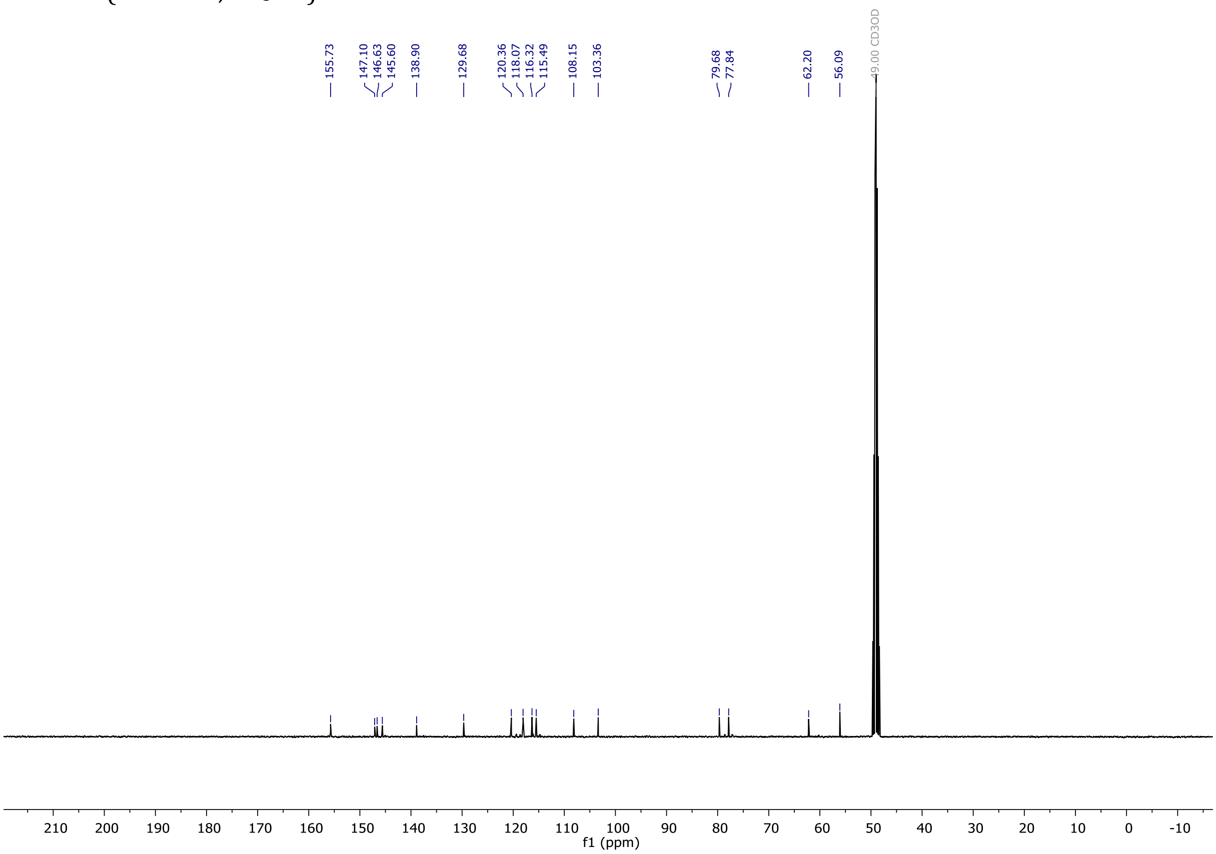

^{1}H NMR (400 MHz, CD_3OD) of catechol **48c**:

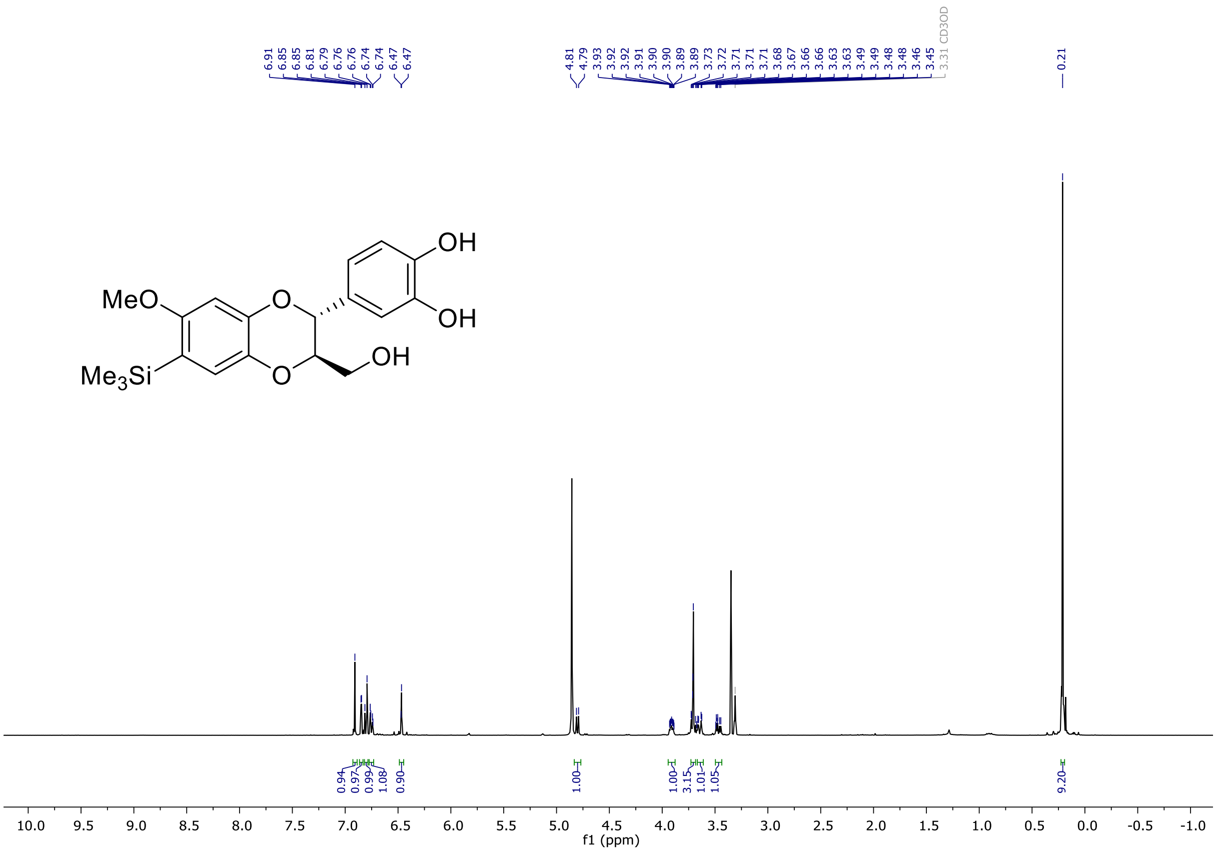

^{13}C NMR (101 MHz, CD_3OD) of catechol **48c:**

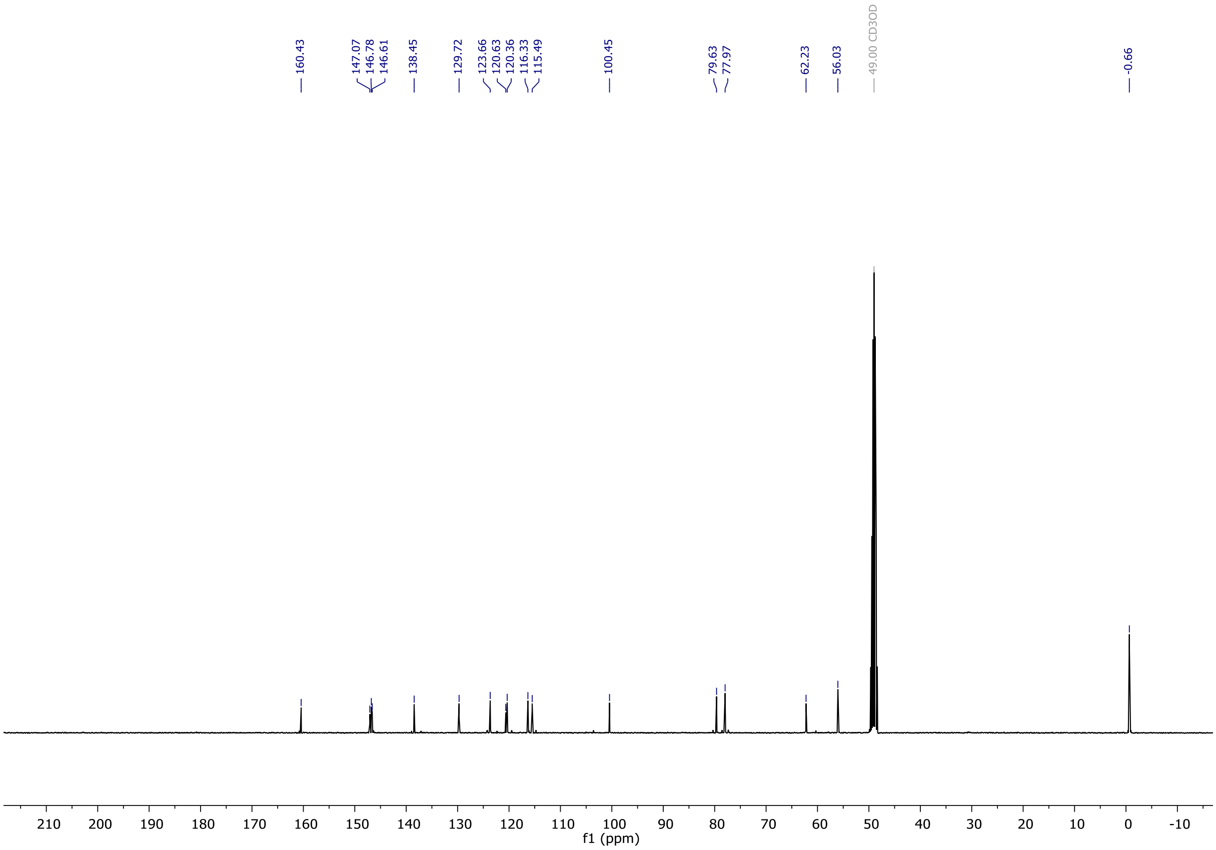

^{1}H NMR (400 MHz, $CDCl_3$) of catechol **48d**:

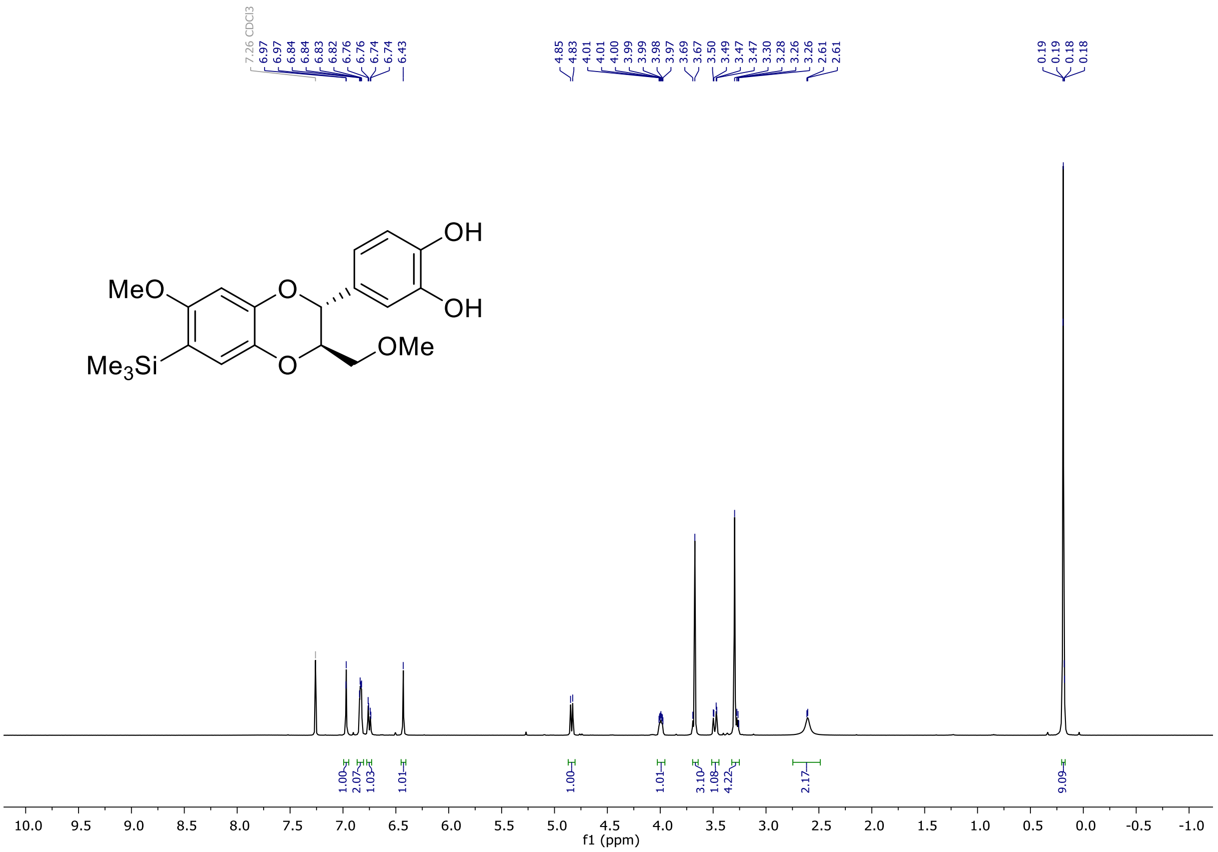

^{1}H NMR (400 MHz, CD_3CN) of catechol **48d**:

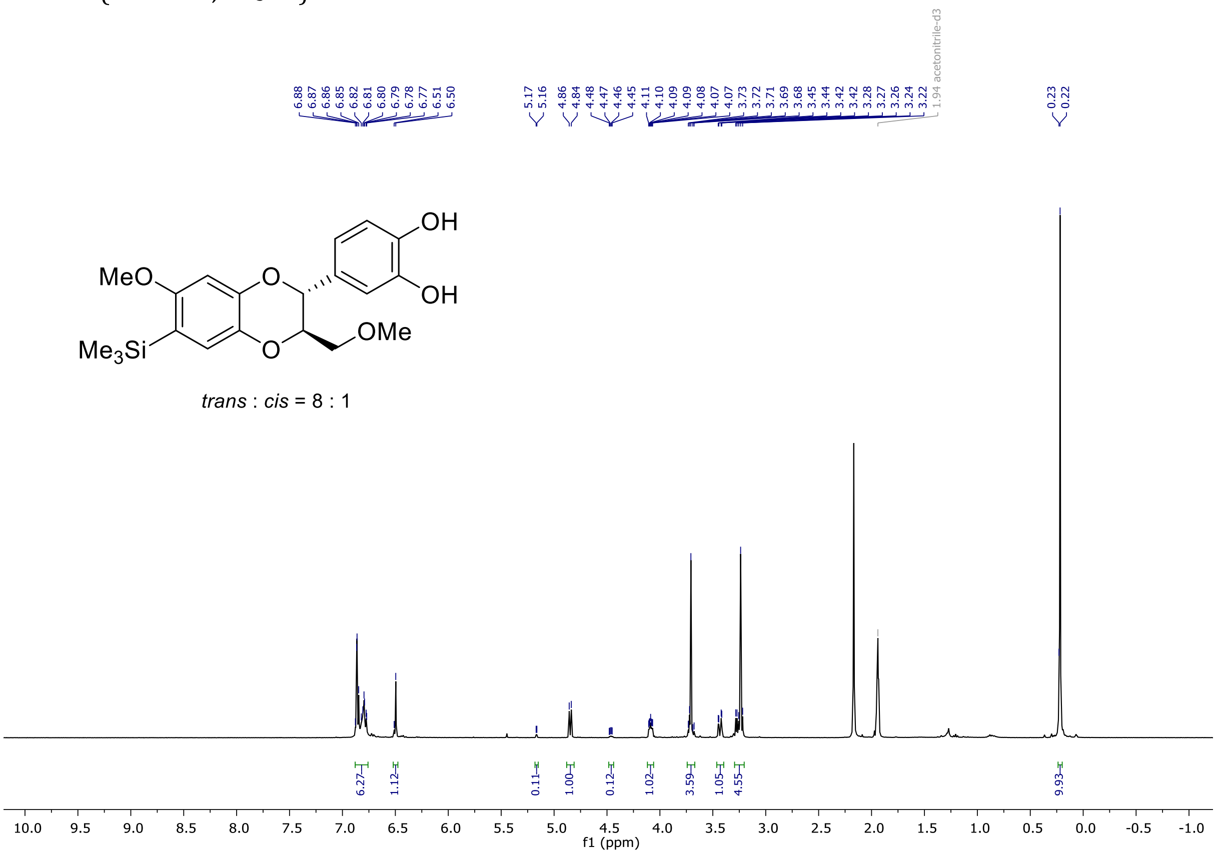

^{13}C NMR (101 MHz, CD_3CN) of catechol **48d**:

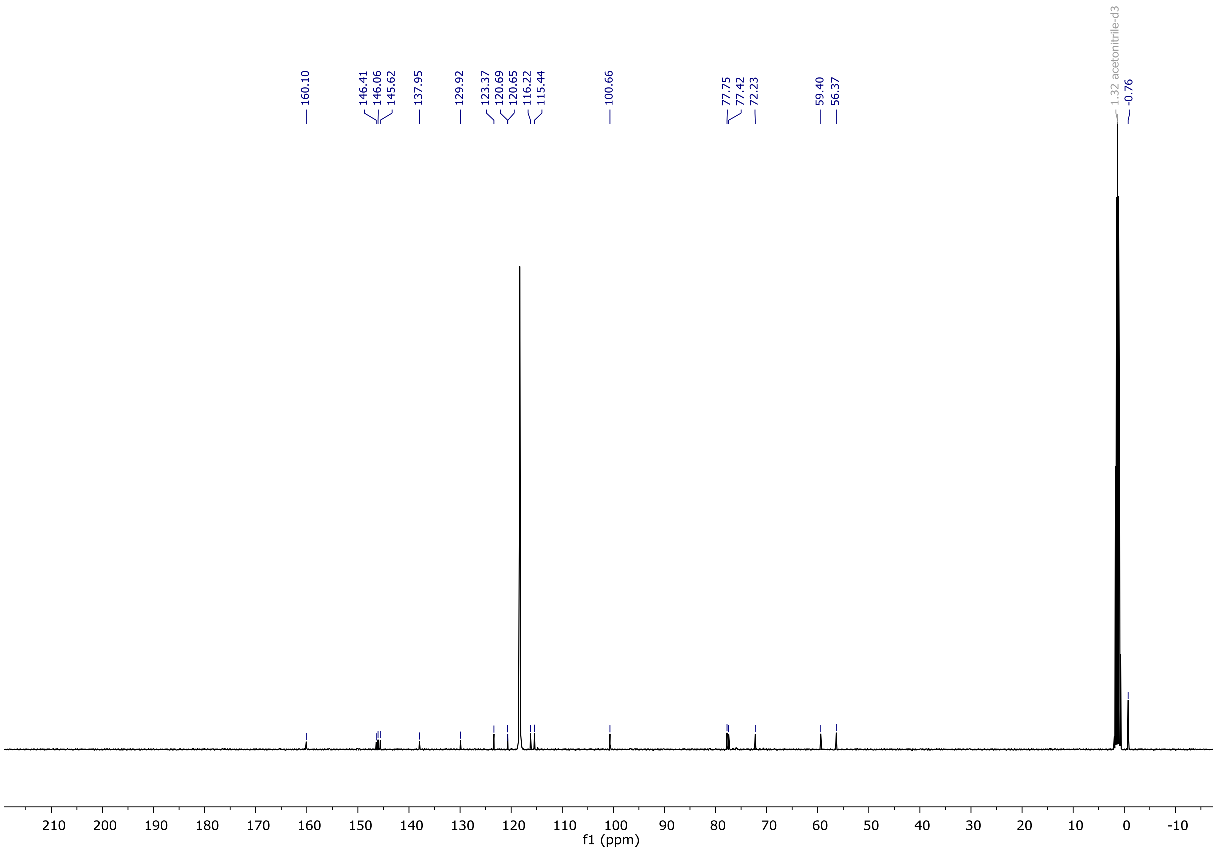

^{1}H NMR (500 MHz, CD_3OD) of catechol **52**:

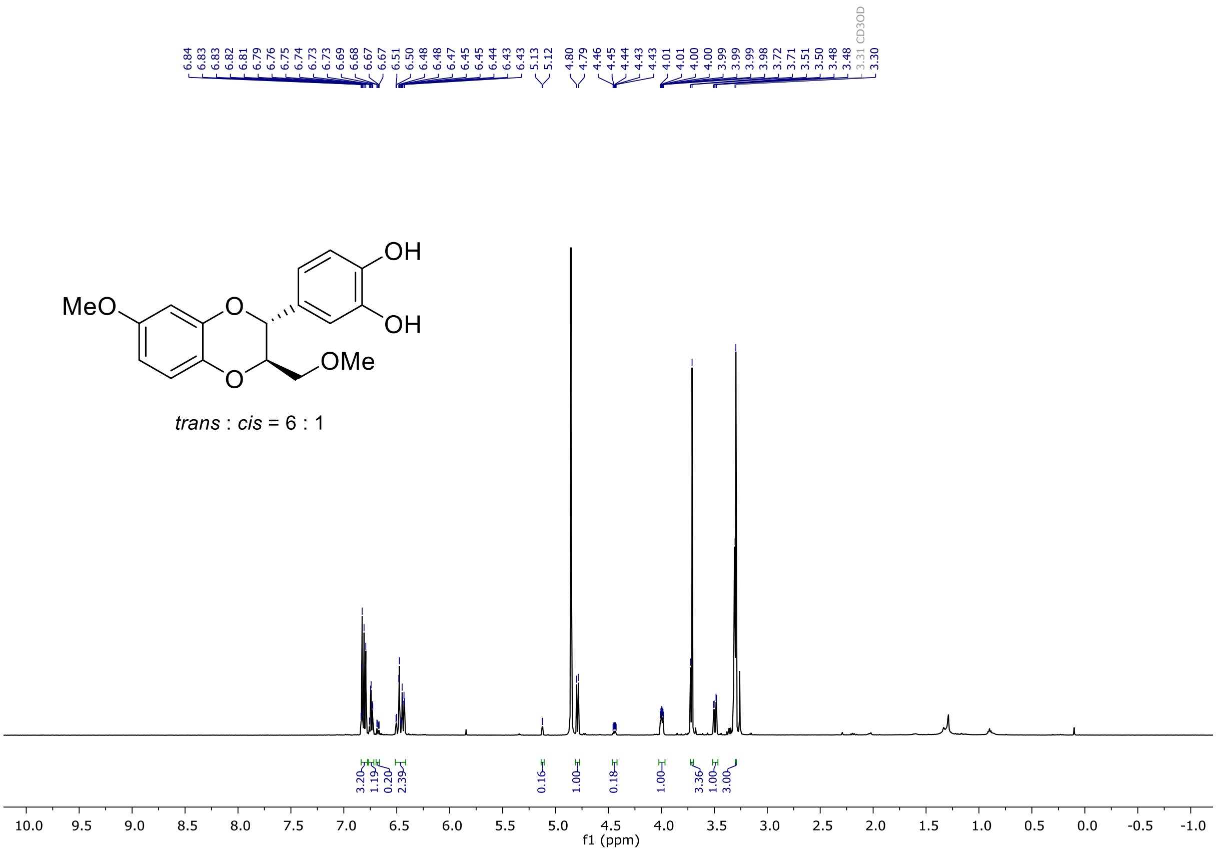

^{13}C NMR (101 MHz, CD_3OD) of catechol **52**:

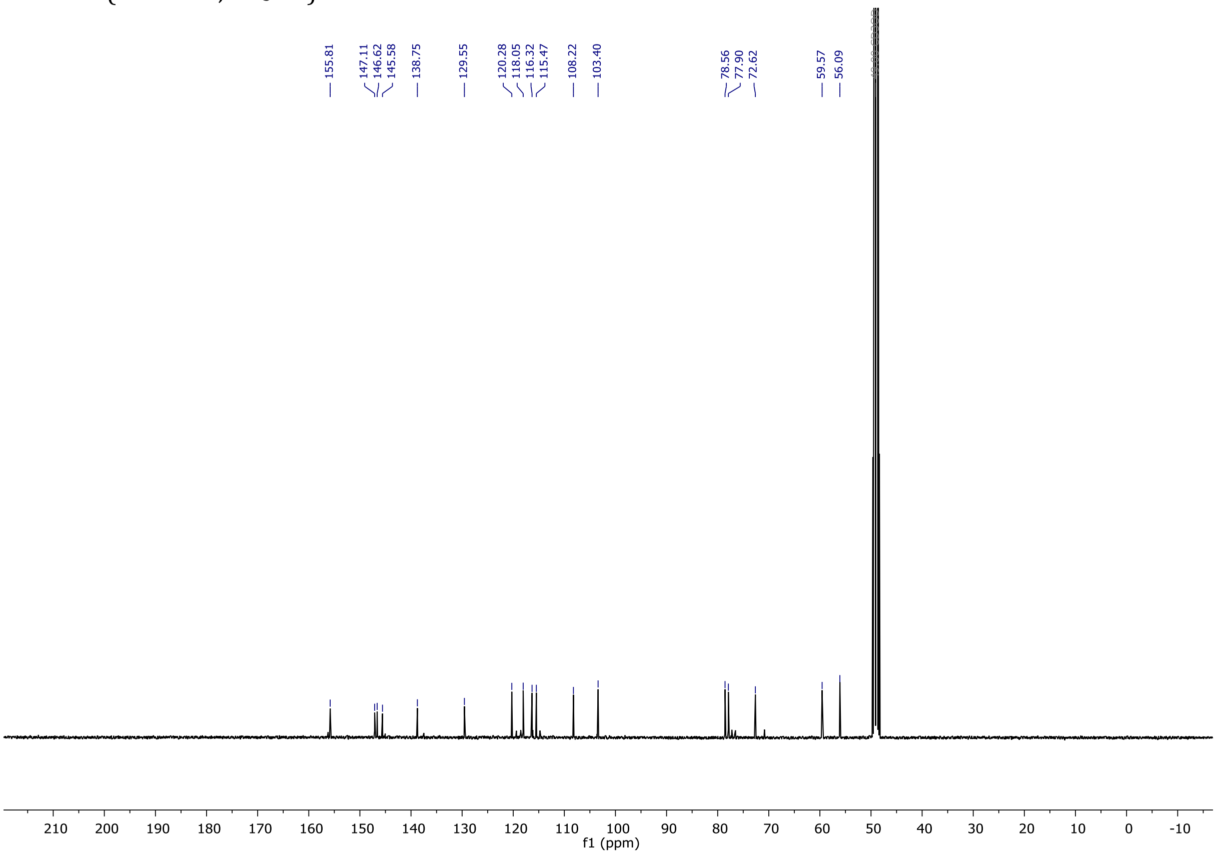

^{1}H NMR (400 MHz, $CDCl_3$) of benzodioxane **SI-21**:

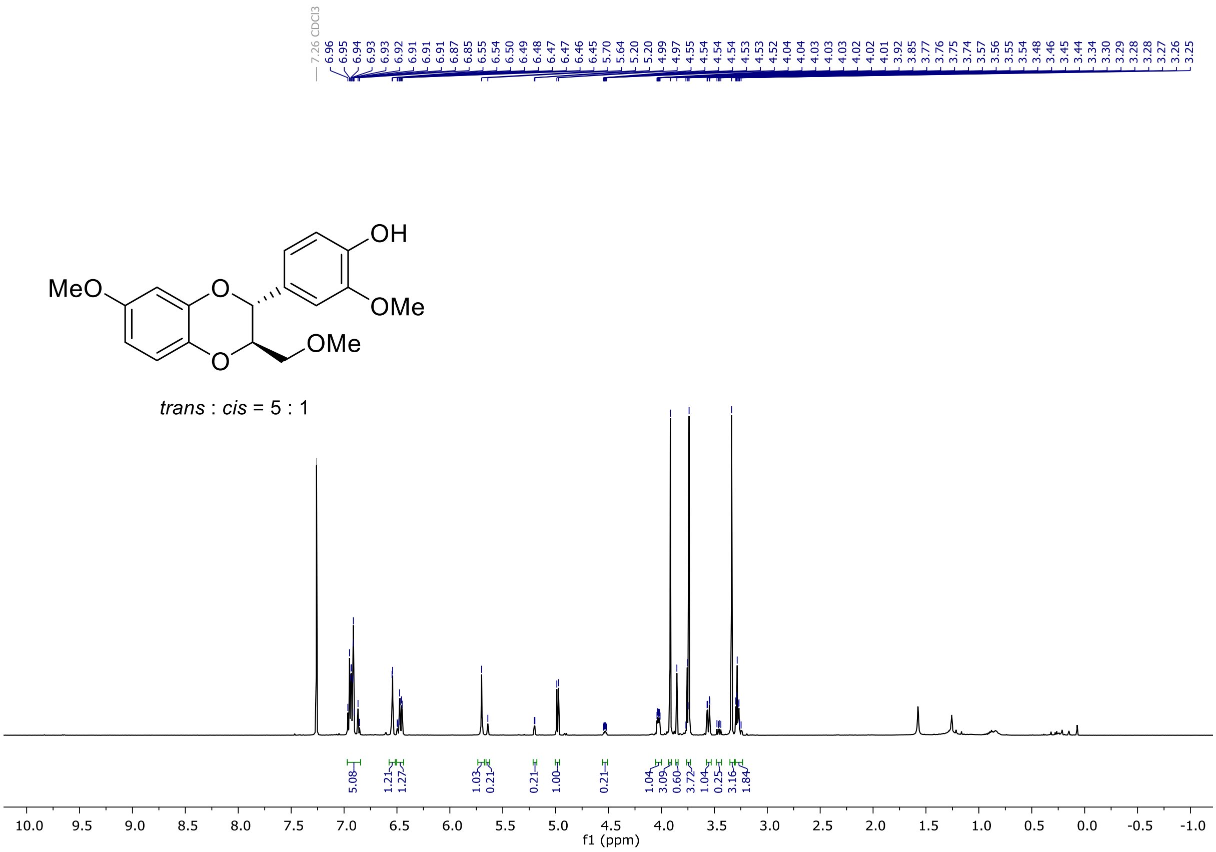

^{13}C NMR (101 MHz, $CDCl_3$) of benzodioxane **SI-21**:

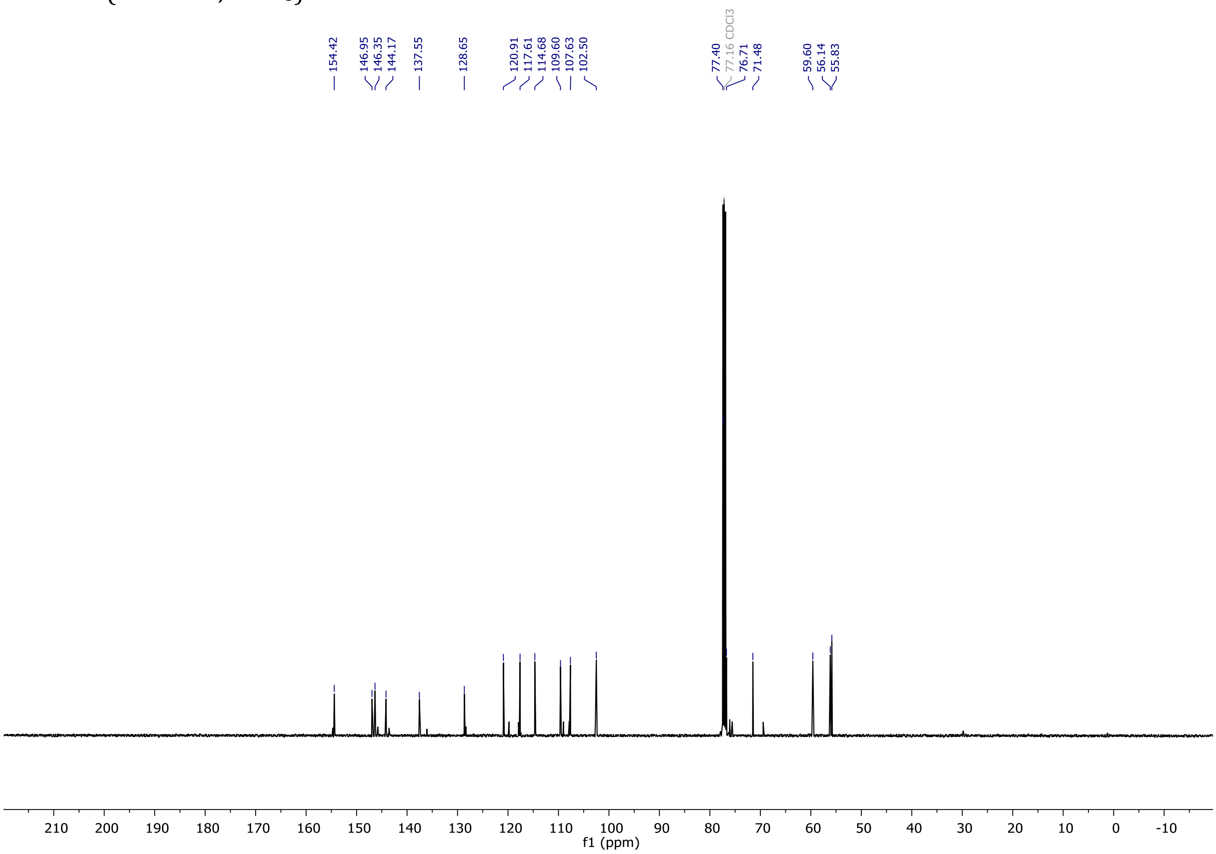

^{1}H NMR (500 MHz, acetonitrile-d_3) of diol **50**:

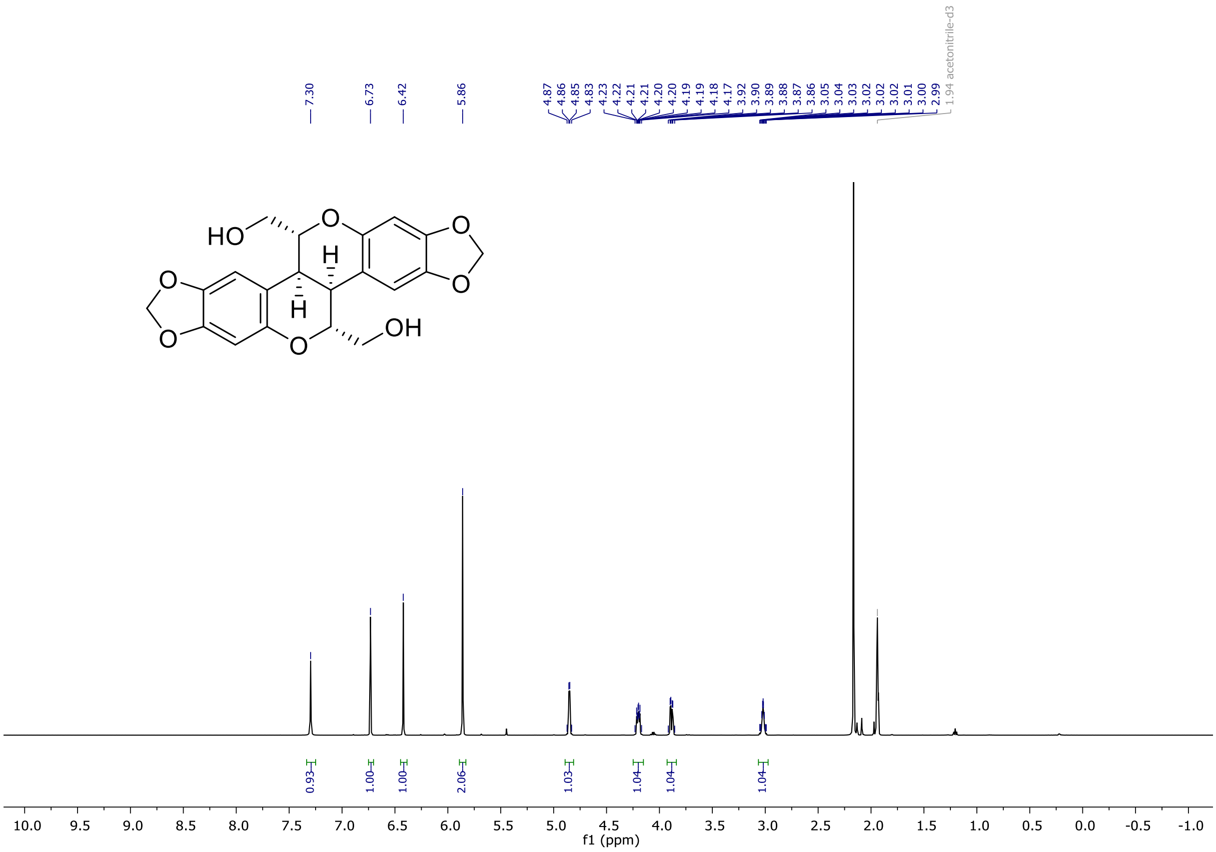

^{13}C NMR (126 MHz, acetonitrile-d_3) of diol **50:**

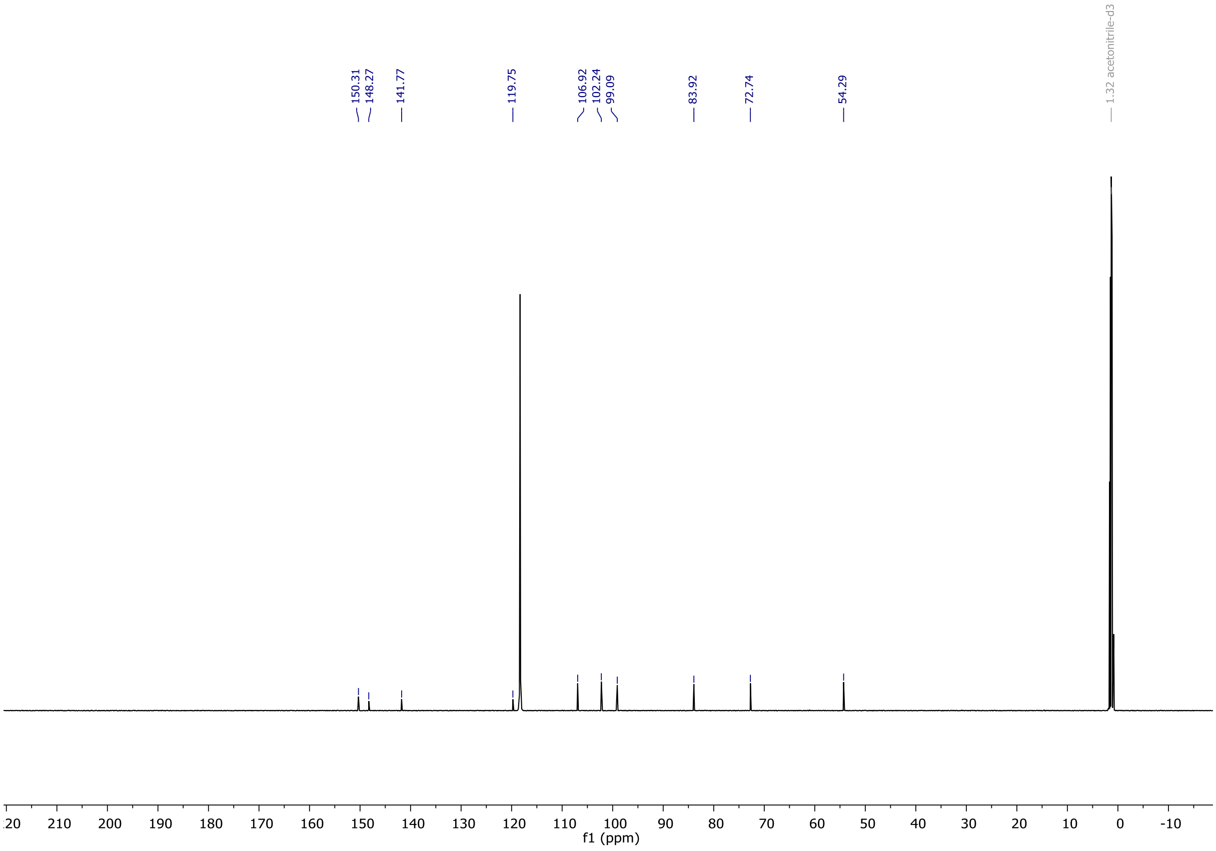

COSY NMR (500 MHz, acetonitrile-d_3) of diol **50**:

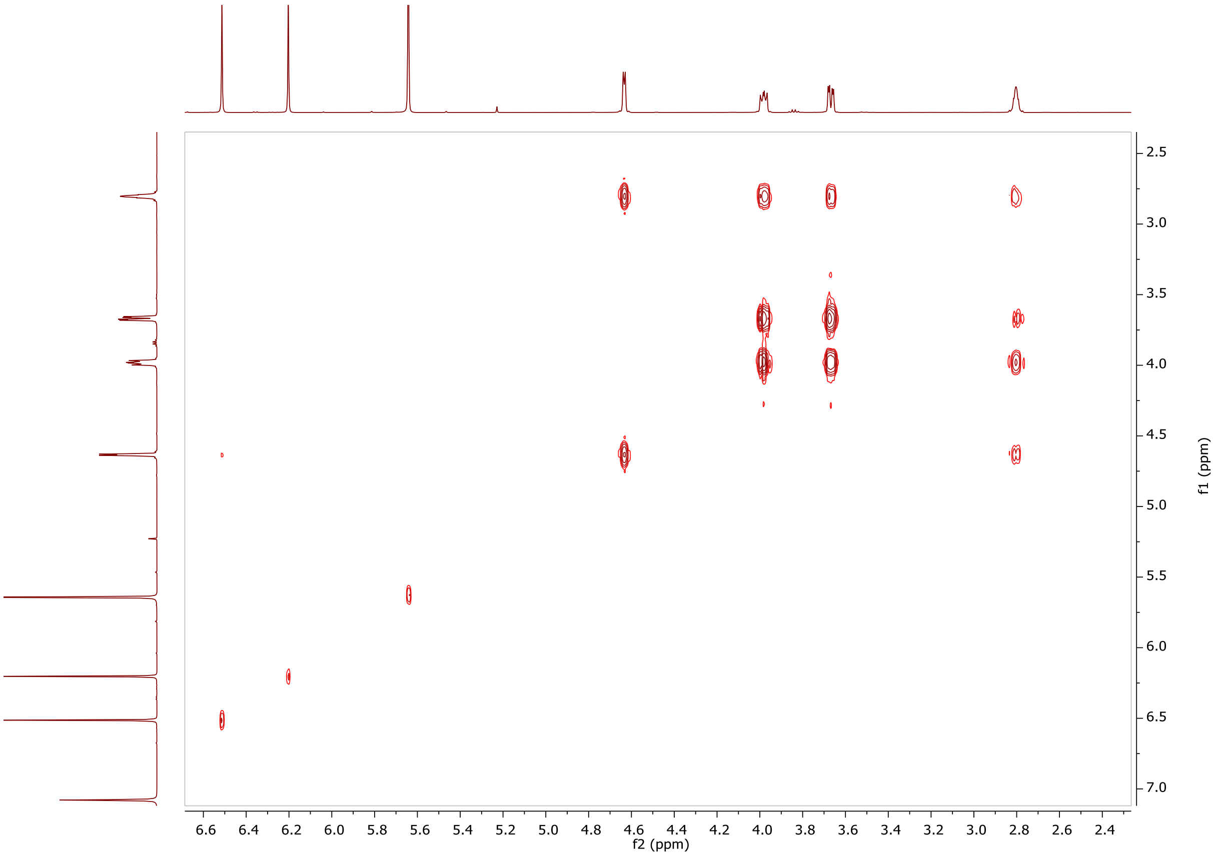

HSQC NMR (500 MHz, acetonitrile-d_3) of diol **50**:

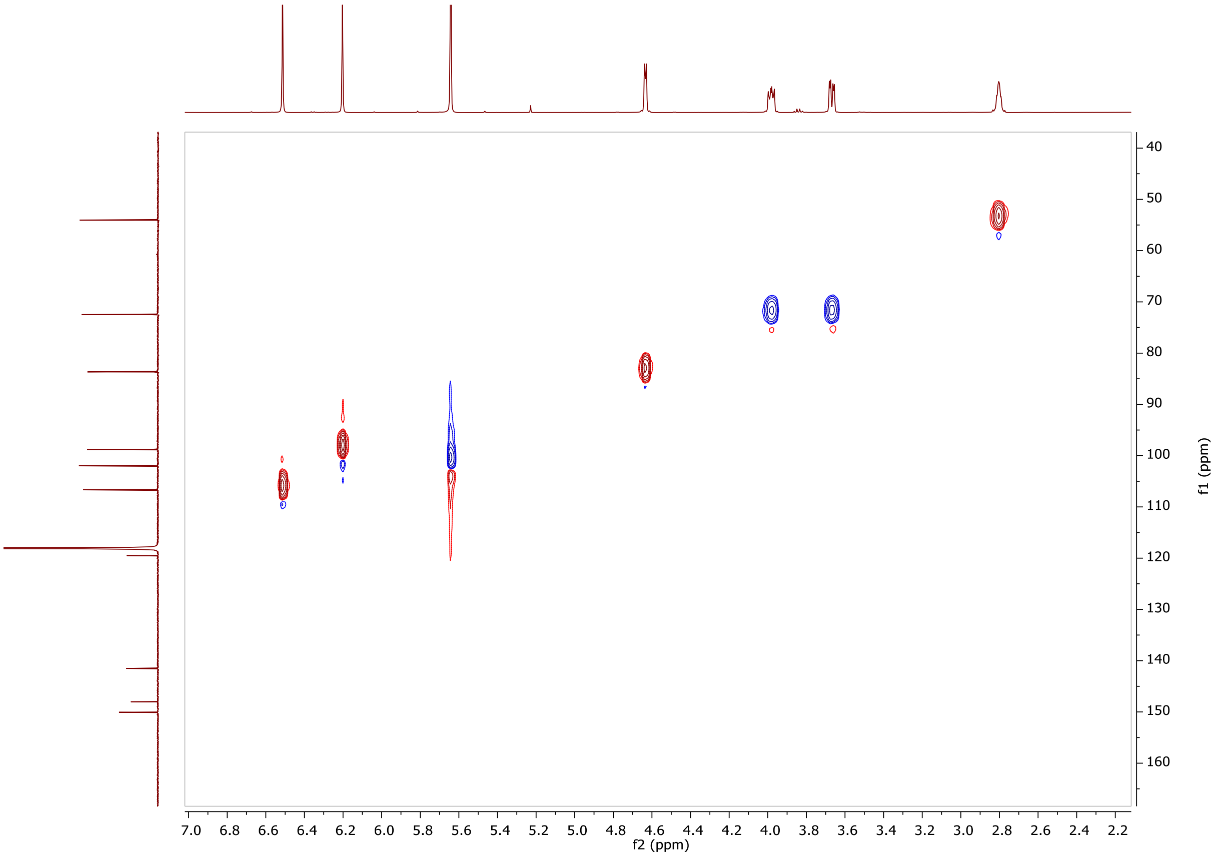

HMBC NMR (500 MHz, acetonitrile-*d*3) of diol **50**:

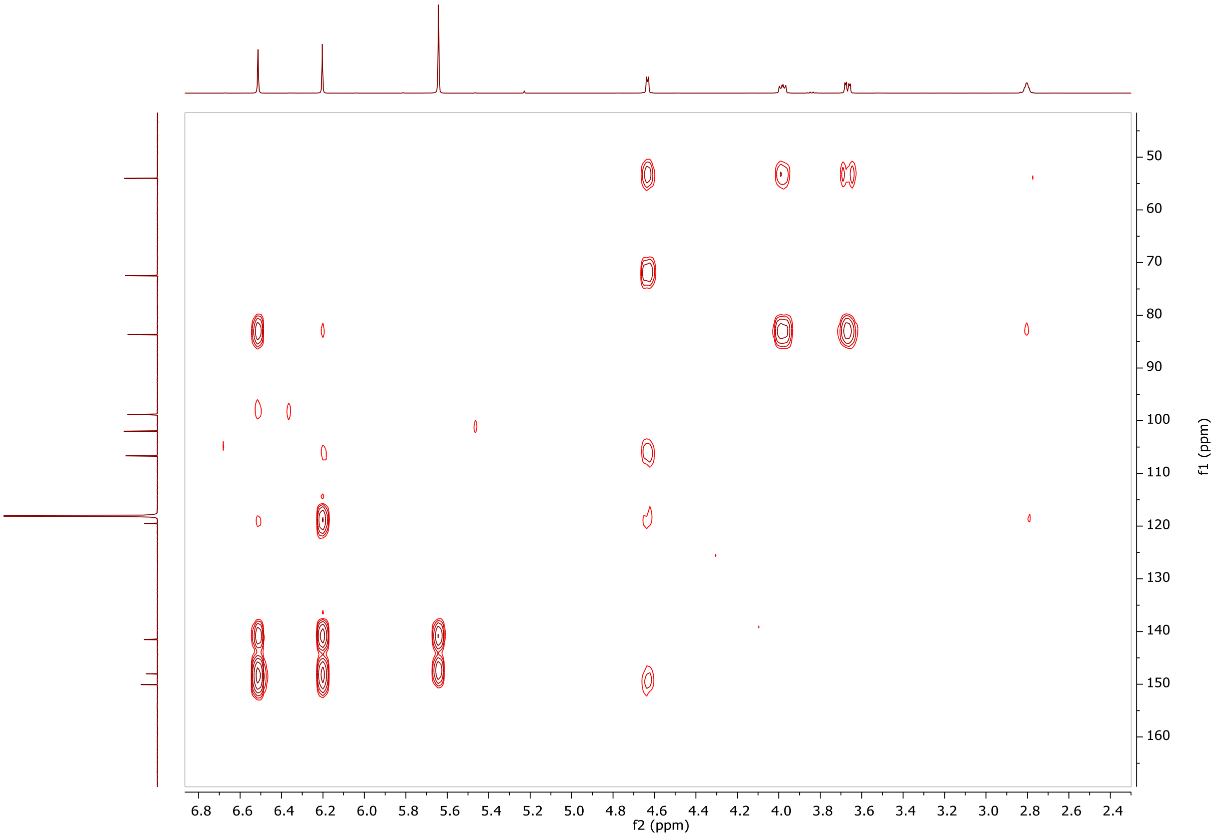

NOESY NMR (500 MHz, acetonitrile-*d*3) of diol **50**:

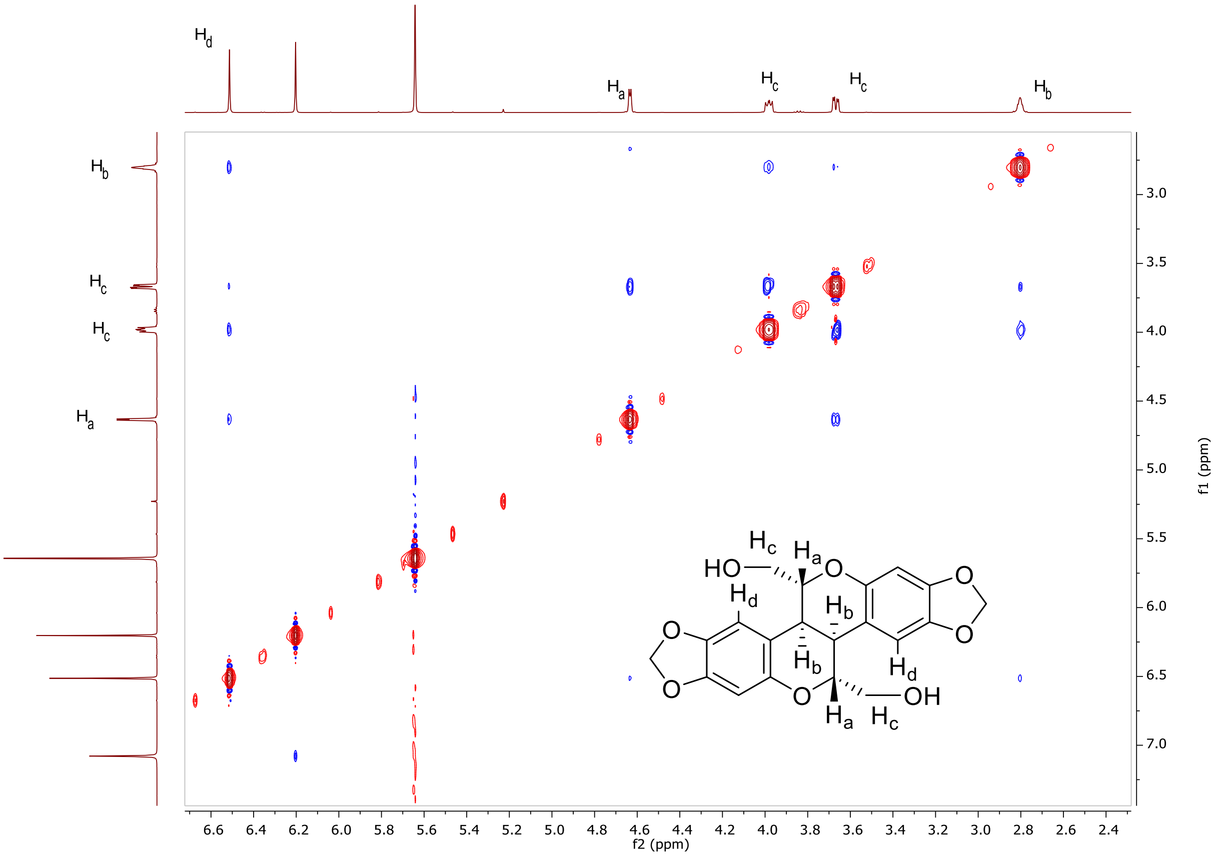

^{1}H NMR (400 MHz, $CDCl_3$) of benzodioxane **49a**:

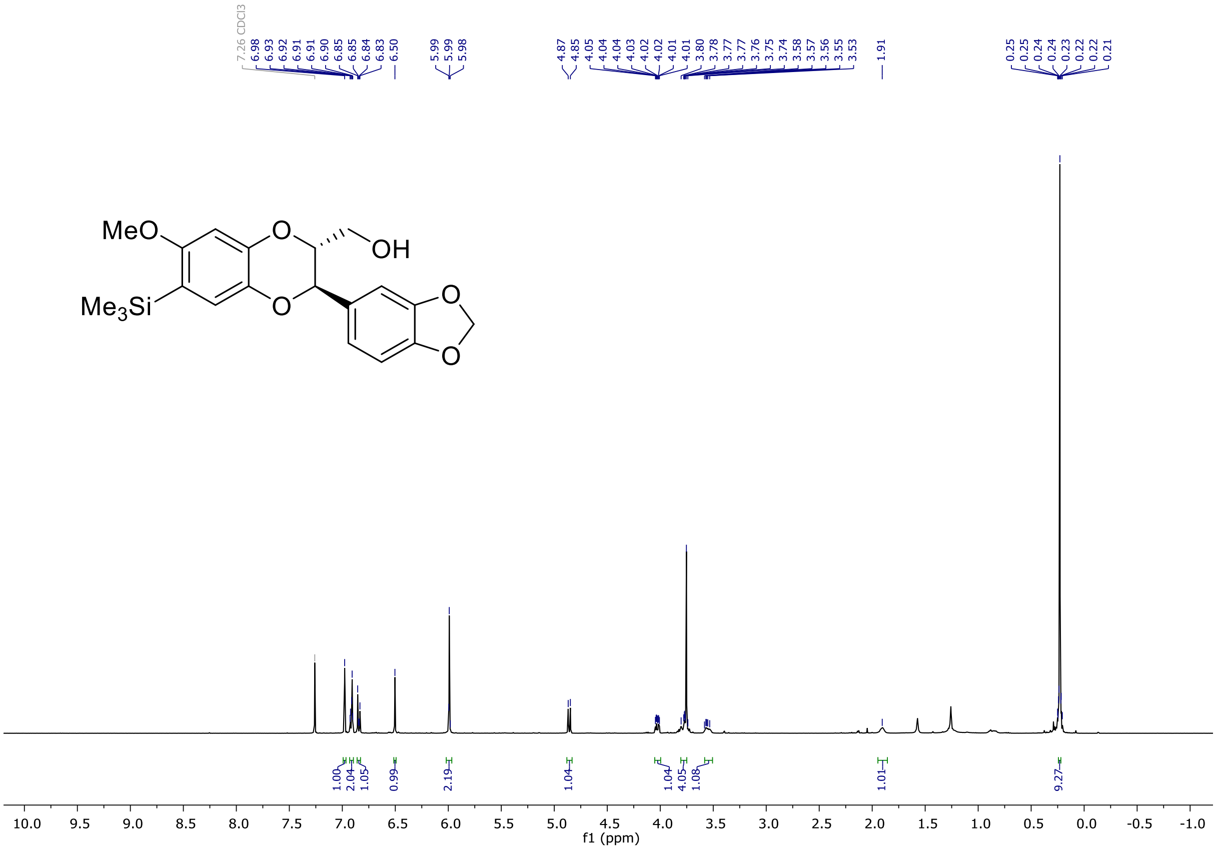

^{13}C NMR (101 MHz, $CDCl_3$) of benzodioxane **49a:**

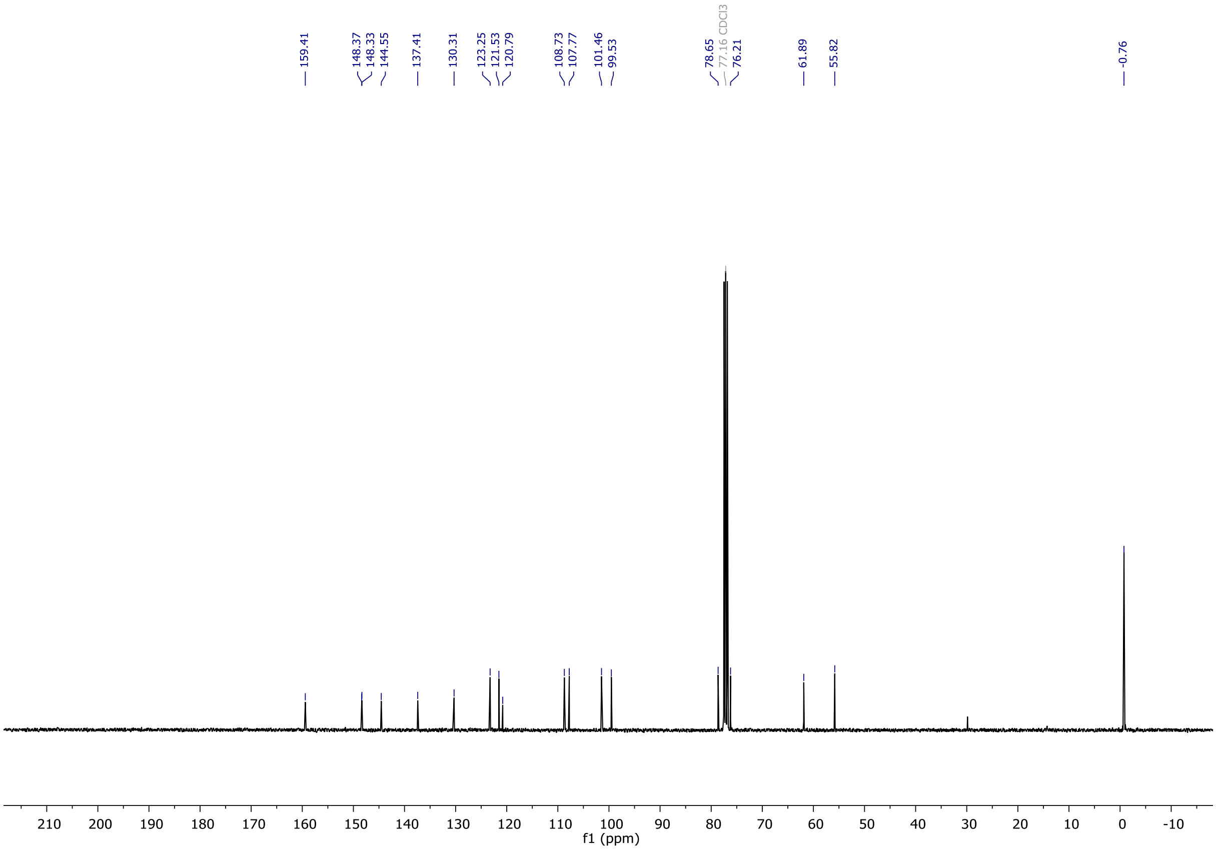

^{1}H NMR (400 MHz, $CDCl_3$) of benzodioxane **SI-22a**:

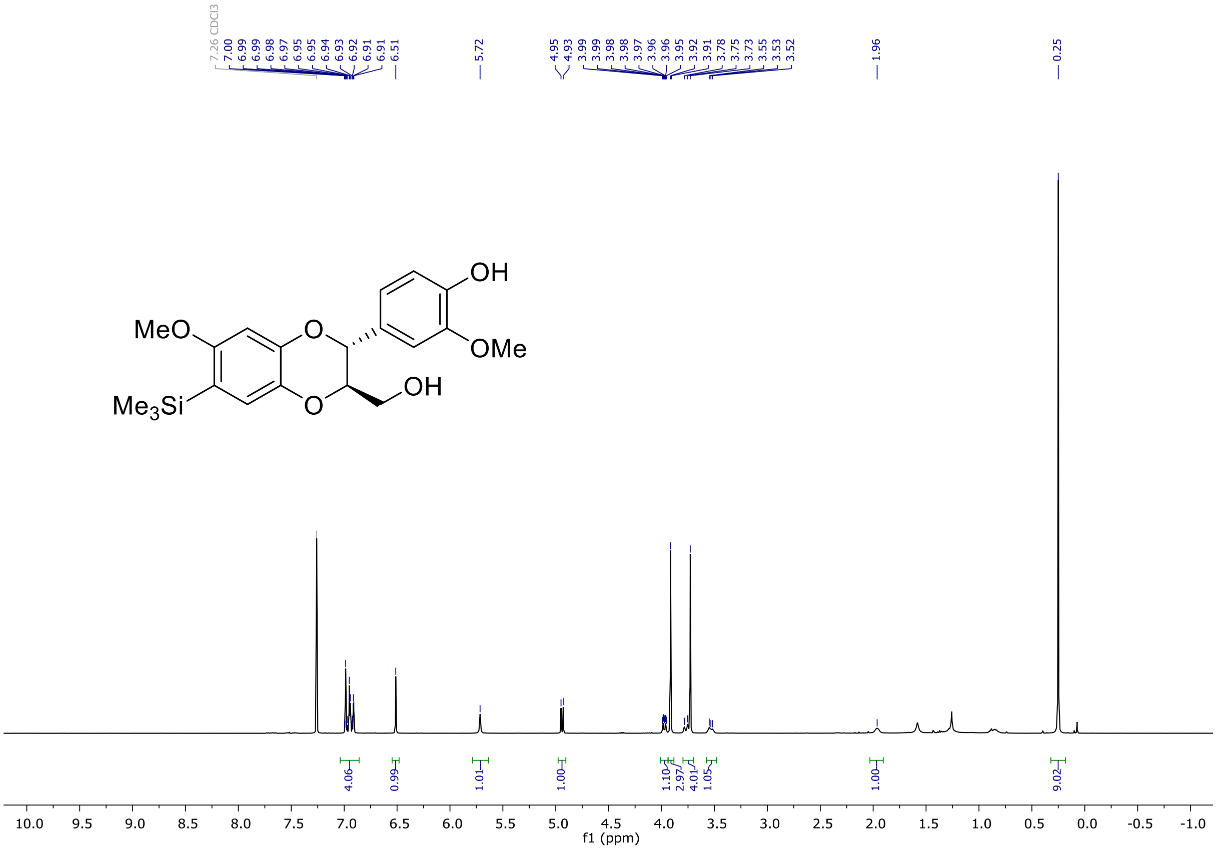

^{13}C NMR (101 MHz, $CDCl_3$) of benzodioxane **SI-22a:**

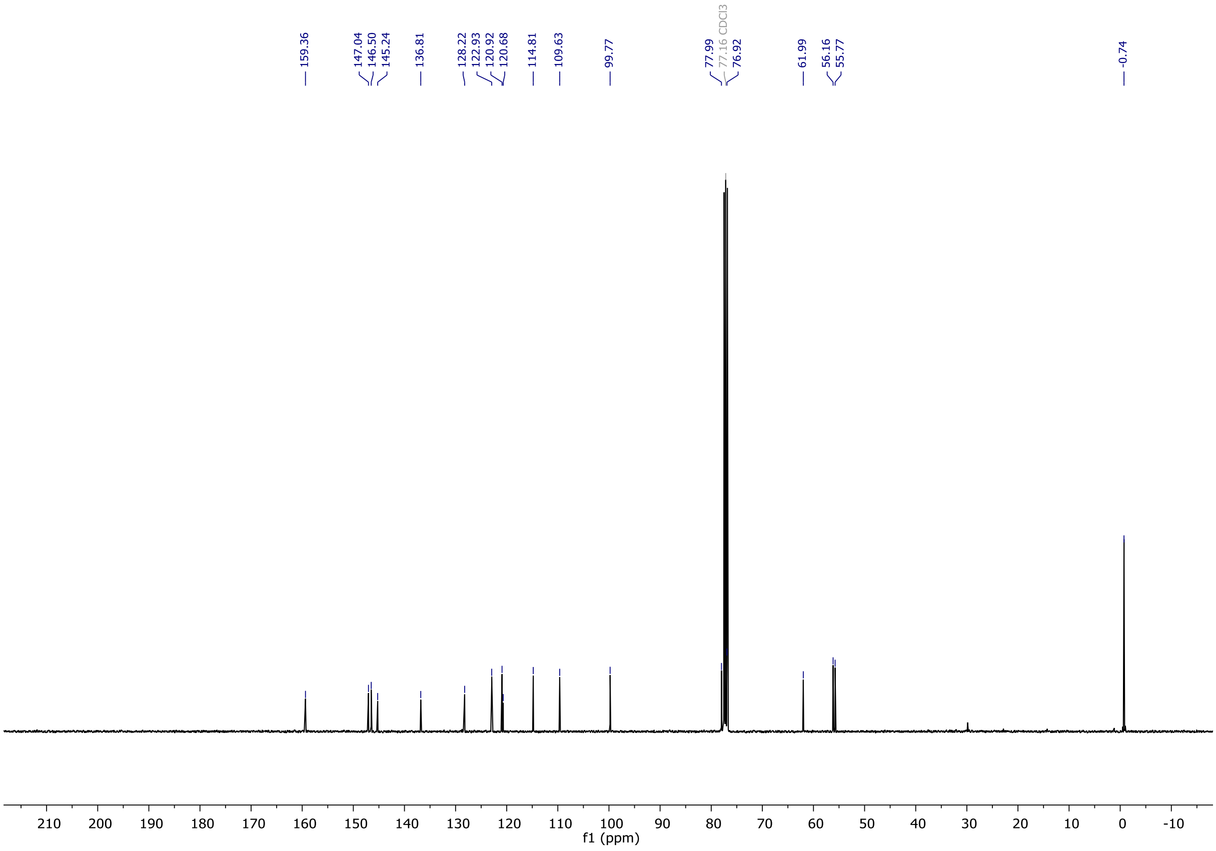

^{1}H NMR (400 MHz, $CDCl_3$) of benzodioxane **SI-22b**:

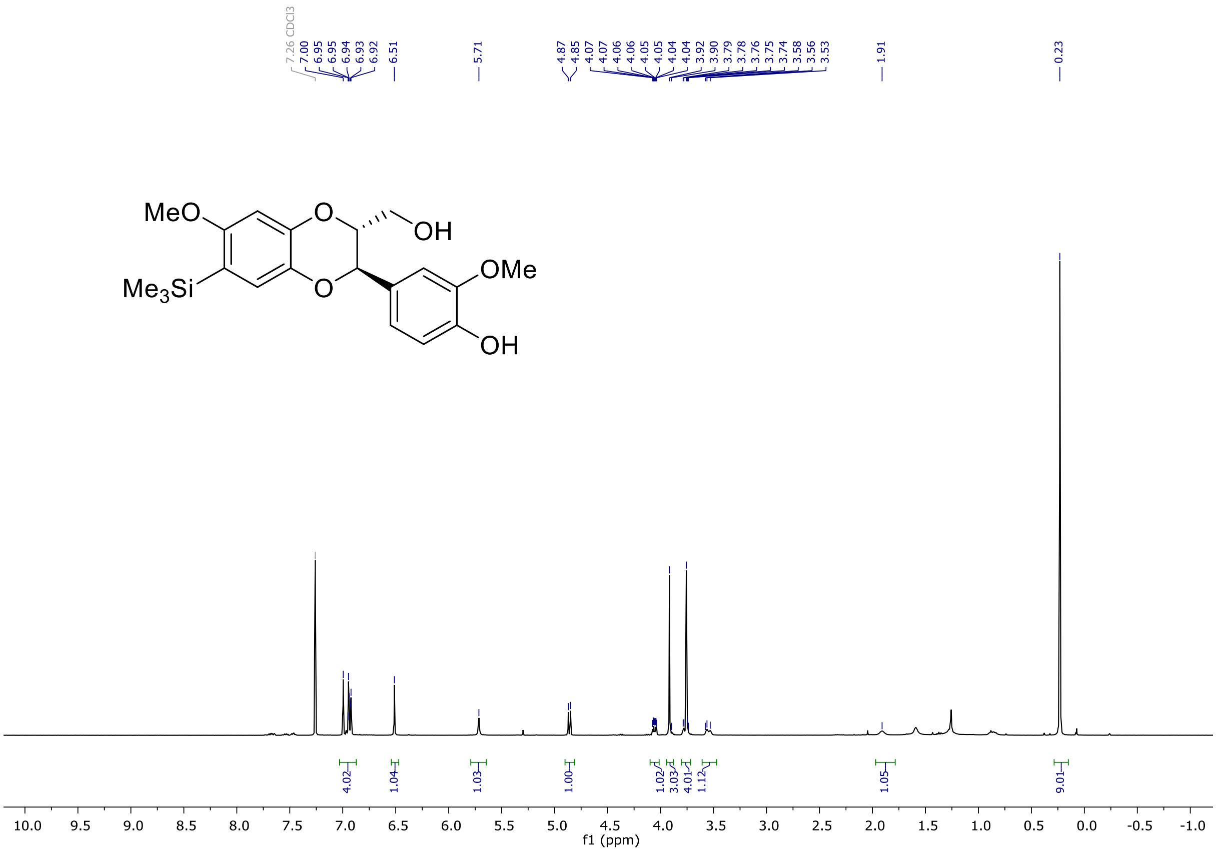

^{13}C NMR (101 MHz, $CDCl_3$) of benzodioxane **SI-22b:**

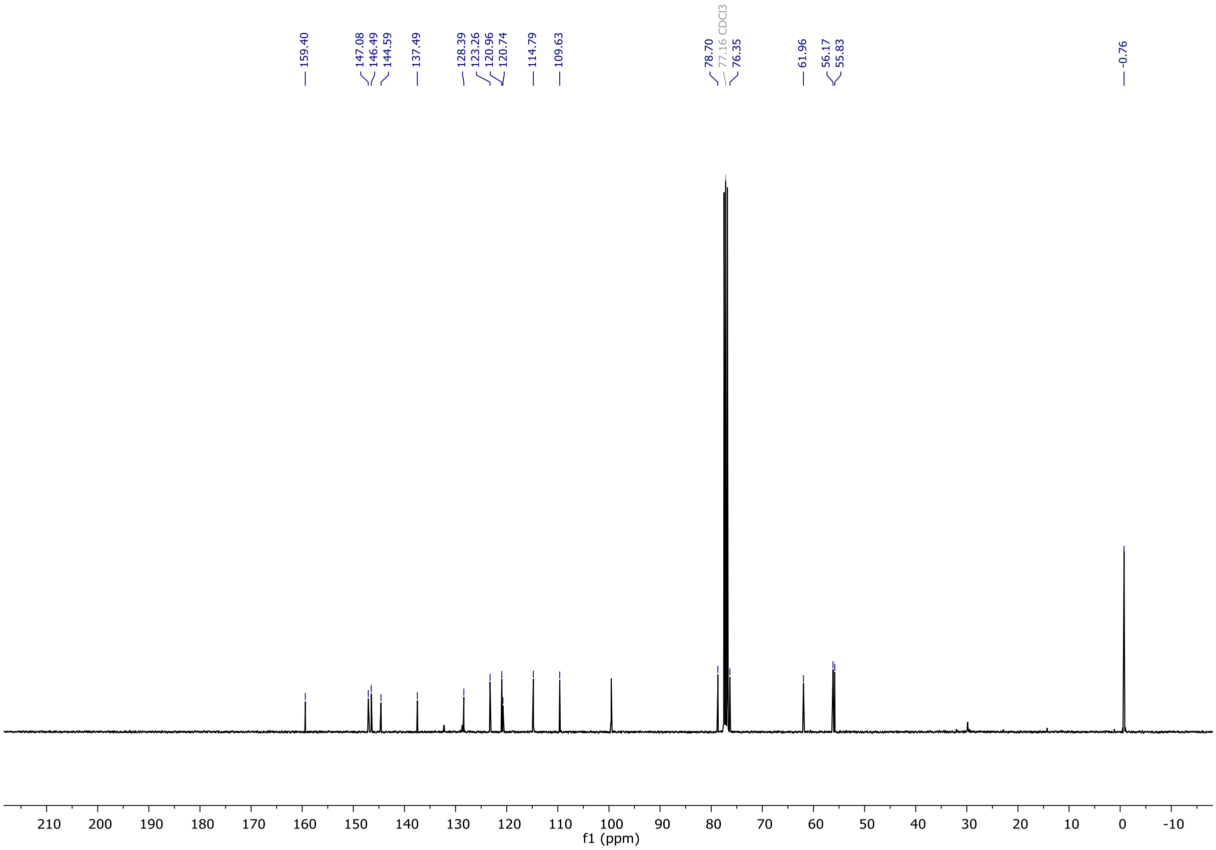

^{1}H NMR (400 MHz, $CDCl_3$) of benzodioxane **SI-23a**:

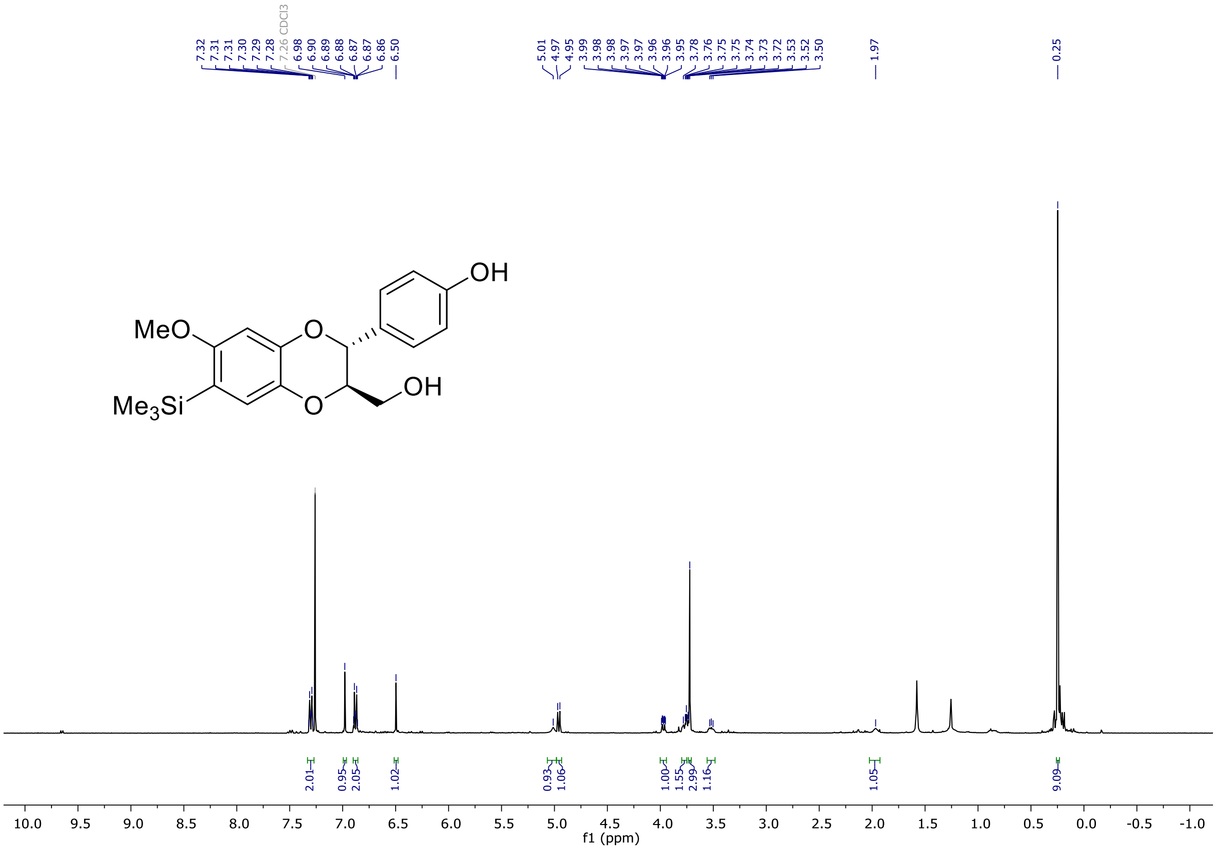

^{13}C NMR (101 MHz, $CDCl_3$) of benzodioxane **SI-23a:**

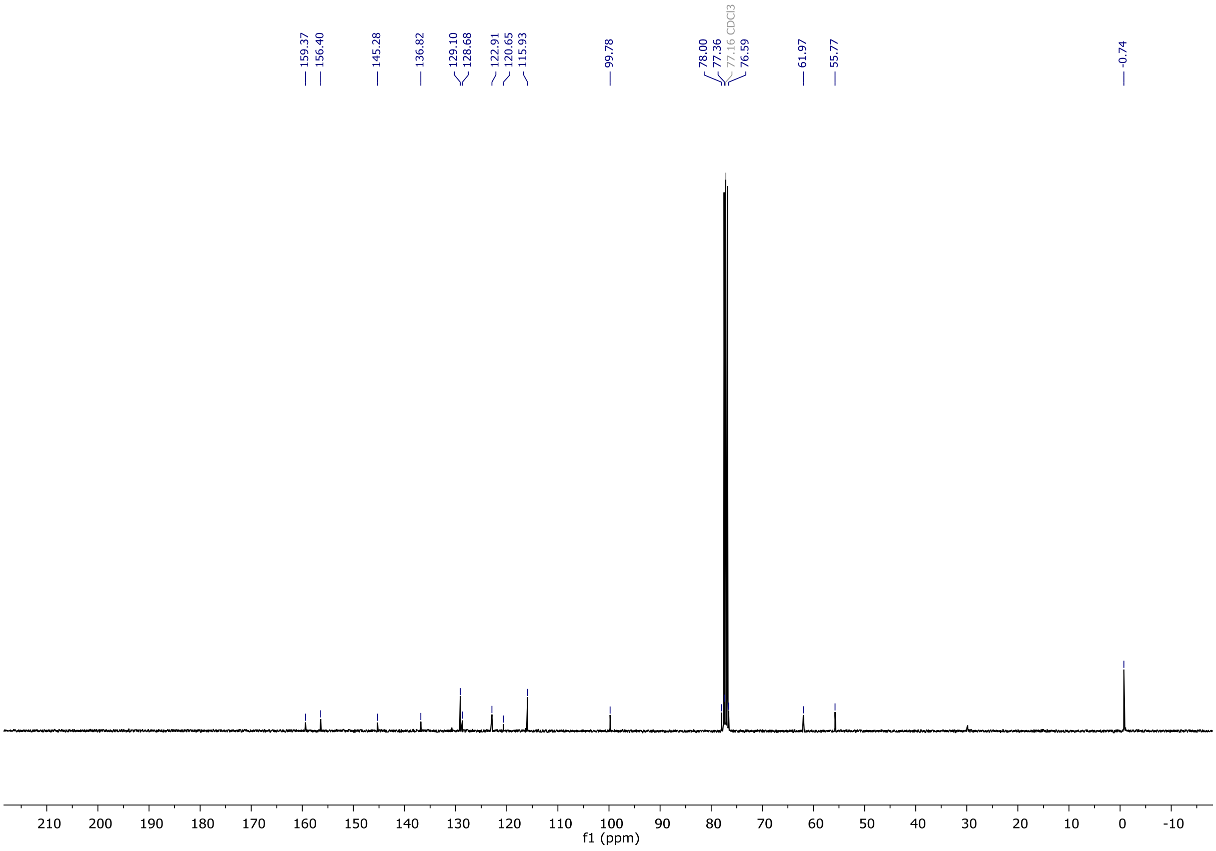

^{1}H NMR (400 MHz, CD_3OD) of benzodioxane **SI-23b**:

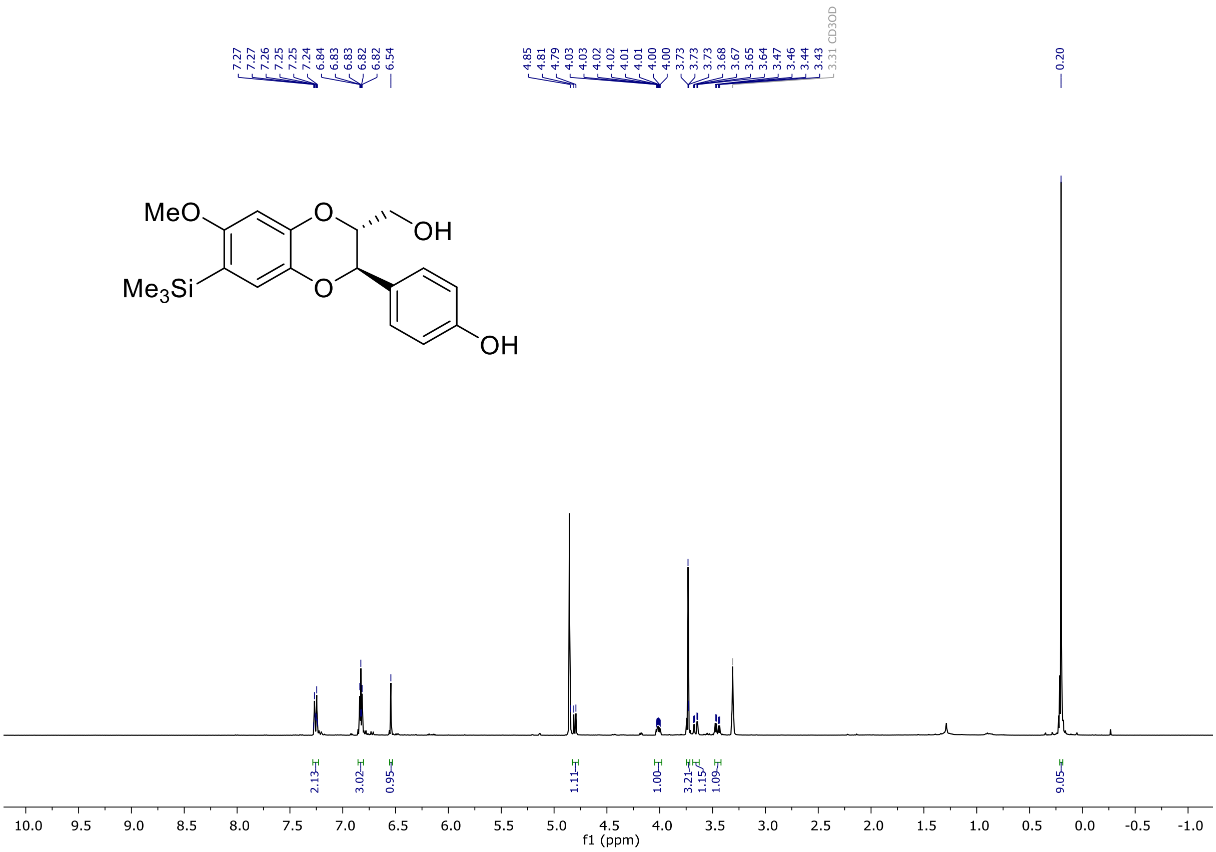

^{13}C NMR (101 MHz, CD_3OD) of benzodioxane **SI-23b:**

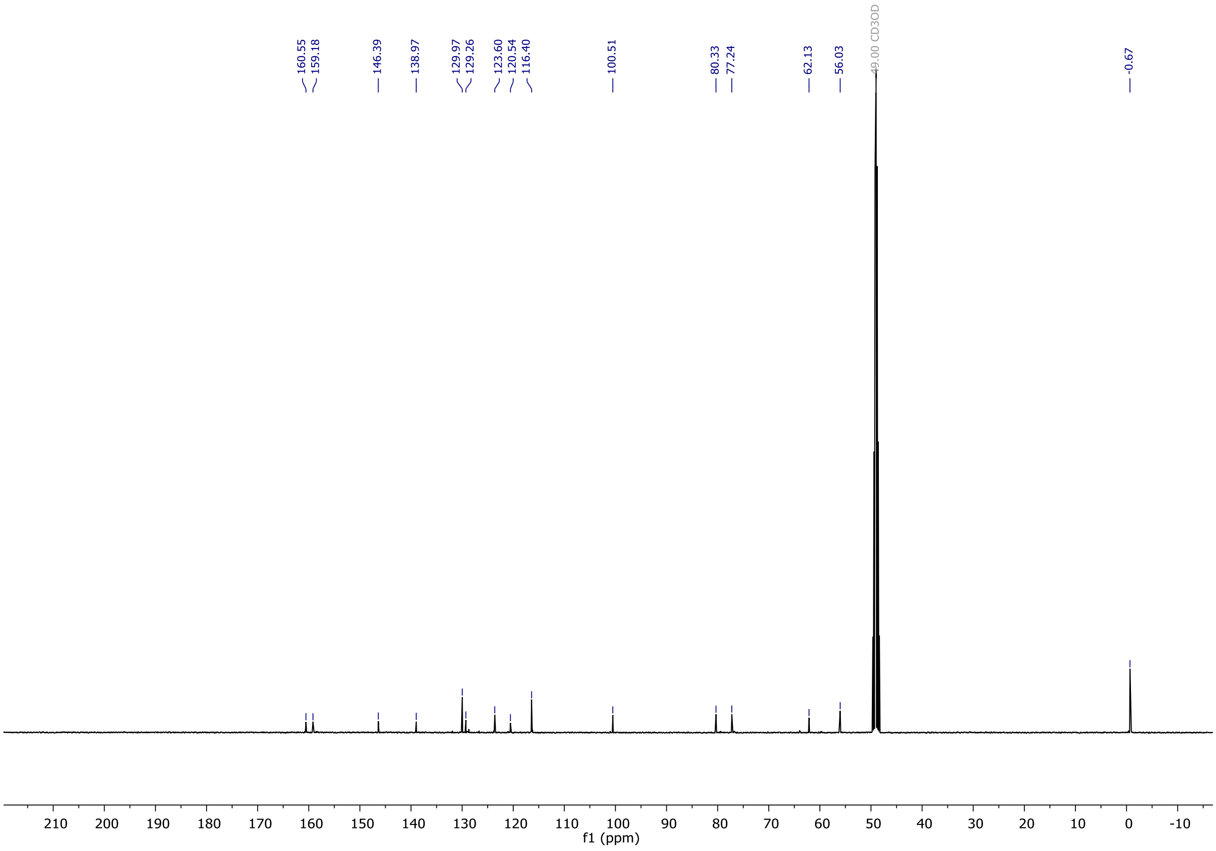

^{1}H NMR (400 MHz, $CDCl_3$) of benzodioxane **SI-25**:

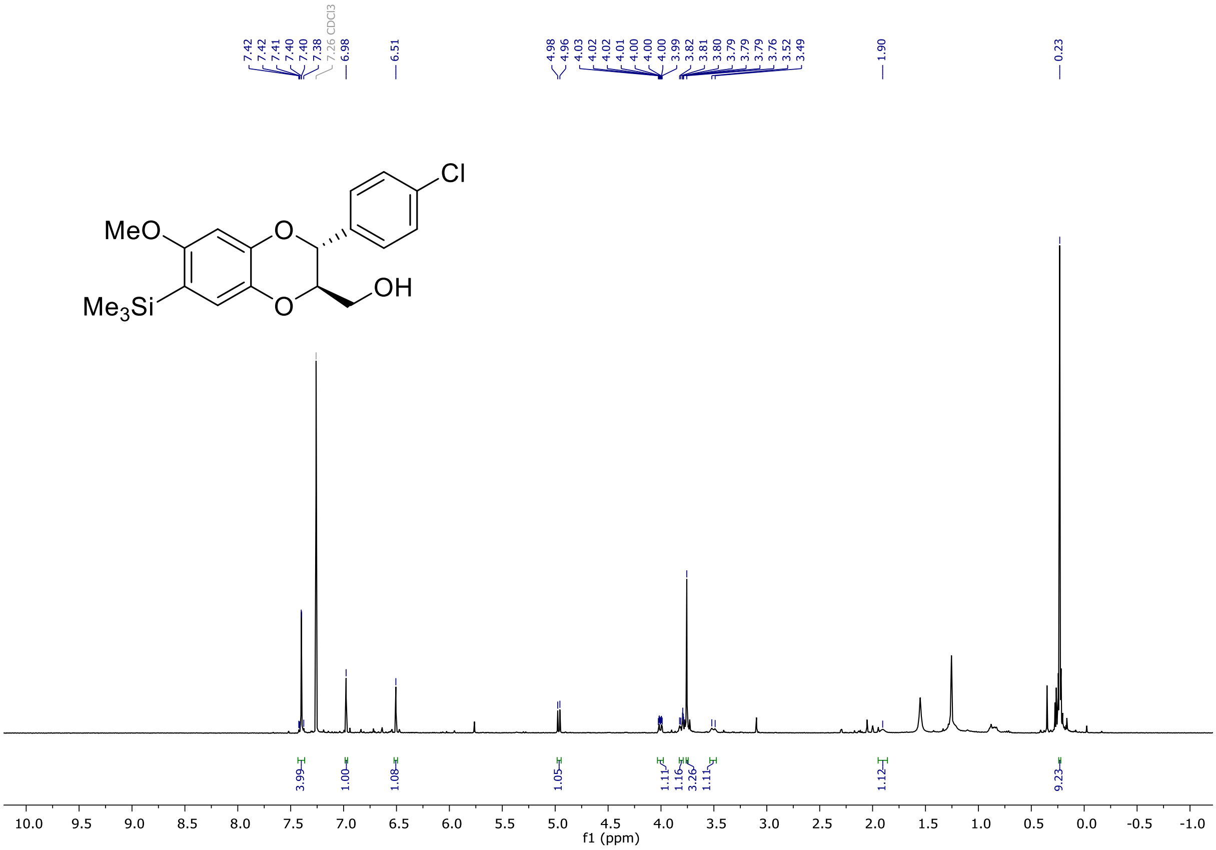

^{13}C NMR (101 MHz, $CDCl_3$) of benzodioxane **SI-25:**

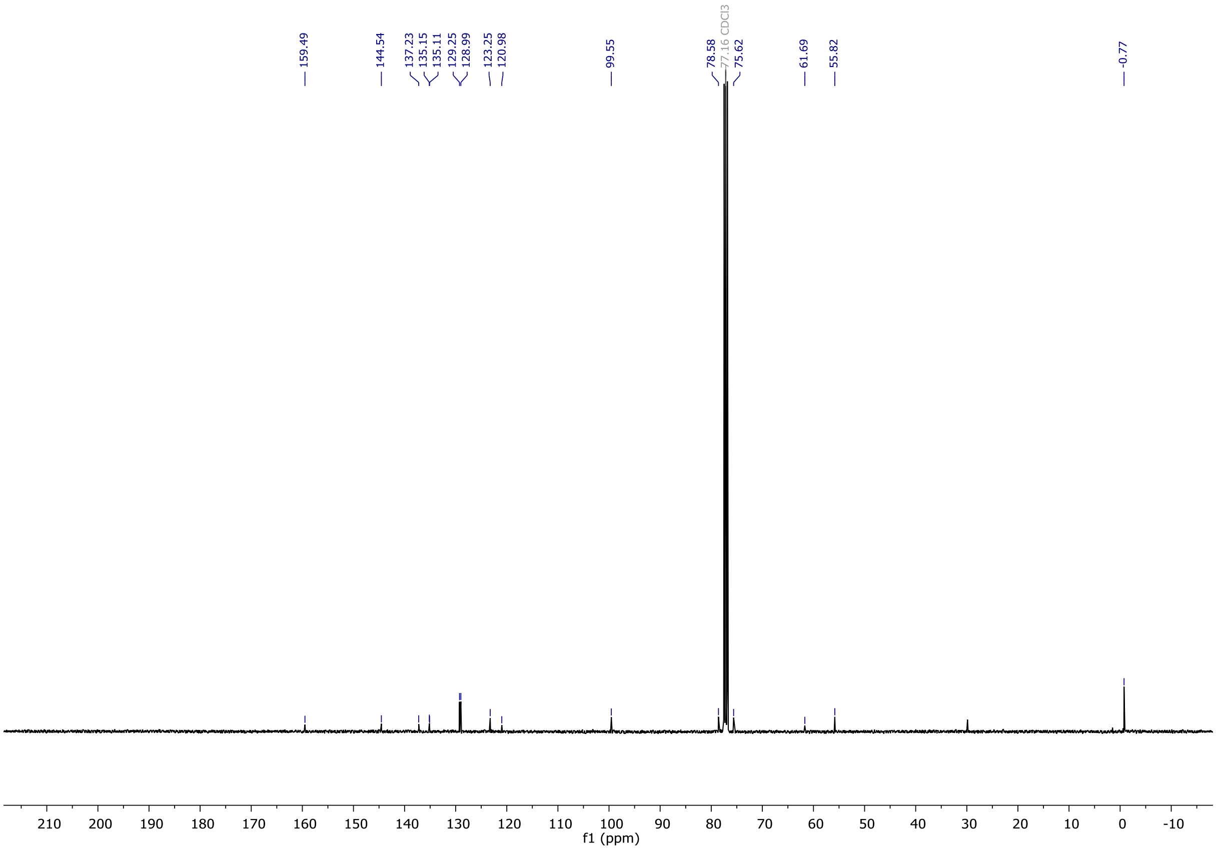

^{1}H NMR (400 MHz, $CDCl_3$) of benzodioxane **SI-26**:

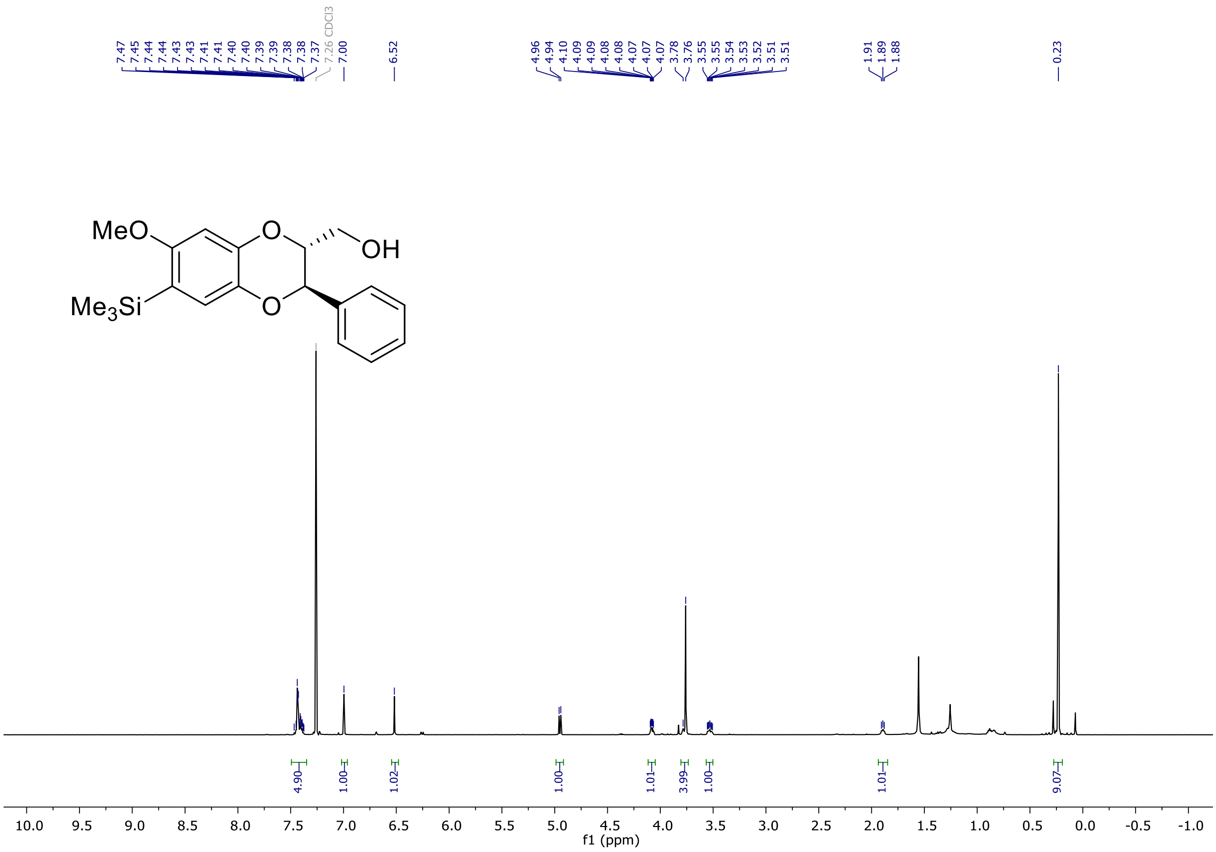

^{13}C NMR (101 MHz, $CDCl_3$) of benzodioxane **SI-26:**

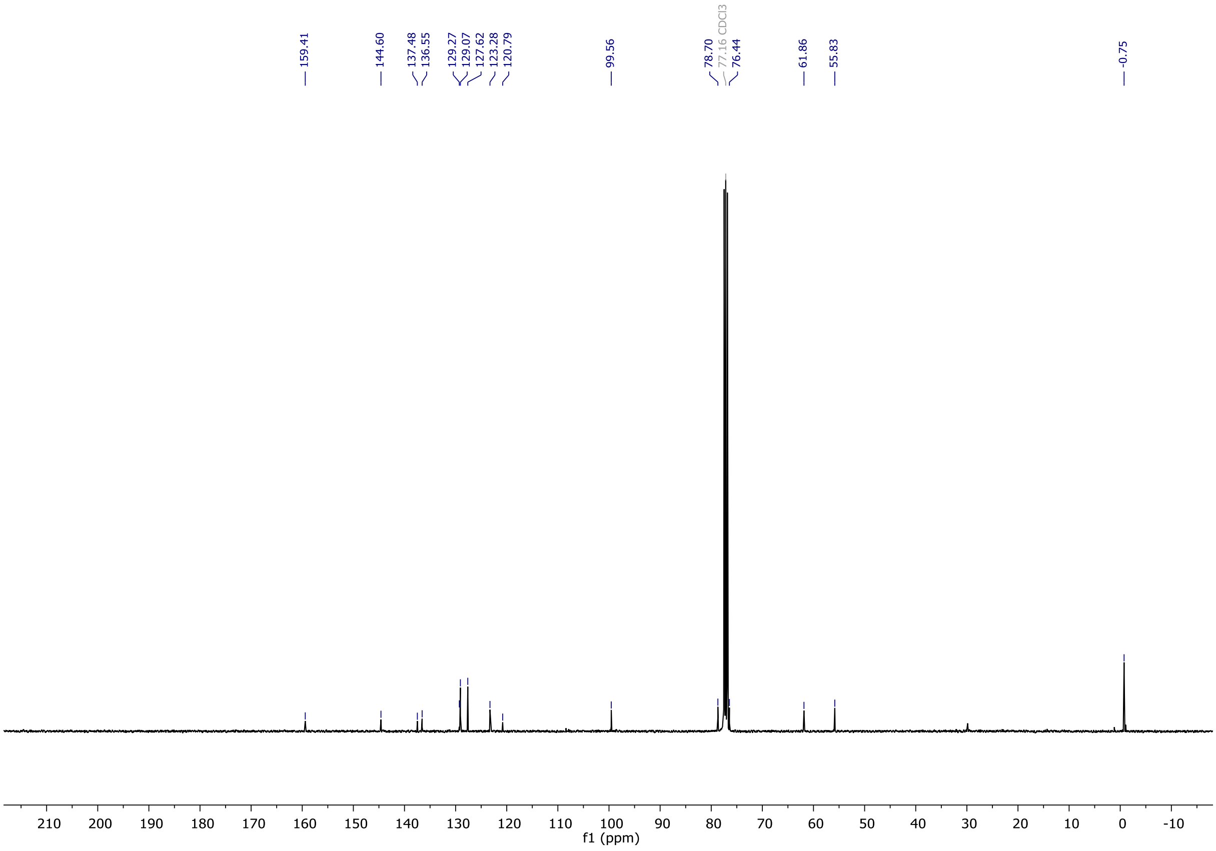

^{1}H NMR (400 MHz, $CDCl_3$) of benzodioxane **SI-28**:

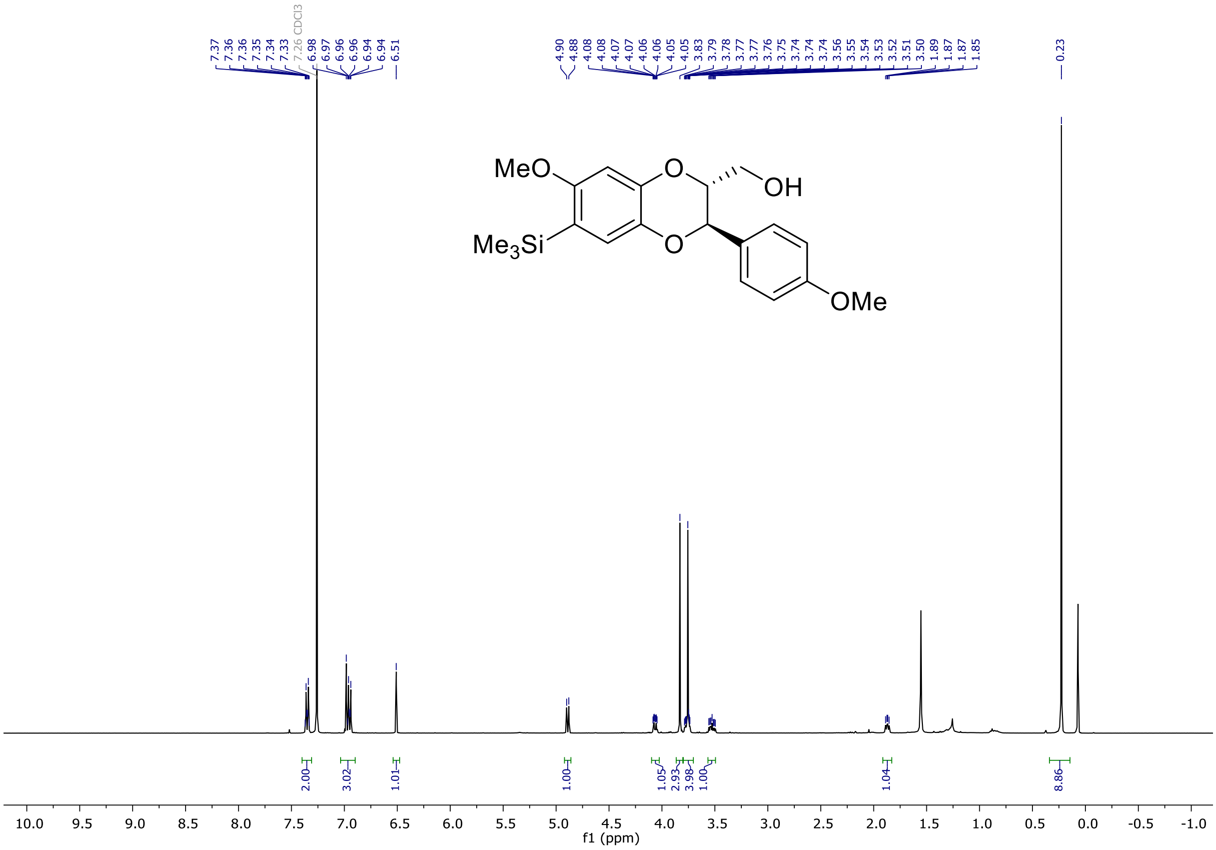

^{13}C NMR (101 MHz, $CDCl_3$) of benzodioxane **SI-28:**

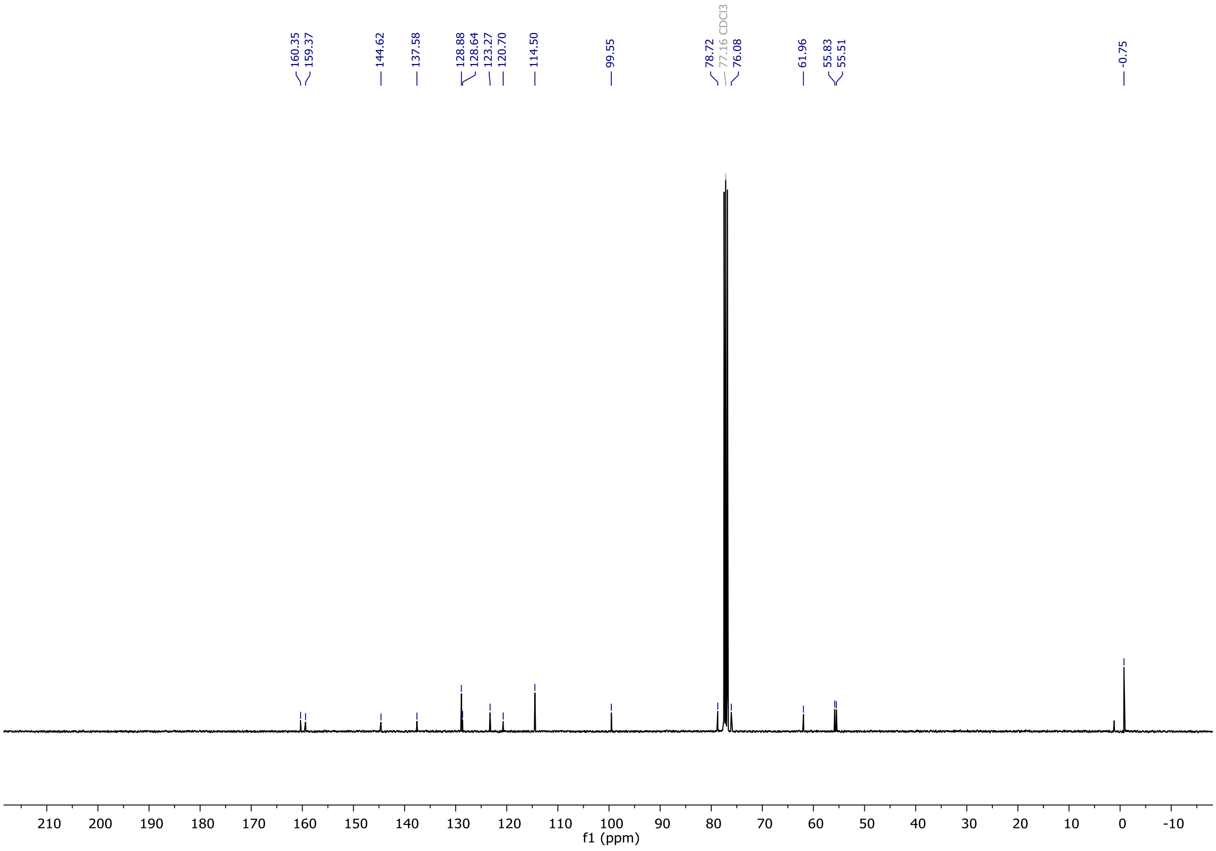

^{1}H NMR (400 MHz, $CDCl_3$) of dioxolane **SI-29**:

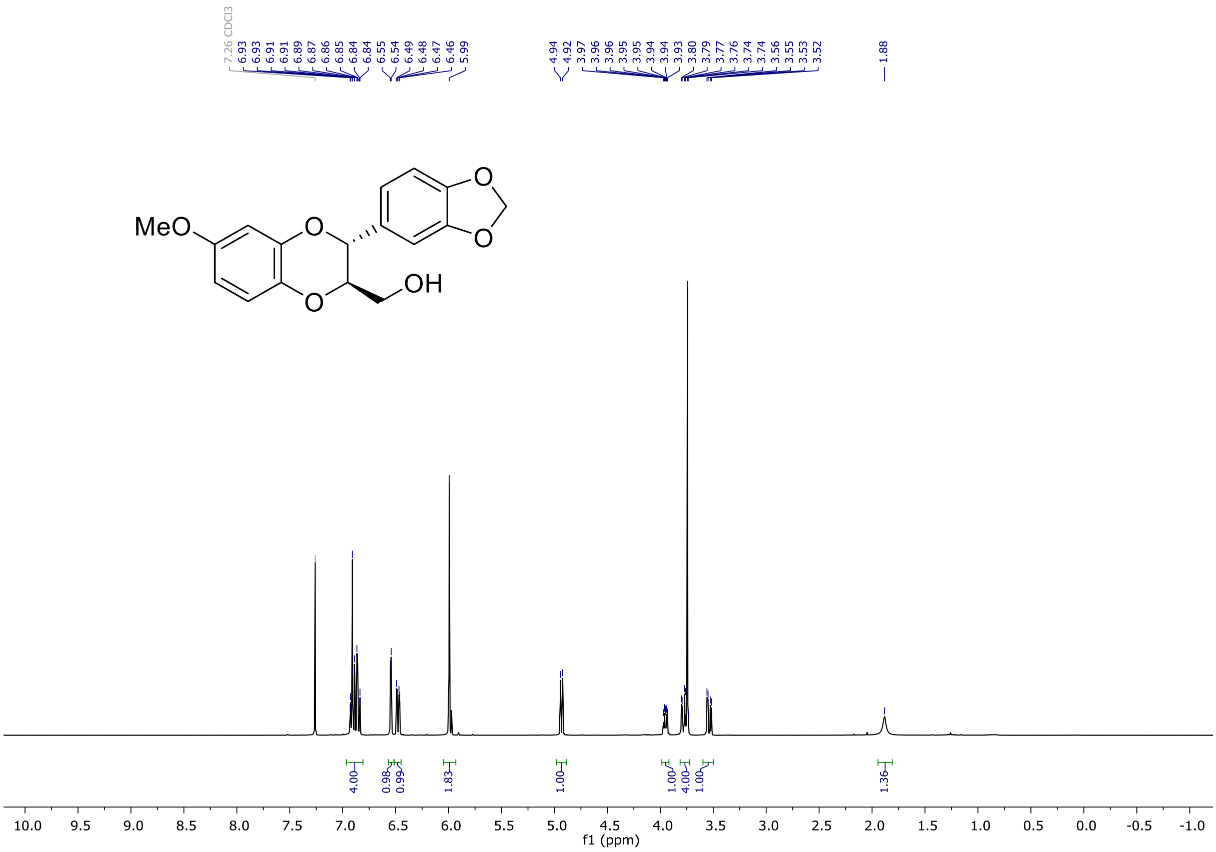

^{1}H NMR (400 MHz, $CDCl_3$) of benzodioxane *trans*-**48a**:

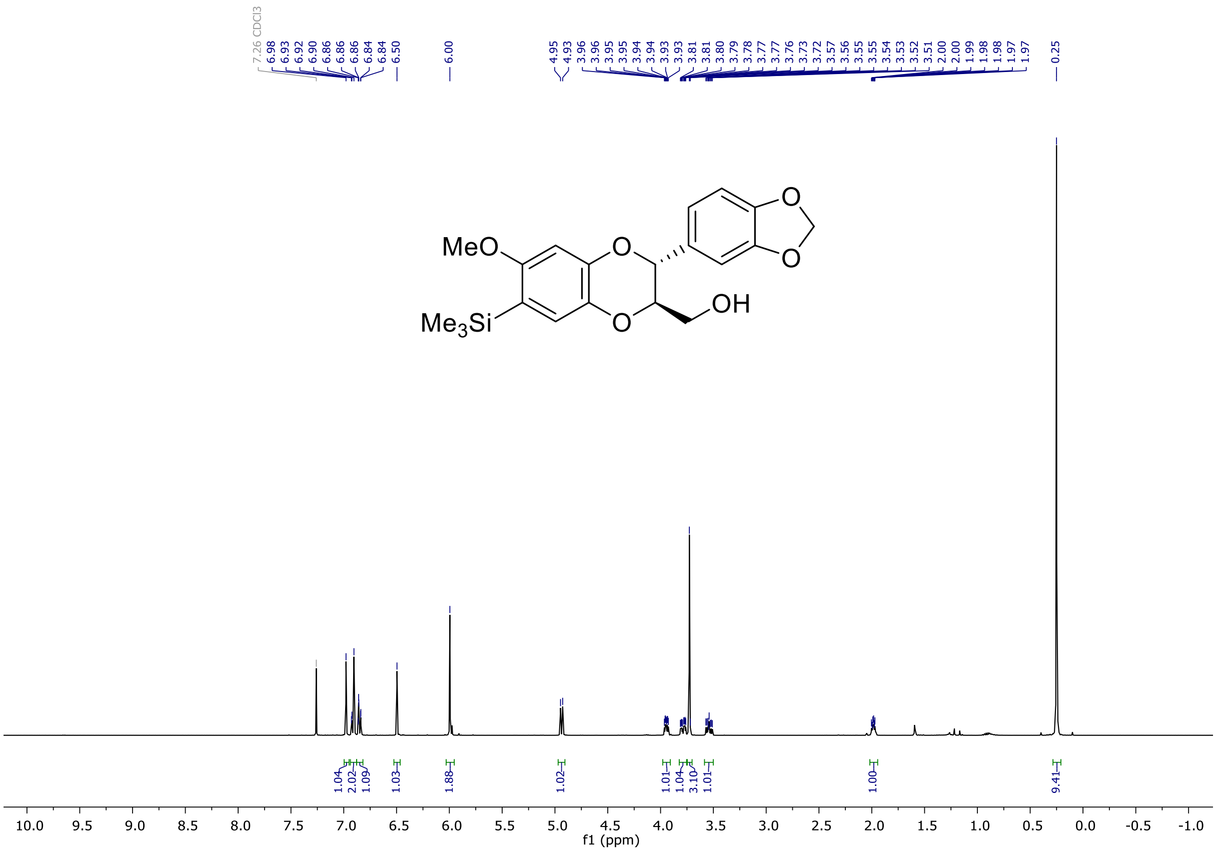

^{13}C NMR (101 MHz, $CDCl_3$) of benzodioxane *trans*-**48a:**

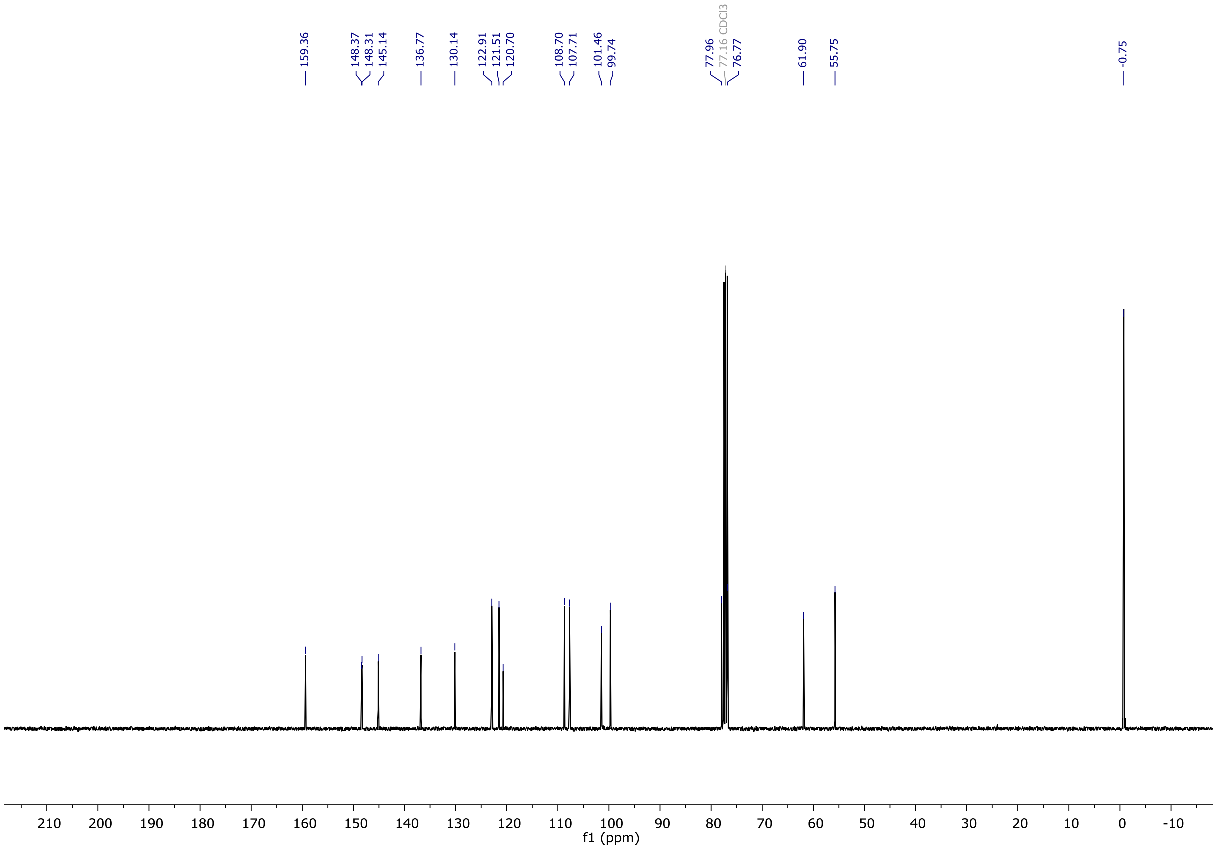

^{1}H NMR (400 MHz, $CDCl_3$) of *cis*-benzodioxane *cis*-**48a**:

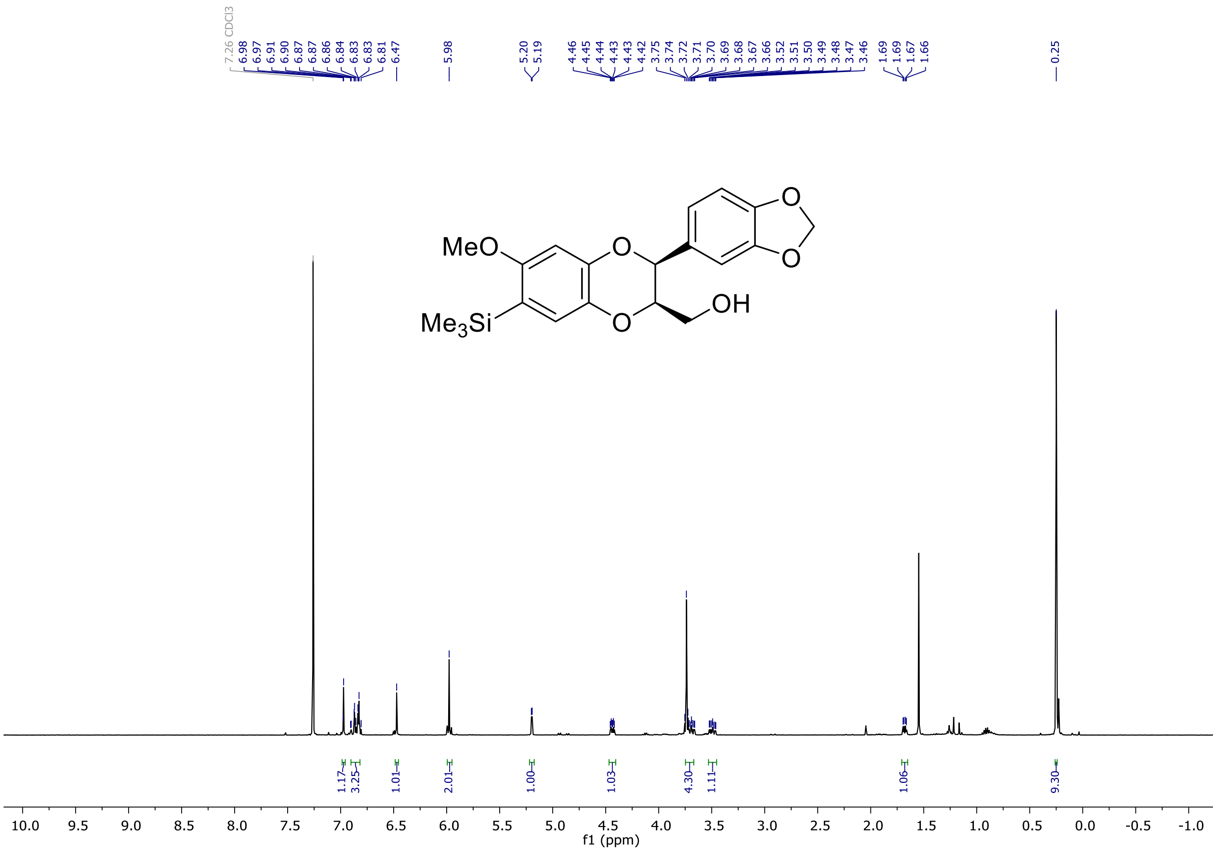

^{13}C NMR (101 MHz, $CDCl_3$) of benzodioxane *cis*-**48a:**

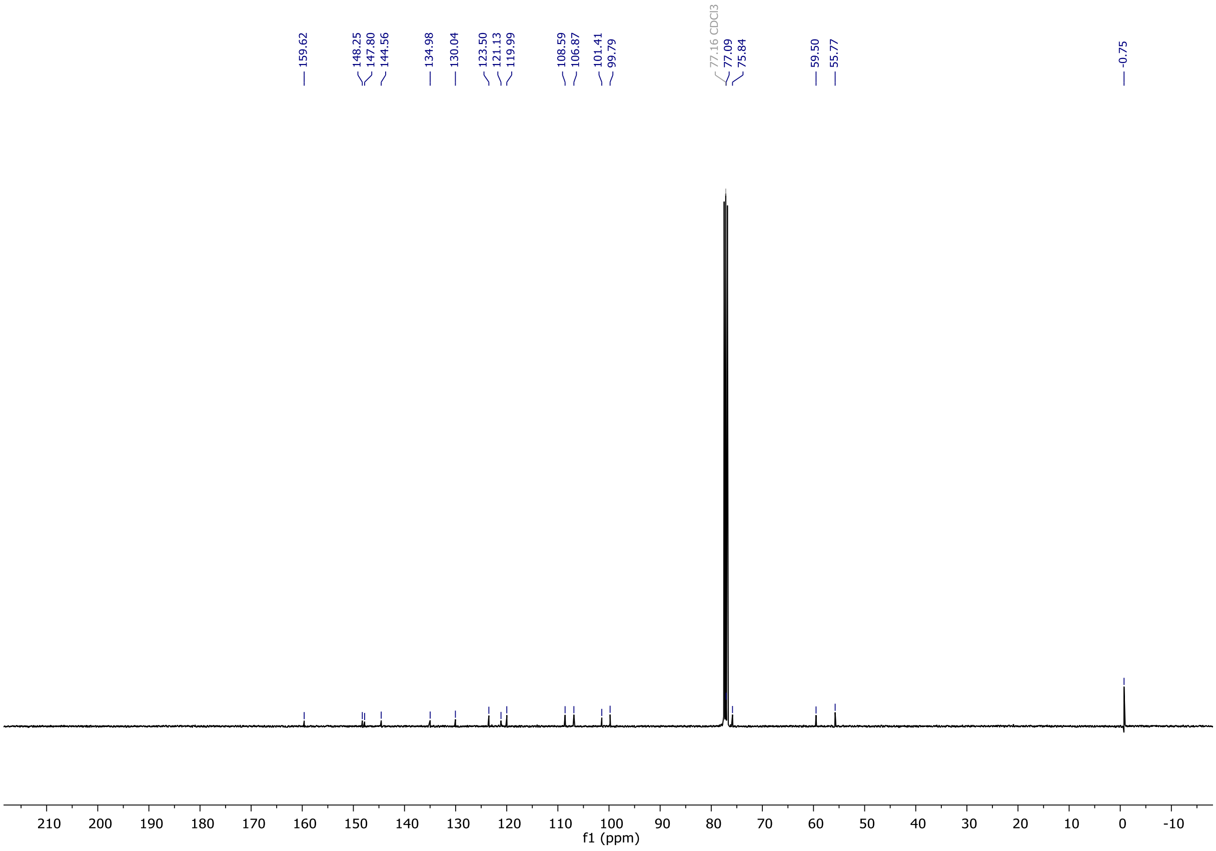

^{1}H NMR (500 MHz, C_6D_6) of benzodioxane **51**:

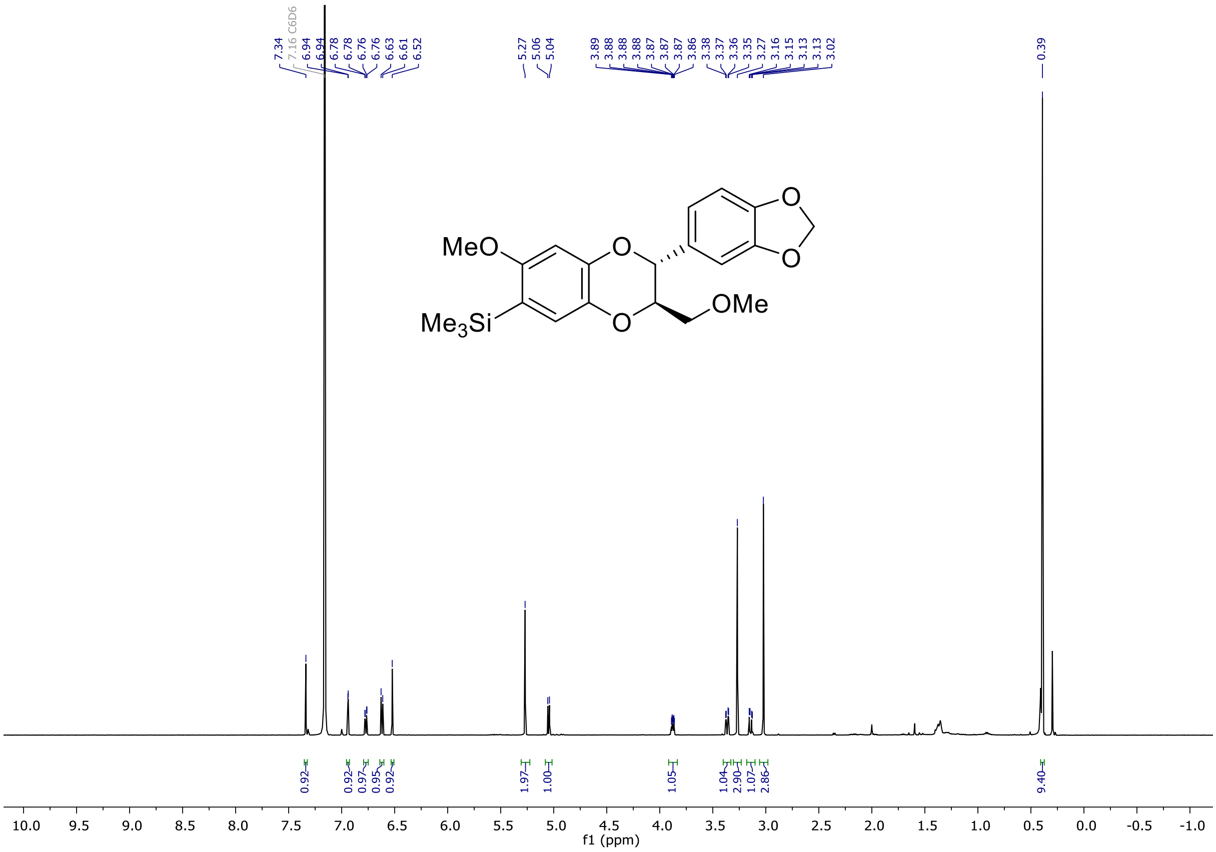

^{13}C NMR (126 MHz, C_6D_6) of benzodioxane **51:**

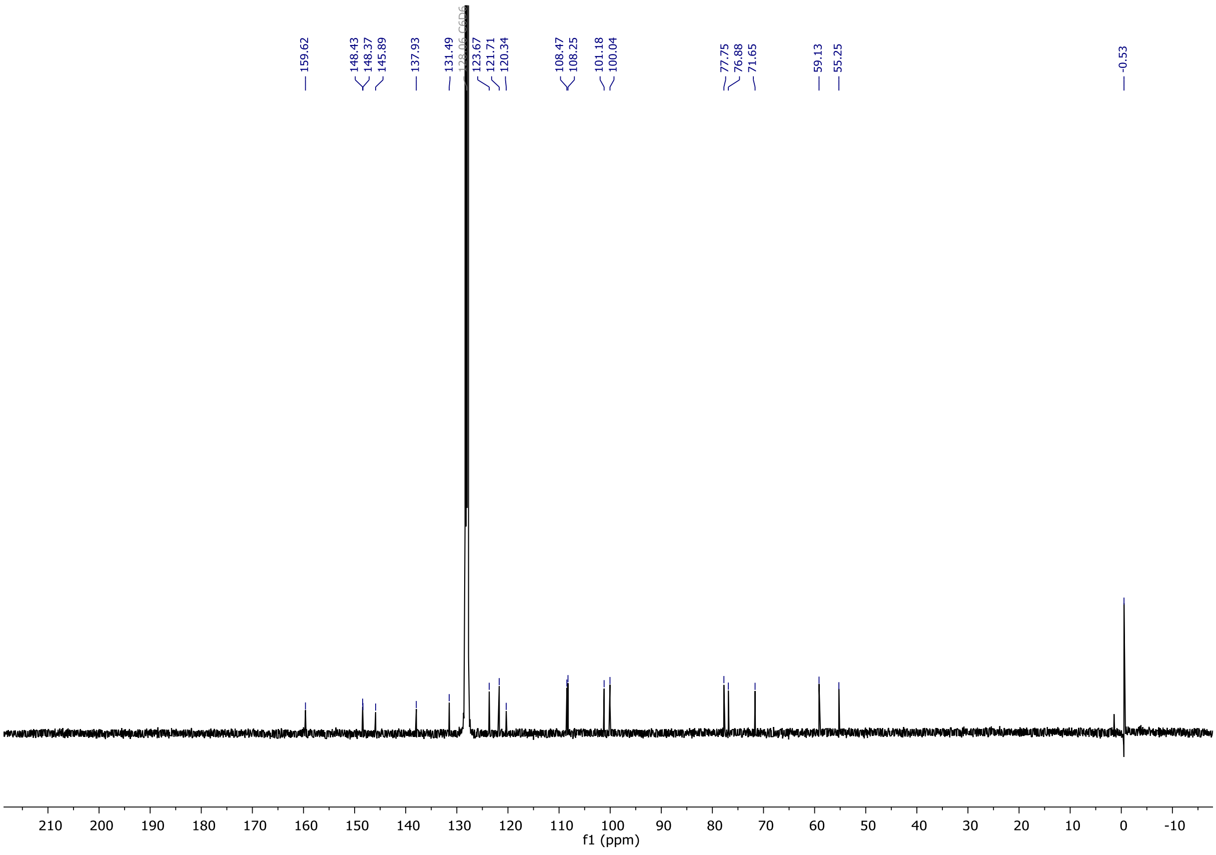

^{1}H NMR (500 MHz, $CDCl_3$) of dioxolane **53**:

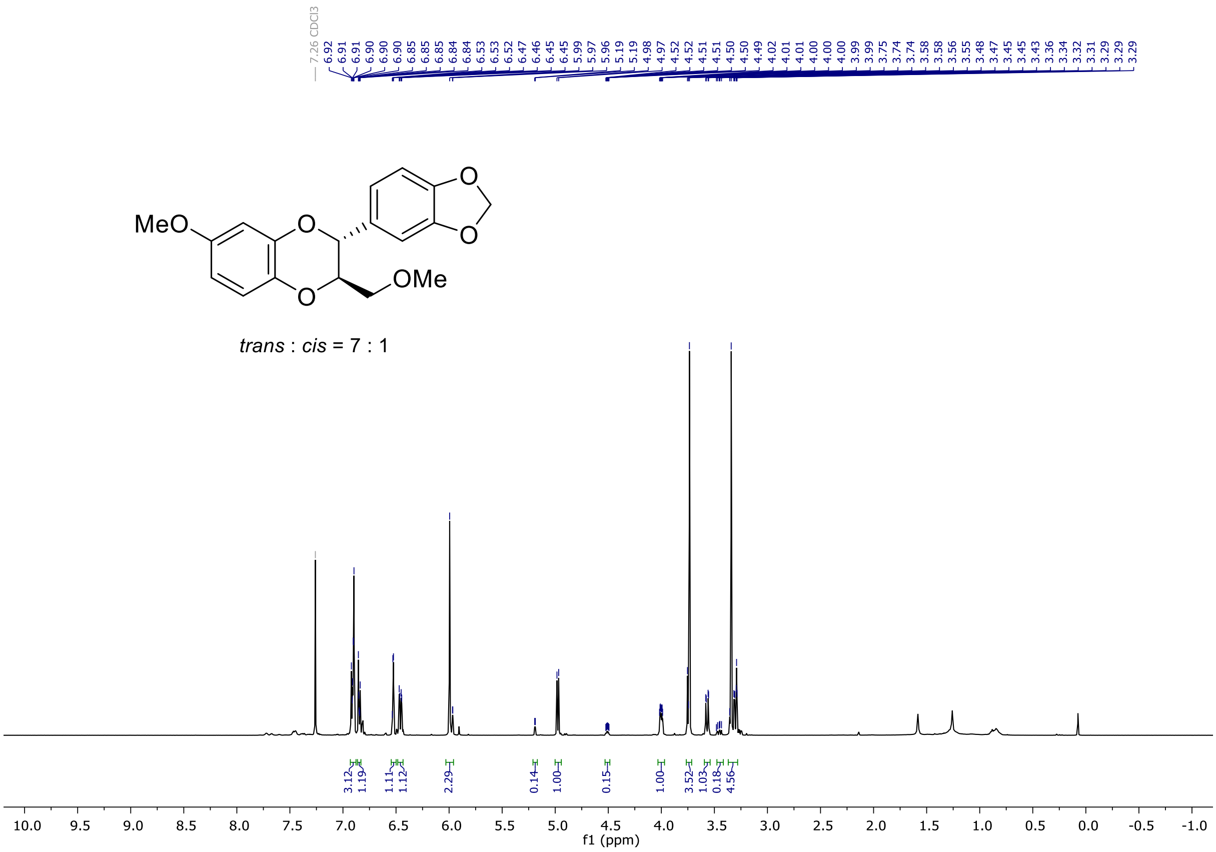

^{13}C NMR (126 MHz, $CDCl_3$) of dioxolane **53:**

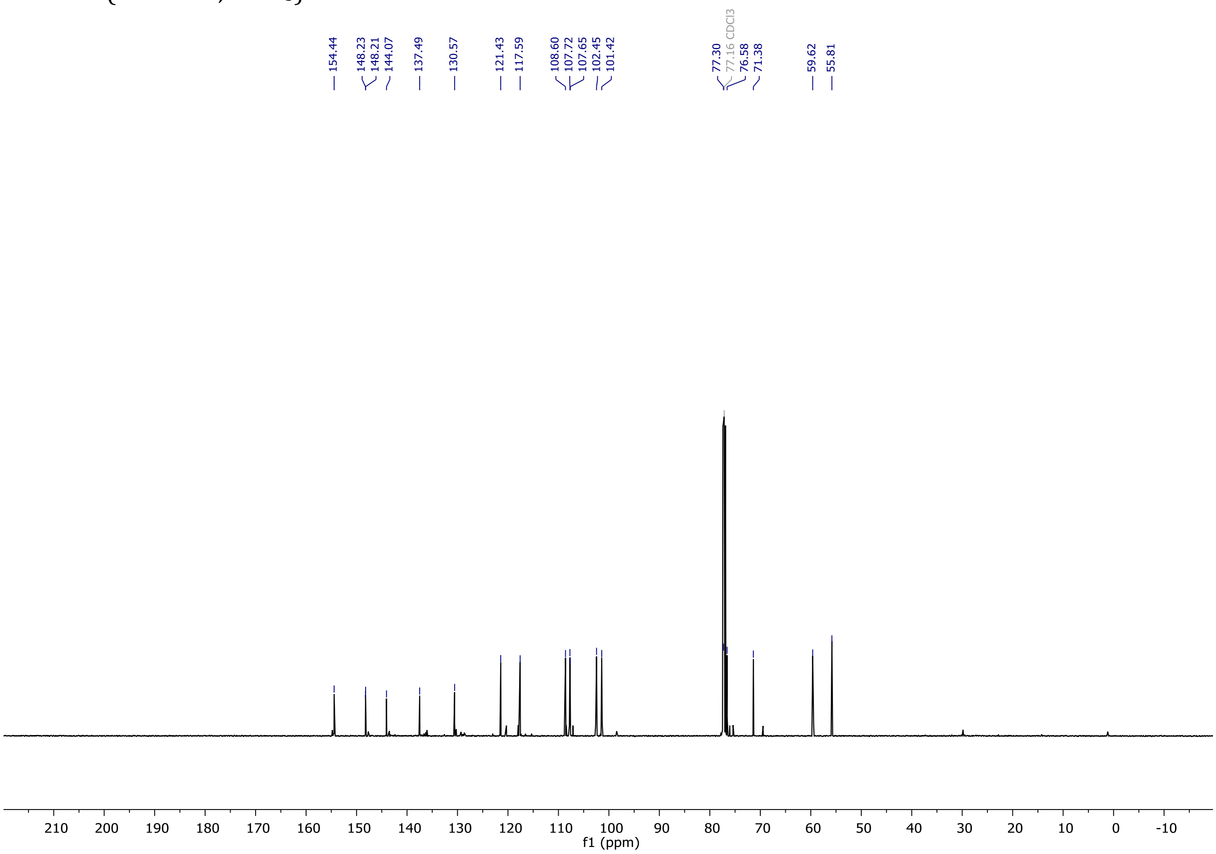

^{1}H NMR (400 MHz, $CDCl_3$) of phenol **28**:

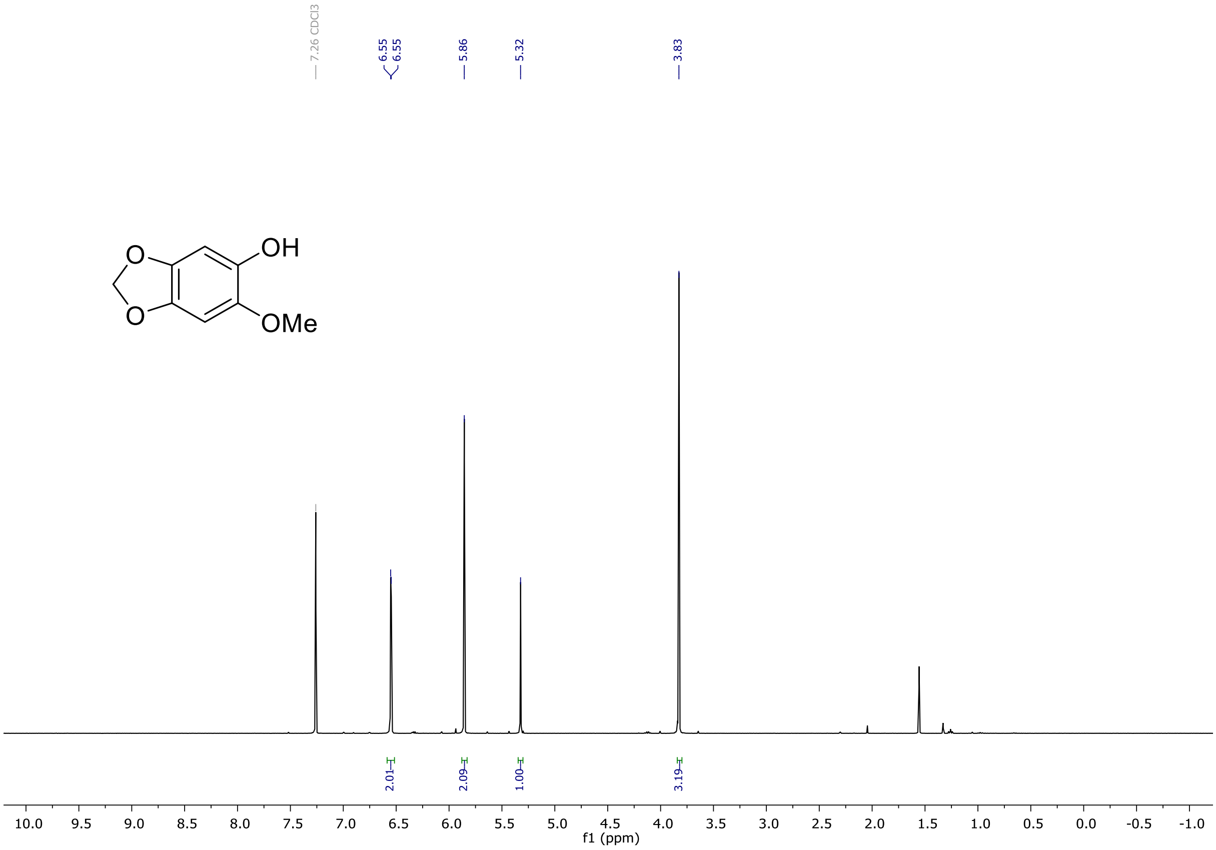

^{1}H NMR (400 MHz, $CDCl_3$) of aldehyde **SI-31**:

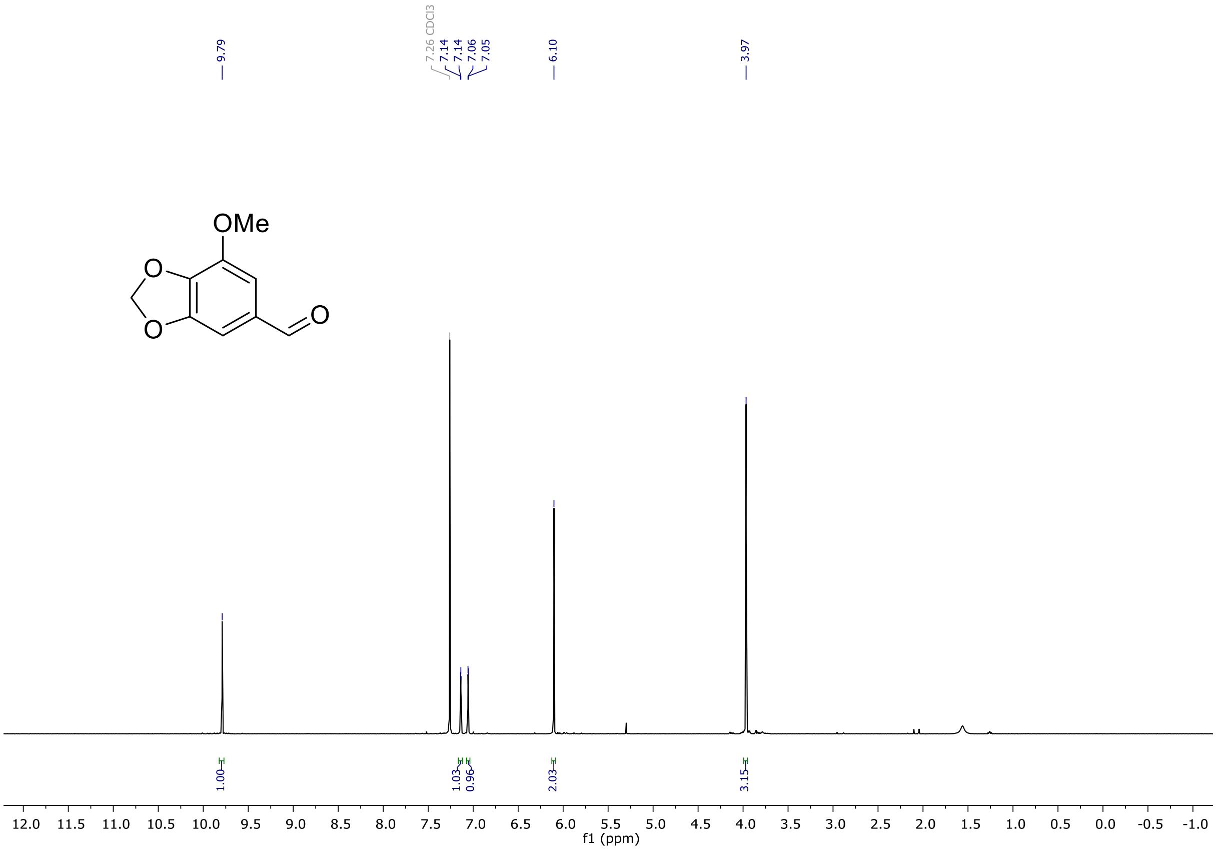

^{1}H NMR (400 MHz, $CDCl_3$) of phenol **SI-33**:

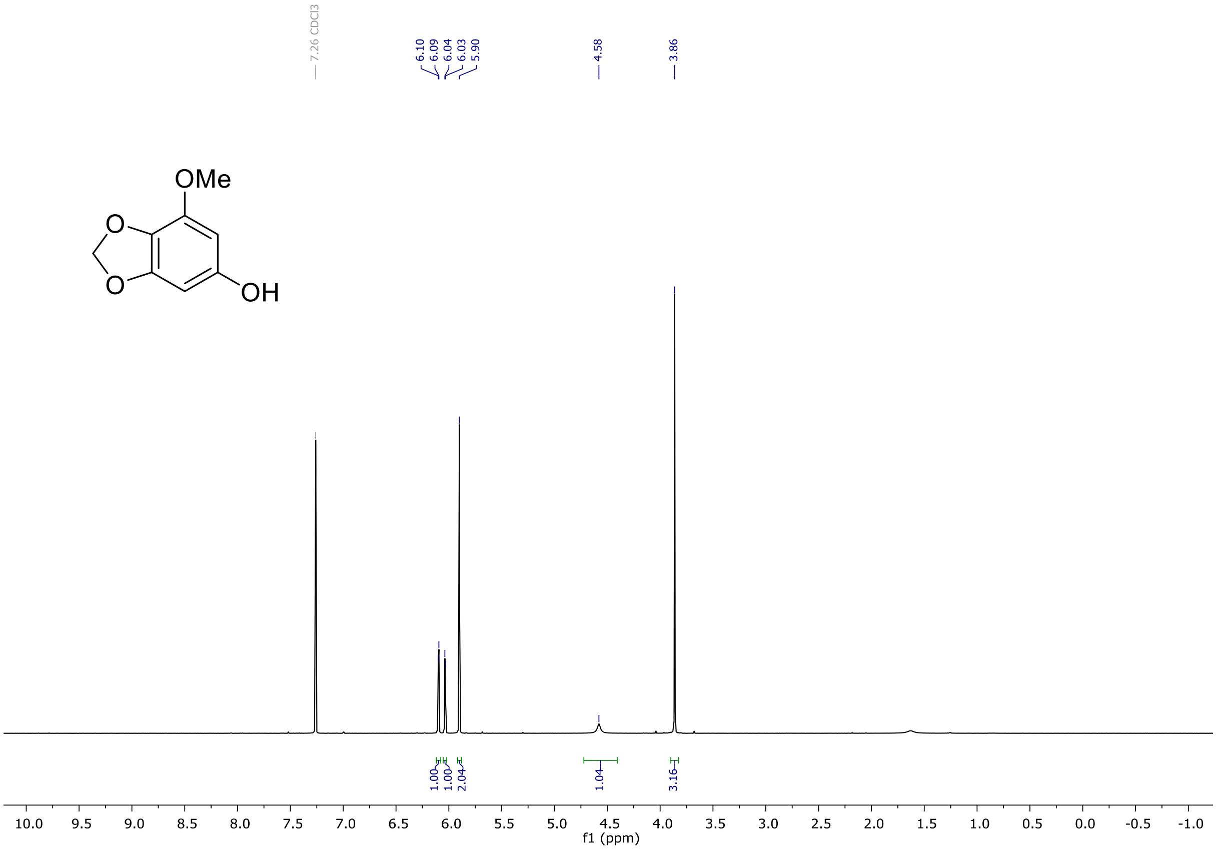

^{1}H NMR (400 MHz, $CDCl_3$) of benzodioxole **SI-34**:

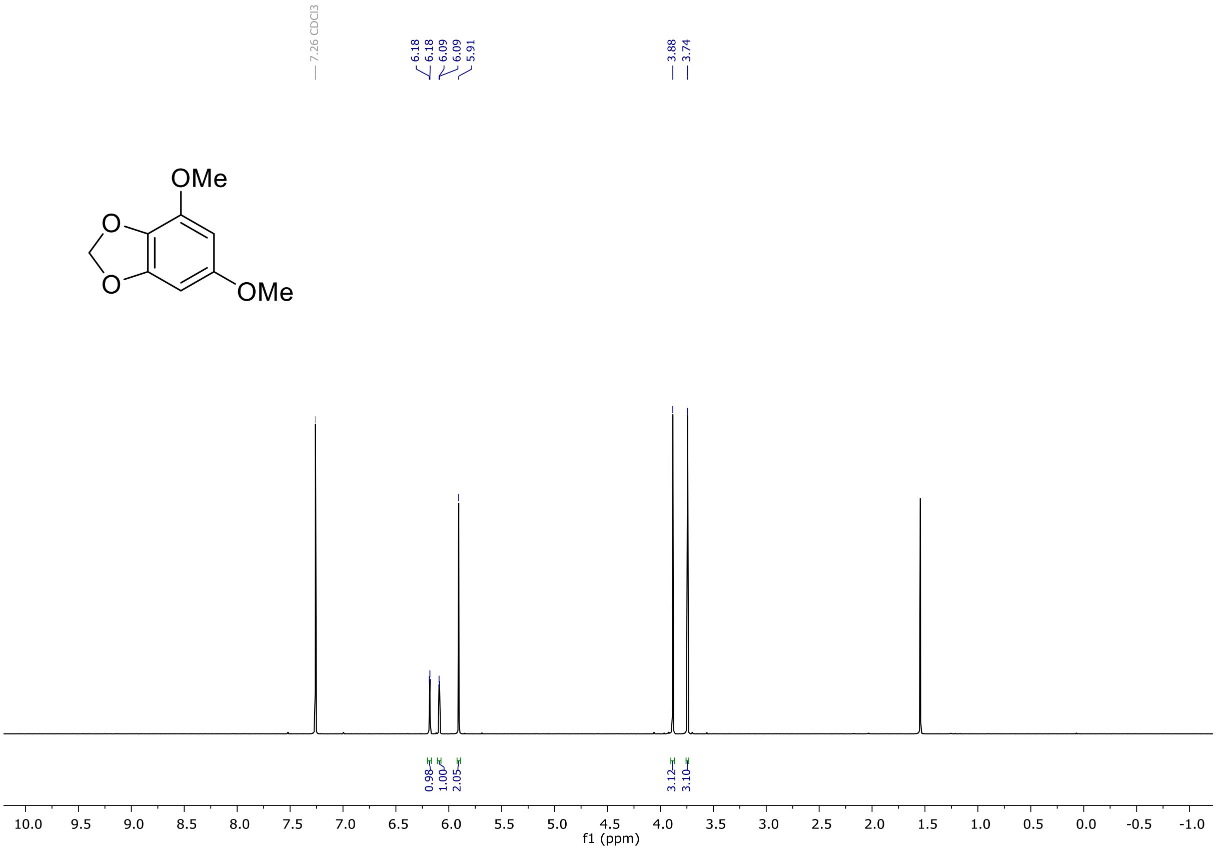

^{1}H NMR (400 MHz, $CDCl_3$) of aryl bromide **SI-35**:

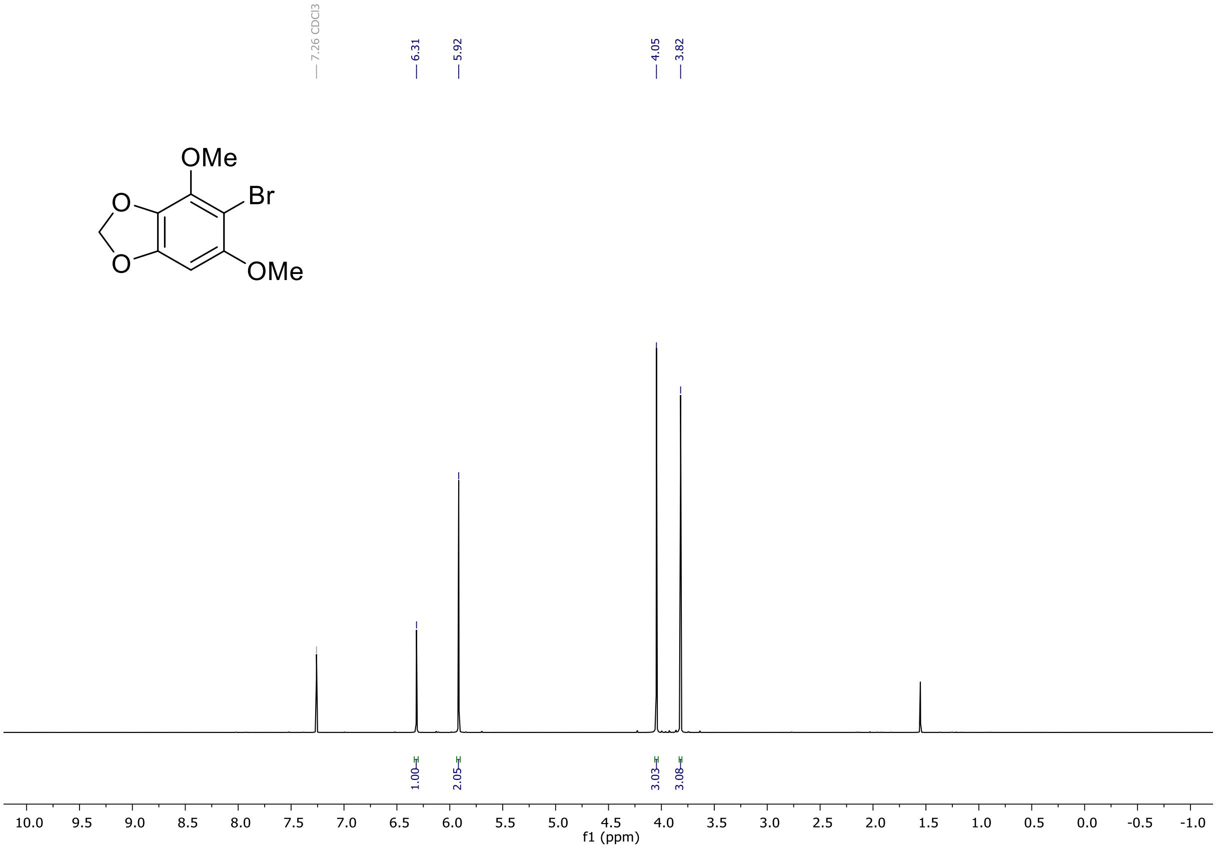

^{1}H NMR (400 MHz, $CDCl_3$) of phenol **60**:

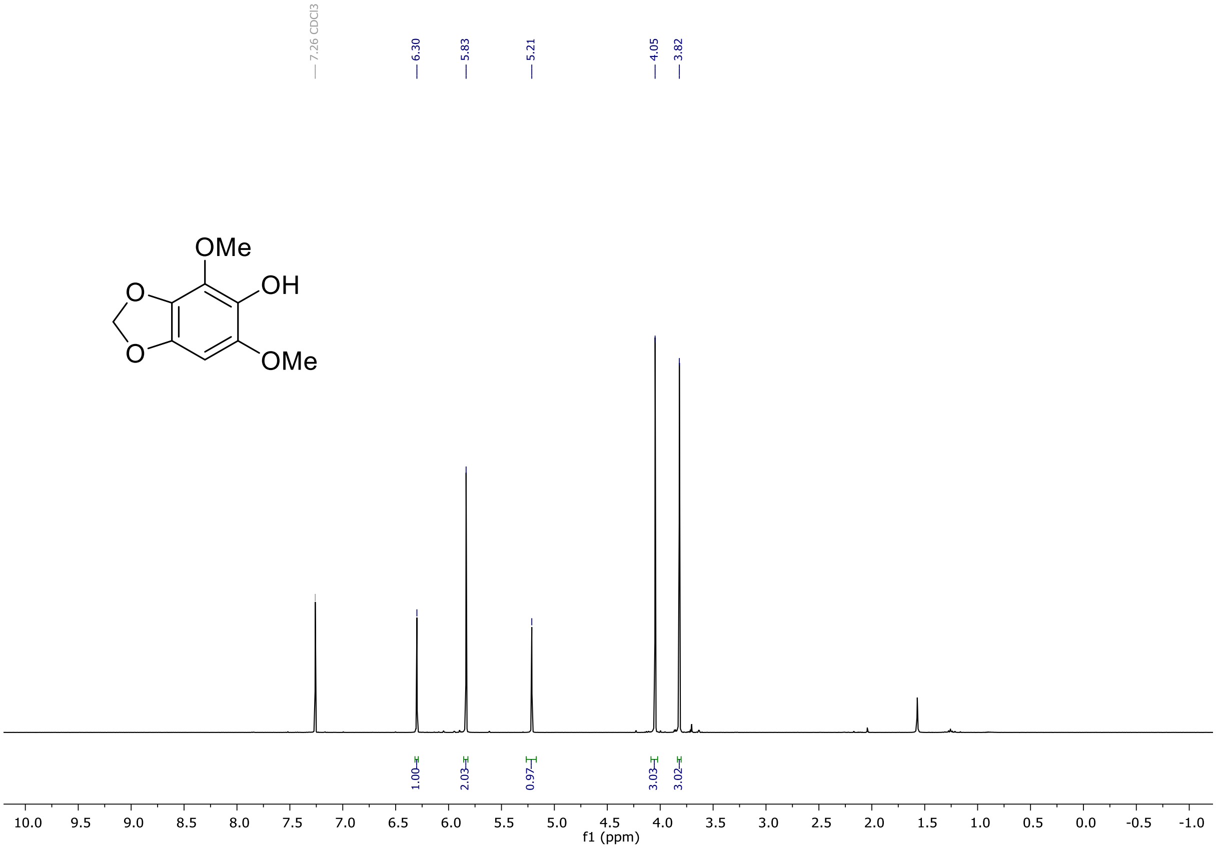

^{1}H NMR (400 MHz, $CDCl_3$) of phenol **SI-36**:

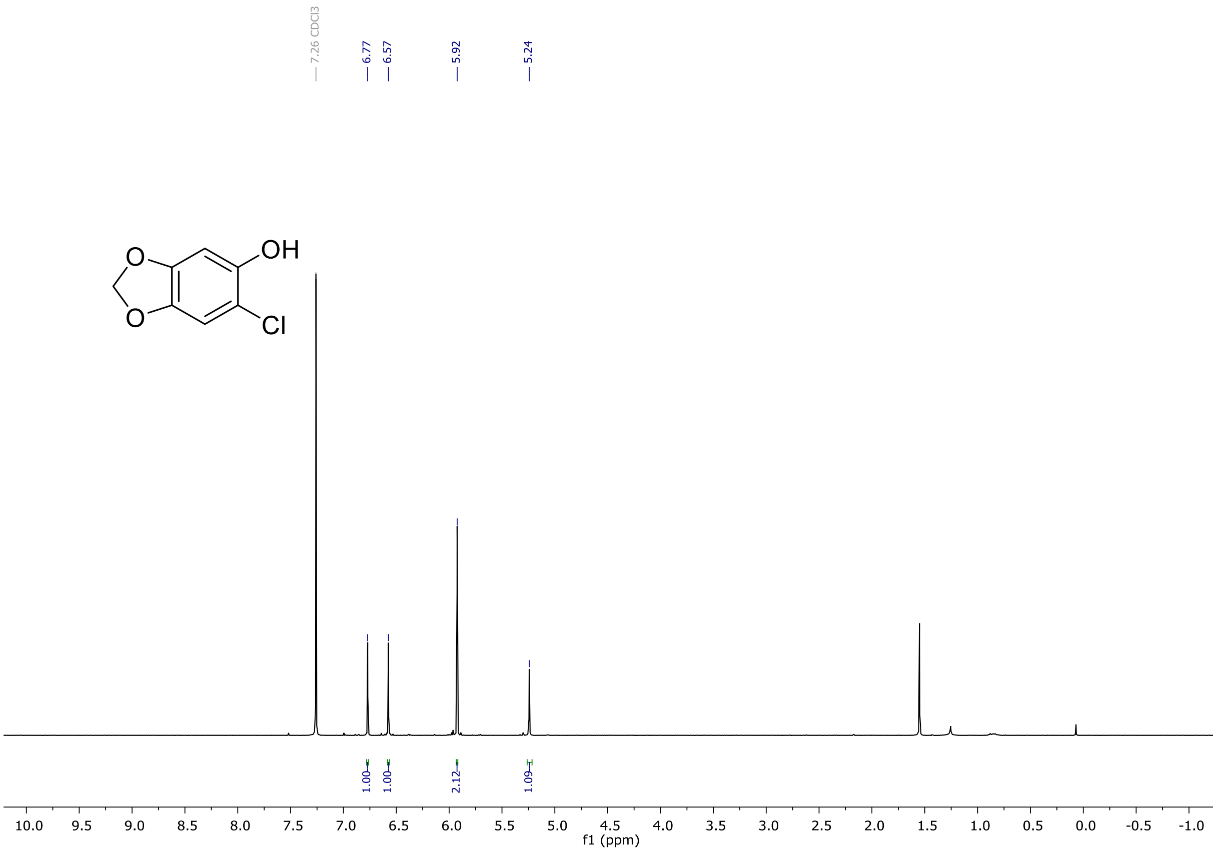

^{1}H NMR (400 MHz, $CDCl_3$) of ether **SI-37**:

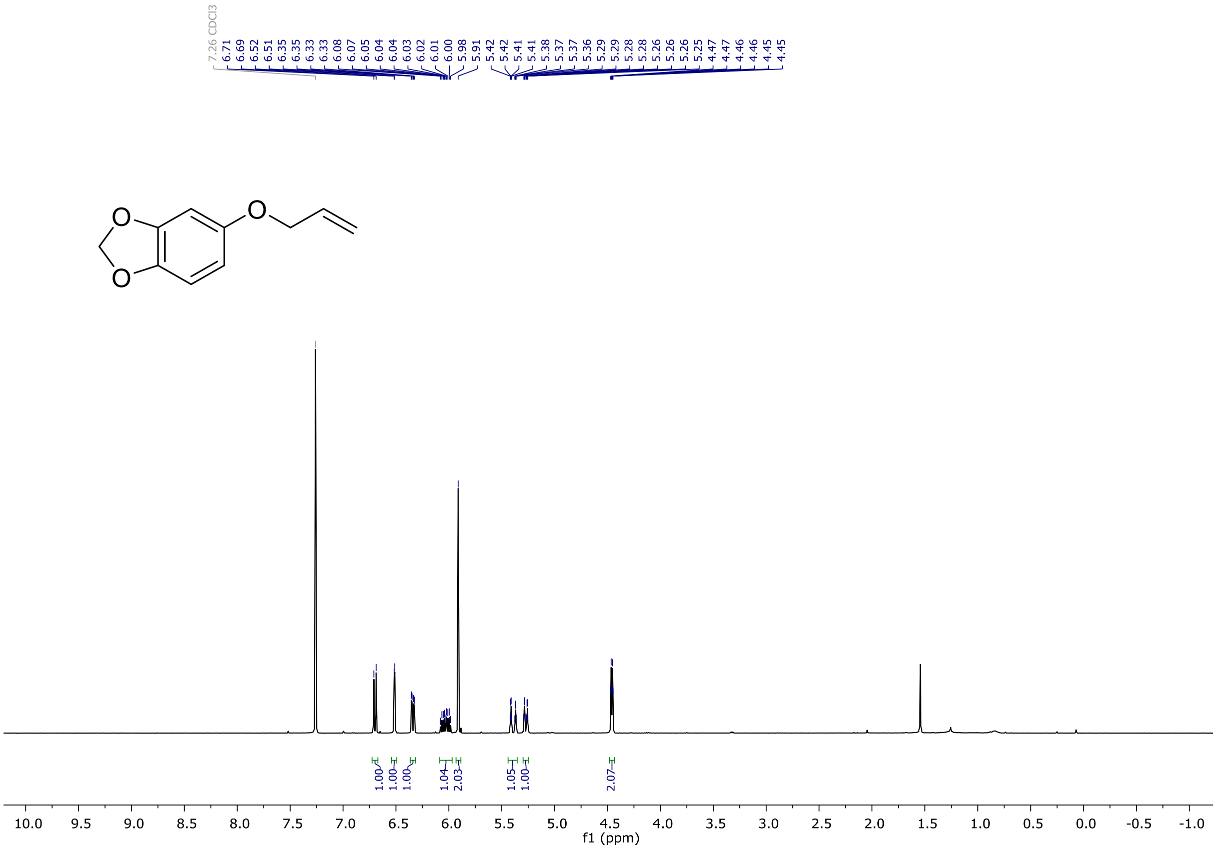

^{1}H NMR (400 MHz, $CDCl_3$) of phenol **SI-38**:

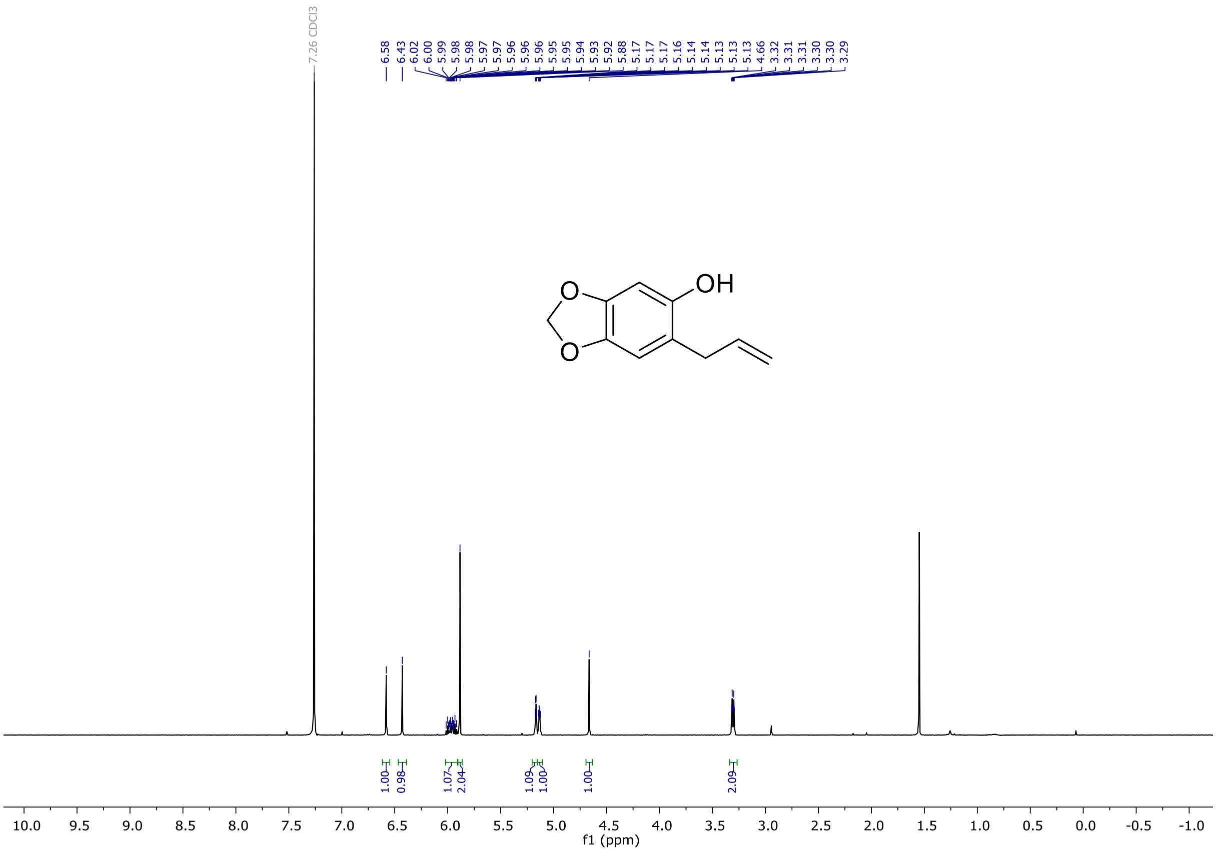

^{1}H NMR (400 MHz, $CDCl_3$) of phenol **SI-39**:

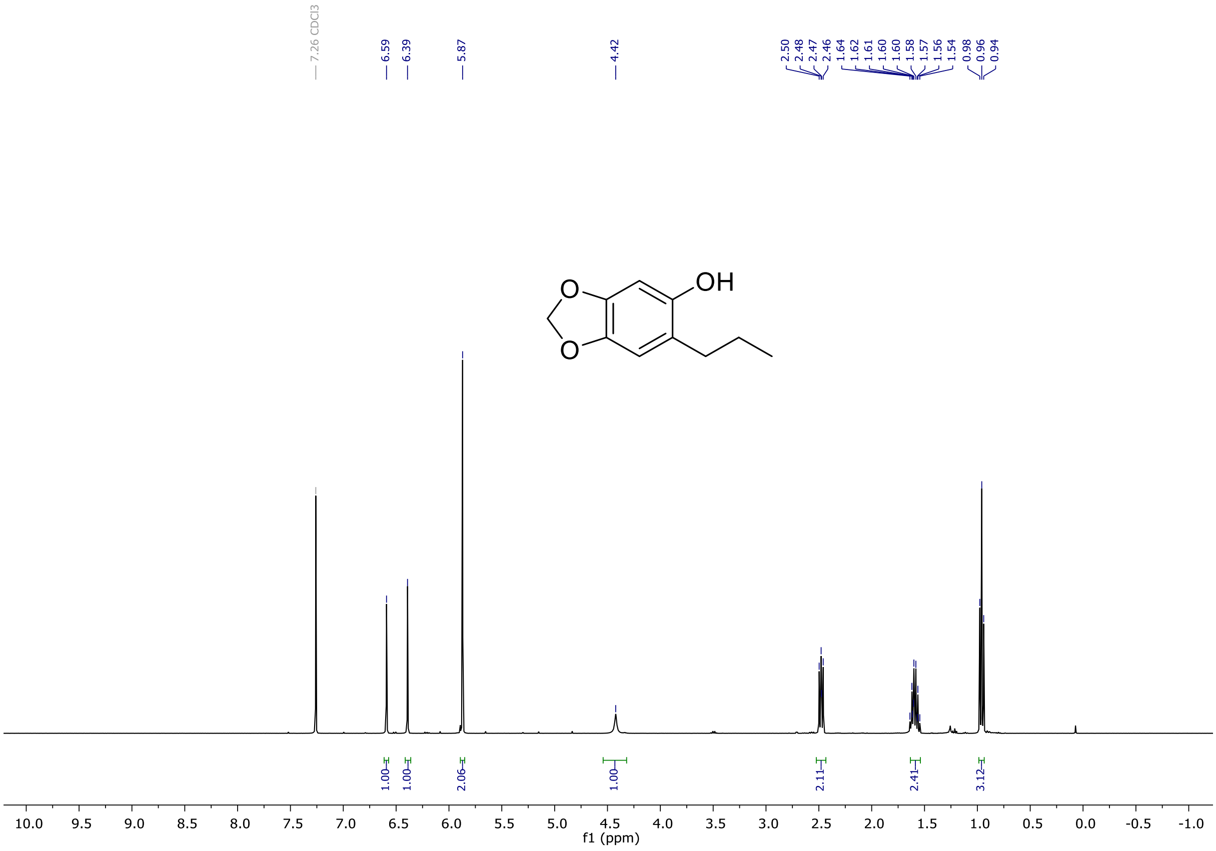

^{1}H NMR (400 MHz, CDCl$_3$) of *ortho*-quinone **SI-40**:

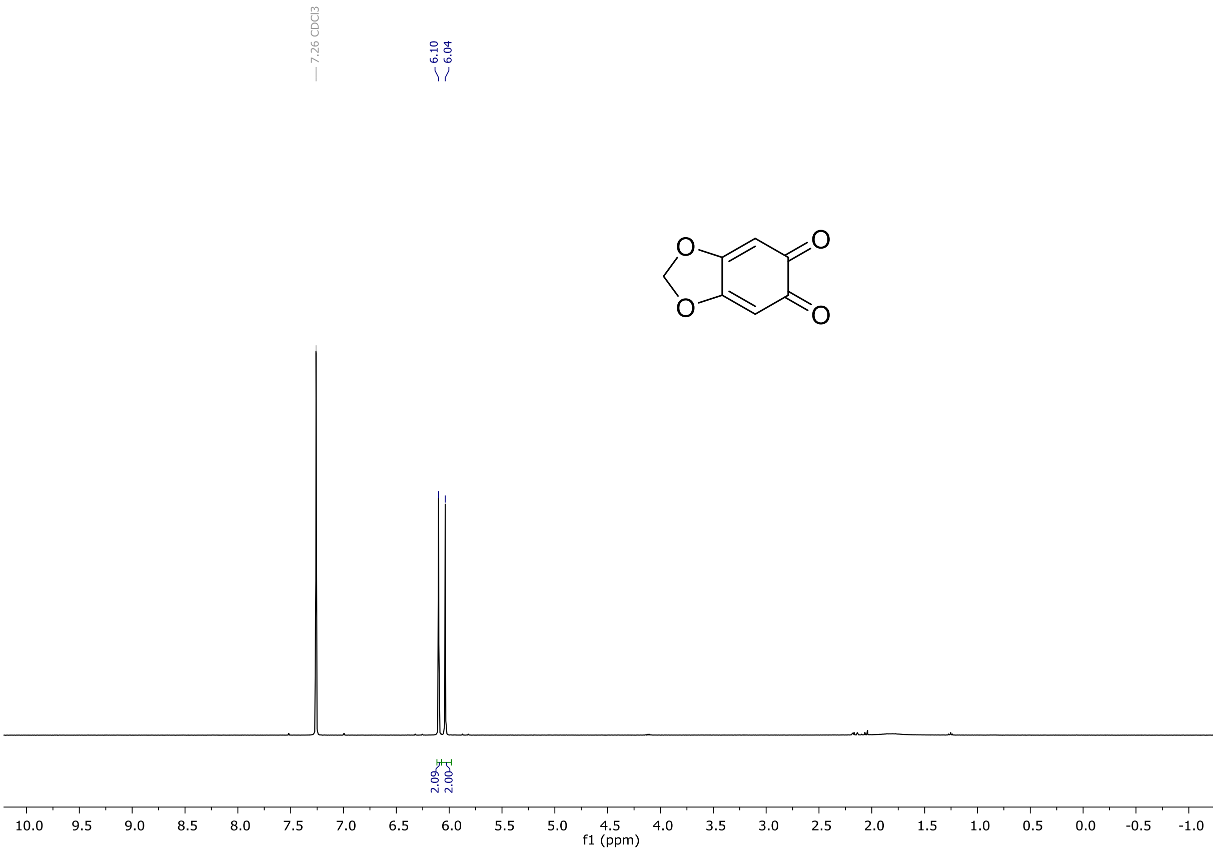

^{1}H NMR (400 MHz, CDCl$_3$) of catechol **SI-41**:

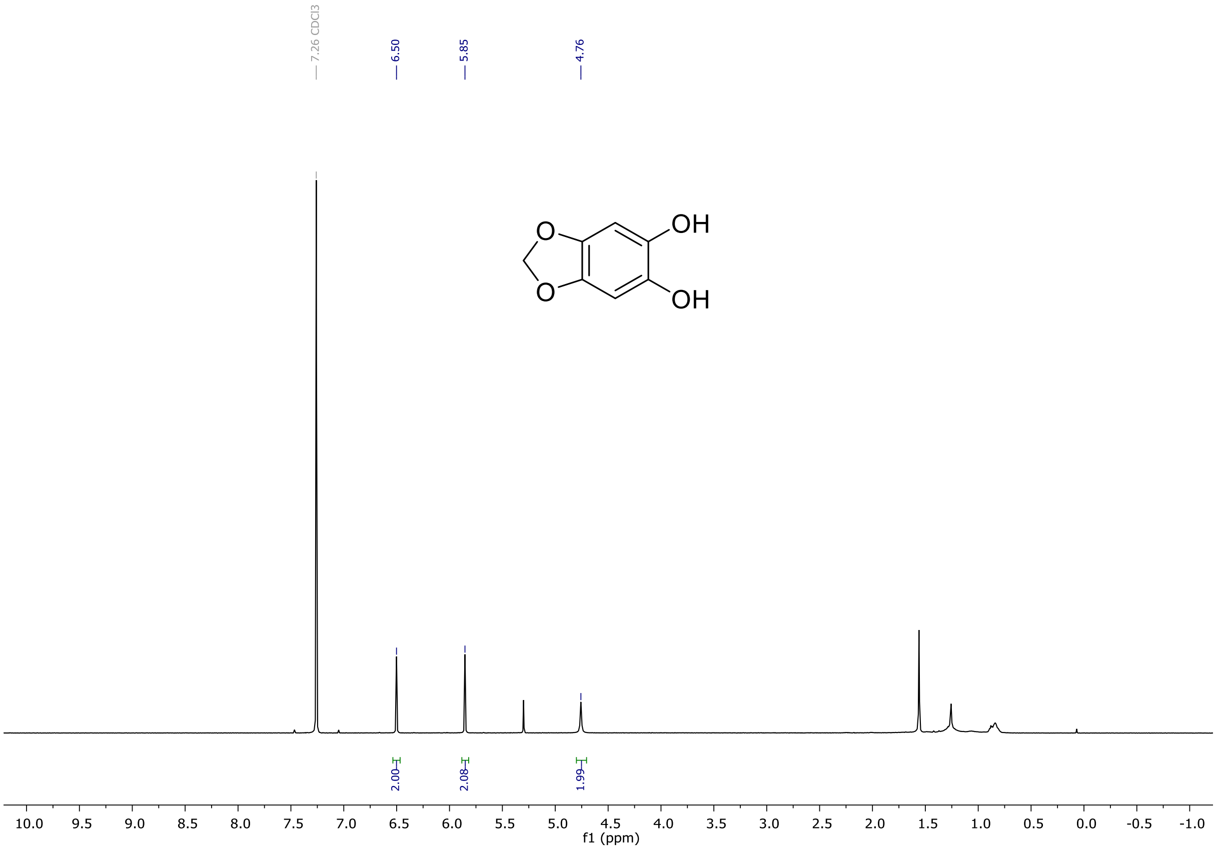

^{1}H NMR (400 MHz, $CDCl_3$) of phenol **SI-42**:

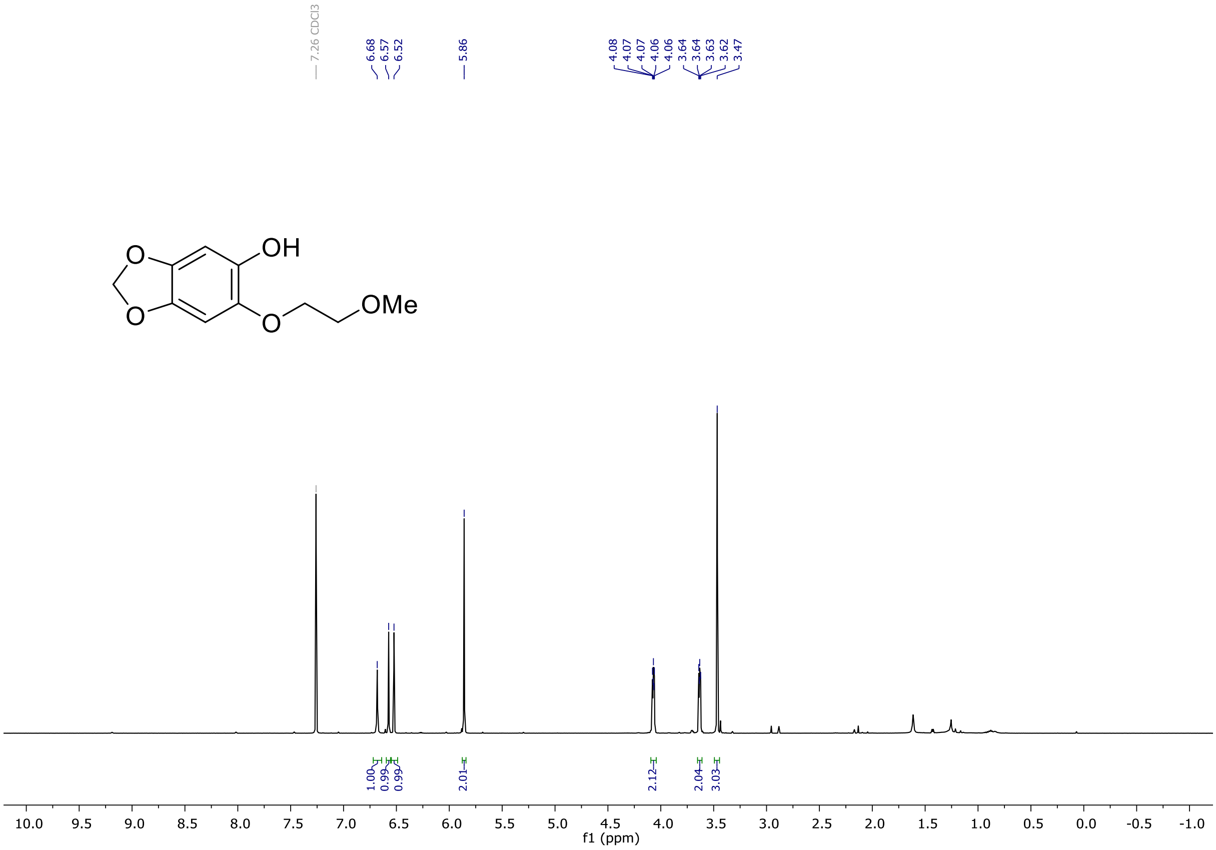

^{13}C NMR (101 MHz, $CDCl_3$) of phenol **SI-42**:

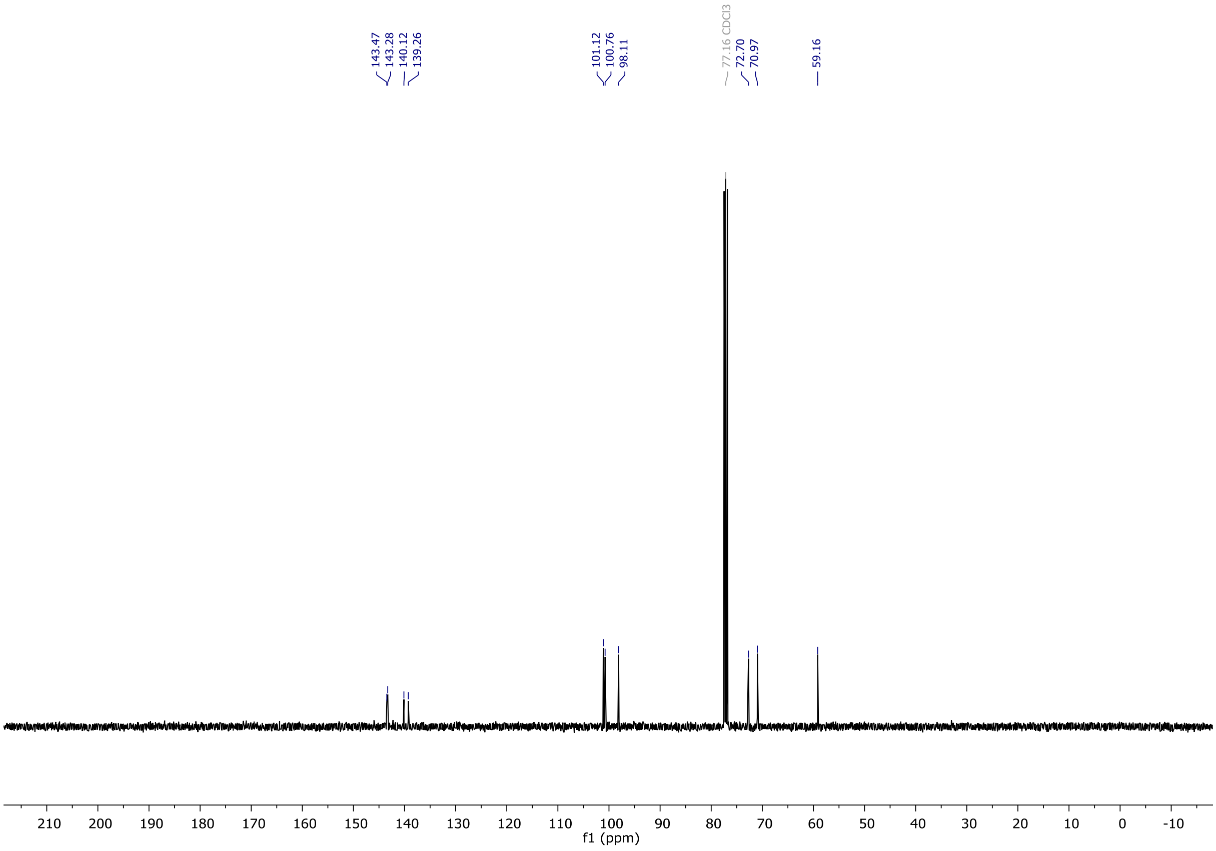

^{1}H NMR (400 MHz, $CDCl_3$) of phenol **SI-43**:

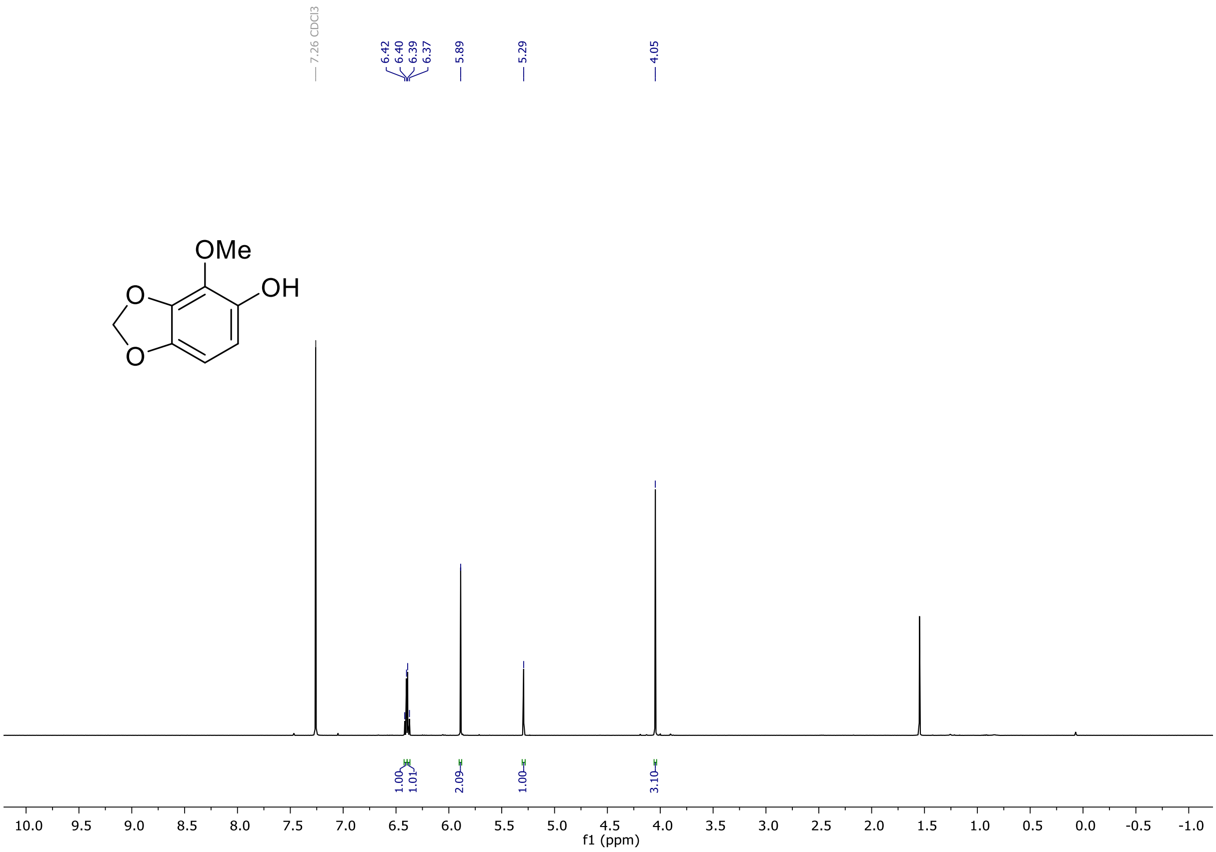

^{1}H NMR (400 MHz, $CDCl_3$) of phenol **SI-45**:

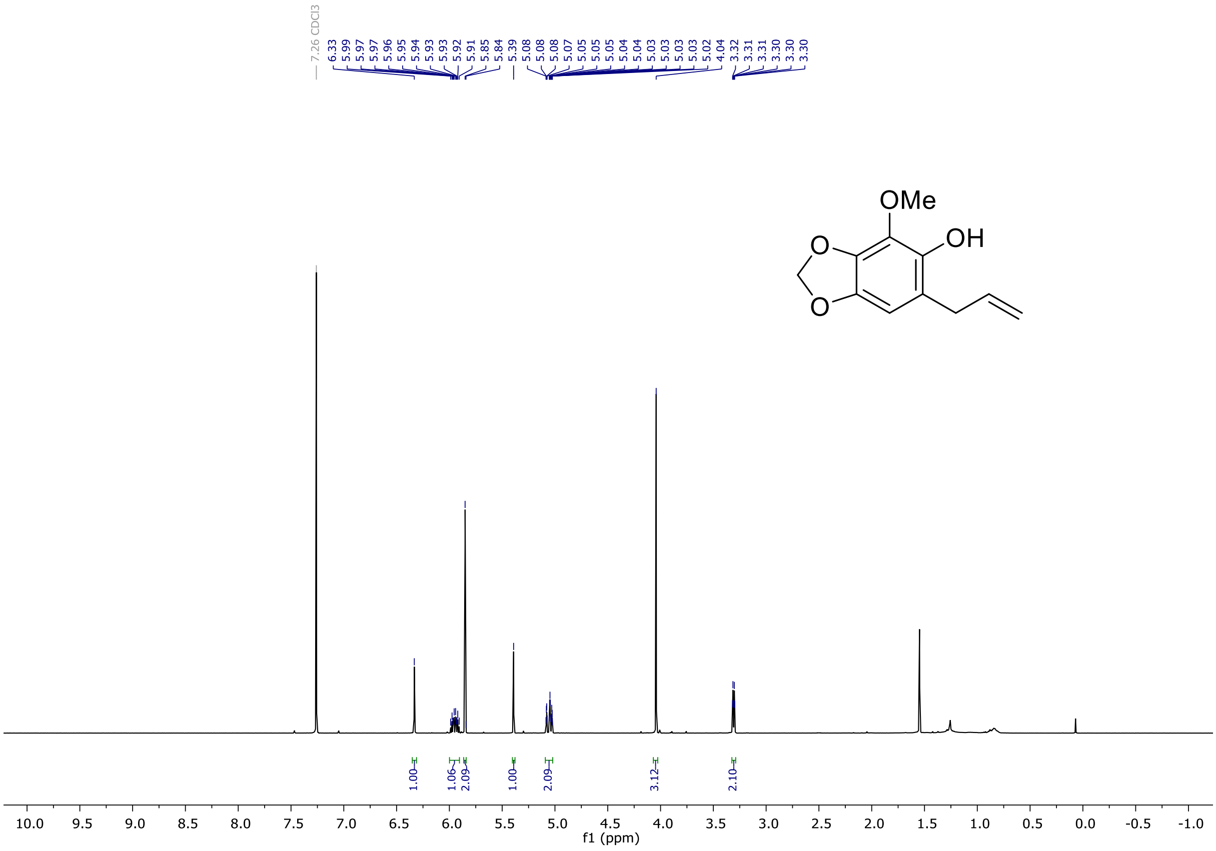

^{1}H NMR (500 MHz, $CDCl_3$) of benzodioxane **58**:

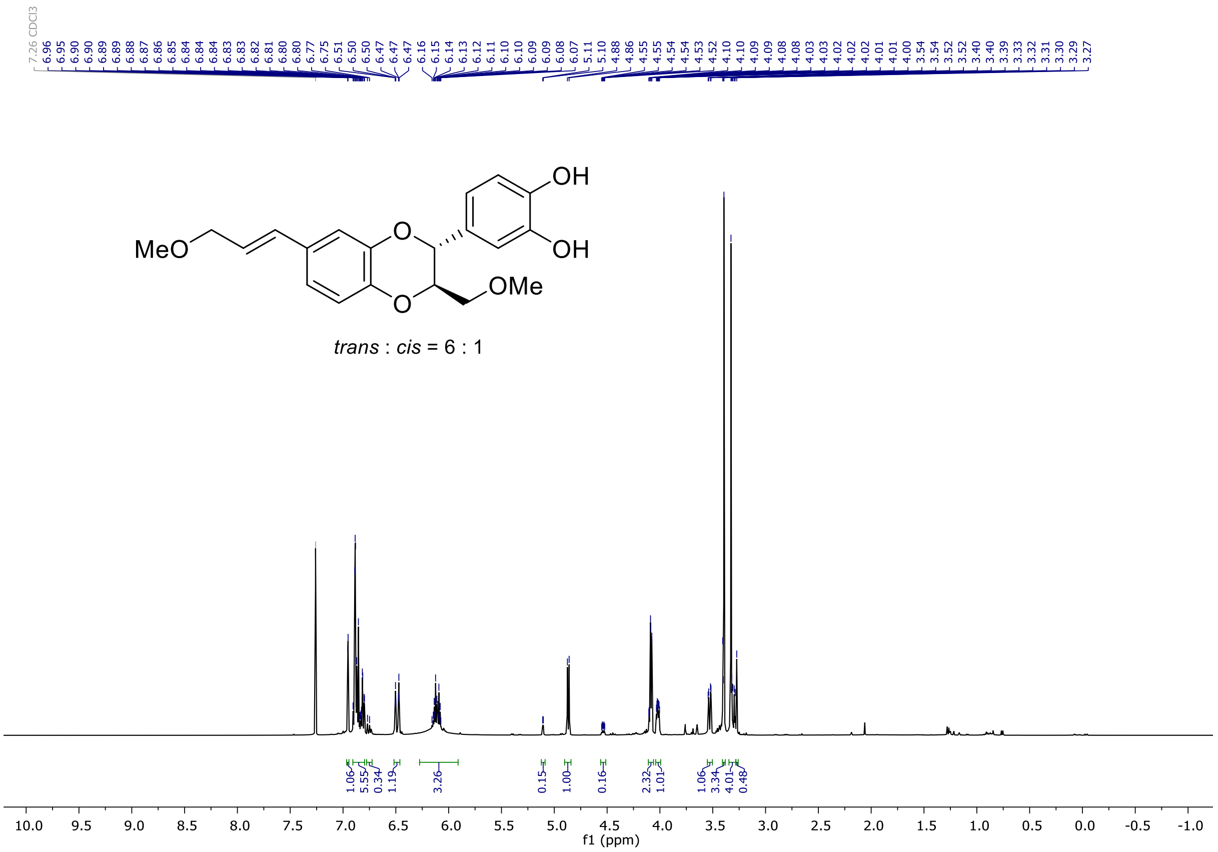

^{13}C NMR (126 MHz, $CDCl_3$) of benzodioxane **58:**

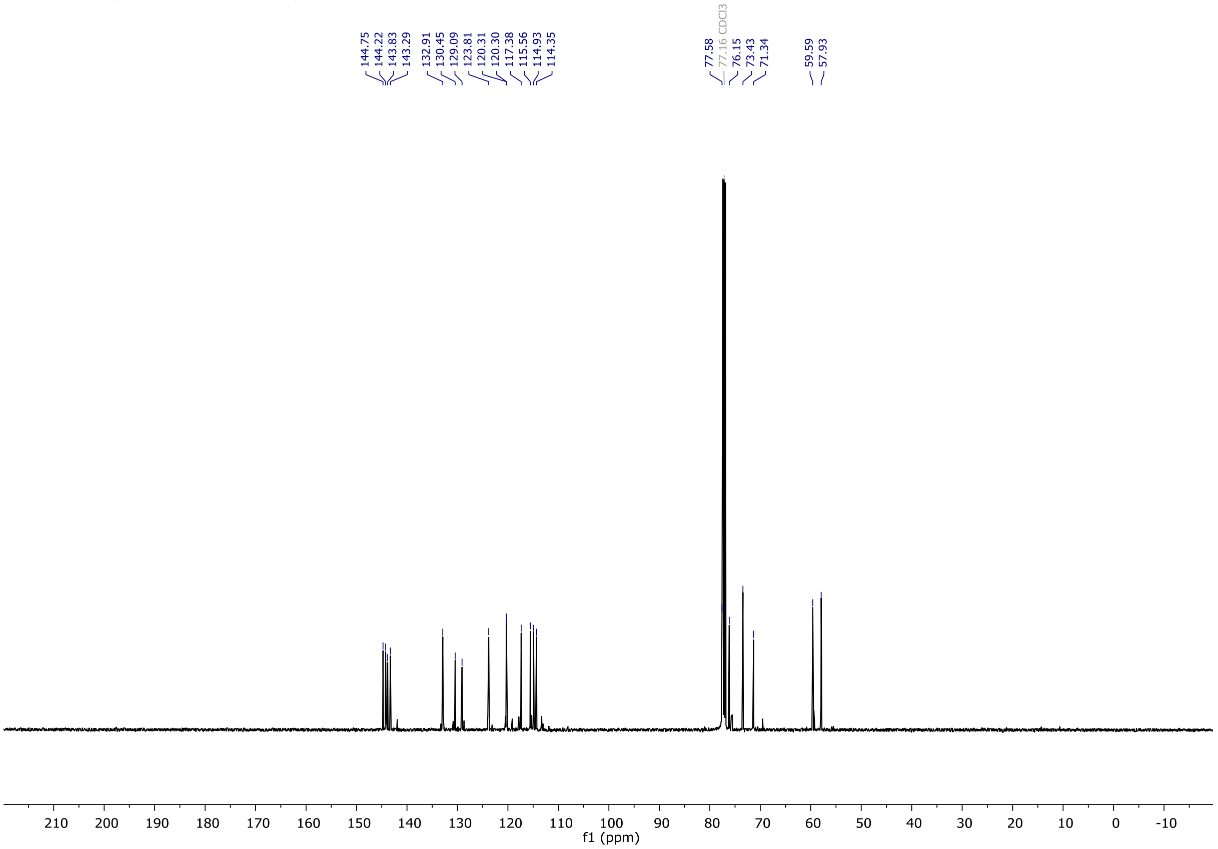

^{1}H NMR (400 MHz, CDCl$_3$) of benzodioxole **59a**:

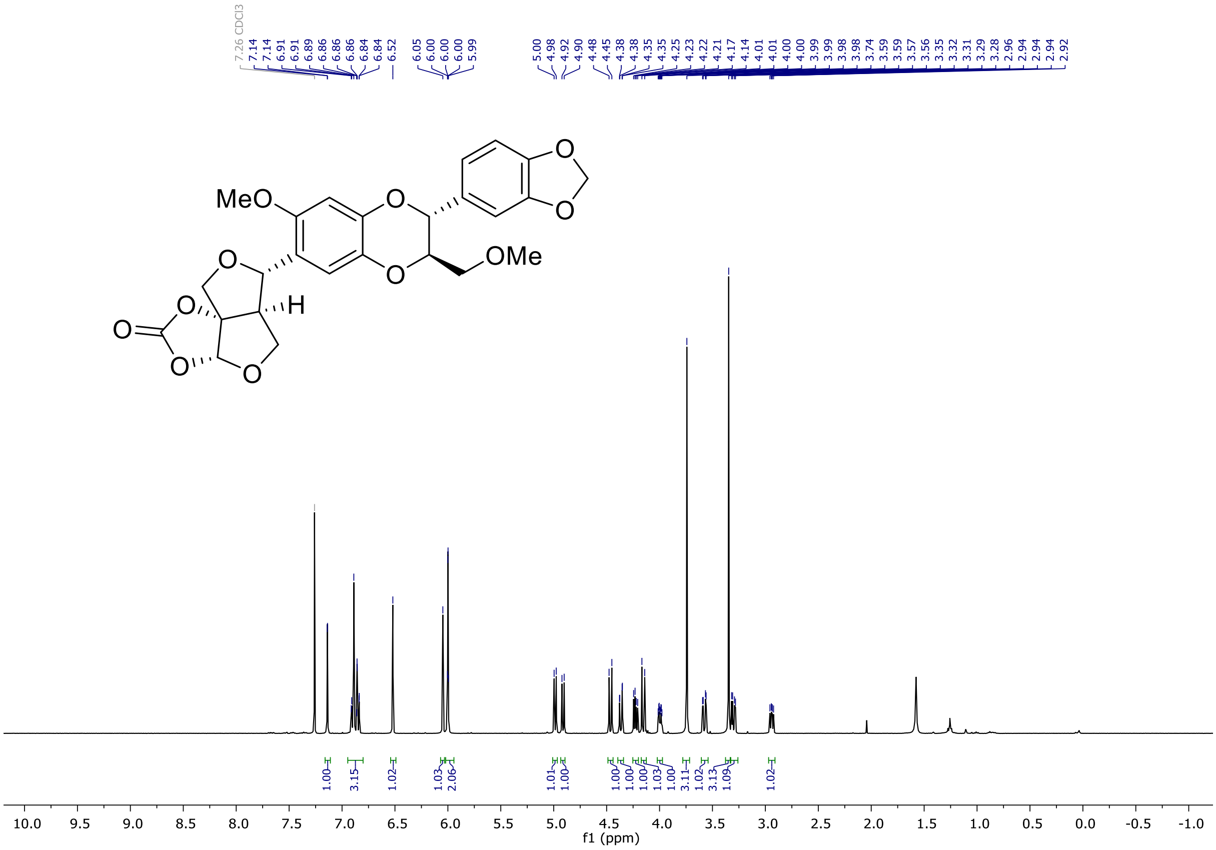

^{13}C NMR (101 MHz, CDCl$_3$) of benzodioxole **59a:**

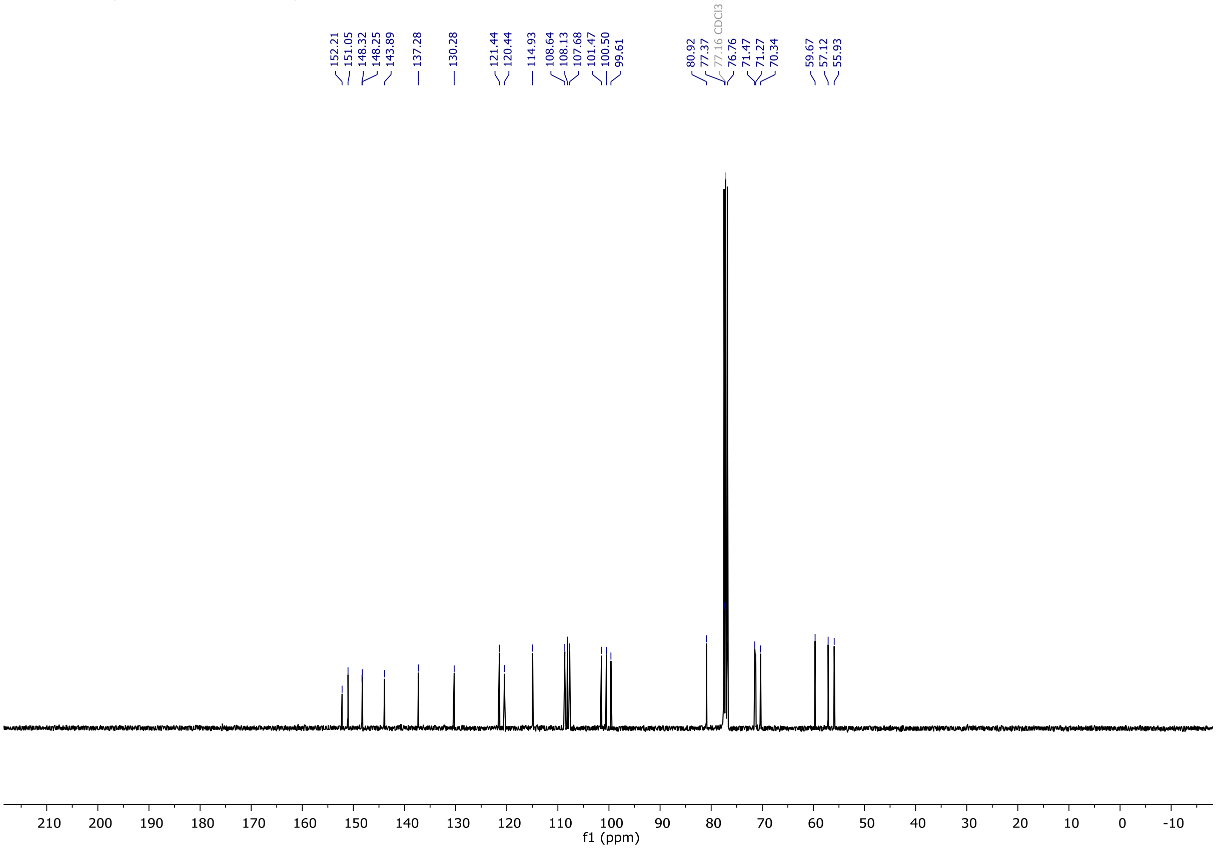

^{1}H NMR (400 MHz, $CDCl_3$) of benzodioxole **59b**:

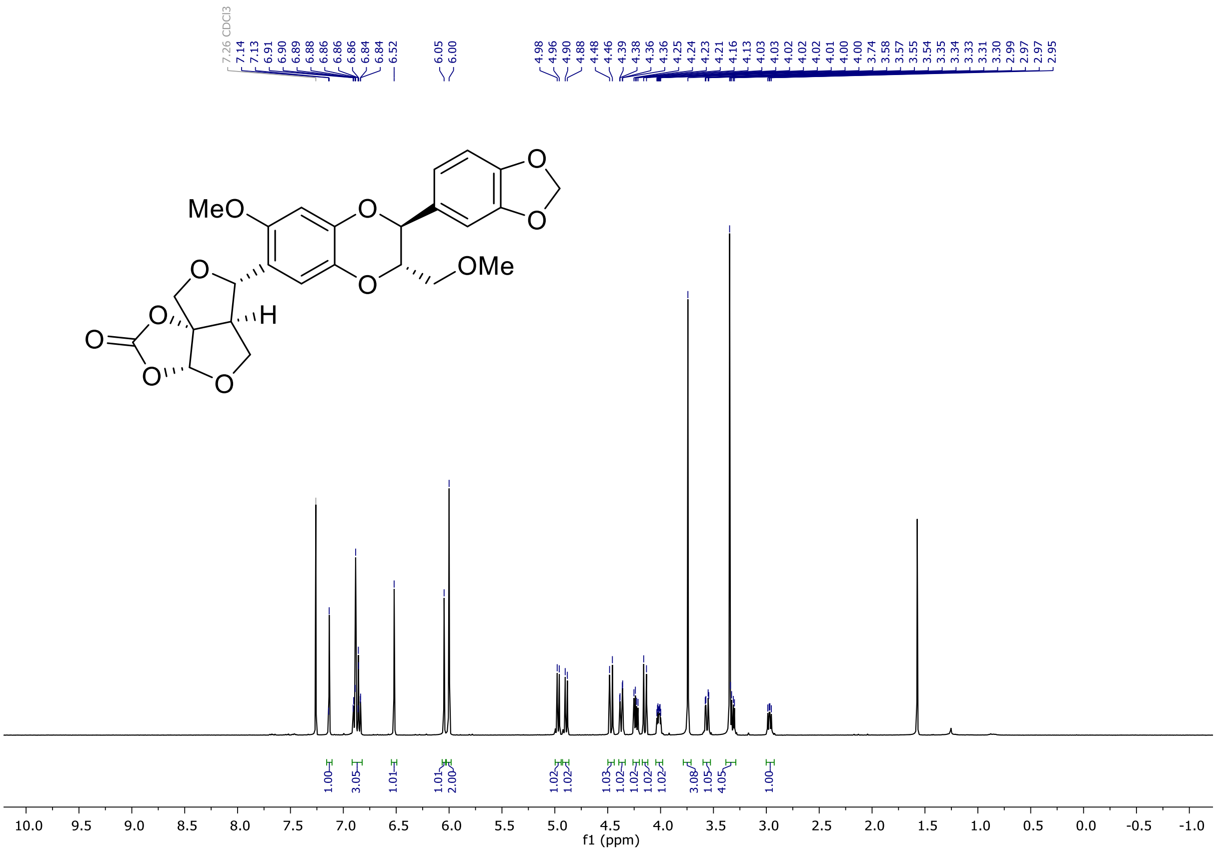

^{13}C NMR (101 MHz, $CDCl_3$) of benzodioxole **59b:**

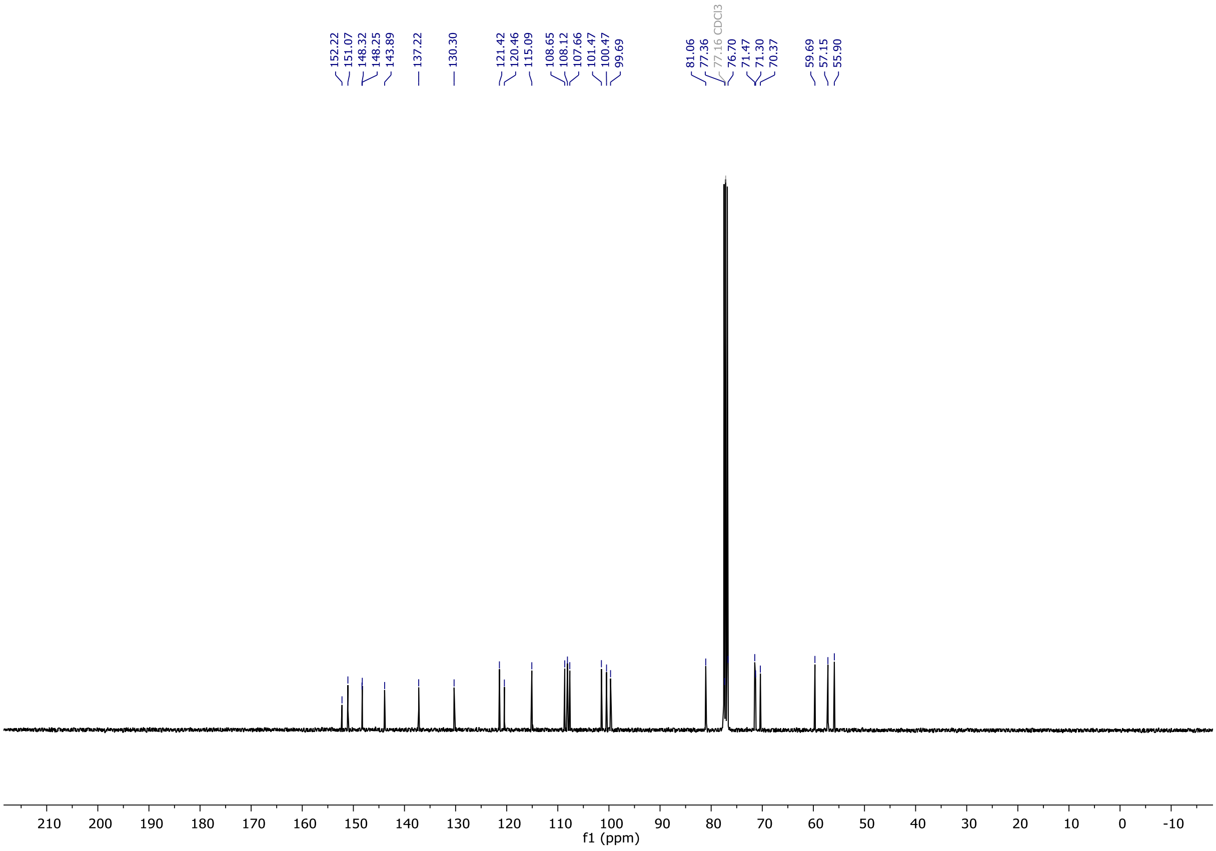

^{1}H NMR (400 MHz, $CDCl_3$) of diol **12**:

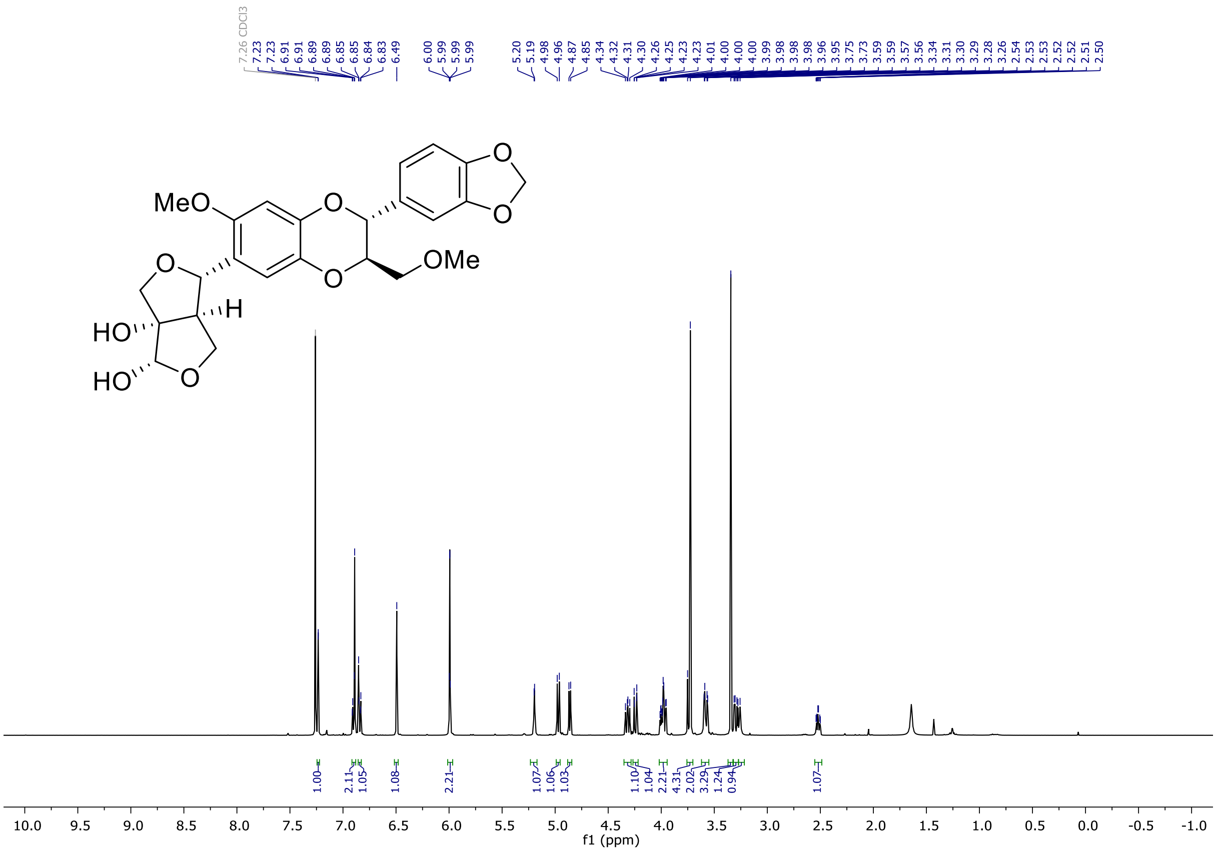

^{13}C NMR (101 MHz, $CDCl_3$) of diol **12:**

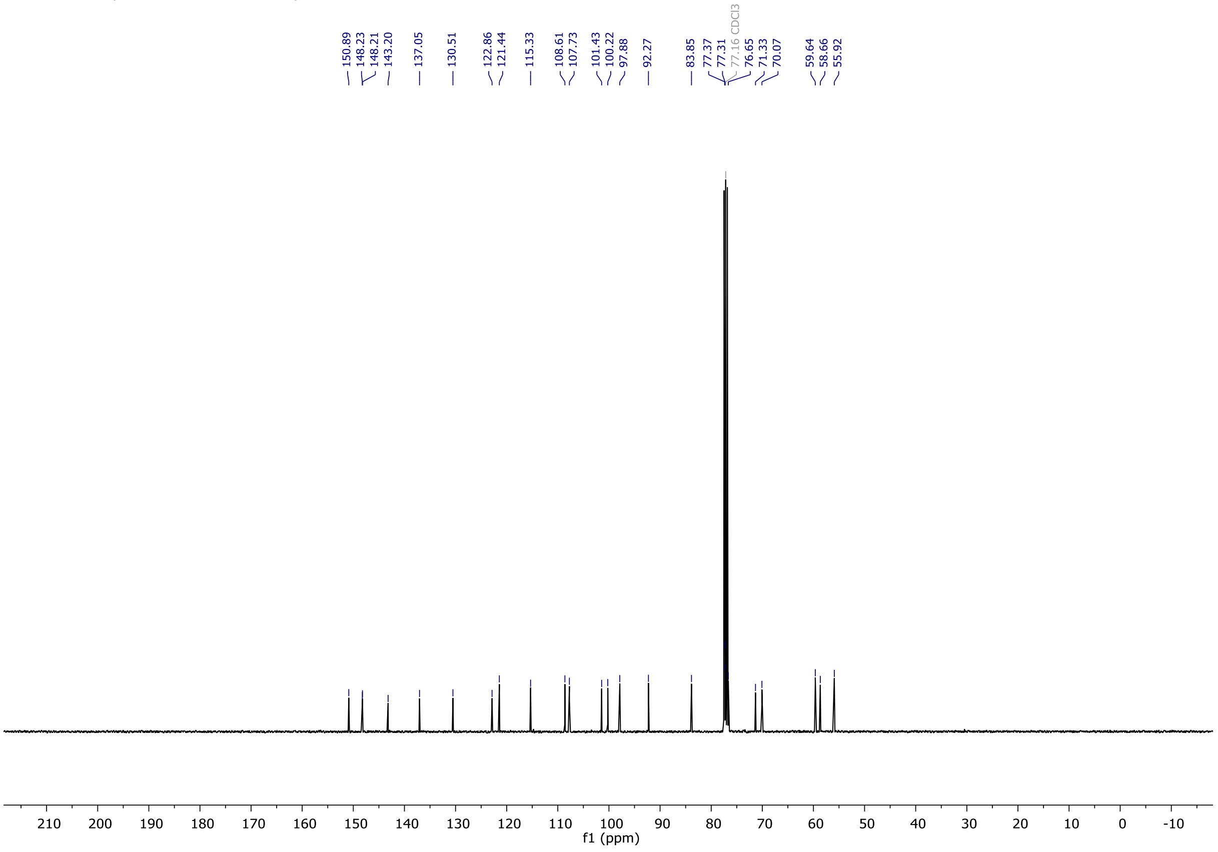

^{1}H NMR (400 MHz, $CDCl_3$) of diol **SI-47**:

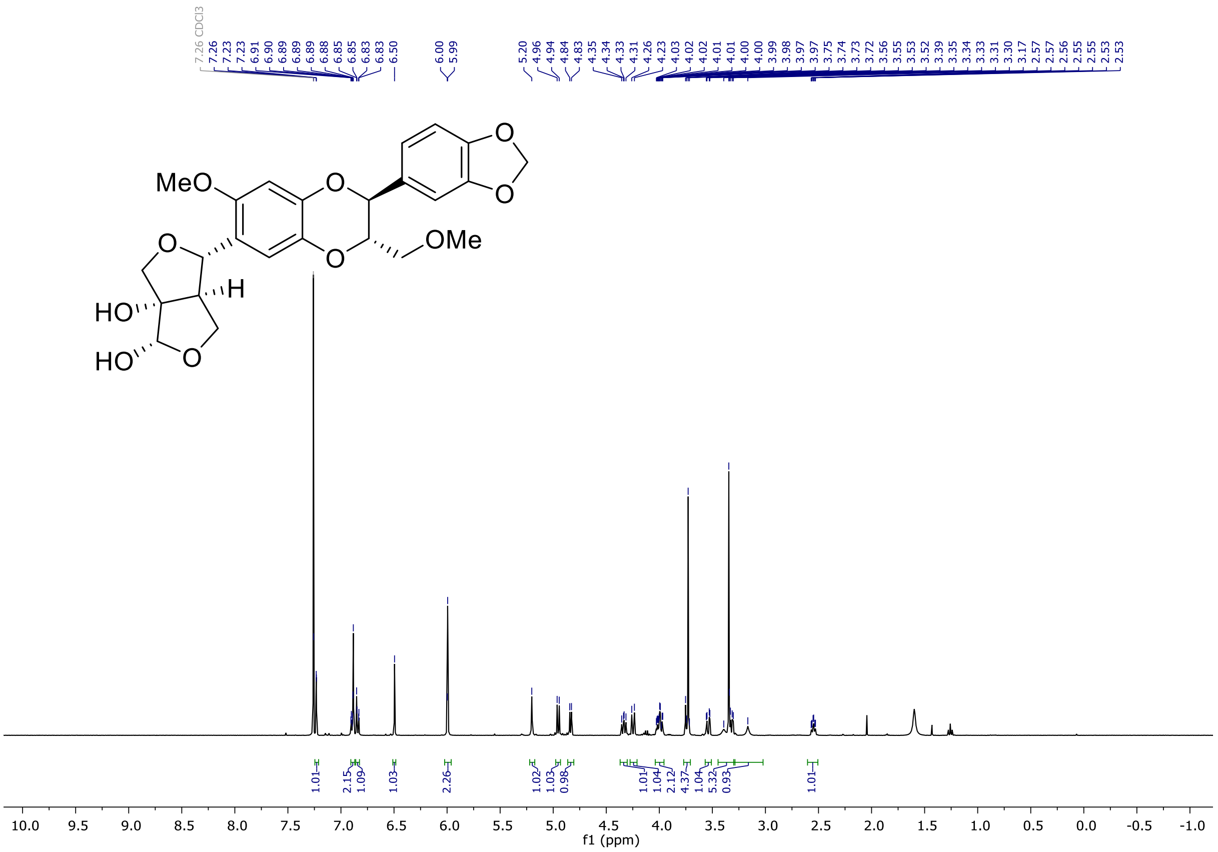

^{13}C NMR (101 MHz, $CDCl_3$) of diol **SI-47:**

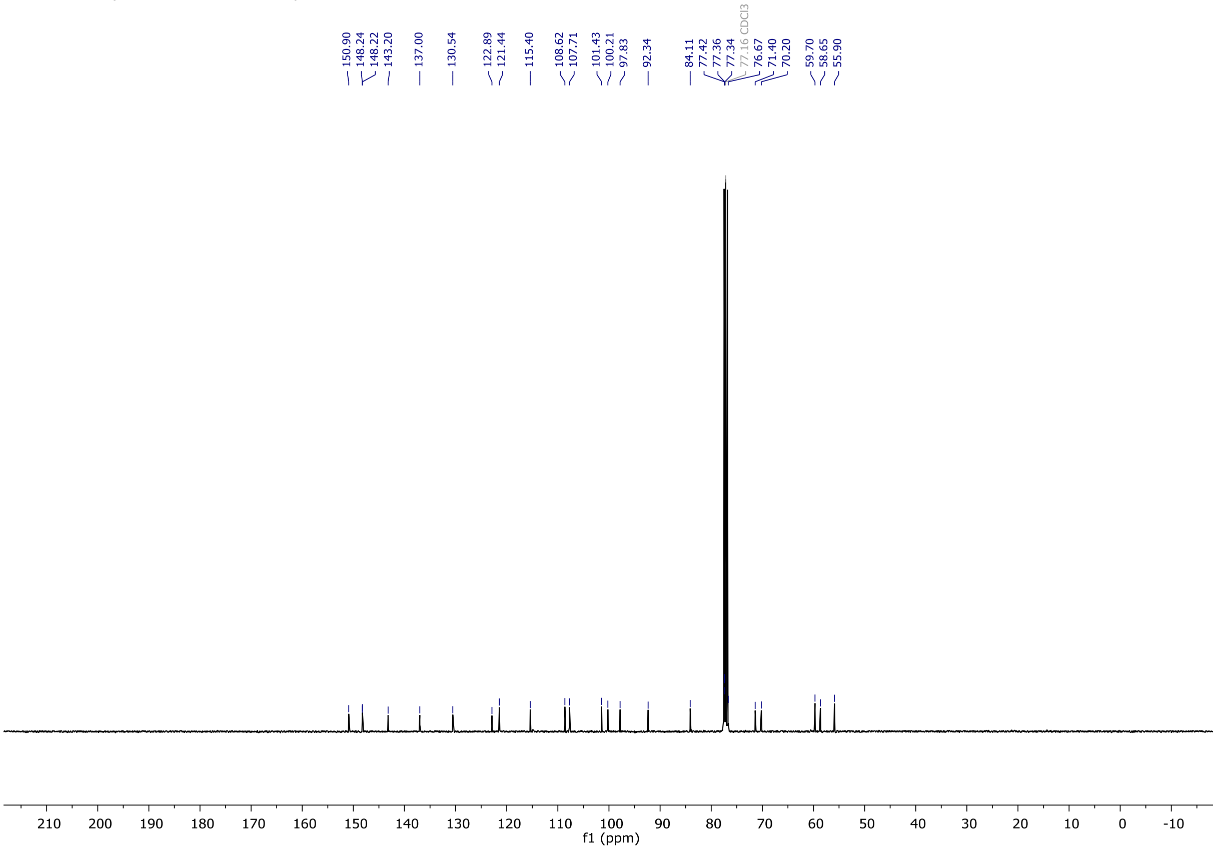

^{1}H NMR (400 MHz, $CDCl_3$) of haedoxan A (**1a**):

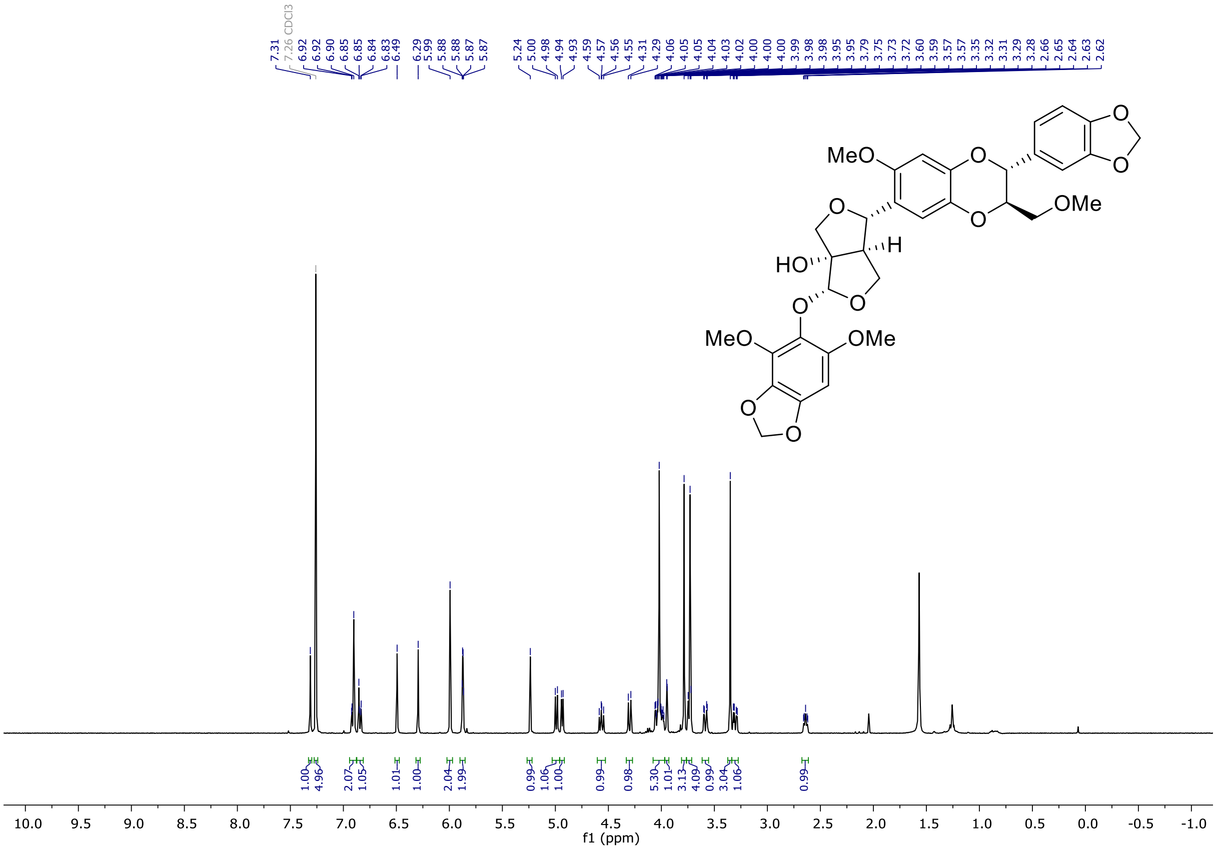

^{13}C NMR (101 MHz, $CDCl_3$) of haedoxan A (**1a**):

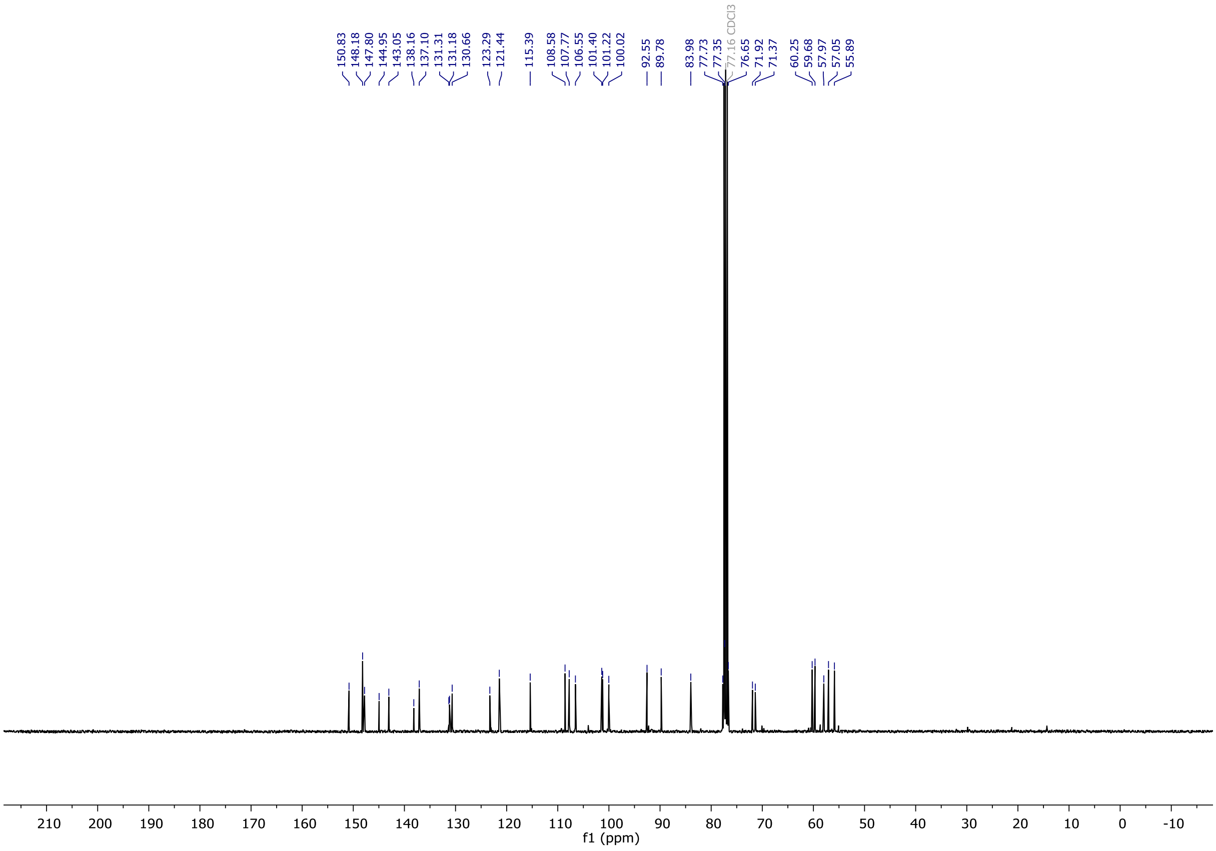

^{1}H NMR (400 MHz, $CDCl_3$) of diastereomer of haedoxan A (**SI-48**):

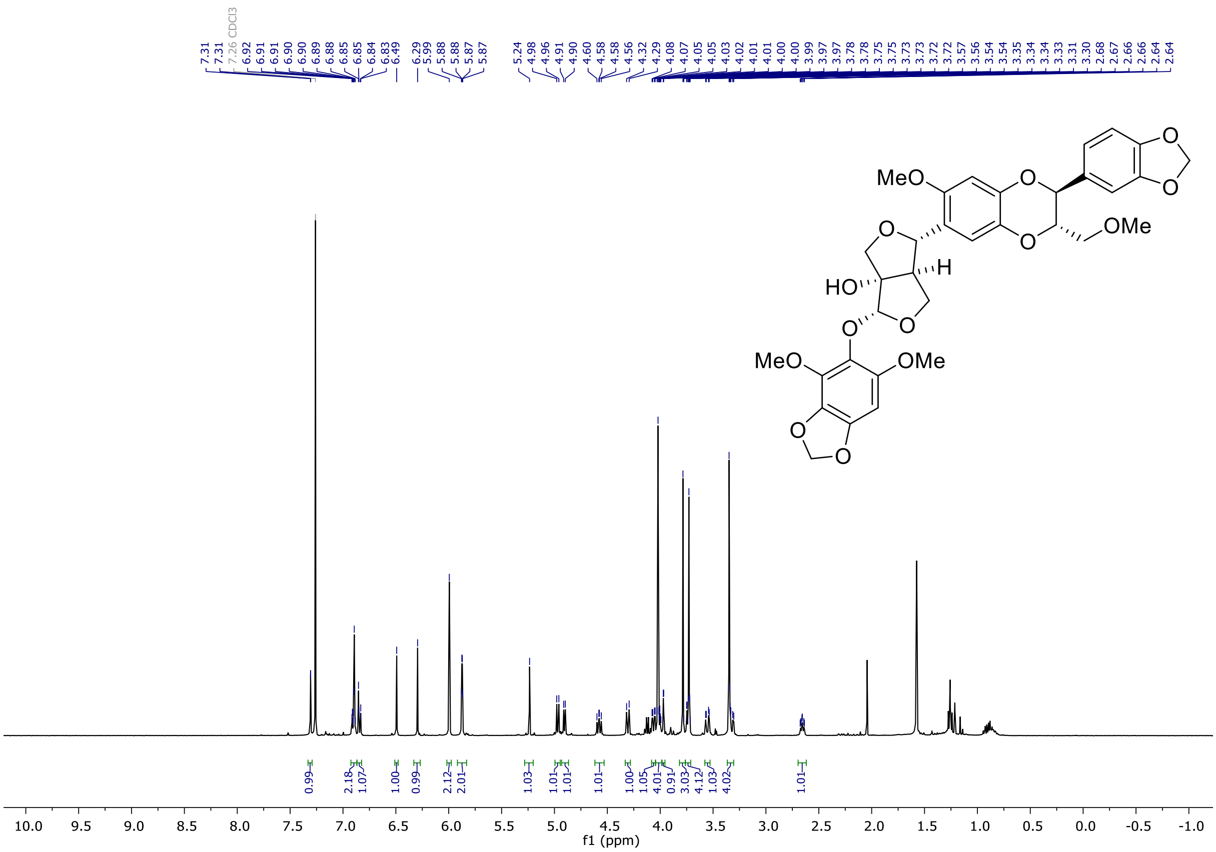

^{13}C NMR (101 MHz, $CDCl_3$) of diastereomer of haedoxan A (**SI-48**):

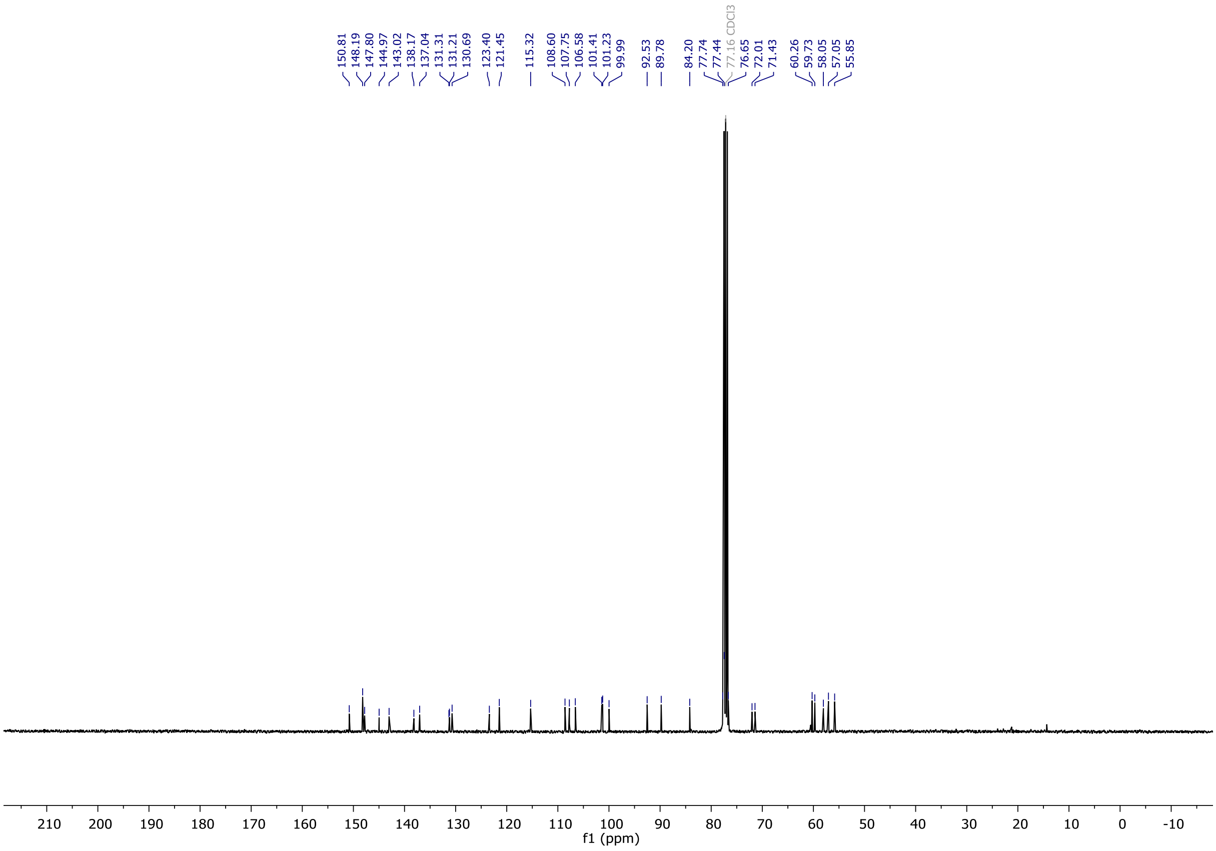

^{1}H NMR (400 MHz, $CDCl_3$) of haedoxan D (**1b**):

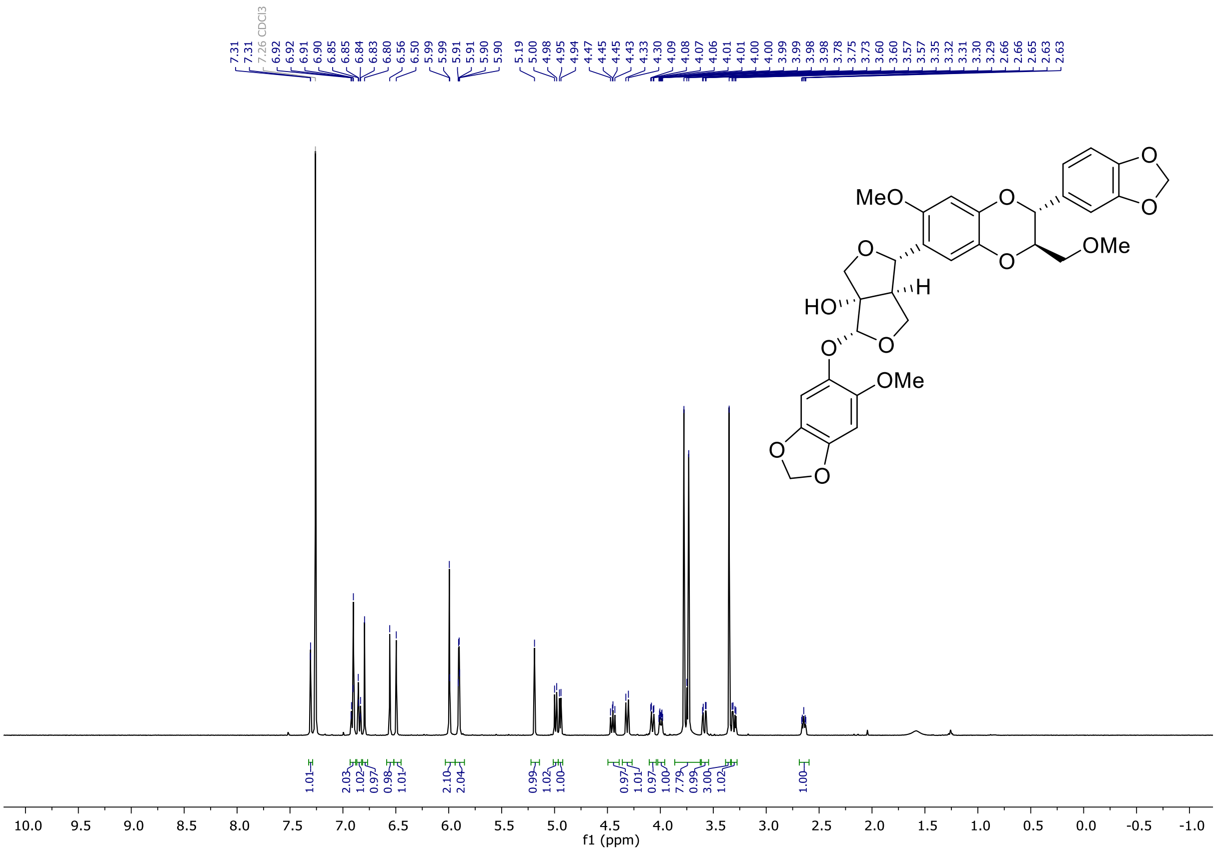

^{13}C NMR (101 MHz, $CDCl_3$) of haedoxan D (**1b**):

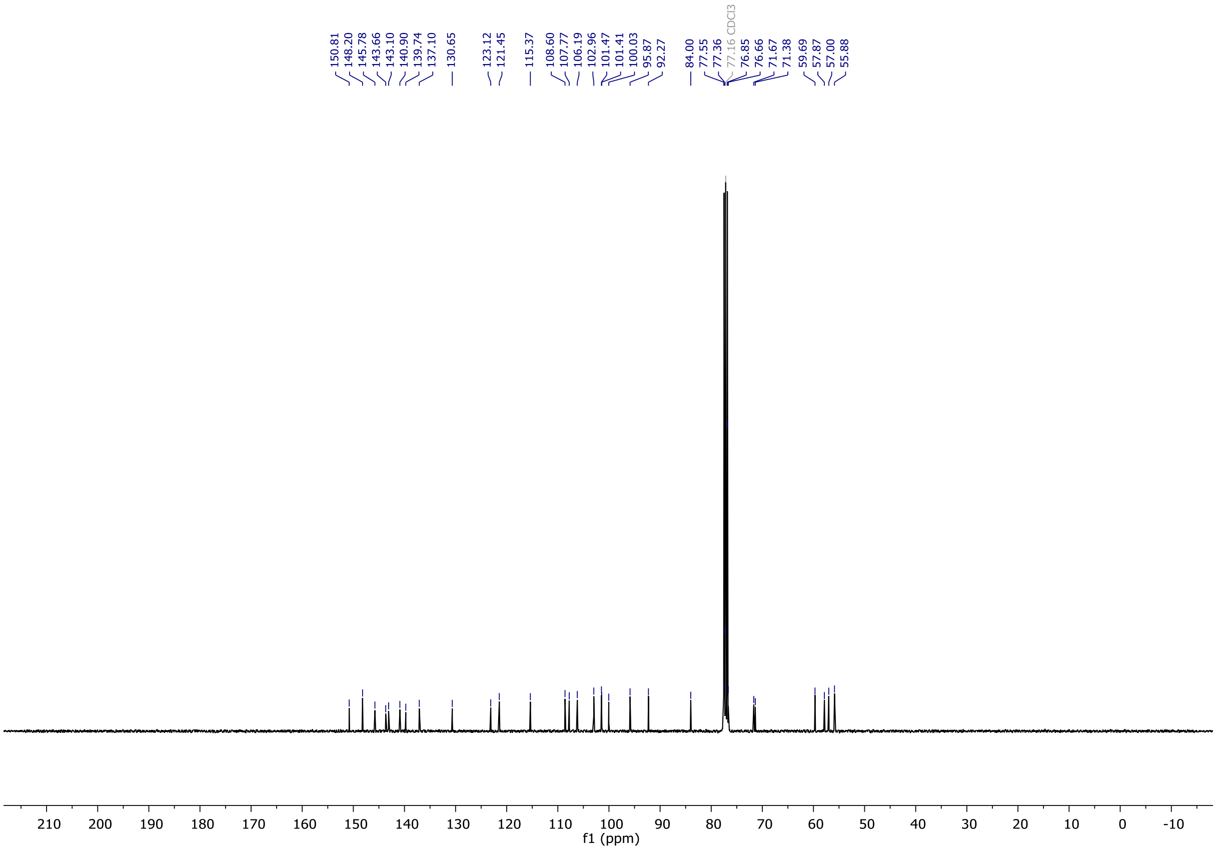

^{1}H NMR (400 MHz, $CDCl_3$) of diastereomer of haedoxan D (**SI-49**):

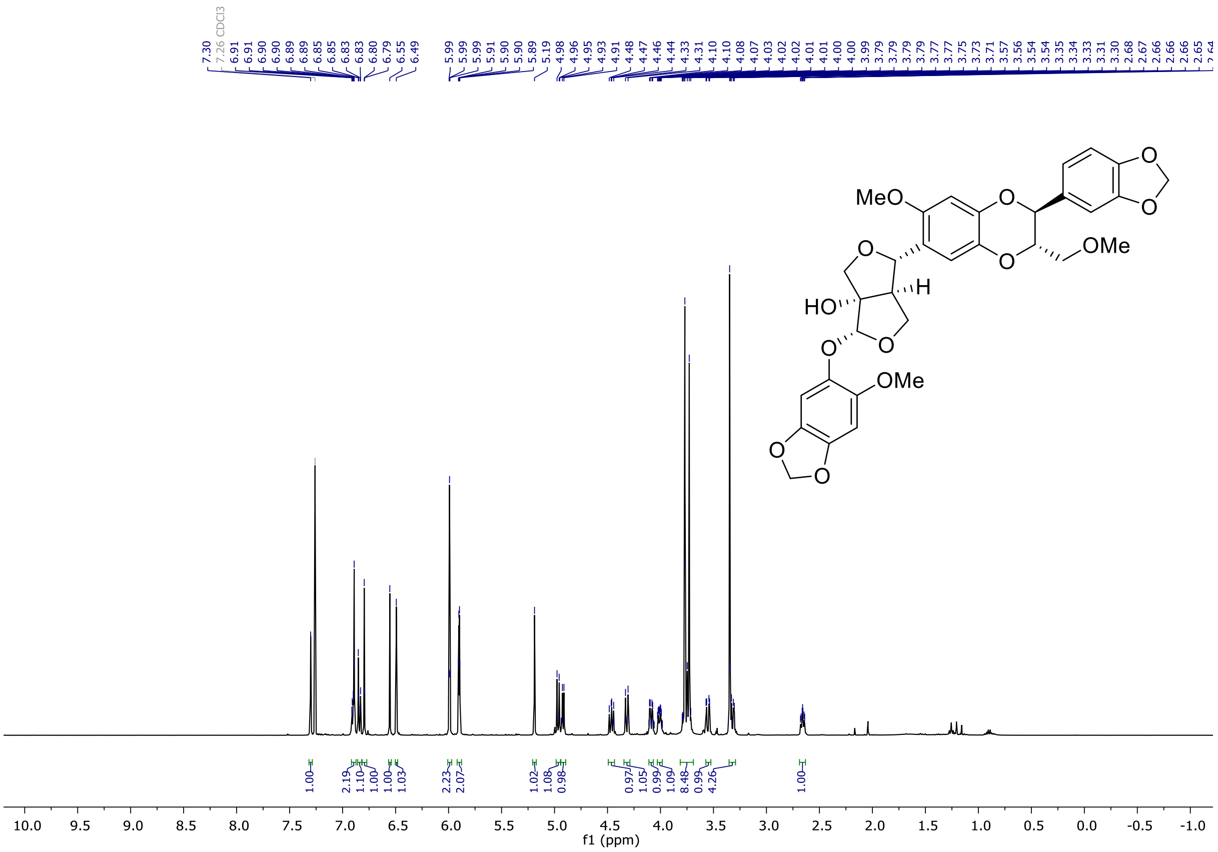

^{13}C NMR (101 MHz, $CDCl_3$) of diastereomer of haedoxan D (**SI-49**):

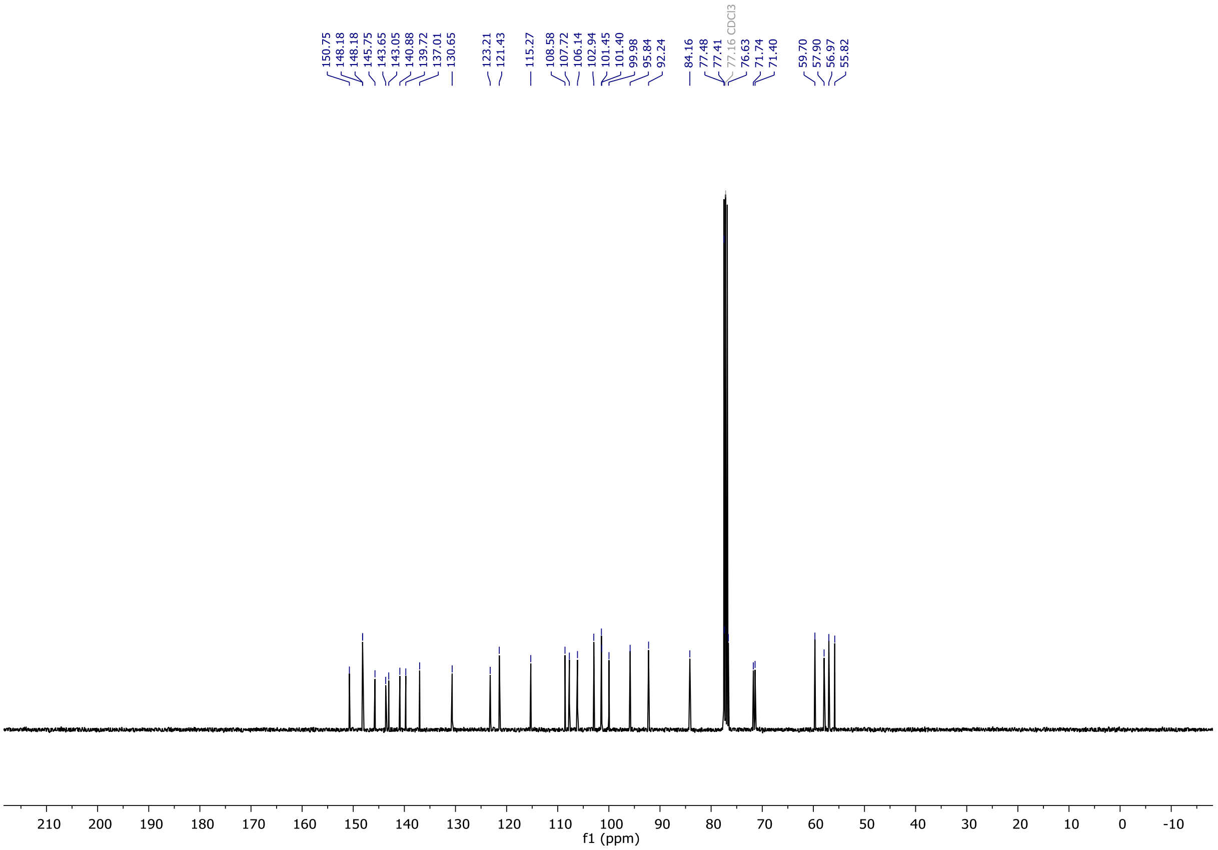

^{1}H NMR (400 MHz, $CDCl_3$) of acetal **61a**:

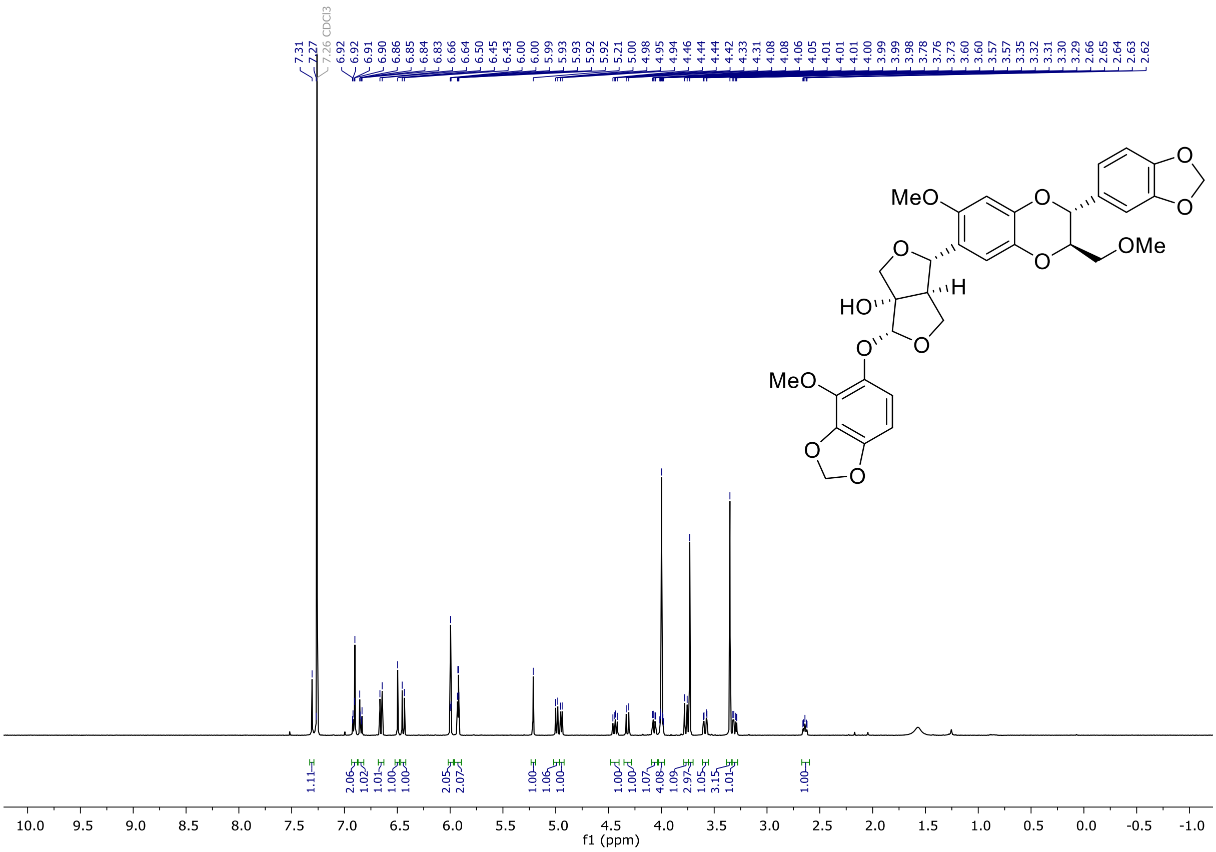

^{13}C NMR (101 MHz, $CDCl_3$) of acetal **61a**:

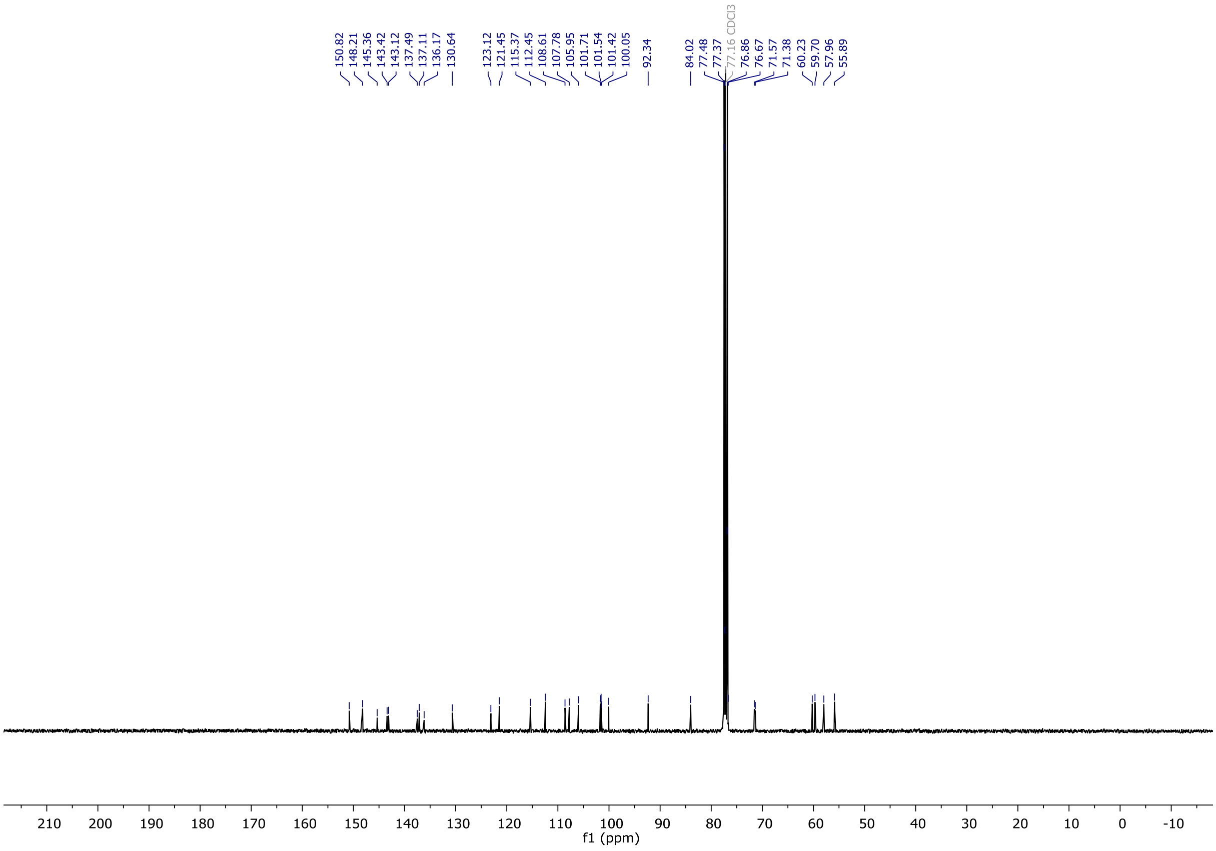

^{1}H NMR (400 MHz, $CDCl_3$) of acetal **61b**:

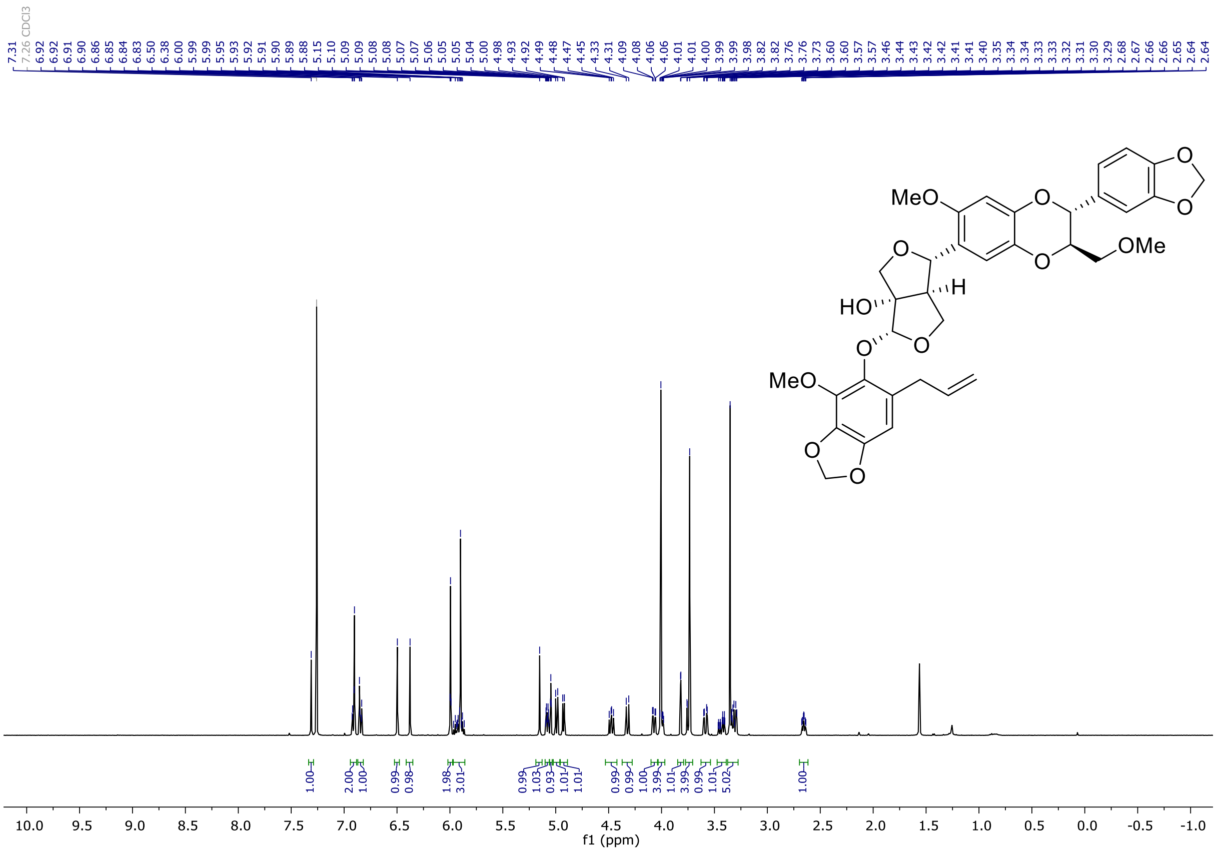

^{13}C NMR (101 MHz, $CDCl_3$) of acetal **61b**:

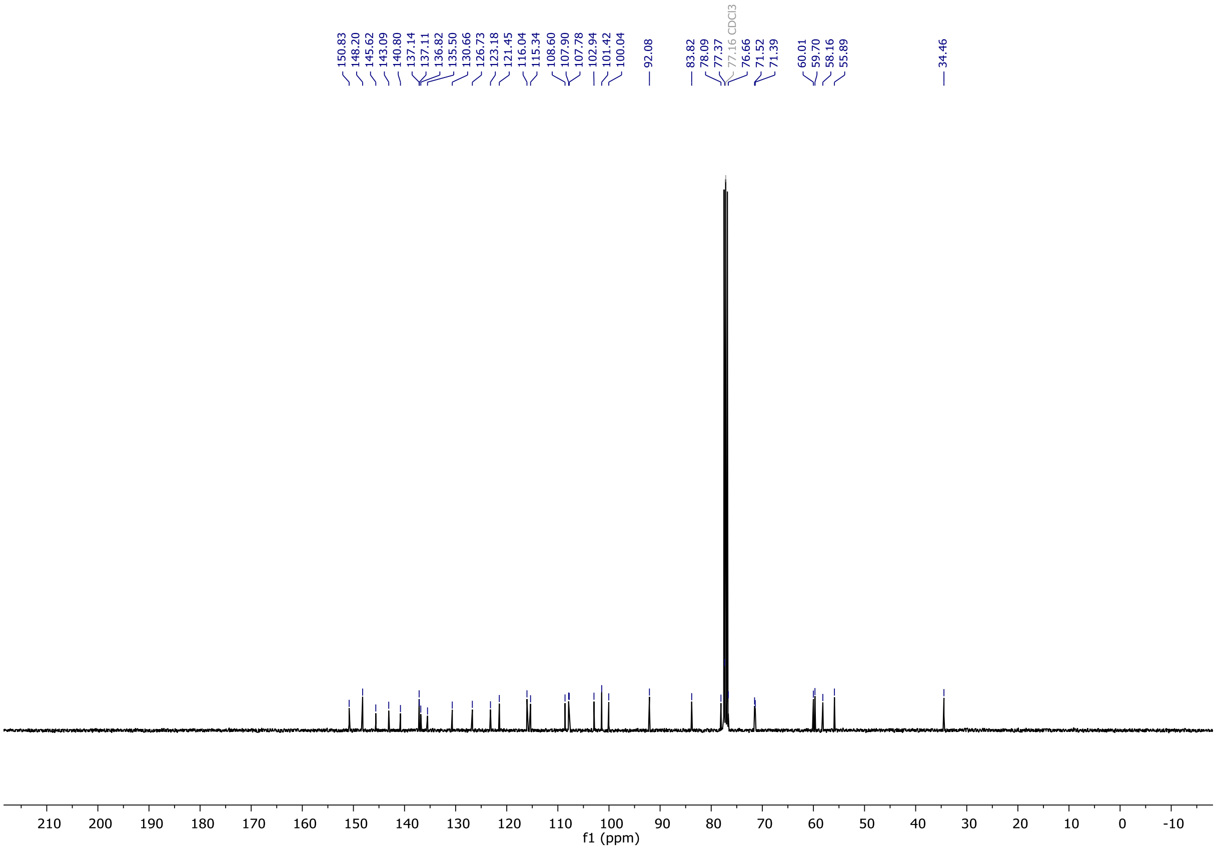

^{1}H NMR (400 MHz, $CDCl_3$) of acetal **61c**:

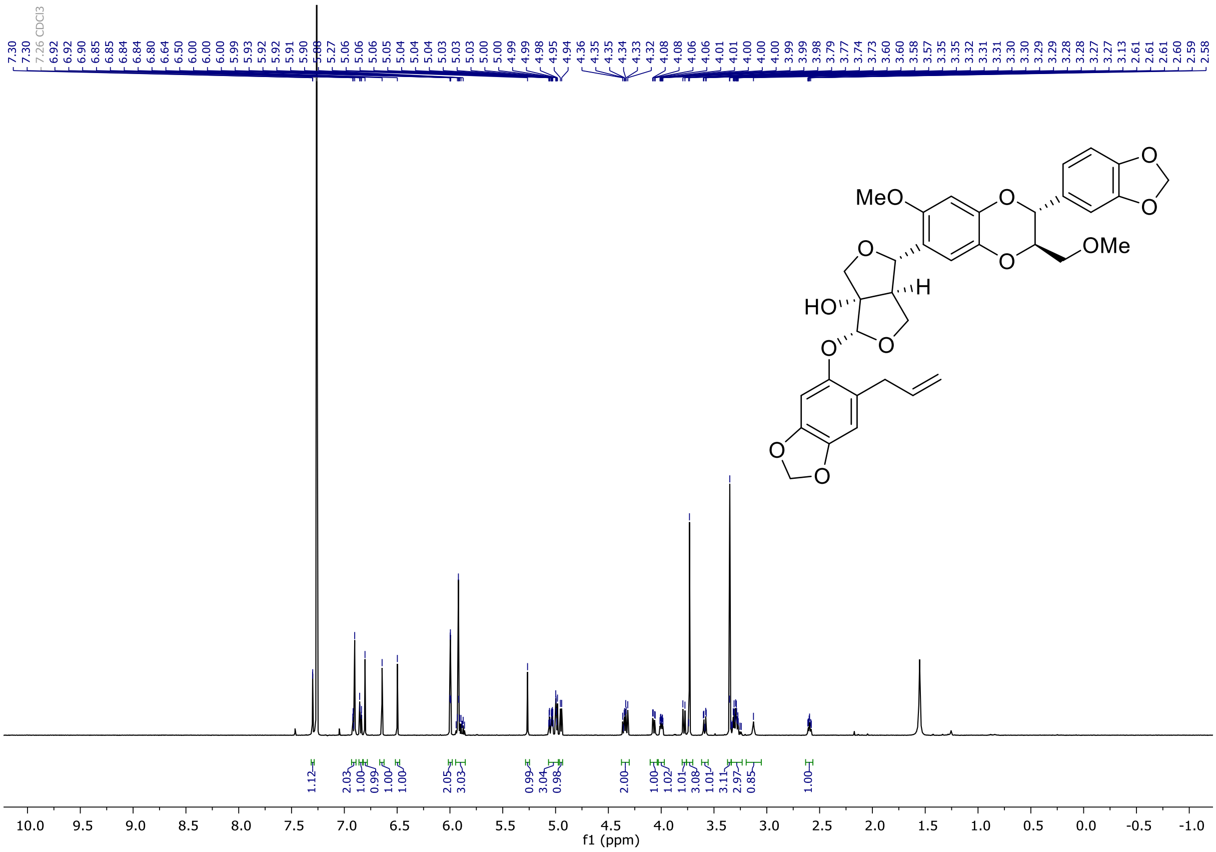

^{13}C NMR (101 MHz, $CDCl_3$) of acetal **61c**:

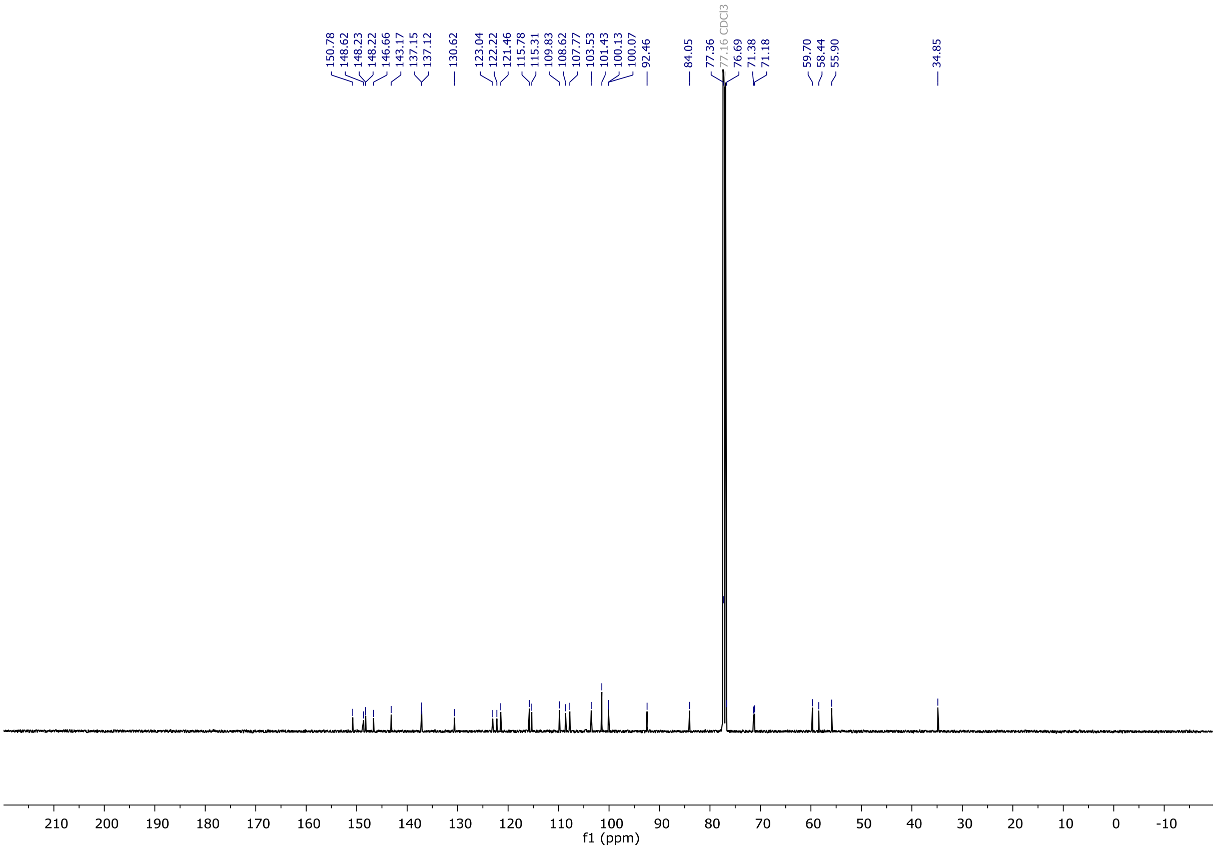

^{1}H NMR (400 MHz, $CDCl_3$) of acetal **61d**:

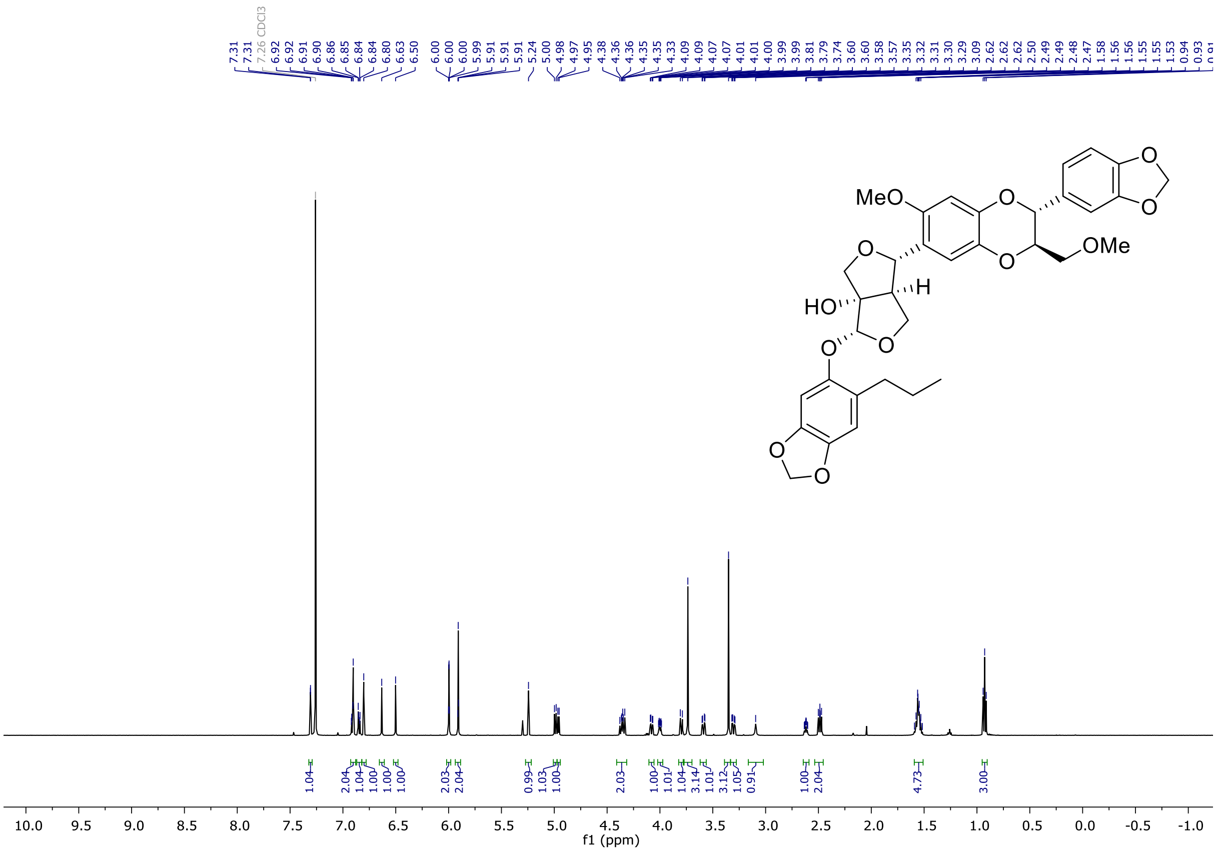

^{13}C NMR (126 MHz, $CDCl_3$) of acetal **61d**:

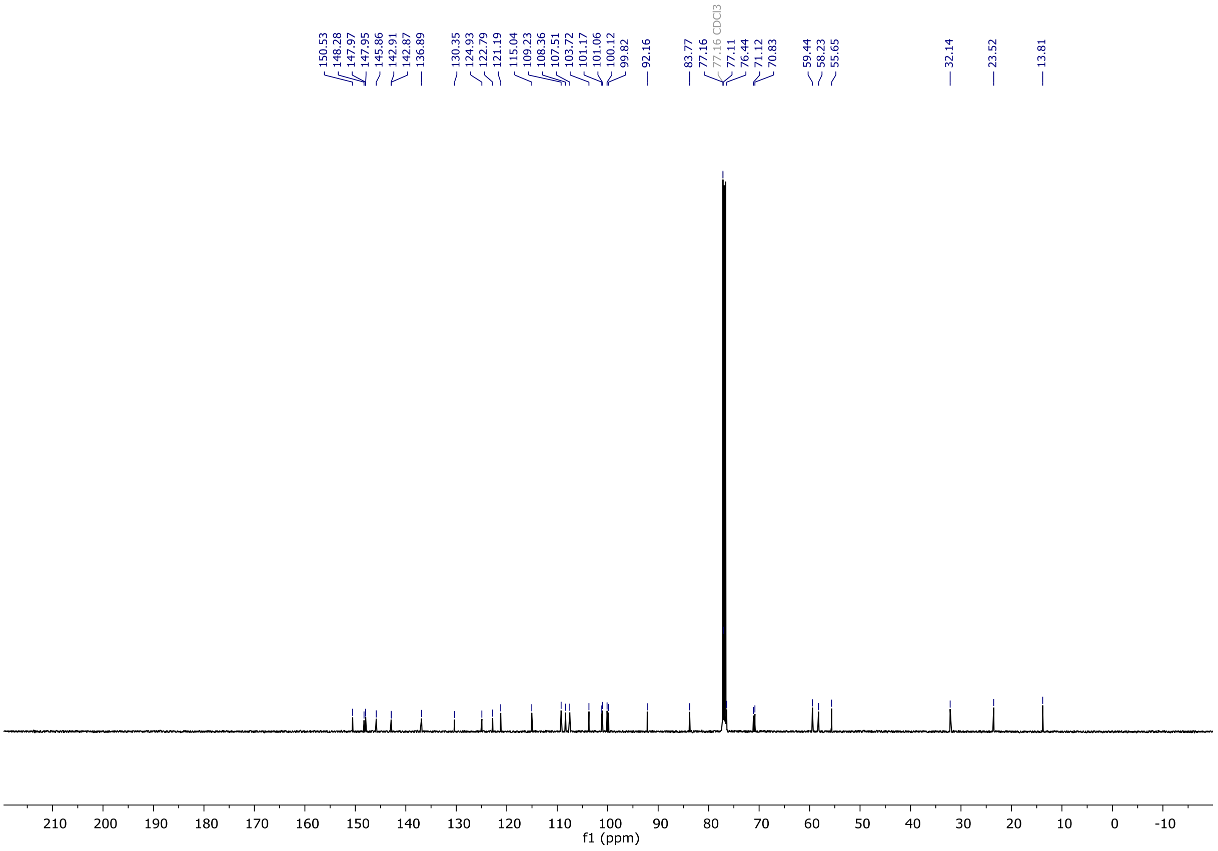

^{1}H NMR (500 MHz, $CDCl_3$) of acetal **61e**:

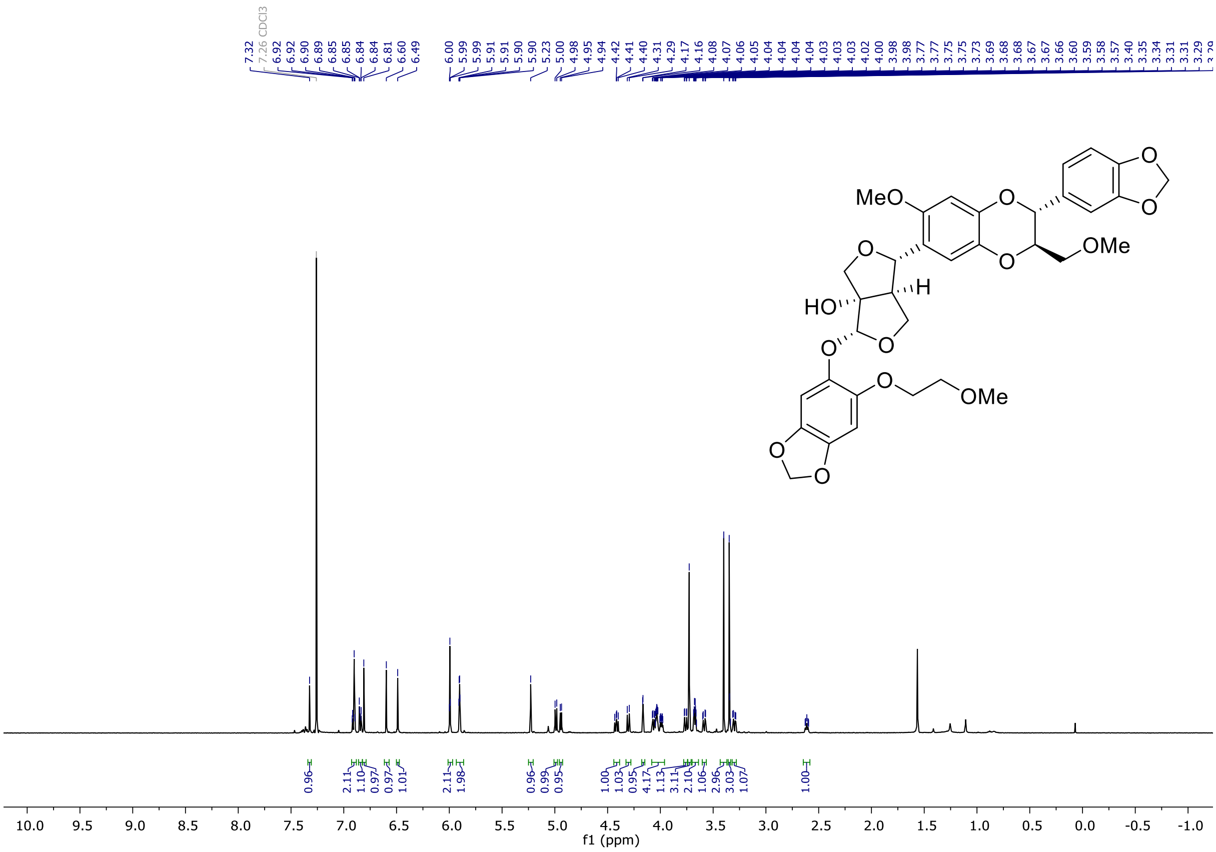

^{13}C NMR (126 MHz, $CDCl_3$) of acetal **61e**:

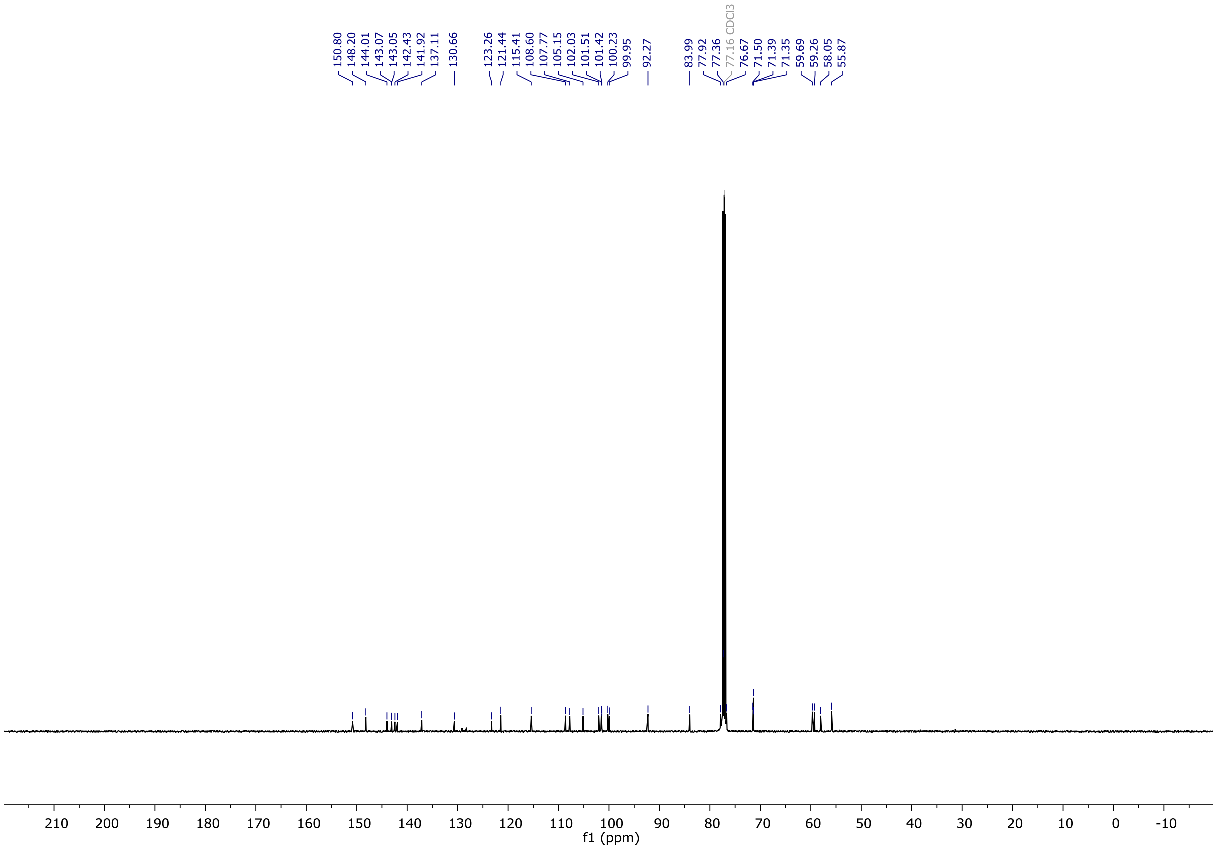

^{1}H NMR (400 MHz, $CDCl_3$) of acetal **61f**:

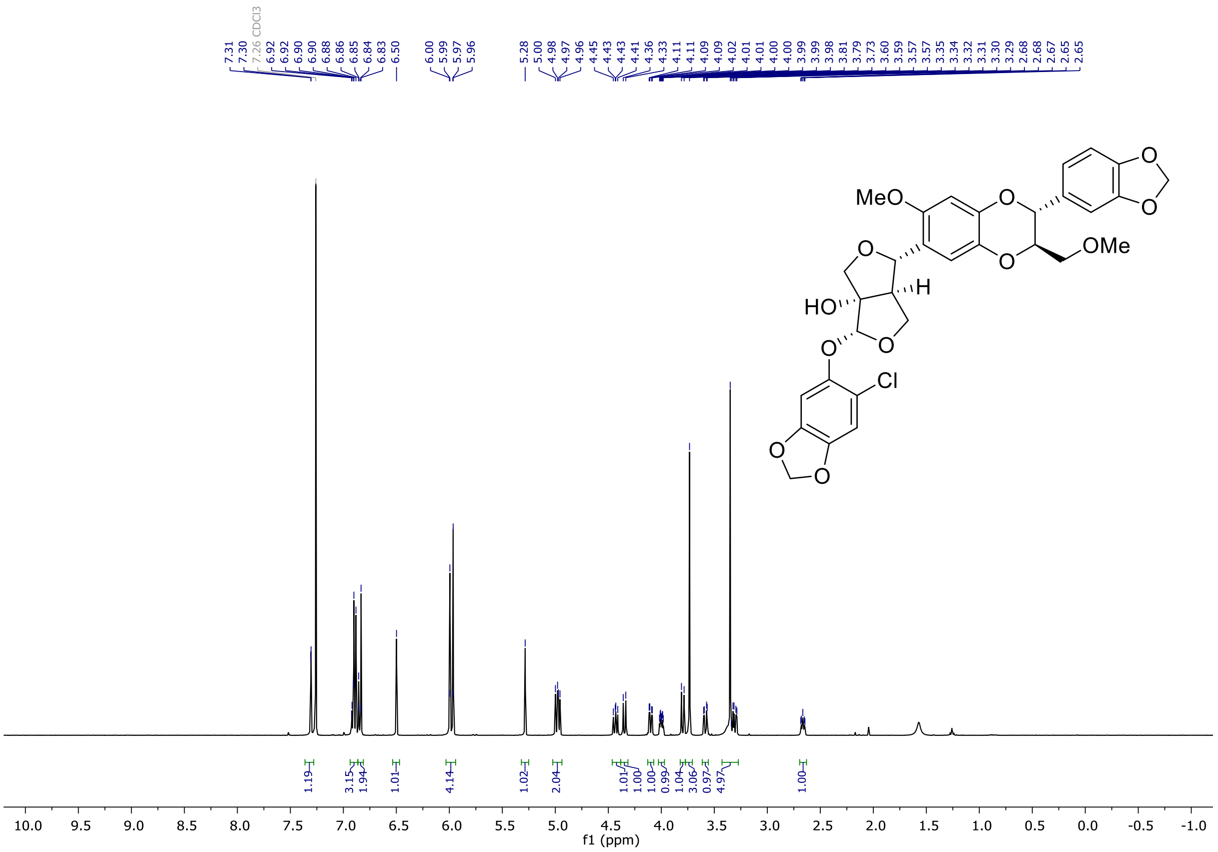

^{13}C NMR (101 MHz, $CDCl_3$) of acetal **61f**:

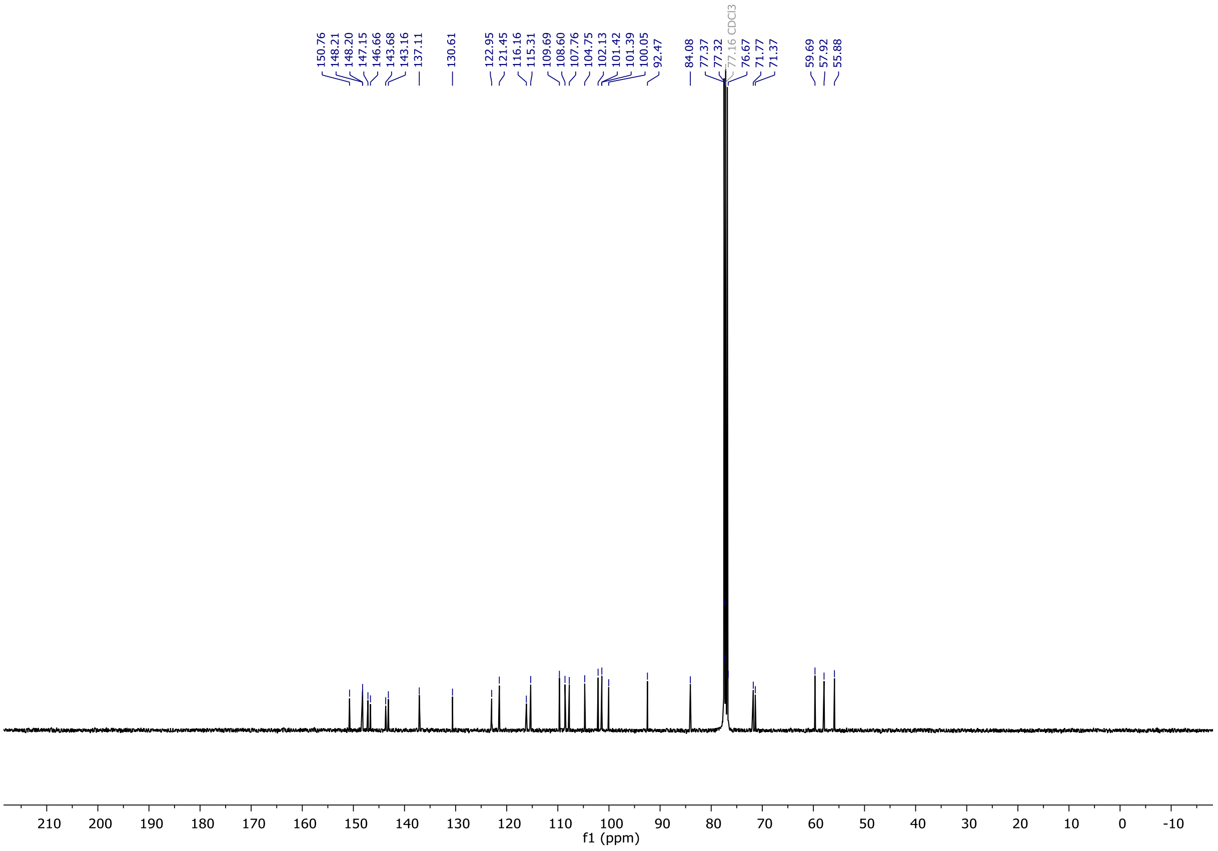

^{1}H NMR (400 MHz, $CDCl_3$) of acetal **61g**:

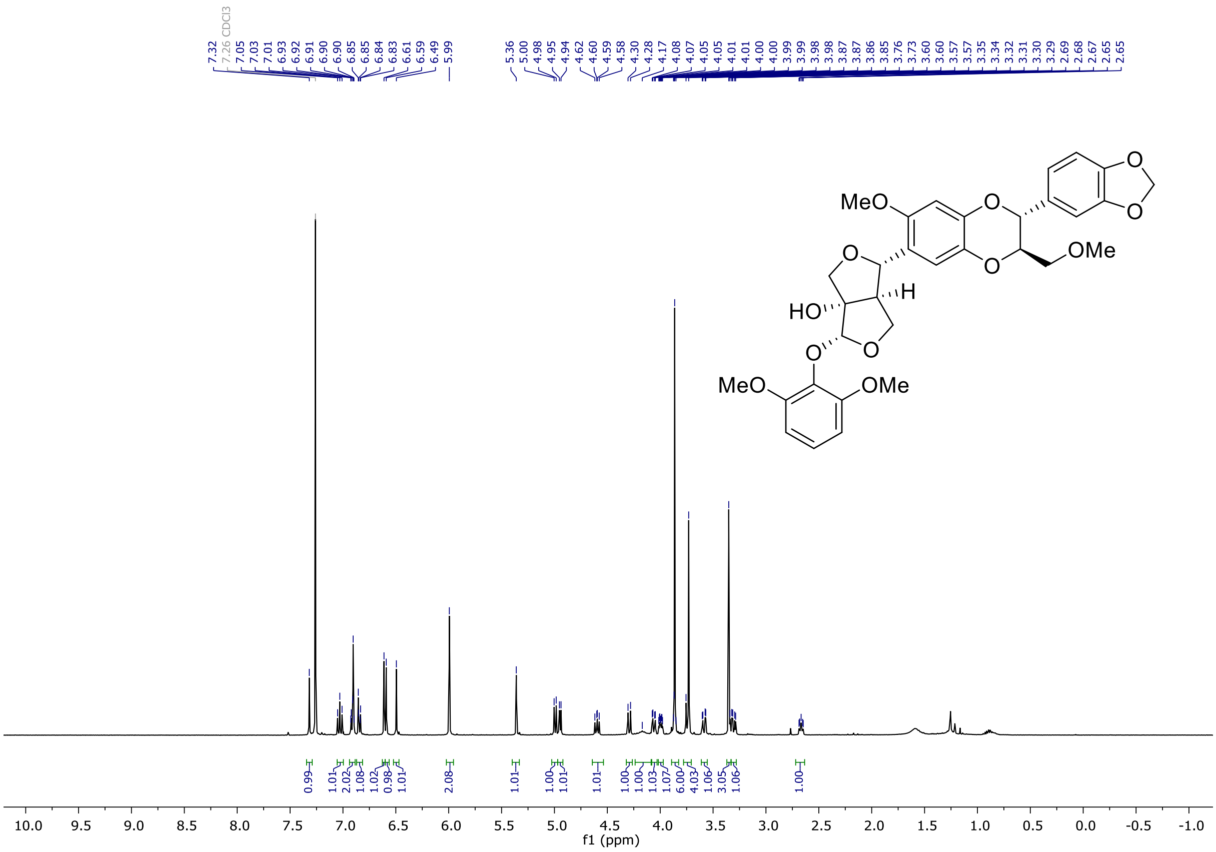

^{13}C NMR (101 MHz, $CDCl_3$) of acetal **61g**:

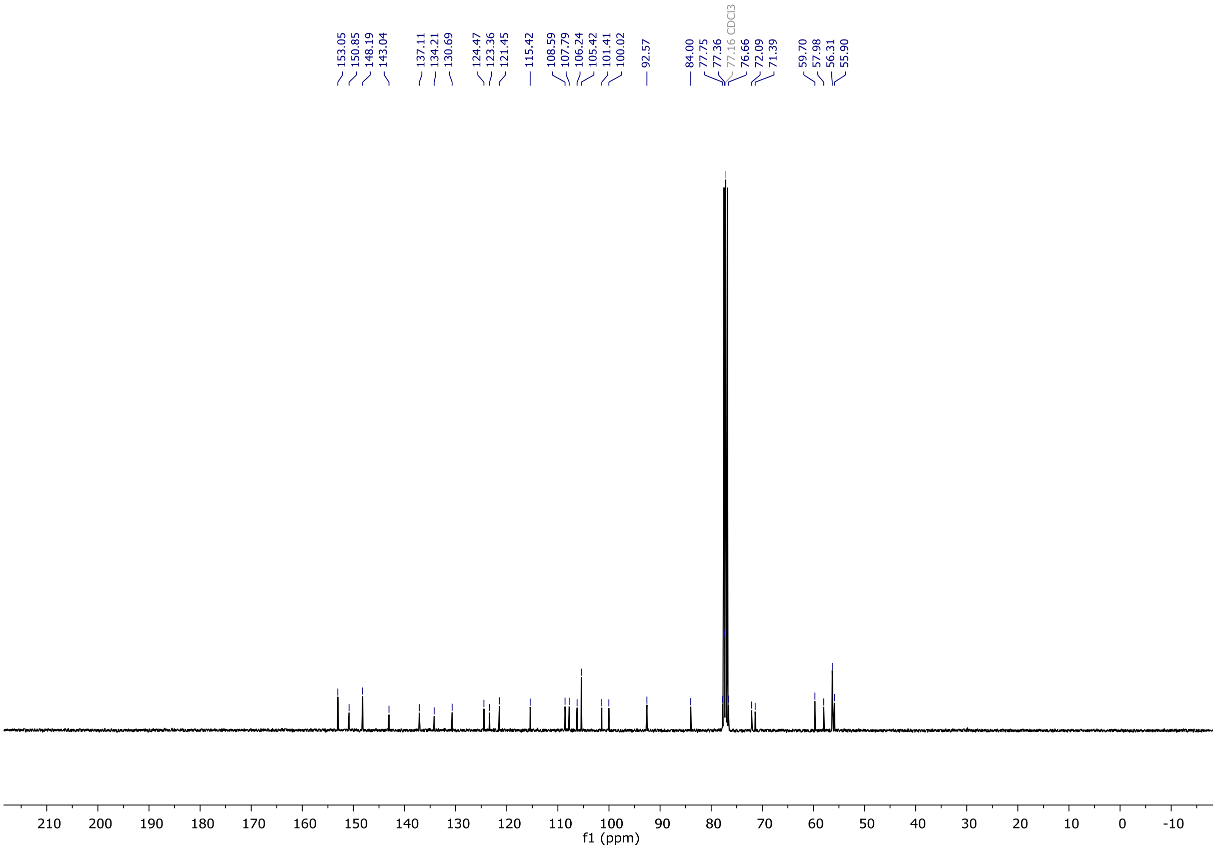

^{1}H NMR (400 MHz, $CDCl_3$) of acetal **61h**:

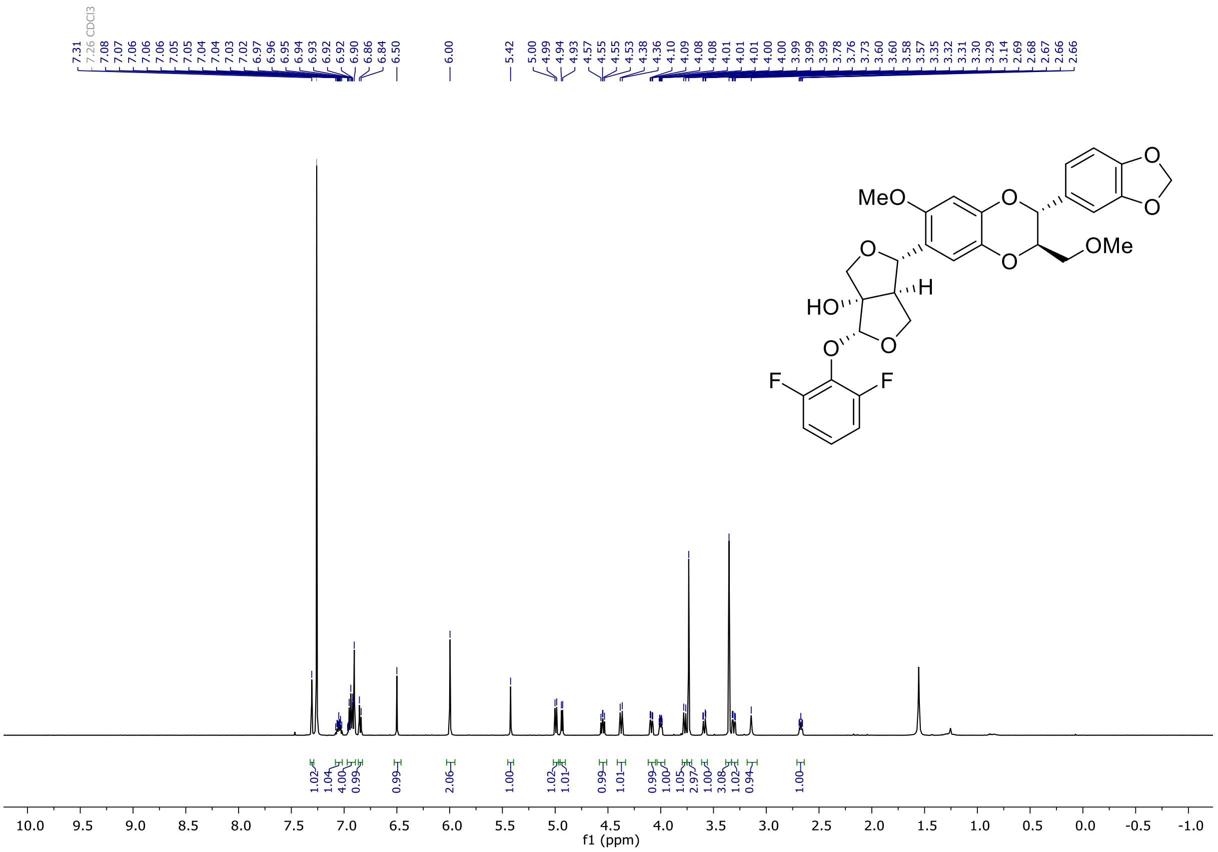

^{13}C NMR (101 MHz, $CDCl_3$) of acetal **61h:**

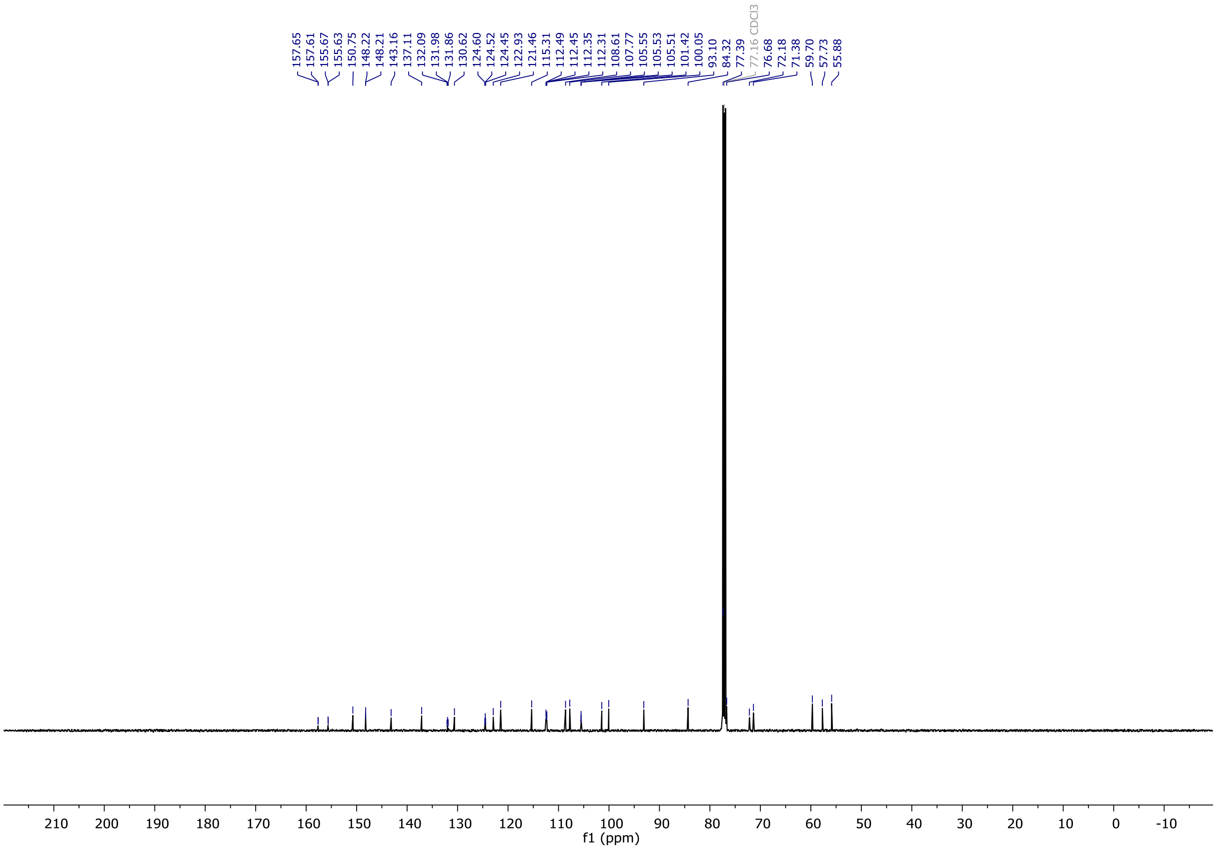

^{1}H NMR (400 MHz, $CDCl_3$) of thioacetal **SI-51**:

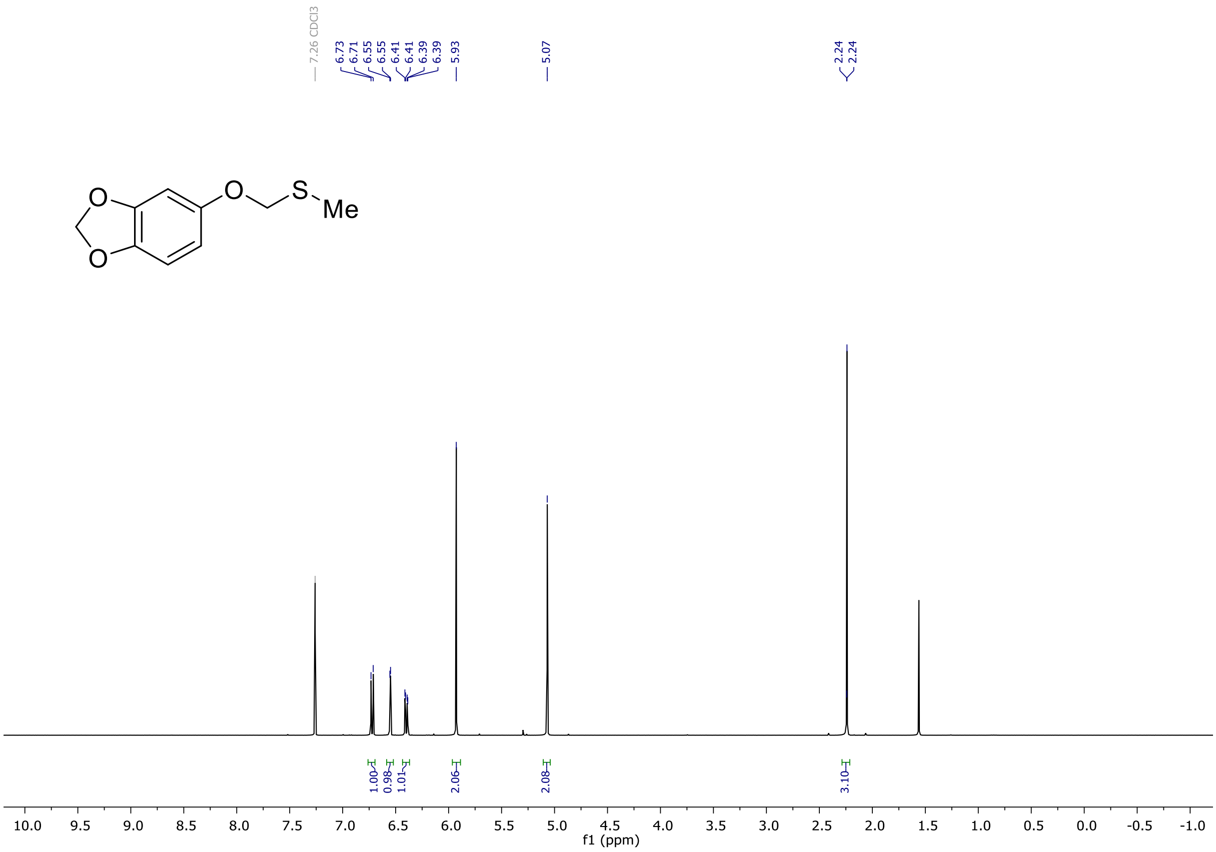

^{13}C NMR (101 MHz, $CDCl_3$) of thioacetal **SI-51:**

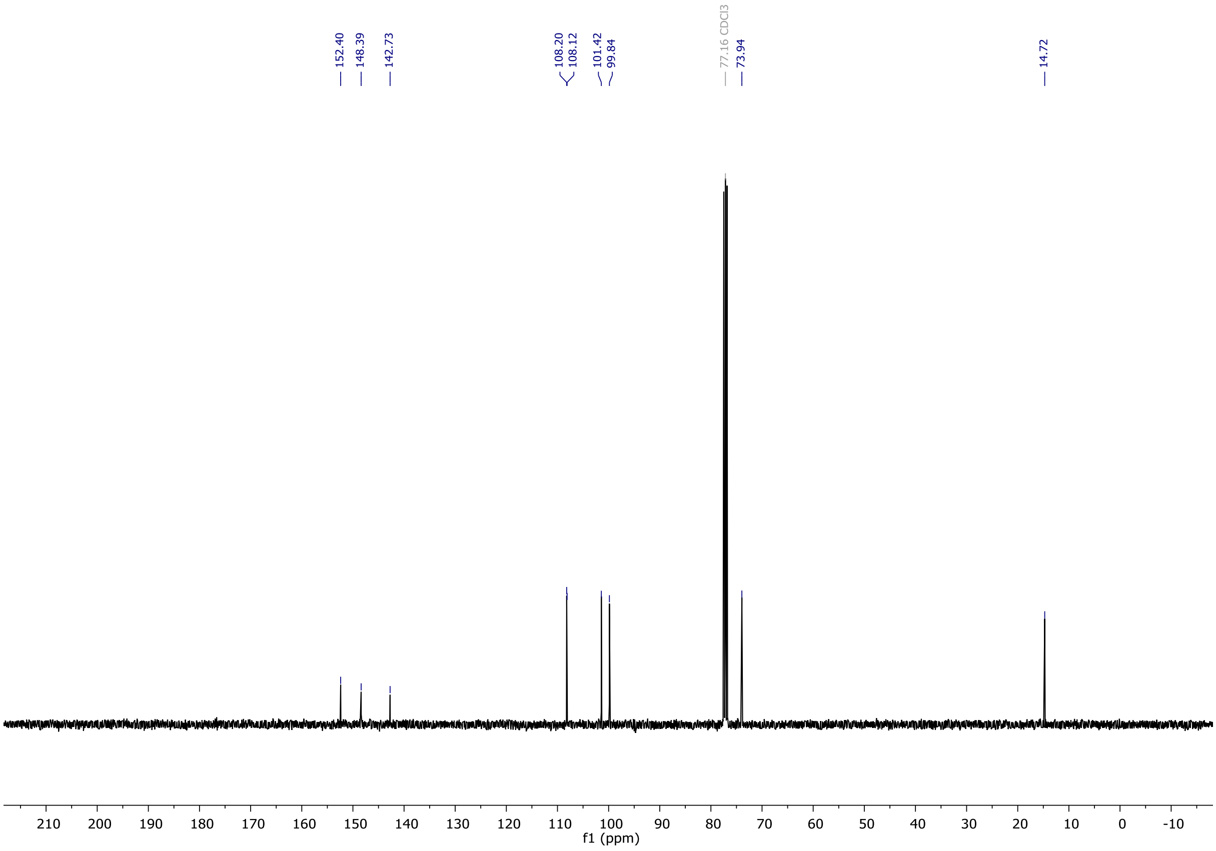

^{1}H NMR (400 MHz, $CDCl_3$) of chloromethyl ether **SI-52**:

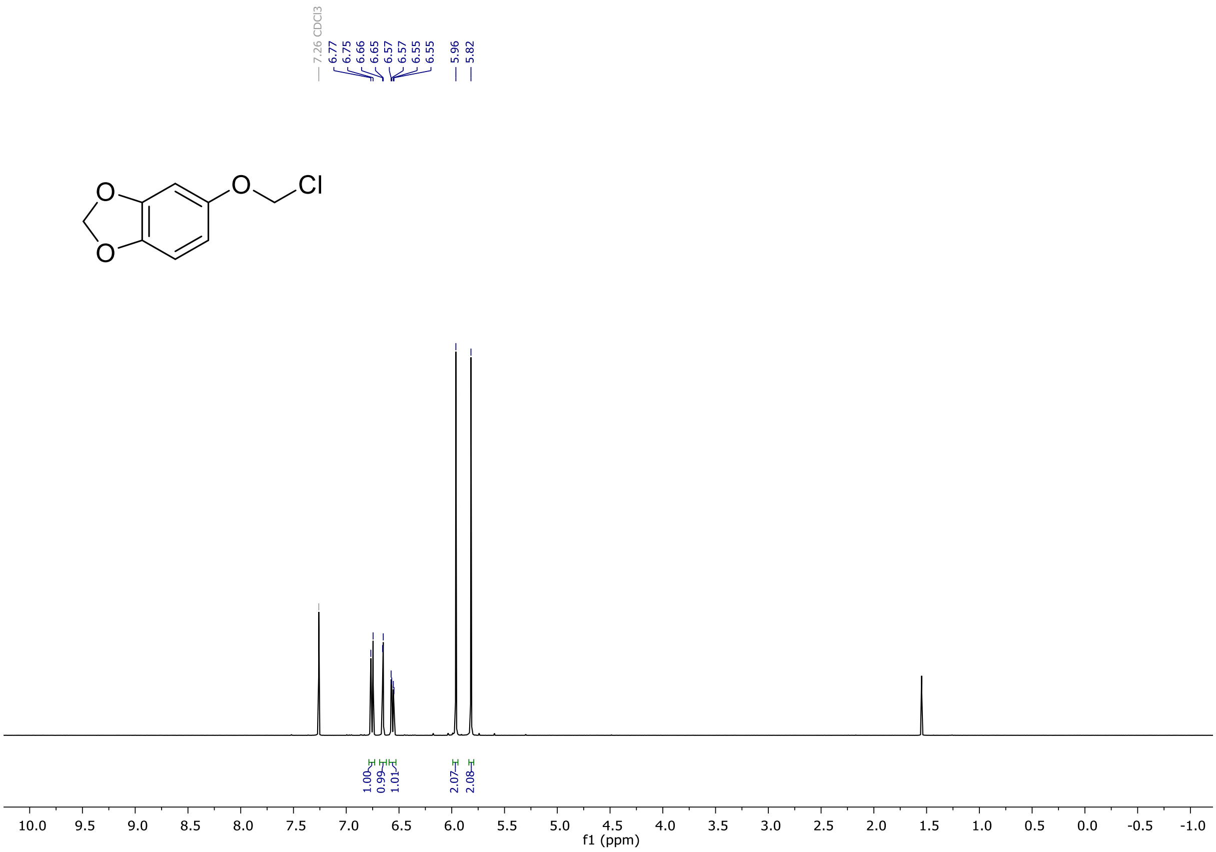

^{13}C NMR (101 MHz, $CDCl_3$) of chloromethyl ether **SI-52:**

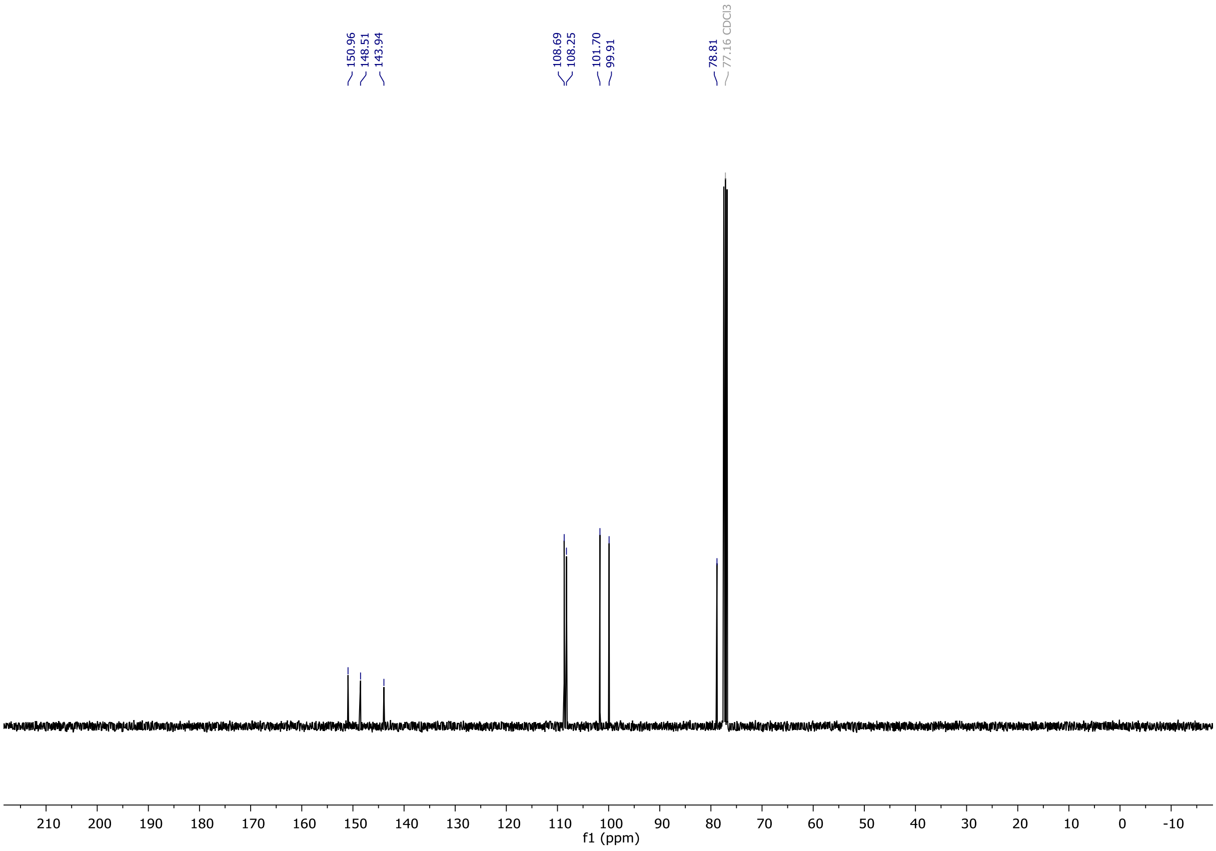

^{1}H NMR (400 MHz, $CDCl_3$) of thioacetal **SI-53**:

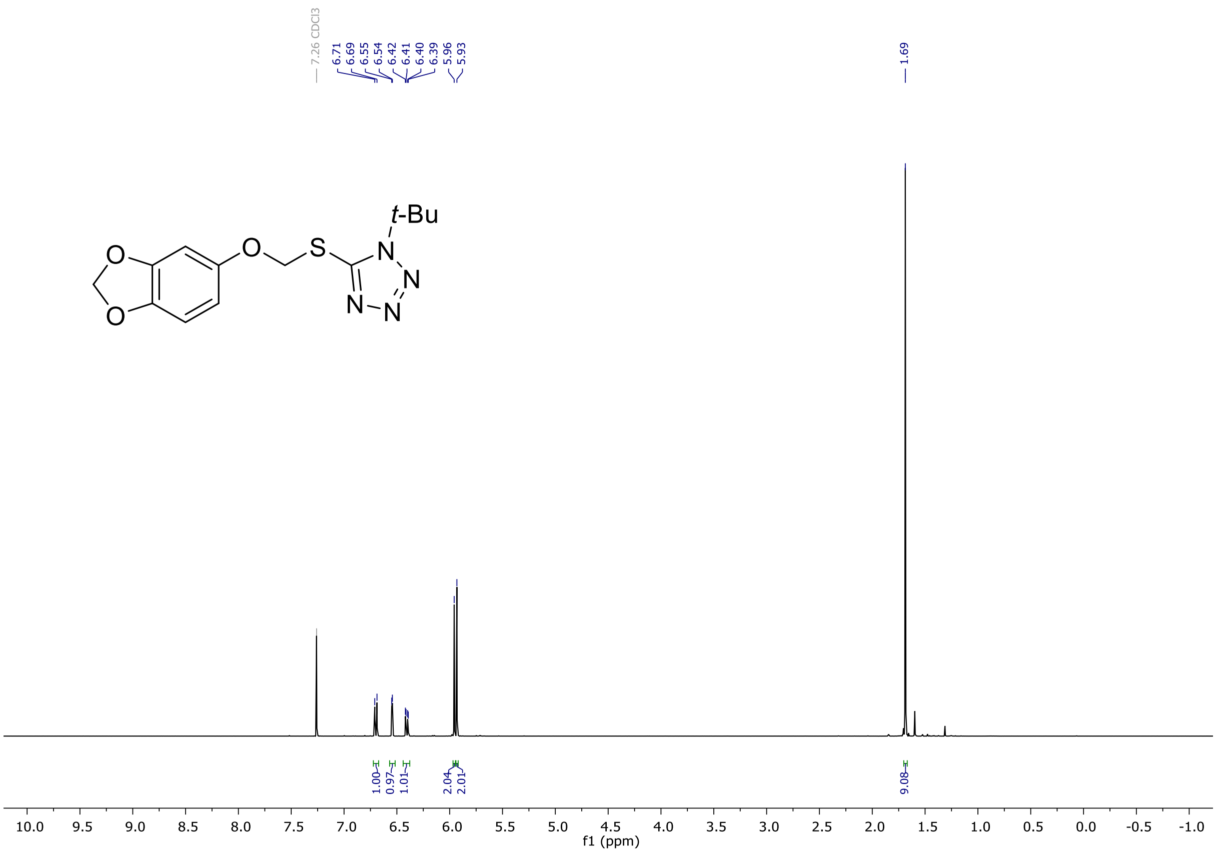

^{13}C NMR (101 MHz, $CDCl_3$) of thioacetal **SI-53:**

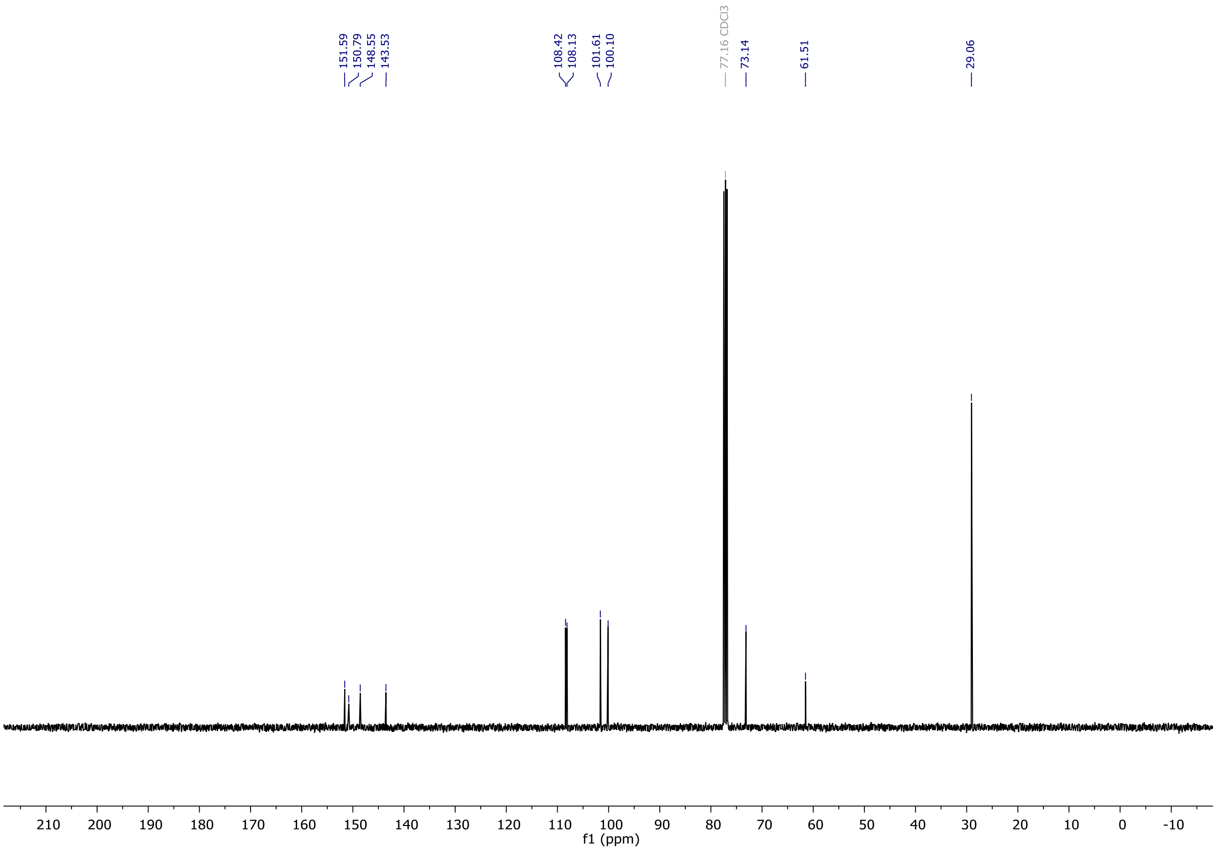

^{1}H NMR (400 MHz, $CDCl_3$) of sulfone **42b**:

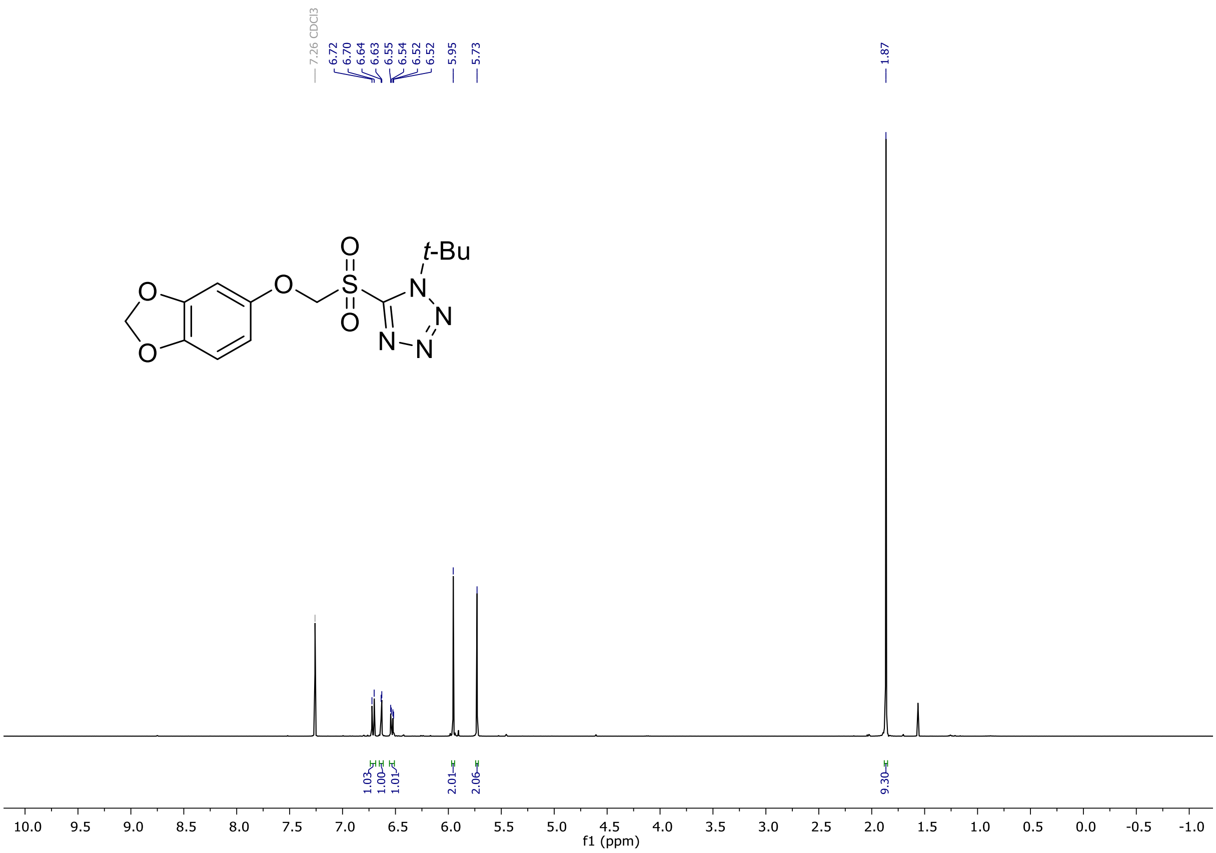

^{13}C NMR (101 MHz, $CDCl_3$) of sulfone **42b:**

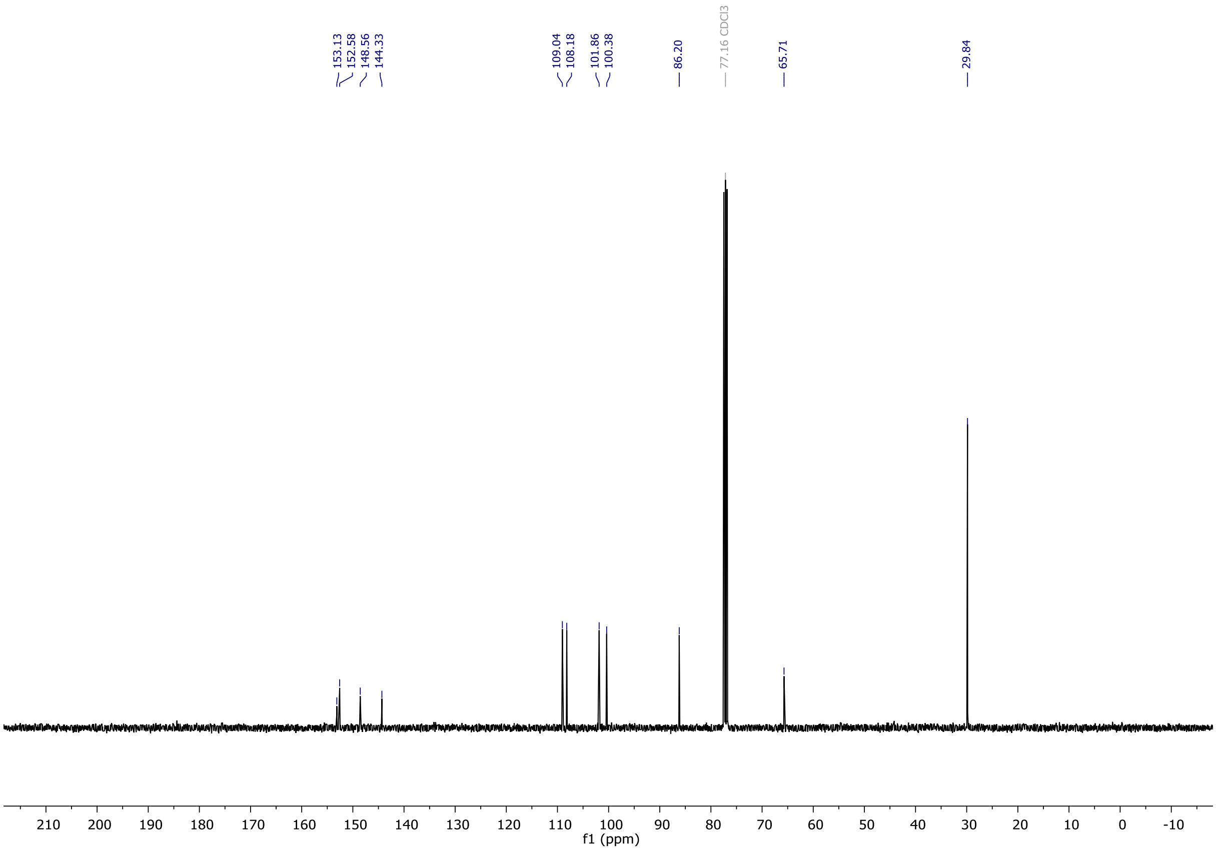

^{1}H NMR (400 MHz, $CDCl_3$) of thioacetal **SI-54**:

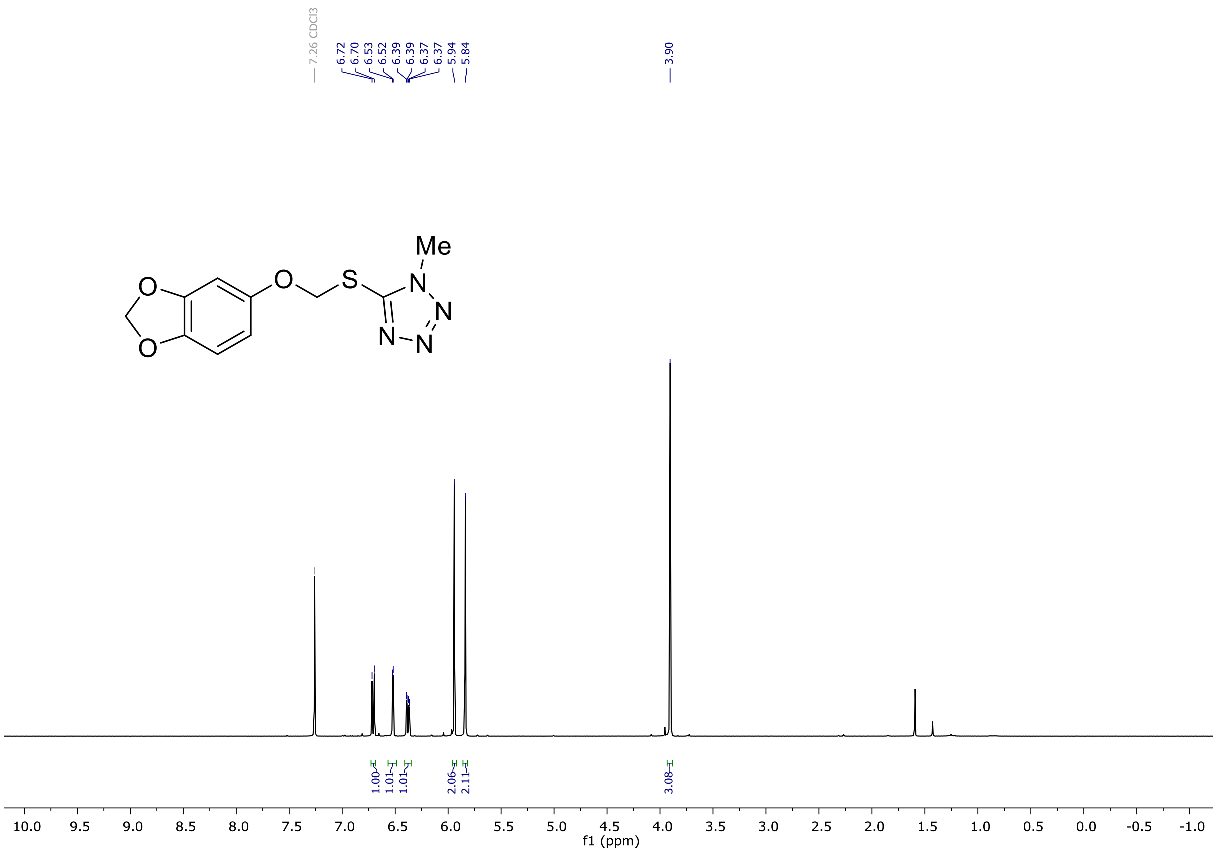

^{13}C NMR (101 MHz, $CDCl_3$) of thioacetal **SI-54:**

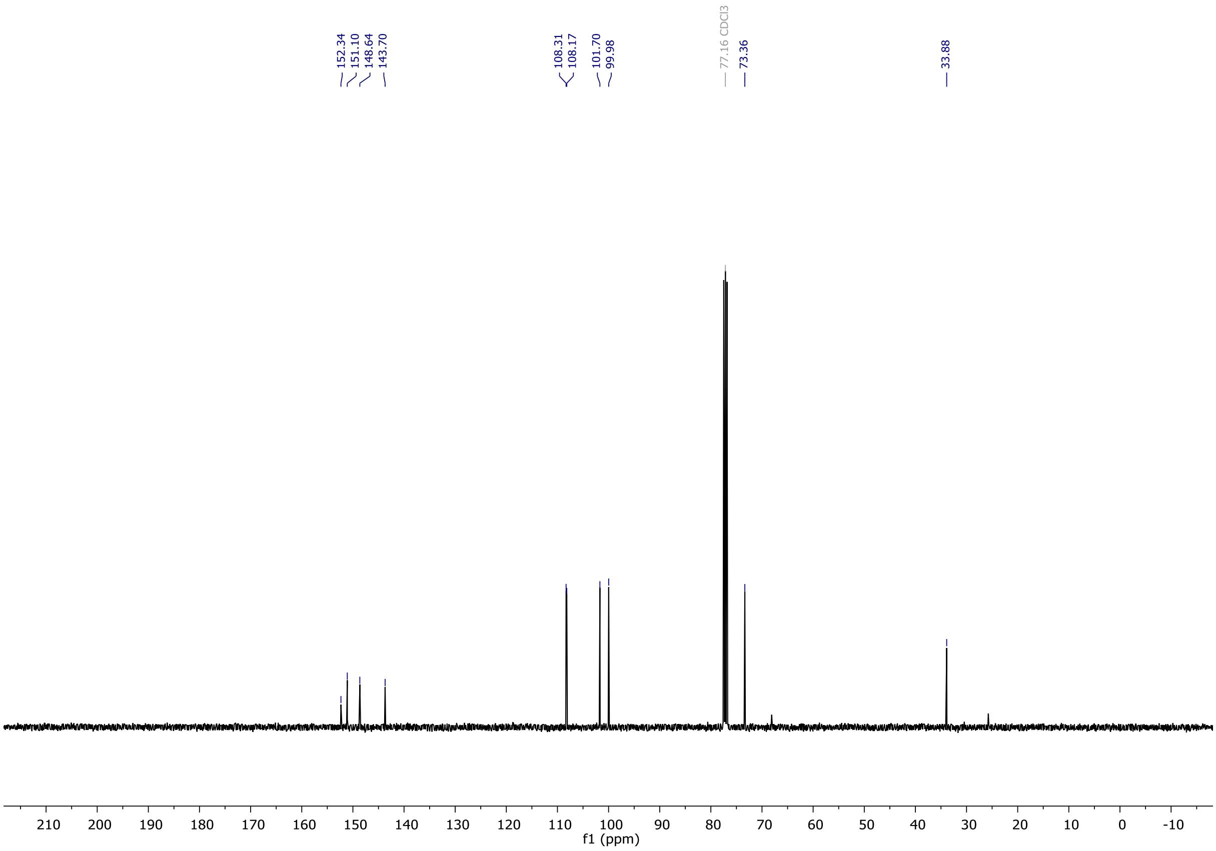

^{1}H NMR (400 MHz, $CDCl_3$) of sulfone **42a**:

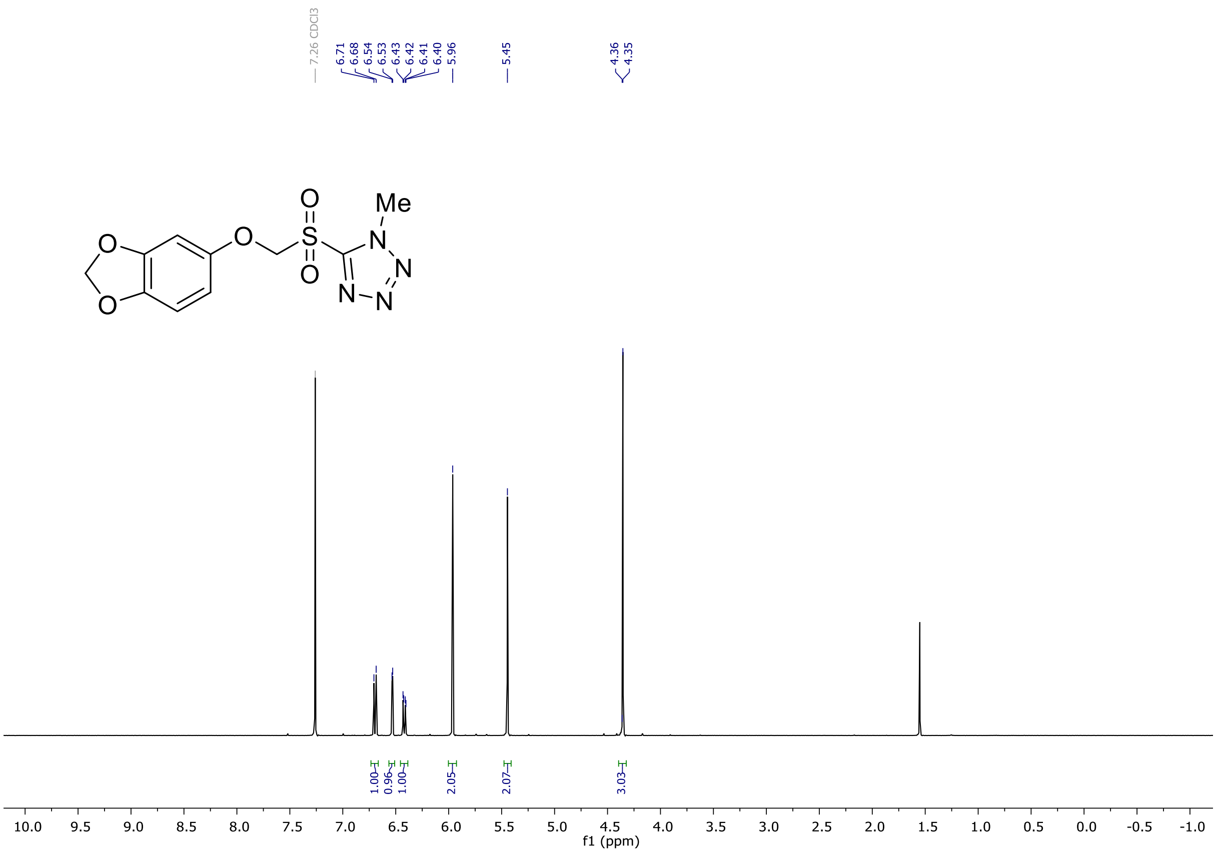

^{13}C NMR (101 MHz, $CDCl_3$) of sulfone **42a:**

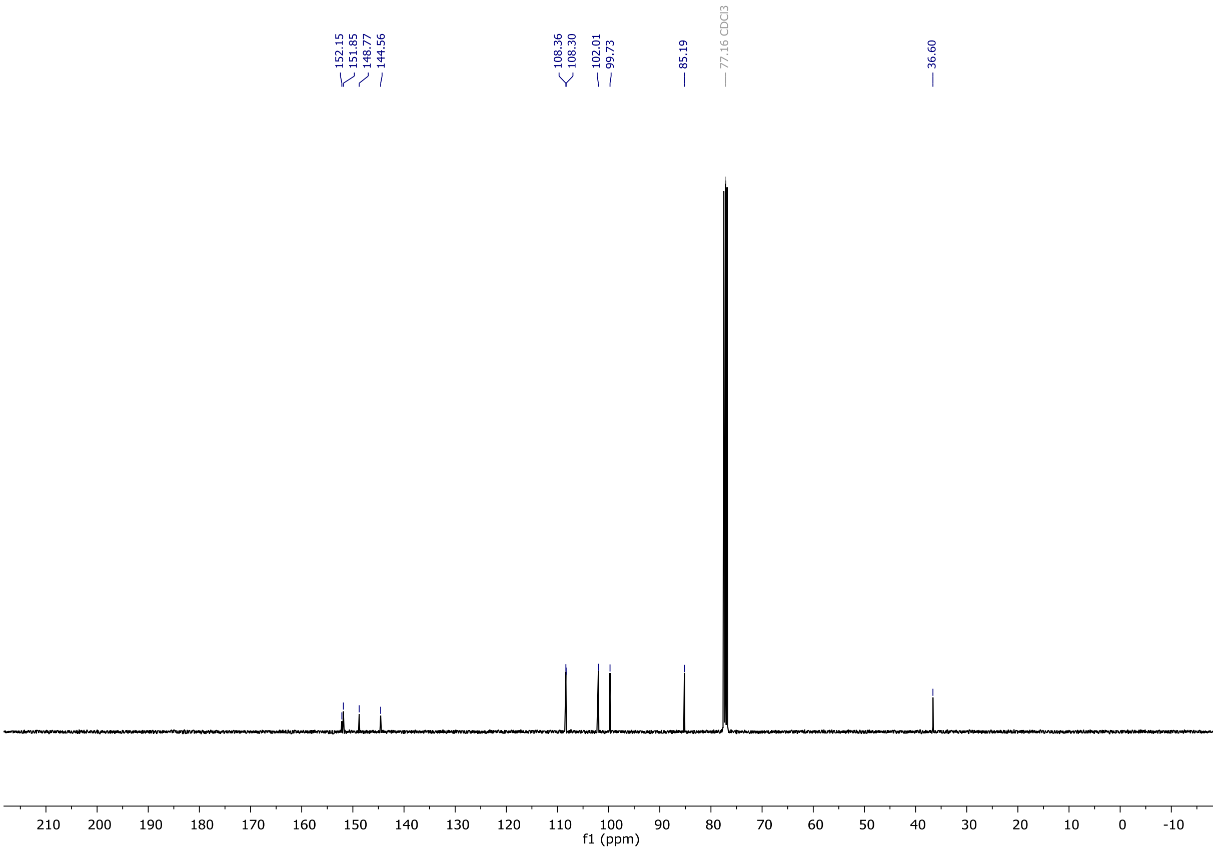

^{1}H NMR (400 MHz, $CDCl_3$) of enol ether (*Z*)-**41**:

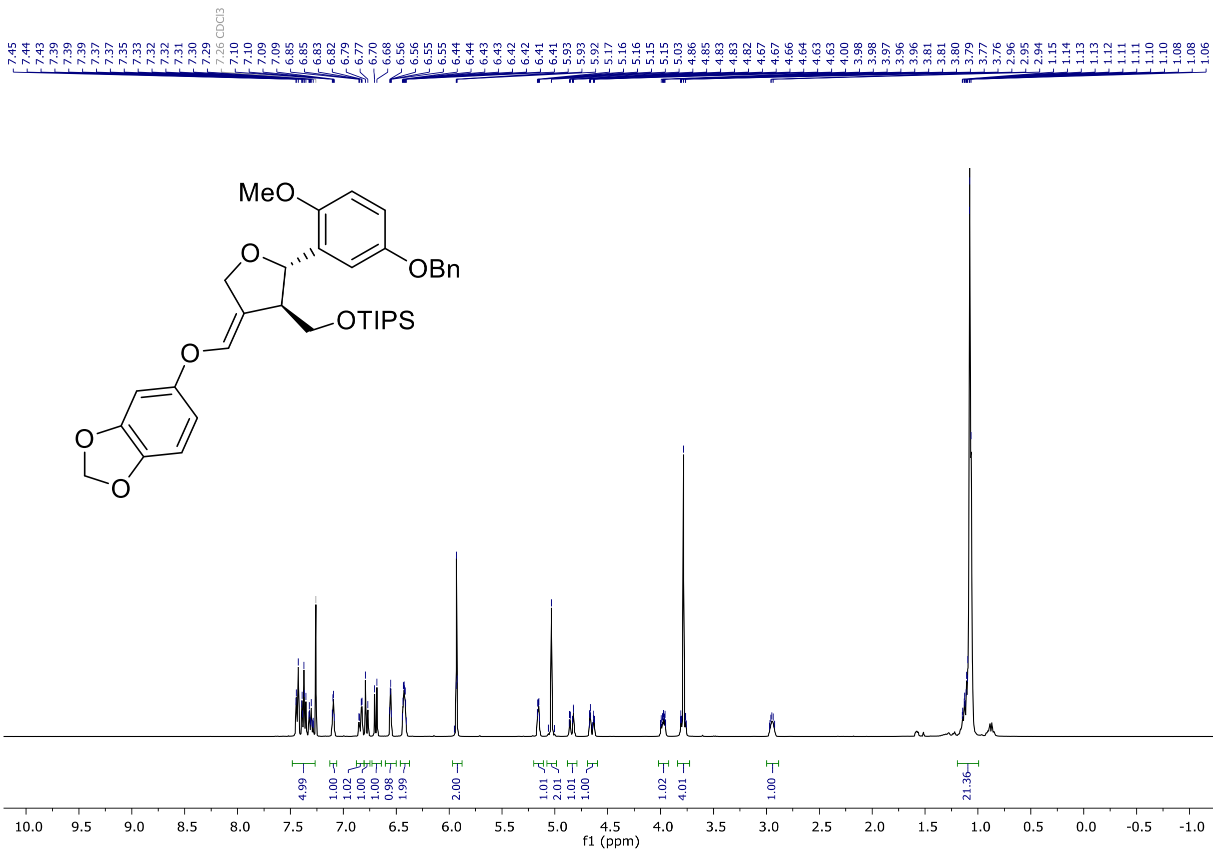

^{13}C NMR (101 MHz, $CDCl_3$) of enol ether (*Z*)-**41:**

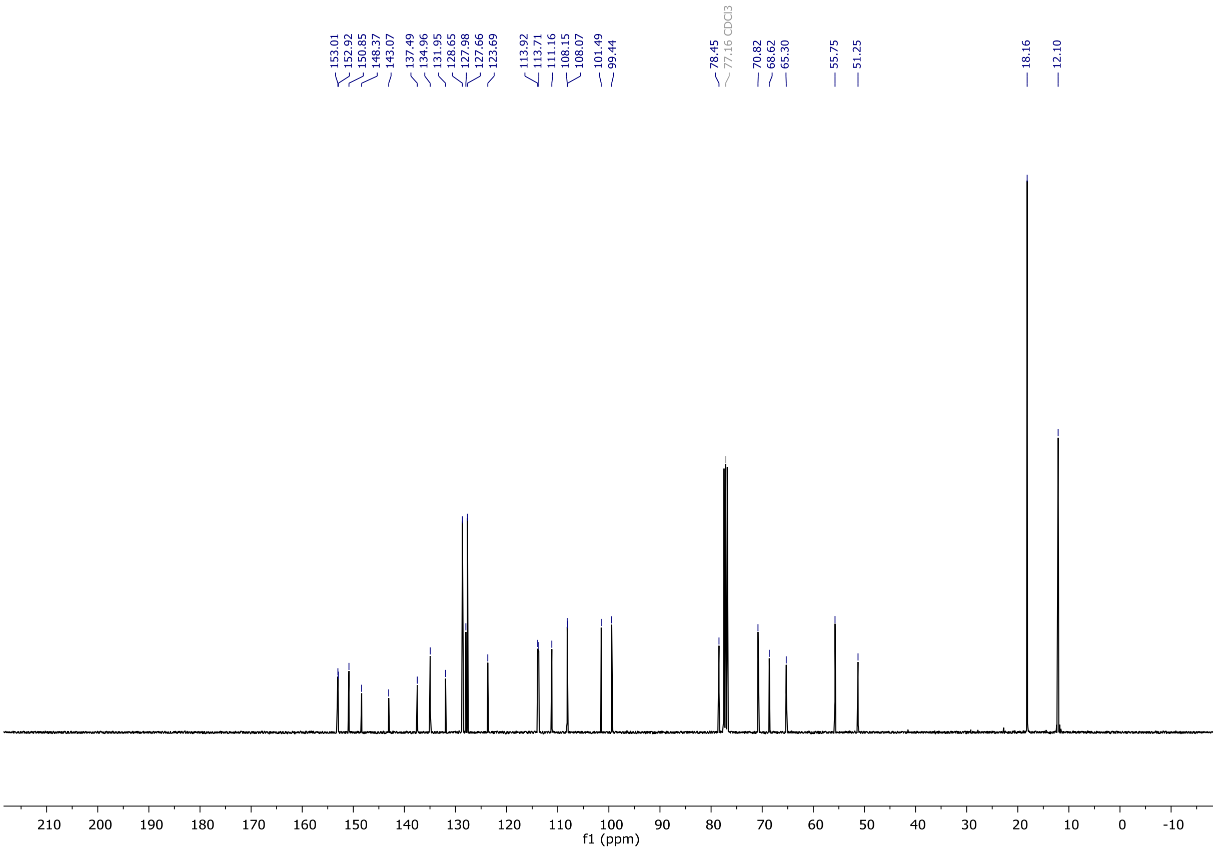

^{1}H NMR (400 MHz, $CDCl_3$) of enol ether (*E*)-**41**:

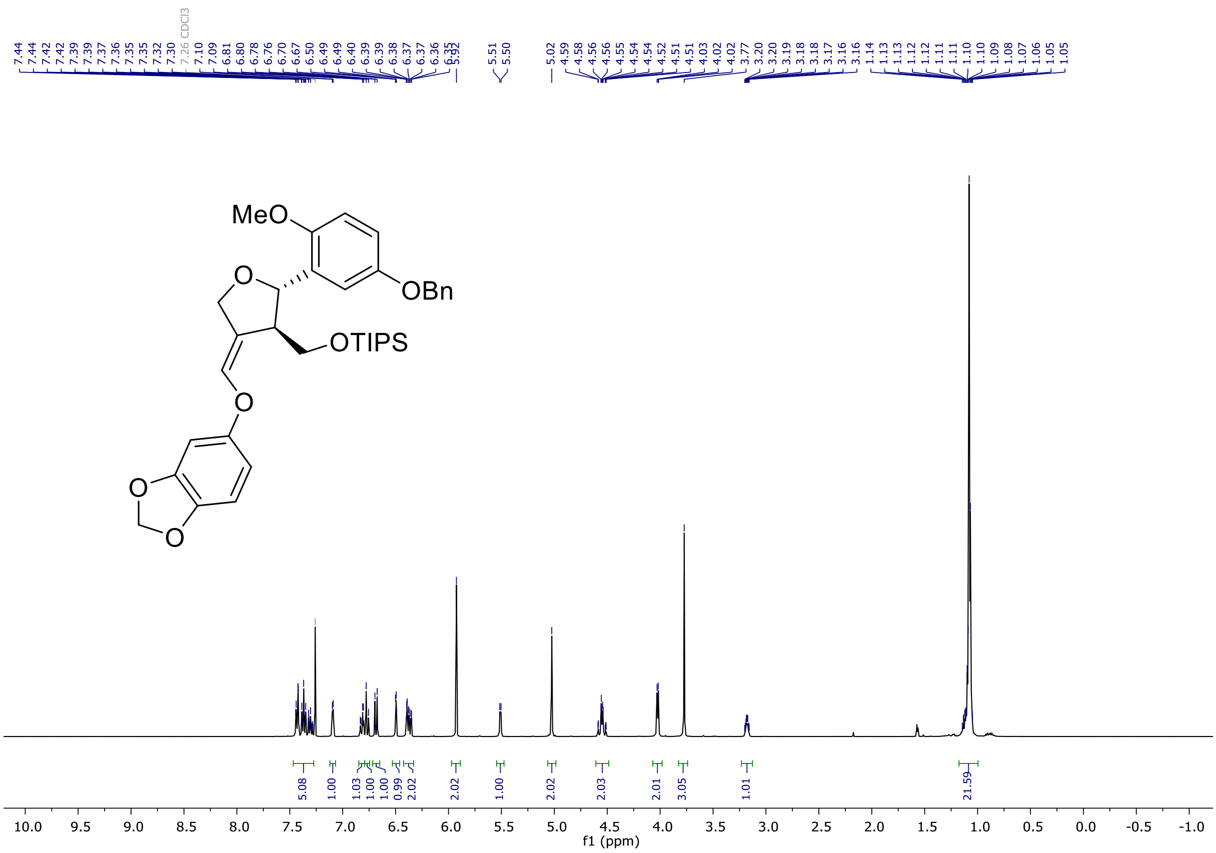

^{13}C NMR (101 MHz, $CDCl_3$) of enol ether (*E*)-**41**:

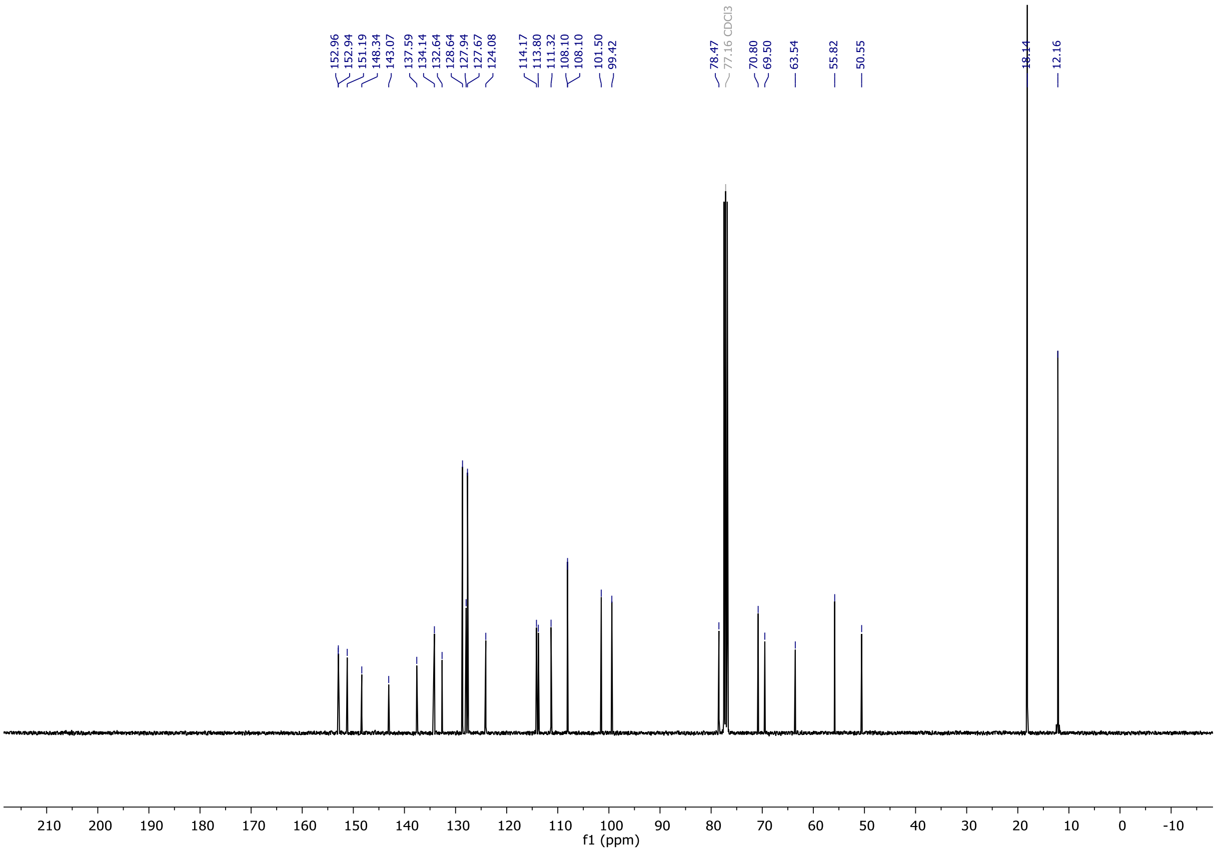

^{1}H NMR (400 MHz, $CDCl_3$) of epoxide **43**:

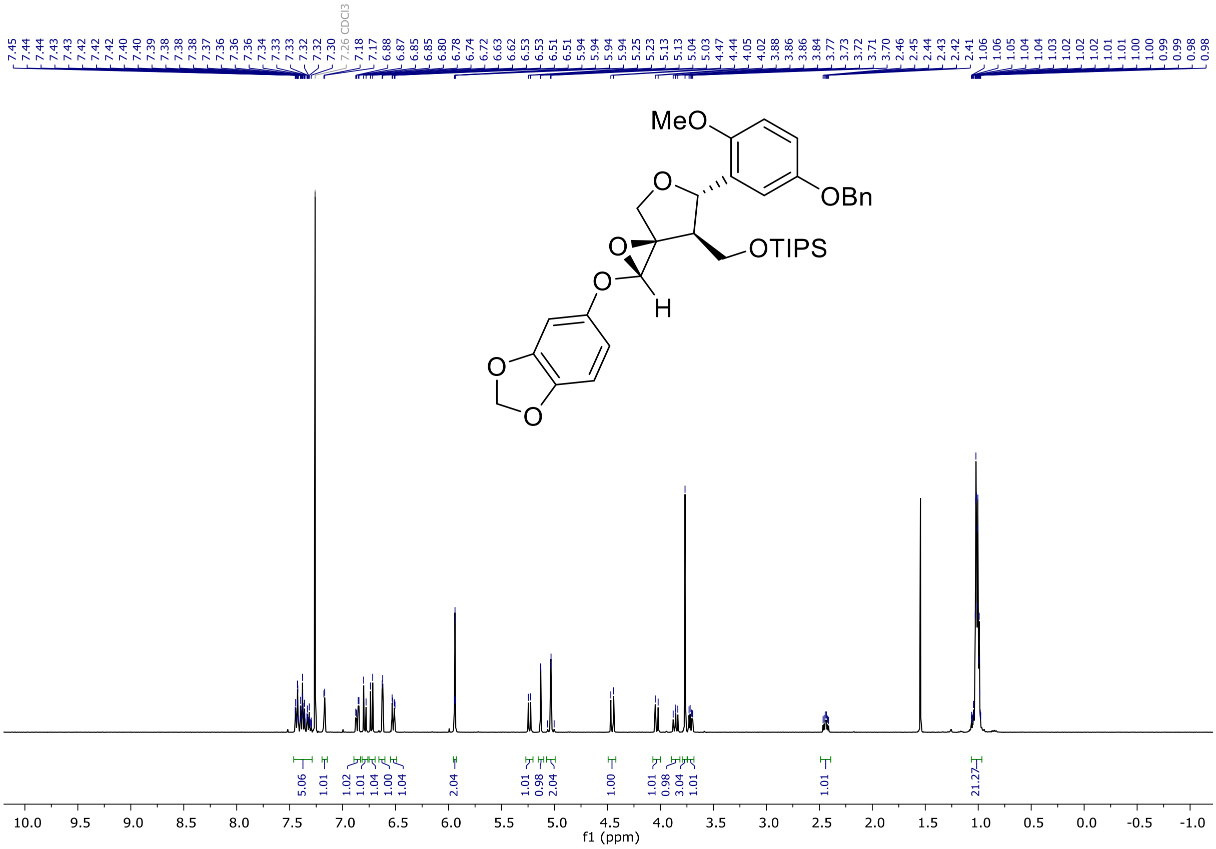

^{13}C NMR (101 MHz, $CDCl_3$) of epoxide **43:**

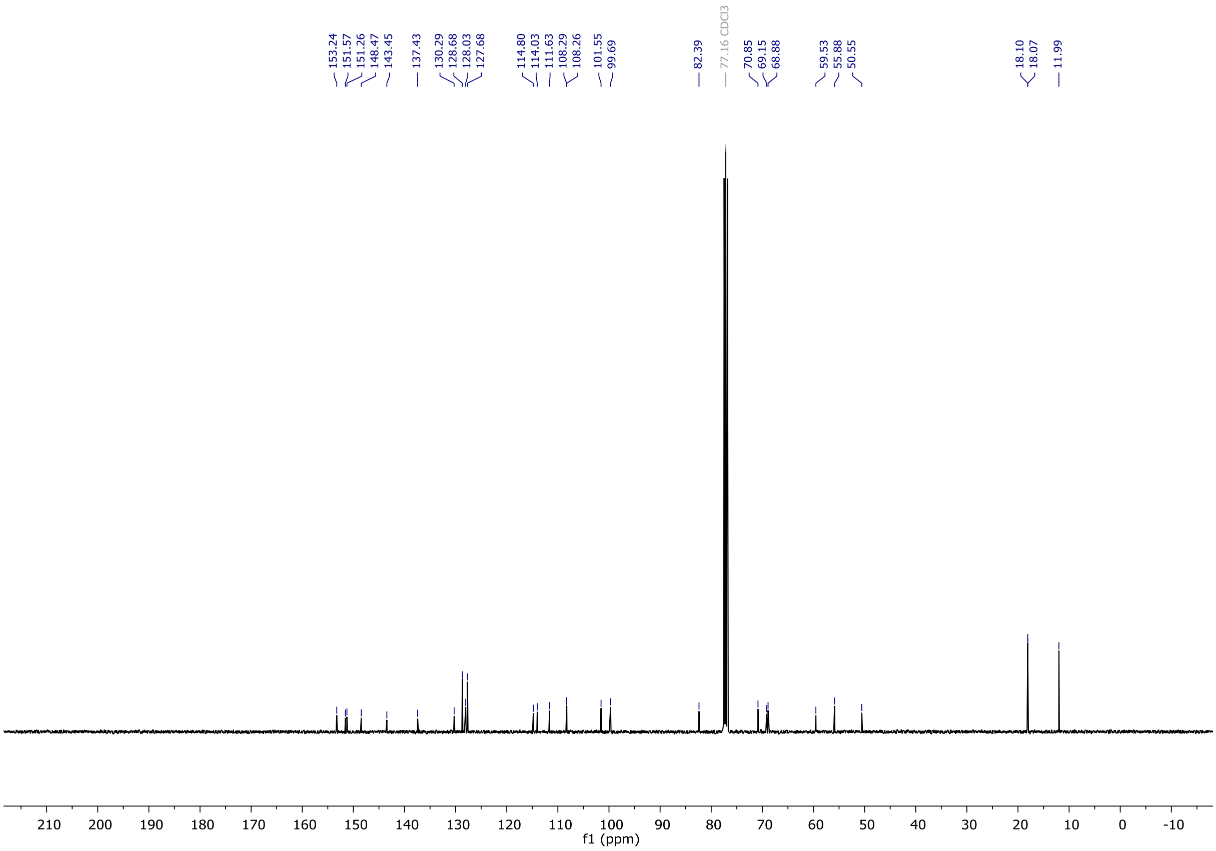

^{1}H NMR (400 MHz, $CDCl_3$) of epoxide 2-*epi*-**40**:

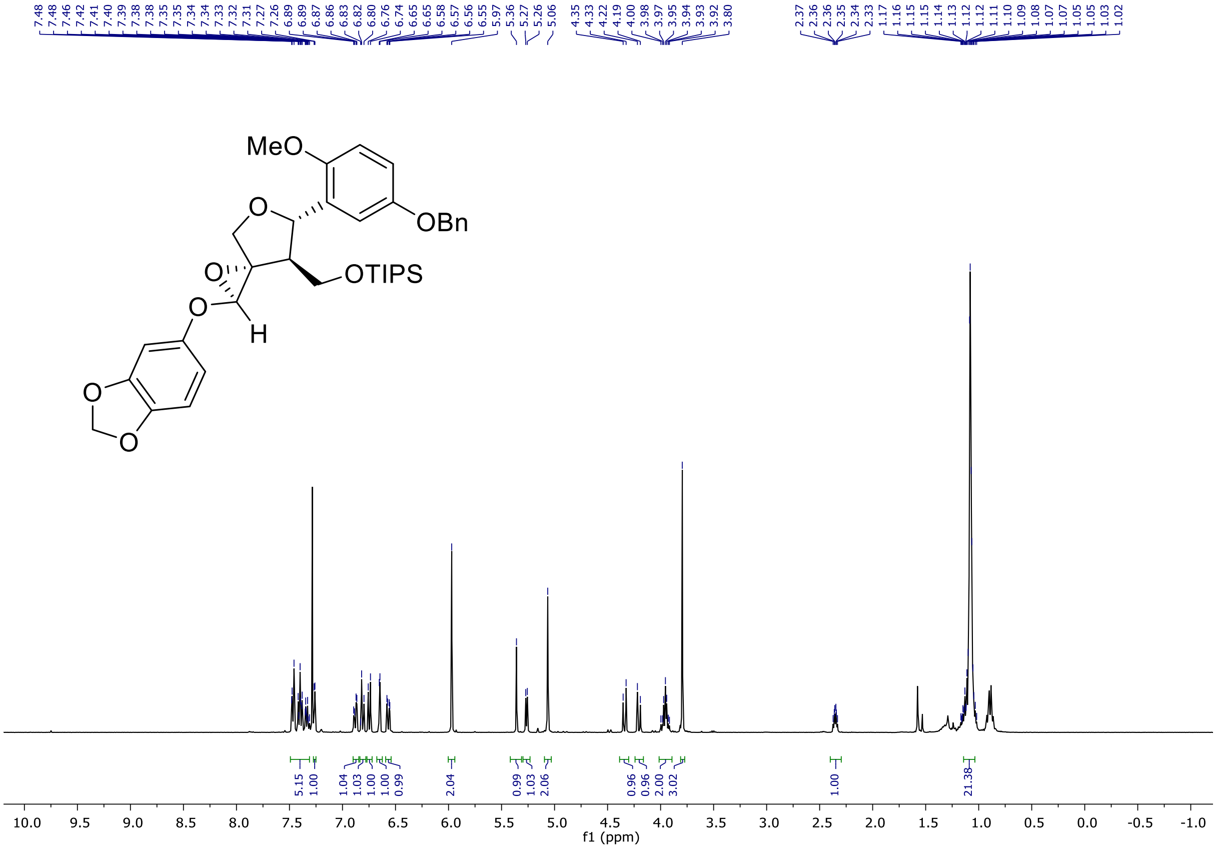

^{13}C NMR (101 MHz, $CDCl_3$) of epoxide 2-*epi*-**40:**

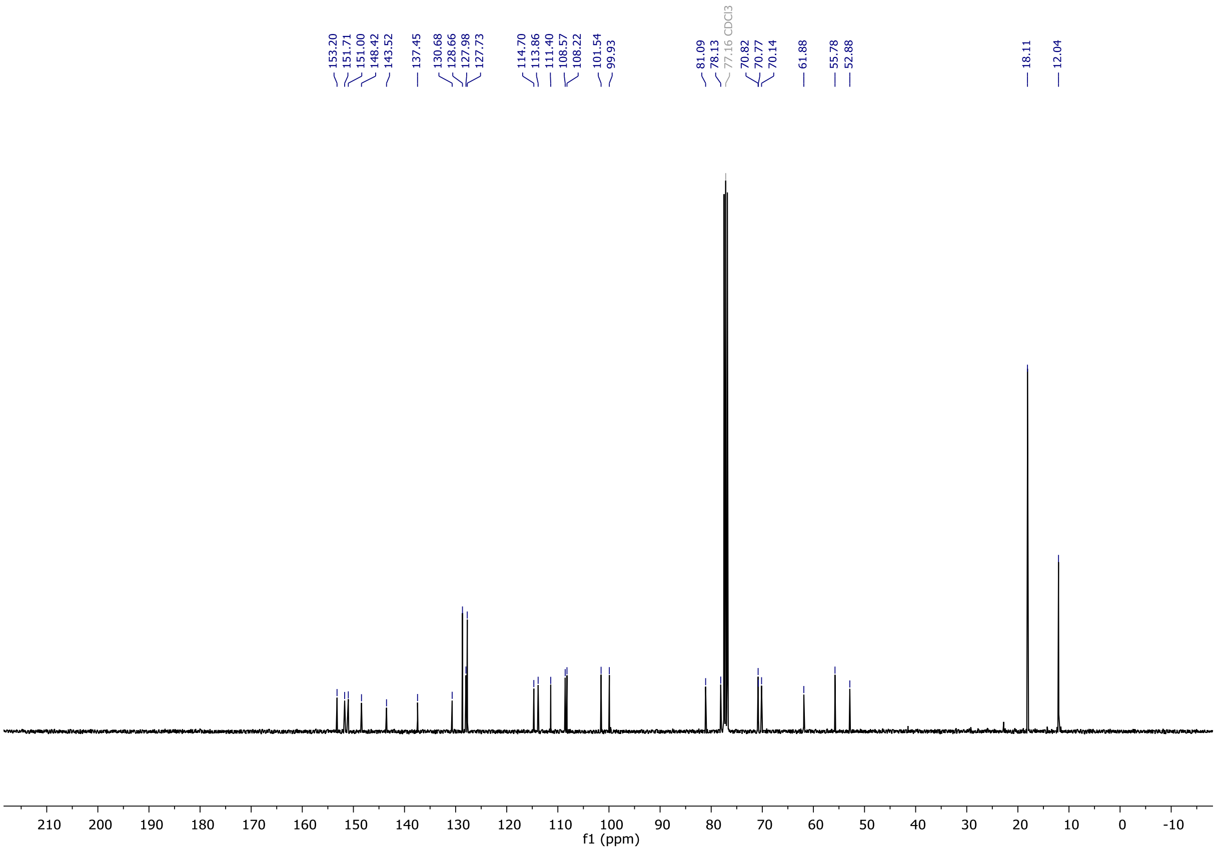

^{1}H NMR (400 MHz, $CDCl_3$) of epoxide **40**:

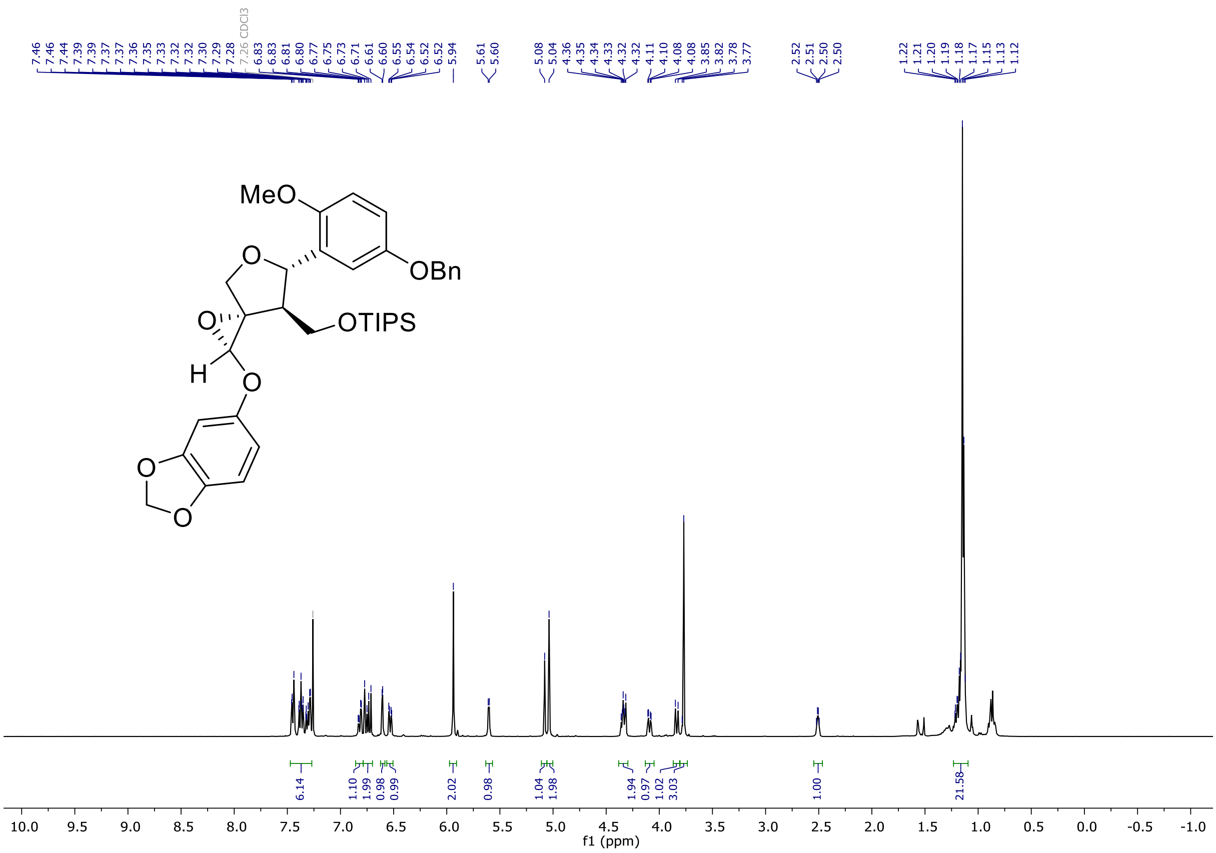

^{13}C NMR (101 MHz, $CDCl_3$) of epoxide **40**:

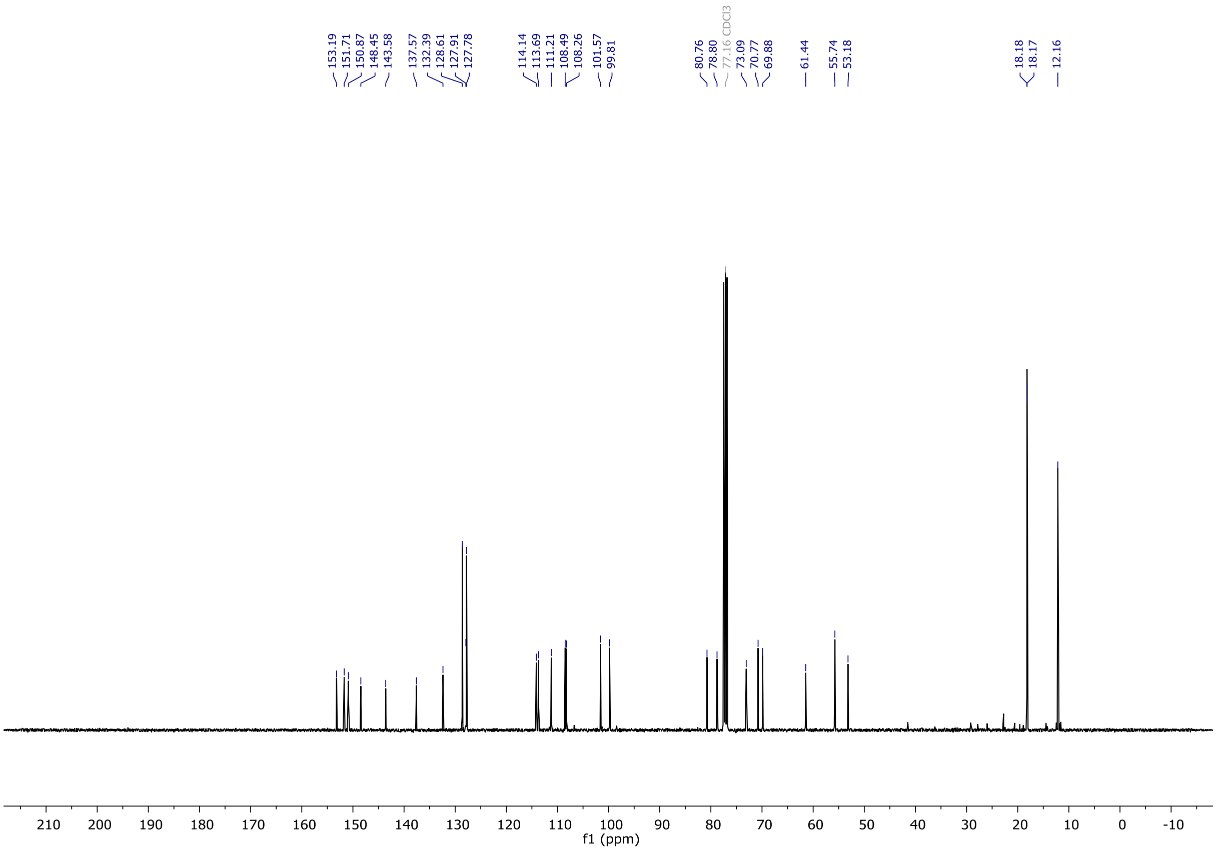

^{1}H NMR (400 MHz, $CDCl_3$) of acetal 2-*epi*-**39**:

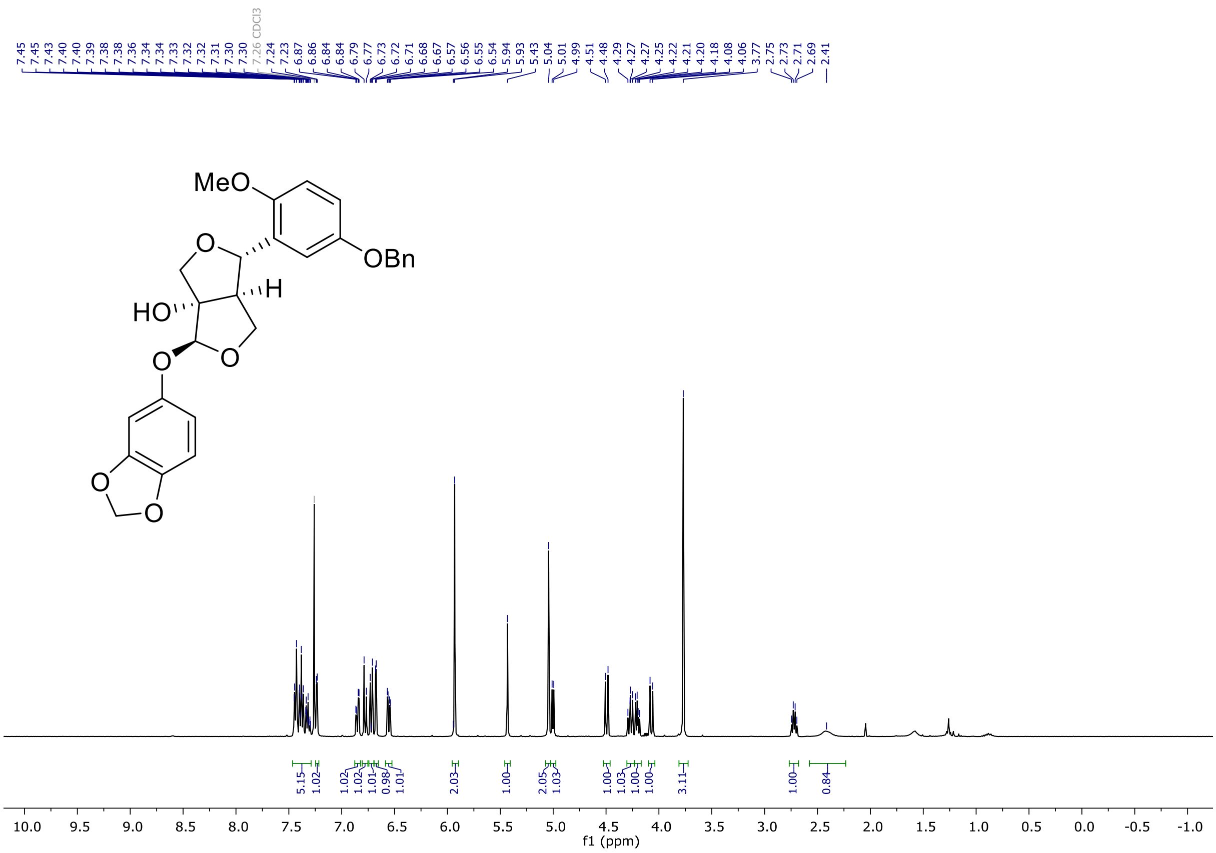

^{13}C NMR (101 MHz, $CDCl_3$) of acetal 2-*epi*-**39**:

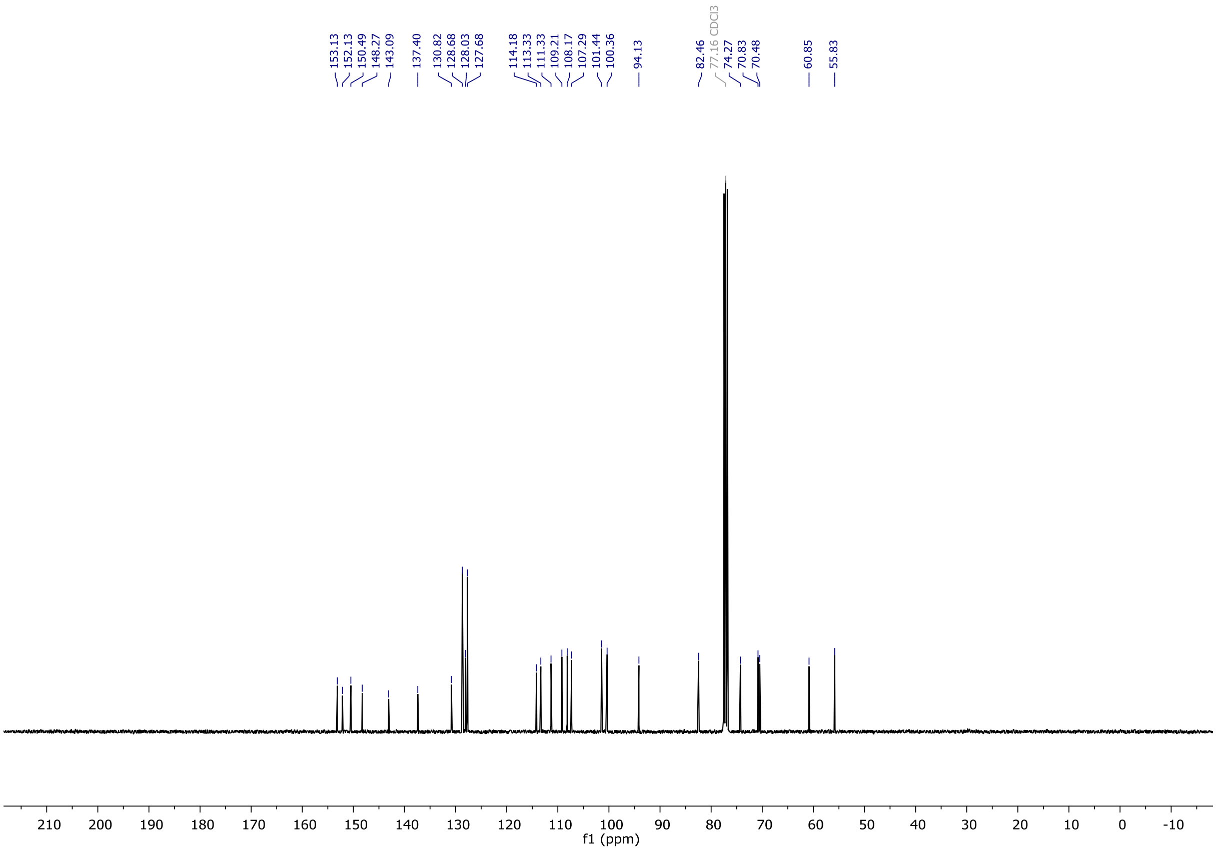

^{1}H NMR (400 MHz, $CDCl_3$) of fluorides **SI-55**:

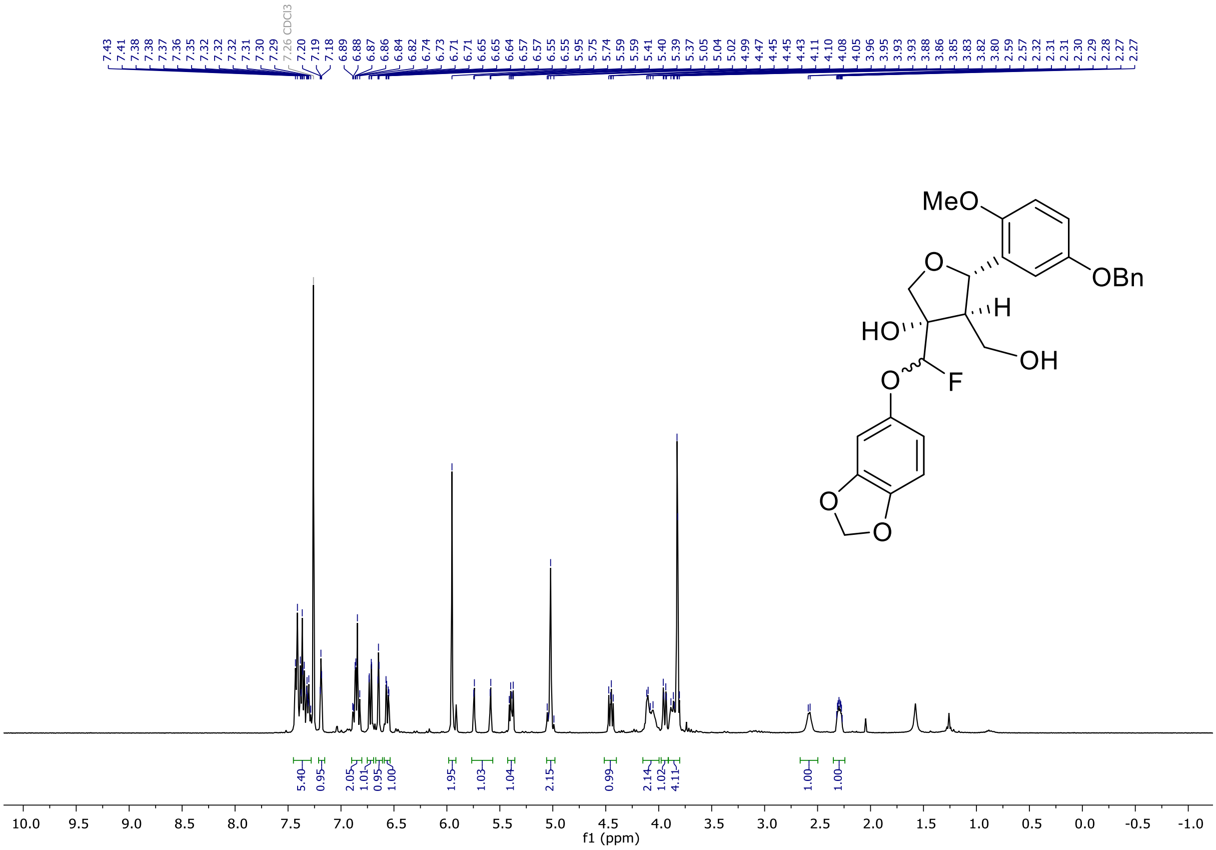

^{19}F NMR (376 MHz, $CDCl_3$) of fluorides **SI-55:**

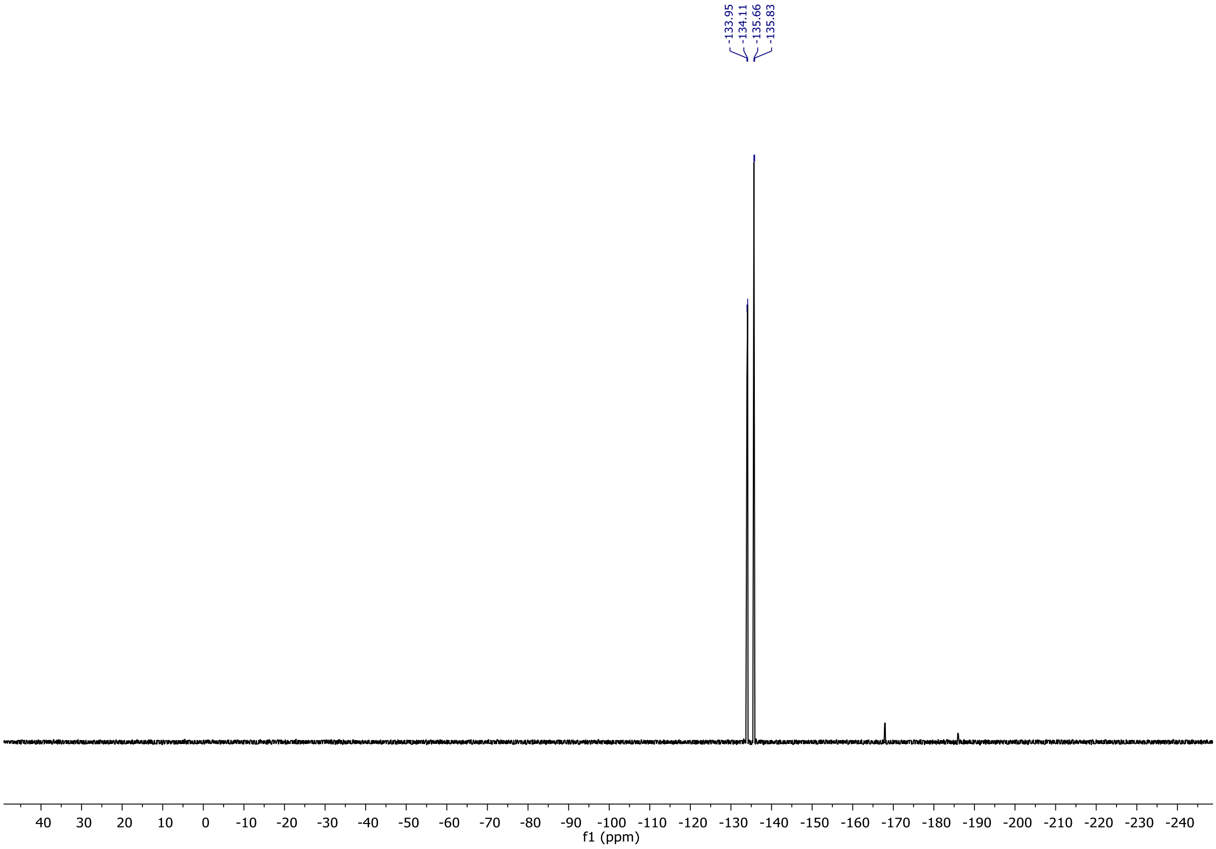

Supplement: Supplementary file 1 [file ja5c16676_si_001.pdf]
